# Supplementary material for: A practical preparation of bicyclic boronates via metal-free heteroatom-directed alkenyl sp2-C‒H borylation
Source: Commun Chem. 2023 Aug 23;6:176. doi: 10.1038/s42004-023-00976-5 (PMC10447525; doi:10.1038/s42004-023-00976-5)
Supplement: Supplementary file 2 — Supplementary Information [file 42004_2023_976_MOESM2_ESM.pdf]

Supplementary Information

A practical preparation of bicyclic boronates via metal-free heteroatom-directed alkenyl *sp*^{2}-C–H borylation

Pei-Ying Peng,[1] Gui-Shan Zhang,[1] Mei-Ling Gong,[1] Jian-Wei Zhang,[1] Xi-Liang, Liu,[1] Dingding Gao,[1] Guo-Qiang Lin,[1] Qing-Hua Li,[1,*] & Ping Tian,[1,*]

[1]The Research Center of Chiral Drugs,
Shanghai Frontiers Science Center for TCM Chemical Biology,
Innovation Research Institute of Traditional Chinese Medicine,
Shanghai University of Traditional Chinese Medicine,
1200 Cailun Road, Shanghai 201203, China.

*Email: qinghuali@shutcm.edu.cn
tianping@shutcm.edu.cn

Table of Contents

Supplementary Methods **S2**

General information S2

The preparation of substrates S3

Experimental procedures and characterization of products S15

10 mmol-Scale experiment S28

Supplementary Crystallographic Data **S34**

Supplementary References **S47**

Supplementary Methods

General information

Reactions were monitored by thin layer chromatography using UV light to visualize the course of reaction. Purification of reaction products was carried out by flash chromatography on silica gel (300-400 mesh). Chemical yields referred to as pure isolated substances. ^{1}H, ^{13}C and ^{19}F NMR spectra were obtained using a Bruker 300, 400 or 600 MHz spectrometer. Chemical shifts for ^{1}H, ^{13}C NMR spectra were reported in ppm from $CDCl_3$, DMSO-d_6 with the solvent resonance as the internal standard. The following abbreviations were used to designate chemical shift multiplicities: s = singlet, d = doublet, t = triplet, m = multiplet, br = broad. High resolution mass spectra were acquired by Agilent 6545 Accurate-Mass Q-TOF LC/MS System. Optical rotations were measured on Rudolph Research Analytical AUTOPOL IV Automatic Polarimeter. Infrared spectra were recorded on a Shimadzu Fourier Transform Infrared Spectrophotometer IRAffinity-1. Melting points were determined on a digital melting point apparatus and temperatures were uncorrected. Enantiomeric excess was determined by chiral HPLC analysis on Agilent 1260 Infinity II LC System. X-ray structure was determined on a Bruker D8 Venture X-ray Diffraction meter.

Unless otherwise indicated, all starting materials purchased from commercial suppliers were used without further purification. All solvents were dried before use following the standard procedures. Unless otherwise noted, experiments involving moisture and/or air sensitive components were performed in nitrogen.

The preparation of substrates

The known compounds **1aa**[1], **1ab**[2], **1ad**[3], **1ae**[4], **1af**[5], **1ai**[6], **1aj**[7], **1al**[8], **1aq**[9], **1at**[10], **1aw**[11], **1ay**[12], **1bc**[13], **1bd**[14], **1be**[15], **1bh**[16], **1bk**[17], **1bn**[18], **1bo**[19], **1bp**[20], **1bq**[21], **1br**[22], **1bs**[23], **1bt**[24], and **1by**[25] were prepared according to the literature.

The preparation of compounds 1ag, 1aj, 1ak, 1al, 1am, 1an, 1ao, 1ap, 1ar, 1as, 1au, 1ax, 1bb, 1bb', 1be, 1bf, 1bk, 1bl, and 1bw.

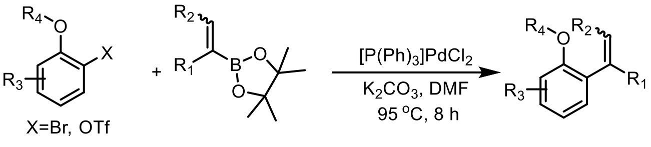

General procedure: Aryl bromide (**31**, 1.0 equiv), isopropenyl boronic acid pinacol ester (**32**, 1.2 equiv), and $(PPh_3)_2PdCl_2$ (5 mol %) were added to 50 mL Schlenk tube. After 3 evacuation-backfill cycles with nitrogen, *N*,*N*-dimethylformamide and K_2CO_3 (2.0 equiv, 2.0 M aq) were added and the reaction mixture was then stirred at 95 °C for 8 h. The reaction mixture was allowed to cool to room temperature, then transferred to the known compounds, a separatory funnel, and extracted with ethyl acetate (EA, 30 mL×3). The combined organic layers were washed with brine, dried with Na_2SO_4, filtered, and concentrated in vacuo. The crude mixture was purified by silica gel column chromatography to afford the desired *o*-isopropenylanisole.

1-Fluoro-3-methoxy-2-(prop-1-en-2-yl)benzene (1ag)

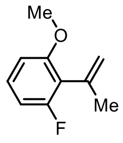

1ag

Following the general procedure on 5.0 mmol scale, the substrate **1ag** was obtained as colorless oil. Eluent: PE, 709 mg, 85% yield; **^{1}H NMR** (600 MHz, $CDCl_3$) δ 7.17 (q, *J* = 7.7 Hz, 1H), 6.71 (t, *J* = 8.7 Hz, 1H), 6.68 (d, *J* = 8.4 Hz, 1H), 5.37 (s, 1H), 4.99 (s, 1H), 3.83 (s, 3H), 2.06 (s, 3H**^{13}C NMR** (151 MHz, $CDCl_3$) δ 160.7, 159.1, 157.8, 157.8, 136.4, 128.1, 128.0, 120.6, 120.5, 117.3, 108.2, 108.0, 106.3, 106.3, 56.0, 23.3**HRMS (ESI)**: Exact mass calcd for $C_{10}H_{12}FO[M+H]^+$: 167.0872, Found: 167.0871.

4-(tert-butyl)-1-Methoxy-2-(prop-1-en-2-yl)benzene (1ak)

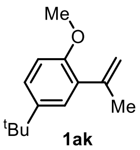

1ak

Following the general procedure on 4.0 mmol scale, the substrate **1ak** was obtained as colorless oil. Eluent: PE, 457 mg, 56% yield; **^{1}H NMR** (600 MHz, $CDCl_3$) δ 7.27 (d, *J* = 8.4 Hz, 1H), 7.22 (s, 1H), 6.82 (d, *J* = 8.9 Hz, 1H), 5.16 (s, 1H), 5.06 (s, 1H), 3.83 (s, 3H), 2.15 (s, 3H), 1.33 (s, 9H); **^{13}C NMR** (151 MHz,

$CDCl_3$) δ 154.3, 144.9, 143.1, 132.1, 126.5, 124.8, 114.8, 110.3, 55.5, 34.0, 31.5, 23.2; **HRMS (ESI)**: Exact mass calcd for [M+H] $C_{14}H_{21}O[M+H]^+$: 205.1596 Found: 205.1597.

2,5-dimethoxy-4-(prop-1-en-2-yl)-1,1'-biphenyl (1am)

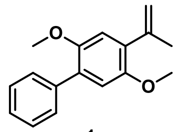

1am

Following the general procedure on 5.0 mmol scale, the substrate **1am** was obtained as white solid. Mp: 113–115 °C; Eluent: PE/EA (5:1), 0.53 g, 42% yield; **^{1}H NMR** (600 MHz, $CDCl_3$) δ 7.55 (dd, J = 8.2, 1.3 Hz, 2H), 7.42 (t, J = 7.4 Hz, 2H), 7.35 – 7.34 (m, 1H), 6.86 (d, J = 5.7 Hz, 2H), 5.20 (dd, J = 2.2, 1.5 Hz, 1H), 5.15 (dt, J = 2.1, 1.0 Hz, 1H), 3.83 (s, 3H), 3.76 (s, 3H), 2.17 (s, 3H).; **^{13}C NMR** (151 MHz, $CDCl_3$) δ 150.8, 150.3, 144.1, 138.4, 132.5, 130.1, 129.4, 128.0, 127.0, 115.3, 114.3, 113.6, 56.4, 56.3, 23.2. **HRMS (ESI)**: Exact mass calcd for $C_{17}H_{18}O_2Na$ $[M+Na]^+$: 277.1199, Found: 277.1205.

4-(4-methoxy-3-(prop-1-en-2-yl)phenoxy)Benzonitrile (1an)

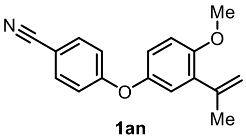

1an

Following the general procedure on 10.0 mmol scale, the substrate **1an** was obtained as white solid. Mp: 113–115 °C; Eluent: PE/EA (5:1), 1.0 g, 38% yield; **^{1}H NMR** (600 MHz, MeOD) δ 7.66 (d, J = 8.9 Hz, 2H), 7.03 – 6.99 (m, 3H), 6.98 – 6.96 (m, 1H), 6.90 (d, J = 2.9 Hz, 1H), 5.10 (s, 1H), 5.01 (s, 1H), 3.83 (s, 3H), 2.08 (s, 3H); **^{13}C NMR** (151 MHz, MeOD) δ 164.1, 155.6, 149.1, 144.8, 135.8, 135.4, 122.6, 121.3, 119.8, 118.4, 116.1, 113.6, 106.2, 56.4, 23.2; **HRMS (ESI)**: Exact mass calcd for $C_{17}H_{16}NO_2$ $[M+H]^+$: 266.1185, Found: 266.1180.

4-Methoxy-3-(prop-1-en-2-yl)phenol (1ao)

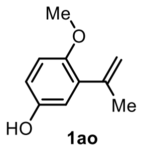

1ao

Following the general procedure on 10.0 mmol scale, the substrate **1ao** was obtained as colorless oil, Eluent: PE/EA (5:1), 1.3 g, 80% yield; **^{1}H NMR** (600 MHz, $CDCl_3$) δ 6.76 (d, J = 5.3 Hz, 1H), 6.73 – 6.69 (m, 2H), 5.14 (s, 1H), 5.07 (s, 1H), 3.78 (d, J = 1.1 Hz, 3H), 2.10 (s, 3H); **^{13}C NMR** (151 MHz, $CDCl_3$) δ 150.8, 149.2, 143.7, 133.9, 116.5, 115.3, 114.2, 112.5, 56.3, 23.1; **HRMS (ESI)**: Exact mass calcd for $C_{10}H_{12}O_2[M+H]^+$: 165.0913, Found: 165.0911.

***N*-(4-methoxy-3-(prop-1-en-2-yl)phenyl)Pivalamide (1ap)**

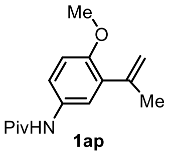

1ap

Following the general procedure on 13.0 mmol scale, the substrate **1ap** was obtained as light yellow oil, Eluent: PE/EA (10:1), 2.5 g, 78% yield; **^{1}H NMR** (600 MHz, MeOD) δ 7.37 (d, J = 8.7 Hz, 1H), 7.28 (s, 1H), 6.90 (d, J = 8.6 Hz, 1H), 5.08 (s, 1H), 5.01 (s, 1H), 4.61 (s, 1H), 3.79 (s, 3H), 2.08 (s, 3H), 1.28 (s, 9H);

^{13}C NMR (151 MHz, MeOD) 179.9, 155.1, 145.5, 134.0, 132.4, 124.6, 123.3, 115.5, 112.2, 56.2, 40.3, 27.9, 23.4; **HRMS (ESI)**: Exact mass calcd for $C_{15}H_{22}NO_2[M+H]^+$: 248.1651, Found: 248.1647.

Ethyl 2-(4-methoxy-3-(prop-1-en-2-yl)phenyl)acetate (1ar)

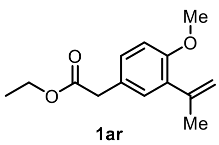

1ar

Following the general procedure on 2.7 mmol scale, the substrate **1ar** was obtained as colorless oil. Eluent: PE/EA (10:1), 0.3 g, 47% yield; **^{1}H NMR** (600 MHz, $CDCl_3$) δ 7.13 (d, *J* = 7.5 Hz, 1H), 6.89 – 6.79 (m, 2H), 5.14 (s, 1H), 5.05 (s, 1H), 4.17 (q, *J* = 7.1 Hz, 2H), 3.83 (s, 3H), 3.60 (s, 2H), 2.10 (s, 3H), 1.27 (t, *J* = 7.1 Hz, 3H); **^{13}C NMR** (151 MHz, $CDCl_3$) δ 171.6, 156.6, 143.9, 134.3, 131.5, 129.4, 121.3, 115.1, 111.8, 60.9, 55.4, 41.3, 23.1, 14.2; **HRMS (ESI)**: Exact mass calcd for $C_{14}H_{18}NaO_3[M+Na]^+$: 257.1154, Found: 257.1152.

4-(*tert*-Butyl)-1-methoxy-2-(prop-1-en-2-yl)benzene (1as)

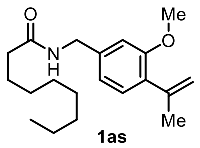

1as

Following the general procedure on 3.0 mmol scale, the substrate **1as** was obtained as colorless oil. Eluent: PE/EA (10:1), 0.5 g, 52% yield; **^{1}H NMR** (600 MHz, $CDCl_3$) δ 7.13 (d, *J* = 7.6 Hz, 1H), 6.81 (d, *J* = 7.5 Hz, 1H), 6.80 (s, 1H), 5.75 (s, 1H), 5.14 – 5.13 (m, 1H), 5.04 – 5.03 (m, 1H), 4.42 (d, *J* = 5.7 Hz, 2H), 3.82 (s, 3H), 2.27 – 2.17 (m, 2H), 2.09 (s, 3H), 1.68 – 1.63 (m, 2H), 1.34 – 1.17 (m, 10H), 0.87 (t, *J* = 7.0 Hz, 3H); **^{13}C NMR** (151 MHz, $CDCl_3$) δ 172.9, 156.8, 143.8, 138.8, 132.0, 129.5, 119.7, 115.2, 110.4, 55.5, 43.5, 36.8, 31.8, 29.3, 29.3, 29.1, 25.8, 23.1, 22.6, 14.1; **HRMS (ESI)**: Exact mass calcd for $C_{20}H_{32}NO_2[M+H]^+$: 318.2433, Found: 318.2431.

4-Fluoro-2-methoxy-1-(prop-1-en-2-yl)benzene (1au)

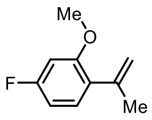

1au

Following the general procedure on 5.0 mmol scale, the substrate **1au** was obtained as colorless oil. Eluent: PE, 513 mg, 62% yield; **^{1}H NMR** (600 MHz, $CDCl_3$) δ 7.16 (t, *J* = 7.6 Hz, 1H), 6.64 (t, *J* = 9.4 Hz, 2H), 5.18 (s, 1H), 5.07 (s, 1H), 3.84 (s, 3H), 2.13 (s, 3H); **^{13}C NMR** (151 MHz, $CDCl_3$) δ 163.5, 161.9, 157.5, 157.4, 143.4, 129.9, 129.8, 128.3, 128.3, 115.3, 106.5, 106.3, 98.8, 98.6, 55.4, 23.1; **HRMS (ESI)**: Exact mass calcd for $C_{10}H_{12}FO[M+H]^+$: 167.0872, Found: 167.0865.

1,5-Difluoro-2-methoxy-3-(prop-1-en-2-yl)benzene (1ax)

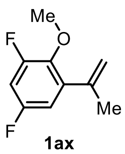

1ax

Following the general procedure on 10.0 mmol scale, the substrate **1ax** was obtained as colorless oil. Eluent: PE, 1.2 g, 67% yield; **^{1}H NMR** (600 MHz, $CDCl_3$) δ 6.76 (d, *J* = 19.2 Hz, 1H), 6.71 (t, *J* = 8.5 Hz, 1H), 5.21 (s, 1H), 5.14 (s, 1H),

3.82 (s, 3H), 2.11 (s, 3H); **^{13}C NMR** (151 MHz, $CDCl_3$) δ 158.4, 158.3, 156.8, 156.7, 156.5, 156.4, 154.8, 154.7, 141.8, 141.5, 141.4, 139.3, 139.2, 116.7, 111.0, 110.9, 103.7, 103.6, 103.4, 61.4, 61.4, 23.0; **^{19}F NMR** (565 MHz, $CDCl_3$) δ -116.85 (d, *J* = 4.1 Hz), -126.78 (d, *J* = 4.0 Hz); **HRMS (ESI)**: Exact mass calcd for $C_{10}H_{10}F_2NaO$ $[M+Na]^+$: 207.0597, Found: 207.0594.

(8*R*,9*S*,13*S*,14*S*)-3-Hydroxy-13-methyl-2-(prop-1-en-2-yl)-6,7,8,9,11,12,13,14,15,16-decahydro-17H-cyclopenta[a]phenanthren-17-one (1bb')

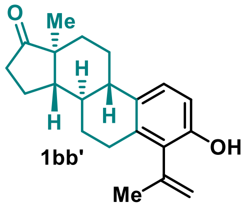

1bb'

Following the general procedure on 2.0 mmol scale, the substrate **1bb'** was obtained as white solid; Mp: 231–233 °C; Eluent: PE/EA (5:1), 155 mg, 25% yield; **^{1}H NMR** (600 MHz, $CDCl_3$) δ 7.15 (d, *J* = 8.5 Hz, 1H), 6.80 (d, *J* = 8.5 Hz, 1H), 5.52 (d, *J* = 18.9 Hz, 1H), 5.22 (d, *J* = 101.7 Hz, 1H), 5.03 (d, *J* = 18.9 Hz, 1H), 2.81 (m, 2H), 2.51 (dd, *J* = 19.1, 8.6 Hz, 1H), 2.43 – 2.37 (m, 1H), 2.26 (m, 1H), 2.19 – 2.10 (m, 1H), 2.07 – 1.97 (m, 4H), 1.96 – 1.95 (m, 1H), 1.60 (s, 3H), 1.54 – 1.48 (m, 3H), 1.43 – 1.37 (m, 1H), 0.92– 0.91(m, 3H). **^{13}C NMR** (151 MHz, $CDCl_3$) δ 221.0, 149.4, 141.7, 141.3, 134.1, 131.9, 131.7, 128.8, 125.3, 125.1, 118.7, 118.3, 112.4, 50.5, 50.4, 48.01, 44.4, 44.1, 38.0, 37.8, 31.6, 27.0, 26.8, 26.5, 26.4, 26.0, 23.4, 23.0, 21.6, 13.9. **HRMS (ESI)**: Exact mass calcd for $C_{21}H_{26}NaO_2$$[M+Na]^+$: 333.1830, Found: 333.1828.

(8*R*,9*S*,13*S*,14*S*)-2-Methoxy-13-methyl-1-(prop-1-en-2-yl)-6,7,8,9,11,12,13,14,15,16-decahydro-17H-cyclopenta[a]phenanthren-17-one (1bb)

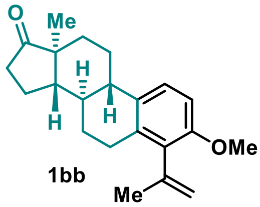

1bb

Following the general procedure on 6.0 mmol scale, the substrate **1bb** was obtained as white solid; Mp: 213–215 °C; Eluent: PE/EA (10:1), 0.5 g, 26% yield; **^{1}H NMR** (600 MHz, $CDCl_3$) δ 7.20 (d, *J* = 8.6 Hz, 1H), 6.76 (d, *J* = 8.6 Hz, 1H), 5.29 (s, 1H), 4.79 (s, 1H), 3.80 (s, 3H), 3.10 – 2.87 (m, 1H), 2.73 – 2.57 (m, 1H), 2.50 (dd, *J* = 19.1, 8.6 Hz, 1H), 2.44 – 2.38 (m, 1H), 2.29 – 2.23 (m, 1H), 2.19 – 2.09 (m, 1H), 2.09 – 2.04 (m, 1H), 2.00 – 1.95 (m, 5H), 1.66 – 1.46 (m, 6H), 1.42 (br, 1H), 0.91 (s, 3H). **^{13}C NMR** (151 MHz, $CDCl_3$) δ 221.0, 154.4, 142.5, 134.7, 132.4, 132.0, 124.5, 115.1, 108.2, 55.8, 50.5, 48.0, 44.5, 37.9, 35.9, 31.6, 27.9, 26.8, 26.1, 23.3, 21.6, 13.8. **HRMS (ESI)**: Exact mass calcd for $C_{22}H_{28}NaO_2$$[M+Na]^+$: 347.1987, Found: 347.1984.

1-Isopropoxy-2-(prop-1-en-2-yl)benzene (1be)

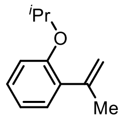

1be

Following the general procedure on 5.0 mmol scale, the substrate **1be** was obtained as colorless oil. Eluent: PE, 634 mg, 72% yield; **^{1}H NMR** (600 MHz, $CDCl_3$) δ 7.21 (t, *J* = 7.6 Hz, 2H), 6.92 – 6.88 (m, 2H), 5.11 (s, 1H), 5.07 (s, 1H),

4.58 – 4.54 (m, 1H), 2.15 (s, 3H), 1.34 (d, *J* = 6.1 Hz, 6H); **^{13}C NMR** (151 MHz, $CDCl_3$) δ 154.7, 144.8, 133.6, 129.7, 128.1, 120.2, 114.9, 113.5, 70.0, 23.1, 22.0. **HRMS (ESI)**: Exact mass calcd for $C_{12}H_{17}O[M+H]^+$: 177.1274, Found: 177.1270.

1-(*tert*-Butoxy)-2-(prop-1-en-2-yl)benzene (1bf)

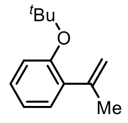

1bf

Following the general procedure on 1.0 mmol scale, the substrate **1bf** was obtained as colorless oil. Eluent: PE, 143 mg, 75% yield; **^{1}H NMR** (600 MHz, $CDCl_3$) δ 7.22 – 7.20 (m, 1H), 7.18 – 7.15 (m, 1H), 7.03 – 7.00 (m, 2H), 5.10 (s, 1H), 5.06 (s, 1H), 2.15 (s, 3H), 1.35 (s, 9H); **^{13}C NMR** (151 MHz, $CDCl_3$) δ 153.2, 145.8, 138.8, 129.6, 127.5, 123.2, 122.9, 115.1, 79.7, 29.1, 23.1; **HRMS (ESI)**: Exact mass calcd for $C_{13}H_{19}O[M+H]^+$: 191.1436, Found: 191.1432.

1-Phenoxy-2-(prop-1-en-2-yl)benzene (1bg)

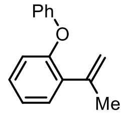

1bg

Following the general procedure on 4.0 mmol scale, the substrate **1bg** was obtained as colorless oil. Eluent: PE, 0.7 g, 83% yield; **^{1}H NMR** (600 MHz, $CDCl_3$) δ 7.32 (d, *J* = 7.5 Hz, 1H), 7.28 (t, *J* = 7.8 Hz, 2H), 7.21 (t, *J* = 7.6 Hz, 1H), 7.10 (t, *J* = 7.4 Hz, 1H), 7.03 (t, *J* = 7.4 Hz, 1H), 6.93 (d, *J* = 7.9 Hz, 2H), 6.90 (d, *J* = 8.2 Hz, 1H), 5.11 (s, 2H), 2.11 (s, 3H); **^{13}C NMR** (151 MHz, $CDCl_3$) δ 157.9, 153.4, 142.8, 135.7, 129.9, 129.6, 128.4, 123.8, 122.5, 120.1, 117.8, 115.9, 23.2; **HRMS (ESI)**: Exact mass calcd for $C_{15}H_{15}O[M+H]^+$: 211.1123, Found: 211.1118;

7-(Prop-1-en-2-yl)-1H-indole (1bl)

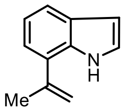

1bl

Following the general procedure on 20.0 mmol scale, the substrate **1bl** was obtained as yellow oil. Eluent: PE, 2.5 g, 80% yield; **^{1}H NMR** (600 MHz, $CDCl_3$) δ 8.49 (s, 1H), 7.64 (d, *J* = 6.1 Hz, 1H), 7.25 – 7.23 (m, 1H), 7.20 (m, 2H), 6.64 (d, *J* = 3.8 Hz, 1H), 5.44 (s, 1H), 5.42 (s, 1H), 2.31 (s, 3H); **^{13}C NMR** (151 MHz, $CDCl_3$) δ 142.8, 133.1, 128.2, 126.3, 124.1, 119.8, 119.7, 113.6, 102.9, 23.6; **HRMS (ESI)**: Exact mass calcd for $C_{11}H_{10}N[M-H]^-$: 156.0813, Found: 156.0812.

1-(Prop-1-en-2-yl)-9H-carbazole (1bm)

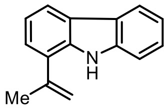

1bm

Following the general procedure on 15.0 mmol scale, the substrate **1bm** was obtained as white solid, Mp: 125–126 ^{o}C; Eluent: PE, 2.6 g, 83% yield; **^{1}H NMR** (600 MHz, $CDCl_3$) δ 8.37 (s, 1H), 8.08 (d, *J* = 7.8 Hz, 1H), 8.00 (d, *J* = 7.7 Hz, 1H), 7.45 (t, *J* = 7.7 Hz, 1H), 7.42 (d, *J* = 7.8 Hz, 1H), 7.35 (d, *J* = 7.4 Hz, 1H),

7.27 – 7.21 (m, 2H), 5.47 (s, 1H), 5.45 (s, 1H), 2.29 (s, 3H); **^{13}C NMR** (151 MHz, $CDCl_3$) δ 142.7, 139.3, 136.7, 125.8, 123.7, 123.6, 123.5, 120.3, 119.4, 119.3, 119.2, 114.3, 110.6, 23.7; **HRMS (ESI)**: Exact mass calcd for $C_{15}H_{14}N[M+H]^+$: 208.1126, Found: 208.1119.

4-(*tert*-Butyl)-1-Methoxy-2-(prop-1-en-2-yl)benzene (1bx)

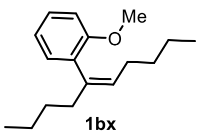

1bx

Following the general procedure on 2.4 mmol scale, the substrate **1bx** was obtained as colorless oil. Eluent: PE, 0.4 g, 67% yield; **^{1}H NMR** (600 MHz, $CDCl_3$) δ 7.22 (t, *J* = 7.8 Hz, 1H), 6.97 (d, *J* = 7.3 Hz, 1H), 6.94 – 6.86 (m, 2H), 5.47 (t, *J* = 7.2 Hz, 1H), 3.79 (s, 3H), 2.29 (t, *J* = 7.0 Hz, 2H), 1.79 (q, *J* = 7.2 Hz, 2H), 1.40 – 1.18 (m, 8H), 0.84 (t, *J* = 6.8 Hz, 3H), 0.80 (t, *J* = 7.1 Hz, 3H); **^{13}C NMR** (151 MHz, $CDCl_3$) δ 156.6, 138.3, 130.5, 130.4, 127.7, 127.6, 120.1, 110.6, 55.4, 37.8, 32.0, 30.3, 28.7, 22.3, 22.2, 13.9; **HRMS (ESI)**: Exact mass calcd for $C_{17}H_{25}O[M-H]^-$: 245.1905, Found: 245.1906.

4-Methoxy-3-(prop-1-en-2-yl)benzaldehyde (1fa)

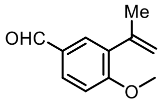

1fa

Following the general procedure on 5.0 mmol scale, the substrate **1fa** was obtained as colorless oil. Eluent: PE/EA (20:1), 176 mg, 20% yield; **^{1}H NMR** (600 MHz, MeOD) δ 9.83 (s, 1H), 7.84 (d, *J* = 8.5 Hz, 1H), 7.70 (s, 1H), 7.14 (d, *J* = 8.5 Hz, 1H), 5.16 (s, 1H), 5.05 (s, 1H), 3.92 (s, 3H), 2.09 (s, 3H); **^{13}C NMR** (151 MHz, MeOD) δ 191.60, 162.04, 143.26, 133.34, 131.45, 130.00, 129.61, 115.06, 110.73, 54.96, 21.76; **HRMS (ESI)**: Exact mass calcd for $C_{11}H_{13}O_2$ $[M+H]^+$: 177.0915, Found: 177.0912.

4-Methoxy-4-(prop-1-en-2-yl)benzonitrile (1fb)

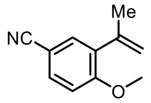

1fb

Following the general procedure on 5.0 mmol scale, the substrate **1fb** was obtained as colorless oil. Eluent: PE/EA (20:1), 450 mg, 52% yield; **^{1}H NMR** (400 MHz, $CDCl_3$) δ 7.54 (d, *J* = 8.5, 1H), 7.45 (s, 1H), 6.91 (d, *J* = 8.6 Hz, 1H), 5.22 – 5.17 (m, 1H), 5.09 – 5.04 (m, 1H), 3.88 (s, 3H), 2.07 (s, 3H); **^{13}C NMR** (101 MHz, $CDCl_3$) δ 160.13, 142.40, 134.09, 133.11, 133.09, 119.31, 116.82, 111.24, 103.95, 55.83, 22.81; **HRMS (ESI)**: Exact mass calcd for $C_{11}H_{15}N_2O$ $[M+NH_4]^+$: 191.1185, Found: 191.1180.

4-Methoxy-3-(prop-1-en-2-yl)pyridine (1fc)

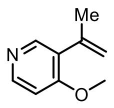

1fd

Following the general procedure on 5.0 mmol scale, the substrate **1fd** was obtained as colorless oil. Eluent: PE/EA (10:1), 470 mg, 63% yield; **^{1}H NMR** (300 MHz, $CDCl_3$) δ 8.38 (d, *J* = 5.7 Hz, 1H), 8.29 (s, 1H), 6.76 (d, *J* = 5.7 Hz, 1H), 5.18 (s, 1H), 5.09 (s, 1H), 3.86 (s, 3H), 2.08 (s, 3H); **^{13}C NMR** (101

MHz, $CDCl_3$) δ 162.87, 150.49, 149.59, 140.71, 128.45, 116.63, 106.18, 55.32, 22.91; **HRMS (ESI)**: Exact mass calcd for $C_{12}H_{16}NO$ $[M+C_3H_5]^+$: 190.1227, Found: 190.1229.

2-(prop-1-en-2-yl)Aniline (1fd)

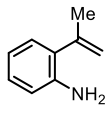

1fe

Following the general procedure on 5.0 mmol scale, the substrate **1fe** was obtained as colorless oil. Eluent: PE/EA (5:1), 386 mg, 58% yield; **^{1}H NMR** (600 MHz, $CDCl_3$) δ 7.09 (m, 2H), 6.77 (td, J = 7.5, 1.1 Hz, 1H), 6.73 (dd, J = 7.9, 1.0 Hz, 1H), 5.33 (m, 1H), 5.12 – 5.08 (m, 1H), 3.82 (s, 2H), 2.11 (s, 3H); **^{13}C NMR** (151 MHz, $CDCl_3$) δ 143.55, 142.90, 129.33, 128.30, 127.98, 118.28, 115.64, 115.39, 23.98; **HRMS (ESI)**: Exact mass calcd for $C_9H_{12}N$ $[M+H]^+$: 134.0969, Found: 134.0970.

The preparation of compounds 1ac, 1ah, 1bt, 1bu, 1bv, and 1by.

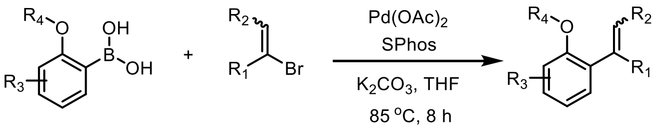

33 **34** **1**

General procedure: Aryl boronic acid (**31**, 1.0 equiv), isopropenyl bromide (**32**, 1.2 equiv), $Pd(OAc)_2$ (5 mol %), 2-dicyclohexylphosphino-2',6'-dimethoxybiphenyl (**SPhos**, 10 mol %) were added to 50 mL seal tube. After 3 evacuation-backfill cycles with nitrogen, THF and K_2CO_3 (2.0 equiv, 2.0 M aq) were added, and the reaction mixture was then stirred at 80 °C for 12 h. The reaction mixture was allowed to cool to room temperature, then transferred to a separatory funnel, and extracted with ethyl acetate (EA, 30 mL × 3). The combined organic layers were washed with brine, dried with Na_2SO_4, filtered, and concentrated in vacuo. The crude mixture was purified by silica gel column chromatography to afford the desired *o*-isopropenylanisole.

3-(2-Methoxyphenyl)but-3-en-1-yl methanesulfonate (1ac)

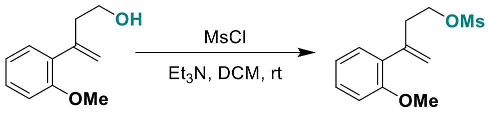

1ac' **1ac**

Following the general procedure on 4.0 mmol scale, the substrate 3-(2-methoxyphenyl)but-3-en-1-ol (**1ac'**) was obtained as colorless oil in 47% yield (340 mg). To a flame dried flask was added 3-(2-methoxyphenyl)but-3-en-1-ol (**1ac'**, 340 mg, 1.9 mmol), triethylamine (3.8 mmol, 0.5 mL, 2 equiv), and dichloromethane (5 mL). Methanesulfonyl chloride (3.8 mmol, 0.3 mL, 2 equiv) was adde

d at 0° C. The mixture was warmed to room temperature and stirred for 16 h. After completion of the reaction as indicated by TLC analysis, water (10 mL) was poured into, and the mixture was transferred to a separatory funnel. The organic layer was separated, and the aqueous layer was extracted with dichloromethane (3 x 5 mL). The combined organic layers were then washed with brine, dried with Na_2SO_4, filtered, and concentrated in vacuo. The crude mixture was purified by silica gel column chromatography to give the desired **1ac**. Eluent: PE/EA (5:1), colorless oil, 350 mg, 82% yield; **^{1}H NMR** (600 MHz, $CDCl_3$) δ 7.28 (t, J = 8.0 Hz, 1H), 7.16 (d, J = 7.3 Hz, 1H), 6.93 (t, J = 7.4 Hz, 1H), 6.88 (d, J = 8.2 Hz, 1H), 5.24 (s, 1H), 5.17 (s, 1H), 4.21 (t, J = 6.7 Hz, 2H), 3.83 (s, 3H), 2.96 (t, J = 6.8 Hz, 2H), 2.87 (s, 3H); **^{13}C NMR** (151 MHz, $CDCl_3$) δ 156.4, 143.6, 130.3, 130.2, 129.1, 120.8, 117.5, 110.6, 68.7, 55.4, 37.2, 35.6.; **HRMS (ESI)**: Exact mass calcd for $C_{12}H_{17}O_4S$ $[M+H]^+$: 257.0848, Found: 257.0839.

4-Fluoro-1-methoxy-2-(prop-1-en-2-yl)benzene (1ah)

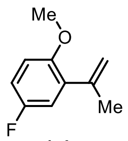

1ah

Following the general procedure on 5.0 mmol scale, the substrate **1ah** was obtained as colorless oil. Eluent: PE, 0.7 g, 84% yield; **^{1}H NMR** (600 MHz, $CDCl_3$) δ 6.92 (d, J = 3.1 Hz, 1H), 6.91 – 6.90 (m, 1H), 6.79 (dd, J = 9.8, 4.6 Hz, 1H), 5.17 (s, 1H), 5.09 (s, 1H), 3.81 (s, 3H), 2.10 (s, 3H); **^{13}C NMR** (151 MHz, $CDCl_3$) δ 157.7, 156.1, 152.8, 143.1, 134.1, 134.0, 116.2, 116.0, 115.9, 113.9, 113.8, 111.8, 111.8, 56.1, 22.9; **HRMS (ESI)**: Exact mass calcd for $C_{10}H_{12}FO$ $[M+H]^+$: 167.0872, Found: 167.0869.

4-(2-Methoxyphenyl)-3,6-dihydro-2H-pyran (1bu)

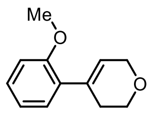

1bu

Following the general procedure on 10.0 mmol scale, the substrate 1bu was obtained as colorless oil. Eluent: PE, 1.2 g, 63% yield; **^{1}H NMR** (600 MHz, $CDCl_3$) δ 7.24 (t, J = 7.5 Hz, 1H), 7.16 (d, J = 7.3 Hz, 1H), 6.92 (t, J = 7.4 Hz, 1H), 6.87 (d, J = 8.2 Hz, 1H), 5.86 – 5.82 (m, 1H), 4.31 (d, J = 2.9 Hz, 2H), 3.90 (t, J = 5.5 Hz, 2H), 3.81 (s, 3H), 2.52 – 2.51 (m, 2H); **^{13}C NMR** (151 MHz, CDCl3) δ 156.7, 134.8, 131.2, 129.1, 128.3, 124.5, 120.6, 110.8, 65.7, 64.5, 55.3, 28.4; **HRMS (ESI)**: Exact mass calcd for $C_{12}H_{15}O_2$ $[M+H]^+$: 191.1072, Found: 191.1066.

(*Z*)-1-Methoxy-2-(1-(p-tolyl)prop-1-en-1-yl)benzene (1bv)

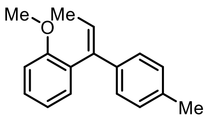

1bv

Following the general procedure on 5.0 mmol scale, the substrate **1bv** was obtained as colorless oil. Eluent: PE, 0.8 g, 67% yield; **^{1}H NMR** (600 MHz, $CDCl_3$) δ 7.33 (t, J = 7.8 Hz, 1H), 7.12 (d, J = 8.0 Hz, 2H), 7.09 (d, J = 7.4 Hz, 1H), 7.05 (d, J = 8.0 Hz, 2H), 7.00 (t, J = 7.3 Hz, 1H), 6.97 (d, J = 8.3 Hz, 1H), 6.27 (q, J = 6.8 Hz, 1H), 3.72 (s, 3H), 2.31 (s, 3H), 1.64 (d, J = 6.8 Hz, 3H); **^{13}C NMR** (151 MHz, $CDCl_3$) δ

157.2, 139.2, 138.5, 136.0, 131.6, 128.9, 128.7, 128.4, 126.0, 123.8, 120.6, 111.2, 55.7, 21.0, 15.5; **HRMS (ESI)**: Exact mass calcd for $C_{17}H_{19}O[M+H]^+$: 239.1436, Found: 239.1427.

(*Z*)-1-(2-Bromo-1-(*p*-tolyl)vinyl)-2-methoxybenzene (1bw)

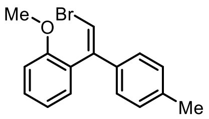

1bw

Following the general procedure on 2.0 mmol scale, the substrate **1bw** was obtained as yellow oil. Eluent: PE, 60 mg, 10% yield; **^{1}H NMR** (600 MHz, $CDCl_3$) δ 7.28 (t, *J* = 7.9 Hz, 1H), 7.25 (d, *J* = 6.7 Hz, 2H), 7.15 – 7.13 (m, 3H), 6.91 (t, *J* = 7.5 Hz, 1H), 6.86 (d, *J* = 8.2 Hz, 1H), 6.66 (s, 1H), 3.65 (s, 3H), 2.35 (s, 3H); **^{13}C NMR** (151 MHz, $CDCl_3$) δ 156.9, 143.4, 137.2, 136.6, 130.9, 130.4, 129.3, 128.9, 128.5, 120.4, 111.5, 106.3, 55.6, 21.3; **HRMS (ESI)**: Exact mass calcd for $C_{16}H_{16}OBr[M+H]^+$: 303.0385, Found: 303.0374.

The preparation of compounds 1az, 1ba, 1bi, and 1bj.

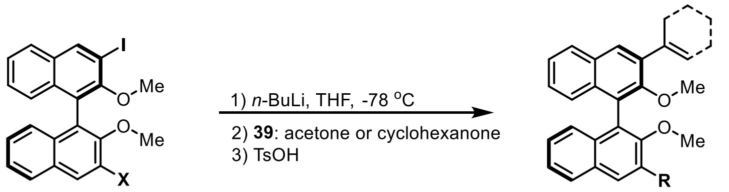

37, X = H, **39**: acetone
38, X = I, **39**: acetone
37, X = H, **39**: cyclohexanone

1ay, R = H
1az, R = isopropenyl
1bz, R = H

To a round bottom flask was added (*R*)-3-iodo-2,2'-dimethoxy-1,1'-binaphthyl (350 mg, 0.8 mmol) and tetrahydrofuran (4 mL). The solution was then cooled to –78 °C followed by the dropwise addition of 1.6 M ^{n}BuLi (0.6 mL, 1.2 equiv). After stirring at –78°C for 0.5 h, acetone (0.2 mL, 3.0 equiv) was added to the solution and the reaction mixture was allowed to warm to 0 °C to stir for one additional hour. Subsequently, the reaction was quenched by the saturated ammonium chloride solution, and the layers were separated. The aqueous layer was then extracted twice with EtOAc and the organic layers were combined, washed with brine, dried over sodium sulfate and concentrated. The residue was dissolved in dichloromethane (8 mL), followed by the addition of *p*-toluenesulfonic acid (0.3 g, 1.6 mmol), and stirred overnight at room temperature. The acid was removed by filtration through celite pad, and the filtration was concentrated and purified by column chromatography to give the desired product.

(*R*)-2,2'-Dimethoxy-3-(prop-1-en-2-yl)-1,1'-binaphthalene (1az)

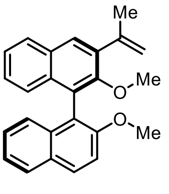

1az

Eluent: PE, white solid, 120 mg, 43% yield, Mp: 134–136 °C; **^{1}H NMR** (600 MHz, $CDCl_3$) δ 8.00 (d, *J* = 9.1 Hz, 1H), 7.89 – 7.85 (m, 2H), 7.83 (s, 1H), 7.47 (d, *J* = 9.0 Hz, 1H), 7.37 (t, *J* = 7.5 Hz, 1H), 7.32 (t, *J* = 7.4 Hz, 1H), 7.25 – 7.22 (m, 1H), 7.20 (t, *J* = 7.5 Hz, 1H), 7.15 (d, *J* = 8.5 Hz, 1H), 7.09 (d, *J* = 8.5 H

z, 1H), 5.35 (s, 1H), 5.26 (s, 1H), 3.80 (s, 3H), 3.41 (s, 3H), 2.28 (s, 3H); **^{13}C NMR** (151 MHz, CDCl$_3$) δ 154.9, 154.2, 144.7, 137.3, 134.1, 133.4, 130.6, 129.6, 129.1, 128.5, 127.9, 127.8, 126.5, 125.9, 125.4, 125.3, 125.1, 124.7, 123.6, 119.5, 115.5, 113.7, 60.5, 56.6, 23.3; **HRMS (ESI)**: Exact mass calcd for $C_{25}H_{23}O_2[M+H]^+$: 355.1698, Found: 355.1707.

(*R*)- 2,2'-Dimethoxy-3,3'-di(prop-1-en-2-yl)-1,1'-binaphthalene (1ba)

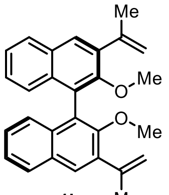

1ba

Eluent: PE, white solid, 165 mg, 42% yield, Mp: 122–123 °C;; **^{1}H NMR** (600 MHz, CDCl$_3$) δ 7.86 (d, *J* = 8.2 Hz, 2H), 7.83 (s, 2H), 7.37 (t, *J* = 7.3 Hz, 2H), 7.21 (t, *J* = 7.6 Hz, 2H), 7.12 (d, *J* = 8.5 Hz, 2H), 5.36 (s, 2H), 5.26 (s, 2H), 3.45 (s, 6H), 2.26 (s, 6H); **^{13}C NMR** (151 MHz, CDCl$_3$) δ 154.1, 144.6, 137.2, 133.5, 130.6, 128.7, 127.8, 126.0, 125.6, 125.3, 124.8, 115.6, 60.5, 23.2; **HRMS (ESI)**: Exact mass calcd for $C_{28}H_{27}O_2[M+H]^+$: 395.2011, Found: 395.2009.

3-(cyclohex-1-en-1-yl)-2,2'-Dimethoxy-1,1'-binaphthalene (1bz)

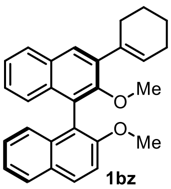

1bz

Eluent: PE/EA (10:1), 180 mg, 40% yield, Mp: 121–122 °C; **^{1}H NMR** (600 MHz, CDCl$_3$) δ 7.99 (d, *J* = 8.6 Hz, 1H), 7.86 (t, *J* = 9.1 Hz, 2H), 7.78 (s, 1H), 7.47 (d, *J* = 9.1 Hz, 1H), 7.36 (t, *J* = 7.4 Hz, 1H), 7.32 (t, *J* = 7.6 Hz, 1H), 7.24 (t, *J* = 7.6 Hz, 1H), 7.21 – 7.14 (m, 2H), 7.09 (d, *J* = 8.4 Hz, 1H), 6.07 – 6.03 (m, 1H), 3.80 (s, 3H), 3.42 (s, 3H), 2.54 (t, *J* = 18.8 Hz, 2H), 2.26 (dd, *J* = 6.3, 3.4 Hz, 2H), 1.81 – 1.79 (m, 2H), 1.77 – 1.71 (m, 2H).; **^{13}C NMR** (151 MHz, CDCl$_3$) δ 154.8, 154.4, 138.2, 137.6, 134.1, 133.0, 130.8, 129.4, 129.1, 128.5, 127.8, 127.7, 126.7, 126.5, 125.6, 125.4, 125.3, 124.9, 124.6, 123.5, 119.7, 113.7, 60.4, 56.5, 29.2, 25.9, 23.3, 22.2; **HRMS (ESI)**: Exact mass calcd for $C_{28}H_{27}O_2[M+H]^+$: 395.2011, Found: 395.2013.

The preparation of compounds 1bi and 1bj.

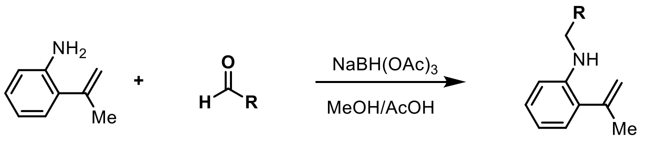

35   **36**, R= isobutyl; **37**, R= cyclohexylmethyl   **1bi** **1bj**

To a mixture of 2-(prop-1-en-2-yl)aniline (533 mg, 4.0 mmol) in MeOH (8 mL) was added aldehyde (433 mg, 1.2 equiv), $MgSO_4$ (240 mg, 0.5 equiv) and AcOH (0.5 ml). After stirring for 15 min, $NaBH(OAc)_3$ (1.7 g, 2.0 equiv) was added at 0 °C. The reaction mixture was then allowed to raise to room temperature and stirred for 6 h until compound **35** disappeared. The reaction was then filtered, and the filtrate was then transferred to a separatory funnel, diluted with water (30 mL), and extracted with ethyl acetate (EA, 30 mL×3). The combined organic layers were then washed with

brine (30 mL), dried with Na_2SO_4, filtered, and concentrated in vacuo. The crude mixture was purified by silica gel column chromatography to afford the desired product.

N-Isobutyl-2-(prop-1-en-2-yl)aniline (1bi)

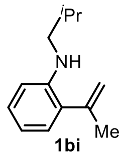

1bi

Eluent: PE/DCM (100:1), yellow oil, 0.42 g, 55% yield; **^{1}H NMR** (600 MHz, $CDCl_3$) δ 7.14 (t, *J* = 7.7 Hz, 1H), 7.01 (d, *J* = 7.1 Hz, 1H), 6.66 (t, *J* = 7.4 Hz, 1H), 6.62 (d, *J* = 8.1 Hz, 1H), 5.31 (s, 1H), 5.05 (s, 1H), 4.20 (s, 1H), 2.94 (d, *J* = 6.8 Hz, 2H), 2.07 (s, 3H), 1.91 (dq, *J* = 13.4, 6.7 Hz, 1H), 0.98 (d, *J* = 6.7 Hz, 6H) ; **^{13}C NMR** (151 MHz, $CDCl_3$) δ 144.5, 143.7, 129.1, 128.1, 127.8, 116.1, 115.4, 110.1, 51.7, 27.9, 24.1, 20.5; **HRMS (ESI)**: Exact mass calcd for $C_{13}H_{20}N[M+H]^+$: 190.1596, Found: 190.1599.

N-(Cyclohexylmethyl)-2-(prop-1-en-2-yl)aniline (1bj)

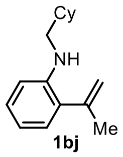

1bj

Eluent: PE/DCM (100:1), yellow oil, 1.8 g, 67% yield; **^{1}H NMR** (600 MHz, $CDCl_3$) δ 7.15 (t, *J* = 7.7 Hz, 1H), 7.01 (d, *J* = 7.3 Hz, 1H), 6.67 (t, *J* = 7.4 Hz, 1H), 6.62 (d, *J* = 8.2 Hz, 1H), 5.31 (s, 1H), 5.05 (s, 1H), 4.20 (s, 1H), 2.96 (d, *J* = 6.7 Hz, 2H), 2.07 (s, 3H), 1.83 – 1.73 (m, 4H), 1.61 (td, *J* = 7.9, 4.4 Hz, 2H), 1.32 – 1.21 (m, 3H), 0.99 (q, *J* = 12.3 Hz, 2H); **^{13}C NMR** (151 MHz, $CDCl_3$) δ 144.6, 143.8, 129.0, 128.1, 127.8, 116.0, 115.5, 110.1, 50.6, 37.4, 31.4, 26.6, 26.0, 24.1; **HRMS (ESI)**: Exact mass calcd for $C_{16}H_{24}N[M+H]^+$: 230.1909, Found: 230.1908.

The preparation of compounds 15.

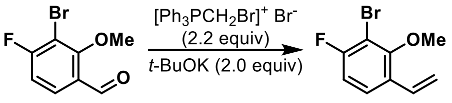

14 **15**, 94% yield

To a mixture of methyltriphenylphosphonium bromide (3.8 g, 8.8 mmol, 2.2 equiv.) suspended in THF (40.0 mL) was added portionwise *t*-BuOK (0.9 g, 8.0 mmol, 2.0 equiv.) under nitrogen atmospheres at 0 °C. The reaction was then stirred at 20 °C for 15 min and cooled to 0 °C again, aldehyde **14** (0.9 g, 4.0 mmol, 1.0 equiv) was slowly added and stirred overnight at 20 °C. After that the reaction was filtered through a short silica gel column, and the filtrate was then transferred to a separatory funnel, diluted with water (50 mL), and extracted with ethyl acetate (EA, 30 mL×3). The combined organic layers were then washed with brine (30 mL), dried with Na_2SO_4, filtered, and concentrated in vacuo. The crude mixture was purified by silica gel column chromatography (eluent: PE/EA = 10/1),) to give the desired product **15**. Yellow oil, 0.87 g, 94% yield; **^{1}H NMR** (600 MHz, $CDCl_3$) δ 7.43 (t, *J* = 7.4 Hz, 1H), 7.04 – 6.84 (m, 2H), 5.72 (d, *J* = 17.7 Hz, 1H), 5.33 (d, *J* = 11.1 Hz, 1H), 3.82 (s, 3H). **^{13}C NMR** (151 MHz, $CDCl_3$) δ 160.28, 158.63, 155.87, 130.38, 128.79,

128.76, 125.78, 125.72, 115.93, 115.92, 112.28, 112.13, 105.48, 105.35, 61.29. ^{19}F NMR (565 MHz, $CDCl_3$) δ -105.17. **HRMS (ESI)**: $[M+H]^{\oplus}$ Exact mass calcd for $C_9H_9BrFO^{\oplus}$ 230.9821，Found: 230.9816.

The preparation of compounds 1fe.

Scheme: 1

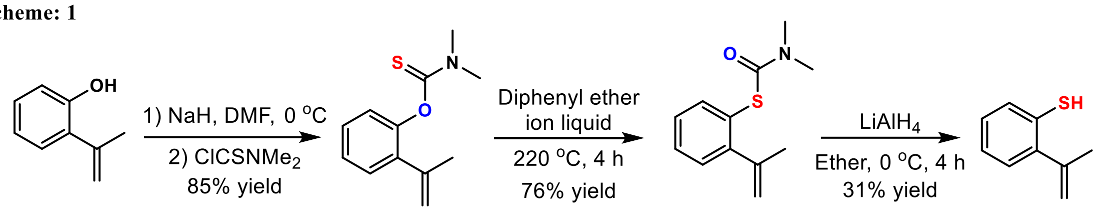

1bb **18** **19** **1fe**

O-(2-(prop-1-en-2-yl)phenyl) dimethylcarbamothioate (18)

To a mixture of **1bb** (268 mg, 2.0 mmol) in DMF (10 mL) was added NaH (120 mg, 60% in mineral oil, 3.0 mmol, 1.5 equiv) at 0 ℃ under N_2 atmosphere. After 30 min *N*,*N*-Dimethylcarbamothioic chloride was added and the reaction mixture was then stirred at room temperature for 18 h. The reaction was quenched by saturated NH_4Cl and then transferred to a separatory funnel, and extracted with ethyl acetate (EA, 30 mL×3). The combined organic layers were washed with brine, dried with Na_2SO_4, filtered, and concentrated in vacuo. The crude mixture was purified by silica gel column chromatography to afford the desired product 18 as pale yellow oil in 85% yield (374 mg).

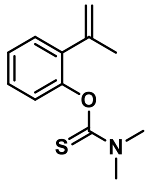

18

Eluent: PE/EA (10:1), **^{1}H NMR** (600 MHz, $CDCl_3$) δ 7.30 – 7.26 (m, 2H), 7.24 – 7.21 (m, 1H), 7.05 (d, *J* = 8.3 Hz, 1H), 5.17 – 5.16 (m, 1H), 5.06 – 5.05 (m, 1H), 3.44 (s, 3H), 3.31 (s, 3H), 2.07 (s, 3H).; **^{13}C NMR** (151 MHz, $CDCl_3$) δ 187.6, 150.6, 141.3, 136.7, 129.0, 127.6, 125.9, 124.0, 116.0, 43.2, 38.6, 23.7.; **HRMS (ESI)**: Exact mass calcd for $C_{12}H_{15}NOS[M+Na]^+$: 244.0767, Found: 244.0754.

S-(2-(prop-1-en-2-yl)phenyl) dimethylcarbamothioate (19)

To a 25-mL sealed Schlenk tube containing a magnetic stir bar was added **18** (354 mg, 1.6 mmol), 1-butyl-3-methylimidazolium tetrafluoroborate (ion liquid, 0.8 mL) and diphenyl ether (1 mL) under nitrogen atmosphere. The reaction mixture was then heated to 240 ℃ and stirred for 4hours. After that, the resulting solution was cooled to room temperature and mixture was directly purified by column chromatography to afford the desired product **19** as yellow oil in 76% yield (270 mg).

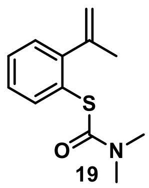

Eluent: PE/EA (5:1), **^{1}H NMR** (600 MHz, $CDCl_3$) δ 7.51 (dd, *J* = 7.8, 1.5 Hz, 1H), 7.33 (td, *J* = 7.5, 1.4 Hz, 1H), 7.26 (td, *J* = 7.6, 1.6 Hz, 1H), 7.22 (dd, *J* = 7.6, 1.7 Hz, 1H), 5.16 (p, *J* = 1.6 Hz, 1H), 4.88 (dd, *J* = 2.1, 1.0 Hz, 1H), 3.01 (d, *J* = 39.0 Hz, 6H), 2.09 – 2.03 (m, 3H).; **^{13}C NMR** (151 MHz, $CDCl_3$) δ 166.8, 148.7, 145.2, 137.4, 129.1, 128.5, 127.2, 126.0, 115.5, 36.7, 24.2; **HRMS (ESI)**: Exact mass calcd for $C_{12}H_{15}NOS[M+H]^+$: 222.0947, Found: 222.0956.

2-(prop-1-en-2-yl)benzenethiol (1fe)

To a one-necked 50-mL round bottomed flask equipped with a Teflon-coated magnetic stir bar was added **19** (50 mg 0.23 mmol, 1.0 equiv) and dry ether (10 mL) under nitrogen atomsphere in an ice bath. Lithium aluminum hydride ($LiAlH_4$, 17 mg, 0.46 mmol, 2 equiv) was then added in portion and the reaciton mixture was stirred for 30 min. The excess $LiAlH_4$ is quenched by the slow addition of water (10 mL). To the resulting mixture was added 1 N HCl (2 mL) and then transferred to a separatory funnel, and extracted with ether (10 mL×3). The combined organic layers were washed with brine, dried with Na_2SO_4, filtered, and concentrated in vacuo. The crude mixture was purified by silica gel column chromatography to afford the desired product **1fe** as colorless oil in 31% yield (11 mg).

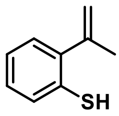

1fe

Eluent: PE/EA (20:1), **^{1}H NMR** (600 MHz, $CDCl_3$) δ 7.60 (dd, *J* = 7.8, 1.4 Hz, 1H), 7.21– 7.18 (m, 1H), 7.17– 7.15 (m, 1H), 7.12 – 7.11(m, 1H), 5.33 – 5.32 (m, 1H), 5.02 – 5.01 (m, 1H), 2.12 – 2.12 (m, 3H).; **^{13}C NMR** (600 MHz, CDCl3) δ 143.6, 142.9, 134.1, 128.1, 127.8, 126.7, 126.3, 117.4, 24.2. **HRMS (ESI)**: Exact mass calcd for $C_9H_{10}S[M]^+$: 150.0498, Found: 150.0503.

Experimental procedures and characterization of products

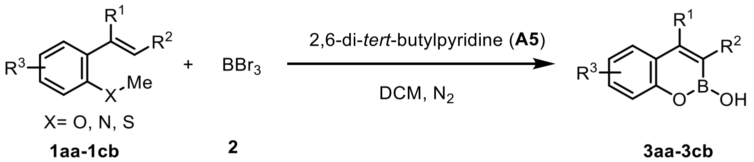

A dried Schlenk flask was charged with 2,6-di-*tert*-butylpyridine (**A5**, 0.22 mmol, 1.1 equiv) and 0.8 mL of DCM at –60 °C under nitrogen atmosphere. BBr_3 (**2**, 0.22 mmol, 1.1 equiv; 1.0 M solution in DCM) was subsequently added dropwise while stirring. After the reaction mixture was stirred for 5 min., a solution of **1aa** (0.20 mmol, 1.0 equiv) was added dropwise and the resulting

mixture was stirred for another 2.0 hours at –60 °C. Finally, the reaction was quenched with 2,6-di-*tert*-butylpyridine (1.0 equiv), methanol (1.0 mL), and water (0.2 mL). Upon the removal of solvents in vacuo, the residue was purified by flash silica gel (300-400 mesh) chromatography (petroleum ether/ethyl acetate = 5/1) to afford the desired product **3aa**.

4-Methyl-2H-benzo[e][1,2]oxaborinin-2-ol (3aa)

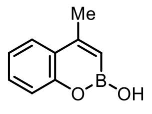

3aa

White solid, 30 mg, 95% yield; Mp: 136–138 °C; **^{1}H NMR** (600 MHz, MeOD) δ 7.63 (d, *J* = 7.9 Hz, 1H), 7.38 (t, *J* = 7.7 Hz, 1H), 7.25 (d, *J* = 8.2 Hz, 1H), 7.17 (t, *J* = 7.6 Hz, 1H), 5.98 (s, 1H), 2.41 (s, 3H); **^{13}C NMR** (151 MHz, MeOD) δ 156.9, 153.9, 130.3, 126.3, 123.3, 119.6, 22.2; **^{11}B NMR** (193 MHz, MeOD) δ 27.02; **HRMS (ESI)**: Exact mass calcd for $C_9H_8BO_2$ [M-H]$^-$: 159.0617, Found: 159.0626.

4-Ethyl-2H-benzo[e][1,2]oxaborinin-2-ol (3ab)

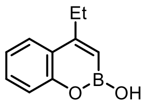

3ab

Eluent: PE/EA (10:1 to 5:1), white solid, 31 mg, 89% yield; Mp: 150–152 °C; **^{1}H NMR** (600 MHz, MeOD) δ 7.69 (d, *J* = 6.6 Hz, 1H), 7.41 – 7.35 (m, 1H), 7.27 (d, *J* = 7.1 Hz, 1H), 7.17 (t, *J* = 7.0 Hz, 1H), 6.00 (s, 1H), 2.79 (q, *J* = 7.4 Hz, 2H), 1.28 (t, *J* = 7.4 Hz, 3H); **^{13}C NMR** (151 MHz, MeOD) δ 162.3, 154.1, 130.2, 125.9, 125.5, 123.2, 119.9, 28.2, 13.3; **^{11}B NMR** (193 MHz, MeOD) δ 27.22; **HRMS (ESI)**: Exact mass calcd for $C_{10}H_{11}BKO_2$ [M+K]$^+$: 213.0489, Found: 213.0485.

(2-Hydroxy-2H-benzo[e][1,2]oxaborinin-4-yl)methyl methanesulfonate (3ac)

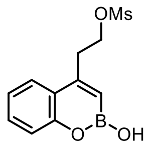

3ac

Eluent: PE/EA (10:1 to 5:1), white solid, 31 mg, 58% yield; Mp: 141–143 °C; **^{1}H NMR** (600 MHz, MeOD) δ 7.70 (d, *J* = 7.9 Hz, 1H), 7.42 (t, *J* = 7.8 Hz, 1H), 7.30 (d, *J* = 8.6 Hz, 1H), 7.21 (t, *J* = 7.6 Hz, 1H), 6.11 (s, 1H), 4.54 (t, *J* = 6.7 Hz, 2H), 3.23 (t, *J* = 6.7 Hz, 2H), 3.04 (s, 3H); **^{13}C NMR** (151 MHz, MeOD) δ 155.2, 154.2, 130.7, 125.8, 125.0, 123.5, 120.1, 69.7, 37.2, 35.0; **^{11}B NMR** (193 MHz, MeOD) δ 26.75; **HRMS (ESI)**: Exact mass calcd for $C_{10}H_{10}BO_5S$[M-H]$^-$: 267.0498, Found: 267.0499.

4-Phenyl-2H-benzo[e][1,2]oxaborinin-2-ol (3ad)

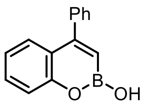

3ad

Eluent: PE/EA (10:1 to 5:1), white solid, 41 mg, 93% yield; Mp: 152–154 °C; **^{1}H NMR** (600 MHz, MeOD) δ 7.44 – 7.33 (m, 3H), 7.35 – 7.32 (m, 1H), 7.28 – 7.26 (m, 4H), 6.99 (t, *J* = 7.0 Hz, 1H), 5.97 (s, 1H); **^{13}C NMR** (151 MHz, MeOD) δ 161.4, 154.2, 142.1, 130.6, 129.4, 129.3, 129.0, 128.6, 125.2, 123.0,

119.9; ^{11}**B NMR** (193 MHz, MeOD) δ 26.88; **HRMS (ESI)**: Exact mass calcd for $C_{14}H_{10}BO_2$[M-H]$^-$: 221.0774, Found: 221.0775.

2H-Benzo[e][1,2]oxaborinin-2-ol (3ae)

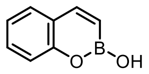

3ae

Following the general procedure, 0.6 mmol BBr_3 (3.0 equiv) was used to improve conversion. Eluent: PE/EA (5:1), white solid, 23 mg, 78% yield; Mp: 192–194 °C; ^{1}**H NMR** (600 MHz, MeOD) δ 7.79 (d, *J* = 10.0 Hz, 1H), 7.44 (d, *J* = 7.6 Hz, 1H), 7.38 (t, *J* = 7.9 Hz, 1H), 7.26 (d, *J* = 8.1 Hz, 1H), 7.15 (t, *J* = 7.5 Hz, 1H), 6.17 (d, *J* = 11.9 Hz, 1H); ^{13}**C NMR** (151 MHz,) δ 153.8, 149.9, 130.4, 129.8, 125.9, 123.5, 119.3; ^{11}**B NMR** (193 MHz, MeOD) δ 27.02; **HRMS (ESI)**: Exact mass calcd for $C_8H_6{}^{10}BO_2$ [M-H]$^-$: 144.0497, Found: 144.0492.

4,5-Dimethyl-2H-benzo[e][1,2]oxaborinin-2-ol (3af)

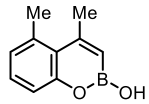

3af

Eluent: PE/EA (10:1 to 5:1), white solid, 31 mg, 89% yield; Mp: 140–142 °C; ^{1}**H NMR** (600 MHz, MeOD) δ 7.18 (t, *J* = 7.8 Hz, 1H), 7.09 (d, *J* = 8.1 Hz, 1H), 6.93 (d, *J* = 7.4 Hz, 1H), 5.90 (s, 1H), 2.68 (s, 3H), 2.60 (s, 3H); ^{13}**C NMR** (151 MHz, MeOD) δ 159.2, 155.4, 138.0, 129.5, 127.5, 125.5, 118.9, 29.4, 25.5; ^{11}**B NMR** (193 MHz, MeOD) δ 26.63; **HRMS (ESI)**: Exact mass calcd for $C_{10}H_{12}{}^{10}BO_2$[M+H]$^+$: 174.0967, Found: 174.0967.

5-Fluoro-4-methyl-2H-benzo[e][1,2]oxaborinin-2-ol (3ag)

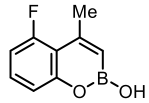

3ag

Eluent: PE/EA (10:1 to 5:1), white solid, 29 mg, 82% yield; Mp: 151–152 °C; ^{1}**H NMR** (600 MHz, MeOD) δ 7.36 – 7.31 (m, 1H), 7.09 (d, *J* = 7.1 Hz, 1H), 6.90 – 6.86 (m, 1H), 5.94 (s, 1H), 2.54 – 2.52 (m, 3H); ^{13}**C NMR** (151 MHz, MeOD) δ 162.7, 161.1, 155.5, 155.3, 130.3, 130.2, 116.1, 116.1, 115.6, 115.5, 110.7, 110.5, 26.6, 26.5.; ^{11}**B NMR** (193 MHz, MeOD) δ 26.74; ^{19}**F NMR** (565 MHz, MeOD) δ -112.51; **HRMS (ESI)**: Exact mass calcd for $C_9H_7BFO_2$[M-H]$^-$: 177.0523, Found: 177.0529.

5-Fluoro-4-methyl-2H-benzo[e][1,2]oxaborinin-2-ol (3ah)

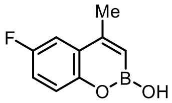

3ah

Eluent: PE/EA (10:1 to 5:1), white solid, 31 mg, 90% yield; Mp: 133–135 °C; ^{1}**H NMR** (600 MHz, MeOD) δ 7.23 (dd, *J* = 9.8, 2.9 Hz, 1H), 7.17 (dd, *J* = 8.9, 4.9 Hz, 1H), 7.08 (td, *J* = 8.5, 3.0 Hz, 1H), 5.96 (s, 1H), 2.30 (s, 3H); ^{13}**C NMR** (151 MHz, MeOD) δ 159.9, 158.3, 155.8, 155.8, 150.0, 149.9, 127.2, 127.1,

120.8, 120.7, 117.0, 116.9, 111.8, 111.7, 22.1; **^{11}B NMR** (193 MHz, MeOD) δ 26.79.^{19}F NMR (565 MHz, MeOD) δ -122.85; **HRMS (ESI)**: Exact mass calcd for $C_9H_9{}^{10}BFO_2$ [M+H]$^+$: 178.0716, Found: 178.0714.

6-Chloro-4-methyl-2H-benzo[e][1,2]oxaborinin-2-ol (3ai)

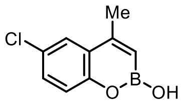

3ai

Eluent: PE/EA (10:1 to 5:1), white solid, 32 mg, 82% yield; Mp: 154–156 °C; **^{1}H NMR** (600 MHz, MeOD) δ 7.50 (d, *J* = 2.2 Hz, 1H), 7.30 (dd, *J* = 8.7, 2.3 Hz, 1H), 7.16 (d, *J* = 8.7 Hz, 1H), 5.97 (s, 1H), 2.32 (s, 3H). ; **^{13}C NMR** (151 MHz, MeOD) δ 155.6, 152.4, 130.0, 128.3, 127.6, 125.8, 121.1, 22.0; **^{11}B NMR** (193 MHz, MeOD) δ 26.84; **HRMS (ESI)**: Exact mass calcd for $C_9H_7BClO_2$[M-H]$^-$: 193.0228, Found: 1 93.0227.

4-Methyl-6-(trifluoromethyl)-2H-benzo[e][1,2]oxaborinin-2-ol (3aj)

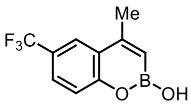

3aj

Eluent: PE/EA (10:1 to 5:1), white solid, 36 mg, 80% yield; Mp: 136–138 °C; **^{1}H NMR** (600 MHz, MeOD) δ 7.17 (dd, *J* = 9.8, 2.9 Hz, 1H), 7.12 (dd, *J* = 8.9, 4.9 Hz, 1H), 7.06 – 7.02 (m, 1H), 5.91 (s, 1H), 2.26 (s, 3H); **^{13}C NMR** (151 MHz, MeOD) δ 159.8, 158.2, 155.7, 155.7, 149.9, 127.1, 127.1, 120.7, 120.7, 117.0, 116.8, 111.7, 111.6, 22.1; **^{11}B NMR** (193 MHz, MeOD) δ 26.74; **^{19}F NMR** (565 MHz, MeOD) δ -122.75; **HRMS (ESI)**: Exact mass calcd for $C_{10}H_7{}^{10}BF_3O_2$[M-H]$^-$: 226.0528, Found: 226.0531.

6-(tert-butyl)-4-Methyl-2H-benzo[e][1,2]oxaborinin-2-ol (3ak)

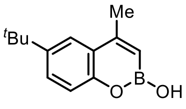

3ak

Eluent: PE/EA (10:1 to 5:1), white solid, 39 mg, 90% yield; Mp: 142–144 °C; **^{1}H NMR** (600 MHz, MeOD) δ 7.58 (d, *J* = 2.4 Hz, 1H), 7.43 (dd, *J* = 8.6, 2.4 Hz, 1H), 7.16 (d, *J* = 8.6, 1H), 5.94 (s, 1H), 2.40 (s, 3H), 1.34 (s, 9H); **^{13}C NMR** (151 MHz, MeOD) δ 157.1, 151.8, 146.0, 127.7, 125.5, 122.5, 119.2, 35.3, 32.0, 22.3; **^{11}B NMR** (193 MHz, MeOD) δ 27.16; **HRMS (ESI)**: Exact mass calcd for $C_{13}H_{16}BO_2$[M-H]$^-$: 215.1243, Found: 215.1241.

6-Methoxy-4-methyl-2H-benzo[e][1,2]oxaborinin-2-ol (3al)

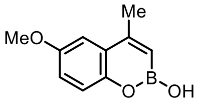

3al

Eluent: PE/EA (10:1 to 5:1), white solid, 30 mg, 79% yield; Mp: 131–133 °C; **^{1}H NMR** (600 MHz, MeOD) δ 7.17 (d, *J* = 8.9 Hz, 1H), 7.07 (d, *J* = 3.0 Hz, 1H), 6.98 (dd, *J* = 8.9 Hz, *J* = 3.0 Hz, 1H), 5.97 (s, 1H), 3.81 (s, 3H), 2.38 (s, 3H); **^{13}C NMR** (151 MHz, MeOD) δ 156.6, 156.1, 148.2, 126.6, 120.2, 116.9,

110.0, 56.2, 22.3; ^{11}**B NMR** (193 MHz, MeOD) δ 26.93; **HRMS (ESI)**: Exact mass calcd for $C_{10}H_{12}BO_3[M+H]^+$: 191.0879, Found: 191.0873.

6-methoxy-4-methyl-7-phenyl-2H-benzo[e][1,2]oxaborinin-2-ol (3am)

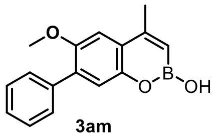

3am

Following the general procedure on 0.1 mmol scale. Eluent: PE/EA (5:1), white solid, 19 mg, 71% yield; Mp: 165–167 °C; ^{1}**H NMR** (600 MHz, MeOD) δ 7.53 (dd, *J* = 8.2, 1.3 Hz, 2H), 7.39 (t, *J* = 7.6 Hz, 2H), 7.32 (d, *J* = 7.4 Hz, 1H), 7.22 (s, 1H), 7.20 (s, 1H), 6.01 (d, *J* = 1.3 Hz, 1H), 3.82 (s, 3H), 2.46 (d, *J* = 1.3 Hz, 3H); ^{13}**C NMR** (151 MHz, MeOD) δ 153.2, 145.8, 138.8, 129.6, 127.5, 123.2, 122.9, 115.1, 79.7, 29.1, 23.1; ^{11}**B NMR** (193 MHz, MeOD) δ 27.13; **HRMS (ESI)**: Exact mass calcd for $C_{16}H_{15}BO_3$ $[M-H]^-$: 313.0035, Found: 313.0032.

4-((2-hydroxy-4-methyl-2H-benzo[e][1,2]oxaborinin-6-yl)oxy)Benzonitrile (3an)

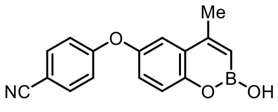

3an

Following the general procedure, 0.4 mmol BBr_3 (2.0 equiv) and 0.4 mmol **A5** (2.0 equiv) were used to improve conversion. Eluent: PE/EA (10:1 to 5:1), white solid, 39 mg, 70% yield; Mp: 124–125 °C; ^{1}**H NMR** (600 MHz, MeOD) δ 7.68 (d, *J* = 8.8 Hz, 2H), 7.38 (d, *J* = 2.9 Hz, 1H), 7.35 (d, *J* = 8.7 Hz, 1H), 7.16 (dd, *J* = 8.8 Hz, *J* = 2.8 Hz, 1H), 7.05 (d, *J* = 8.7 Hz, 2H), 6.07 (s, 1H), 2.36 (s, 3H); ^{13}**C NMR** (151 MHz, MeOD) δ 163.9, 156.1, 151.2, 150.5, 135.5, 127.7, 122.9, 121.3, 119.7, 118.6, 118.2, 106.6, 22.1; ^{11}**B NMR** (193 MHz, MeOD) δ 27.05; **HRMS (ESI)**: Exact mass calcd for $C_{16}H_{13}BNO_3[M+H]^+$: 278.0988, Found: 278.0990.

4-Methyl-2H-benzo[e][1,2]oxaborinine-2,6-diol (3ao)

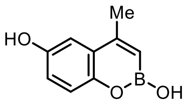

3ao

Following the general procedure, 0.6 mmol BBr_3 (3.0 equiv) was used to improve conversion. Eluent: PE/EA (5:1 to 4:1), white solid, 20 mg, 56% yield; Mp: 136–138 °C; ^{1}**H NMR** (600 MHz, MeOD) δ 7.10 (d, *J* = 8.7 Hz, 1H), 7.01 (d, *J* = 2.8 Hz, 1H), 6.85 (dd, *J* = 8.8 Hz, *J* = 2.8 Hz, 1H), 5.96 (s, 1H), 2.36 (s, 3H); ^{13}**C NMR** (151 MHz, MeOD) δ 156.6, 153.3, 147.4, 126.7, 120.1, 117.9, 111.4, 22.3; ^{11}**B NMR** (193 MHz, MeOD) δ 26.98; **HRMS (ESI)**: Exact mass calcd for $C_9H_8{}^{10}BO_3$ $[M-H]^-$: 174.0603, Found: 174.0604.

***N*-(2-hydroxy-4-methyl-2H-benzo[e][1,2]oxaborinin-6-yl)Pivalamide (3ap)**

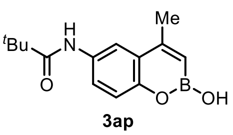

3ap

Following the general procedure, 0.6 mmol BBr_3 (3.0 equiv) was used to improve conversion. Eluent: PE/EA (20:1 to 10:1), white solid, 48 mg, 92% yield;

Mp: 147.2 – 148.6 °C; **^{1}H NMR** (600 MHz, MeOD) δ 9.16 (s, 1H), 7.83 (d, *J* = 5.9 Hz, 1H), 7.52 (s, 1H), 7.20 (d, *J* = 8.7 Hz, 1H), 5.98 (s, 1H), 2.39 (s, 3H), 1.31 (s, 9H); **^{13}C NMR** (151 MHz, MeOD) δ 179.9, 156.7, 150.7, 134.1, 126.2, 124.4, 119.5, 119.5, 40.4, 27.8, 22.3; **^{11}B NMR** (193 MHz, MeOD) δ 27.02; **HRMS (ESI)**: Exact mass calcd for $C_{14}H_{17}{}^{10}BNO_3$ [M-H]$^-$: 257.1338, Found: 257.1332.

4,7-Dimethyl-2H-benzo[e][1,2]oxaborinin-2-ol (3aq)

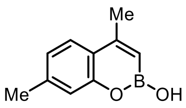

3aq

Eluent: PE/EA (10:1 to 5:1), white solid, 31 mg, 90% yield; Mp: 140–142 °C; **^{1}H NMR** (600 MHz, MeOD) δ 7.46 (d, *J* = 8.0 Hz, 1H), 7.05 (s, 1H), 6.96 (d, *J* = 7.0 Hz, 1H), 5.87 (s, 1H), 2.37 (s, 3H), 2.35 (d, *J* = 1.3 Hz, 3H); **^{13}C NMR** (151 MHz, MeOD) δ 156.8, 153.9, 141.0, 126.1, 124.2, 123.8, 119.9, 22.2, 21.3; **^{11}B NMR** (193 MHz, MeOD) δ 26.93; **HRMS (ESI)**: Exact mass calcd for $C_{10}H_{10}BO_2$[M-H]$^-$: 173.0774, Found: 173.0783.

Ethyl 2-(2-hydroxy-4-methyl-2H-benzo[e][1,2]oxaborinin-7-yl)acetate (3ar)

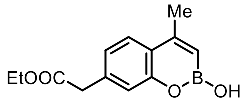

3ar

Eluent: PE/EA (5:1), white solid, 42 mg, 85% yield; Mp: 89–91 °C; **^{1}H NMR** (600 MHz, MeOD) δ 7.59 (d, *J* = 8.1 Hz, 1H), 7.20 (s, 1H), 7.10 (d, *J* = 8.0 Hz, 1H), 5.97 (s, 1H), 4.15 (q, *J* = 7.1 Hz, 2H), 3.69 (s, 2H), 2.40 (s, 3H), 1.25 (t, *J* = 7.1 Hz, 3H); **^{13}C NMR** (151 MHz, MeOD) δ 173.2, 156.7, 153.9, 137.2, 126.4, 125.2, 124.4, 120.4, 62.1, 41.7, 22.2, 14.5; **^{11}B NMR** (193 MHz, MeOD) δ 27.50; **HRMS (ESI)**: Exact mass calcd for $C_{13}H_{16}{}^{10}BO_4$[M+H]$^+$: 246.1178, Found: 246.1179.

***N*-((2-hydroxy-4-methyl-2H-benzo[e][1,2]oxaborinin-7-yl)methyl)Nonanamide (3as)**

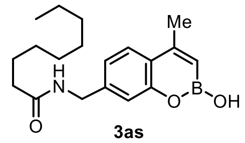

3as

Eluent: PE/EA (10:1 to 5:1), white solid, 54 mg, 82% yield; Mp: 92–94 °C; **^{1}H NMR** (600 MHz, MeOD) δ 8.49 (s, 1H), 7.59 (d, *J* = 8.1 Hz, 1H), 7.19 (s, 1H), 7.11 (d, *J* = 7.9 Hz, 1H), 5.95 (s, 1H), 4.42 – 4.40 (m, 2H), 2.39 (d, *J* = 1.1 Hz, 3H), 2.26 (t, *J* = 7.4 Hz, 2H), 1.66 – 1.62 (m, 2H), 1.32 – 1.27 (m, 10H), 0.88 (t, *J* = 7.1 Hz, 3H); **^{13}C NMR** (151 MHz, MeOD) δ 176.4, 156.7, 154.0, 142.0, 126.5, 125.3, 122.5, 118.5, 43.6, 37.1, 33.1, 33.0, 30.4, 30.3, 30.3, 27.1, 23.7, 22.2, 14.4; **^{11}B NMR** (193 MHz, MeOD) δ 27.08; **HRMS (ESI)**: Exact mass calcd for $C_{19}H_{29}BNO_3$ [M+H]$^+$: 330.2240, Found: 330.2239.

4-Methyl-7-(trifluoromethyl)-2H-benzo[e][1,2]oxaborinin-2-ol (3at)

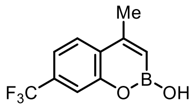

3at

Eluent: PE/EA (10:1 to 5:1), white solid, 41 mg, 89% yield; Mp: 123–125 °C; **^{1}H NMR** (600 MHz, MeOD) δ 7.70 (d, *J* = 8.2 Hz, 1H), 7.44 (s, 1H), 7.39 (d, *J* = 8.2 Hz, 1H), 6.06 (s, 1H), 2.37 (s, 3H); **^{13}C NMR** (151 MHz, MeOD) δ 155.6, 153.5, 132.1, 131.9, 131.7, 131.4, 129.2, 128.0, 127.3, 126.2, 124.4, 122.6, 119.6, 119.6, 119.6, 119.5, 116.7, 116.6, 116.6, 116.6, 22.1; **^{11}B NMR** (193 MHz, MeOD) δ 26.77; **^{19}F NMR** (565 MHz, MeOD) δ -63.92; **HRMS (ESI)**: Exact mass calcd for $C_{10}H_7BF_3O_2$[M-H]$^-$: 227.0491, Found: 227.0496.

7-Fluoro-4-methyl-2H-benzo[e][1,2]oxaborinin-2-ol (3au)

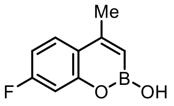

3au

Eluent: PE/EA (10:1 to 5:1), white solid, 30 mg, 85% yield; Mp: 148–150 °C; **^{1}H NMR** (600 MHz, MeOD) δ 7.66 (dd, *J* = 8.8, 6.5 Hz, 1H), 7.01 (dd, *J* = 10.0, 2.6 Hz, 1H), 6.95 (td, *J* = 8.6, 2.6 Hz, 1H), 5.95 (s, 1H), 2.40 (s, 3H); **^{13}C NMR** (151 MHz, MeOD) δ 159.9, 158.3, 155.8, 155.7, 150.0, 149.9, 127.2, 127.1, 120.8, 120.7, 117.0, 116.9, 111.8, 111.7, 22.1 **^{11}B NMR** (193 MHz, MeOD) δ 27.17; **^{19}F NMR** (565 MHz, MeOD) δ -138.28; **HRMS (ESI)**: Exact mass calcd for $C_9H_8BFO_2$[M+H]$^+$: 179.0680, Found: 179.0677;

8-Bromo-7-fluoro-4-methyl-2H-benzo[e][1,2]oxaborinin-2-ol (3av)

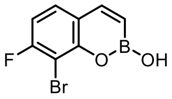

3av

Following the general procedure, 0.6 mmol BBr_3 (3.0 equiv) was used to improve conversion. Eluent: PE/EA (20:1 to 10:1), white solid, 43 mg, 88% yield; Mp: 178–180 °C; **^{1}H NMR** (600 MHz, MeOD) δ 7.76 (d, *J* = 11.4 Hz, 1H), 7.50 – 7.44 (m, 1H), 7.05 (t, *J* = 8.4 Hz, 1H), 6.17 (d, *J* = 12.0 Hz, 1H); **^{13}C NMR** (151 MHz, MeOD) δ 160.5, 158.9, 150.2, 147.5, 128.3, 128.3, 121.9, 110.0, 109.8, 99.7, 99.5; **^{11}B NMR** (193 MHz, MeOD) δ 27.06; **HRMS (ESI)**: Exact mass calcd for $C_8H_6BBrFO_2$[M+H]$^+$: 242.9628, Found: 242.9625.

8-Fluoro-4-methyl-2H-benzo[e][1,2]oxaborinin-2-ol (3aw)

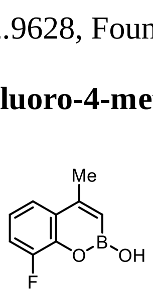

3aw

Eluent: PE/EA (10:1 to 5:1), white solid, 29 mg, 81% yield; Mp: 159–161 °C; **^{1}H NMR** (600 MHz, MeOD) δ 7.43 (d, *J* = 8.0 Hz, 1H), 7.25 – 7.19 (m, 1H), 7.16 – 7.09 (m, 1H), 6.05 (s, 1H), 2.41 (s, 3H); **^{13}C NMR** (151 MHz, MeOD) δ 156.5, 156.5, 154.3, 152.6, 142.1, 142.1, 128.5, 122.8, 122.7, 121.6, 121.6, 116.5, 116.4, 22.4; **^{11}B NMR** (193 MHz, MeOD) δ 26.75; **^{19}F NMR** (565 MHz, MeOD) δ -138.28; **HRMS (ESI)**: Exact mass calcd for $C_9H_9BFO_2$ [M+H]$^+$: 179.0680, Found: 179.0679.

6,8-Difluoro-4-methyl-2H-benzo[e][1,2]oxaborinin-2-ol (3ax)

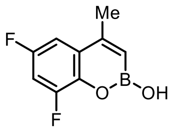

3ax

Eluent: PE/EA (10:1 to 5:1), white solid, 34 mg, 86% yield; Mp: 182–183 °C; **^{1}H NMR** (600 MHz, MeOD) δ 7.16 (d, *J* = 9.6 Hz, 1H), 7.12 – 7.06 (m, 1H), 6.10 (s, 1H), 2.36 (s, 3H); **^{13}C NMR** (151 MHz, MeOD) δ 158.7, 158.6, 157.1, 157.0, 155.6, 155.6, 155.6, 154.0, 153.9, 152.3, 152.2, 138.8, 138.7, 128.6, 128.5, 107.4, 107.4, 107.3, 107.2, 105.3, 105.1, 105.1, 104.9, 22.3; **^{11}B NMR** (193 MHz, MeOD) δ 26.60; **^{19}F NMR** (565 MHz, MeOD) δ -120.24 -133.52; **HRMS (ESI)**: Exact mass calcd for $C_9H_6{}^{10}BF_2O_2[M-H]^-$: 194.0465, Found: 194.0469.

4-Methyl-2H-naphtho[2,1-e][1,2]oxaborinin-2-ol (3ay)

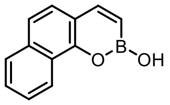

3ay

Following the general procedure, 0.6 mmol BBr_3 (3.0 equiv) was used to improve conversion. Eluent: PE/EA (10:1 to 5:1), white solid, 12 mg, 30% yield; Mp: 99–101 °C; **^{1}H NMR** (600 MHz, MeOD) δ 8.47 (d, *J* = 8.5 Hz, 1H), 7.90 (d, *J* = 11.8 Hz, 1H), 7.85 (d, *J* = 7.3 Hz, 1H), 7.63 – 7.55 (m, 2H), 7.55 – 7.51 (m, 1H), 7.47 (d, *J* = 8.5 Hz, 1H), 6.27 (d, *J* = 11.8 Hz, 1H); **^{13}C NMR** (151 MHz, MeOD) δ 150.4, 149.5, 135.6, 128.7, 128.1, 127.1, 127.0, 126.8, 123.1, 123.0, 120.5; **^{11}B NMR** (193 MHz, MeOD) δ 27.39; **HRMS (ESI)**: Exact mass calcd for $C_{12}H_{10}BO_2[M+H]^+$: 197.0774, Found: 197.0775.

10-(2-methoxynaphthalen-1-yl)-4-Methyl-2H-naphtho[2,3-e][1,2]oxaborinin-2-ol (3az)

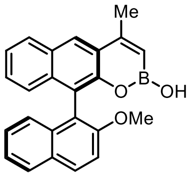

3az

Following the general procedure on 0.1 mmol scale. Eluent: PE/EA (10:1 to 5:1), white solid, 33 mg, 90% yield; Mp: 166–168 °C; **^{1}H NMR** (600 MHz, Acetone) δ 8.38 (s, 1H), 8.11 (d, *J* = 9.1 Hz, 1H), 8.09 (d, *J* = 8.3 Hz, 1H), 7.96 (d, *J* = 8.2 Hz, 1H), 7.63 (d, *J* = 9.1 Hz, 1H), 7.43 – 7.38 (m, 1H), 7.36 – 7.30 (m, 1H), 7.27 (t, *J* = 7.6 Hz, 1H), 7.19 (t, *J* = 7.6 Hz, 1H), 7.07 (d, *J* = 8.5 Hz, 1H), 6.98 (d, *J* = 8.5 Hz, 1H), 6.08 (s, 1H), 3.74 (s, 3H), 3.30 (s, 1H), 2.62 (s, 3H); **^{13}C NMR** (151 MHz, Acetone) δ 156.7, 156.6, 149.8, 135.4, 135.0, 130.8, 130.7, 130.6, 130.1, 129.4, 127.9, 127.6, 127.2, 126.4, 126.2, 125.4, 124.7, 121.8, 120.6, 115.6, 57.3, 23.0; **^{11}B NMR** (193 MHz, Acetone) δ 26.96; **HRMS (ESI)**: Exact mass calcd for $C_{24}H_{18}BO_3[M-H]^-$: 365.1349, Found: 365.1360.

10-(2-methoxynaphthalen-1-yl)-4-Methyl-2H-naphtho[2,3-e][1,2]oxaborinin-2-ol (3az')

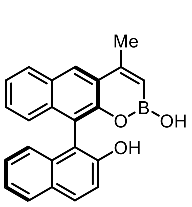

3az'

Following the general procedure on 0.1 mmol scale, 0.3 mmol BBr_3 (3.0 equiv) was used to improve conversion. Eluent: PE/EA (4:1), white solid, 27 mg, 75% yield; Mp: 169–171 °C; **^{1}H NMR** (600 MHz, Acetone-d_6) δ 8.39 (s, 1H), 8.09 (d, *J* = 8.2 Hz, 1H), 7.96 (d, *J* = 8.8 Hz, 1H), 7.91 (d, *J* = 8.1 Hz, 1H), 7.42 (t, *J* = 7.5 Hz, 1H), 7.38 (d, *J* = 8.9 Hz, 1H), 7.33 – 7.25 (m, 2H),

7.18 – 7.15 (m, 2H), 6.93 (d, *J* = 8.0 Hz, 1H), 6.10 (s, 1H), 2.62 (s, 3H); **^{13}C NMR** (151 MHz, Acetone-d_6) δ 156.0, 153.8, 150.2, 135.4, 134.7, 130.4, 130.2, 129.8, 129.7, 128.9, 127.6, 127.0, 127.0, 126.4, 126.0, 125.5, 125.0, 123.6, 120.3, 119.4, 116.4, 22.6; **^{11}B NMR** (193 MHz, Acetone-d_6) δ 27.30; **HRMS (ESI)**: Exact mass calcd for $C_{23}H_{18}BO_3[M+H]^+$: 353.1349, Found: 353.1365.

4,4'-Dimethyl-2H,2'H-[10,10'-binaphtho[2,3-e][1,2]oxaborinine]-2,2'-diol (1ba)

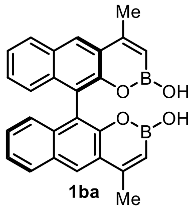

1ba

Following the general procedure, 0.4 mmol BBr_3 (2.0 equiv) and 0.4 mmol **A5** (2.0 equiv) were used to improve conversion. Eluent: PE/EA (5:1), light yellow solid, 75 mg, 90% yield; Mp: 177–179 °C; **^{1}H NMR** (600 MHz, MeOD) δ 8.38 (s, 2H), 8.08 (d, *J* = 8.3 Hz, 2H), 7.43 (t, *J* = 7.6 Hz, 2H), 7.31 (t, *J* = 7.7 Hz, 2H), 7.20 (d, *J* = 8.5 Hz, 2H), 6.02 (s, 2H), 2.64 (s, 6H); **^{13}C NMR** (151 MHz, MeOD) δ 155.4, 147.7, 133.3, 129.4, 128.6, 126.5, 125.5, 125.0, 125.0, 124.0, 120.1, 21.2; **^{11}B NMR** (193 MHz, MeOD) δ 26.63; **HRMS (ESI)**: Exact mass calcd for $C_{26}H_{21}B_2O_4[M+H]^+$: 419.1626, Found: 419.1633.

(3a*S*,3b*R*,11b*S*,13a*S*)-8-Hydroxy-10,13a-dimethyl-3,3a,3b,4,5,8,11b,12,13,13a-decahydrocyclo penta[7,8]phenanthro[3,2-e][1,2]oxaborinin-1(2H)-one (3bb)

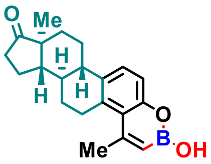

3bb

Following the general procedure on 0.1 mmol scale, 2.0 mmol BBr_3 (10.0 equiv) and 2.0 mmol **A5** (10.0 equiv) were used, .and the reaction mixture was stirred at 0 °C for 8.0 h. Eluent: PE/EA (5:1), light yellow solid, 17 mg, 50% yield; Mp: 231–233 °C; **^{1}H NMR** (600 MHz, MeOD) δ 7.34 (d, *J* = 8.6 Hz, 1H), 7.07 (d, *J* = 8.6 Hz, 1H), 5.95 (s, 1H), 3.21 – 3.14 (m, 1H), 2.64 (s, 3H), 2.49 (m, 1H), 2.41 – 2.33 (m, 1H), 2.28 (dd, *J* = 15.8, 7.4 Hz, 1H), 2.18 – 2.09 (m, 1H), 2.09 – 1.97 (m, 2H), 1.92 – 1.86 (m, 1H), 1.73 – 1.62 (m, 1H), 1.62 – 1.44 (m, 4H), 1.34 – 1.25 (m, 2H), 0.93 (s, 3H). **^{13}C NMR** (151 MHz, MeOD) δ 198.2, 157.9, 152.1, 135.9, 134.7, 126.7, 124.1, 116.9, 50.3, 45.3, 37.4, 35.4, 31.6, 30.6, 29.2, 26.6, 26.3, 21.0, 13.0. ^{11}B NMR (193 MHz, Methanol-d_4) δ 26.60. **HRMS (ESI)**: Exact mass calcd for $C_{21}H_{26}BO_3[M+H]^+$: 337.1975, Found: 337.1972.

1,4-Dimethylbenzo[e][1,2]azaborinin-2(1H)-ol (3bh)

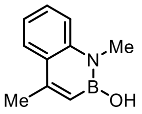

3bh

Following the general procedure, the reaction was performed at 0 °C. Eluent: PE/EA (10:1 to 5:1), white solid, 22 mg, 63% yield; Mp: 92–95 °C; **^{1}H NMR** (600 MHz, MeOD) δ 7.73 (dd, J = 7.9, 1.6 Hz, 1H), 7.44 – 7.42 (m, 1H), 7.35 (dd, J = 8.5, 1.2 Hz, 1H), 7.06 (t, J = 1.1 Hz, 1H), 6.35 (d, J = 1.2 Hz, 1H), 3.31 (p, J = 1.6 Hz, 3H), 2.52 (d, J = 1.2 Hz, 3H); **^{13}C NMR** (151 MHz, MeOD) δ 150.4, 143.2, 128.3,

125.6, 124.0, 118.5, 113.8, 29.6, 22.8; **^{11}B NMR** (193 MHz, DMSO) δ 27.95; **HRMS (ESI)**: Exact mass calcd for $C_{10}H_{13}BNO[M+H]^+$: 174.1090, Found: 174.1081.

1-Isobutyl-4-methylbenzo[e][1,2]azaborinin-2(1H)-ol (3bi)

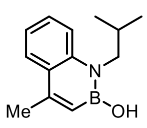

3bi

Following the general procedure, the reaction was performed at 0 °C. Eluent: PE/EA (10:1 to 5:1), white solid, 24 mg, 65% yield; Mp: 95–97 °C; **^{1}H NMR** (600 MHz, MeOD) δ 7.66 (d, *J* = 7.8 Hz, 1H), 7.34 (t, *J* = 7.6 Hz, 1H), 7.28 (d, *J* = 8.5 Hz, 1H), 6.99 (t, *J* = 7.4 Hz, 1H), 6.15 (s, 1H), 3.74 (d, *J* = 7.1 Hz, 2H), 2.44 (s, 3H), 2.13 – 2.07 (m, 1H), 0.91 (d, *J* = 6.5 Hz, 6H); **^{13}C NMR** (151 MHz, MeOD) δ 152.5, 143.8, 128.9, 127.0, 126.3, 119.5, 115.7, 50.2, 27.9, 23.32, 23.30, 20.51, 20.50; **^{11}B NMR** (193 MHz, MeOD) δ 26.66; **HRMS (ESI)**: Exact mass calcd for $C_{13}H_{19}BNO[M+H]^+$: 216.1560, Found: 216.1561.

1-Cyclohexylmethyl -4-methylbenzo[e][1,2]azaborinin-2(1H)-ol (3bj)

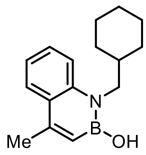

3bj

Following the general procedure, the reaction was performed at 0 °C. Eluent: PE/EA (10:1 to 5:1), white solid, 32 mg, 62% yield; Mp: 136–138 °C; **^{1}H NMR** (600 MHz, $CDCl_3$) δ 7.79 (d, *J* = 8.0 Hz, 1H), 7.54 – 7.43 (m, 1H), 7.41 (d, *J* = 8.4 Hz, 1H), 7.13 (t, *J* = 7.6 Hz, 1H), 6.30 (s, 1H), 3.94 (d, *J* = 6.7 Hz, 2H), 2.53 (s, 3H), 1.89 – 1.85 (m, 1H), 1.75 – 1.54 (m, 5H), 1.20 – 1.08 (m, 5H); **^{13}C NMR** (151 MHz, $CDCl_3$) δ 152.1, 142.5, 128.0, 126.3, 125.5, 118.9, 115.2, 48.9, 36.5, 31.4, 26.7, 26.1, 23.2; **^{11}B NMR** (193 MHz, MeOD) δ 26.65; **HRMS (ESI)**: Exact mass calcd for $C_{16}H_{23}BNO[M+H]^+$: 256.1873, Found: 256.1872.

3-Methyl-7,8-dihydro-1H-[1,2]azaborinino[5,6,1-hi]indol-1-ol (3bk)

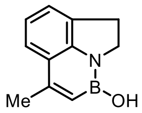

3bk

Following the general procedure, the reaction was performed at 0 °C. Eluent: PE/EA (20:1 to 10:1), white solid, 30 mg, 82% yield; Mp: 221–222 °C; **^{1}H NMR** (600 MHz, DMSO) δ 7.77 (s, 1H), 7.34 (d, *J* = 7.9 Hz, 1H), 7.19 (d, *J* = 7.0 Hz, 1H), 6.89 (t, *J* = 7.6 Hz, 1H), 6.02 (s, 1H), 4.06 (t, *J* = 8.0 Hz, 2H), 3.26 (t, *J* = 8.4 Hz, 2H), 2.36 (s, 3H); **^{13}C NMR** (151 MHz, DMSO) δ 149.7, 146.2, 131.3, 122.5, 121.6, 120.3, 118.9, 46.0, 28.3, 21.1; **^{11}B NMR** (193 MHz, DMSO) δ 27.48; **HRMS (ESI)**: Exact mass calcd for $C_{11}H_{13}{}^{10}BNO[M+H]^+$: 185.1127, Found: 185.1126.

3-Methyl-1H-[1,2]azaborinino[5,6,1-hi]indol-1-ol (3bl)

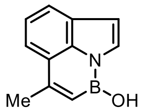

3bl

Following the general procedure, the reaction was performed at 0 °C. Eluent: PE/EA (20:1 to 10:1), white solid, 33 mg, 91 % yield; Mp: 140–142 °C; **^{1}H**

NMR (600 MHz, MeOD) δ 7.73 (d, *J* = 7.3 Hz, 1H), 7.65 (d, *J* = 7.4 Hz, 1H), 7.59 (s, 1H), 7.30 (t, *J* = 6.9 Hz, 1H), 6.75 (s, 1H), 6.34 (s, 1H), 2.60 (s, 3H). **^{13}C NMR** (151 MHz, MeOD) δ 156.3, 137.0, 130.9, 128.0, 123.7, 123.3, 122.4, 121.8, 109.8, 21.5. **^{11}B NMR** (193 MHz, MeOD) δ 28.36; **HRMS (ESI)**: Exact mass calcd for $C_{11}H_9BNO[M-H]^-$: 182.0777, Found: 182.0783.

3-Methyl-1H-[1,2]azaborinino[5,6,1-jk]carbazol-1-ol (3bm)

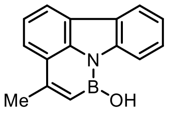

3bm

Following the general procedure, the reaction was performed at 0 °C. Eluent: PE/EA (20:1 to 10:1), white solid, 34 mg, 72% yield; Mp: 194–196 °C; **^{1}H NMR** (600 MHz, MeOD) δ 8.19 (d, *J* = 8.1 Hz, 1H), 8.11 (d, *J* = 7.5 Hz, 1H), 8.06 (d, *J* = 7.7 Hz, 1H), 7.79 (d, *J* = 7.7 Hz, 1H), 7.44 – 7.40 (m, 1H), 7.32 (t, *J* = 7.4 Hz, 1H), 6.46 (s, 1H), 2.6 (s, 3H); **^{13}C NMR** (151 MHz, MeOD) δ 155.5, 143.9, 139.9, 128.4, 127.5, 126.6, 123.9, 123.6, 123.6, 122.3, 121.8, 121.3, 117.5, 21.7; **^{11}B NMR** (193 MHz, MeOD) δ 29.12; **HRMS (ESI)**: Exact mass calcd for $C_{15}H_{13}BNO[M+H]^+$: 234.1090, Found: 234.1084.

(*Z*)-(2-(2-(dimethylamino)phenyl)prop-1-en-1-yl)Boronic acid (3bn)

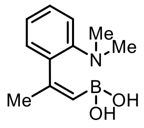

3bn

Following the general procedure, the reaction was performed at 0 °C. Eluent: PE/EA (1:1 to 1:2), white solid, 21 mg, 52% yield; Mp: 93–94 °C; **^{1}H NMR** (600 MHz, MeOD) δ 7.54 (d, *J* = 8.0 Hz, 1H), 7.46 (d, *J* = 7.8 Hz, 1H), 7.33 (t, *J* = 7.4 Hz, 1H), 7.30 (t, *J* = 7.6 Hz, 1H), 6.07 (s, 1H), 2.82 (s, 6H), 2.20 (s, 3H); **^{13}C NMR** (151 MHz, MeOD) δ 147.2, 139.2, 135.1, 128.9, 128.8, 127.2, 120.3, 46.1, 23.0; **^{11}B NMR** (193 MHz, MeOD) δ 28.30; **HRMS (ESI)**: Exact mass calcd for $C_{11}H_{15}BNO_2[M-H]^-$: 204.1196, Found: 204.1202.

(*Z*)-(2-(2-(dimethyl-l3-sulfaneyl)phenyl)prop-1-en-1-yl)Boronic acid (3bo)

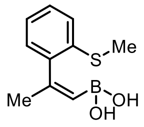

3bo

Following the general procedure, the reaction was performed at 0 °C. Eluent: PE/EA (10:1 to 5:1), white solid, 17 mg, 42% yield; Mp: 126–128 °C; **^{1}H NMR** (600 MHz, MeOD) δ 7.26 – 7.20 (m, 2H), 7.08 (t, *J* = 7.5 Hz, 1H), 7.01 (d, *J* = 7.4 Hz, 1H), 5.65 (d, *J* = 1.5 Hz, 1H), 2.41 (s, 3H), 2.16 (s, 3H); **^{13}C NMR** (151 MHz, MeOD) δ 155.1, 144.8, 136.8, 129.1, 128.6, 126.8, 125.7, 27.8, 16.0; **^{11}B NMR** (193 MHz, MeOD) δ 26.76; **HRMS (ESI)**: Exact mass calcd for $C_{10}H_{12}BSO_2[M-H]^-$: 207.0651, Found: 207.0650.

3-Methyl-2H-benzo[e][1,2]oxaborinin-2-ol (3bp)

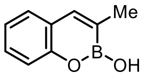

3bp

Following the general procedure, the reaction was performed at 0 °C. Eluent: PE/EA (5:1), white solid, 27 mg, 85% yield; Mp: 165–166 °C; **^{1}H NMR** (600

MHz, MeOD) δ 7.39 (s, 1H), 7.33 (d, J = 7.6 Hz, 1H), 7.28 (t, J = 7.7 Hz, 1H), 7.21 (d, J = 8.0 Hz, 1H), 7.09 (t, J = 7.5 Hz, 1H), 1.98 (s, 3H).; **^{13}C NMR** (151 MHz, MeOD) δ 153.0, 144.3, 129.0, 128.9, 126.2, 123.4, 118.8, 18.5; **^{11}B NMR** (193 MHz, MeOD) δ 26.69; **HRMS (ESI)**: Exact mass calcd for $C_9H_9BO_2Na[M+Na]^+$: 183.0593, Found: 183.0583.

3-Hexyl-2H-benzo[e][1,2]oxaborinin-2-ol (3bq)

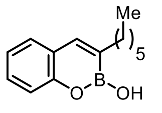

3bq

Following the general procedure, the reaction was performed at –20 °C. Eluent: PE/EA (10:1 to 5:1), white solid, 38 mg, 82% yield; Mp: 124–126 °C; **^{1}H NMR** (600 MHz, MeOD) δ 7.39 (s, 1H), 7.37 (d, J = 7.9 Hz, 1H), 7.30 (t, J = 7.7 Hz, 1H), 7.23 (d, J = 8.1 Hz, 1H), 7.11 (t, J = 7.4 Hz, 1H), 2.35 (t, J = 7.4 Hz, 2H), 1.53 (q, J = 7.4 Hz, 2H), 1.38 – 1.26 (m,6H), 0.90 (t, J = 6.6 Hz, 3H); **^{13}C NMR** (151 MHz, MeOD) δ 153.0, 143.5, 129.1 (d, J = 4.9 Hz), 126.2, 123.4, 118.8, 34.1, 32.9, 30.8, 30.3, 23.7, 14.4; **^{11}B NMR** (193 MHz, MeOD) δ 27.59; **HRMS (ESI)**: Exact mass calcd for $C_{14}H_{18}BO_2[M-H]^-$: 229.1400, Found: 229.1401.

3-Phenyl-2H-benzo[e][1,2]oxaborinin-2-ol (3br)

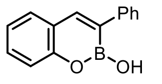

3br

Following the general procedure, the reaction was performed at 0 °C. Eluent: PE/EA (10:1 to 5:1), white solid, 36 mg, 82% yield; Mp: 135–137 °C; **^{1}H NMR** (600 MHz, MeOD) δ 7.86 (s, 1H), 7.66 (d, J = 7.3 Hz, 2H), 7.54 (d, J = 7.5 Hz, 1H), 7.39 (t, J = 7.1 Hz, 1H), 7.35 (t, J = 7.6 Hz, 2H), 7.32 (d, J = 8.2 Hz, 1H), 7.25 (t, J = 7.3 Hz, 1H), 7.18 (t, J = 7.4 Hz, 1H); **^{13}C NMR** (151 MHz, MeOD) δ 161.4, 154.2, 142.1, 130.6, 129.4, 129.3, 129.0, 128.6, 125.2, 123.0, 119.9; **^{11}B NMR** (193 MHz, MeOD) δ 26.74; **HRMS (ESI)**: Exact mass calcd for $C_{14}H_{12}BO_2[M+H]^+$: 223.0930, Found: 223.0931.

Benzo[e]indeno[1,2-c][1,2]oxaborinin-6(11H)-ol (3bs)

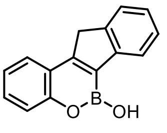

3bs

Following the general procedure, the reaction was performed at –20 °C· Eluent: PE/EA (10:1 to 5:1), white solid, 43 mg, 92% yield; Mp: 170–172 °C; **^{1}H NMR** (600 MHz, $CDCl_3$) δ 7.81 (d, J = 7.3 Hz, 1H), 7.66 (d, J = 7.4 Hz, 1H), 7.57 (d, J = 7.1 Hz, 1H), 7.38 (dt, J = 13.8, 7.5 Hz, 2H), 7.32 (d, J = 8.0 Hz, 1H), 7.22 (t, J = 7.3 Hz, 2H), 4.69 (s, 1H), 3.99 (s, 2H); **^{13}C NMR** (151 MHz, $CDCl_3$) δ 160.1, 152.0, 145.4, 142.8, 128.9, 127.0, 125.4, 124.9, 123.9, 123.1, 122.9, 122.6, 118.7, 38.4; **^{11}B NMR** (193 MHz, $CDCl_3$) δ 26.89; **HRMS (ESI)**: Exact mass calcd for $C_{15}H_{10}BO_2[M-H]^-$: 233.0888, Found: 233.0885.

2,3-Dihydrobenzo[e]cyclopenta[c][1,2]oxaborinin-4(1H)-ol (3bt)

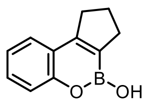

3bt

Following the general procedure, the reaction was performed at –20 °C. Eluent: PE/EA (10:1 to 5:1), white solid, 32 mg, 86% yield; Mp: 87–89 °C; **^{1}H NMR** (600 MHz, MeOD) δ 7.31 (d, *J* = 7.6 Hz, 1H), 7.27 (d, *J* = 7.6 Hz, 1H), 7.20 (d, *J* = 8.1 Hz, 1H), 7.09 (s, 1H), 2.79 (t, *J* = 7.4 Hz, 2H), 2.64 (t, *J* = 7.0 Hz, 2H), 2.00 (t, *J* = 7.4 Hz, 2H); **^{13}C NMR** (151 MHz, MeOD) δ 159.6, 153.8, 129.2, 126.2, 124.4, 123.2, 119.0, 34.3, 33.6, 25.0; **^{11}B NMR** (193 MHz, MeOD) δ 26.20; **HRMS (ESI)**: Exact mass calcd for $C_{11}H_{12}BO_2[M+H]^+$: 187.0930, Found: 187.0931.

1,4-Dihydro-2H,5H-benzo[e]pyrano[3,4-c][1,2]oxaborinin-5-ol (3bu)

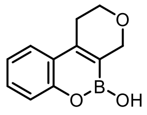

3bu

Following the general procedure, the reaction was performed at –20 °C. Eluent: PE/EA (10:1 to 5:1), white solid, 28 mg, 70% yield; Mp: 85–87 °C; **^{1}H NMR** (600 MHz, MeOD) δ 7.56 (d, *J* = 1.6 Hz, 1H), 7.37 (t, *J* = 7.4 Hz, 1H), 7.28 (d, *J* = 8.0 Hz, 1H), 7.19 (t, *J* = 7.6 Hz, 1H), 4.47 (s, 2H), 3.99 (t, *J* = 5.6 Hz, 2H), 2.77 – 2.72 (m, 2H); **^{13}C NMR** (151 MHz, MeOD) δ 152.7, 146.7, 129.7, 125.5, 124.6, 123.5, 119.5, 67.0, 65.1, 26.3; **^{11}B NMR** (193 MHz, MeOD) δ 26.67; **HRMS (ESI)**: Exact mass calcd for $C_{11}H_{11}BNaO_3[M+Na]^+$: 225.0699, Found: 225.0694.

3-Methyl-4-(p-tolyl)-2H-benzo[e][1,2]oxaborinin-2-ol (3bv)

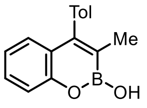

3bv

Following the general procedure on 0.1 mmol scale. Eluent: PE/EA (10:1 to 5:1), white solid, 47 mg, 95% yield; Mp: 155–156 °C; **^{1}H NMR** (600 MHz, MeOD) δ 7.31 (d, *J* = 7.7 Hz, 2H), 7.28 (d, *J* = 3.7 Hz, 2H), 7.02 (d, *J* = 7.9 Hz, 2H), 6.99 – 6.93 (m, 1H), 6.89 (d, *J* = 7.9 Hz, 1H), 2.43 (s, 3H), 1.71 (s, 3H); **^{13}C NMR** (151 MHz, MeOD) δ 155.0, 152.7, 138.2, 136.8, 130.2, 129.9, 129.0, 128.5, 127.1, 123.0, 119.2, 21.3, 15.4; **^{11}B NMR** (193 MHz, MeOD) δ 26.83; **HRMS (ESI)**: Exact mass calcd for $C_{16}H_{16}BO_2[M+H]^+$: 251.1243, Found: 251.1239.

3-Bromo-4-(p-tolyl)-2H-benzo[e][1,2]oxaborinin-2-ol (3bw)

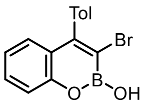

3bw

Following the general procedure on 0.1 mmol scale. Eluent: PE/EA (5:1), white solid, 25 mg, 81% yield; Mp: 169–170 °C; **^{1}H NMR** (600 MHz, MeOD) δ 7.44 – 7.38 (m, 1H), 7.37 – 7.31 (m, 3H), 7.07 (d, *J* = 8.0 Hz, 2H), 7.03 (t, *J* = 7.8 Hz, 1H), 6.97 (d, *J* = 8.1 Hz, 1H), 2.44 (s, 3H); **^{13}C NMR** (151 MHz, MeOD) δ 158.4, 152.7, 139.1, 136.9, 130.6, 130.2, 129.6, 129.2, 126.3, 123.7, 119.6, 21.4; **^{11}B NMR** (193 MHz, MeOD) δ 24.64; **HRMS (ESI)**: Exact mass calcd for $C_{15}H_{11}BBrO_2[M-H]^-$: 313.0035, Found: 313.0032.

3,4-Dibutyl-2H-benzo[e][1,2]oxaborinin-2-ol (3bx)

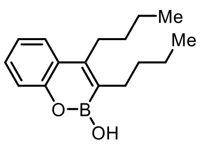

3bx

Eluent: PE/EA (5:1), white solid, 47 mg, 93% yield; Mp: 160–162 °C; **^{1}H NMR** (600 MHz, MeOD) δ 7.61 (d, *J* = 8.0 Hz, 1H), 7.30 (t, *J* = 7.6 Hz, 1H), 7.24 (d, *J* = 8.0 Hz, 1H), 7.15 (t, *J* = 7.6 Hz, 1H), 2.78 (t, *J* = 7.4 Hz, 2H), 2.41 (t, *J* = 7.4 Hz, 2H), 1.52 (dd, *J* = 6.8, 3.5 Hz, 4H), 1.41 (dt, *J* = 7.4, 3.8 Hz, 4H), 0.99 (t, *J* = 6.9 Hz, 3H), 0.96 (t, *J* = 6.8 Hz, 3H); **^{13}C NMR** (151 MHz, MeOD) δ 153.1, 152.7, 128.8, 126.1, 126.0, 123.2, 119.6, 34.0, 33.3, 29.3, 28.6, 24.2, 24.2, 14.4, 14.3; **^{11}B NMR** (193 MHz, MeOD) δ 26.92; **HRMS (ESI)**: Exact mass calcd for $C_{16}H_{24}BO_2[M+H]^+$: 259.1869, Found: 259.1869.

3,4-Dimethyl-2H-benzo[e][1,2]oxaborinin-2-ol (3by)

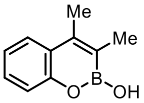

3by

Eluent: PE/EA (10:1 to 5:1), white solid, 29 mg, 93% yield; Mp: 166–168 °C; **^{1}H NMR** (600 MHz, MeOD) δ 7.59 (d, *J* = 7.9 Hz, 1H), 7.29 (t, *J* = 7.6 Hz, 1H), 7.20 (d, *J* = 8.1 Hz, 1H), 7.13 (t, *J* = 7.6 Hz, 1H), 2.24 (s, 3H), 1.94 (s, 3H); **^{13}C NMR** (151 MHz, MeOD) δ 152.6, 148.7, 128.8, 127.0, 125.8, 123.2, 119.3, 14.4, 14.0; **^{11}B NMR** (193 MHz, MeOD) δ 26.55; **HRMS (ESI)**: Exact mass calcd for $C_{10}H_{12}BO_2[M+H]^+$: 175.0930, Found: 175.0926.

10 mmol-Scale experiment

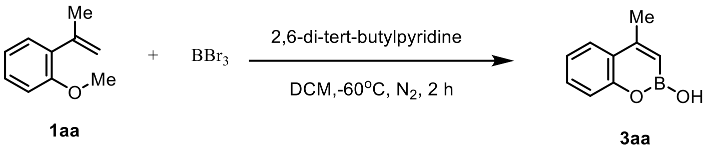

4-Methyl-2H-benzo[e][1,2]oxaborinin-2-ol (3aa)

A dried Schlenk flask was charged with 2,6-di-*tert*-butylpyridine (11 mmol, 1.1 equiv) and 1.0 mL of DCM at –60 °C, backfilled with nitrogen. Then, BBr_3 (22 mmol, 1.1 equiv; present as 1.0 mol/L solution in DCM) was added dropwise into the solution while stirring and the reaction was continued for 5 min. After that, **1aa** (10 mmol, 1.0 equiv) was added dropwise and the solution was stirred for another 2.0 h under –60 °C. Finally, the resulting reaction mixture was quenched with 2,6-di-tert-butylpyridine (10.0 equiv) and methanol (5.0 mL). The solvents were then removed in vacuo on a rotary evaporator and the residue was directly purified by flash silica gel chromatography to afford the desired products **3aa**. Eluent: PE/EA (10:1 to 5:1), white solid, 1.52 g, 95% yield.

Applications of metal-free directed C-H borylation strategy

(*E*)-2-(4-(4-*methoxyphenyl*)but-3-en-2-yl)Phenol (4)[26]

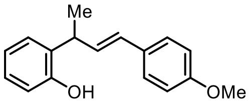

4

To a seal tube was added **3aa** (32 mg, 0.2 mmol), *p*-anisaldehyde tosylhydrazone (1.3 equiv), K_2CO_3 (36 mg, 1.3 equiv), and dioxane (2.0 mL) under nitrogen stream. The reaction mixture was heated to 110 °C and stirred for 1.5 h. After cooling to room temperature, the mixture was transferred to a separatory funnel, and extracted with ethyl acetate (EA, 10 mL×3). The combined organic layers were washed with brine, dried with Na_2SO_4, filtered, and concentrated in vacuo. The residue was purified by silica gel column chromatography (eluent: PE/EA = 10/1) to afford the desired product **4**. White solid, 46 mg, 90% yield, Mp: 101–103 °C; **^{1}H NMR** (600 MHz, MeOD) δ 7.27 (d, *J* = 8.4 Hz, 2H), 7.12 (d, *J* = 7.5 Hz, 1H), 6.99 (t, *J* = 7.6 Hz, 1H), 6.82 (d, *J* = 8.4 Hz, 2H), 6.80 – 6.74 (m, 2H), 6.32 (d, *J* = 4.4 Hz, 2H), 4.02 – 3.95 (m, 1H), 3.76 (s, 3H), 1.38 (d, *J* = 7.0 Hz, 3H); **^{13}C NMR** (151 MHz, MeOD) δ 160.2, 155.6, 133.9, 133.6, 132.1, 128.7, 128.5, 128.1, 127.8, 120.7, 116.1, 114.9, 55.6, 36.6, 20.7.

(*Z*)-2-(1-(*p*-tolyl)prop-1-en-2-yl)Phenol (5)[27]

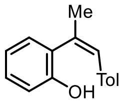

5

To a seal tube was added **3aa** (32 mg, 0.2 mmol), *p*-bromo-toluene (38 mg, 1.2 equiv), $Pd(PPh_3)_4$ (12 mg, 5 mol %), K_2CO_3 (2.0 equiv, 2.0 M aq), and THF (2.0 mL) under nitrogen stream. The mixture was heated to 80 °C and stirred for 5 h. After cooling to room temperature, the mixture was transferred to a separatory funnel, and extracted with ethyl acetate (EA, 10 mL×3). The combined organic layers were washed with brine, dried with Na_2SO_4, filtered, and concentrated in vacuo. The residue was purified by silica gel column chromatography (eluent: PE/EA = 5/1) to obtain product **5**. White solid, 35 mg, 78% yield; Mp: 160–162 °C; **^{1}H NMR** (600 MHz, MeOD) δ 7.09 (t, *J* = 7.7 Hz, 1H), 6.89 – 6.86 (m, H), 6.83 (m, 5H), 6.74 (t, *J* = 7.4 Hz, 1H), 6.48 – 6.43 (m, 1H), 2.17 (s, 3H), 2.13 (s, 3H); **^{13}C NMR** (151 MHz, MeOD) δ 155.1, 136.7, 136.2, 130.7, 130.6, 129.4, 129.4, 129.2, 128.6, 121.0, 116.7, 26.6, 21.1.

4-Methyl-3,4-dihydro-2H-benzo[e][1,2]oxaborinin-2-ol (6)

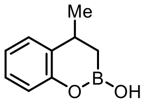

6

To a high-pressure reactor vessel was added **3aa** (32 mg, 0.2 mmol), Pd/C (10 mol %), and MeOH (5.0 mL). The reaction mixture was evacuated, filled with H_2 three times, and stirred under H_2 atmosphere (6 atm) at room temperature for 1.5 h . The reaction mixture was filtered through a celite pad, washed with ethyl acetate (4 mL, 2 times). The filtrate was concentrated under reduced pressure and the residue was purified by silica gel column chromatography (eluent: PE/EA = 10/1) to give product **6**. White solid, 29

mg, 90% yield; Mp: 155–156 °C; **^{1}H NMR** (600 MHz, MeOD) δ 7.10 – 7.07 (m, 2H), 6.95 – 6.87 (m, 2H), 2.94 – 2.87 (m, 1H), 1.20 (d, *J* = 16.5 Hz, 1H), 1.21 – 1.15 (m, 4H), 0.91 – 0.88 (m, 1H); **^{13}C NMR** (151 MHz, MeOD) δ 153.5, 133.8, 128.6, 128.3, 123.7, 119.1, 31.5, 23.9; **^{11}B NMR** (193 MHz, MeOD) δ 32.21; **HRMS (ESI)**: Exact mass calcd for $C_9H_{11}BNaO_2$[M+Na]$^+$: 185.0750, Found: 185.0751.

4-Methyl-2H-chromen-2-one (7)[28]

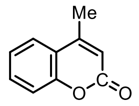

7

To a 25 mL round bottom flask was added to $Pd(OAc)_2$ (5 mg, 10 mol %), **3aa** (32 mg, 0.2 mmol), and DMSO (4 mL). The reaction mixture was evacuated and filled with CO three times. MeOH (2 mL) were then added and the reaction mixture was stirred at room temperature for 3 h until the brown catalyst turned to a black precipitate. After that the mixture was poured into an Erlenmeyer flask containing 20 mL of water, which was then extracted with ethyl acetate (EA, 10 mL×3). The organic phases were combined, washed with brine, and dried over Na_2SO_4. The solvent was removed reduced pressure and the residue was purified by column chromatography (eluent: PE/EA = 5/1) to give product **7**. White solid, 31 mg, 98% yield; Mp: 64–66 °C; **^{1}H NMR** (600 MHz, MeOD) δ 7.76 (d, *J* = 8.0 Hz, 1H), 7.63 – 7.57 (m, 1H), 7.37 (t, *J* = 7.6 Hz, 1H), 7.33 (d, *J* = 8.2 Hz, 1H), 6.32 (s, 1H), 2.48 (s, 3H); **^{13}C NMR** (151 MHz, MeOD) δ 161.5, 154.0, 153.3, 131.8, 124.8, 124.3, 119.8, 116.4, 114.0, 17.2.

3-Methylbenzofuran (8)[29]

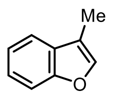

8

To a mixture of $Cu(OAc)_2$ (8 mg, 0.2 equiv), 1,10-phenanthroline (8 mg, 0.22 equiv), and Ag_2CO_3 (165 mg, 3.0 equiv) in ethanol (2.0 mL) and water (0.1 mL) was added **3aa** (32 mg, 0.2 mmol) at room temperature. After stirring for 22 h at 80 °C under air, the reaction mixture was cooled to room temperature and filtered through a cotton-plugged funnel using hexane (15 mL). The filtrate was concentrated under reduced pressure and the residue was purified by silica gel column chromatography (eluent: PE/EA = 10/1) to give product **8**. Colorless oil, 24 mg, 91% yield; **^{1}H NMR** (600 MHz, $CDCl_3$) δ 7.53 (d, *J* = 7.6 Hz, 1H), 7.45 (d, *J* = 8.1 Hz, 1H), 7.41 (s, 1H), 7.29 (t, *J* = 7.6 Hz, 1H), 7.24 (d, *J* = 7.4 Hz, 1H), 2.25 (s, 3H); **^{13}C NMR** (151 MHz, $CDCl_3$) δ 155.2, 141.3, 129.0, 124.0, 122.2, 119.4, 115.6, 111.3, 7.9.

3-Methylbenzofuran-2(3H)-one (9)[29]

To a stirring solution of **3aa** (32 mg, 0.2 mmol) in THF/EtOH (2.0 mL/0.5 mL) was added dropwise 3.0 M NaOH (1.0 mL) and 30% H_2O_2 (1.0 mL) at room temperature. The reaction mixture was stirred under air for 10 min and the most solvents were removed under reduced pressure. The resi-

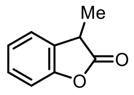

9

due was then directly purified by silica gel column chromatography (eluent: PE/EA = 5/1) to obtain product **9**. White solid, 27 mg, 92% yield, Mp: 60–62 °C; **^{1}H NMR** (600 MHz, $CDCl_3$) δ 7.14 (d, *J* = 7.2 Hz, 1H), 7.05 (t, *J* = 7.7 Hz, 1H), 6.78 (t, *J* = 7.6 Hz, 2H), 4.01 (q, *J* = 7.2 Hz, 1H), 1.41 (d, *J* = 7.2 Hz, 3H); **^{13}C NMR** (151 MHz, $CDCl_3$) δ 179.3, 155.8, 129.0, 128.9, 128.8, 120.6, 116.1, 40.4, 17.7.

1,3-Dimethyl-1H-indole (10)[30]

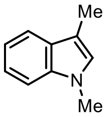

10

To a stirring solution of **3bh** (35 mg, 0.2 mmol) in THF/EtOH (2.0 mL/0.5 mL) was added dropwise 3.0 M NaOH (1.0 mL) and 30% H_2O_2 (1.0 mL) at room temperature. The reaction mixture was stirred under air for 10 min and the most solvents were removed under reduced pressure. The residue was then directly purified by silica gel column chromatography (eluent: PE/EA = 5/1) to obtain product **10**. White solid, 28 mg, 95% yield, Mp: 139–142 °C; **^{1}H NMR** (600 MHz, $CDCl_3$) δ 7.13 (d, *J* = 7.2 Hz, 1H), 7.05 (t, *J* = 7.7 Hz, 1H), 6.78 (t, *J* = 7.6 Hz, 2H), 4.01 (q, *J* = 7.2 Hz, 1H), 1.41 (d, *J* = 7.2 Hz, 3H); **^{13}C NMR** (151 MHz, $CDCl_3$) δ 179.3, 155.8, 129.0, 128.9, 128.8, 120.6, 116.1, 40.4, 17.7.

7-(2-hydroxynaphthalen-1-yl)-1,2,3,4-Tetrahydro-5H-benzo[c]naphtho[2,3-e][1,2]oxaborinin-5-ol (3bz)

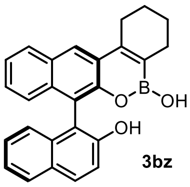

3bz

Eluent: PE/EA (5:1 to 4:1), white solid, 60 mg, 77% yield; Mp: 171–173 °C; **^{1}H NMR** (600 MHz, MeOD) δ 8.17 (s, 1H), 7.93 (d, *J* = 8.3 Hz, 1H), 7.87 (d, *J* = 8.9 Hz, 1H), 7.83 (d, *J* = 8.1 Hz, 1H), 7.34 (t, *J* = 6.3 Hz, 1H), 7.31 (d, *J* = 8.9 Hz, 1H), 7.28 – 7.20 (m, 3H), 7.17 – 7.09 (m, 1H), 6.95 (d, *J* = 8.5 Hz, 1H), 2.84 (td, *J* = 6.3, 2.0 Hz, 2H), 2.34 (q, *J* = 4.3 Hz, 2H), 1.88 (m, 2H), 1.74 – 1.65 (m, 2H); **^{13}C NMR** (151 MHz, MeOD) δ 153.8, 149.1, 148.5, 135.7, 134.1, 131.1, 130.3, 130.2, 129.7, 129.0, 127.4, 127.1, 127.0, 126.3, 125.8, 125.1, 124.3, 123.7, 121.0, 119.0, 116.9, 27.1, 26.5, 23.9, 23.2; **^{11}B NMR** (193 MHz, MeOD) δ 27.39; **HRMS (ESI)**: Exact mass calcd for $C_{26}H_{22}BO_3[M+H]^+$: 393.1662, Found: 393.1664.

7-(2-(trityloxy)naphthalen-1-yl)-1,2,3,4-Tetrahydro-5H-benzo[c]naphtho[2,3-e][1,2]oxaborinin-5-ol (11)[31]

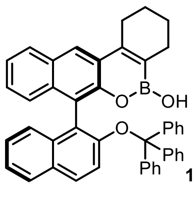

11

Eluent: PE/EA (10:1 to 5:1), white solid, 62 mg, 38% yield; Mp: 192–194 °C; **^{1}H NMR** (600 MHz, DMSO) δ 8.73 (s, 1H), 8.33 (s, 1H), 8.08 (d, *J* = 8.3 Hz, 1H), 7.78 (d, *J* = 8.2 Hz, 1H), 7.56 (d, *J* = 9.2 Hz, 1H), 7.38 (t, *J* = 7.5 Hz, 1H), 7.30 (t, *J* = 7.5 Hz, 1H), 7.26 (t, *J* = 7.6 Hz, 1H), 7.22 (t, *J* = 7.6 Hz, 1H), 7.10 (p, *J* = 7.3 Hz, 15H), 7.03 (d, *J* = 8.5 Hz, 1H), 6.84 (d, *J* = 8.6 Hz, 1H), 6.75 (d, *J* = 9.2 Hz, 1H),

2.96 (t, J = 6.7 Hz, 2H), 2.41 (t, 2H), 1.95 (dt, J = 12.4, 6.8 Hz, 2H), 1.79 – 1.67 (m, 2H); **^{13}C NMR** (151 MHz, DMSO) δ 151.7, 148.3, 144.4, 134.0, 132.4, 129.4, 129.1, 128.7, 128.3, 128.2, 127.6, 127.5, 126.71 126.5, 126.3, 125.21 124.3, 124.0, 123.2, 121.6, 120.3, 119.9, 88.6, 26.4, 26.3, 22.9, 22.3; **^{11}B NMR** (193 MHz, DMSO) δ 27.01; **HRMS (ESI)**: Exact mass calcd for $C_{45}H_{36}BO_3[M+H]^+$: 635.2758, Found: 635.2761.

(*S*)-3-(benzyloxy)-2-phenylpropan-1-ol (13)[31]

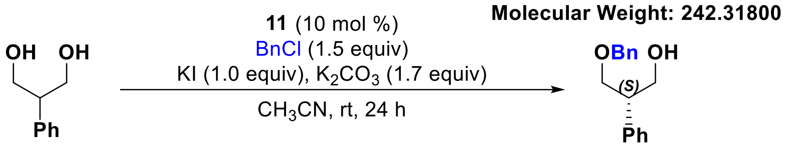

12 (*S*)-13

According to Hall's reported procedure: to a 10 mL Schlenk flask was added the 1,3-diol **12** (0.1 mmol, 15.2 mg), organoboron catalyst **11** (0.01 mmol, 6.4 mg), potassium carbonate (0.170 mmol, 23.5 mg) and potassium iodide (0.100 mmol, 16.6 mg). Acetonitrile (0.5 mL) was then added, followed by benzyl chloride (17 μL, 0.15 mmol). The reaction was stirred at room temperature for 24 hours. After the indicated time, the reaction mixture was filtered through a celite pad with ethyl acetate as solvent and concentrated in vacuo. The residue was then directly purified by silica gel column chromatography (eluent: PE/EA = 10/1) to obtain product **13**. Colorless oil, 12 mg, 51% yield, 85% ee. **^{1}H NMR** (600 MHz, $CDCl_3$): δ 7.42 – 7.22 (m, 10H), 4.59 (s, 2H), 4.04 (dd, J = 10.9, 7.4 Hz, 1H), 3.91 (dd, J = 11.1, 5.3 Hz, 1H), 3.85 (dd, J = 8.9, 8.9 Hz, 1H), 3.79 (dd, J = 9.3, 5.2 Hz, 1H), 3.25 (m, 1H), 2.47 (s, 1H); **^{13}C NMR** (101 MHz, $CDCl_3$): δ 139.62, 137.94, 128.68, 128.49, 128.02, 127.78, 127.67, 127.13, 118.04, 73.56, 73.48, 66.53, 47.75; **HPLC**: Chiralpak IC-3 (150 mm), *n*-hexane/*i*-propanol = 95/5, 0.5 mL/min, detected at 254 nm; Retention time: 15.51 min (major), 16.60 min (minor).

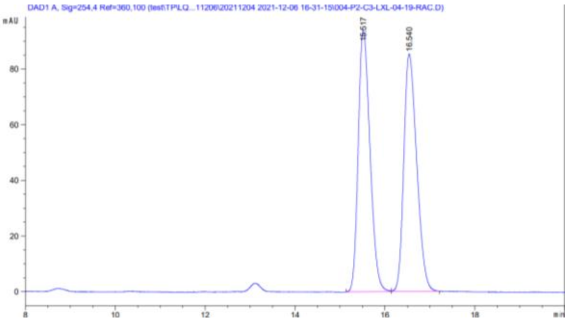

Peak #	RetTime [min]	Type	Width [min]	Area [mAU*s]	Height [mAU]	Area %
1	15.517	BV	0.2668	1713.52783	94.16611	50.1543
2	16.540	VB	0.2967	1702.98633	85.38113	49.8457
Totals :				3416.51416	179.54724	

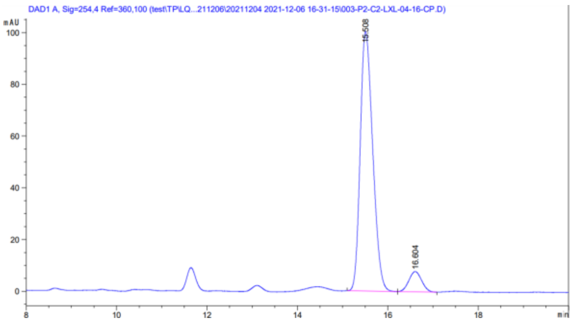

Peak #	RetTime [min]	Type	Width [min]	Area [mAU*s]	Height [mAU]	Area %
1	15.508	BV R	0.2872	1923.12939	100.31904	92.5646
2	16.604	BB	0.2344	154.47856	7.85480	7.4354
Totals :				2077.60796	108.17384	

4-Bromo-5-fluoro-1a,7b-dihydrobenzo[e]cyclopropa[c][1,2]oxaborinin-2(1H)-ol (16)[32]

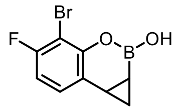

16

To the solution of Et_2Zn (4.0 mL, 1 M in hexanes, 4.0 equiv) in DCM (1 mL) was added dimethoxyethane (42μL, 0.4 mmol, 0.4 equiv) and diiodomethane (1.06 mL, 8.0 mmol, 8 equiv) dropwise in 3 minutes at –78 °C under nitrogen atmosphere. The resulting white mixture was stirred at –78 °C for 10 minutes before compound **3au** (256 mg, 1.0 mmol, 1.0 equiv) in DCM (6 mL) was added dropwise in 5 minutes. The solution was slowly warmed up to rt in 6 hours and stirred for 24 hours. The reaction mixture was quenched with saturated aqueous ammonium chloride solution was extracted with EA (8 mL × 3). The combined organic layer was dried over Na_2SO_4 and then concentrated to dryness. The residue was briefly purified by column chromatography (PE/EA = 3/1 to 1/1) to give **16** as yellow oil 172 mg, 67% yield; **^{1}H NMR** (600 MHz, MeOD) δ 7.29 – 7.23 (m, 1H), 6.79 (t, *J* = 8.4 Hz, 1H), 2.26 (dq, *J* = 7.9, 4.2 Hz, 1H), 1.33 (td, *J* = 9.3, 7.9, 3.5 Hz, 1H), 0.58 (ddd, *J* = 10.3, 8.2, 6.3 Hz, 1H), 0.31 (dt, *J* = 5.8, 3.1 Hz, 1H); **^{13}C NMR** (151 MHz, $CDCl_3$) δ 160.2, 158.6, 150.0, 129.0, 129.0, 126.2, 110.2, 110.0, 101.4, 101.3, 49.0, 17.8, 11.0; **^{11}B NMR** (193 MHz, $CDCl_3$) δ 28.30; **HRMS (ESI)**: Exact mass calcd for $C_9H_6BBrFO_2[M-H]^-$: 254.9628, Found: 254.9634.

Lithium 5-fluoro-2-oxido-1,1a,2,7b-tetrahydrobenzo[e]cyclopropa[c][1,2]oxaborinine-4-carboxylate (17)[32,33]

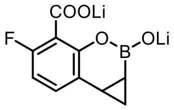

17

To the solution of **16** (51 mg, 0.2 mmol) in THF (1.0 mL) was added 1.6 M ^{n}BuLi (0.25 ml, 2.0 equiv) dropwise in 15 minutes at –78 °C under nitrogen atmosphere. The resulting mixture was added to dry ice at –78 °C. The solution was then slowly warmed up to rt and stirred for 30 min. After that the reaction was quenched by distilled water (1.0 mL) and transferred to a separatory funnel. The mixture was diluted with diethyl ether (2.0 mL) and the organic layer was extracted with distilled water (1.0 mL ×3). The combined aqueous layer was then washed with diethyl ether (1.0 mL×2) and concentrated by freeze-drying to get **17**. White solid, 34 mg, 75% yield; Mp > 300 °C; **^{1}H NMR** (600 MHz, D_2O) δ 6.90 (t, *J* = 7.5 Hz, 1H), 6.35 (t, *J* = 8.6 Hz, 1H), 1.61 – 1.57 (m, 1H), 0.62 – 0.59 (m, 1H), 0.08 – 0.00 (m, 2H); **^{13}C NMR** (151 MHz, D_2O) δ 165.0, 157.9, 156.3, 153.3, 129.1, 129.1, 126.7, 126.6, 105.0, 104.9, 99.5, 99.4, 15.6, 10.5; **^{11}B NMR** (193 MHz, D_2O) δ 3.20; **^{19}F NMR** (565 MHz, D_2O) δ -112.76; **HRMS (ESI)**: Exact mass calcd for $C_{10}H_7BFLi_2O_4[M+H]^+$: 235.0742, Found: 235.0747.

Supplementary Crystallographic Data

Supplementary Note 1:
The Single Crystal Data of 3aa (CCDC 2143877)

Sample preparation: **3aa**

A solution of the substance (15.4 mg) is prepared using methanol (200 μL) and placed in test tube. A second solvent, dichloromethane, is placed in a closed beaker, The test tube containing methanol is then placed in the beaker and the beaker is sealed. Slow diffusion of methanol into test tube and dichloromethane out of test tube will cause crystals to form.

Crystal measurement for compounds **3aa**:

A total of 800 frames were collected. The total exposure time was 0.44 hours. The frames were integrated with the Bruker SAINT software package using a narrow-frame algorithm. The integration of the data using an orthorhombic unit cell yielded a total of 28422 reflections to a maximum θ angle of 28.32 (0.75 Å resolution), of which 4295 were independent (average redundancy 6.617, completeness = 99.9%, R_{int} = 12.04%, R_{sig} = 7.21%) and 2218 (51.64%) were greater than $2\sigma(F^2)$. The final cell constants of a = 7.0450(12) Å, b = 14.852(2) Å, c = 16.556(2) Å, volume = 1732.3(5) $Å^3$, are based upon the refinement of the XYZ-centroids of 3142 reflections above 20 σ(I) with 5.485° < 2θ < 40.27°. Data were corrected for absorption effects using the Multi-Scan method (SADABS). The ratio of minimum to maximum apparent transmission was 0.842.

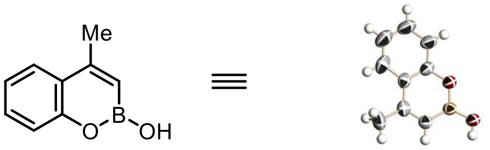

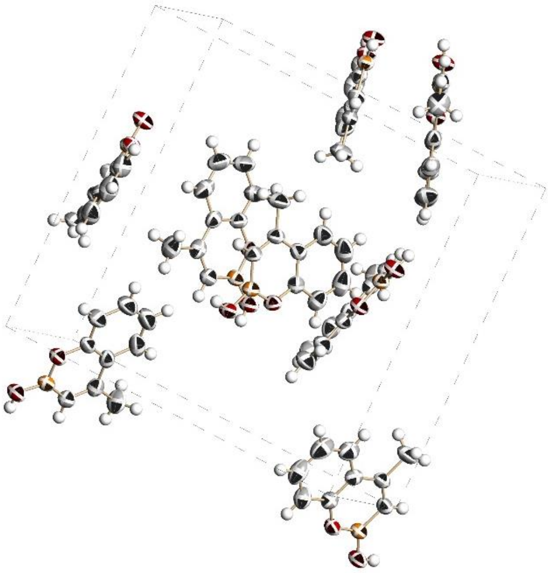

Table S1. Crystal data and structure refinement for **3aa**

**Identification code**	202201200763	
**Chemical formula**	$C_{18}H_{18}B_2O_4$	
**Formula weight**	319.94 g/mol	
**Temperature**	295(2) K	
**Wavelength**	0.71073 Å	
**Crystal system**	orthorhombic	
**Space group**	P 21 21 21	
**Unit cell dimensions**	a = 7.0450(12) Å	α = 90°
	b = 14.852(2) Å	β = 90°
	c = 16.556(2) Å	γ = 90°
**Volume**	1732.3(5) $Å^3$	
**Z**	4	
**Density (calculated)**	1.227 g/cm^3	
**Absorption coefficient**	0.083 mm^{-1}	
**F(000)**	672	
**Diffractometer**	d8 venture	
**Theta range for data collection**	2.46 to 28.32°	
**Index ranges**	-9<=h<=9, -19<=k<=19, -22<=l<=20	
**Reflections collected**	28422	
**Independent reflections**	4295 [R(int) = 0.1204]	
**Coverage of independent reflections**	99.9%	
**Absorption correction**	Multi-Scan	
**Structure solution technique**	direct methods	
**Structure solution program**	SHELXT 2018/2 (Sheldrick, 2018)	
**Refinement method**	Full-matrix least-squares on F2	
**Refinement program**	SHELXL-2018/3 (Sheldrick, 2018)	
**Function minimized**	Σ w(Fo2 - Fc2)2	
**Data / restraints / parameters**	4295 / 0 / 221	
**Goodness-of-fit on F2**	1.034	
**Δ/σmax**	0.001	
**Final R indices**	2218 data; I>2σ(I)	
	all data	
**Weighting scheme**	$w=1/[\sigma^2(F_o^2)+(0.0489P)^2]$ where $P=(F_o^2+2F_c^2)/3$	
**Absolute structure parameter**	-1.0(9)	
	0.121 and -0.178 $eÅ^{-3}$	
**Largest diff. peak and hole**	0.039 $eÅ^{-3}$	
**R.M.S. deviation from mean**	202201200763	

Supplementary Note 2:

The Single Crystal Data of 3ah (CCDC 2143875)

Sample preparation: **3ah**

A solution of the substance (12.2 mg) is prepared using methanol (200 μL) and placed in test tube. A second solvent, *n*-hexane, is placed in a closed beaker, The test tube containing methanol is then placed in the beaker and the beaker is sealed. Slow diffusion of methanol into test tube and *n*-hexane out of test tube will cause crystals to form.

Crystal measurement for compounds **3ah**:

A total of 956 frames were collected. The total exposure time was 0.63 hours. The frames were integrated with the Bruker SAINT software package using a narrow-frame algorithm. The integration of the data using a monoclinic unit cell yielded a total of 12658 reflections to a maximum θ angle of 65.25° (0.85 Å resolution), of which 3018 were independent (average redundancy 4.194, completeness = 99.1%, R_{int} = 6.19%, R_{sig} = 4.97%) and 2419 (80.15%) were greater than 2σ(F^2). The final cell constants of a = 7.4528(2) Å, b = 7.4913(2) Å, c = 31.9715(7) Å, β = 96.4770(10)°, volume = 1773.61(8) $Å^3$, are based upon the refinement of the XYZ-centroids of 5860 reflections above 20 σ(I) with 5.564° < 2θ < 129.9°. Data were corrected for absorption effects using the Multi-Scan method (SADABS). The ratio of minimum to maximum apparent transmission was 0.740. The calculated minimum and maximum transmission coefficients (based on crystal size) are 0.7830 and 0.9250.

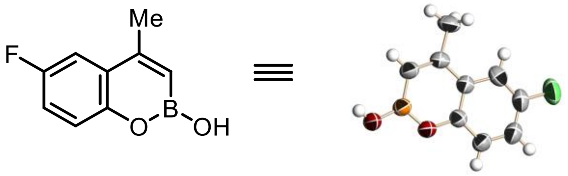

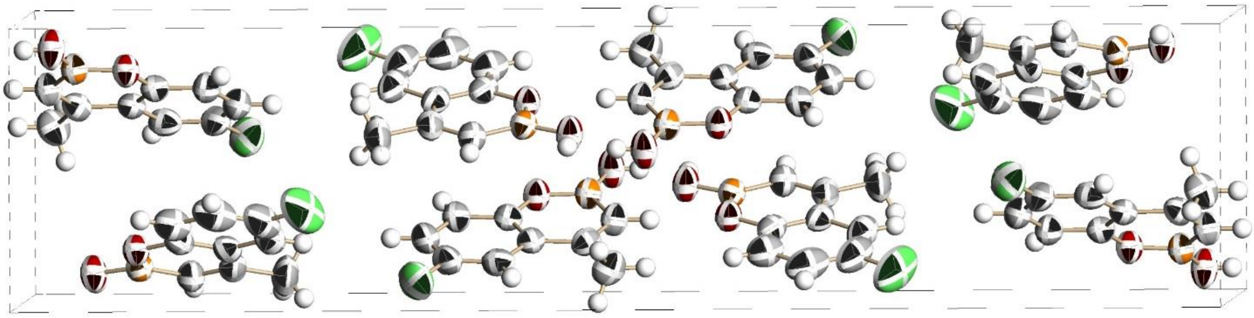

Table S2. Crystal data and structure refinement for **3ah**

**Identification code**	20201010PPY0398	
**Chemical formula**	$C_{18}H_{16}B_2F_2O_4$	
**Formula weight**	355.93 g/mol	
**Temperature**	300(2) K	
**Wavelength**	1.54178 Å	
**Crystal size**	0.090 x 0.150 x 0.290 mm	
**Crystal habit**	clear light colourless block	
**Crystal system**	monoclinic	
**Space group**	P 1 21/n 1	
**Unit cell dimensions**	a = 7.4528(2) Å	α = 90°
	b = 7.4913(2) Å	β = 96.4770(10)°
	c = 31.9715(7) Å	γ = 90°
**Volume**	1773.61(8) $Å^3$	
**Z**	4	
**Density (calculated)**	1.333 g/cm^3	
**Absorption coefficient**	0.885 mm^{-1}	
**F(000)**	736	
**Diffractometer**	d8 venture	
**Theta range for data collection**	2.78 to 65.25°	
**Index ranges**	-7<=h<=8, -8<=k<=8, -37<=l<=33	
**Reflections collected**	12658	
**Independent reflections**	3018 [R(int) = 0.0619]	
**Coverage of independent reflections**	99.1%	
**Absorption correction**	Multi-Scan	
**Max. and min. transmission**	0.9250 and 0.7830	
**Structure solution technique**	direct methods	
**Structure solution program**	SHELXT 2014/5 (Sheldrick, 2014)	
**Refinement method**	Full-matrix least-squares on F2	
**Refinement program**	SHELXL-2018/3 (Sheldrick, 2018)	
**Function minimized**	Σ w(Fo2 - Fc2)2	
**Data / restraints / parameters**	3018 / 0 / 245	
**Goodness-of-fit on F2**	0.600	
**Δ/σmax**	0.025	
**Final R indices**	2419 data; I>2σ(I)	R1 = 0.0522, wR2 = 0.1571
	all data	R1 = 0.0630, wR2 = 0.1783
**Weighting scheme**	$w=1/[\sigma^2(F_o^2)+(0.1747P)^2+1.6599P]$ where $P=(F_o^2+2F_c^2)/3$	
**Largest diff. peak and hole**	0.214 and -0.212 $eÅ^{-3}$	

Supplementary Note 3:

The Single Crystal Data of 3bh (CCDC 2143812)

Sample preparation: **3bh**

A solution of the substance (16.3 mg) is prepared using methanol (200 μL) and placed in test tube. A second solvent, dichloromethane, is placed in a closed beaker, The test tube containing methanol is then placed in the beaker and the beaker is sealed. Slow diffusion of methanol into test tube and dichloromethane out of test tube will cause crystals to form.

Crystal measurement for compounds **3bh**:

A total of 1433 frames were collected. The total exposure time was 1.65 hours. The frames were integrated with the Bruker SAINT software package using a narrow-frame algorithm. The integration of the data using an orthorhombic unit cell yielded a total of 9426 reflections to a maximum θ angle of 64.62° (0.85 Å resolution), of which 2589 were independent (average redundancy 3.641, completeness = 99.7%, R_{int} = 43.78%, R_{sig} = 46.77%) and 663 (25.61%) were greater than $2\sigma(F^2)$. The final cell constants of a = 7.5985(7) Å, b = 8.0704(7) Å, c = 16.6837(15) Å, volume = 1023.09(18) $Å^3$, are based upon the refinement of the XYZ-centroids of 5342 reflections above 20 σ(I) with 12.18° < 2θ < 129.6°. Data were corrected for absorption effects using the Multi-Scan method (SADABS). The ratio of minimum to maximum apparent transmission was 0.805. The calculated minimum and maximum transmission coefficients (based on crystal size) are 0.5549 and 0.7457.

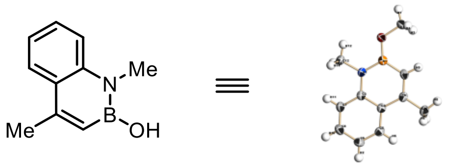

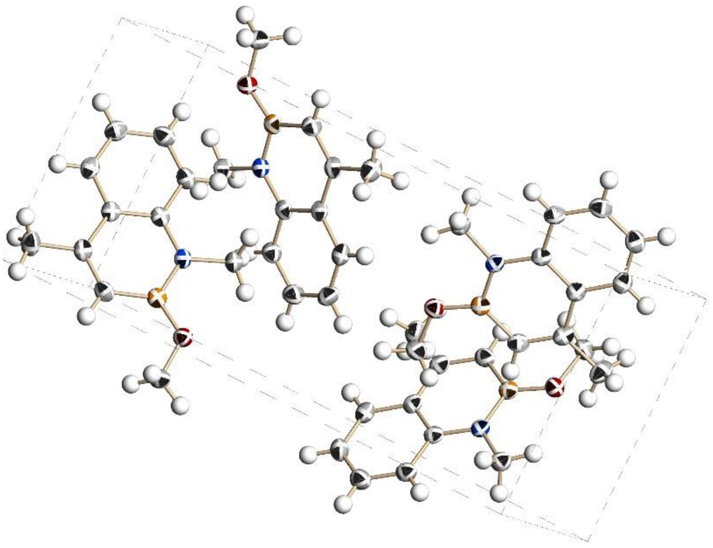

Table S3. Crystal data and structure refinement for 3**bh**

**Identification code**	20210421ppy0561	
**Chemical formula**	$C_{11}H_{15}BNO$	
**Formula weight**	188.05 g/mol	
**Temperature**	186(2) K	
**Wavelength**	1.54184 Å	
**Crystal size**	0.090 x 0.160 x 0.180 mm	
**Crystal habit**	clear light colorless plate	
**Crystal system**	orthorhombic	
**Unit cell dimensions**	a = 7.5985(7) Å	α = 90°
	b = 8.0704(7) Å	β = 90°
	c = 16.6837(15) Å	γ = 90°
**Volume**	1023.09(18) $Å^3$	
**Z**	4	
**Density (calculated)**	1.183 g/cm^3	
**Absorption coefficient**	0.074 mm^{-1}	
**F(000)**	404	
**Diffractometer**	d8 venture	
**Theta range for data collection**	2.41 to 64.62°	
**Index ranges**	-10<=h<=10, -10<=k<=10, -22<=l<=18	
**Reflections collected**	9426	
**Independent reflections**	2589 [R(int) = 0.4378]	
**Coverage of independent reflections**	99.7%	
**Absorption correction**	Multi-Scan	
**Structure solution technique**	direct methods	
**Structure solution program**	SHELXT 2018/2 (Sheldrick, 2018)	
**Refinement method**	Full-matrix least-squares on F2	
**Refinement program**	SHELXL-2018/3 (Sheldrick, 2018)	
**Function minimized**	Σ w(Fo2 - Fc2)2	
**Data / restraints / parameters**	2589 / 0 / 130	
**Goodness-of-fit on F2**	0.933	
**Final R indices**	663 data; I>2σ(I)	
	all data	
**Weighting scheme**	$w=1/[\sigma^2(F_o^2)+(0.0714P)^2]$	
	where $P=(F_o^2+2F_c^2)/3$	
**Largest diff. peak and hole**	0.207 and -0.245 $eÅ^{-3}$	
**R.M.S. deviation from mean**	0.057 $eÅ^{-3}$	

Supplementary Note 4:

The Single Crystal Data of 3bm (CCDC 2144121)

Sample preparation: **3bm**

A solution of the substance (23.1 mg) is prepared using methanol (200 μL) and placed in test tube. A second solvent, dichloromethane, is placed in a closed beaker, The test tube containing methanol is then placed in the beaker and the beaker is sealed. Slow diffusion of methanol into test tube and dichloromethane out of test tube will cause crystals to form.

Crystal measurement for compounds **3bm**:

A total of 1344 frames were collected. The total exposure time was 0.75 hours. The frames were integrated with the Bruker SAINT software package using a narrow-frame algorithm. The integration of the data using a triclinic unit cell yielded a total of 31029 reflections to a maximum θ angle of 28.44° (0.75 Å resolution), of which 6405 were independent (average redundancy 4.844, completeness = 98.8%, R_{int} = 11.73%, R_{sig} = 10.31%) and 2493 (38.92%) were greater than $2\sigma(F^2)$. The final cell constants of a = 7.5307(8) Å, b = 8.9847(9) Å, c = 19.313(2) Å, α = 81.255(3)°, β = 88.525(5)°, γ = 87.084(4)°, volume = 1289.7(2) $Å^3$, are based upon the refinement of the XYZ-centroids of 2978 reflections above 20 σ(I) with 4.592° < 2θ < 46.72°. Data were corrected for absorption effects using the Multi-Scan method (SADABS). The ratio of minimum to maximum apparent transmission was 0.915.

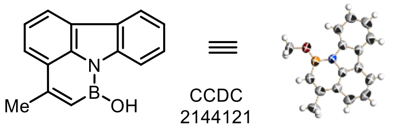

3bm ORTEP of **3bm**

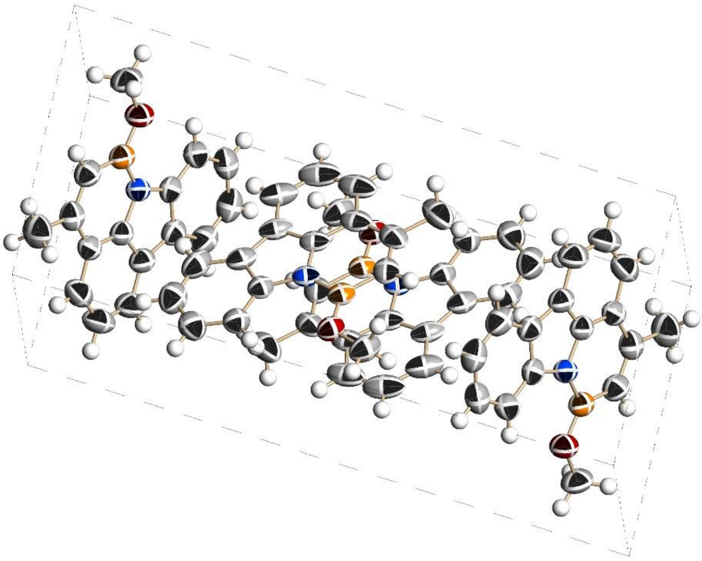

Table S4. Crystal data and structure refinement for **3bm**

**Identification code**	20220122ppy0737	
**Chemical formula**	$C_{32}H_{28}B_2N_2O_2$	
**Formula weight**	494.18 g/mol	
**Temperature**	297(2) K	
**Wavelength**	0.71073 Å	
**Crystal size**	0.100 x 0.150 x 0.200 mm	
**Crystal habit**	clear light colorless block	
**Crystal system**	triclinic	
**Space group**	P -1	
**Unit cell dimensions**	a = 7.5307(8) Å	α = 81.255(3)°
	b = 8.9847(9) Å	β = 88.525(5)°
	c = 19.313(2) Å	γ = 87.084(4)°
**Volume**	1289.7(2) $Å^3$	
**Z**	2	
**Density (calculated)**	1.273 g/cm^3	
**Absorption coefficient**	0.078 mm^{-1}	
**F(000)**	520	
**Diffractometer**	d8 venture	
**Theta range for data collection**	2.13 to 28.44°	
**Index ranges**	-10<=h<=9, -11<=k<=11, -25<=l<=25	
**Reflections collected**	31029	
**Independent reflections**	6405 [R(int) = 0.1173]	
**Coverage of independent reflections**	98.8%	
**Absorption correction**	Multi-Scan	
**Structure solution technique**	direct methods	
**Structure solution program**	SHELXT 2018/2 (Sheldrick, 2018)	
**Refinement method**	Full-matrix least-squares on F2	
**Refinement program**	SHELXL-2018/3 (Sheldrick, 2018)	
**Function minimized**	Σ w(Fo^2 - Fc^2)2	
**Data / restraints / parameters**	6405 / 0 / 347	
**Goodness-of-fit on F2**	1.016	
**Final R indices**	2493 data; I>2σ(I)	R1 = 0.0779, wR2 = 0.1553
	all data	R1 = 0.2199, wR2 = 0.2129
**Weighting scheme**	w=1/[σ2(Fo2)+(0.0801P)2+0.1220P] where P=(Fo^2+2Fc^2)/3	
**Largest diff. peak and hole**	0.184 and -0.214 $Å^{-3}$	
**R.M.S. deviation from mean**	0.044 $Å^{-3}$	

Supplementary Note 5:

The Single Crystal Data of 3bo (CCDC 2143882)

Sample preparation: **3bo**

A solution of the substance (9.5 mg) is prepared using methanol (200 μL) and placed in test tube. A second solvent, dichloromethane, is placed in a closed beaker, The test tube containing methanol is then placed in the beaker and the beaker is sealed. Slow diffusion of methanol into test tube and dichloromethane out of test tube will cause crystals to form.

Crystal measurement for compounds **3bo**:

A total of 1704 frames were collected. The total exposure time was 0.47 hours. The frames were integrated with the Bruker SAINT software package using a narrow-frame algorithm. The integration of the data using a triclinic unit cell yielded a total of 34203 reflections to a maximum θ angle of 28.36° (0.75 Å resolution), of which 5922 were independent (average redundancy 5.776, completeness = 99.8%, Rint = 8.04%, Rsig = 5.39%) and 3445 (58.17%) were greater than 2σ(F2). The final cell constants of a = 7.5430(10) Å, b = 11.731(2) Å, c = 14.371(3) Å, α = 93.335(7)°, β = 99.918(5)°, γ = 106.703(4)°, volume = 1192.0(3) Å3, are based upon the refinement of the XYZ-centroids of 6565 reflections above 20 σ(I) with 5.751° < 2θ < 50.16°. Data were corrected for absorption effects using the Multi-Scan method (SADABS). The ratio of minimum to maximum apparent transmission was 0.901. The calculated minimum and maximum transmission coefficients (based on crystal size) are 0.9500 and 0.9660.

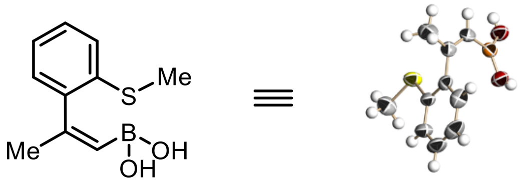

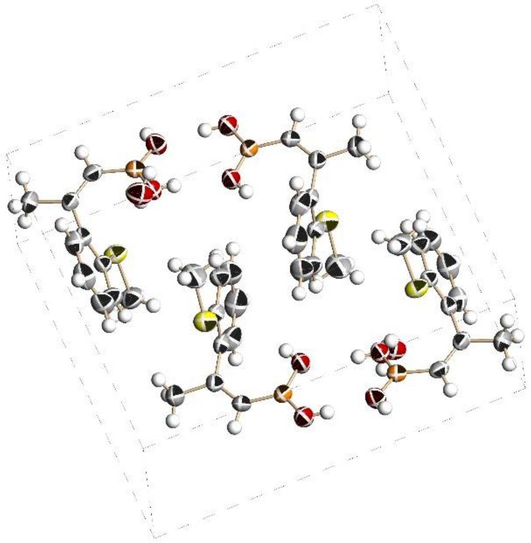

Table S5. Crystal data and structure refinement for **3bo**

**Identification code**	20210119ppy0596	
**Chemical formula**	$C_{22}H_{22}B_{0.50}Cl_2O_5S_{0.50}$	
**Formula weight**	458.73 g/mol	
**Temperature**	296(2) K	
**Wavelength**	0.71073 Å	
**Crystal size**	0.100 x 0.100 x 0.150 mm	
**Crystal habit**	clear light colourless block	
**Crystal system**	triclinic	
**Space group**	P -1	
**Unit cell dimensions**	a = 7.5430(10) Å	α = 93.335(7)°
	b = 11.731(2) Å	β = 99.918(5)°
	c = 14.371(3) Å	γ = 106.703(4)°
**Volume**	1192.0(3) $Å^3$	
**Z**	2	
**Density (calculated)**	1.278 g/cm^3	
**Absorption coefficient**	0.345 mm^{-1}	
**F(000)**	477	
**Diffractometer**	d8 venture	
**Theta range for data collection**	2.20 to 28.36°	
**Index ranges**	-10<=h<=10, -15<=k<=15, -19<=l<=19	
**Reflections collected**	34203	
**Independent reflections**	5922 [R(int) = 0.0804]	
**Coverage of independent reflections**	99.80%	
**Absorption correction**	Multi-Scan	
**Max. and min. transmission**	0.9660 and 0.9500	
**Structure solution technique**	direct methods	
**Structure solution program**	SHELXT 2018/2 (Sheldrick, 2018)	
**Refinement method**	Full-matrix least-squares on F^2	
**Refinement program**	SHELXL-2018/3 (Sheldrick, 2018)	
**Function minimized**	$\Sigma w(F_o^2 - F_c^2)^2$	
**Data / restraints / parameters**	5922 / 0 / 266	
**Goodness-of-fit on F^2**	1.076	
**Δ/σ_{max}**	0.435	
**Final R indices**	3445 data; I>2σ(I)	R1 = 0.0564, wR2 = 0.1640
	all data	R1 = 0.1090, wR2 = 0.1920
**Weighting scheme**	$w=1/[\sigma^2(F_o^2)+(0.1000P)^2]$ where $P=(F_o^2+2F_c^2)/3$	
**Largest diff. peak and hole**	0.379 and -0.298 $eÅ^{-3}$	
**R.M.S. deviation from mean**	0.055 $eÅ^{-3}$	

Supplementary Note 6:

The Single Crystal Data of 3by (CCDC 2196049)

Sample preparation: **3by**

A solution of the substance (8 mg) is prepared using methanol (200 µL) and placed in test tube. A second solvent, dichloromethane, is placed in a closed beaker, The test tube containing methanol is then placed in the beaker and the beaker is sealed. Slow diffusion of methanol into test tube and dichloromethane out of test tube will cause crystals to form.

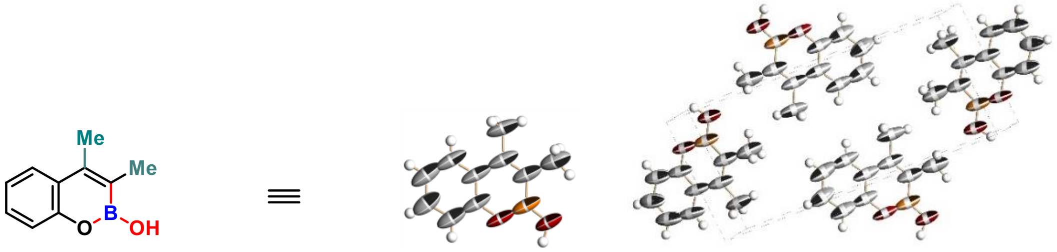

Table S6. Crystal data and structure refinement for **3by**

**Identification code**	cu_20220625PPY0745_0m_a	
**Empirical formula**	$C_{20}H_{22}B_2O_4$	
**Formula weight**	347.99	
**Temperature**	301(2) K	
**Wavelength**	1.54178 Å	
**Crystal system, space group**	Monoclinic, P2(1)/c	
**Unit cell dimensions**	a = 7.3229(11) Å	α = 90 °
	b = 16.640(3) Å	β = 116.085(7) °
	c = 8.2632(9) Å	γ = 90 °
**Volume**	904.3(2) $Å^3$	
**Z**	2	
**Calculated density**	1.278 g/cm^3	
**Absorption coefficient**	0.688 mm^{-1}	
**F(000)**	368	
**Crystal size**	0.15 x 0.1 x 0.1 mm	
**Theta range for data collection**	2.655 to 72.062°	
**Limiting indices**	-9<=h<=8, -20<=k<=19, -10<=l<=9	
**Reflections collected / unique**	8701 / 1804 [R(int) = 0.0522]	
**Completeness to theta = 67.679**	99.90%	
**Refinement method**	Full-matrix least-squares on F^2	
**Data / restraints / parameters**	1804 / 84 / 112	
**Goodness-of-fit on F^2**	1.345	
**Final R indices [I>2sigma(I)]**	R1 = 0.0687, wR2 = 0.1999	
**R indices (all data)**	R1 = 0.1049, wR2 = 0.2293	
**Extinction coefficient**	0.014(5)	
**Largest diff. peak and hole**	0.198 and -0.183 $eÅ^{-3}$	

Supplementary Note 7:
The Single Crystal Data of 4 (CCDC 1970745)

Sample preparation: **4**

A solution of the substance (10 mg) is prepared using methanol (200 μL) and placed in test tube. A second solvent, dichloromethane, is placed in a closed beaker, The test tube containing methanol is then placed in the beaker and the beaker is sealed. Slow diffusion of methanol into test tube and dichloromethane out of test tube will cause crystals to form.

Crystal measurement for compounds **4**:

A total of 1196 frames were collected. The total exposure time was 1.13 hours. The frames were integrated with the Bruker SAINT software package using a narrow-frame algorithm. The integration of the data using an orthorhombic unit cell yielded a total of 20530 reflections to a maximum θ angle of 64.99° (0.85 Å resolution), of which 2339 were independent (average redundancy 8.777, completeness = 98.9%, Rint = 14.60%, Rsig = 5.65%) and 1168 (49.94%) were greater than 2σ(F2). The final cell constants of a = 15.115(7) Å, b = 6.112(4) Å, c = 30.125(15) Å, volume = 2783.(3) Å3, are based upon the refinement of the XYZ-centroids of 1967 reflections above 20 σ(I) with 5.867° < 2θ < 116.4°. Data were corrected for absorption effects using the Multi-Scan method (SADABS). The ratio of minimum to maximum apparent transmission was 0.825. The calculated minimum and maximum transmission coefficients (based on crystal size) are 0.9300 and 0.9520.

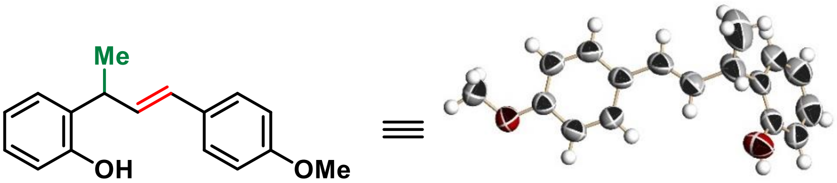

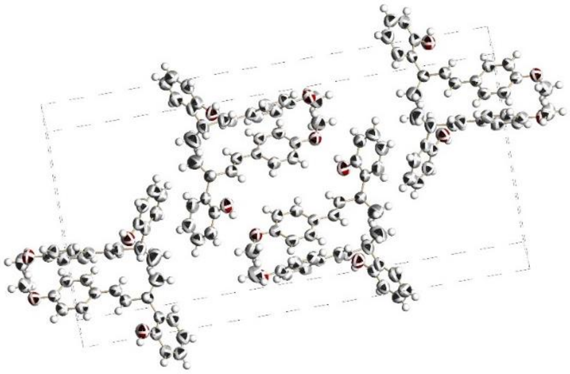

Table S7. Crystal data and structure refinement for **4**

**Identification code**	20210611ppy
**Chemical formula**	$C_{17}H_{18}O_2$
**Formula weight**	254.31 g/mol

**Temperature**	301(2) K	
**Wavelength**	1.54178 Å	
**Crystal size**	0.080 x 0.100 x 0.120 mm	
**Crystal habit**	clear light colourless plate	
**Crystal system**	orthorhombic	
**Space group**	P b c a	
**Unit cell dimensions**	a = 15.115(7) Å	α = 90°
	b = 6.112(4) Å	β = 90°
	c = 30.125(15) Å	γ = 90°
**Volume**	2783.(3) $Å^3$	
**Z**	8	
**Density (calculated)**	1.214 g/cm^3	
**Absorption coefficient**	0.617 mm^{-1}	
**F(000)**	1088	
**Diffractometer**	d8 venture	
**Theta range for data collection**	2.93 to 64.99°	
**Index ranges**	-17<=h<=17, -6<=k<=7, -32<=l<=35	
**Reflections collected**	20530	
**Independent reflections**	2339 [R(int) = 0.1460]	
**Coverage of independent reflections**	98.90%	
**Absorption correction**	Multi-Scan	
**Max. and min. transmission**	0.9520 and 0.9300	
**Structure solution technique**	direct methods	
**Structure solution program**	SHELXT 2018/2 (Sheldrick, 2018)	
**Refinement method**	Full-matrix least-squares on F^2	
**Refinement program**	SHELXL-2018/3 (Sheldrick, 2018)	
**Function minimized**	$\Sigma w(F_o^2 - F_c^2)^2$	
**Data / restraints / parameters**	2339 / 18 / 177	
**Goodness-of-fit on F^2**	1.052	
**Δ/σ_{max}**	0.048	
**Final R indices**	1168 data; I>2σ(I)	R1 = 0.0832, wR2 = 0.2264
	all data	R1 = 0.1461, wR2 = 0.2708
**Weighting scheme**	$w=1/[\sigma^2(F_o^2)+(0.1387P)^2+0.4960P]$ where $P=(F_o^2+2F_c^2)/3$	
**Largest diff. peak and hole**	0.417 and -0.348 $eÅ^{-3}$	
**R.M.S. deviation from mean**	0.054 $eÅ^{-3}$	

Supplementary References

1 Zhao, J.-F., Tsui, H.-Y., Wu, P.-J., Lu, J. & Loh, T.-P. Highly Enantioselective Carbonyl-ene Reactions Catalyzed by In(III)−PyBox Complex. *J. Am. Chem. Soc.* **130**, 16492-16493 (2008).

2 Hu, X.-S., He, J.-X., Dong, S.-Z., Zhao, Q.-H., Yu, J.-S. & Zhou, J. Regioselective Markovnikov hydrodifluoroalkylation of alkenes using difluoroenoxysilanes. *Nat. Commun.* **11**, 5500 (2020).

3 Wang, H., Gao, Y., Zhou, C. & Li, G. Visible-Light-Driven Reductive Carboarylation of Styrenes with CO_2 and Aryl Halides. *J. Am. Chem. Soc.* **142**, 8122-8129 (2020).

4 Greenhalgh, M. D. & Thomas, S. P. Iron-catalyzed, highly regioselective synthesis of alpha-aryl carboxylic acids from styrene derivatives and CO_2. *J. Am. Chem. Soc.* **134**, 11900-11903 (2012).

5 Sangaiah, R. & Rao, G. S. K. Revision of the structure assigned to a monoterpene isolated from. *Tetrahedron Lett.* **22**, 1843-1844 (1981).

6 Barbe, G., Bohnert, G., Calandra, N., Lambert, M. H., III;, Lu, H., Lobera, M., Ramanjulu, J., Ren, F. & Yang, T. Preparation of functionalized nicotineamides, benzamides and related amides as ROR-γ inhibitors for the treatment of ROR-γ-mediated diseases. WO2019063748 (2019).

7 Altenbach, R. J., Bogdan, A., Desroy, N., Gfesser, G., A.;, Greszler, S., N.;, Koenig, J., R.;, Kym, P., R.;, Liu, B., Scanio, M. J. & Searle, X. Preparation of substituted pyrrolidines as CFTR modulators. WO2018065921 (2018).

8 Cheng, W. L. & Luh, T. Y. Tandem Pummerer-type rearrangement and nickel-catalyzed alkylative olefination of the cyclic dithioacetal-S-oxides of aromatic aldehydes with Grignard reagents. *J. Org. Chem.* **57**, 3516-3518 (1992).

9 Zhang, H. & Lu, Z. Dual-Stereocontrol Asymmetric Cobalt-Catalyzed Hydroboration of Sterically Hindered Styrenes. *ACS Catal.* **6**, 6596-6600 (2016).

10 Ohmura, T., Kusaka, S., Torigoe, T. & Suginome, M. Iridium-Catalyzed C(*sp*^{3})−H Addition of Methyl Ethers across Intramolecular Carbon–Carbon Double Bonds Giving 2,3-Dihydrobenzofurans. *Adv. Synth. Catal.* **361**, 4448-4453 (2019).

11 Tasker, S. Z., Gutierrez, A. C. & Jamison, T. F. Nickel-catalyzed Mizoroki-Heck reaction of aryl sulfonates and chlorides with electronically unbiased terminal olefins: high selectivity for branched products. *Angew. Chem. Int. Ed.* **53**, 1858-1861 (2014).

12 Berger, M., Rehwinkel, H., Schaecke, H., Baeurle, S. & Schmees, N. Preparation of 1-aminotetralines as anti-inflammatory agents. WO2007104582 (2007).

13 Seoane, A., Casanova, N., Quiñones, N., Mascareñas, J. L. & Gulías, M. Straightforward Assembly of Benzoxepines by Means of a Rhodium(III)-Catalyzed C–H Functionalization of o-Vinylphenols. *J. Am. Chem. Soc.* **136**, 834-837 (2014).

14 Dong, X., Han, Y., Yan, F., Liu, Q., Wang, P., Chen, K., Li, Y., Zhao, Z., Dong, Y. & Liu, H. Palladium-Catalyzed 6-Endo Selective Alkyl-Heck Reactions: Access to 5-Phenyl-1,2,3,6-tetrahydropyridine Derivatives. *Org. Lett.* **18**, 3774-3777 (2016).

15 Weller, D. D. & Weller, D. L. Synthesis of 3-methyl-2,3,4,4a,5,6-hexahydro-1H-benzofuro[3,2-e]isoquinoline-7(7aH)-ones. *Tetrahedron Lett.* **23**, 5239-5242 (1982).

16 Yasui, Y., Kamisaki, H. & Takemoto, Y. Enantioselective synthesis of 3,3-disubstituted oxindoles through Pd-catalyzed cyanoamidation. *Org. Lett.* **10**, 3303-3306 (2008).

17 Vieira, T. O. & Alper, H. Rhodium(i)-catalyzed hydroaminomethylation of 2-isopropenylanilines as a novel route to 1,2,3,4-tetrahydroquinolines. *Chem. Commun.*, 2710-2711 (2007).

18 Bennett, M. A., Heath, G. A., Hockless, D. C. R., Kovacik, I. & Willis, A. C. Alkene Complexes of Divalent and Trivalent Ruthenium Stabilized by Chelation. Dependence of Coordinated Alkene Orientation on Metal Oxidation State. *J. Am. Chem. Soc.* **120**, 932-941 (1998).

19 Prusek, O. B., Filip; Pytela, Oldrich. ortho-Effect on the acid-catalyzed hydration of 2-substituted α-methylstyrenes. *Collect. Czech. Chem. Commun.* **74**, 85-99 (2009).

20 Thiery, E., Chevrin, C., Le Bras, J., Harakat, D. & Muzart, J. Mechanistic insights into the palladiumII-catalyzed hydroxyalkoxylation of 2-allylphenols. *J. Org. Chem.* **72**, 1859-1862 (2007).

21 Jimenez-Oses, G., Liu, P., Matute, R. A. & Houk, K. N. Competition between concerted and stepwise dynamics in the triplet di-pi-methane rearrangement. *Angew. Chem. Int. Ed.* **53**, 8664-8667 (2014).

22 Das, M. & O'Shea, D. F. Z-Stereoselective Aza-Peterson Olefinations with Bis(trimethylsilane) Reagents and Sulfinyl Imines. *Org. Lett.* **18**, 336-339 (2016).

23 Odedra, A., Datta, S. & Liu, R. S. Ruthenium-catalyzed cyclization of 2-alkyl-1-ethynylbenzenes via a 1,5-hydrogen shift of ruthenium-vinylidene intermediates. *J. Org. Chem.* **72**, 3289-3292 (2007).

24 Artuso, E., Barbero, M., Degani, I., Dughera, S. & Fochi, R. Arenediazonium o-benzenedisulfonimides as efficient reagents for Heck-type arylation reactions. *Tetrahedron* **62**, 3146-3157 (2006).
25 Nguyen, T. N. T., Thiel, N. O. & Teichert, J. F. Copper(I)-catalysed asymmetric allylic reductions with hydrosilanes. *Chem. Commun.* **53**, 11686-11689 (2017).
26 Barluenga, J., Tomás-Gamasa, M., Aznar, F. & Valdés, C. Metal-free carbon–carbon bond-forming reductive coupling between boronic acids and tosylhydrazones. *Nat. Chem.* **1**, 494-499 (2009).
27 Godfrey, C. R. A., Hegarty, P., Motherwell, W. B. & Uddin, M. K. A novel route to unsymmetrical stilbene derivatives via intramolecular free radical ipso substitution reactions. *Tetrahedron Lett.* **39**, 723-726 (1998).
28 Zhou, Q. J., Worm, K. & Dolle, R. E. 10-hydroxy-10,9-boroxarophenanthrenes: versatile synthetic intermediates to 3,4-benzocoumarins and triaryls. *J. Org. Chem.* **69**, 5147-5149 (2004).
29 Sumida, Y., Harada, R., Sumida, T., Johmoto, K., Uekusa, H. & Hosoya, T. Synthesis of Dibenzofurans by Cu-Catalyzed Deborylative Ring Contraction of Dibenzoxaborins. *Org. Lett.* **22**, 6687-6691 (2020).
30 van de Wouw, H. L., Lee, J. Y., Awuyah, E. C. & Klausen, R. S. A BN Aromatic Ring Strategy for Tunable Hydroxy Content in Polystyrene. *Angew. Chem. Int. Ed.* **57**, 1673-1677 (2018).
31 Estrada, C. D., Ang, H. T., Vetter, K. M., Ponich, A. A. & Hall, D. G. Enantioselective Desymmetrization of 2-Aryl-1,3-propanediols by Direct O-Alkylation with a Rationally Designed Chiral Hemiboronic Acid Catalyst That Mitigates Substrate Conformational Poisoning. *J. Am. Chem. Soc.* **143**, 4162-4167 (2021).
32 Hecker, S. J., Reddy, K. R., Lomovskaya, O., Griffith, D. C., Rubio-Aparicio, D., Nelson, K., Tsivkovski, R., Sun, D., Sabet, M., Tarazi, Z., Parkinson, J., Totrov, M., Boyer, S. H., Glinka, T. W., Pemberton, O. A., Chen, Y. & Dudley, M. N. Discovery of Cyclic Boronic Acid QPX7728, an Ultrabroad-Spectrum Inhibitor of Serine and Metallo-beta-lactamases. *J. Med. Chem.* **63**, 7491–7507 (2020).
33 Durka, K., Luliński, S., Dąbrowski, M. & Serwatowski, J. Is Carbon Dioxide Able to Activate Halogen/Lithium Exchange. *Eur. J. Org. Chem.* **2014**, 4562-4570 (2014).
