# Supplementary material for: A practical preparation of bicyclic boronates via metal-free heteroatom-directed alkenyl sp2-C‒H borylation
Source: Commun Chem. 2023 Aug 23;6:176. doi: 10.1038/s42004-023-00976-5 (PMC10447525; doi:10.1038/s42004-023-00976-5)
Supplement: Supplementary file 3 — Description of Additional Supplementary Files [file 42004_2023_976_MOESM3_ESM.pdf]

Description of Additional Supplementary Files

File name: Supplementary Data 1

Description: NMR spectra

File name: Supplementary Data 2

Description: CIF file of 3aa

File name: Supplementary Data 3

Description: CIF file of 3ah

File name: Supplementary Data 4

Description: CIF file of 3bh

File name: Supplementary Data 5

Description: CIF file of 3bm

File name: Supplementary Data 6

Description: CIF file of 3bo

File name: Supplementary Data 7

Description: CIF file of 3by

File name: Supplementary Data 8

Description: CIF file of 4
