# Supplementary figures and images for: A practical preparation of bicyclic boronates via metal-free heteroatom-directed alkenyl sp2-C‒H borylation
Source: Commun Chem. 2023 Aug 23;6:176. doi: 10.1038/s42004-023-00976-5 (PMC10447525; doi:10.1038/s42004-023-00976-5)

# **Supplementary Copies of $^1\text{H}$ , $^{13}\text{C}$ , $^{11}\text{B}$ , and $^{19}\text{F}$ NMR Spectra**

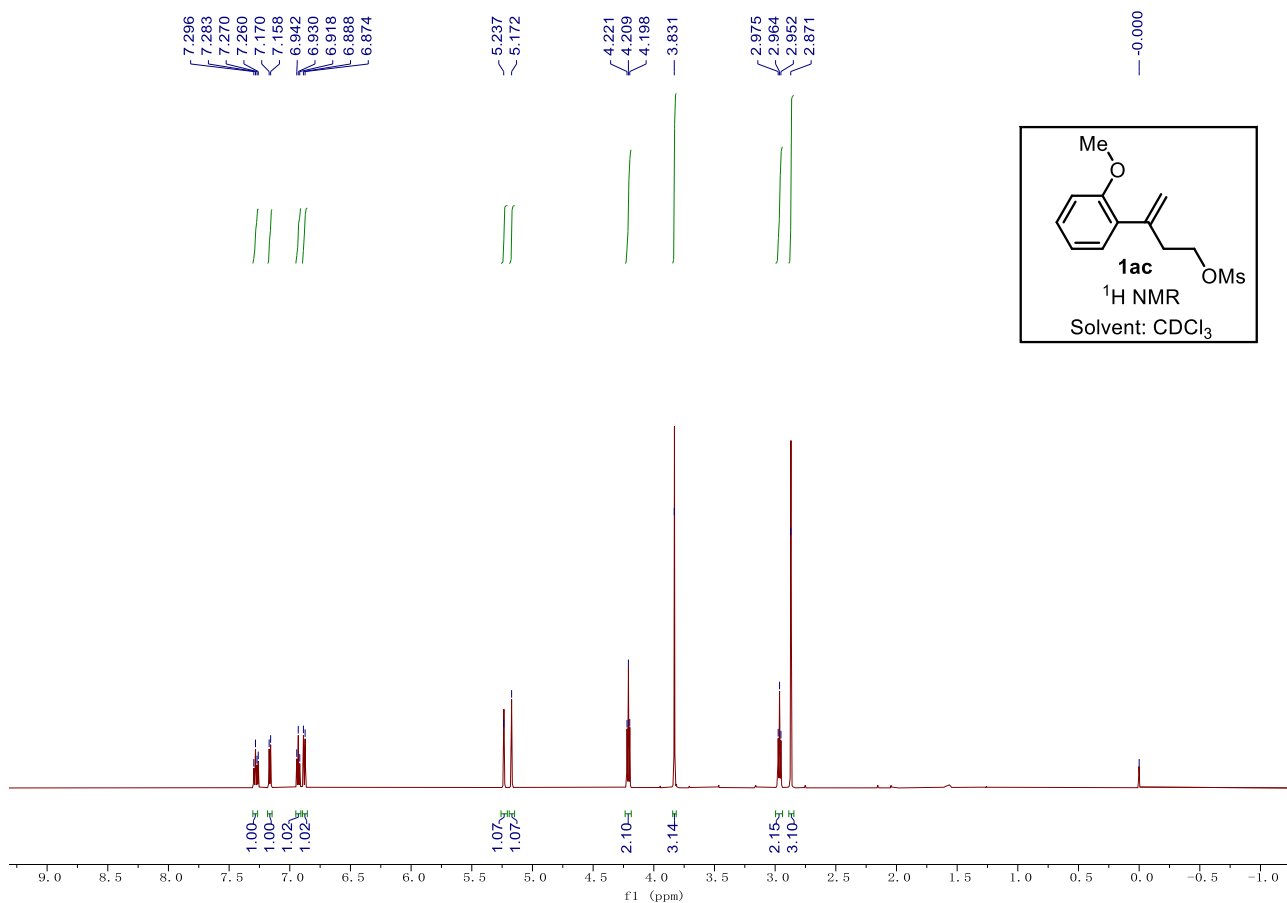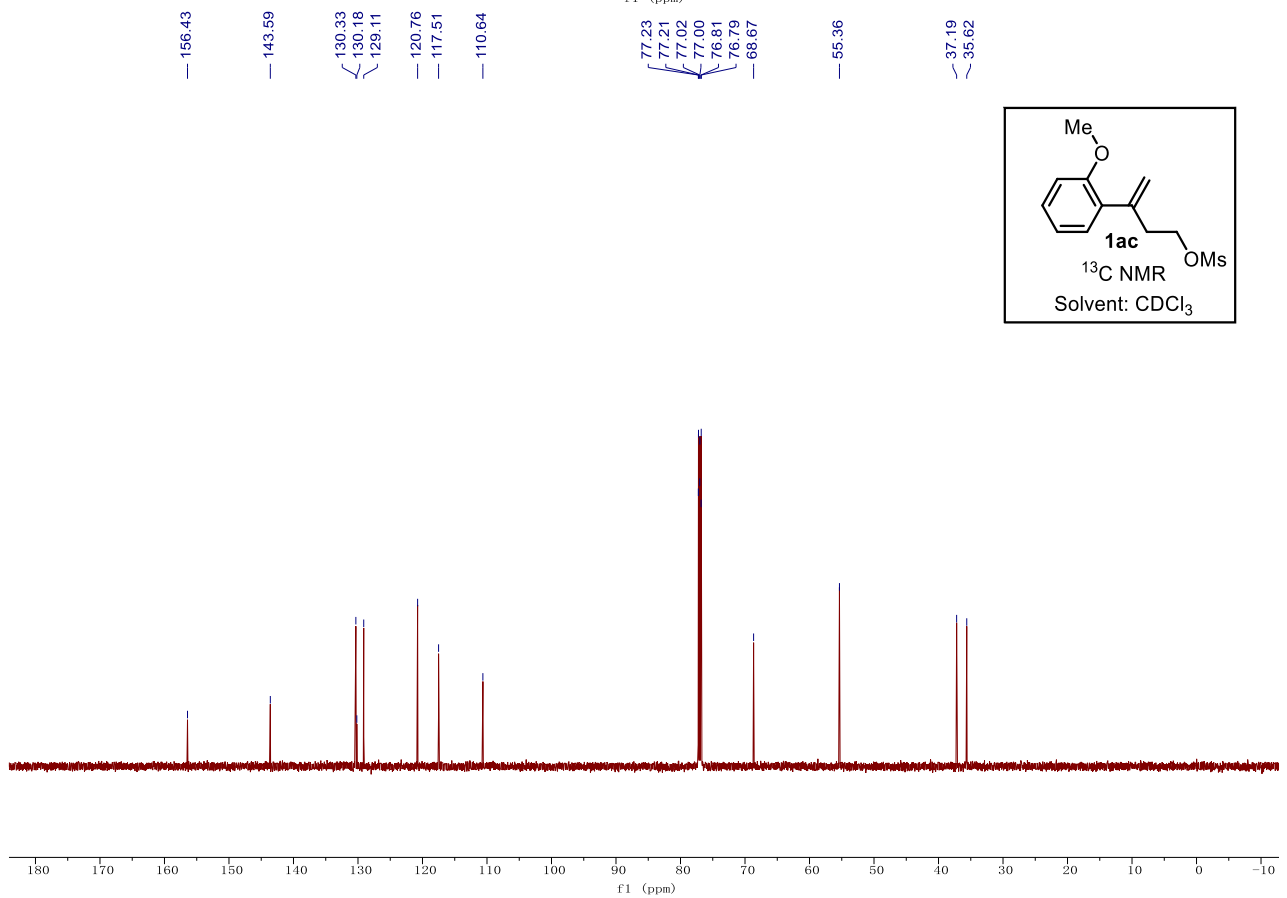

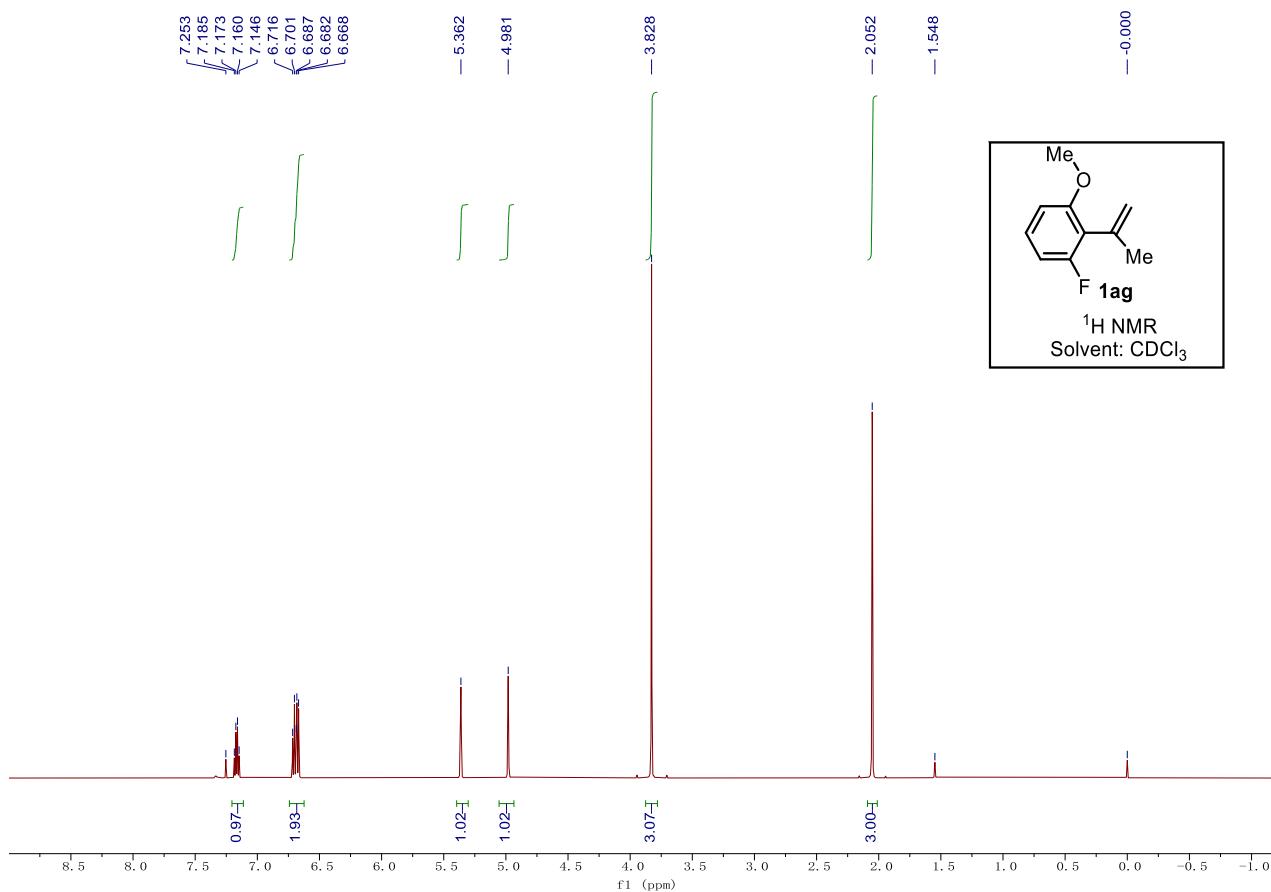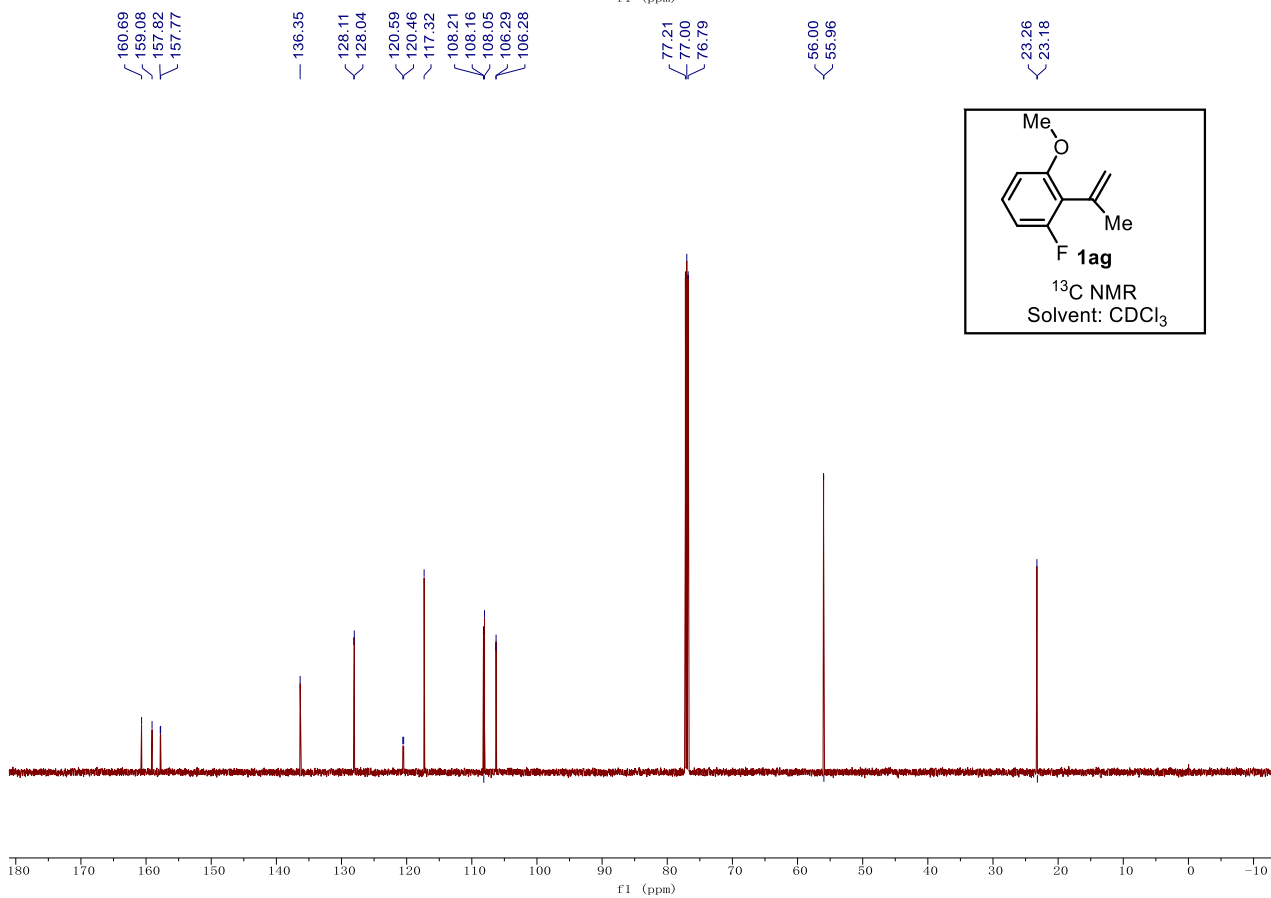

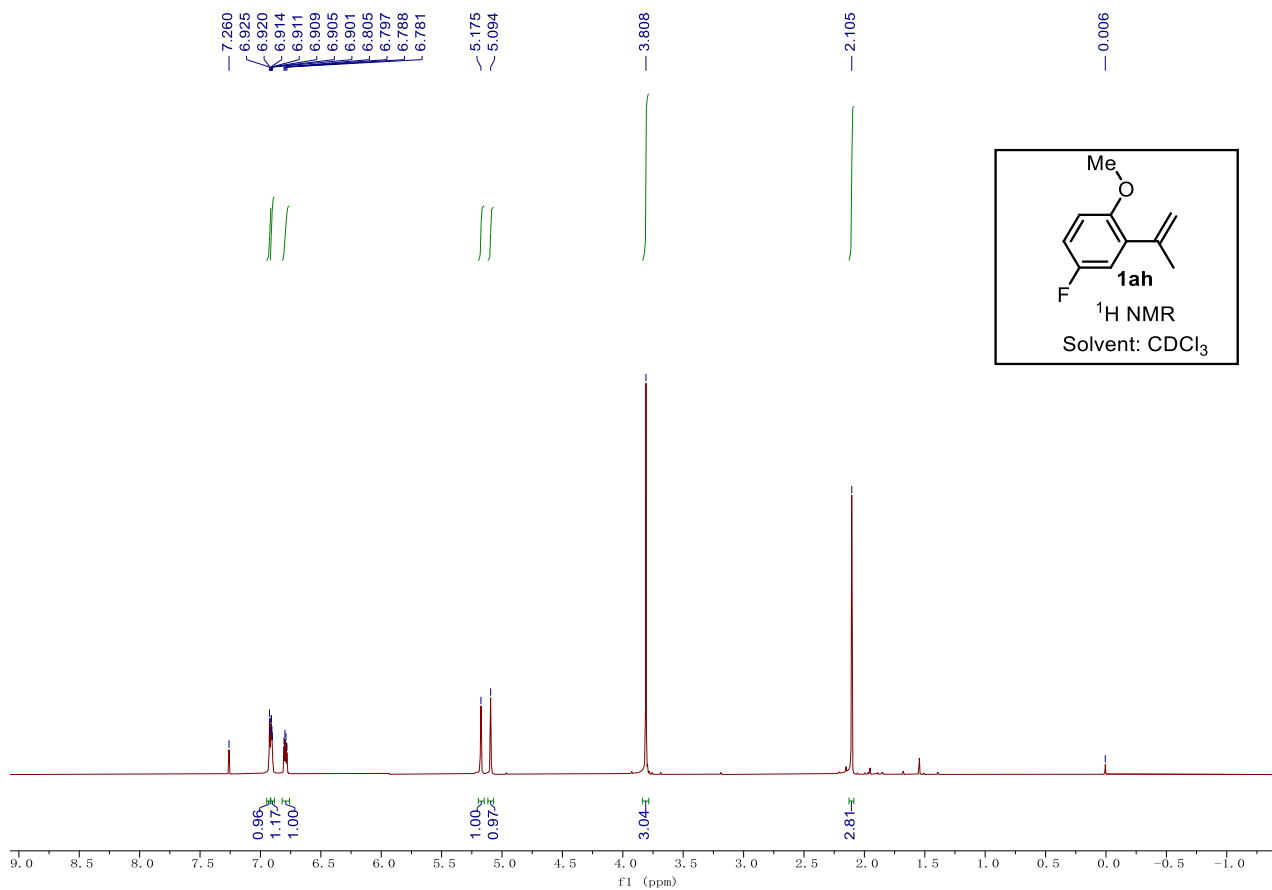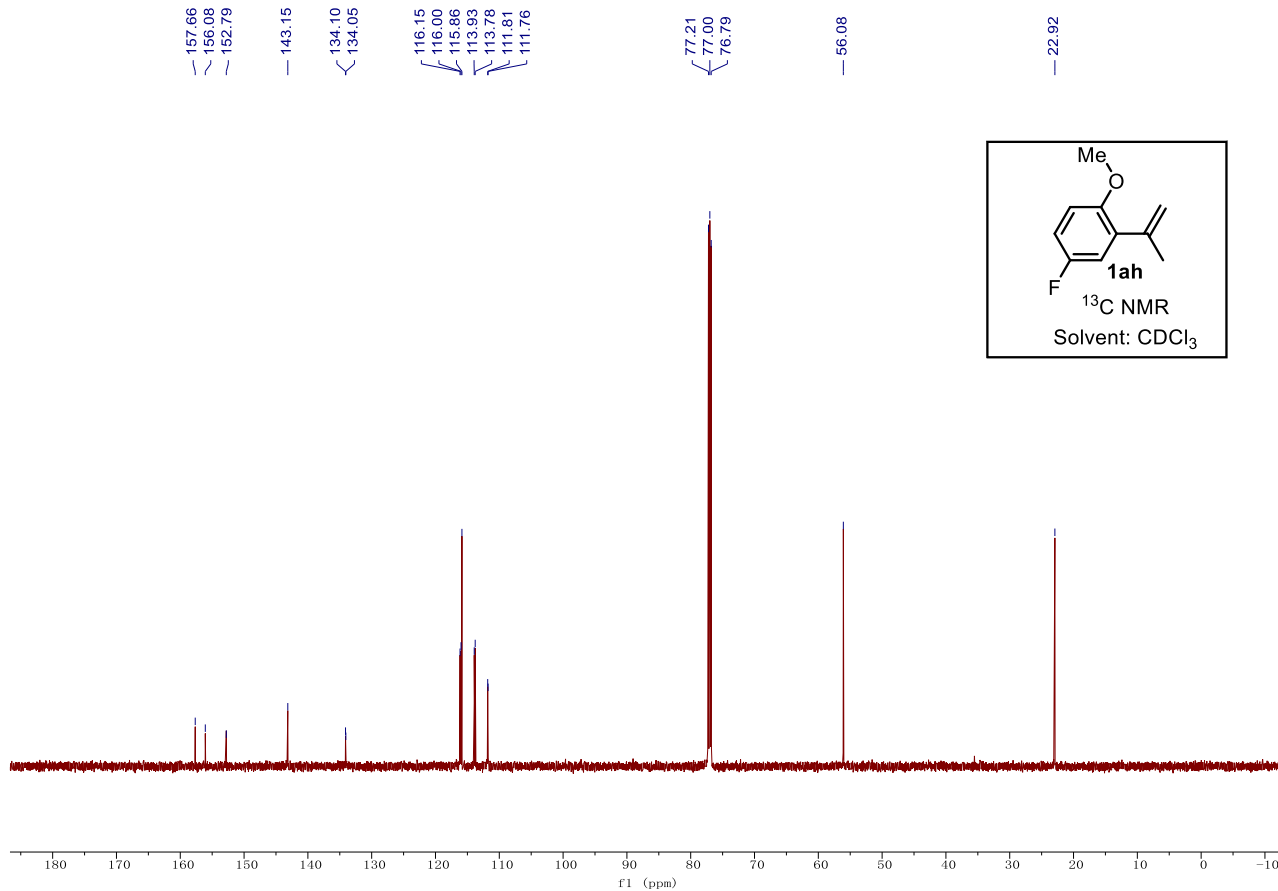

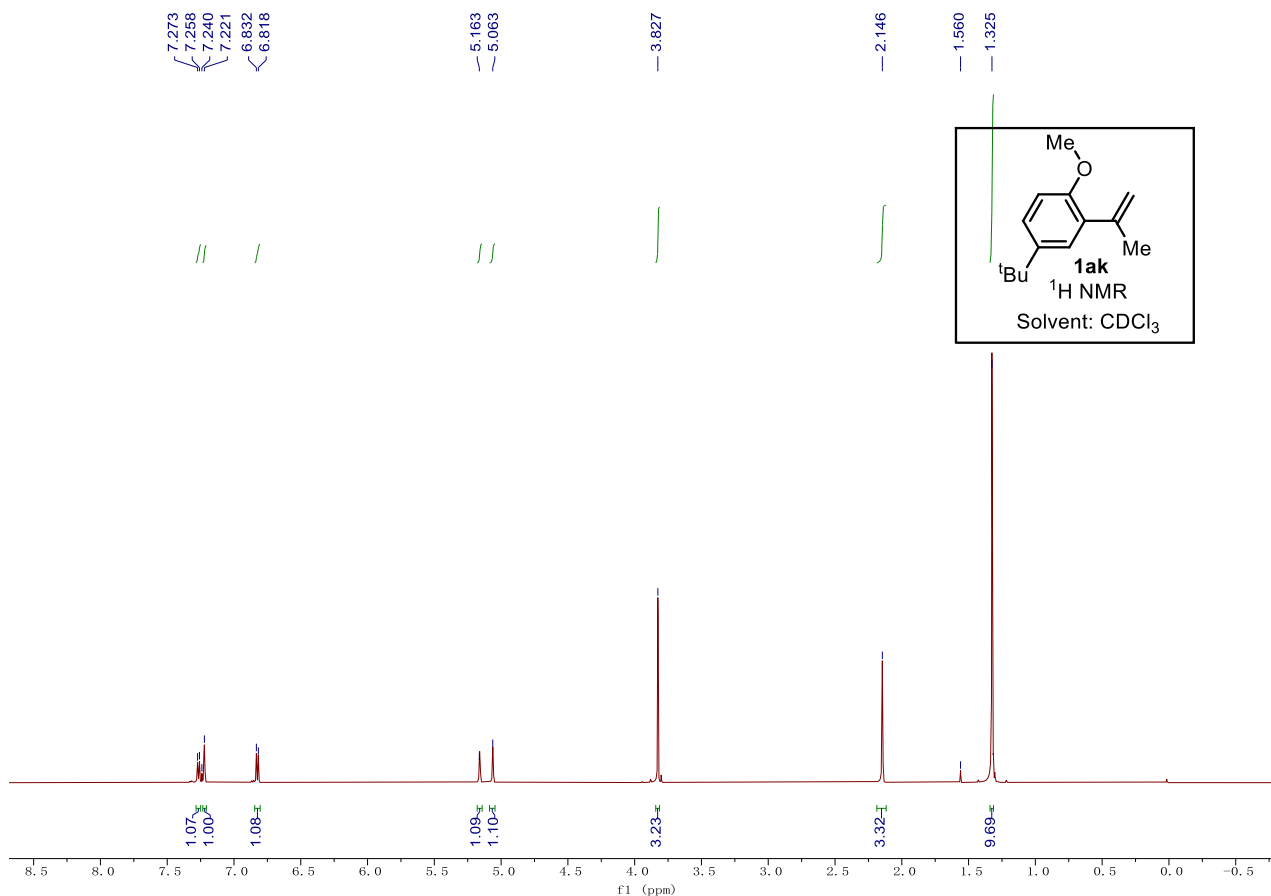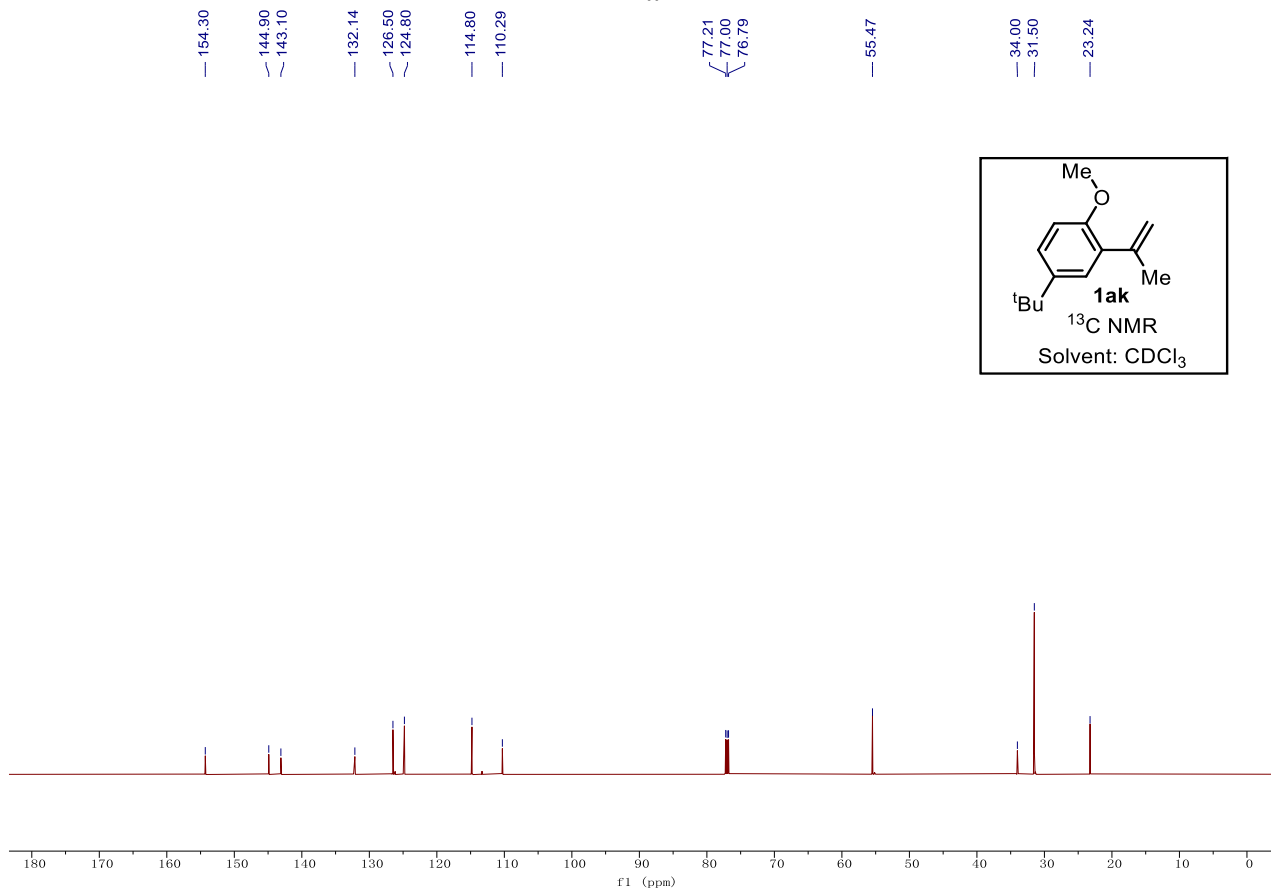

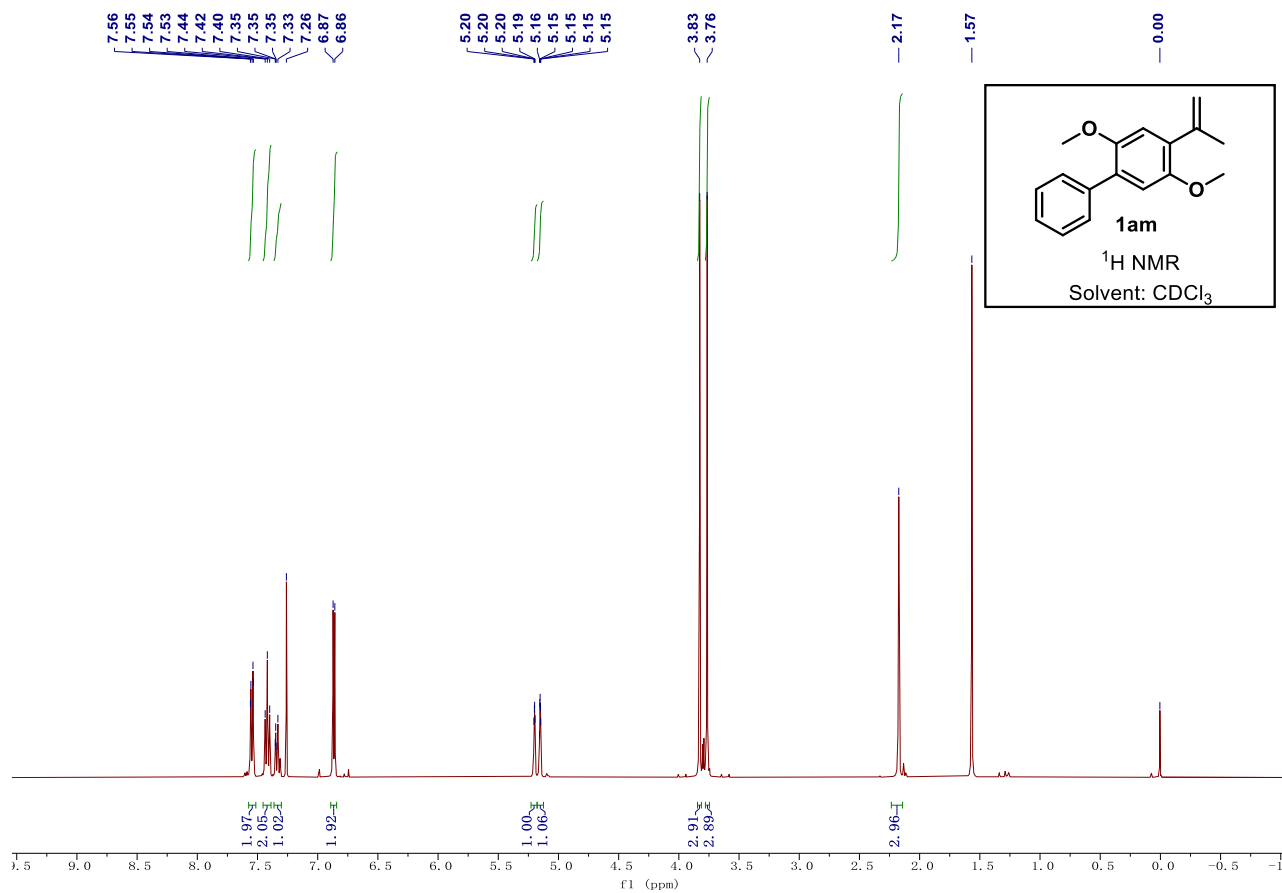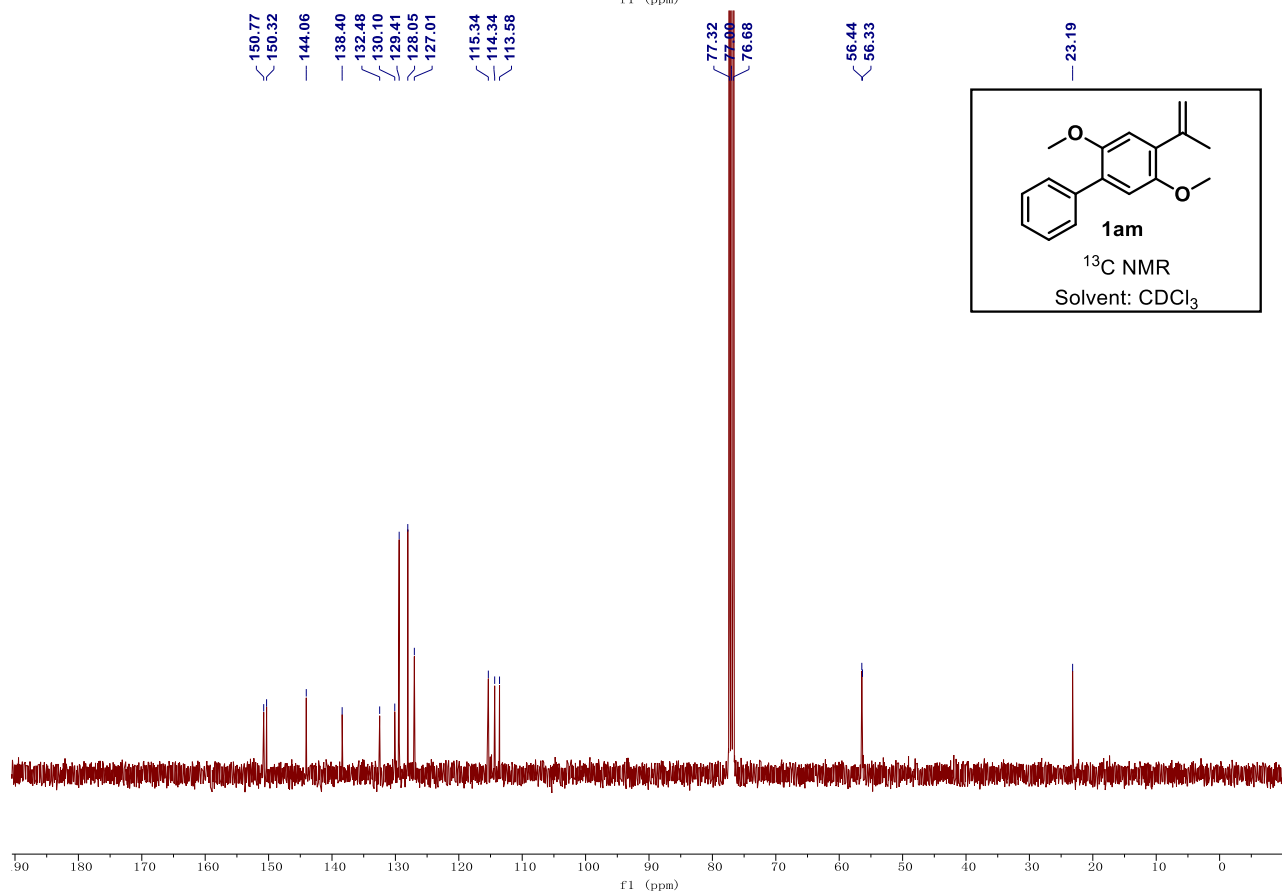

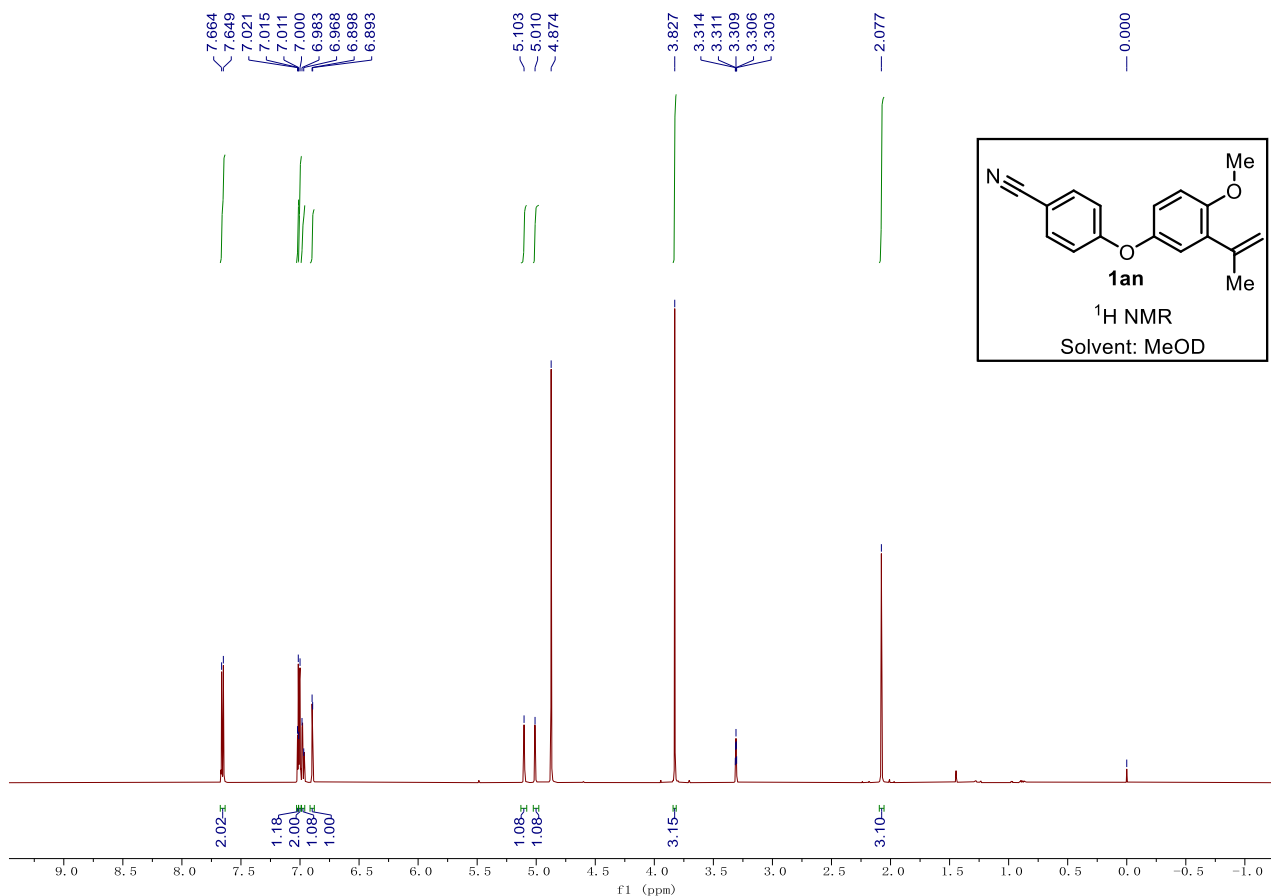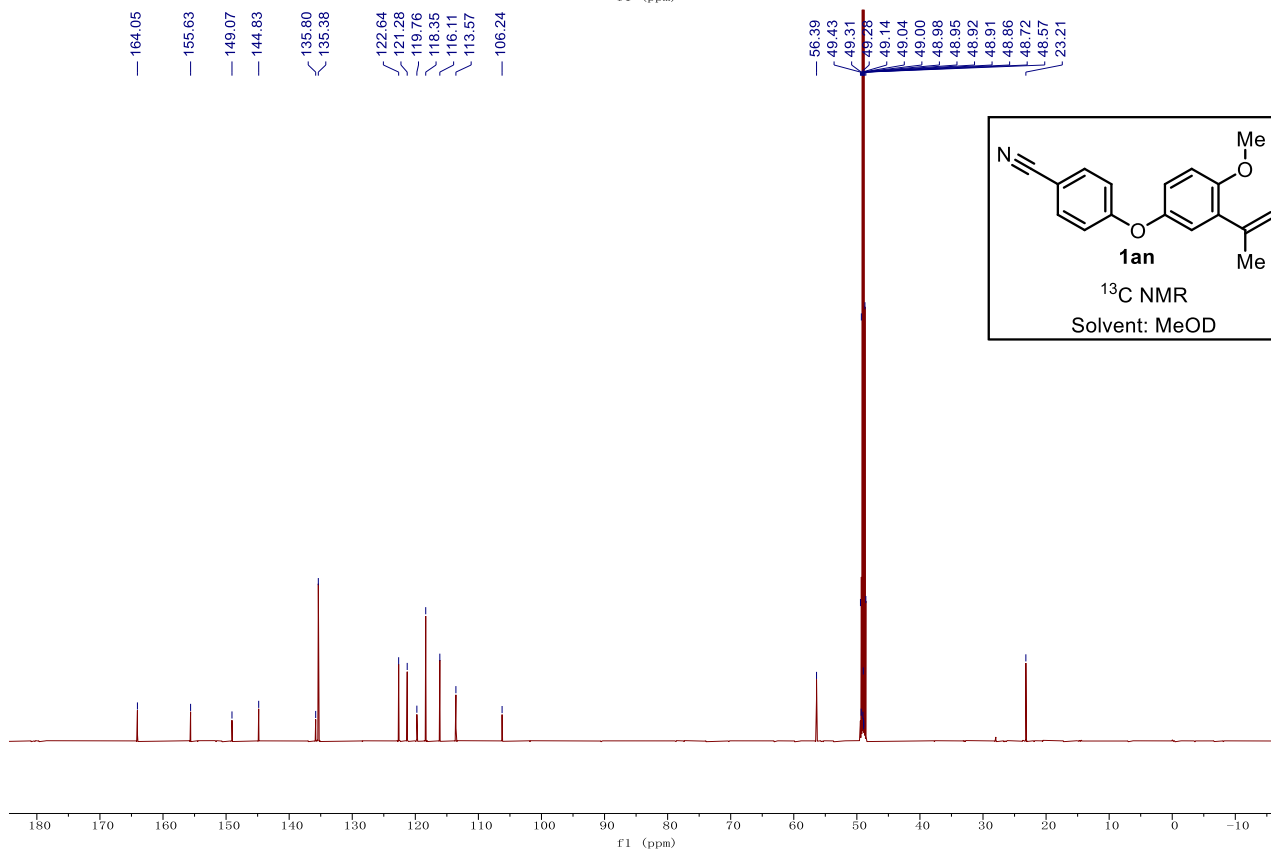

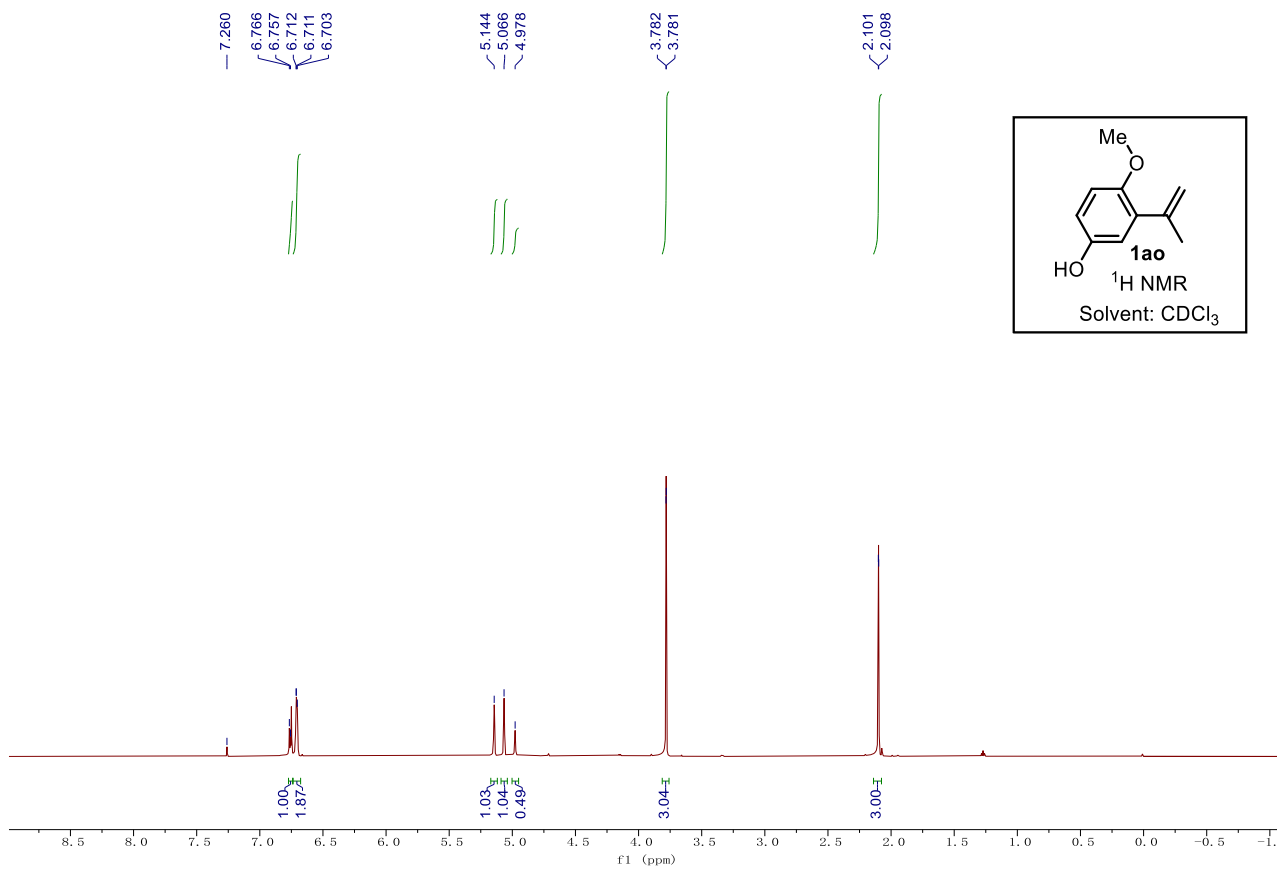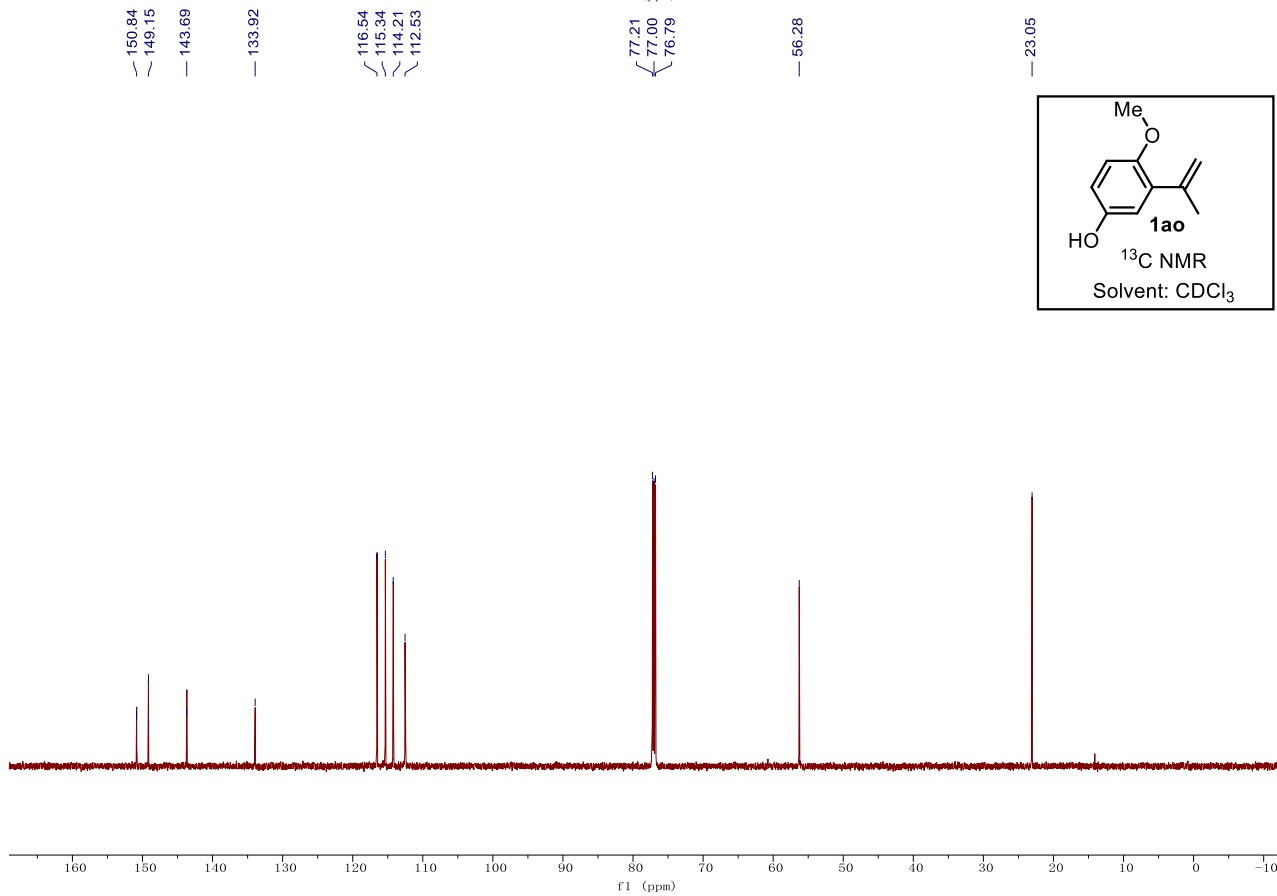

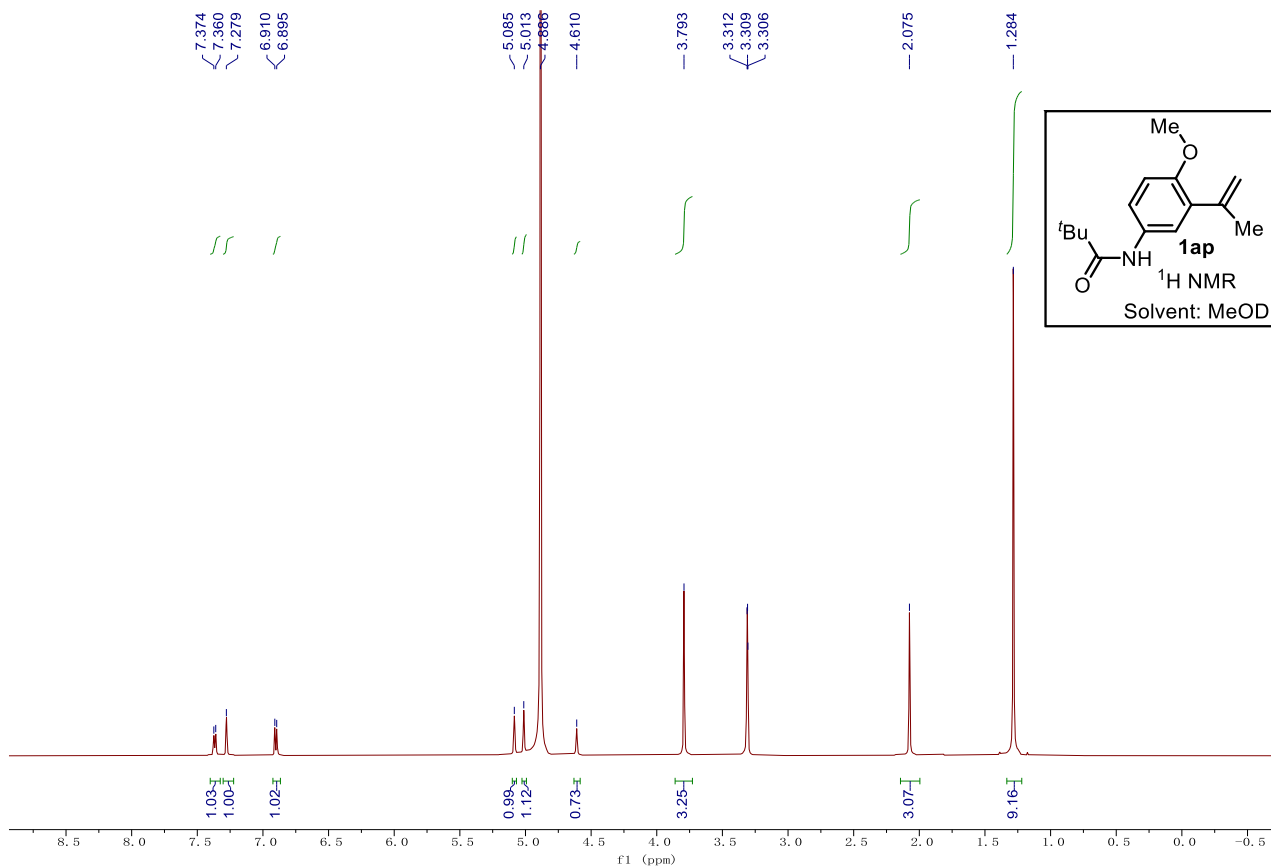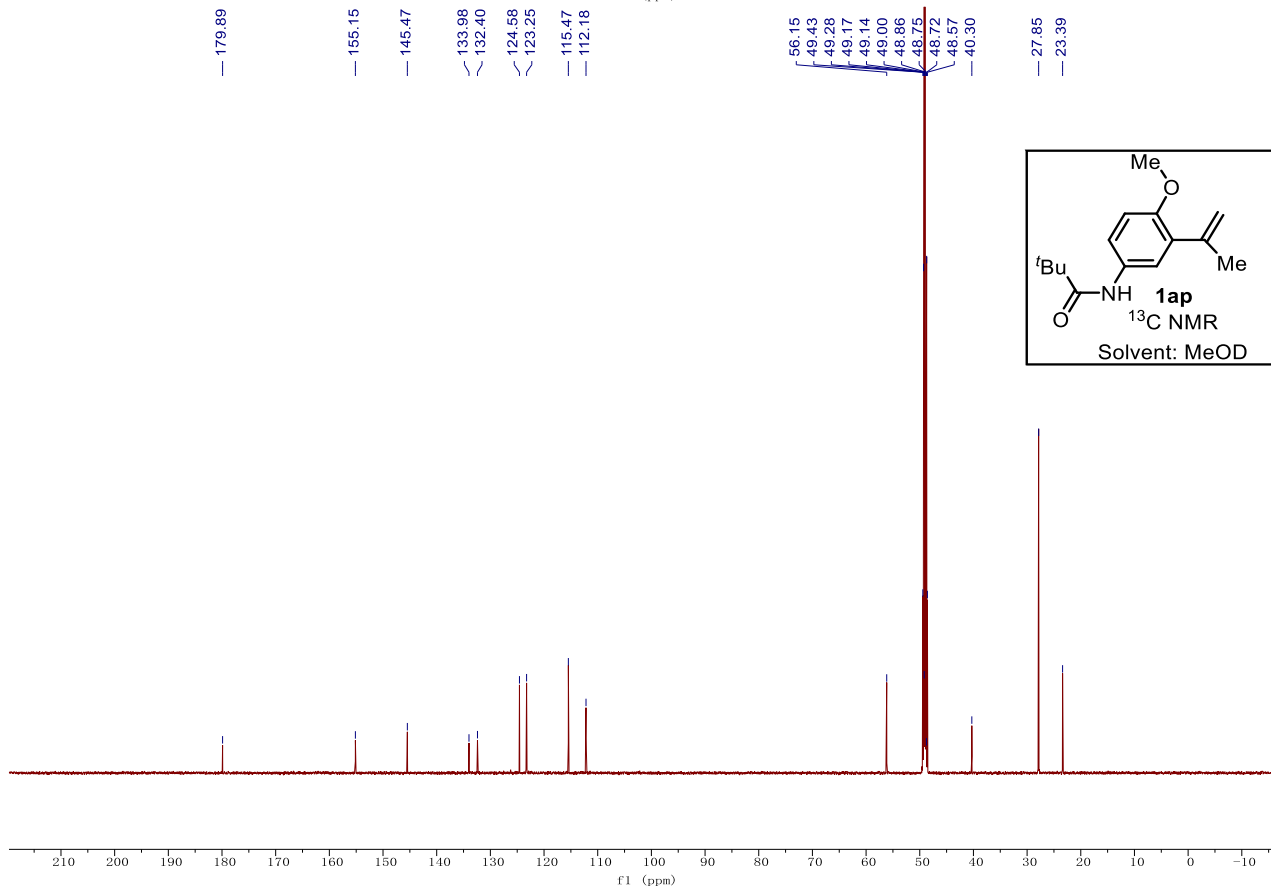

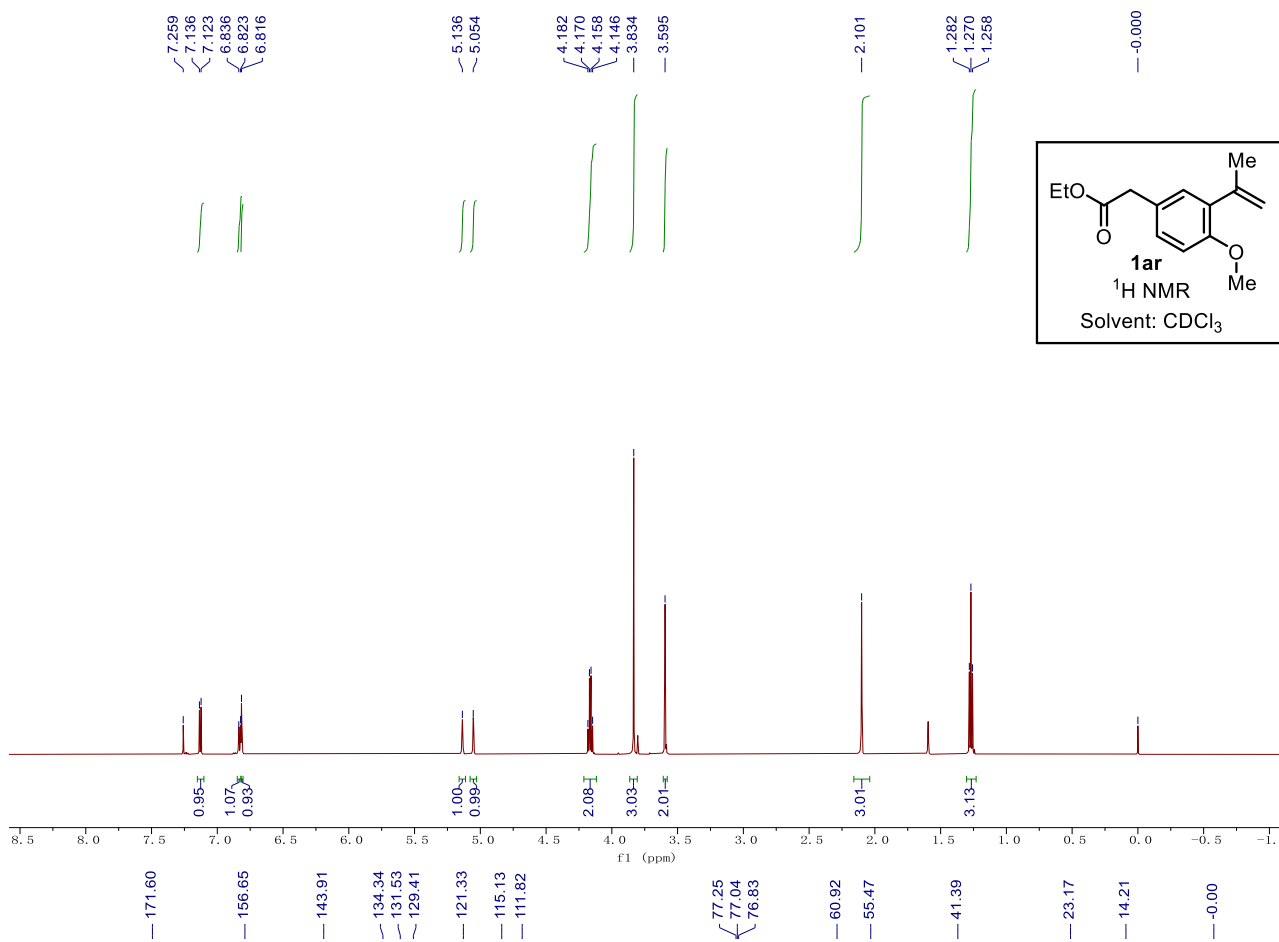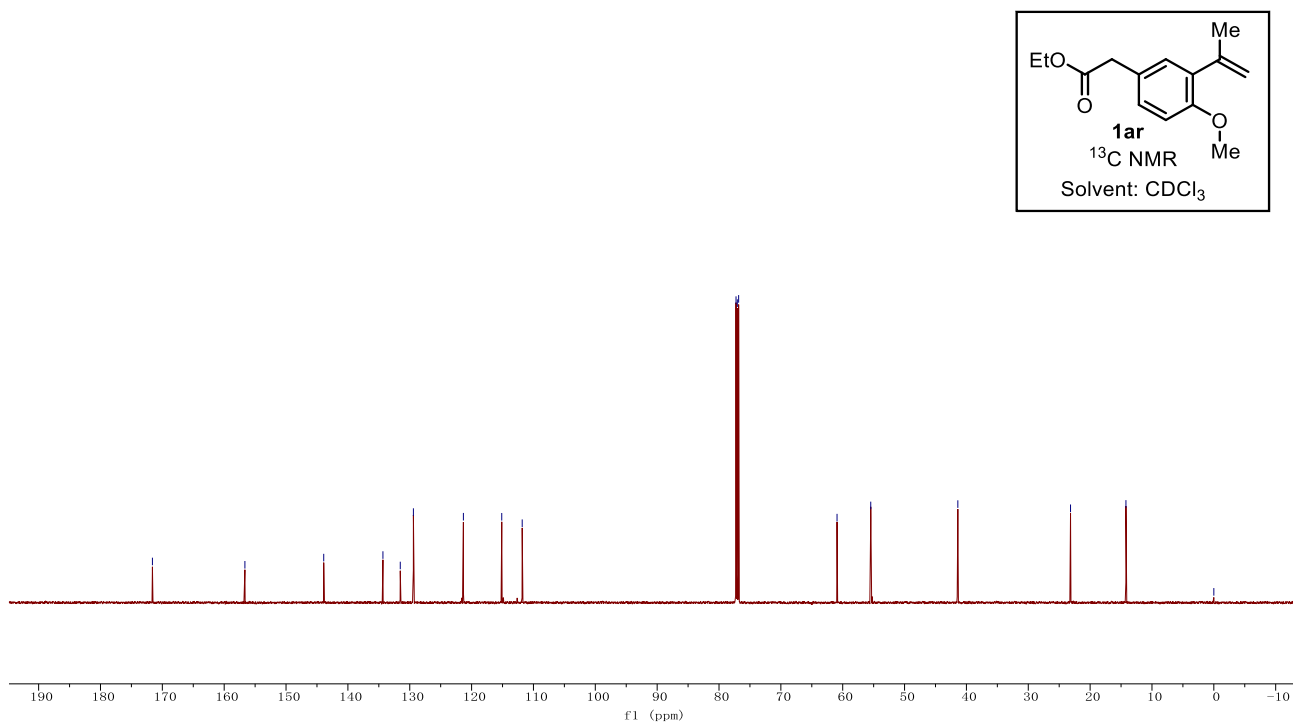

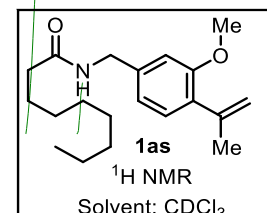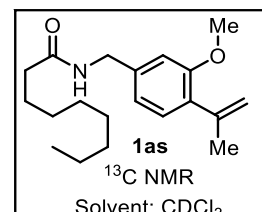

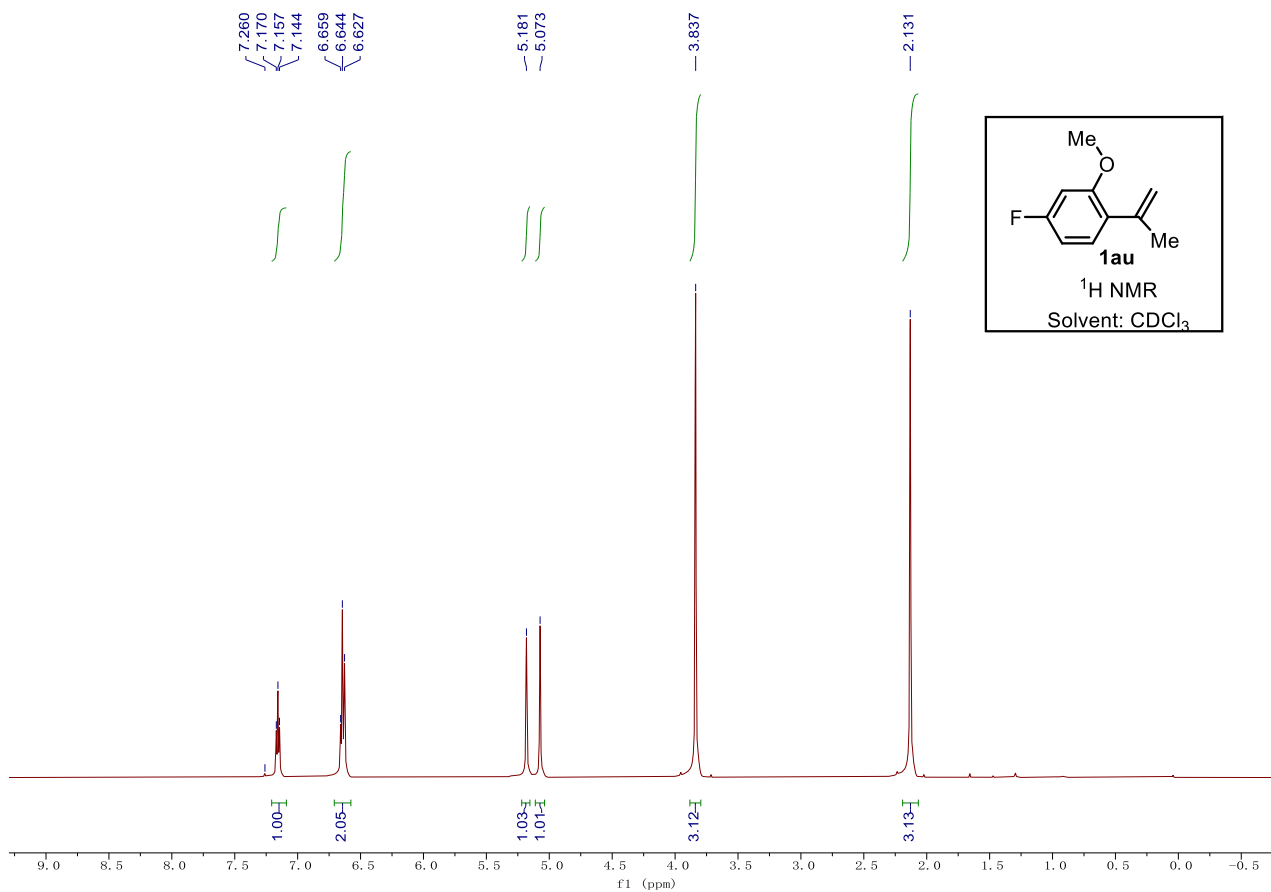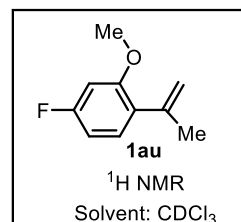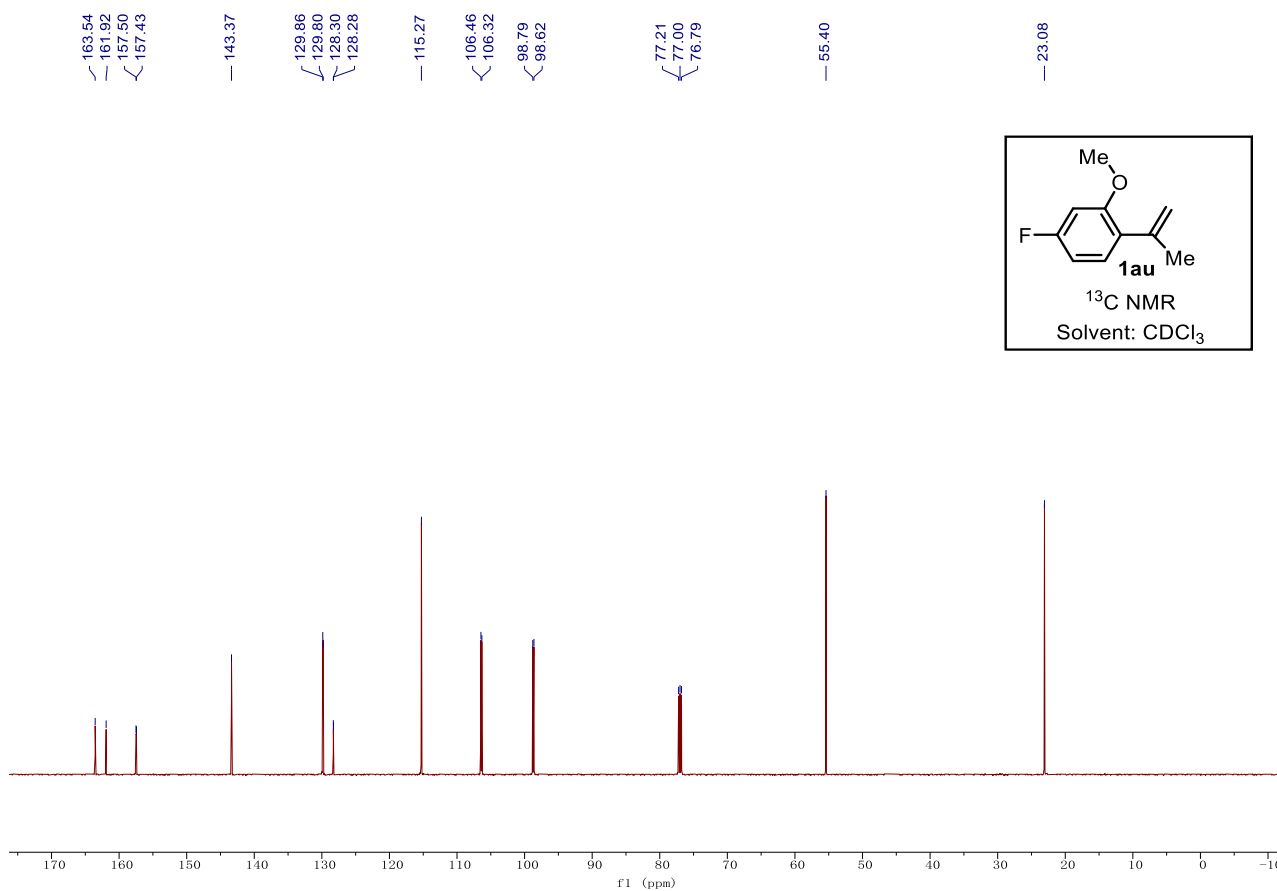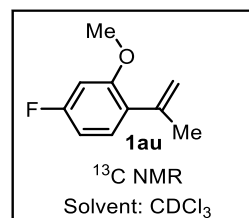

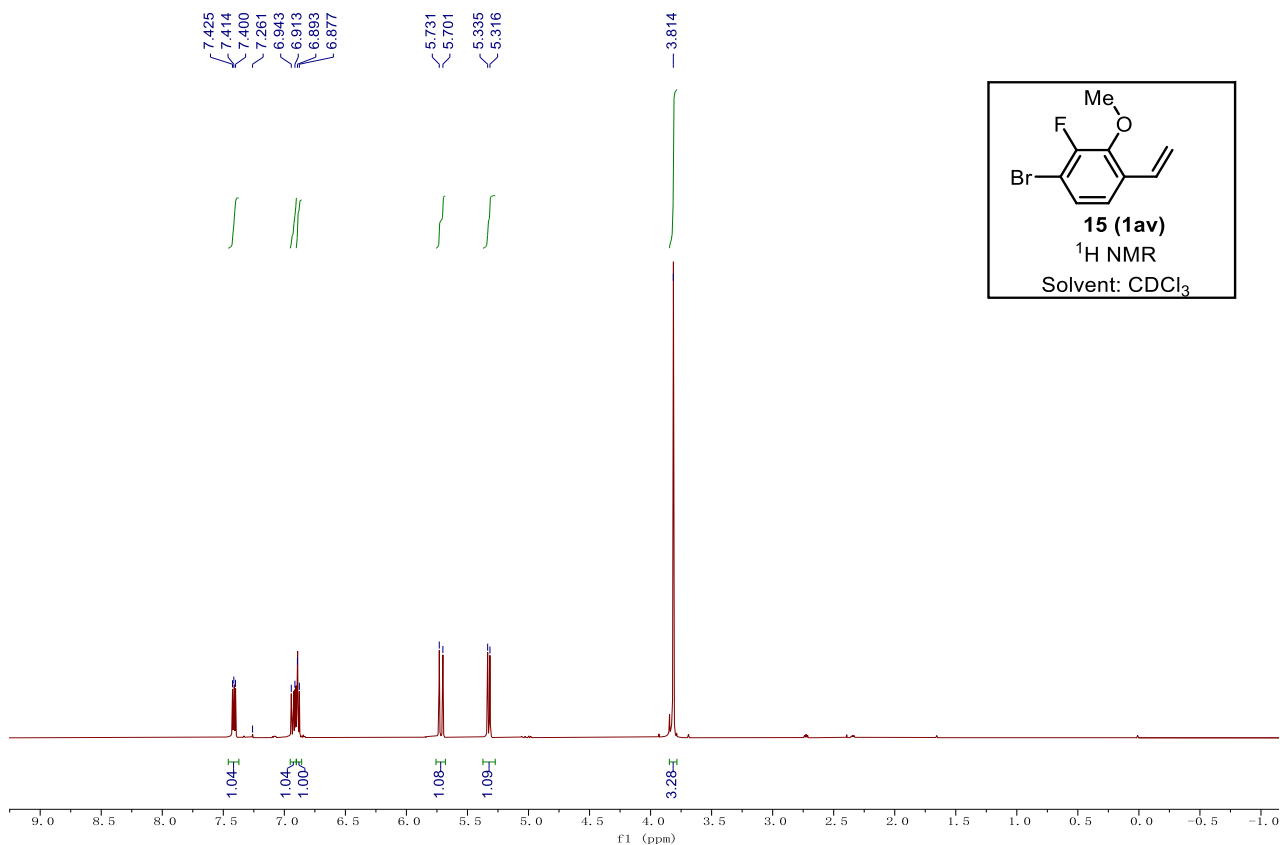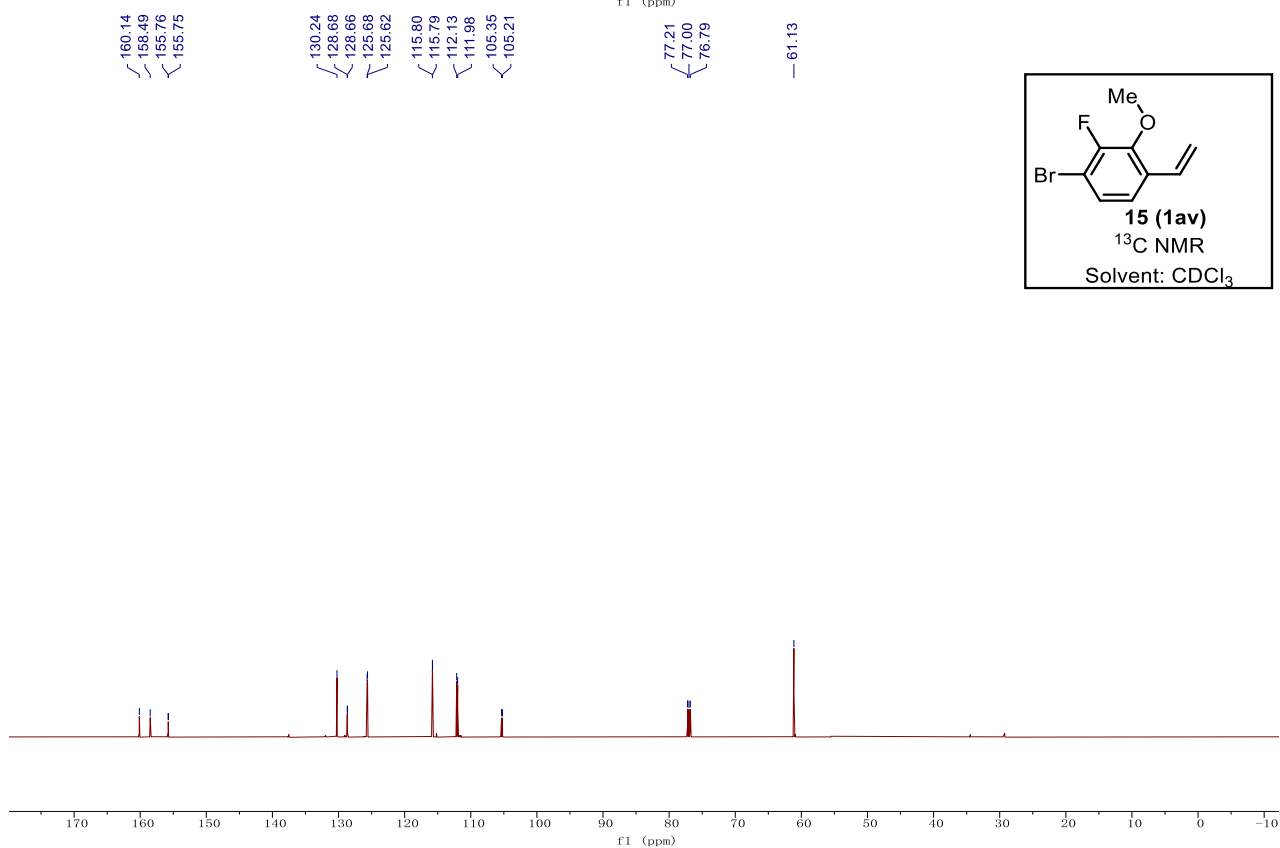

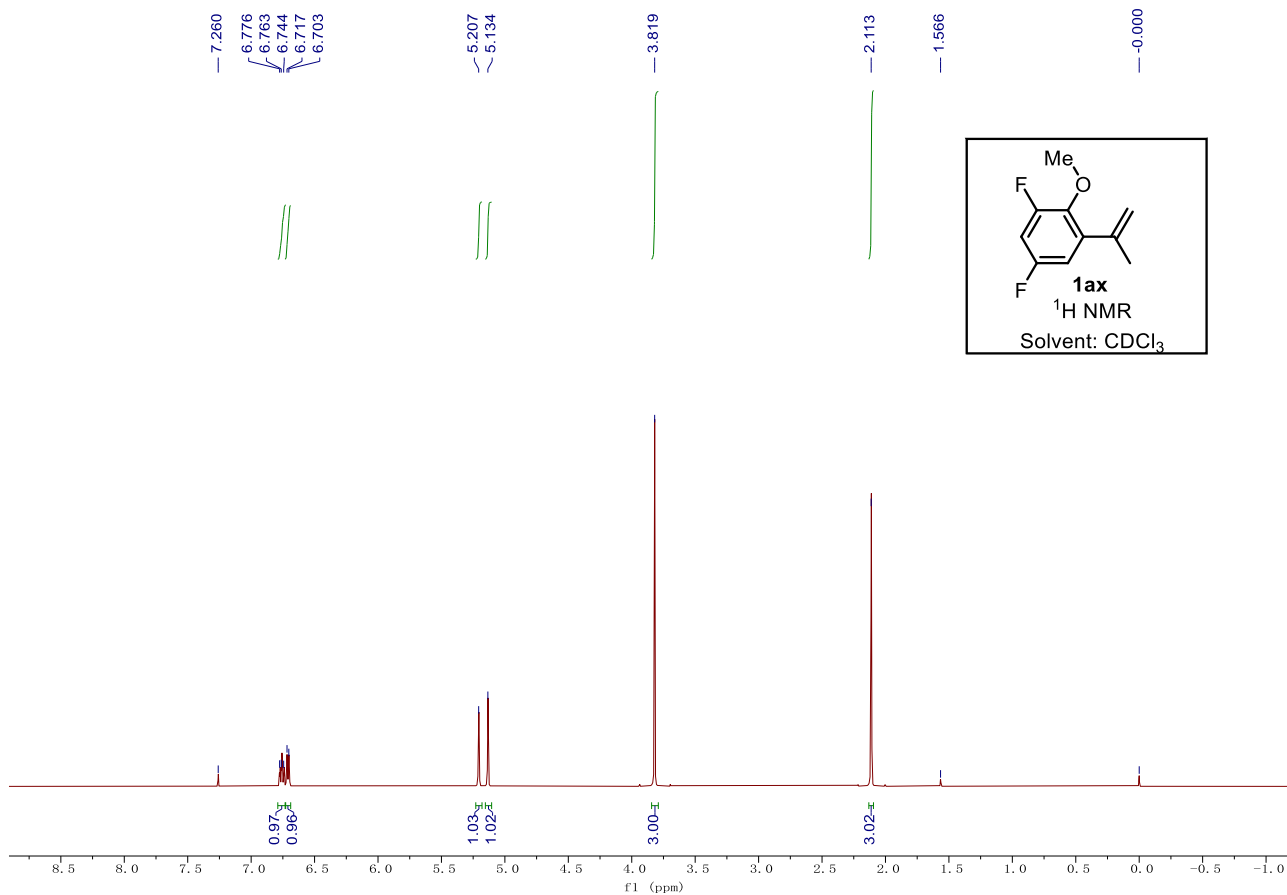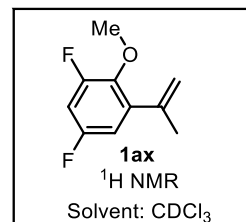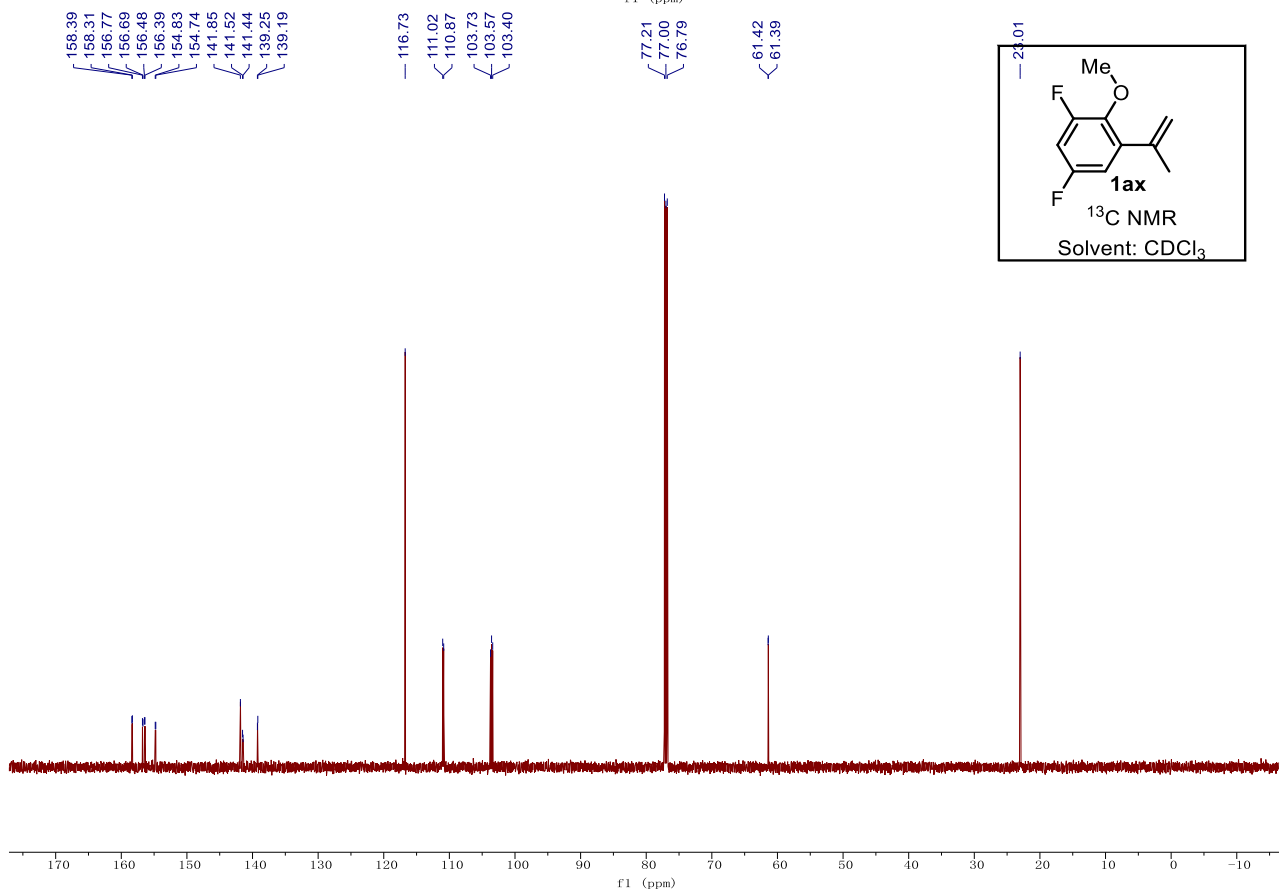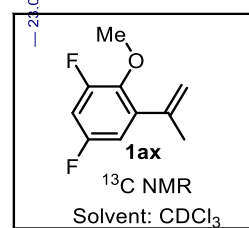

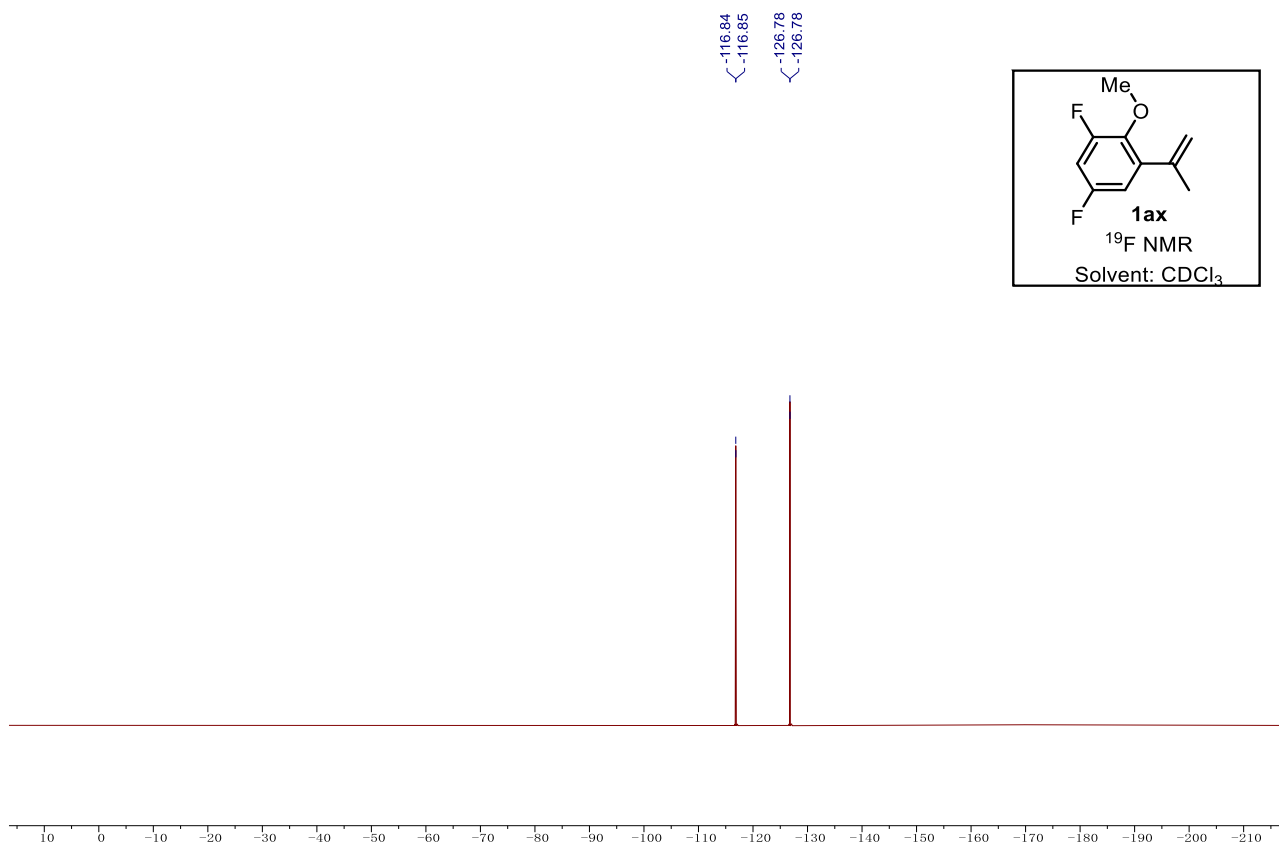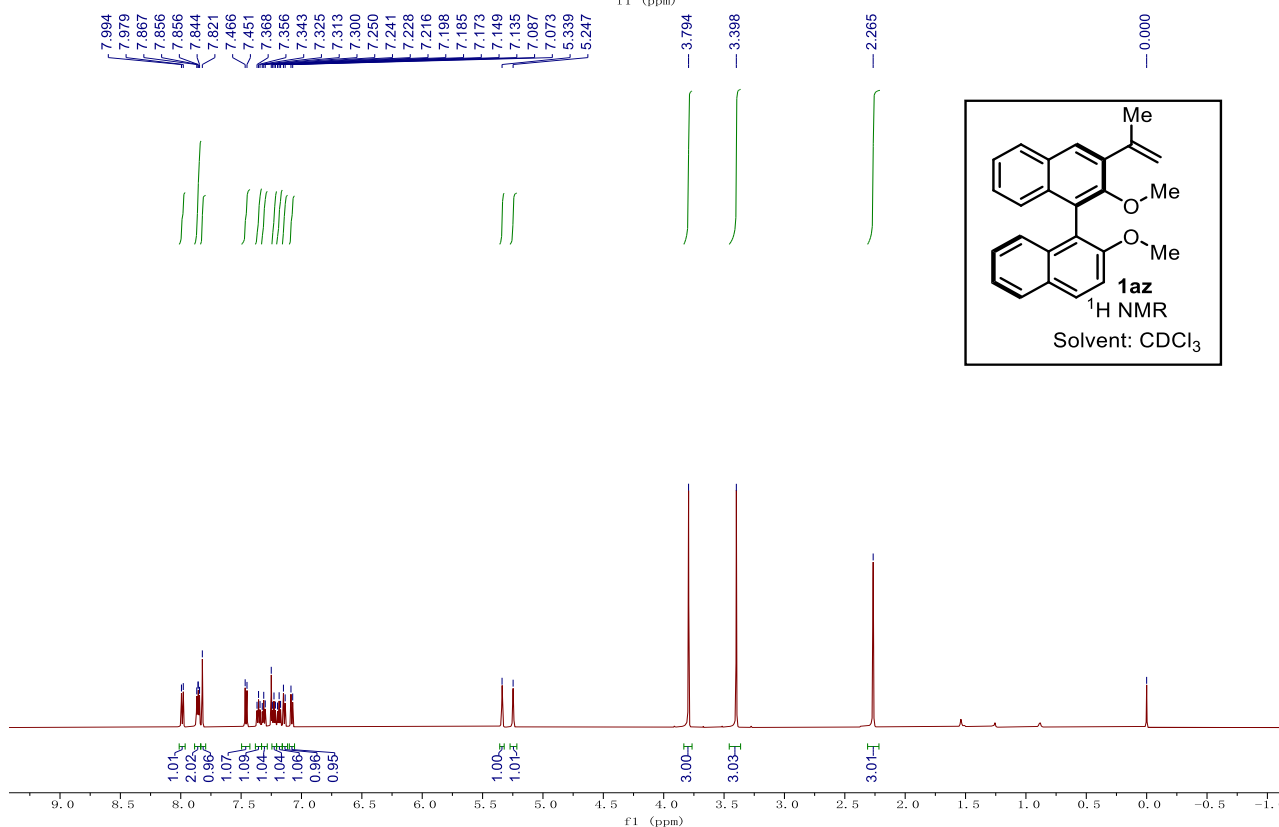

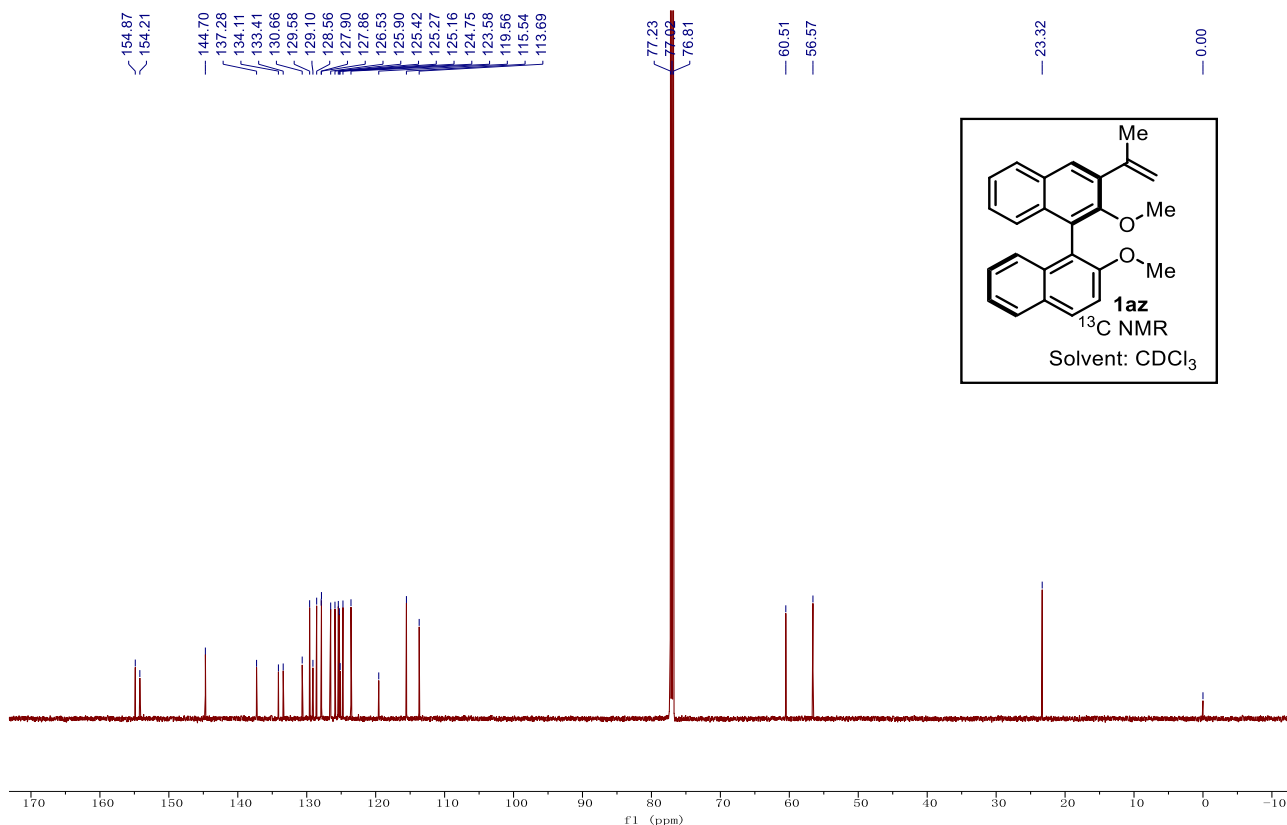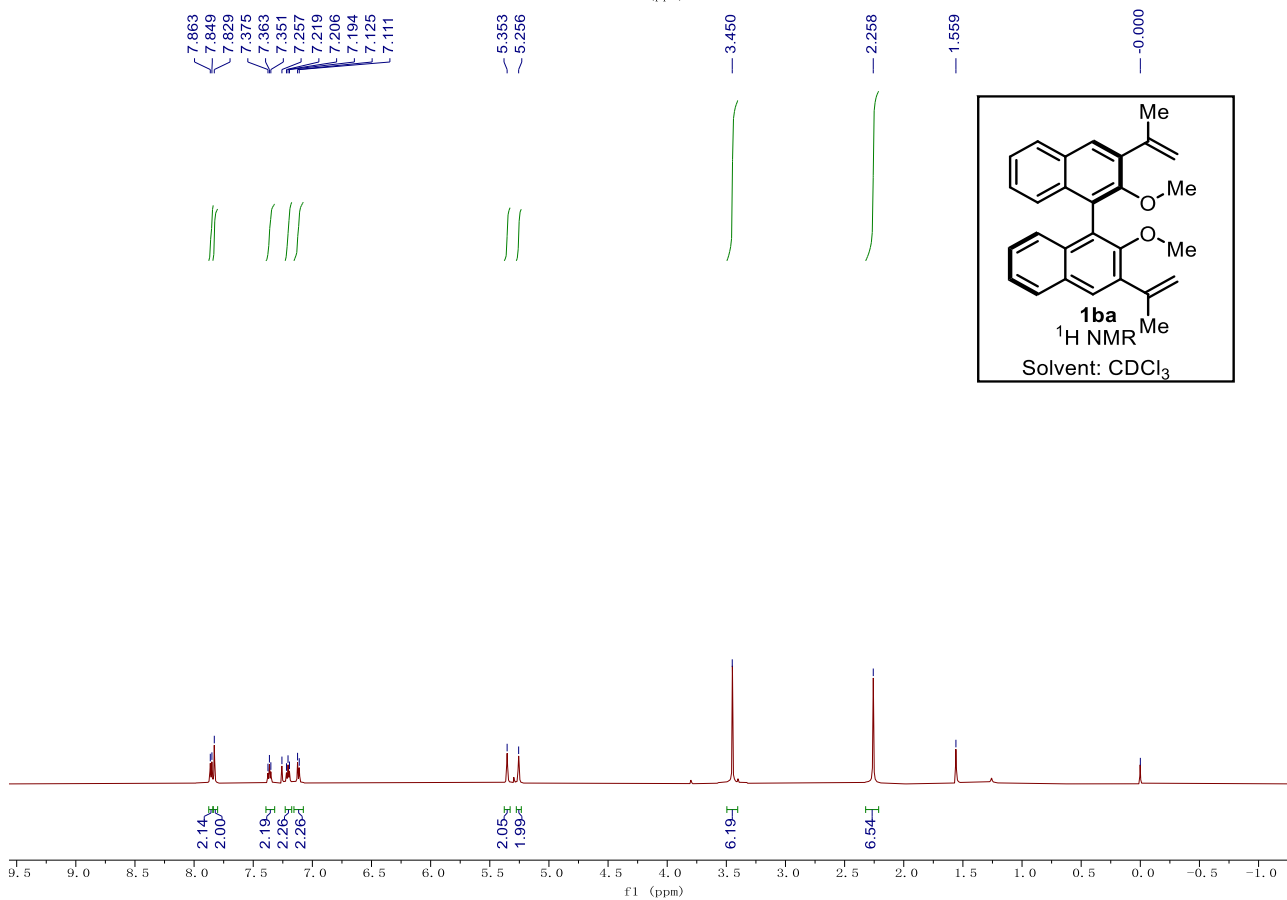

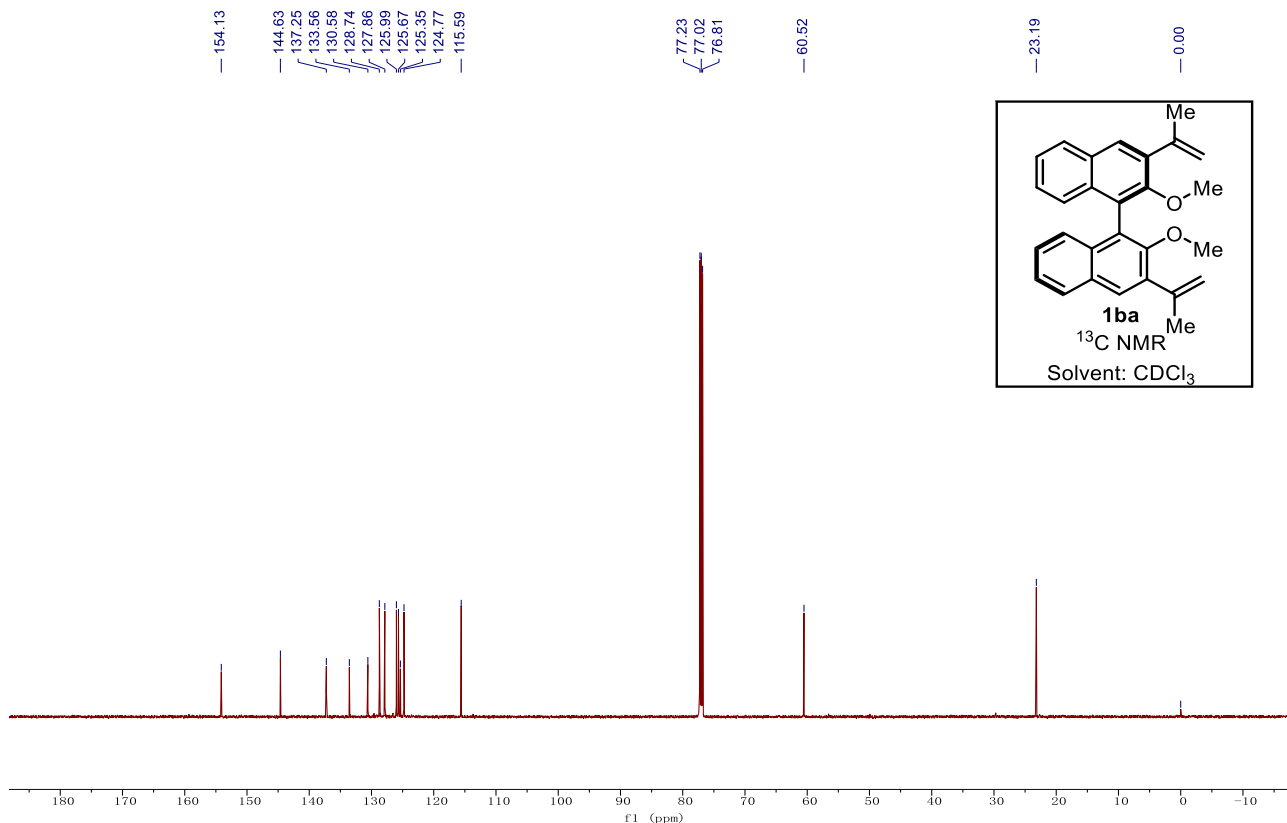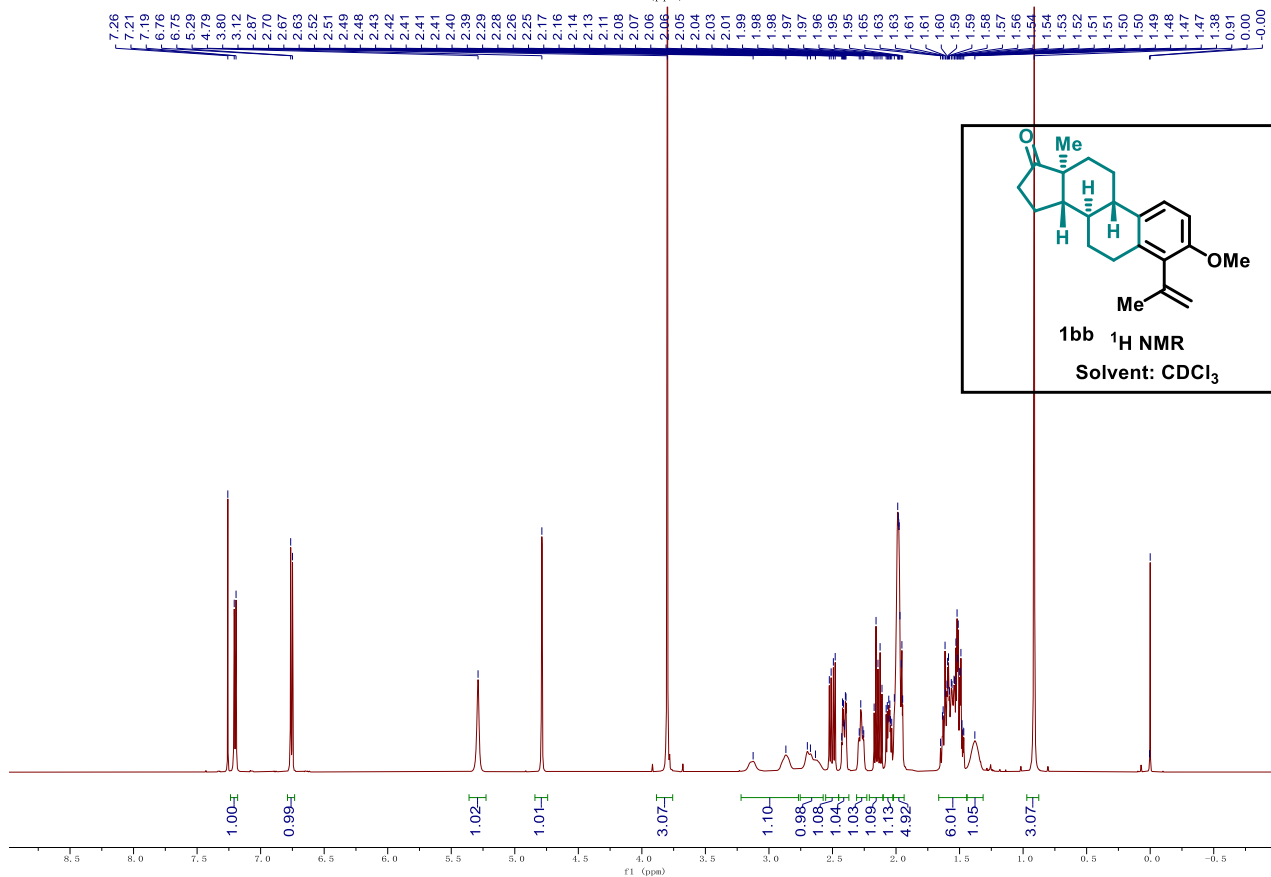

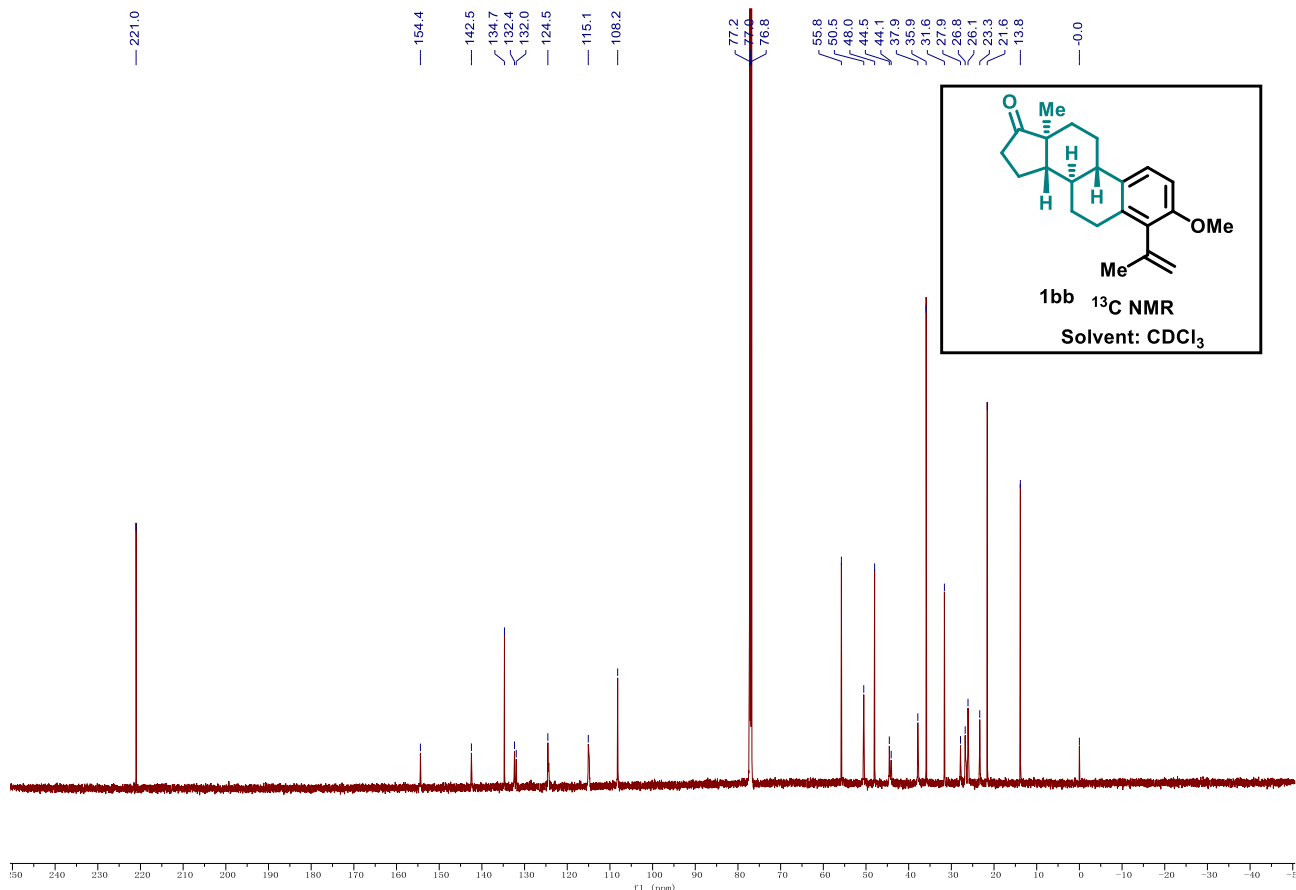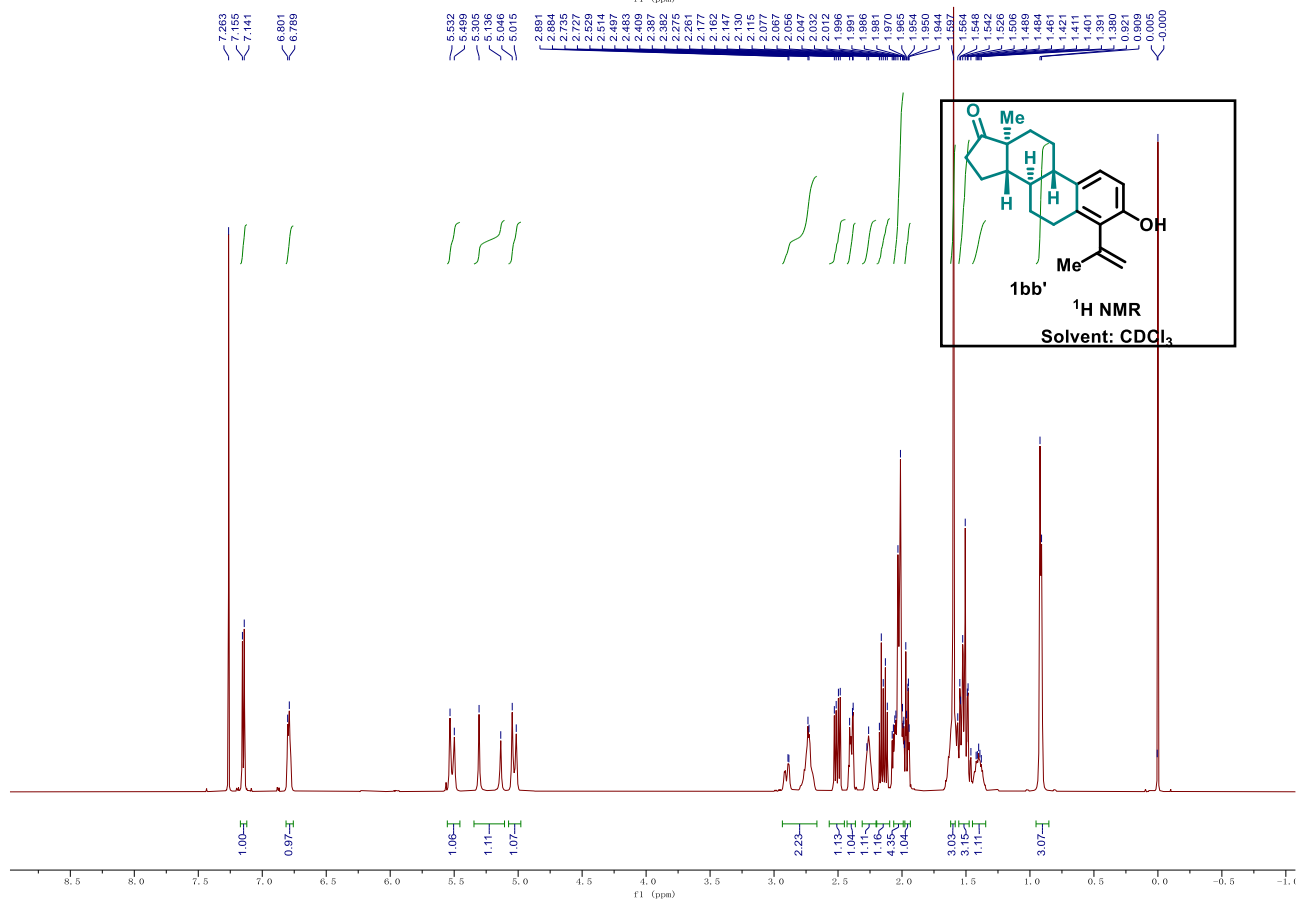

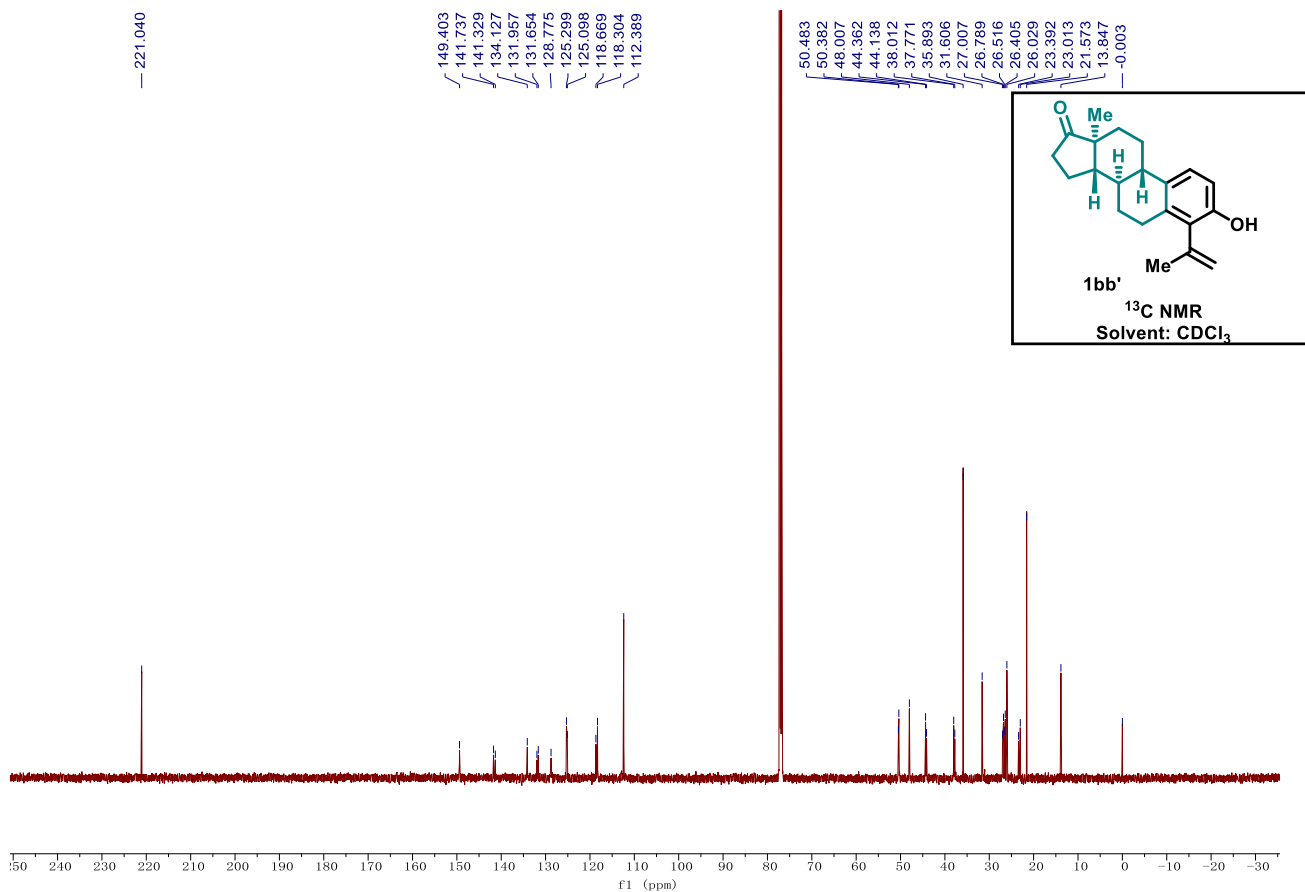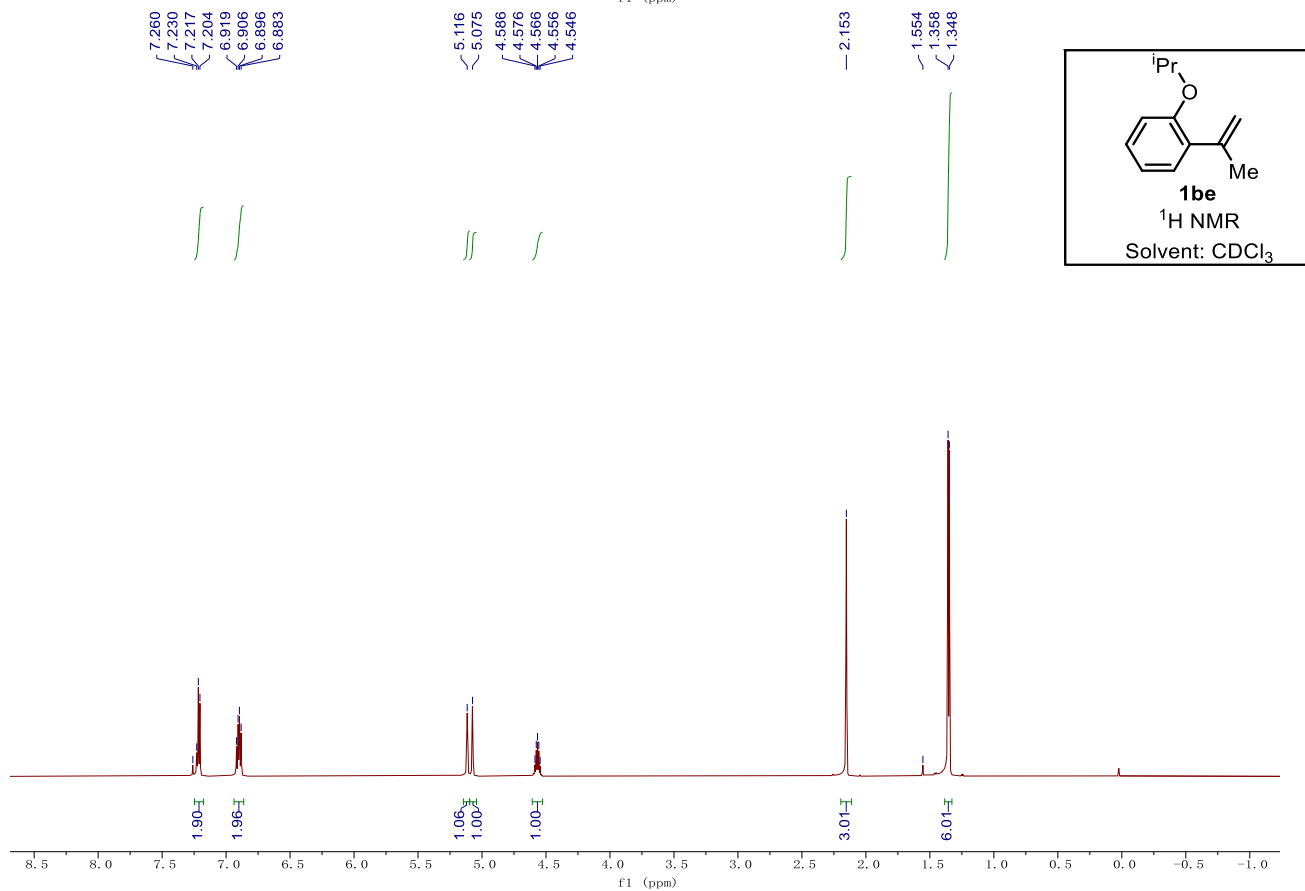

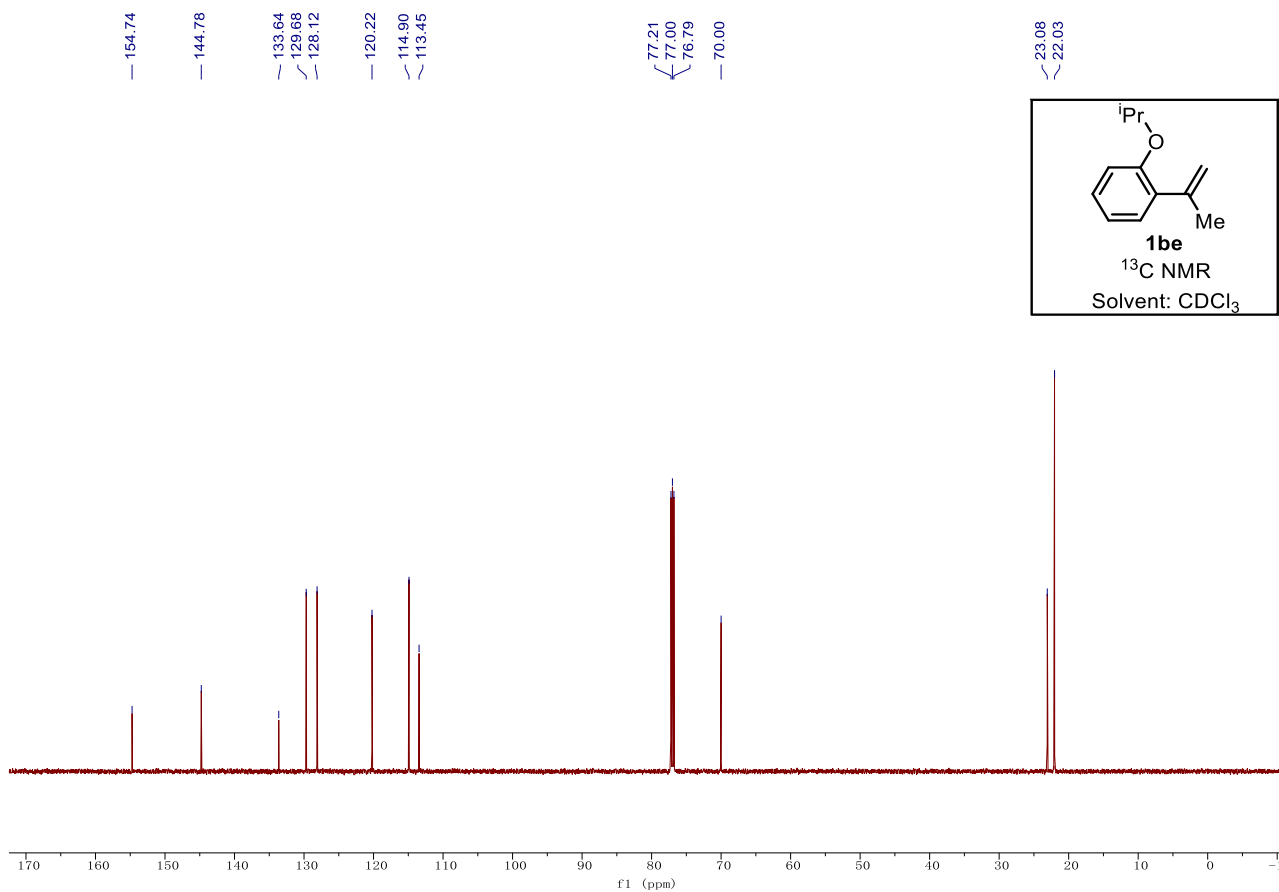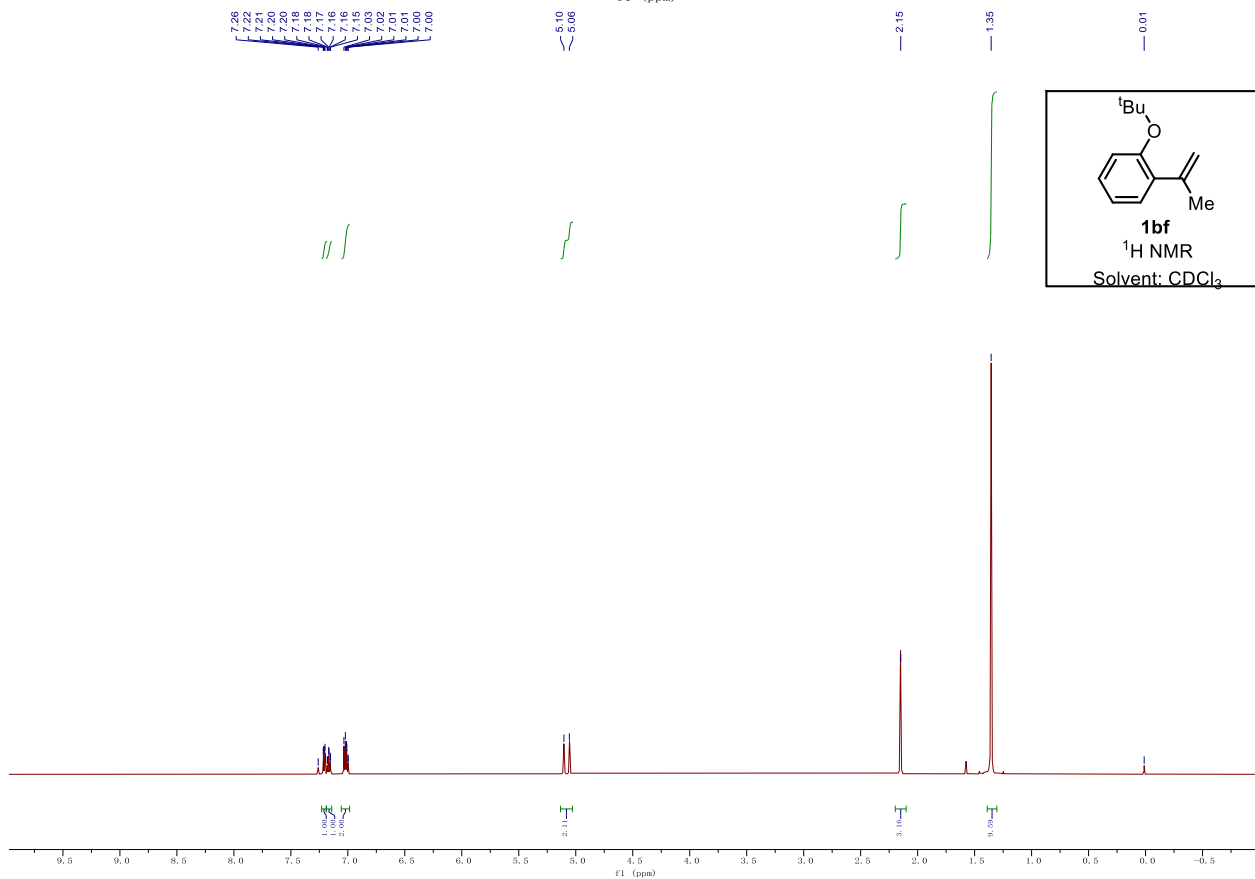

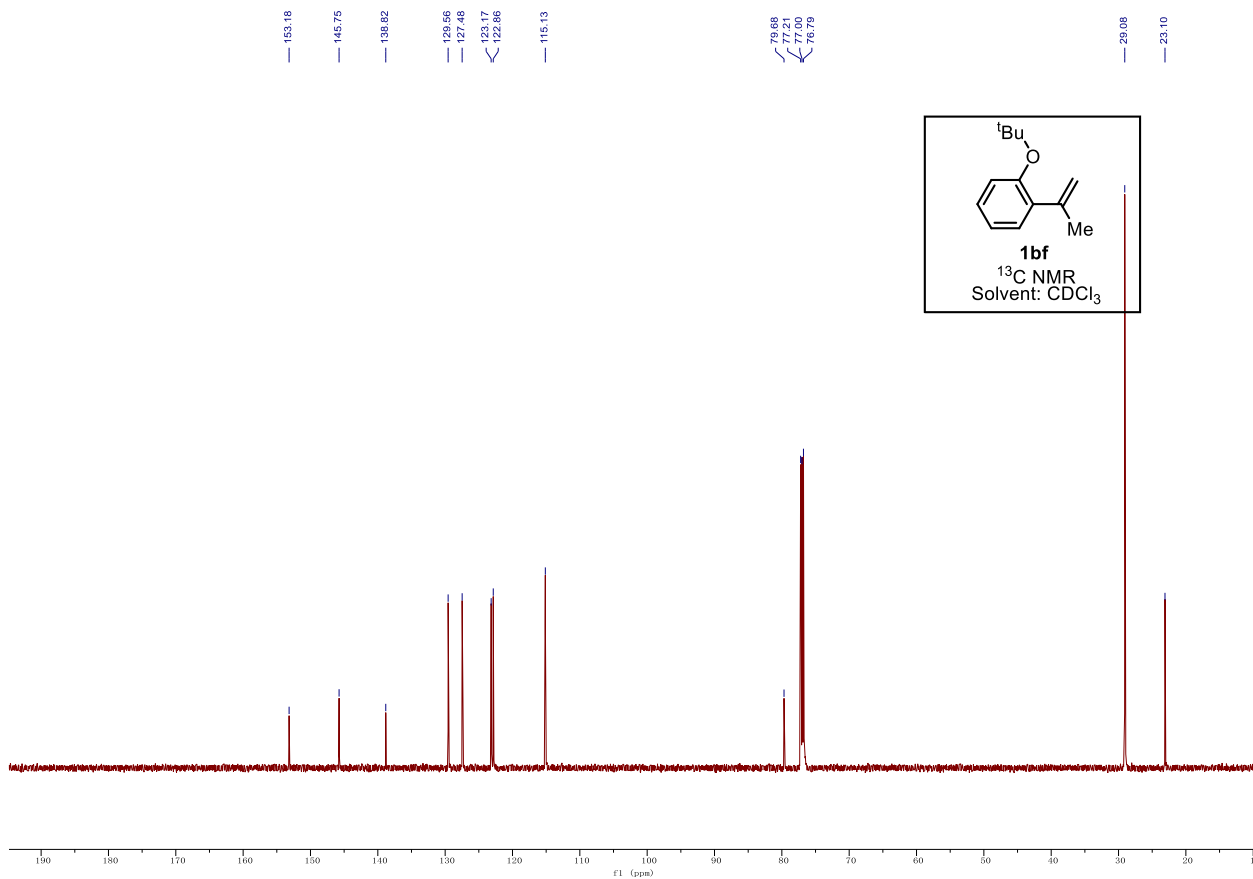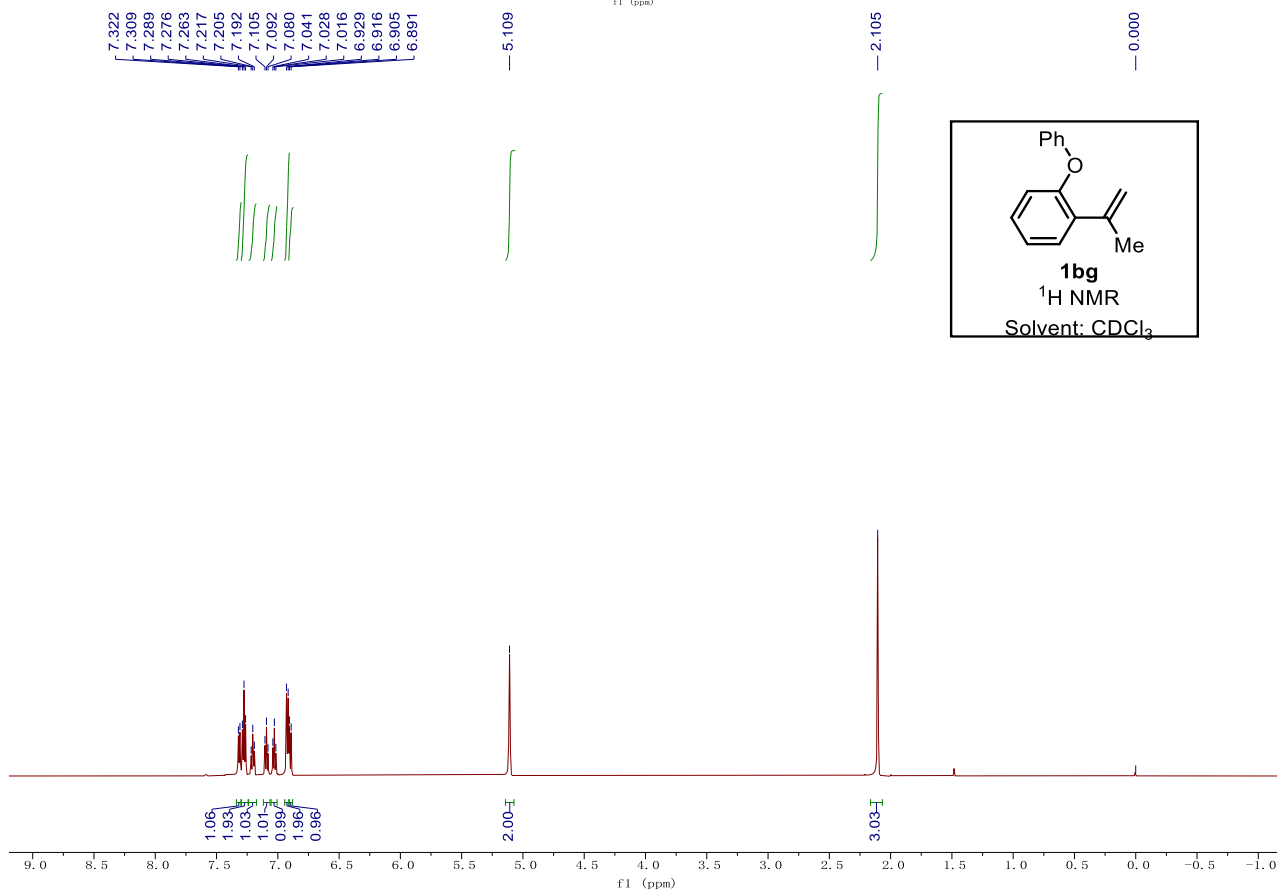

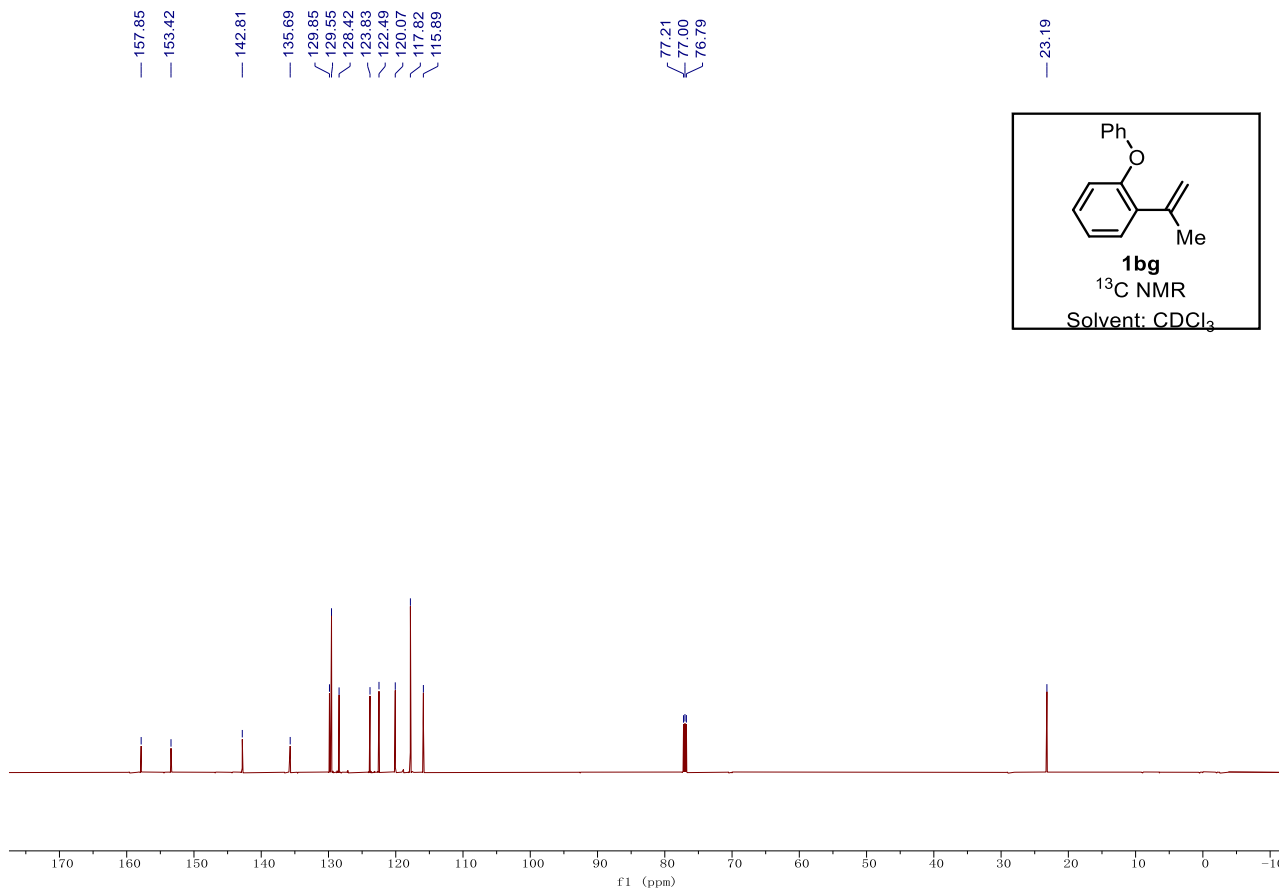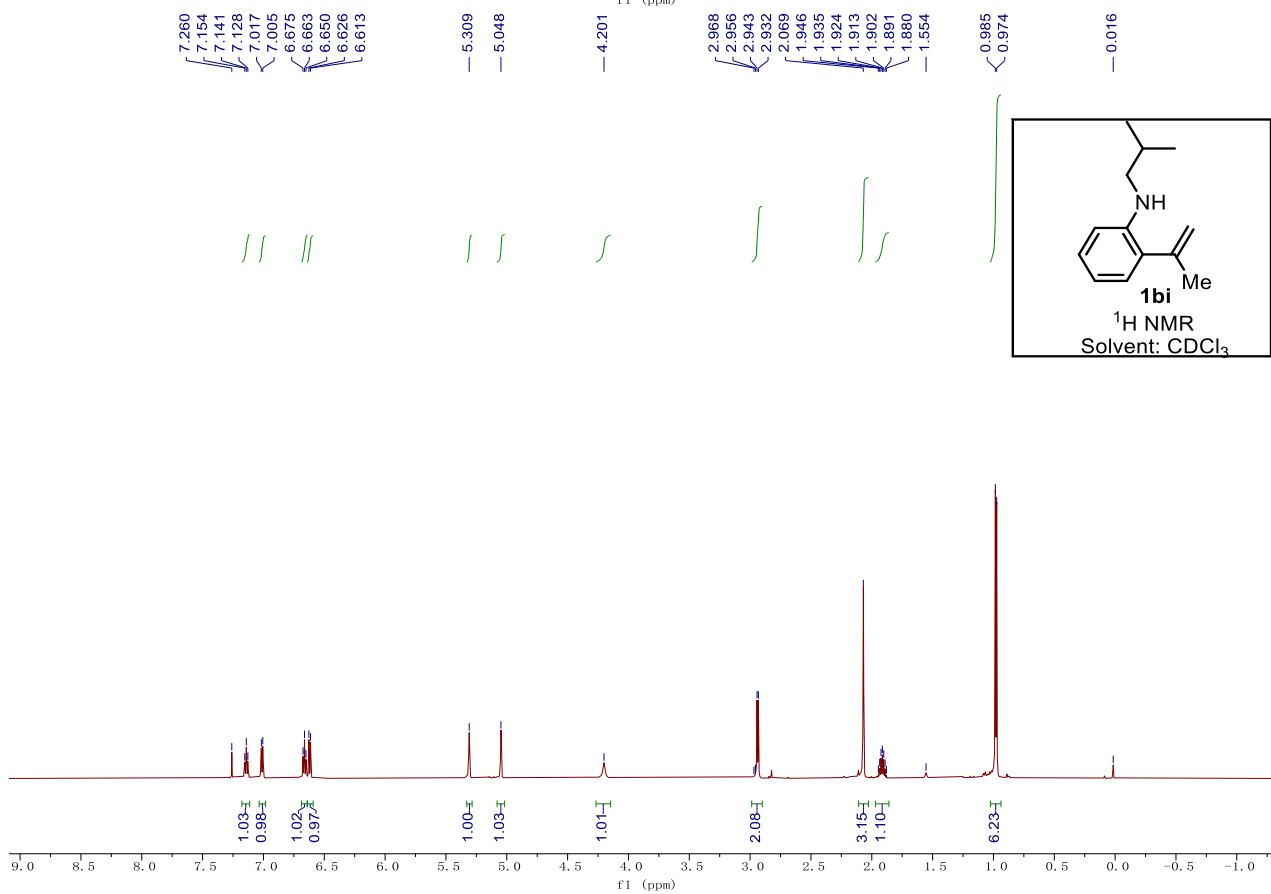

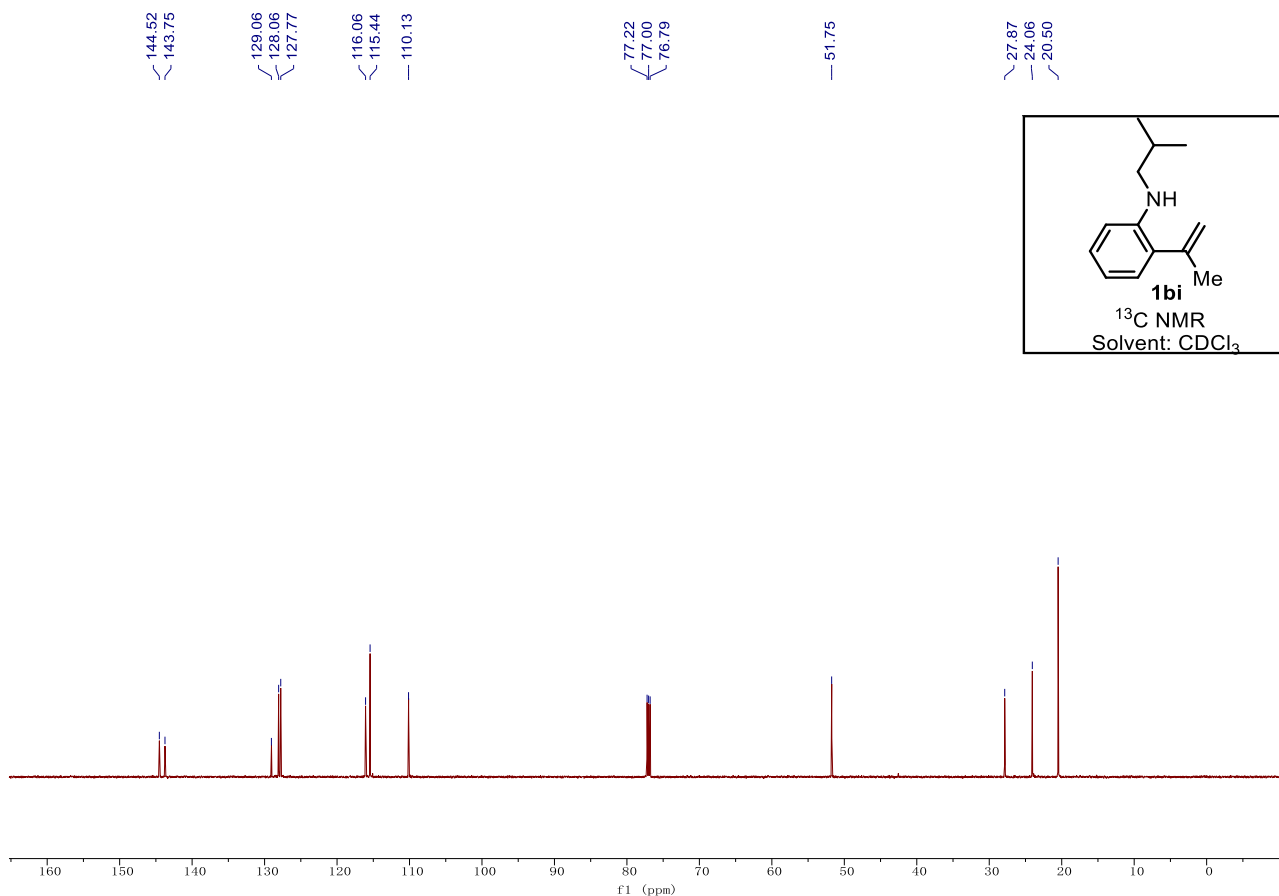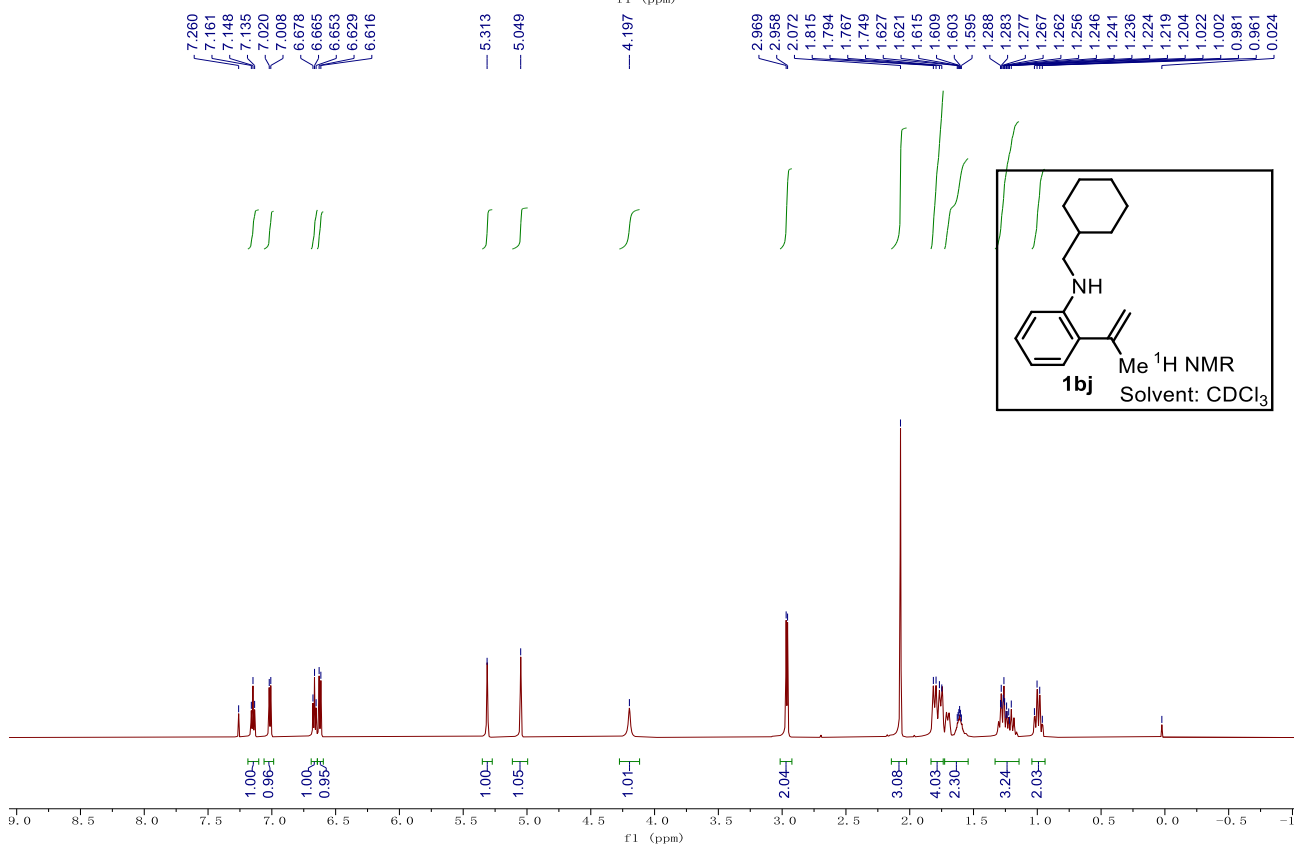

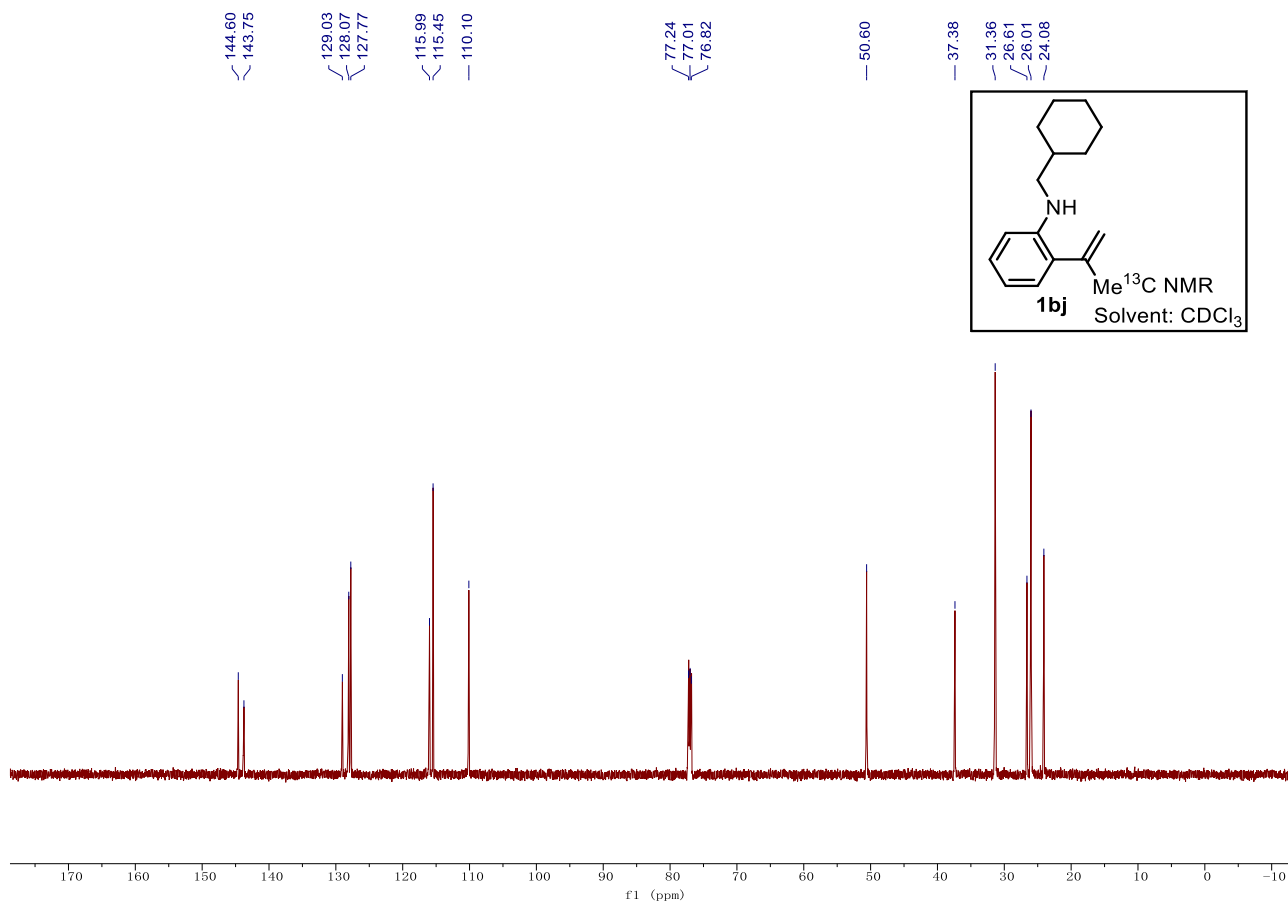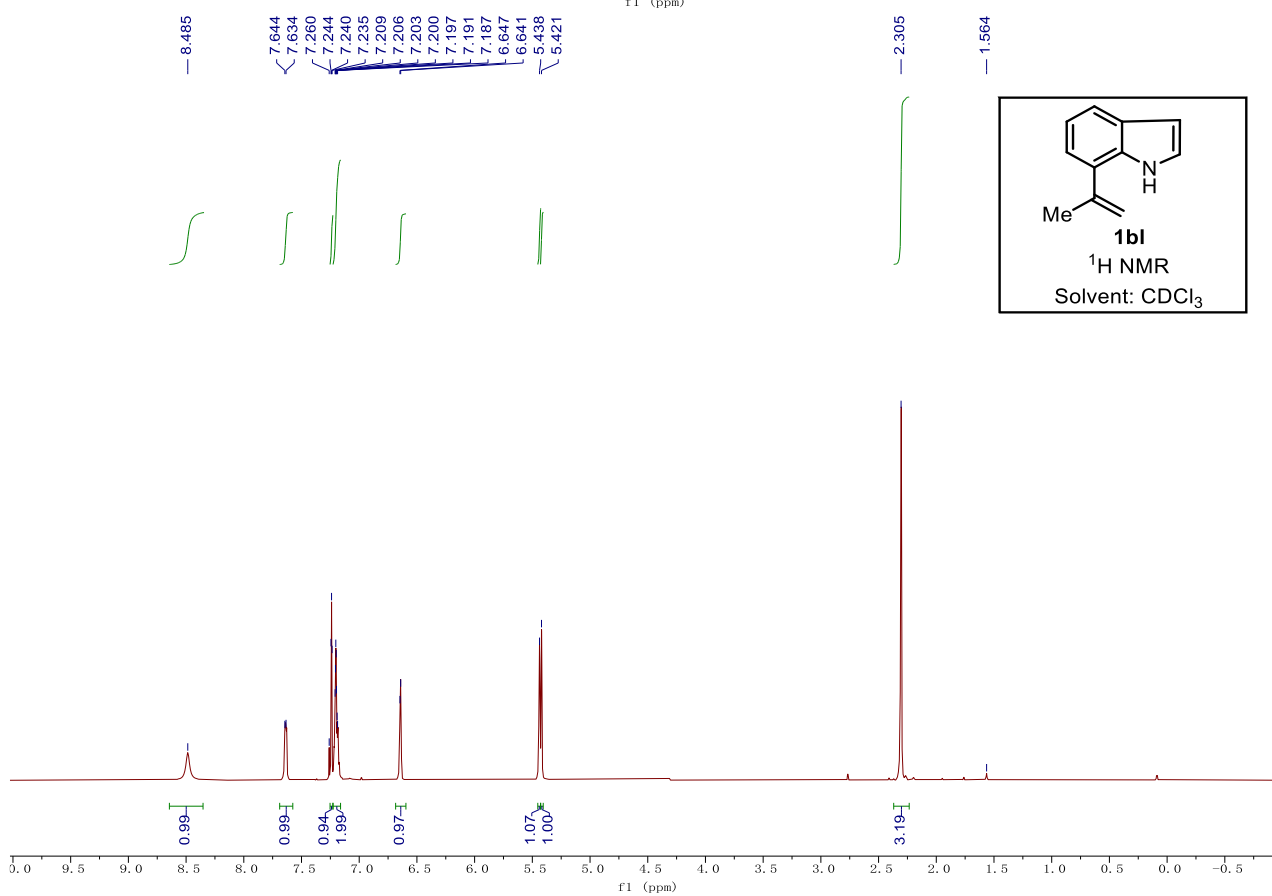

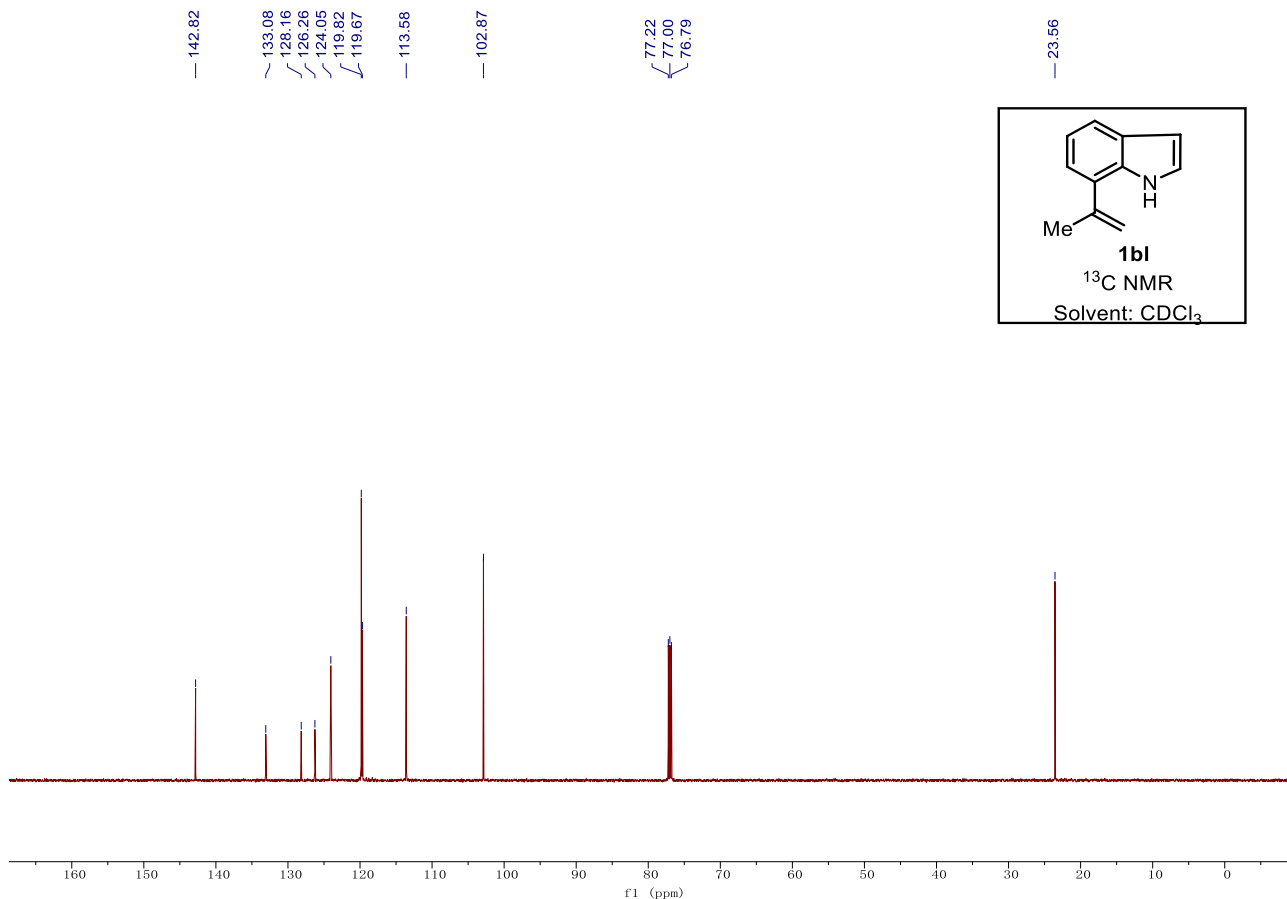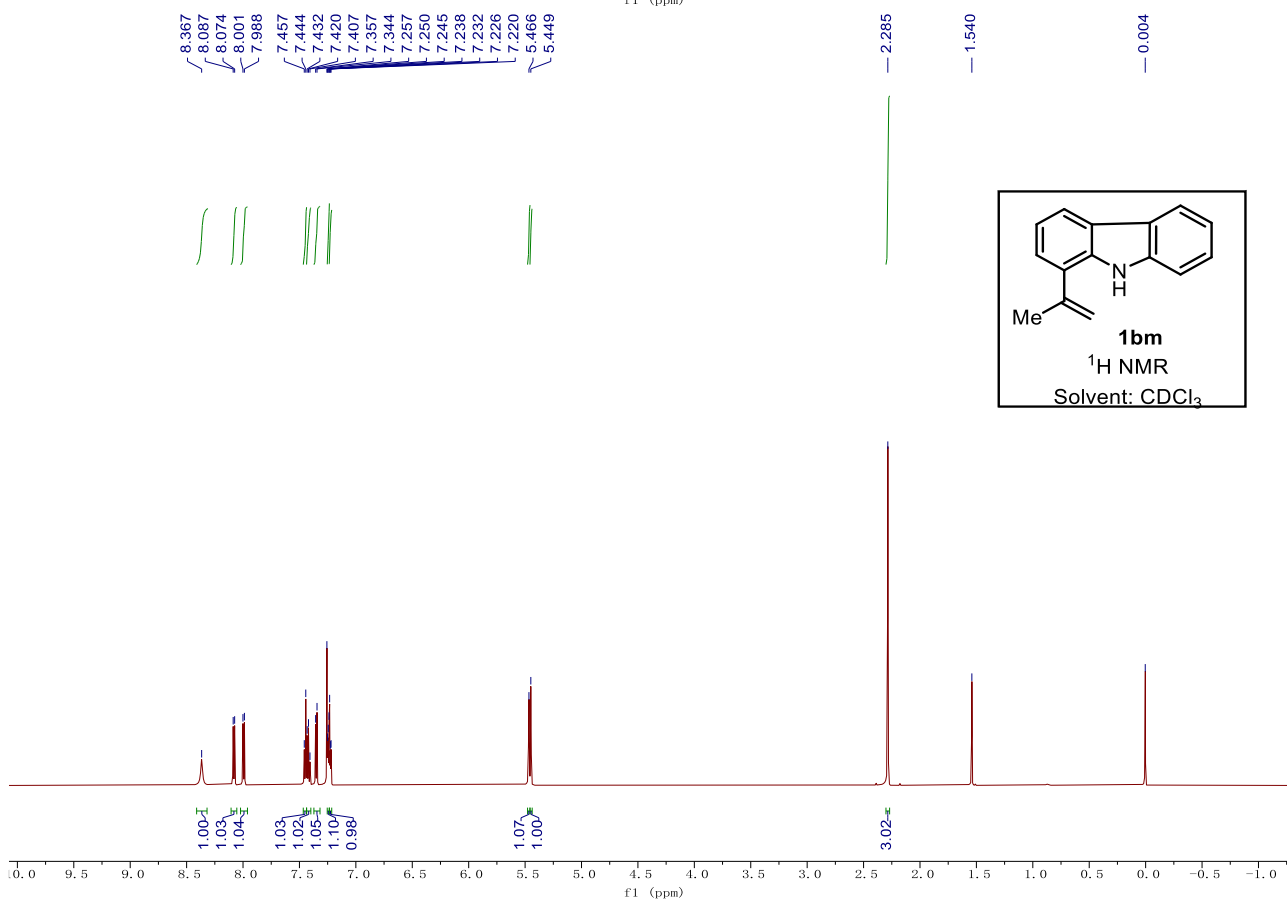

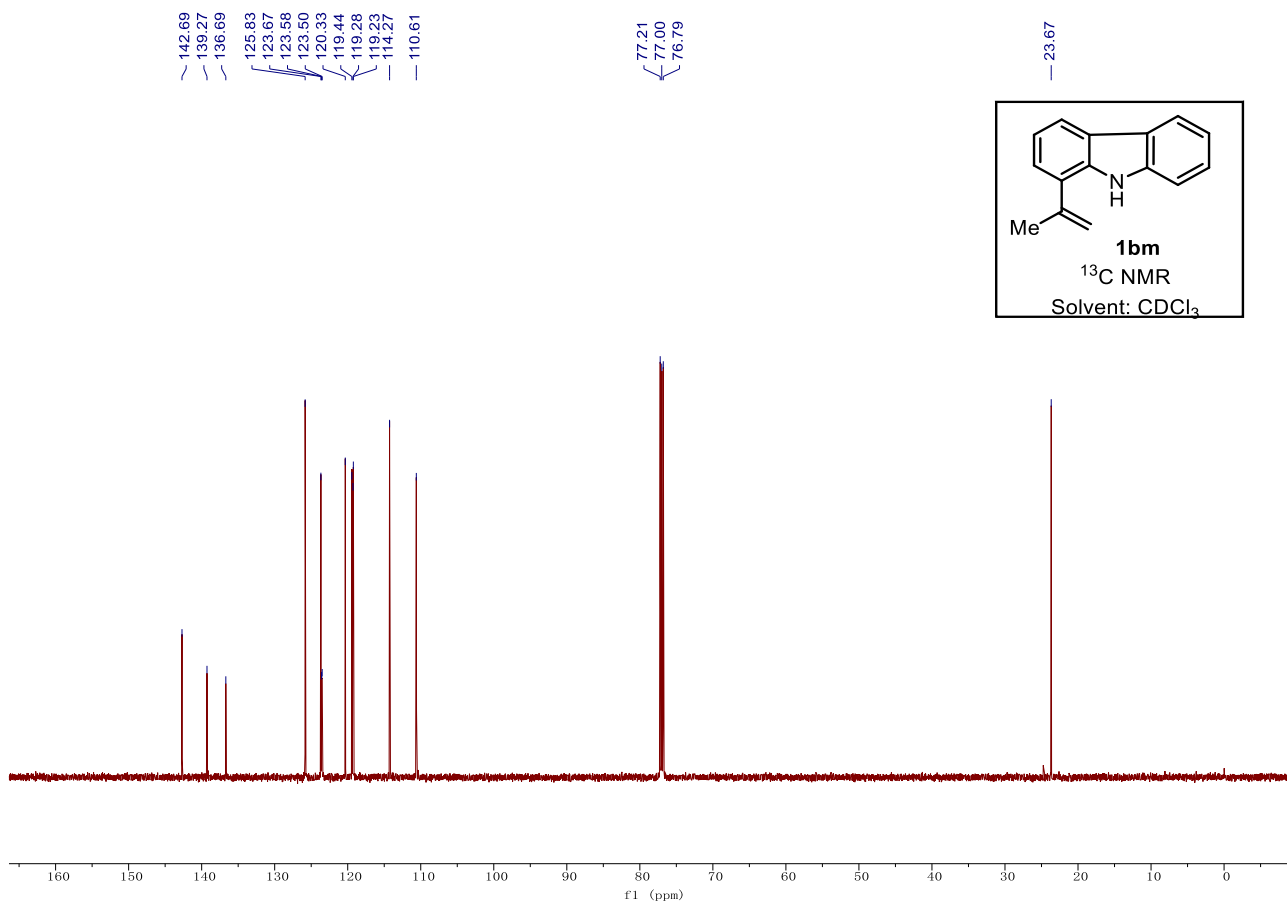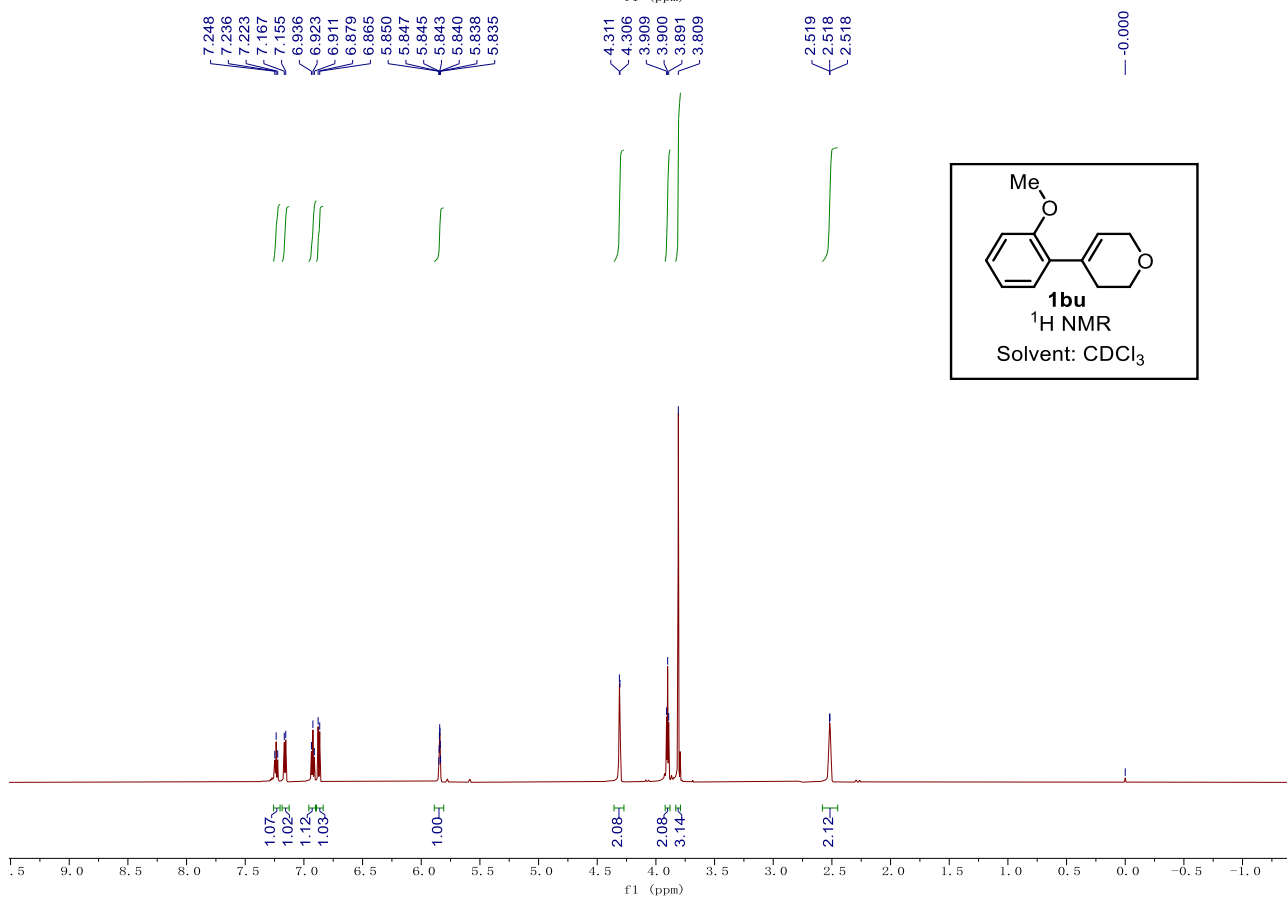

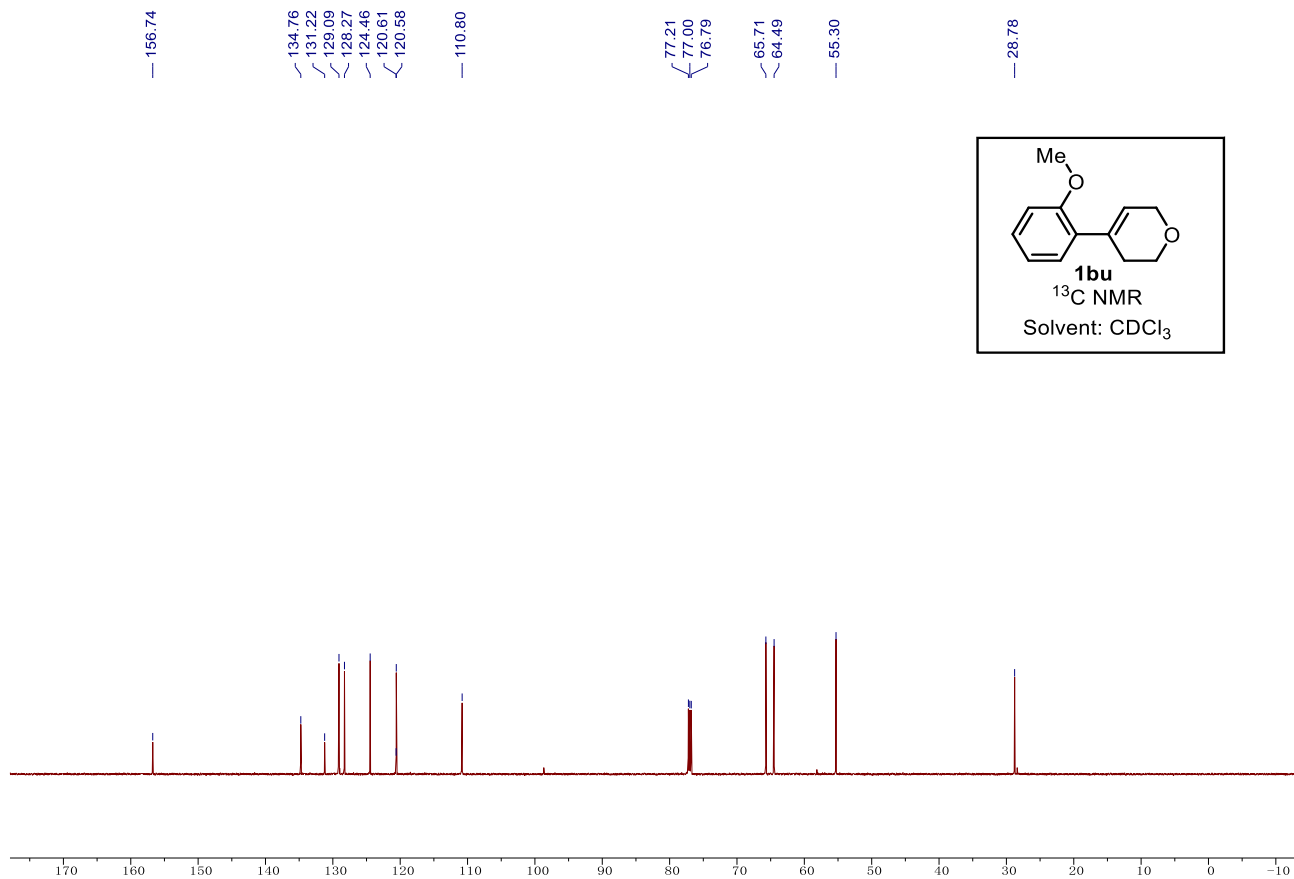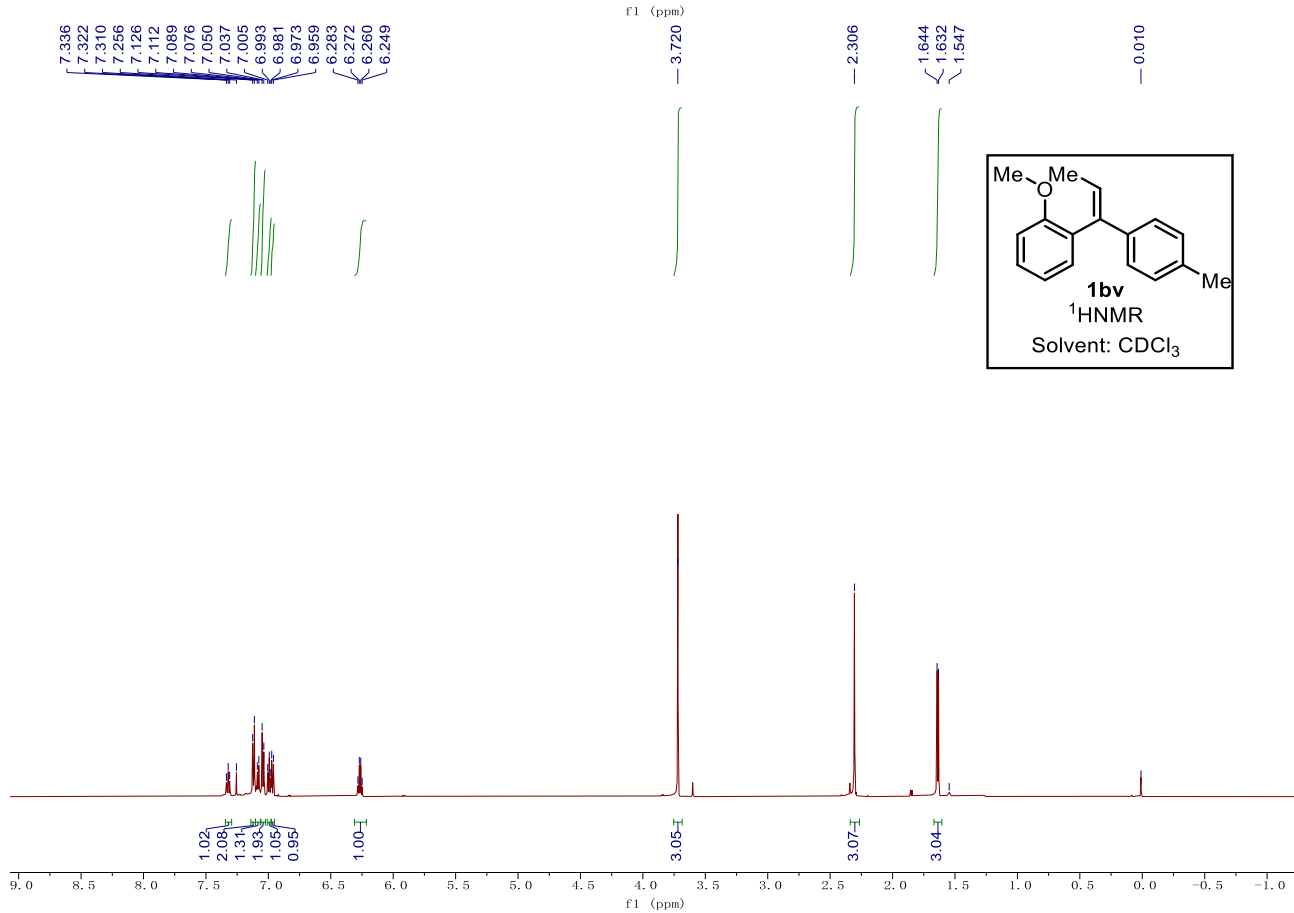

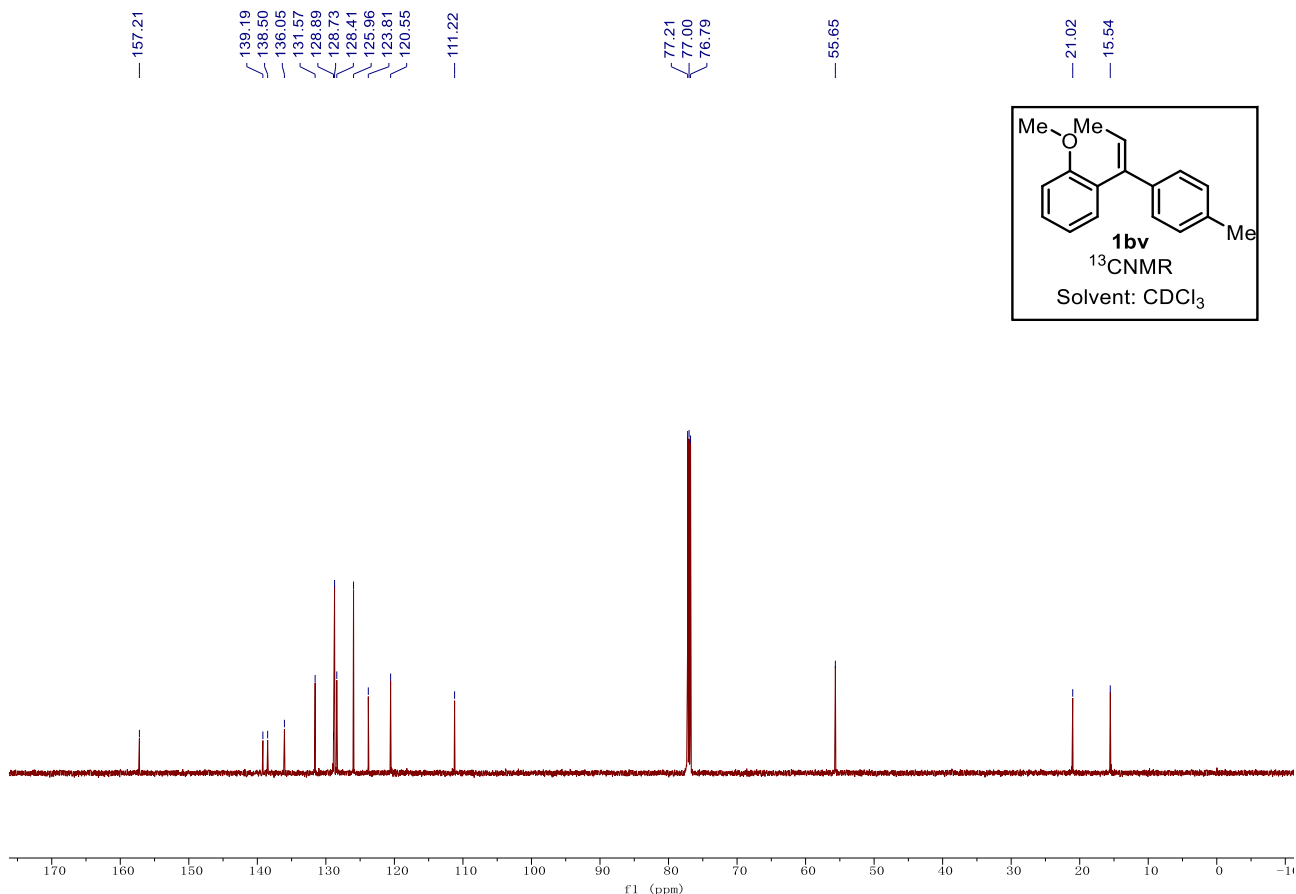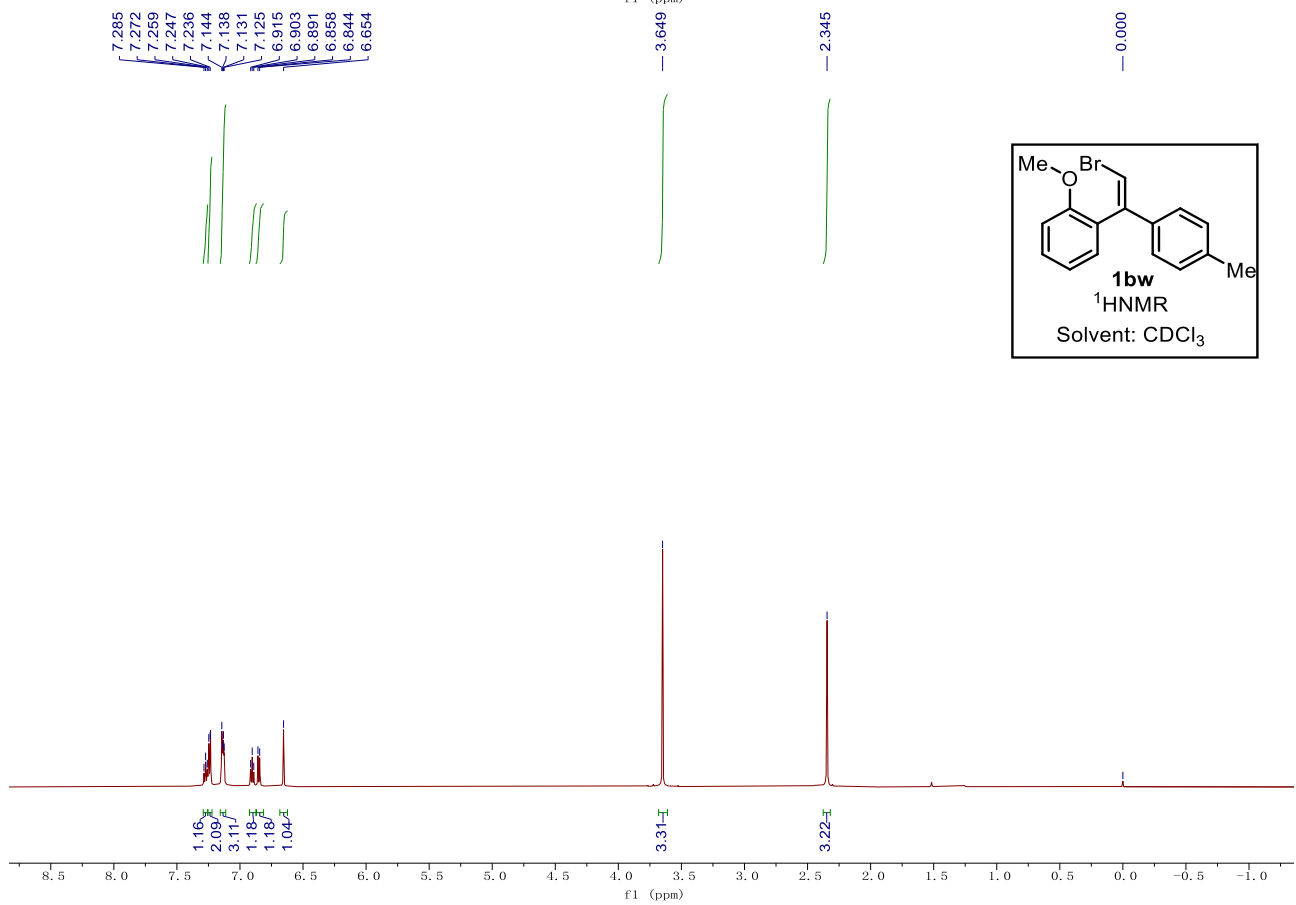

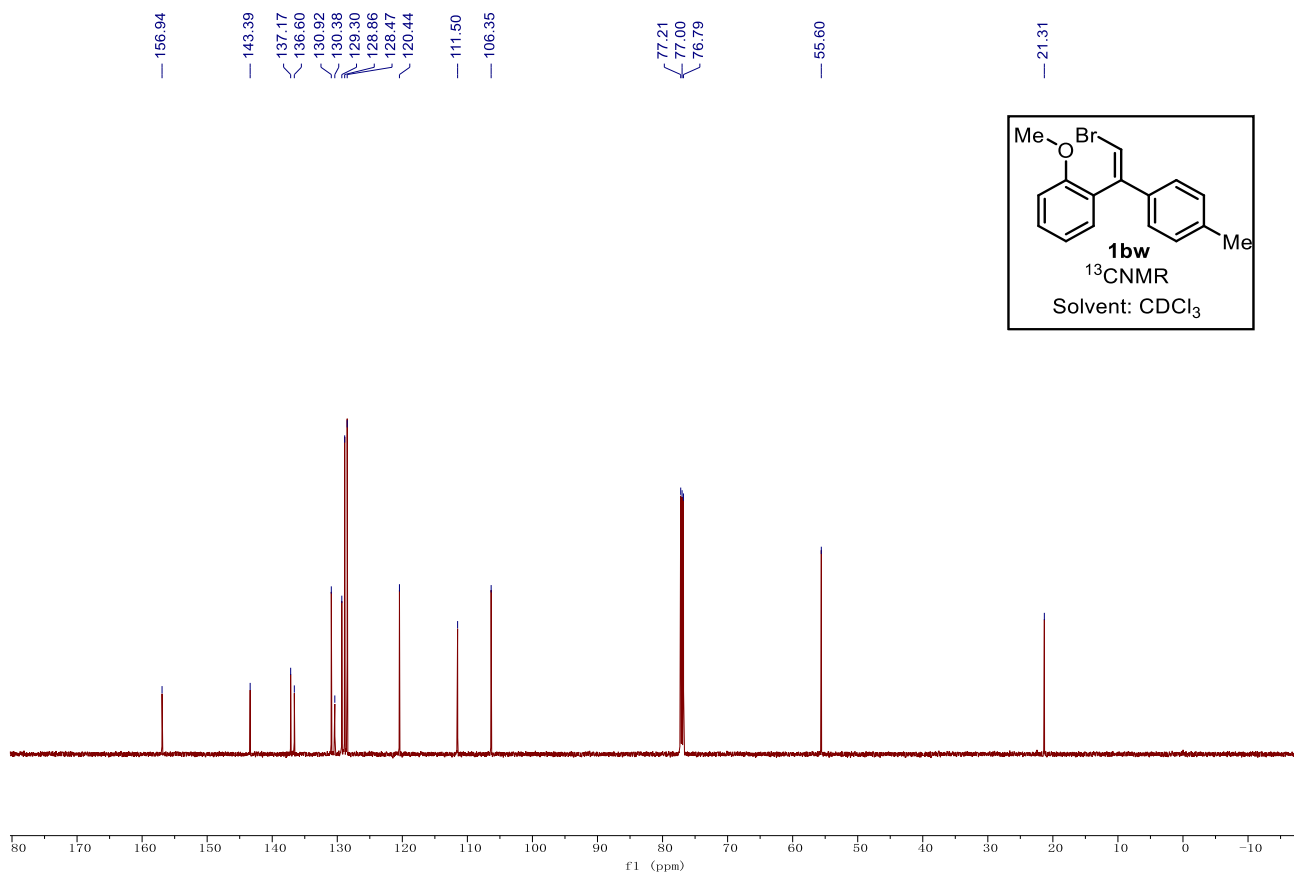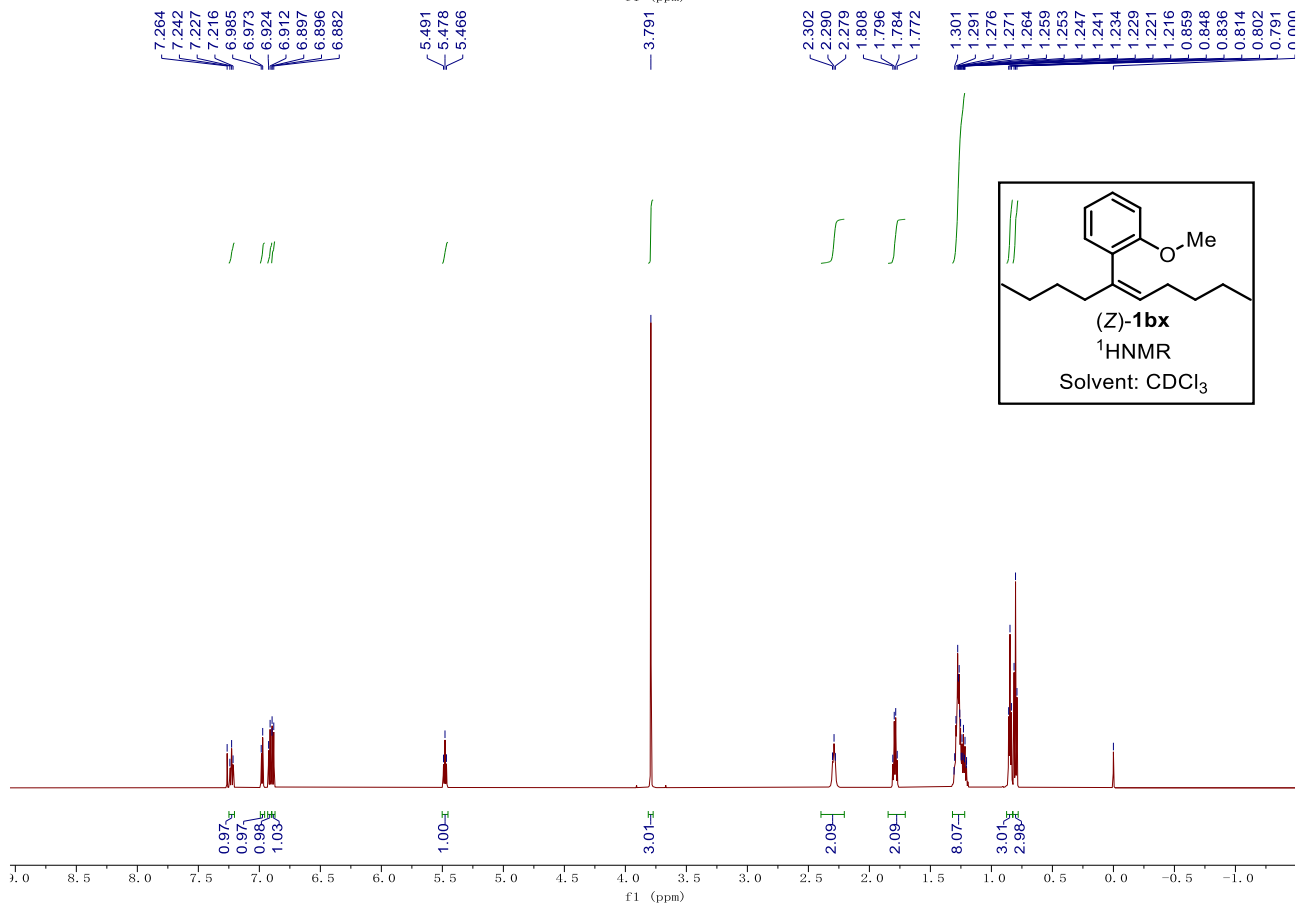

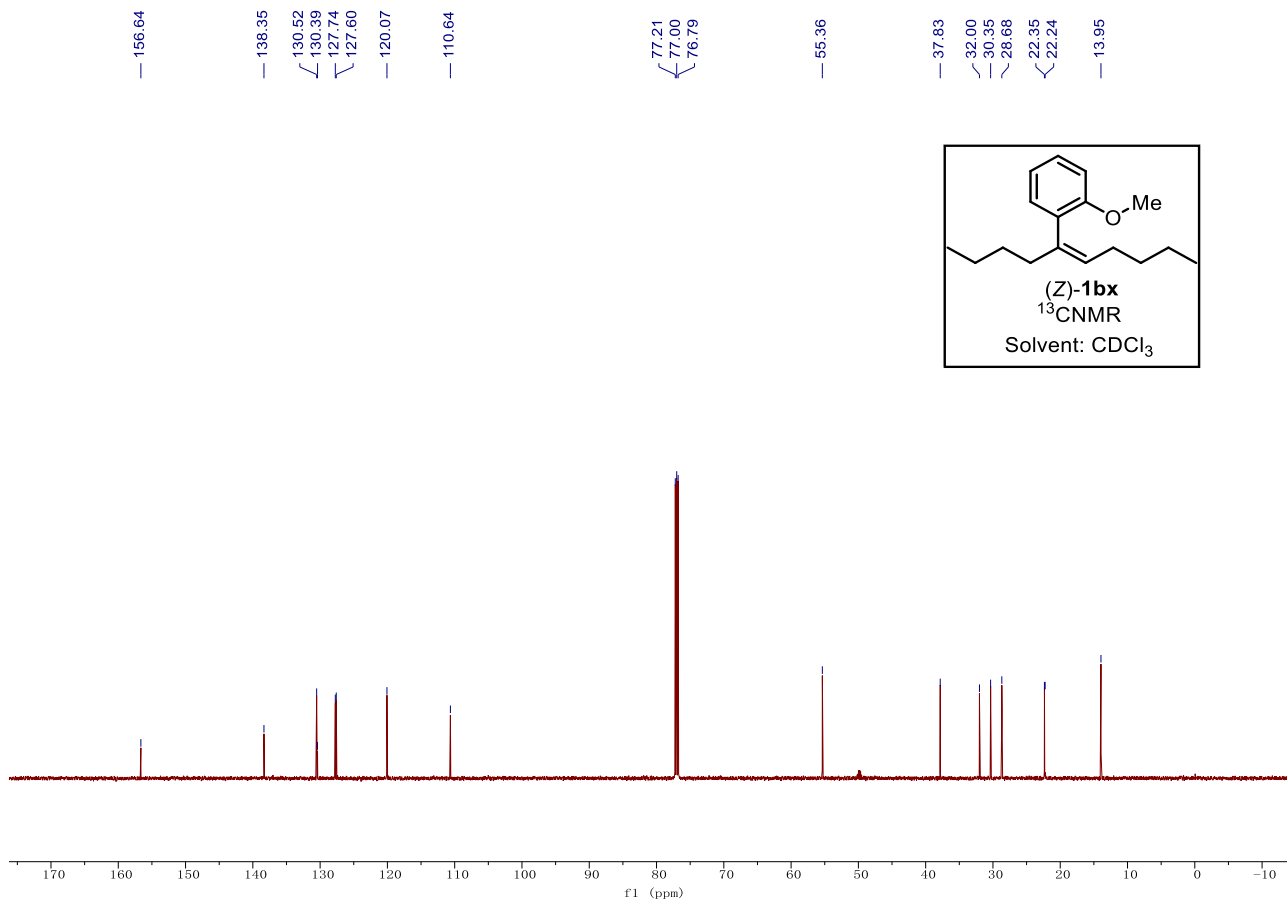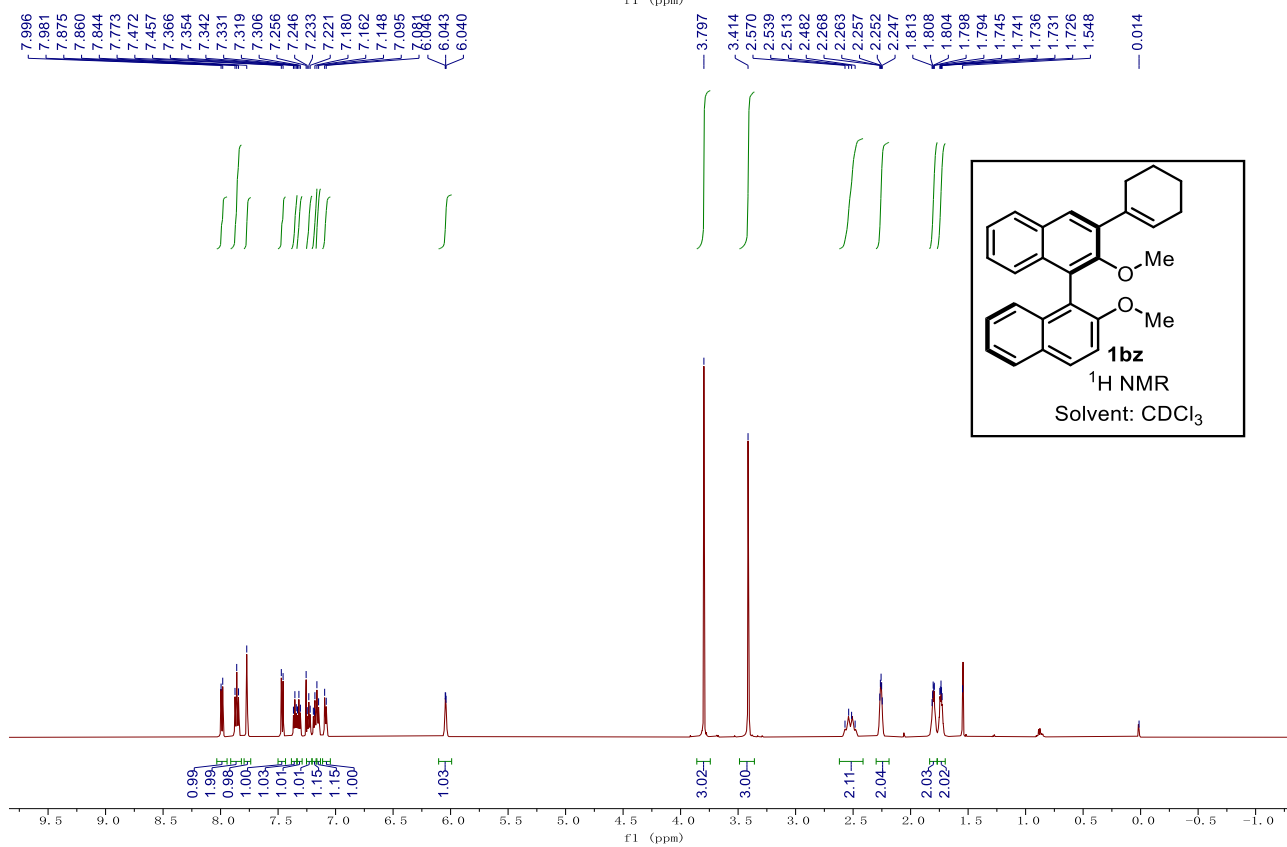

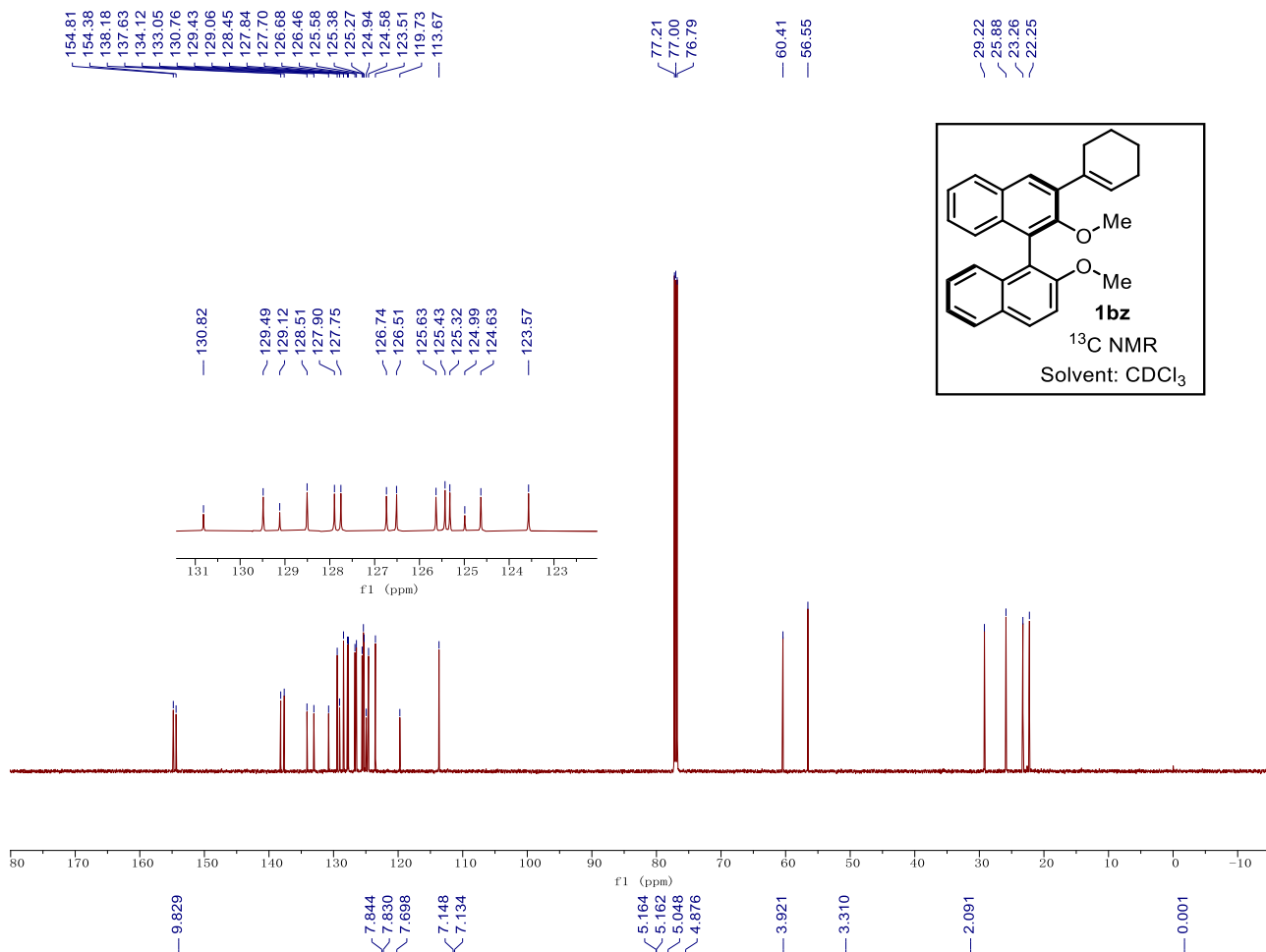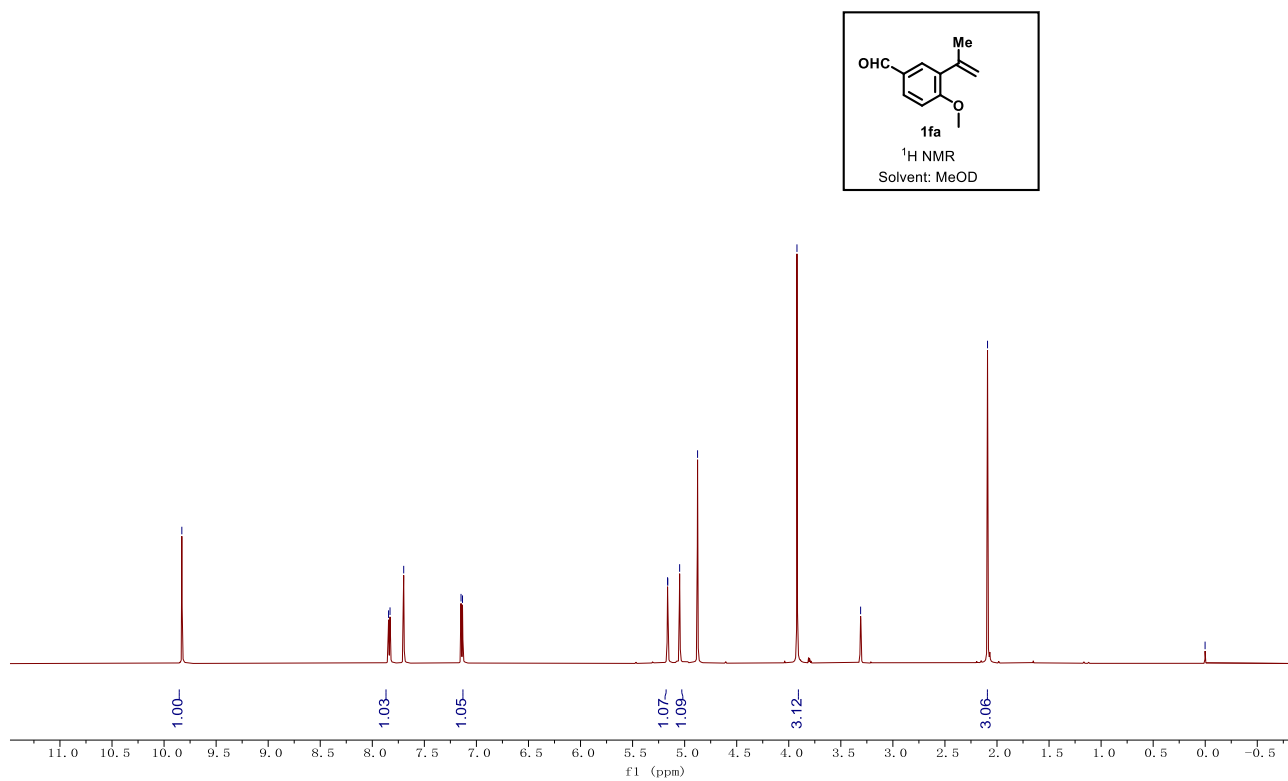

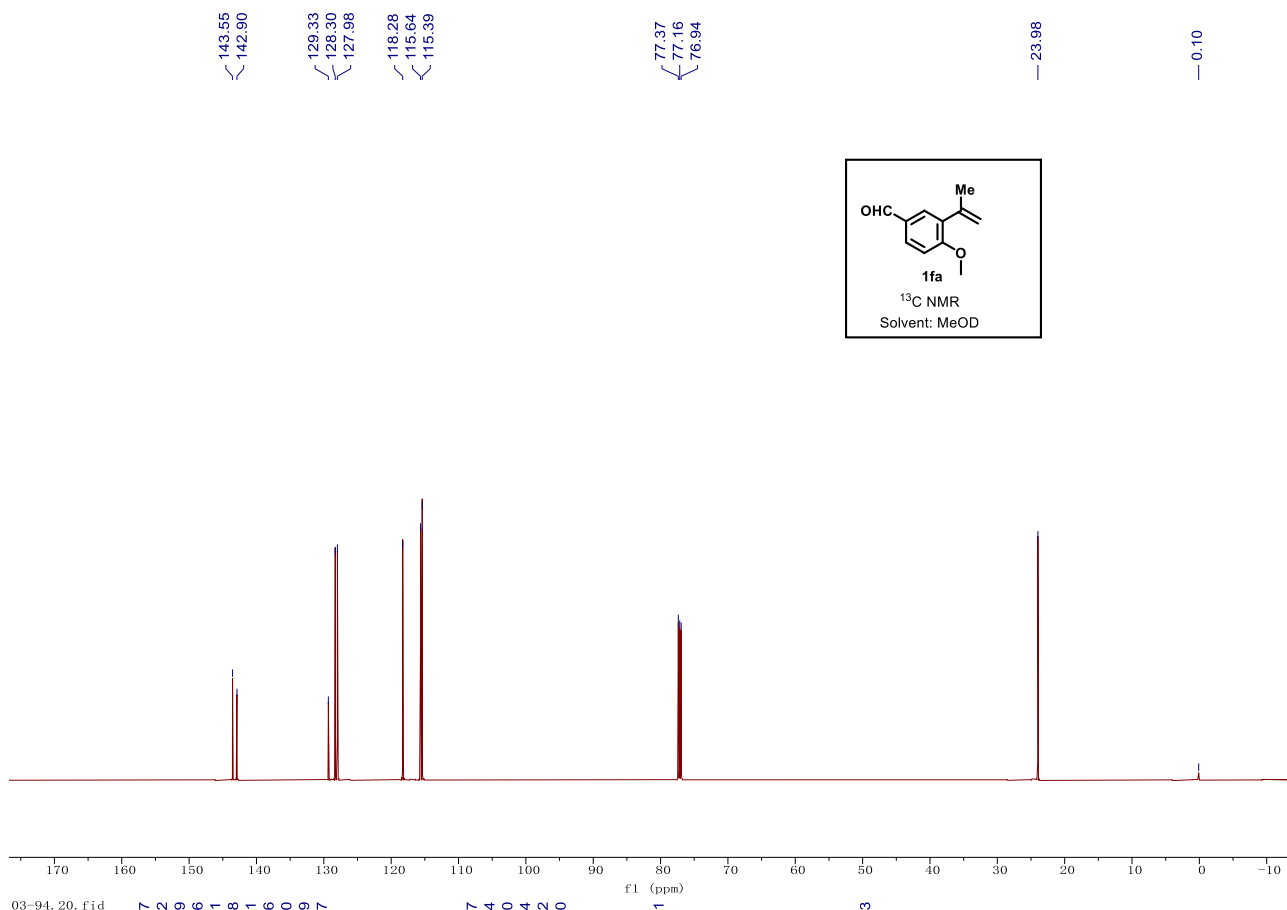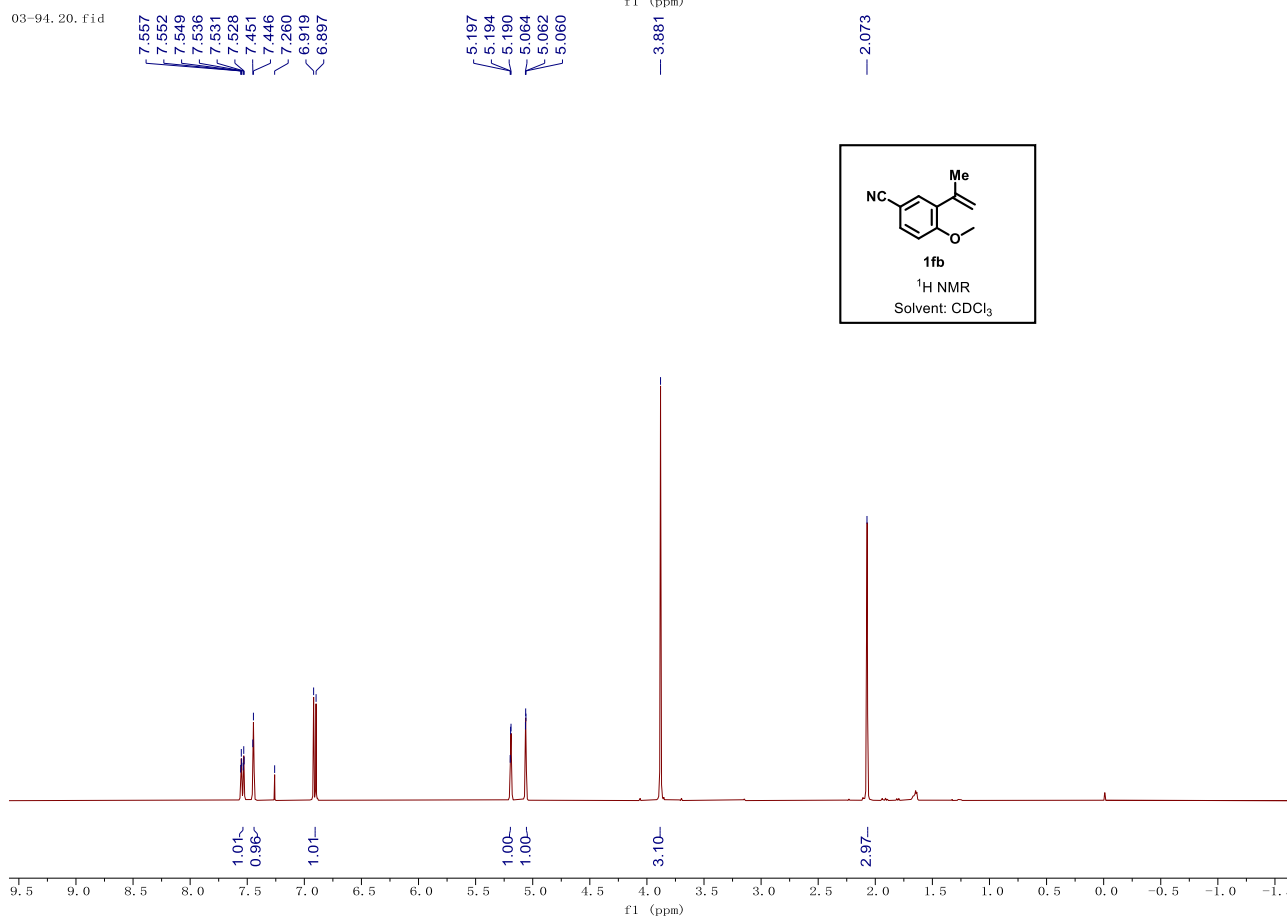

03-94.22.fid

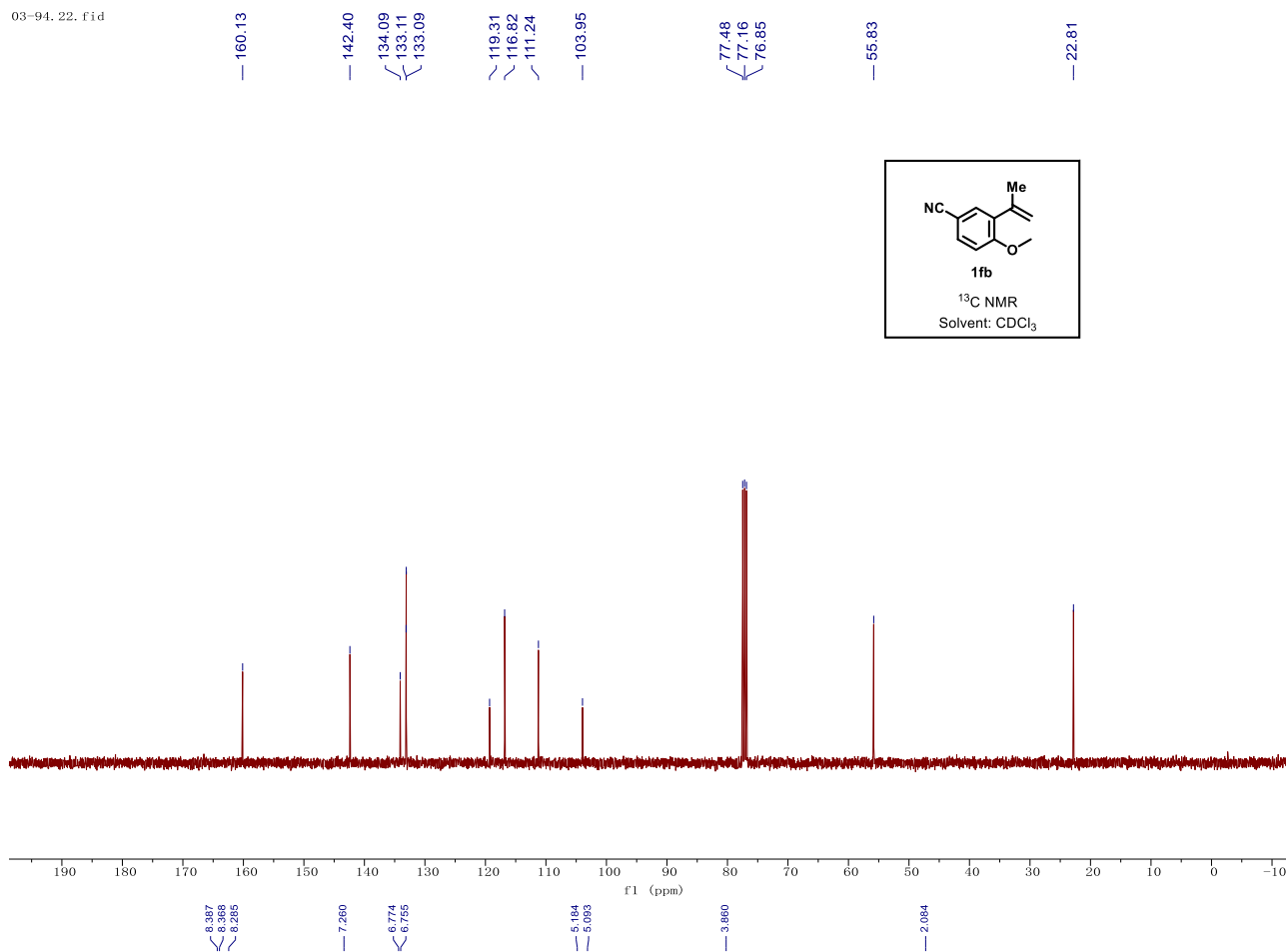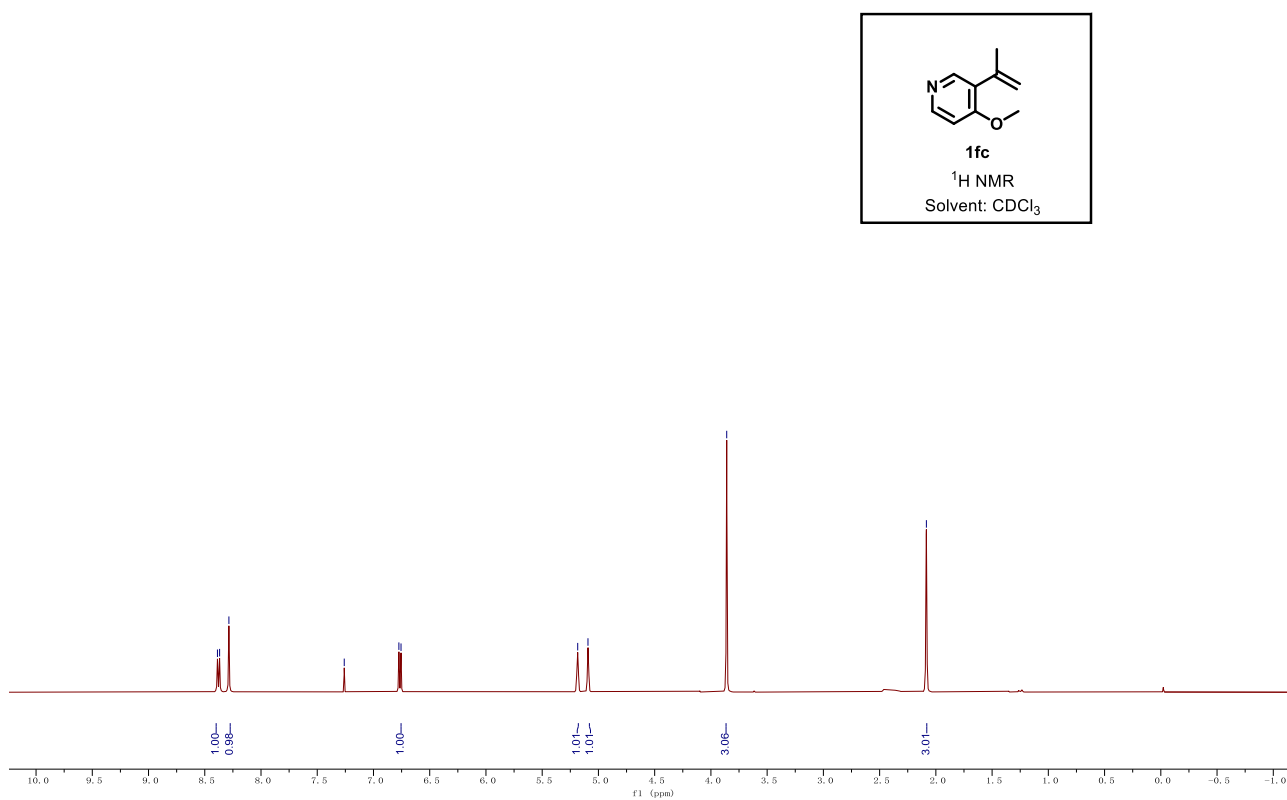

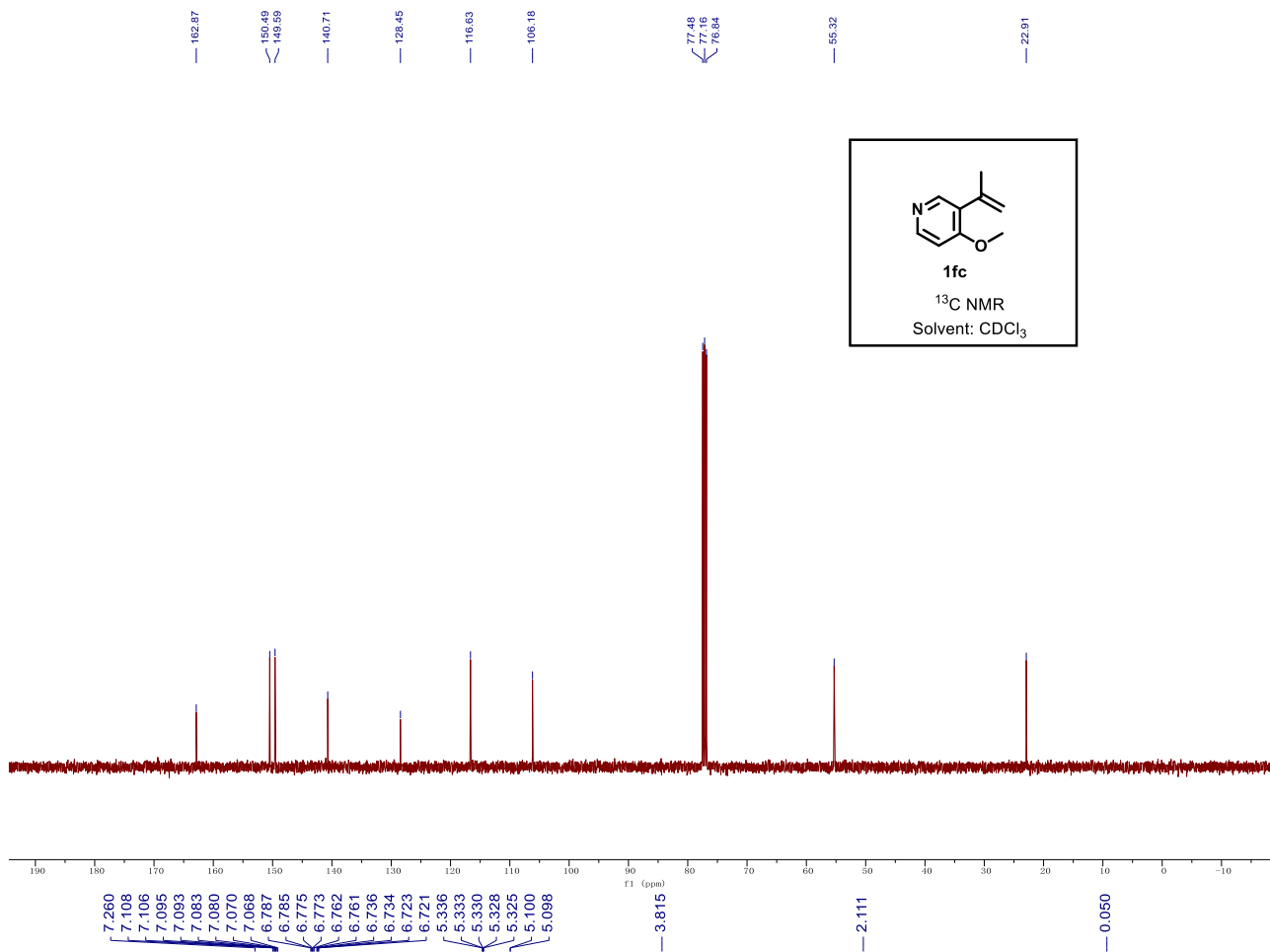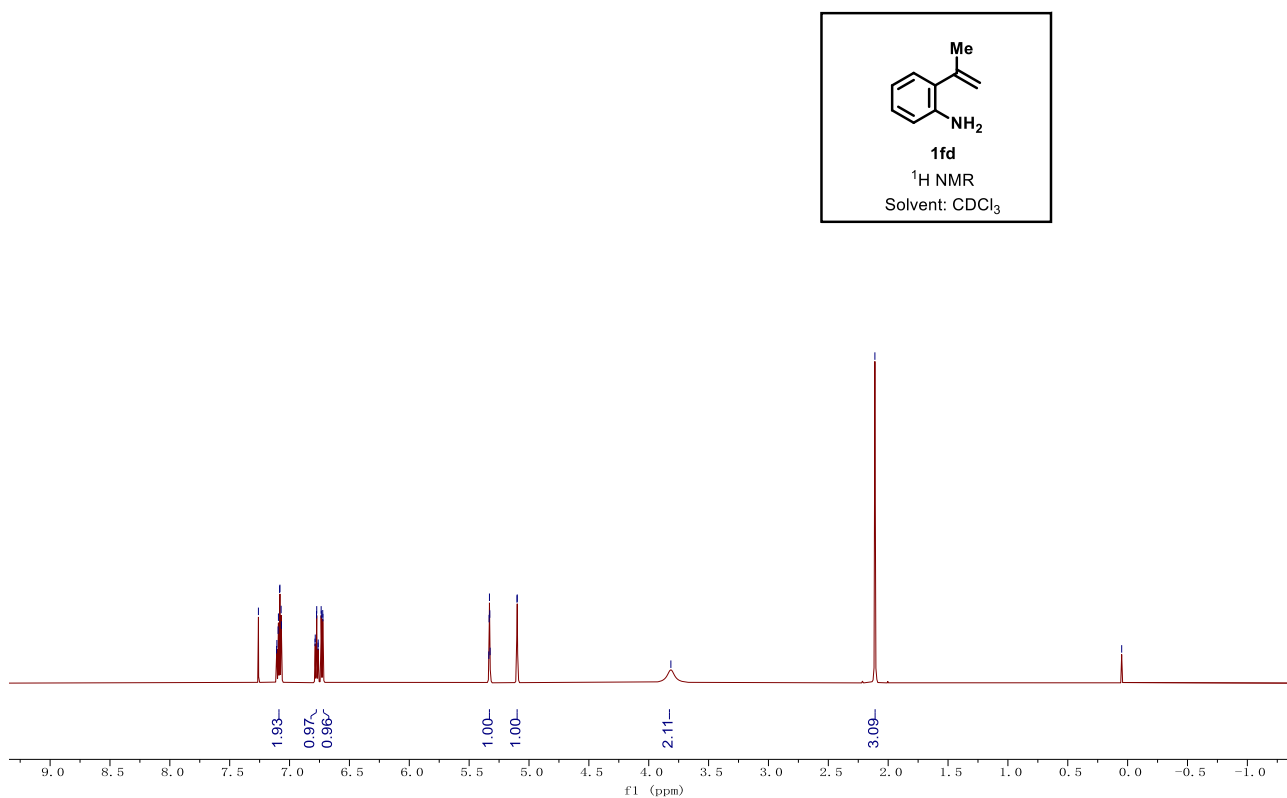

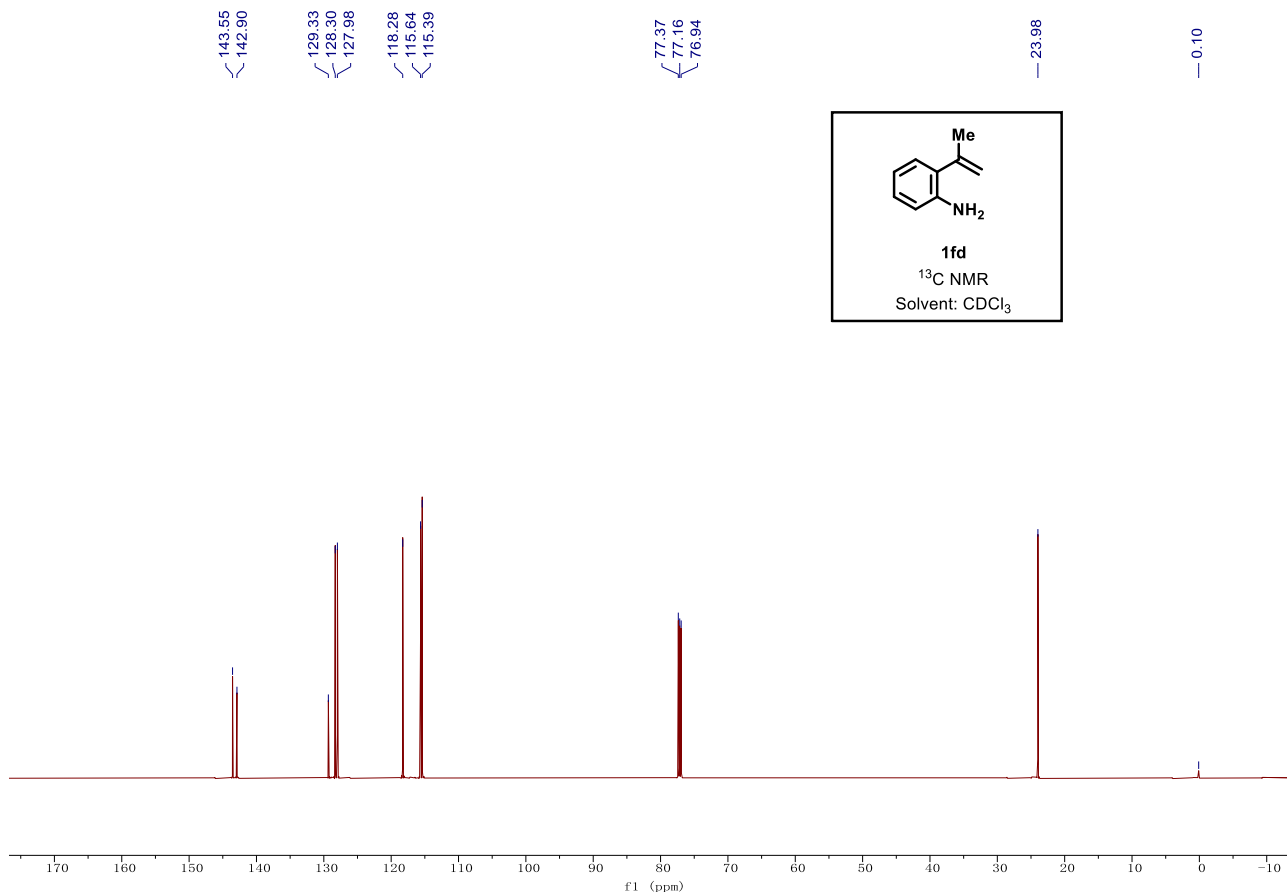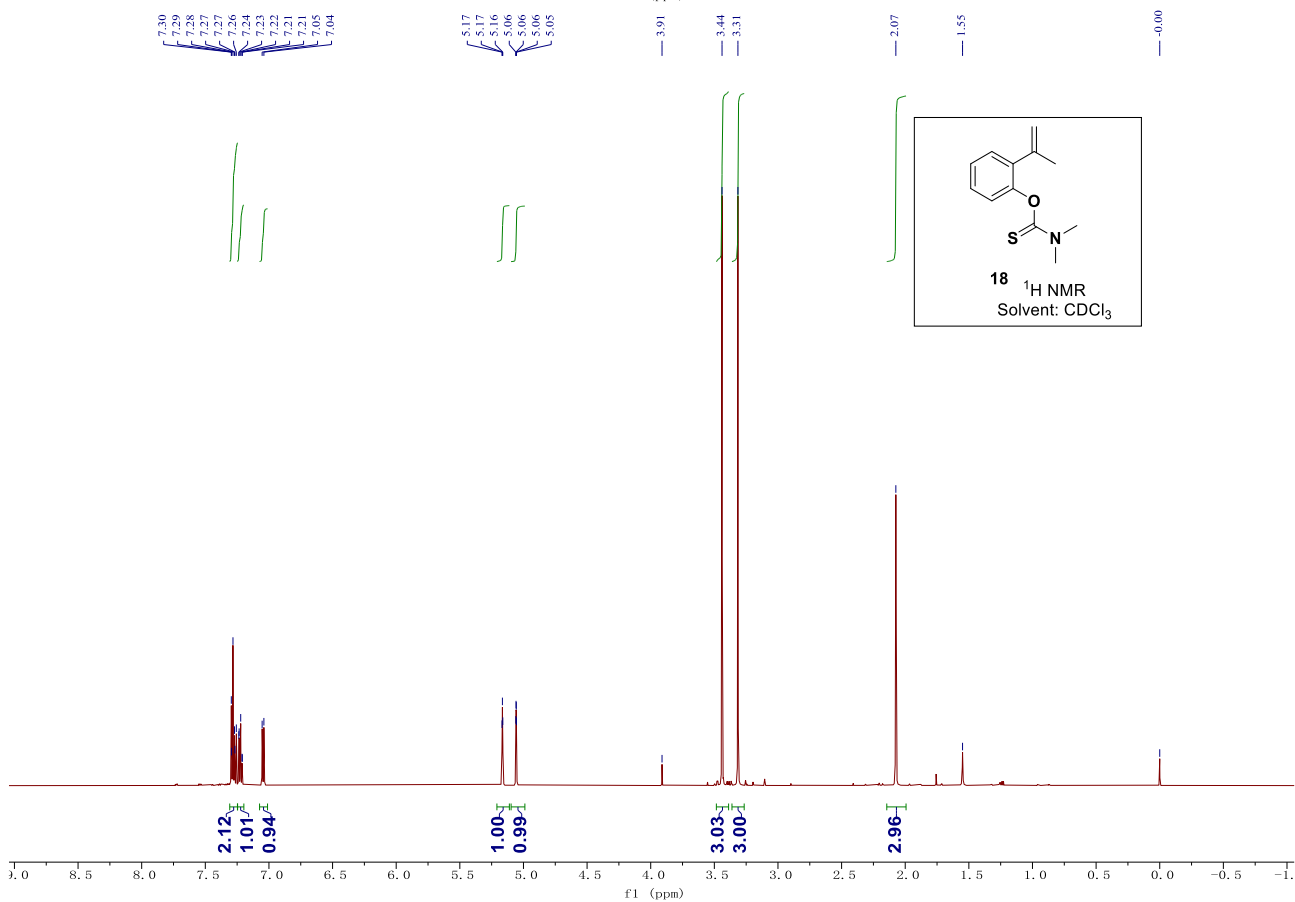

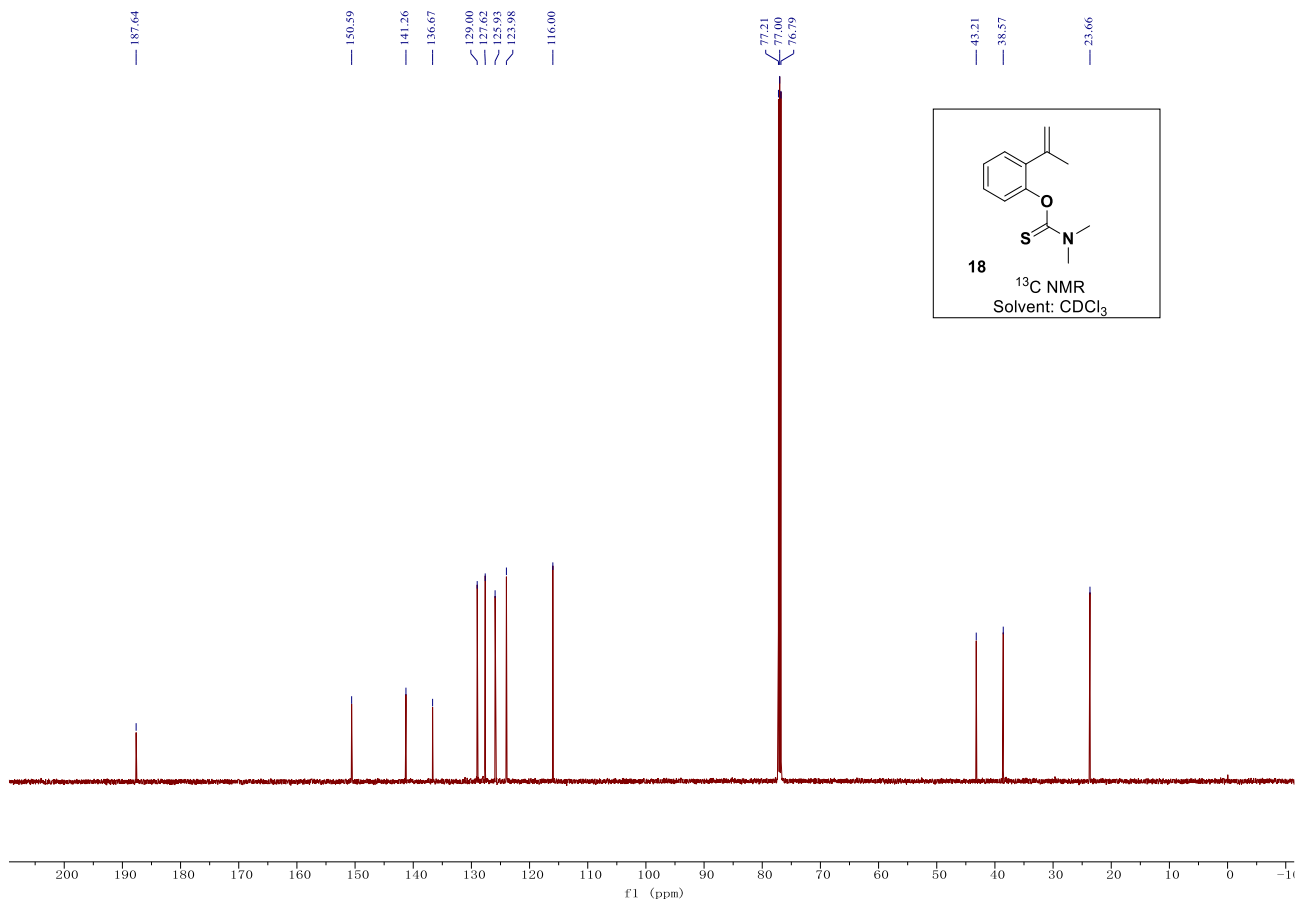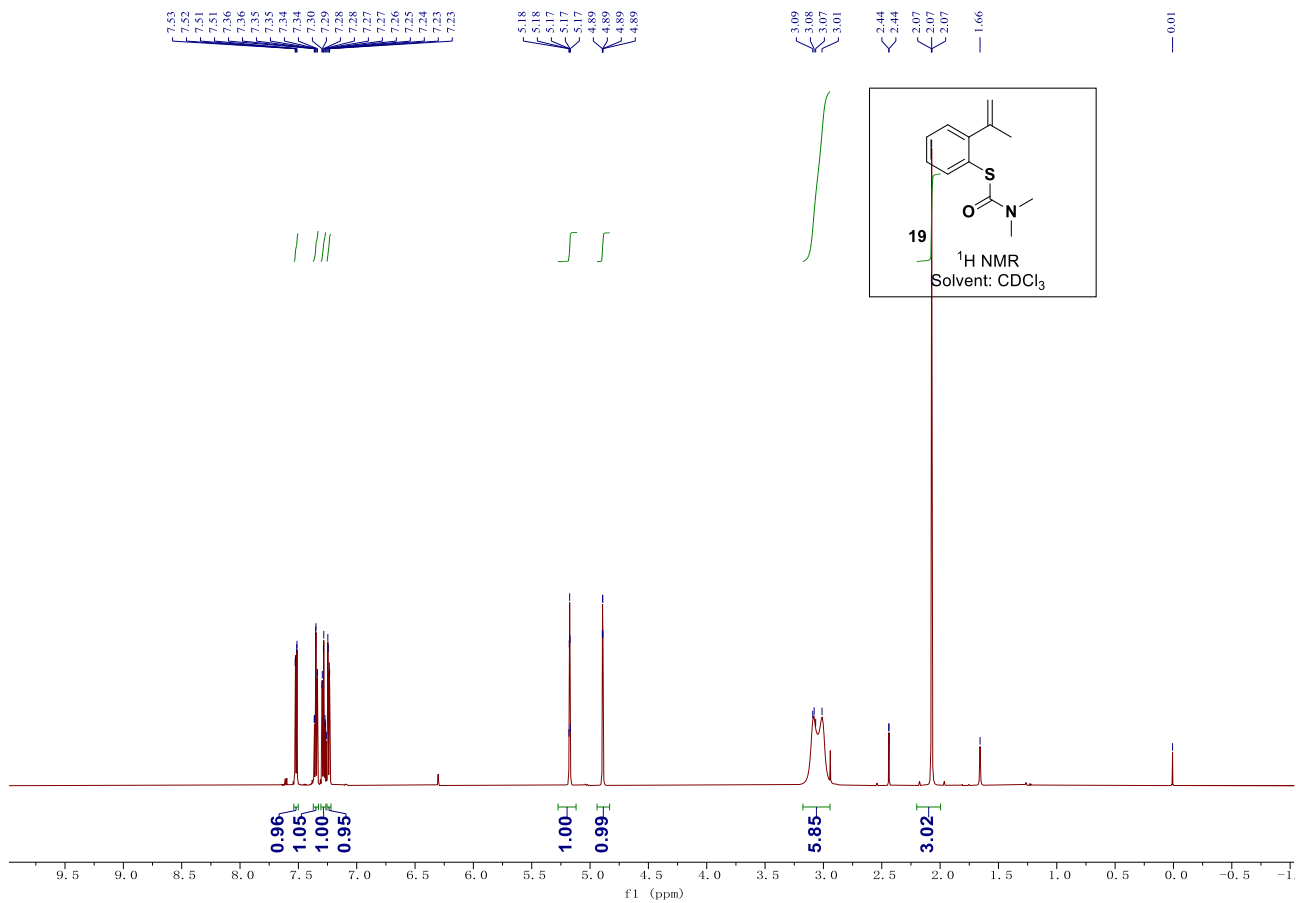

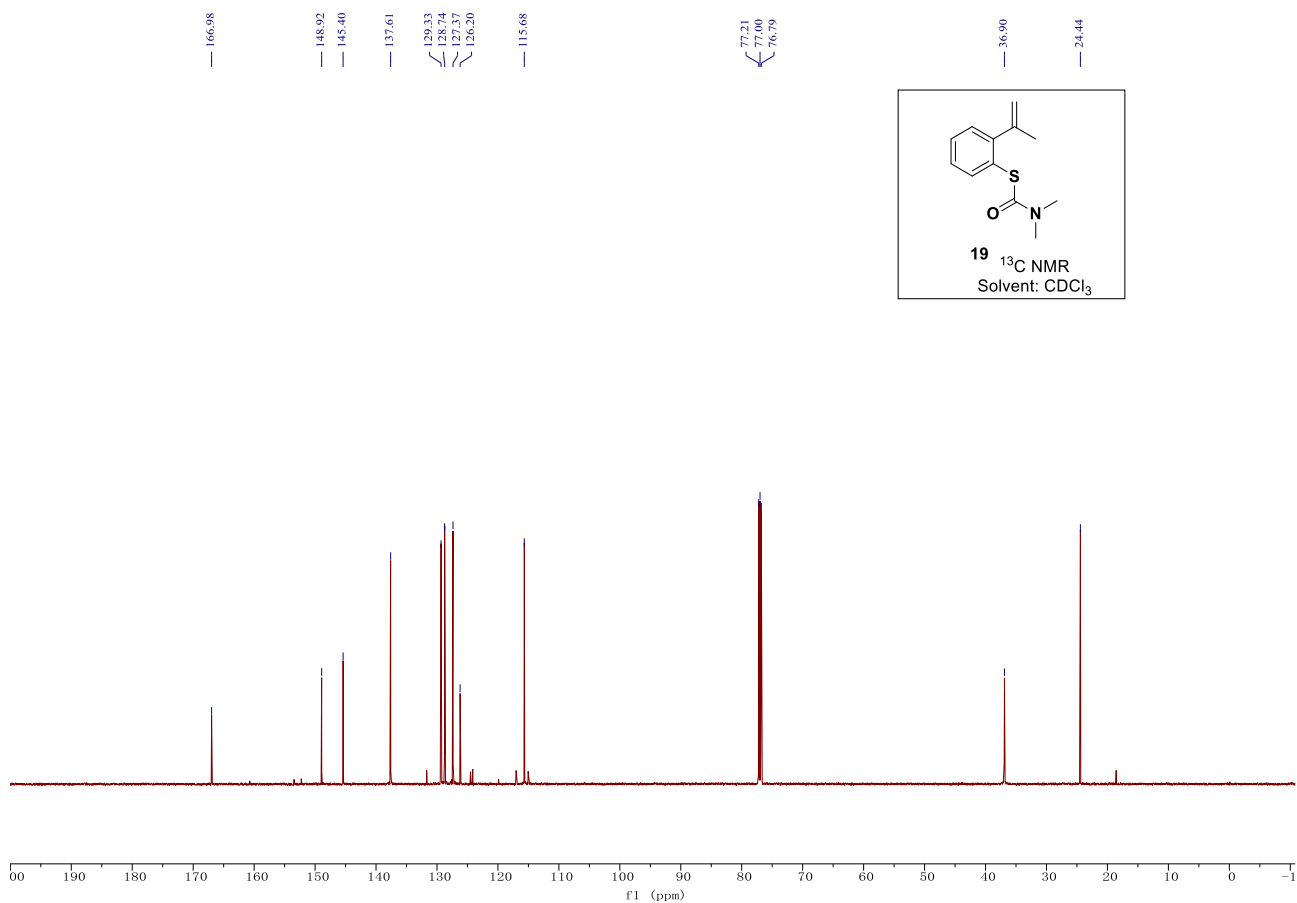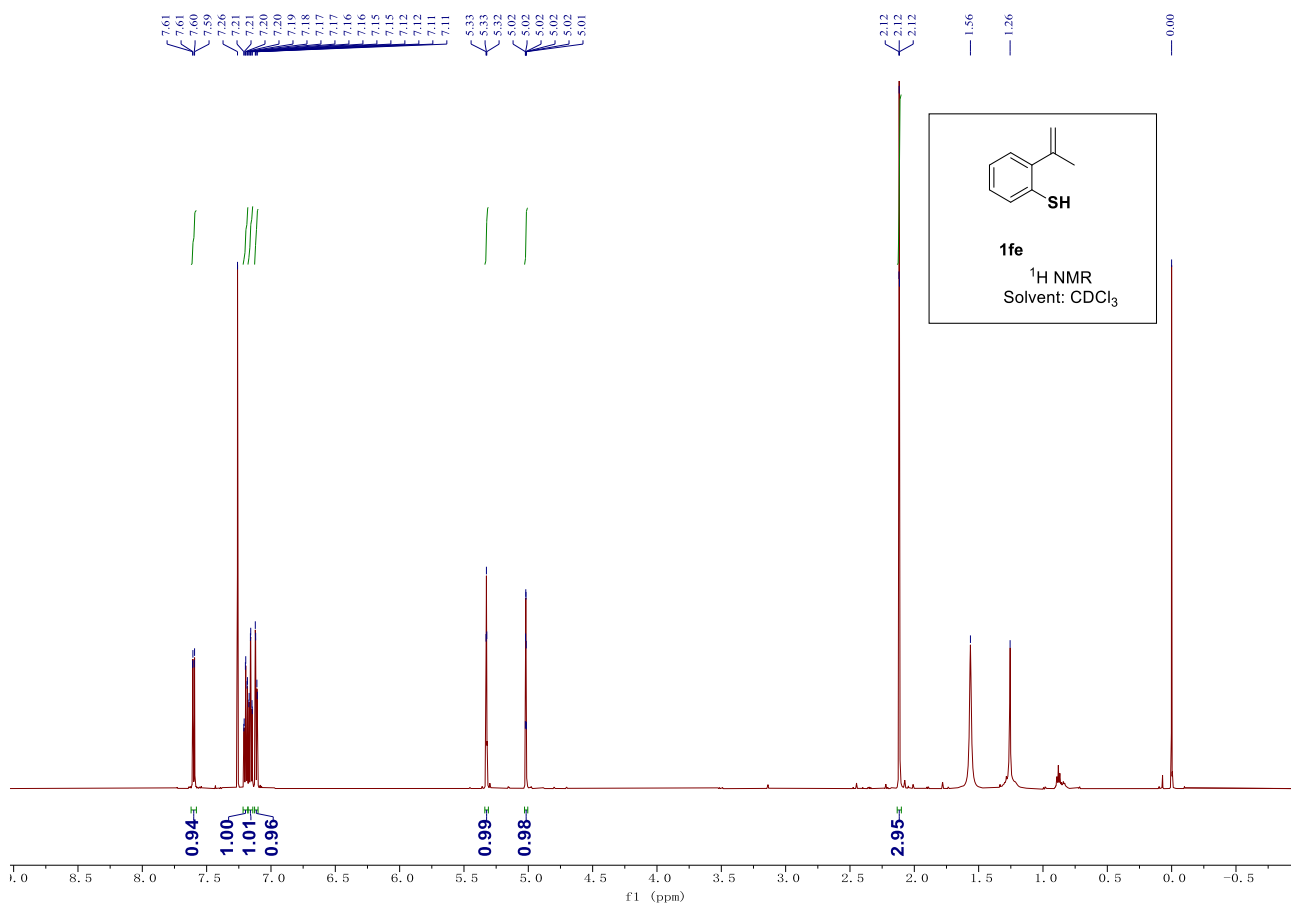

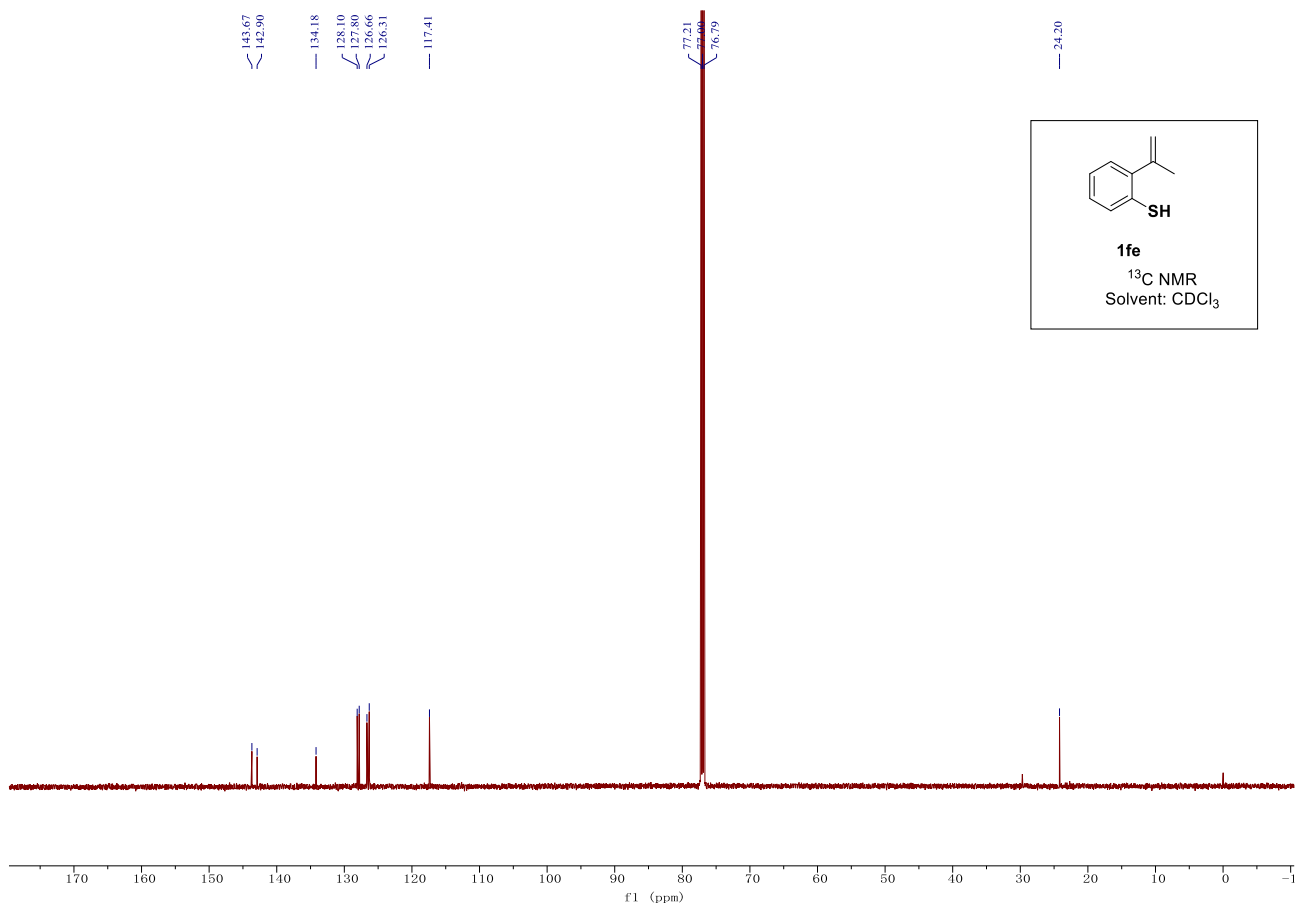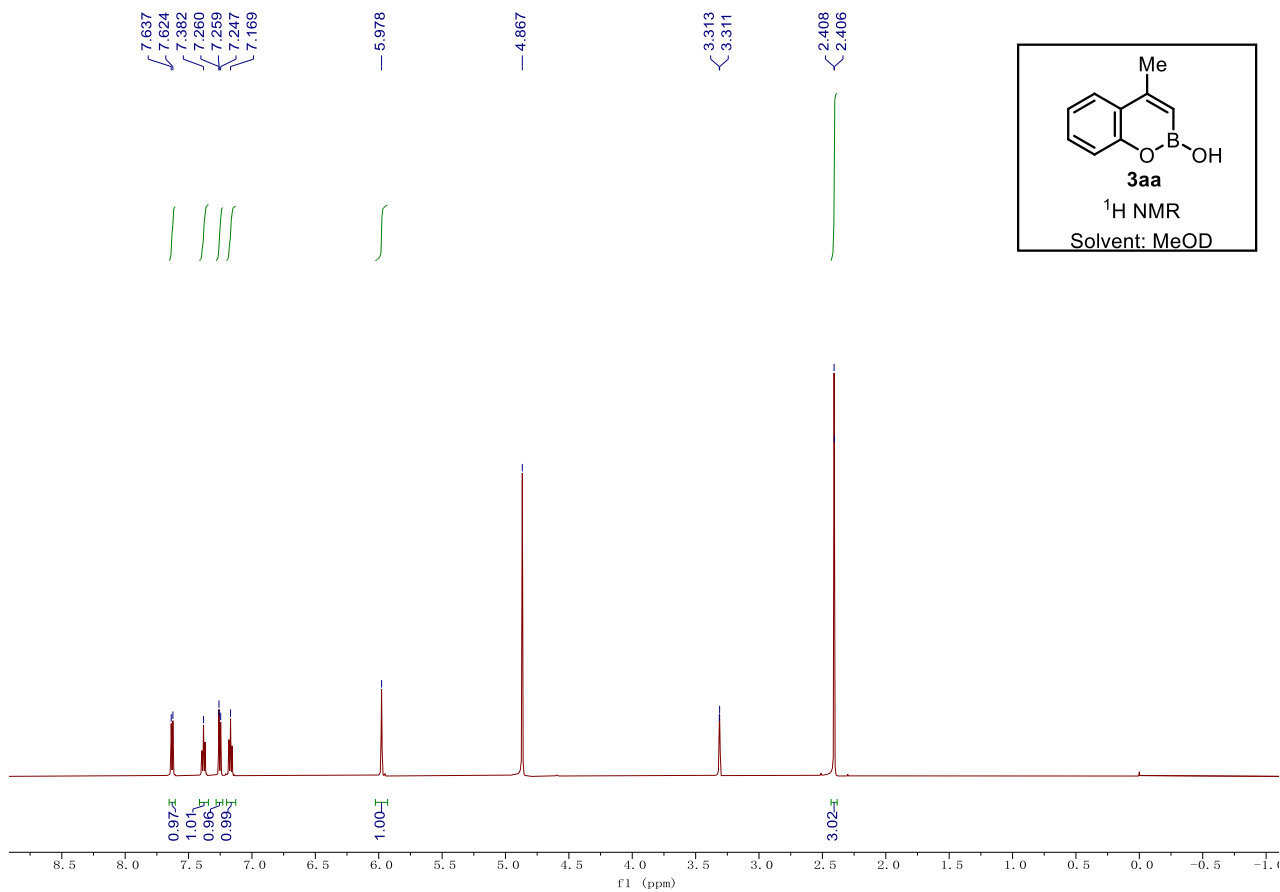

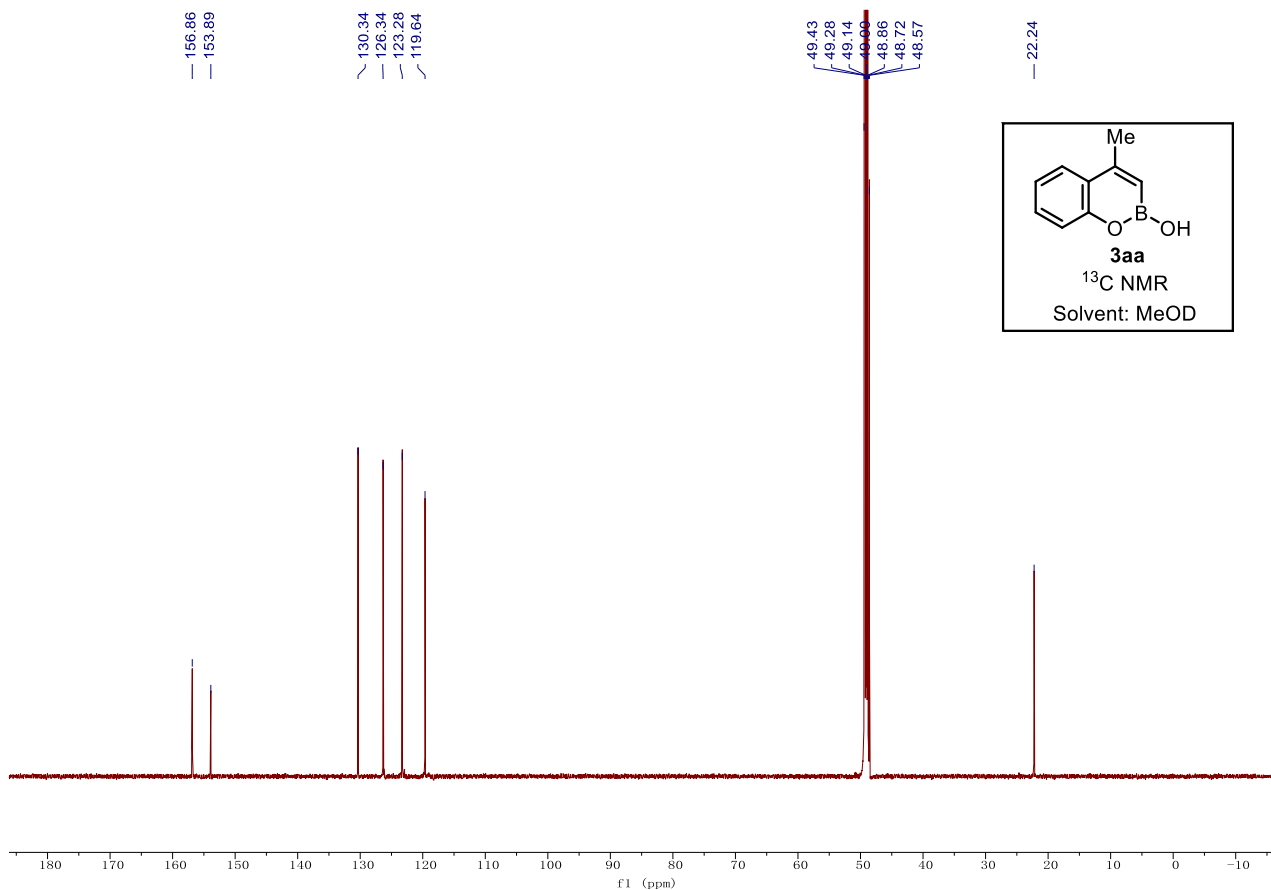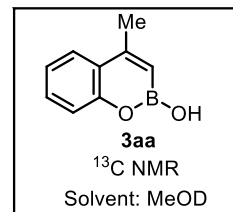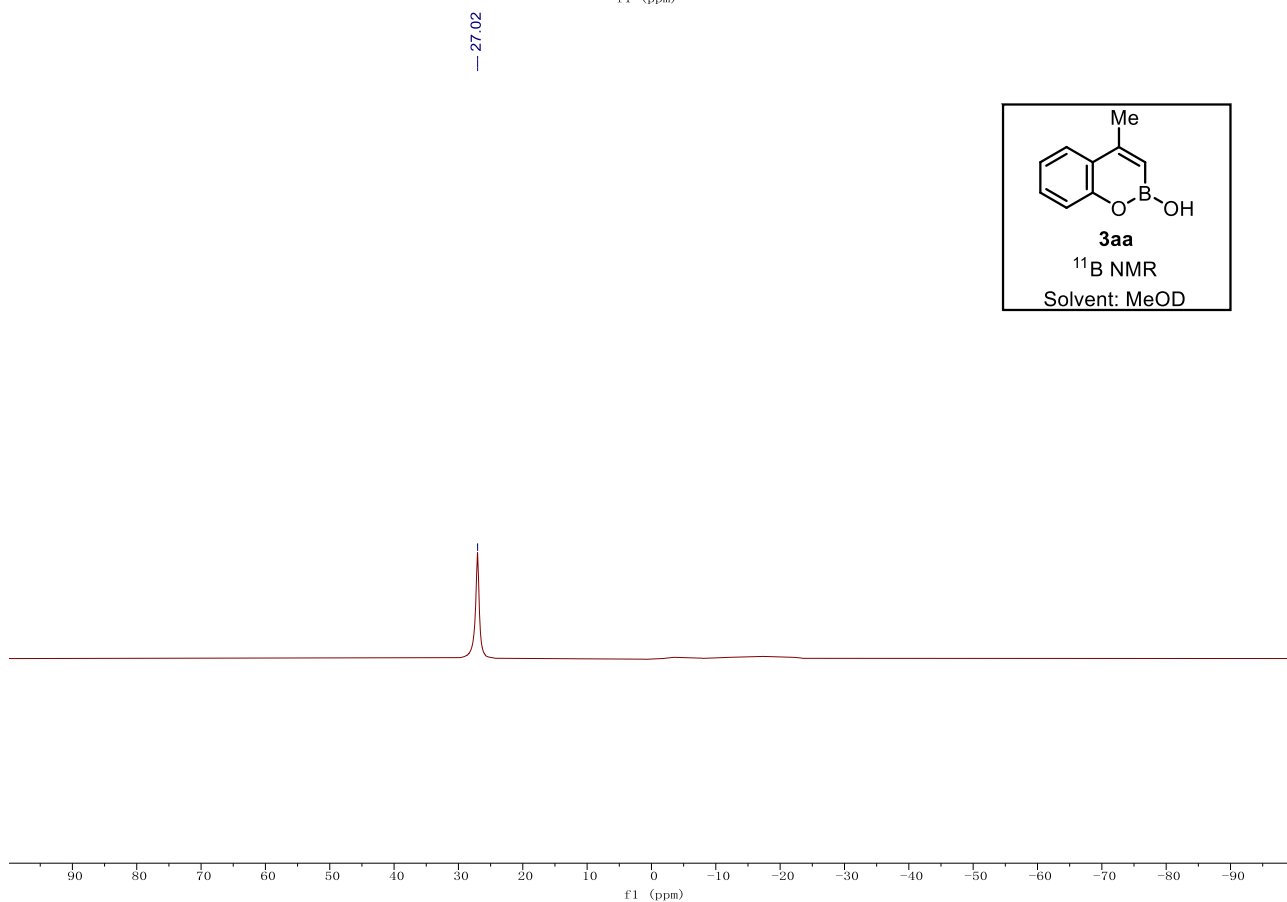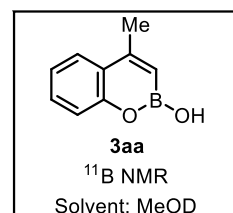

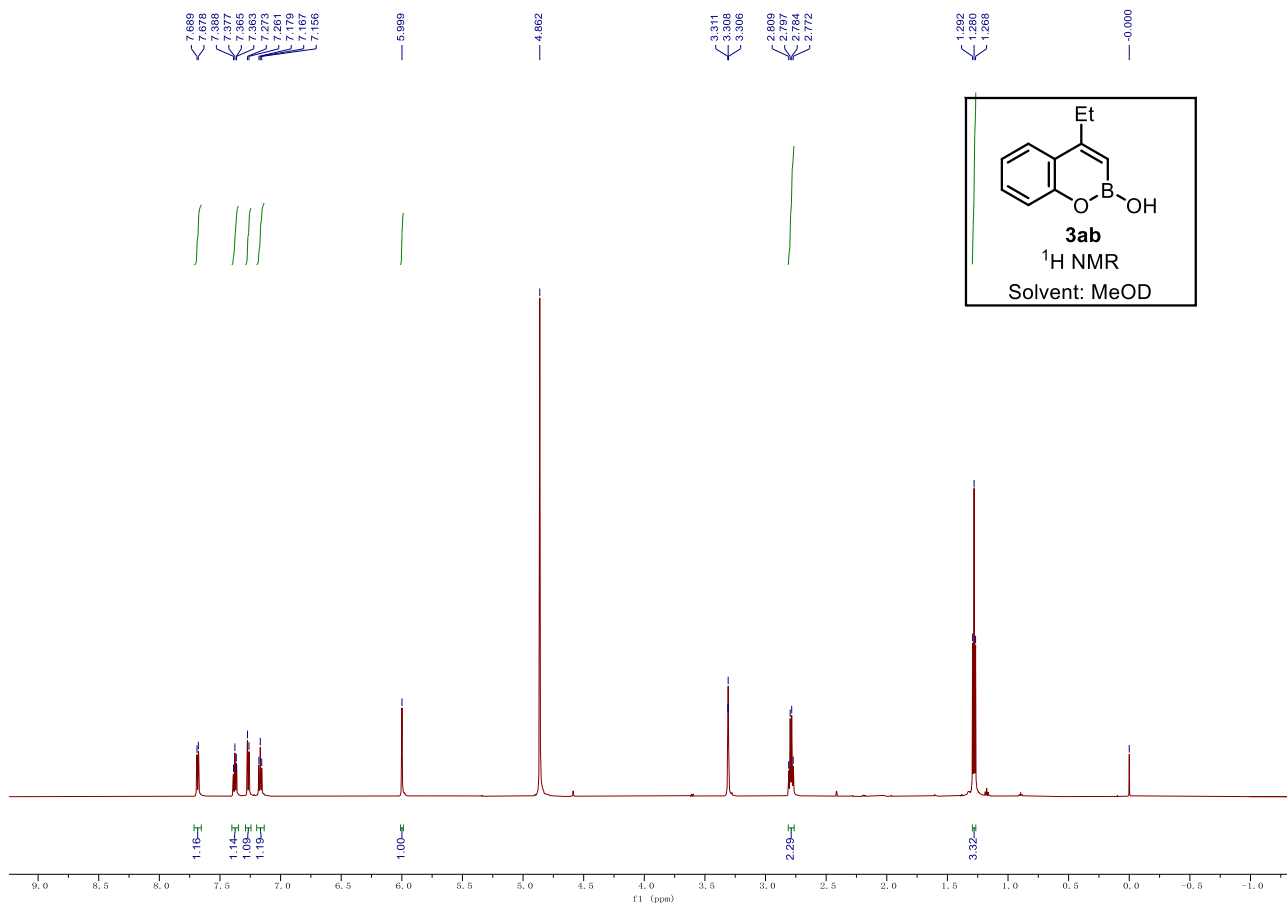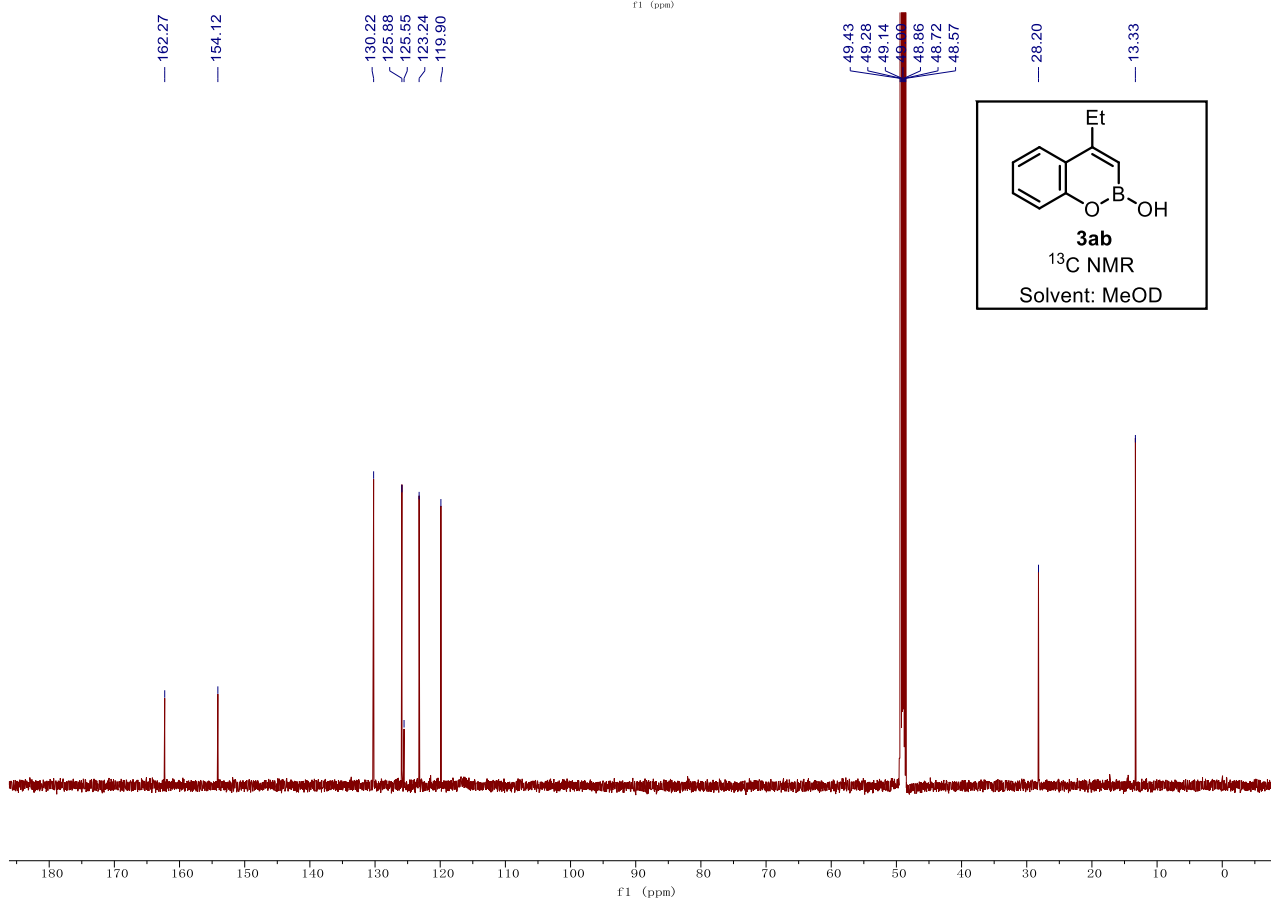

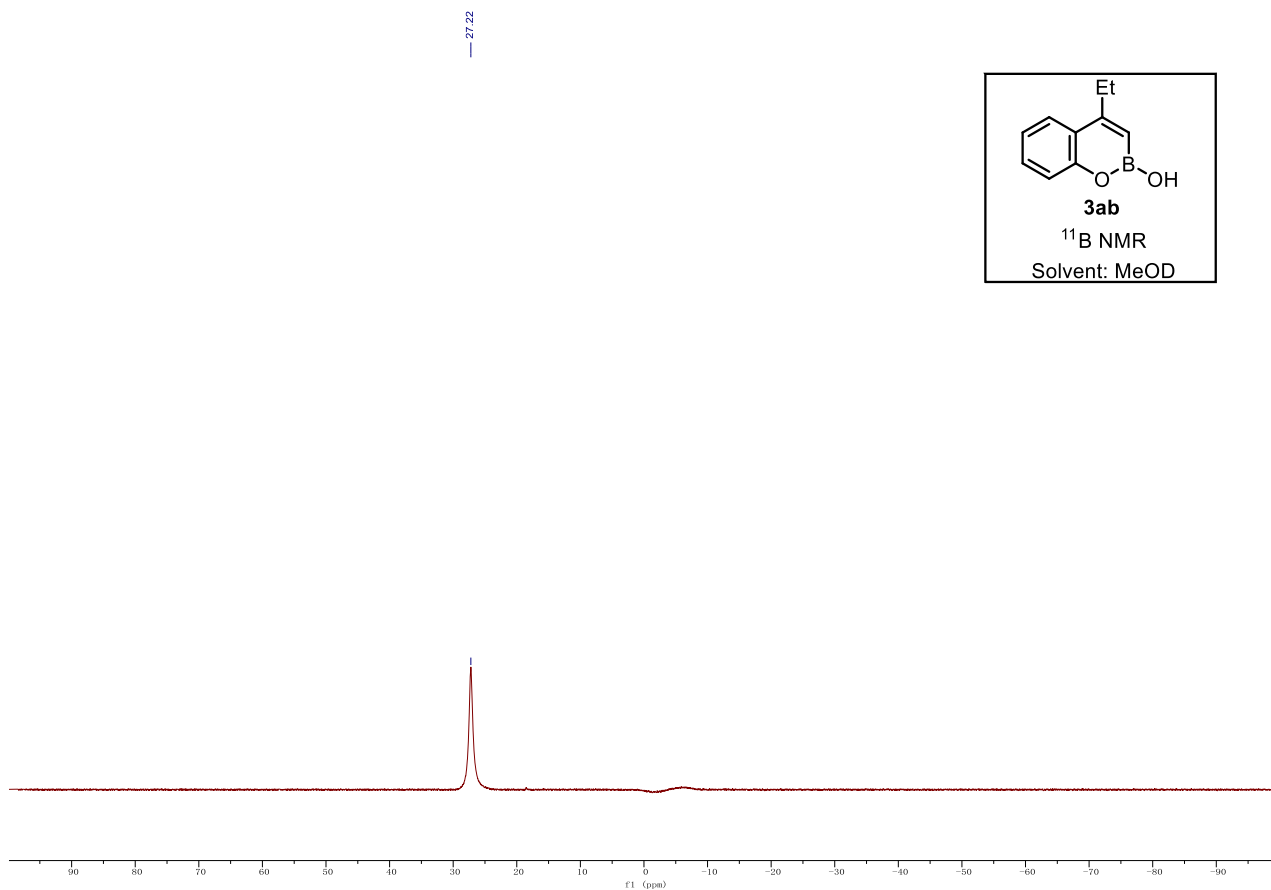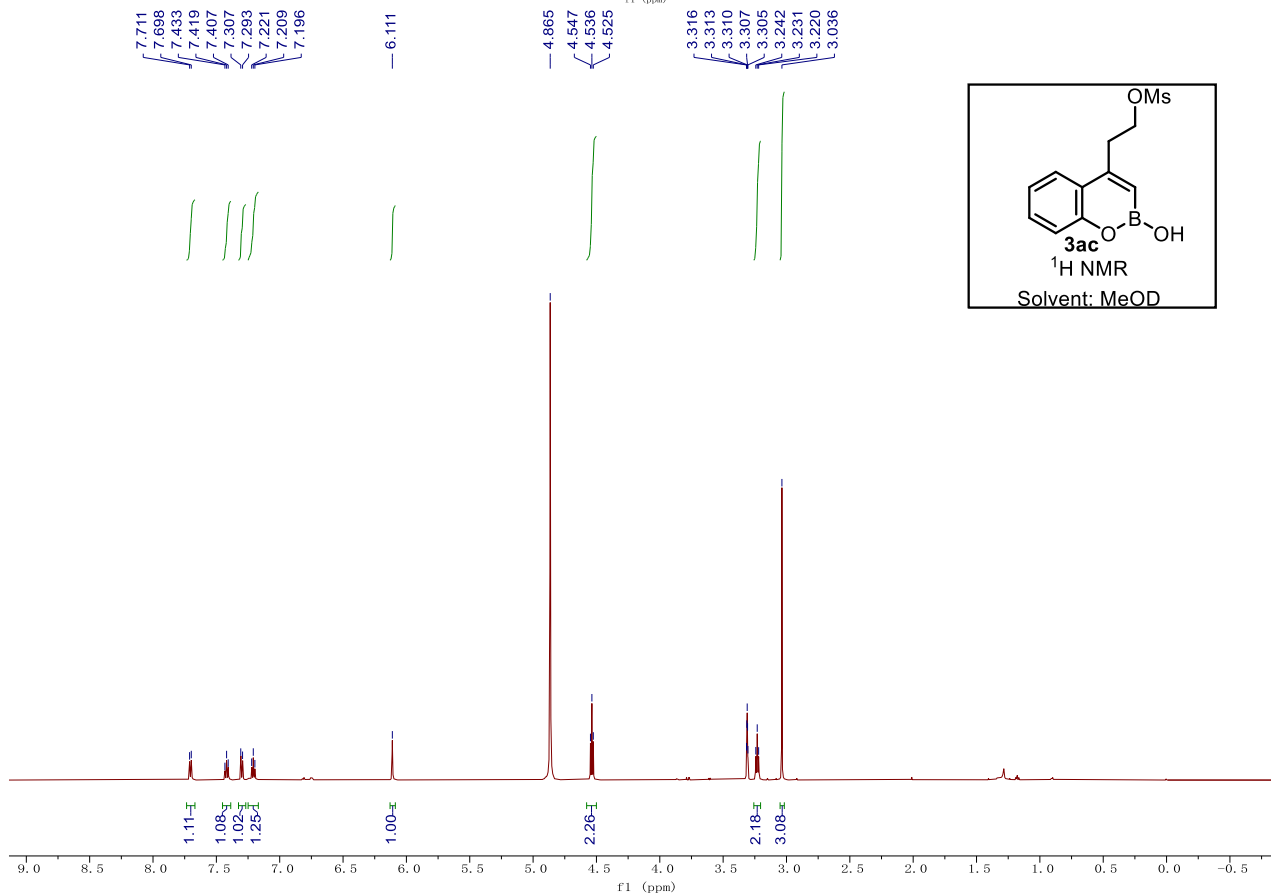

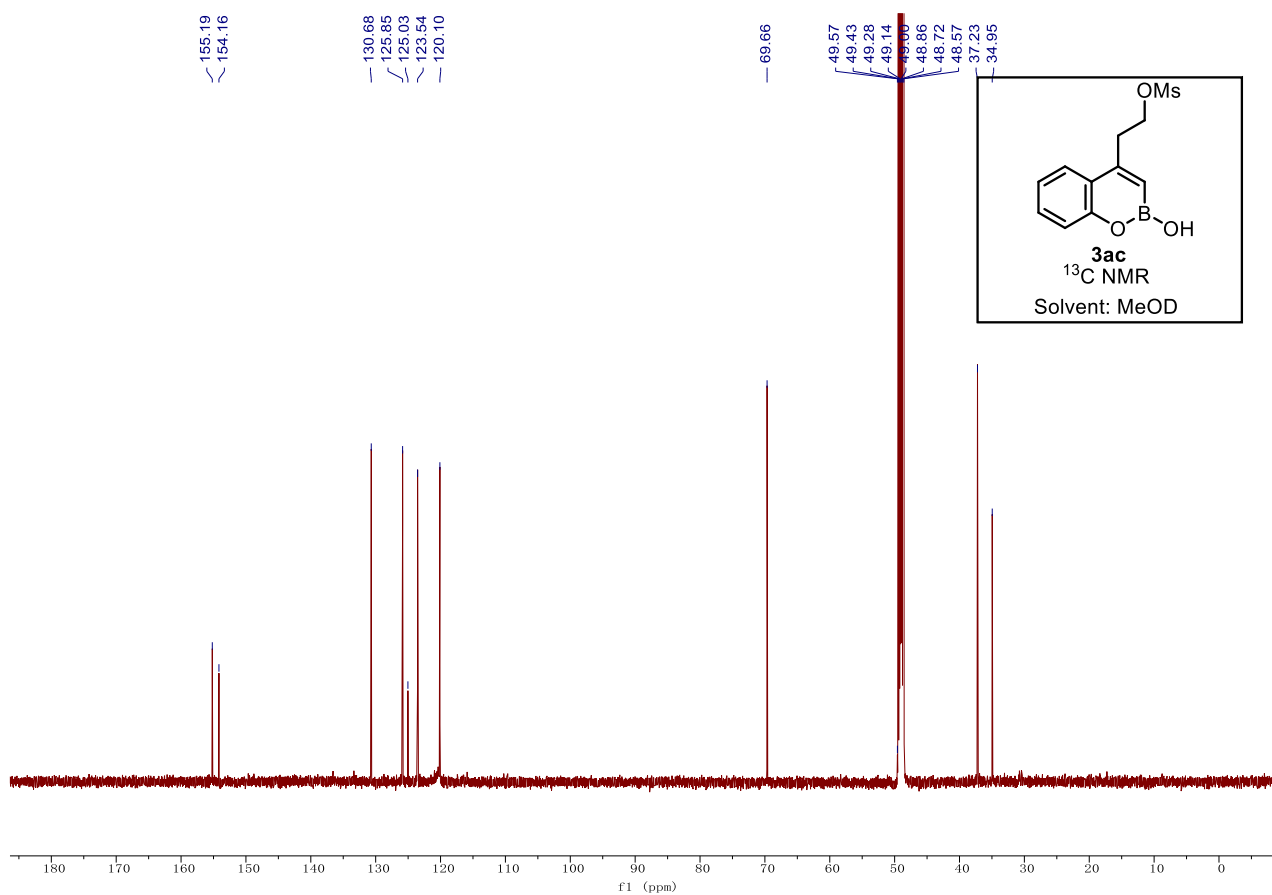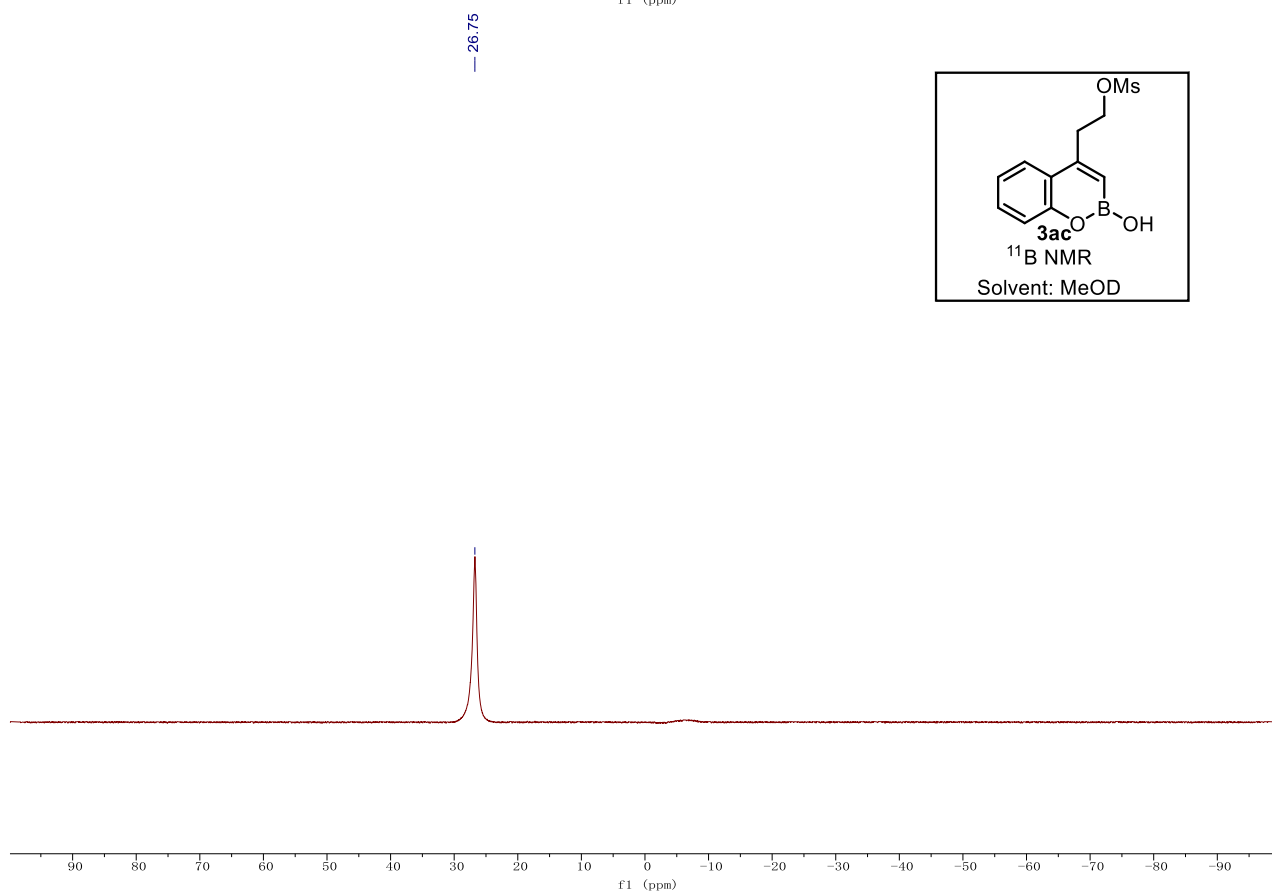

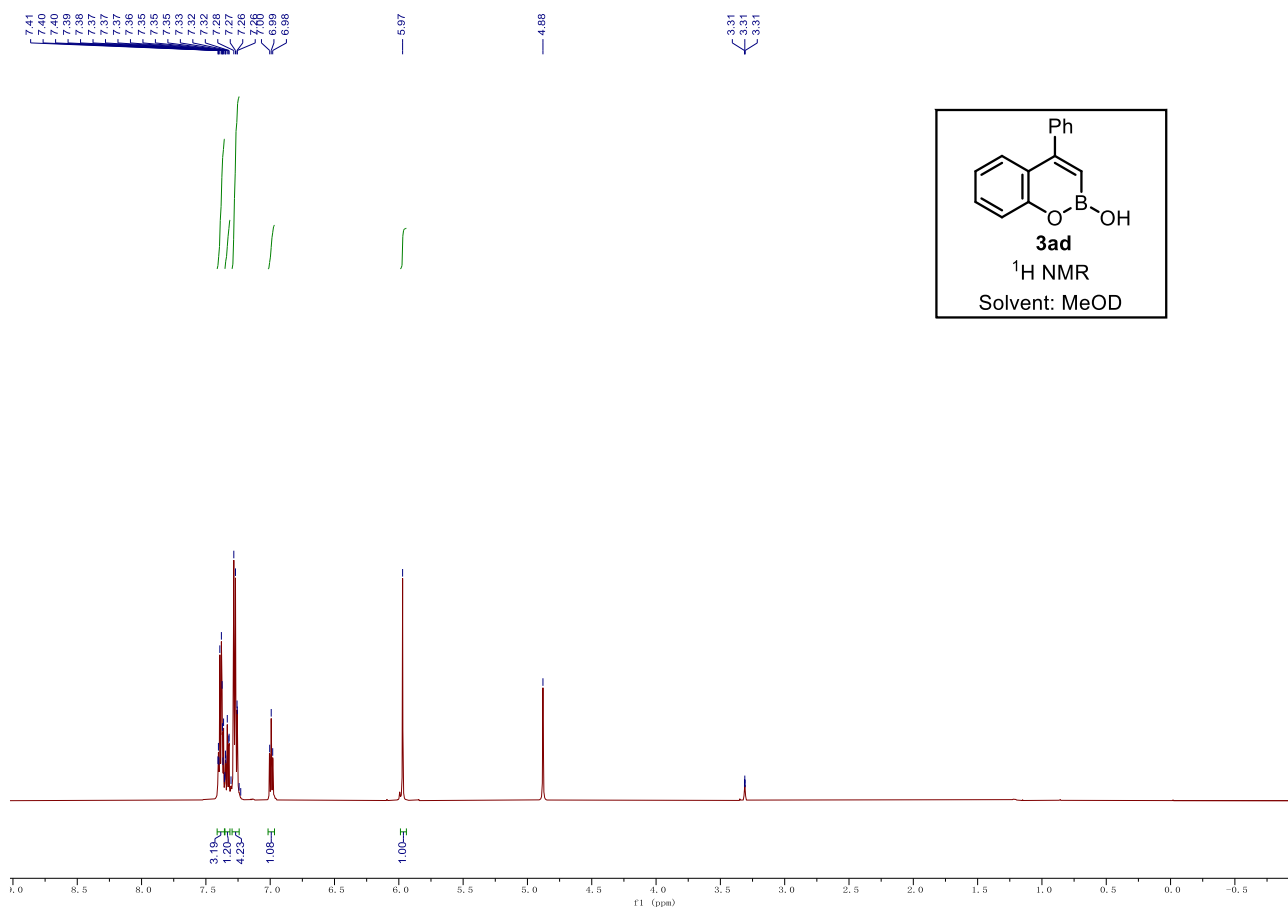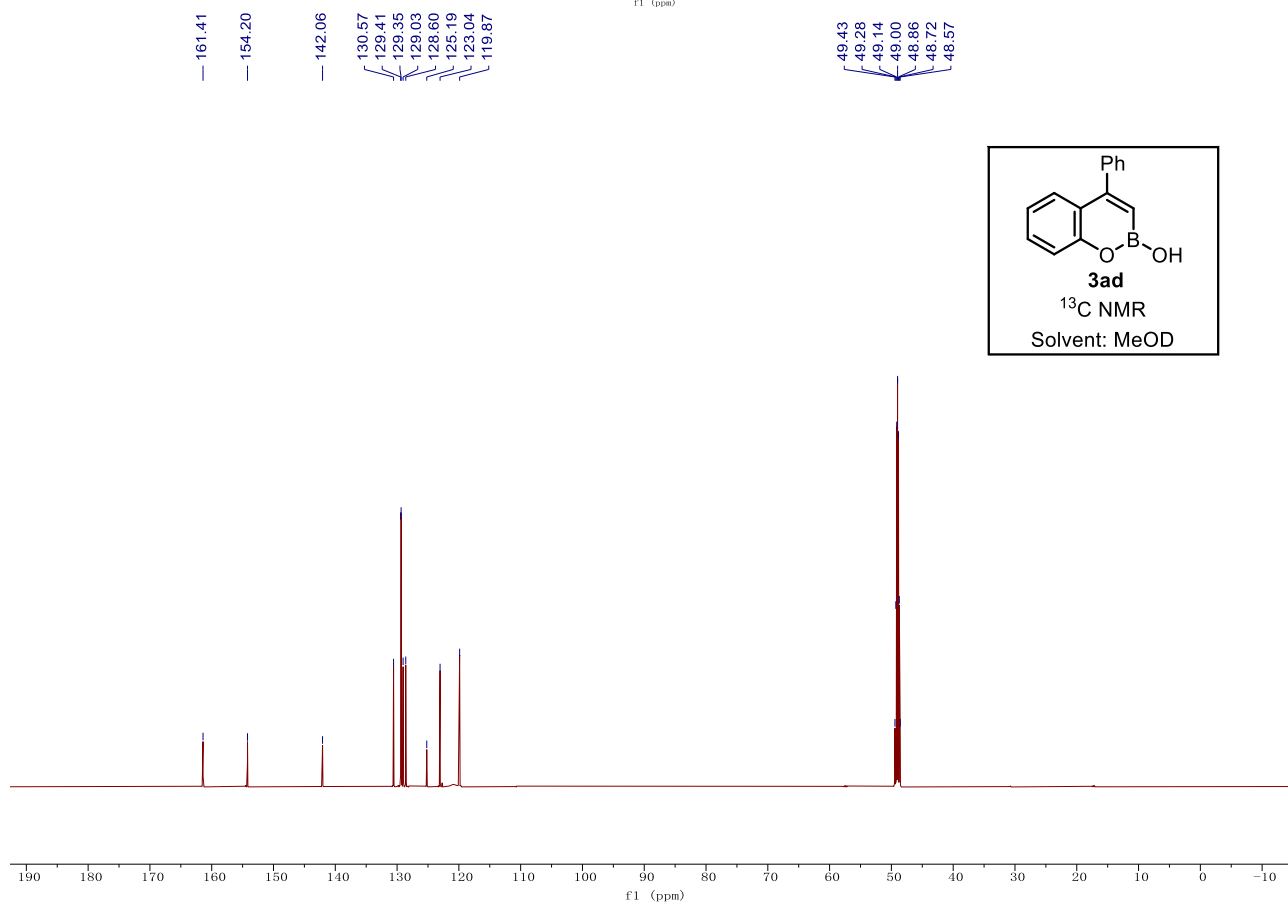

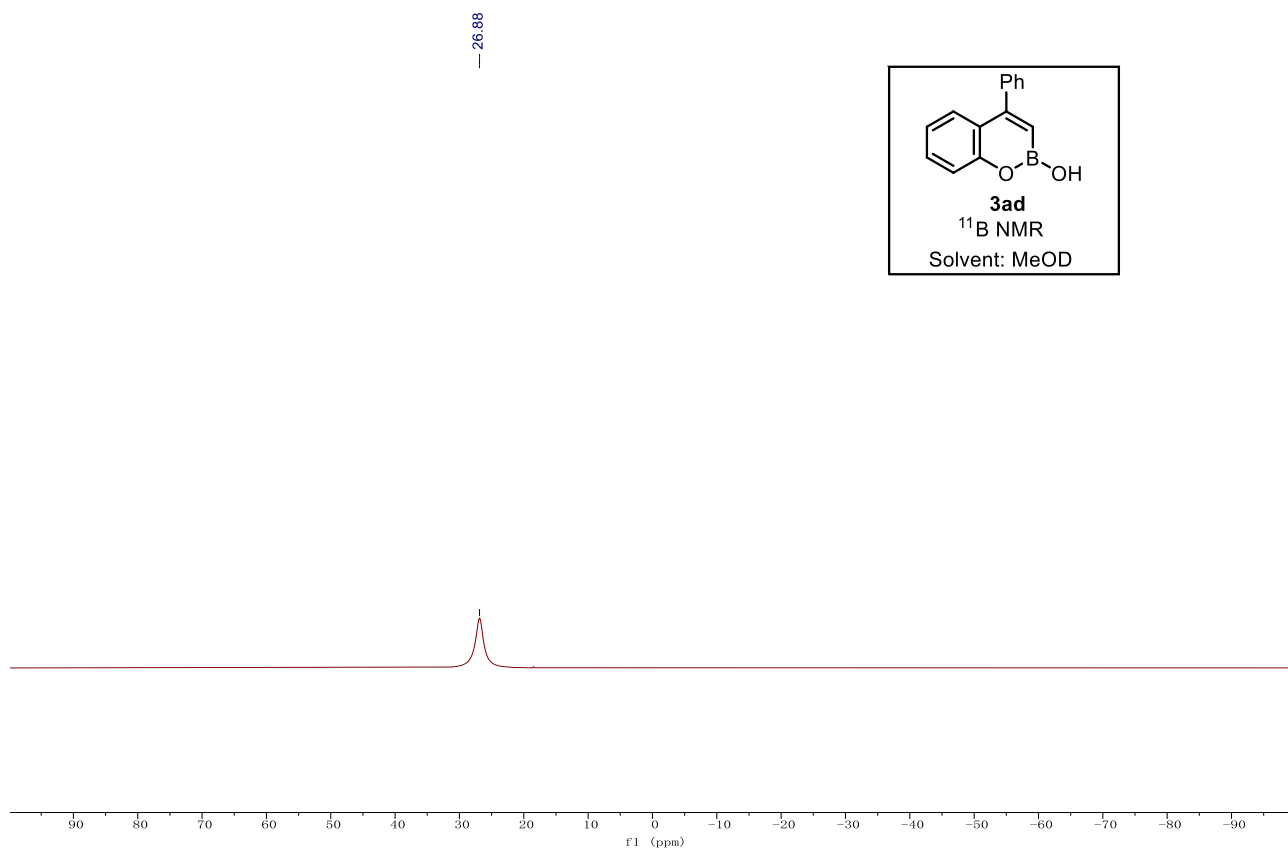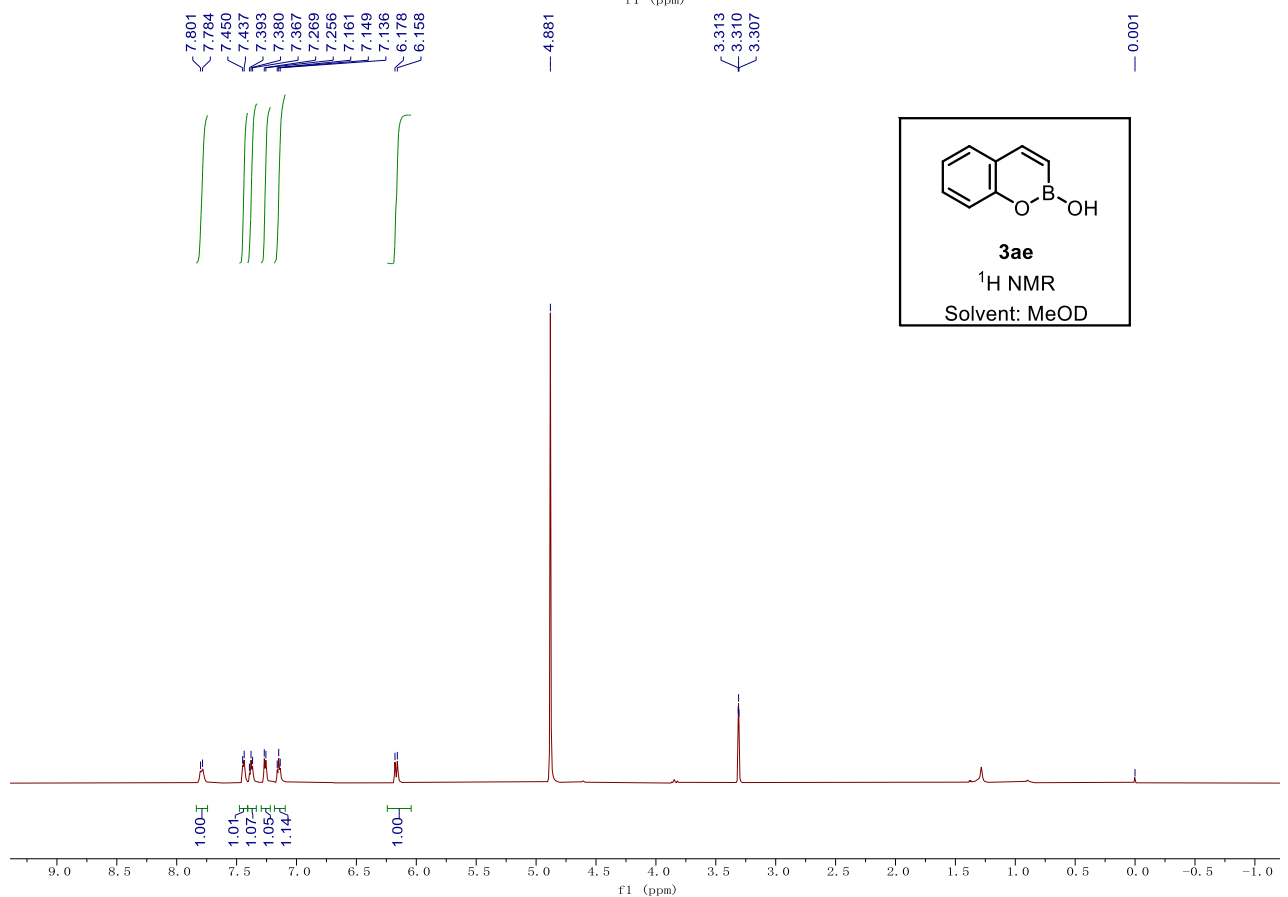

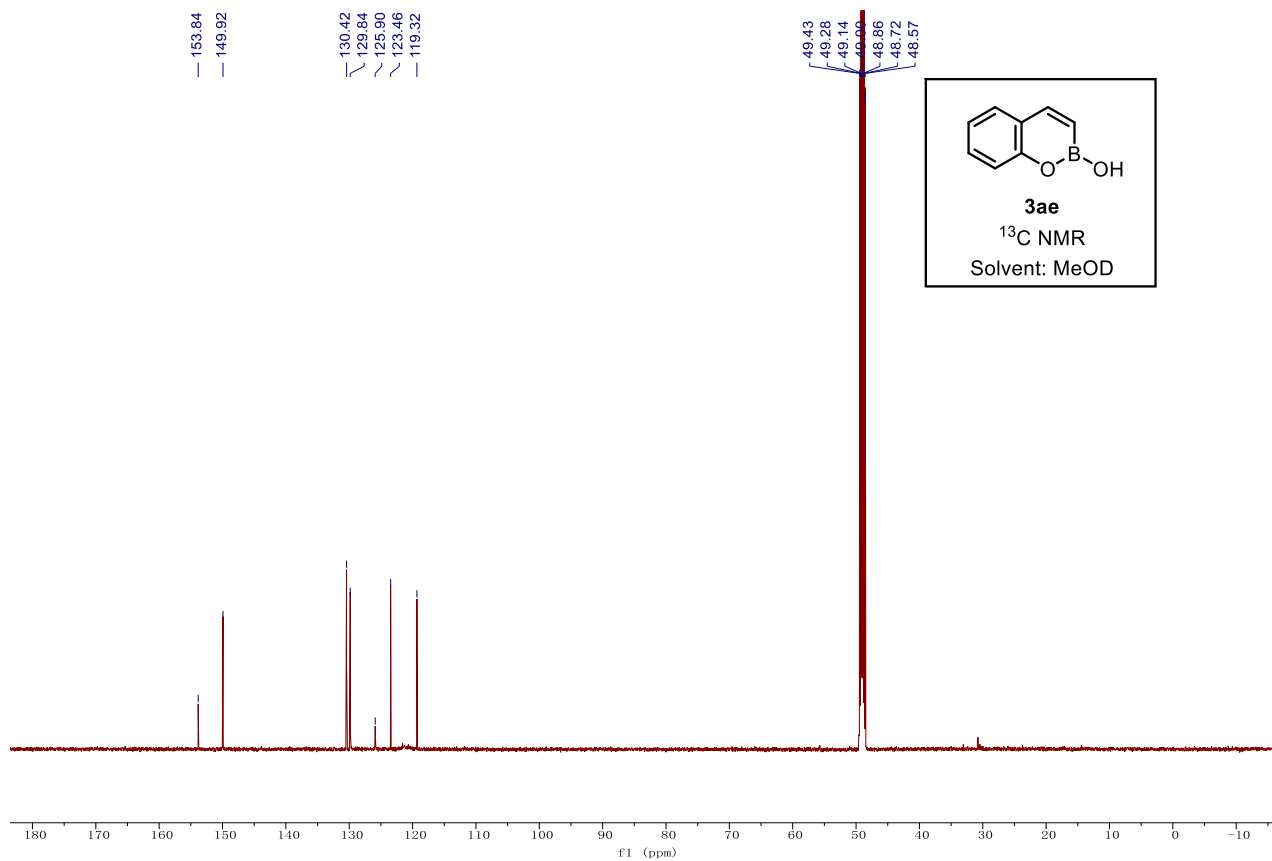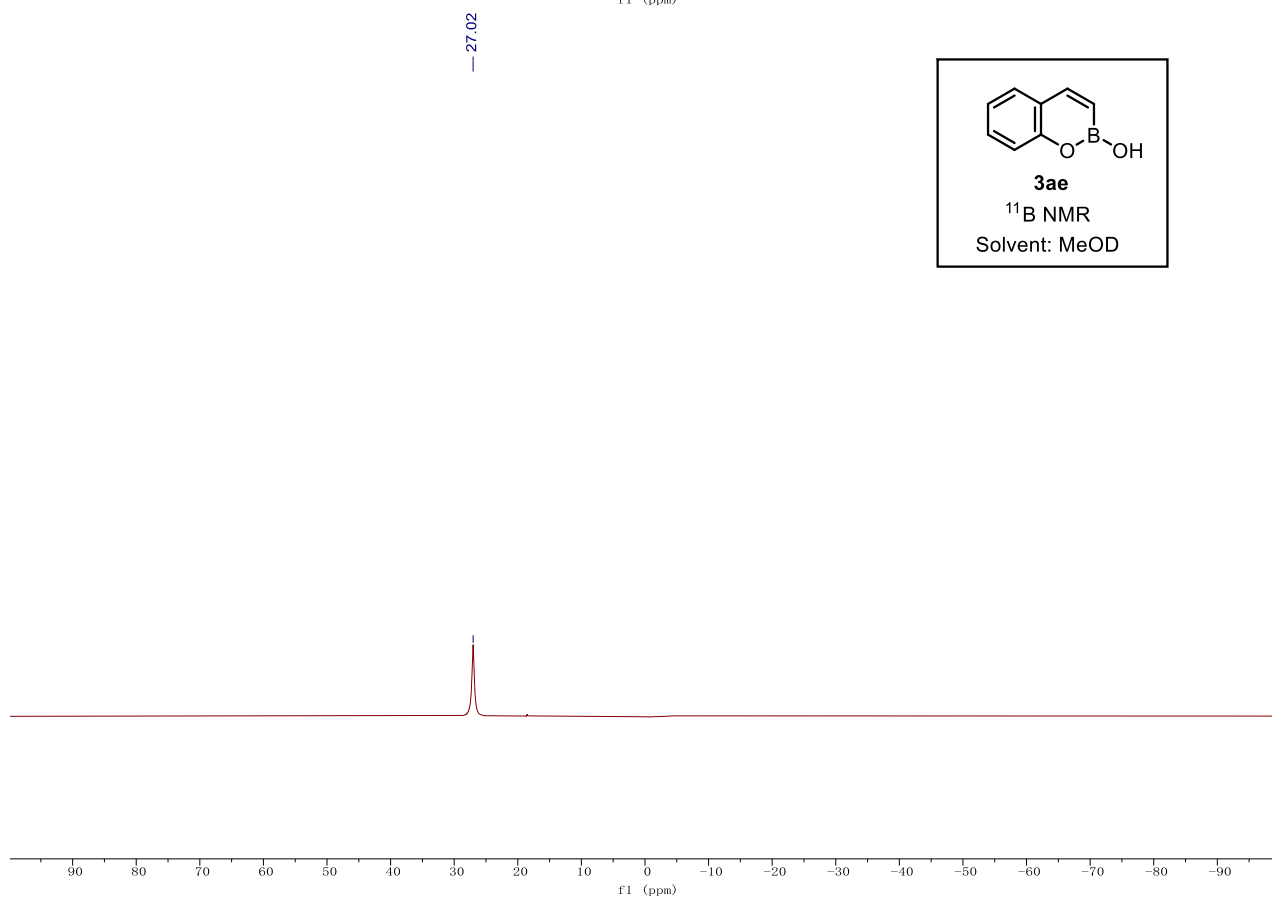

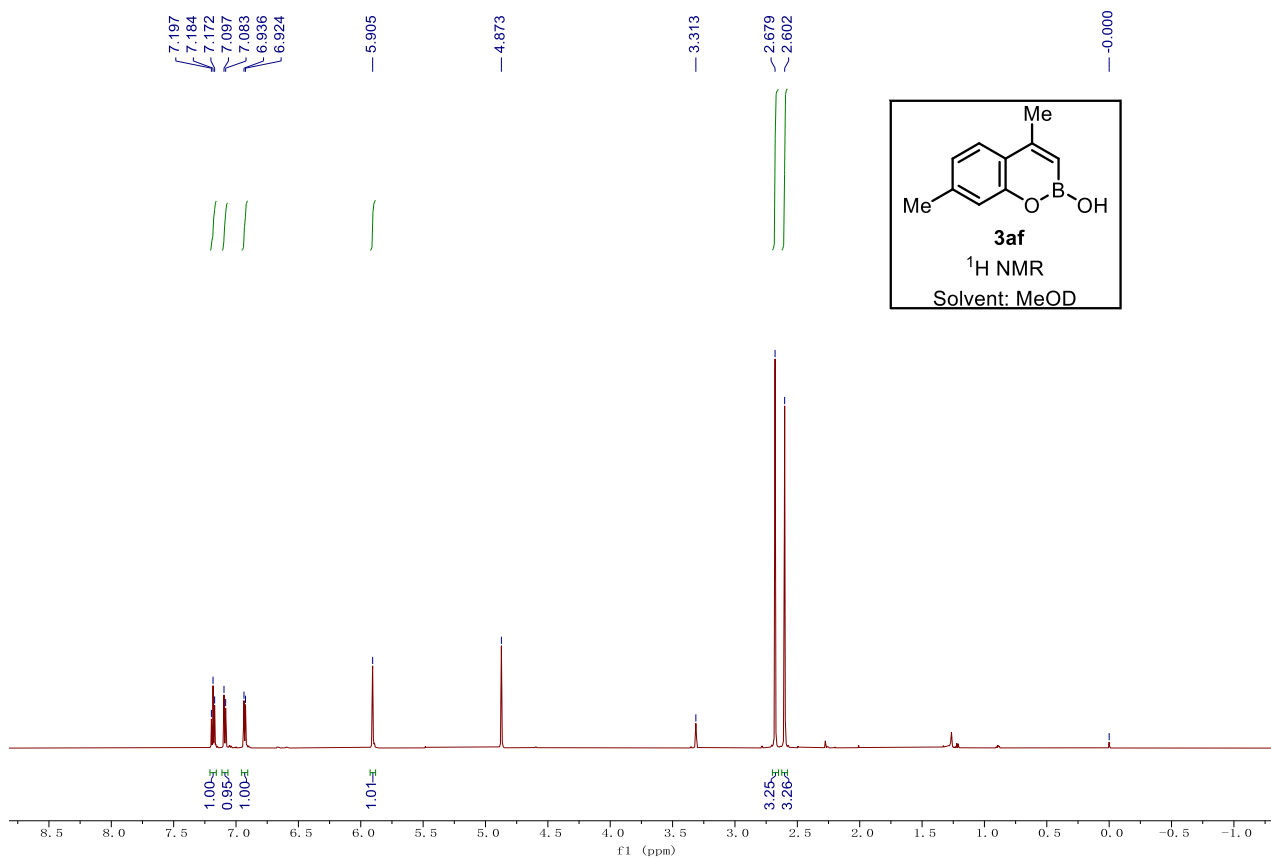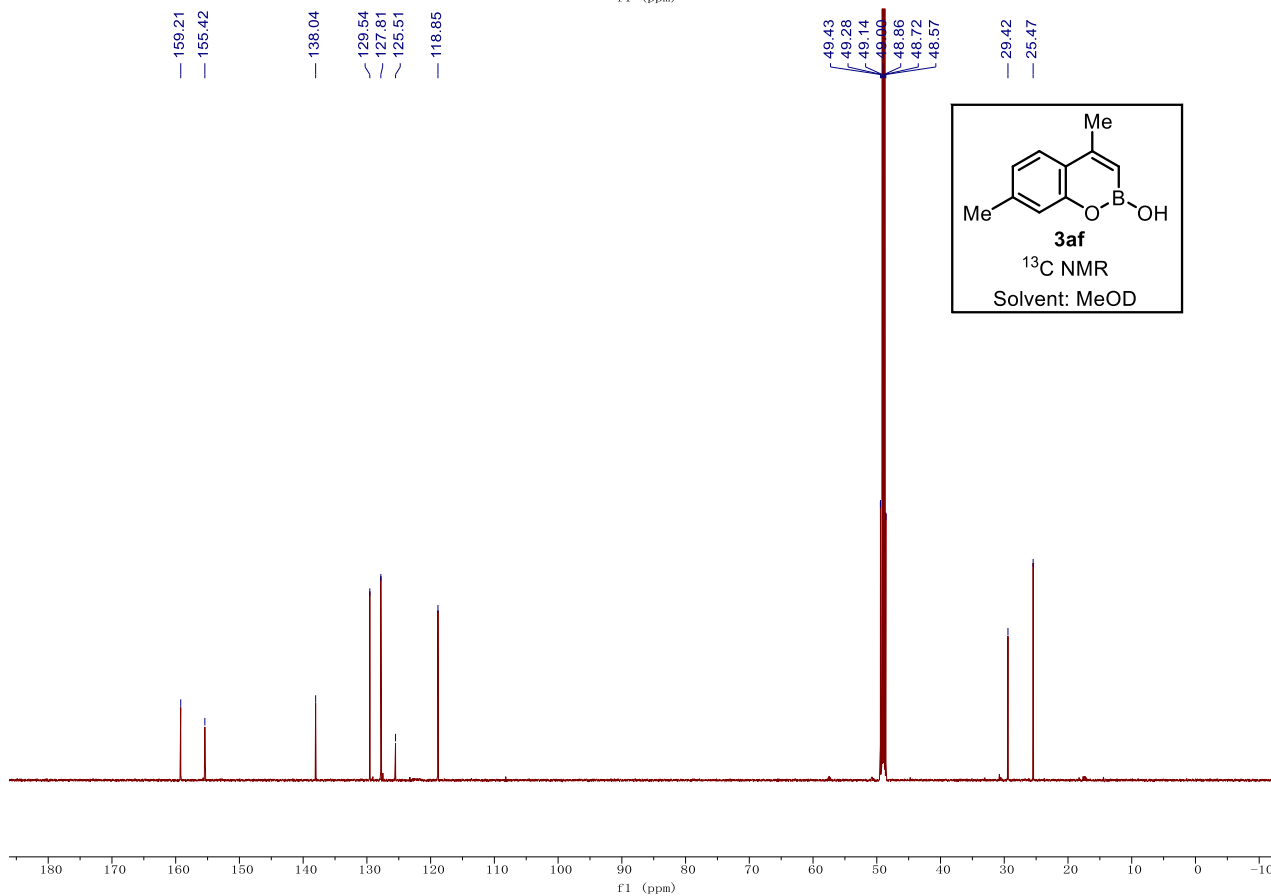

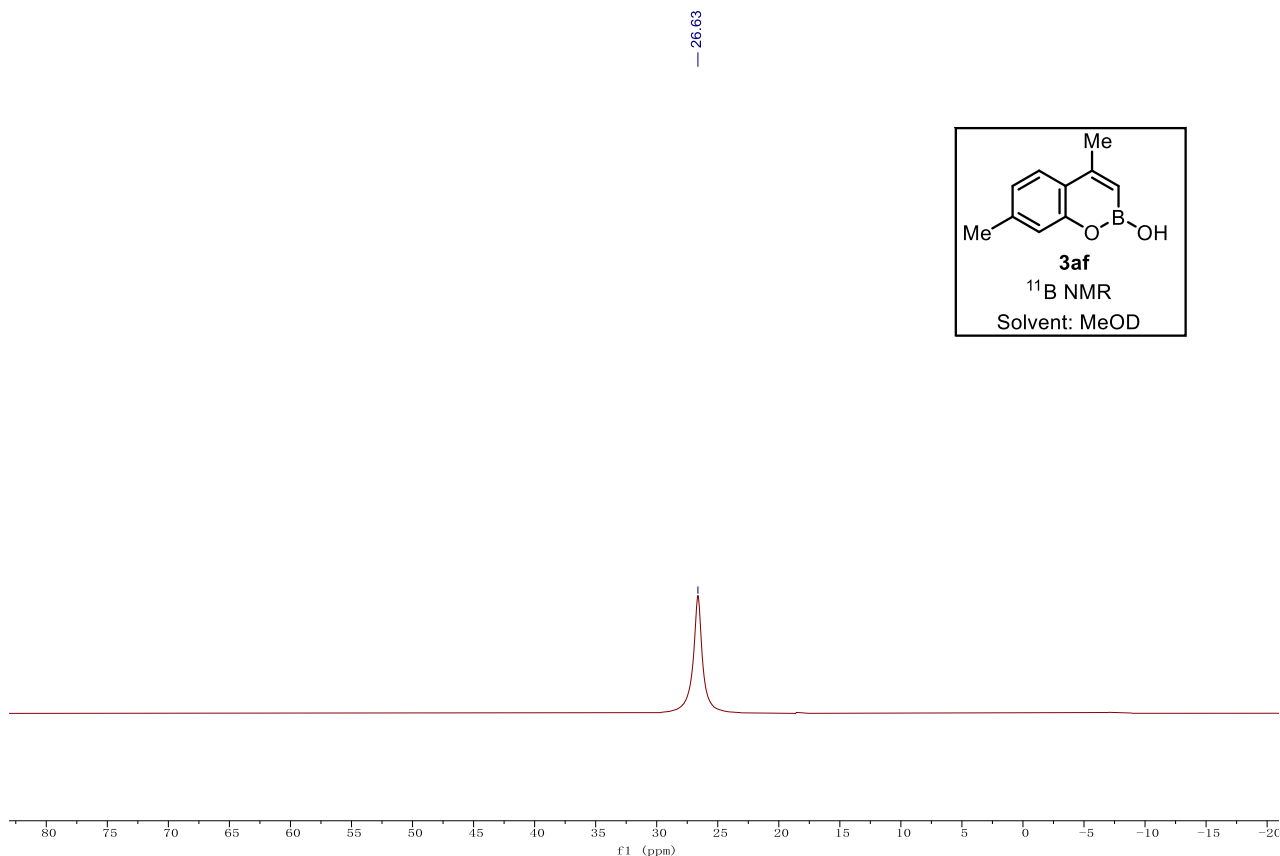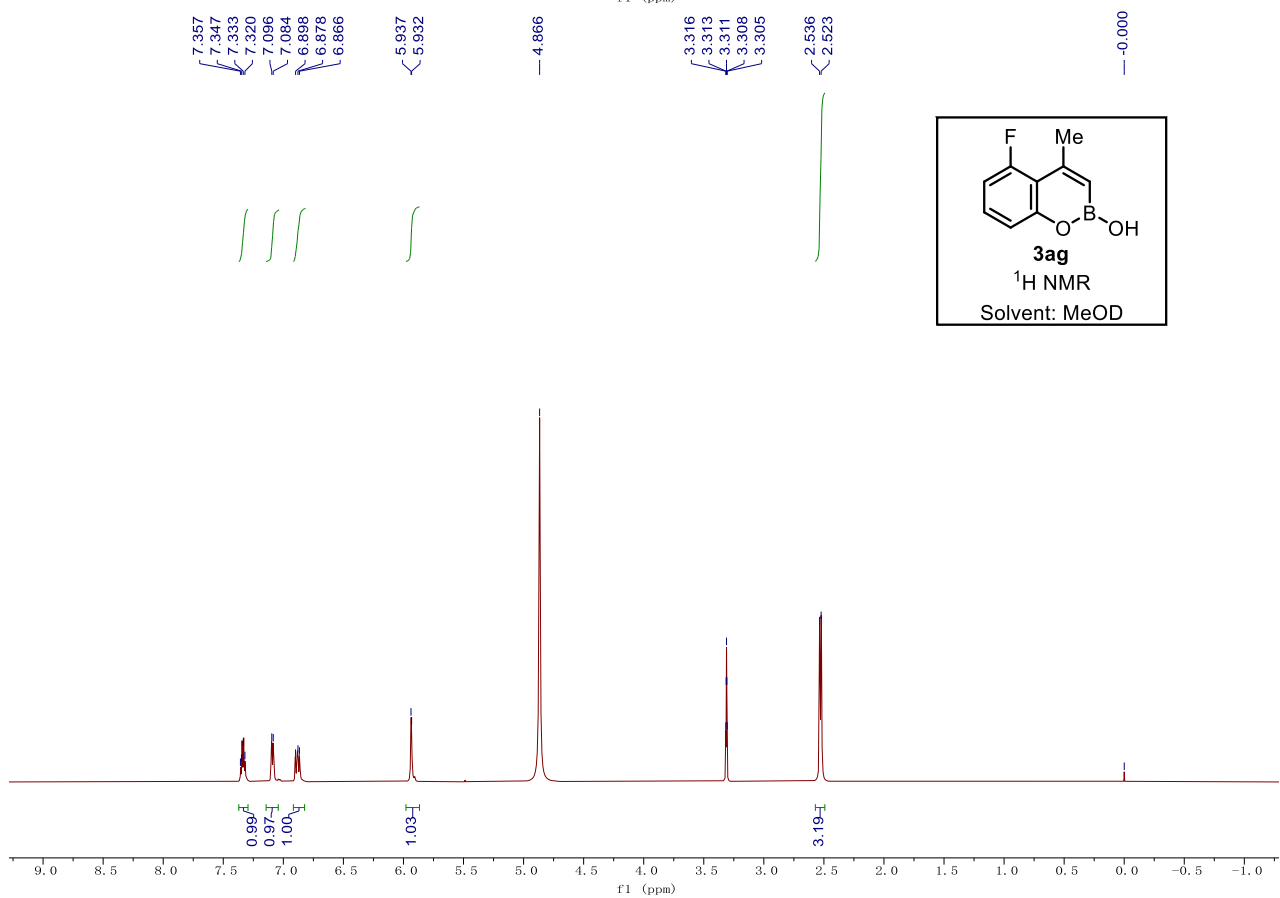

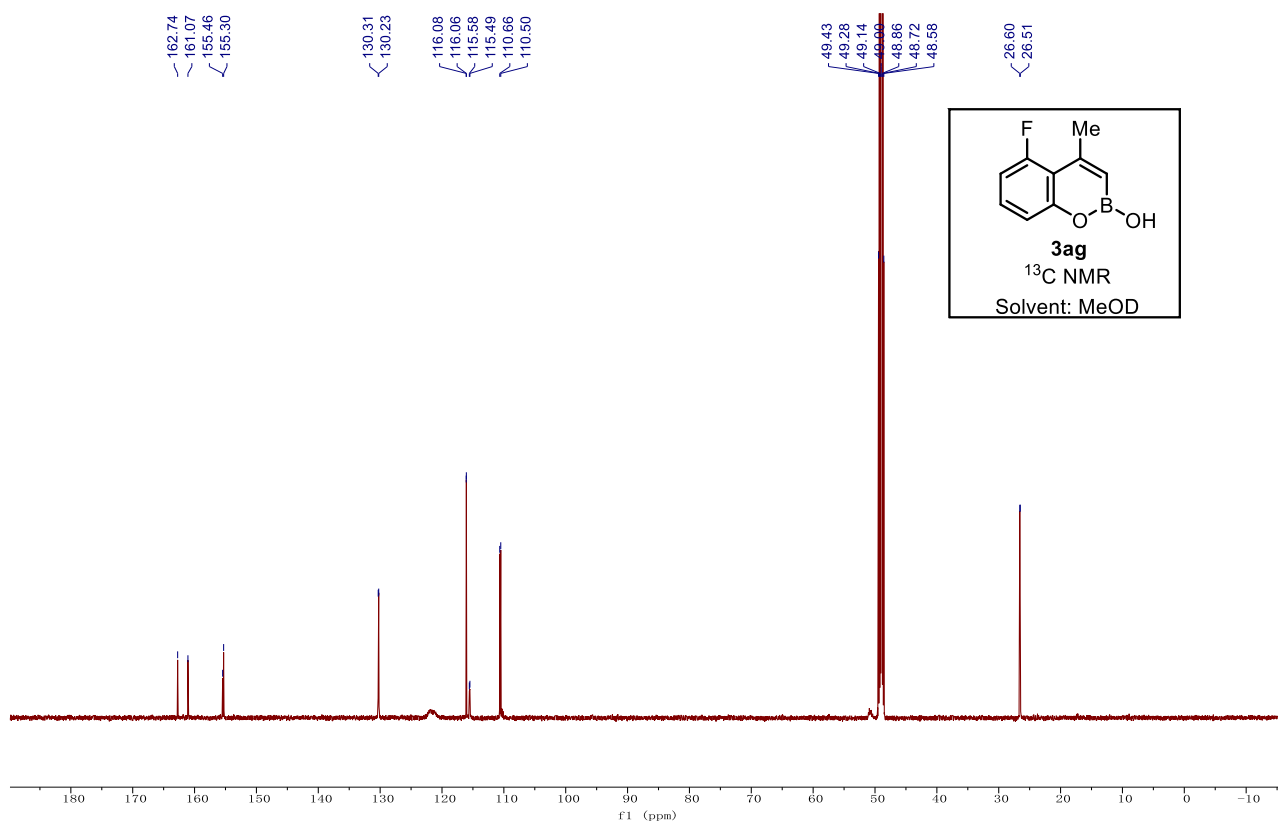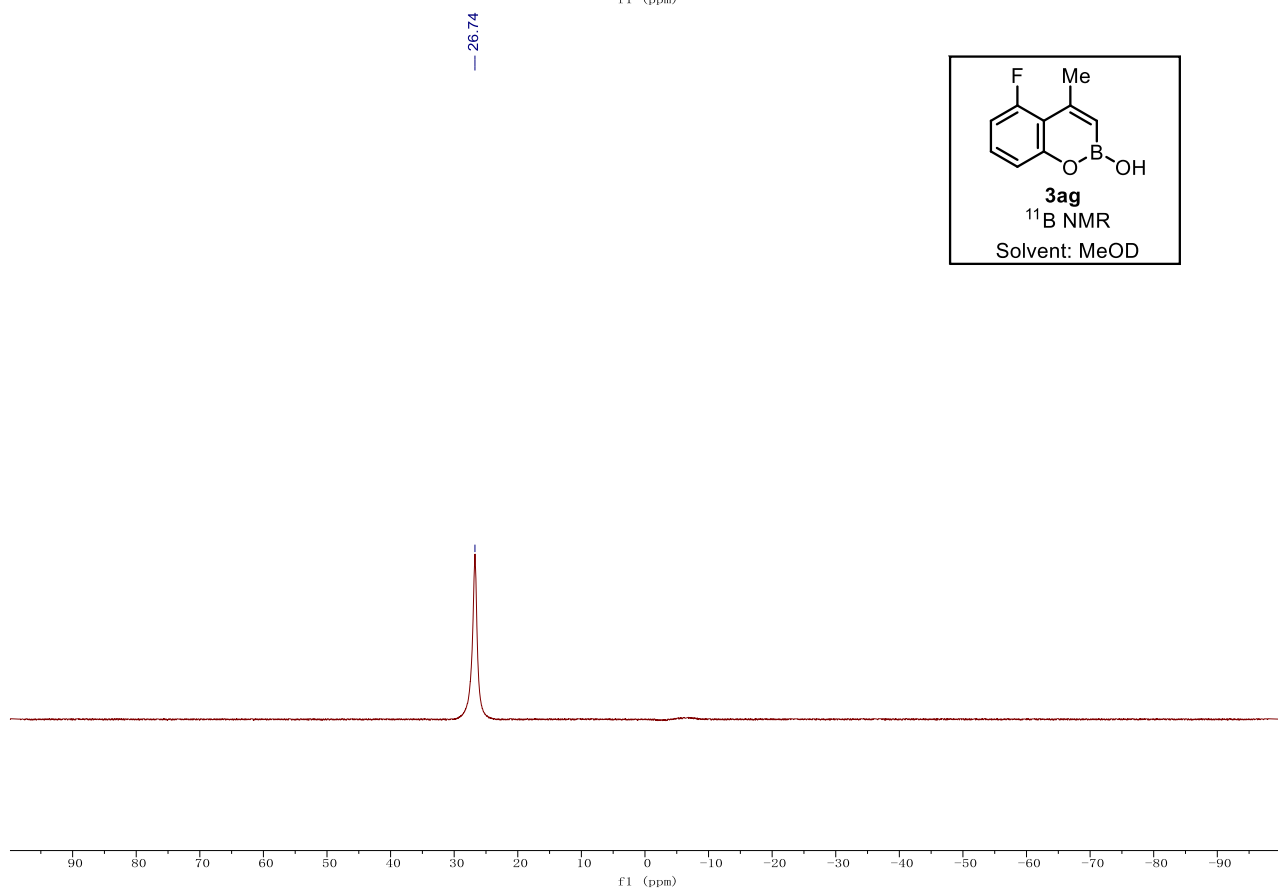

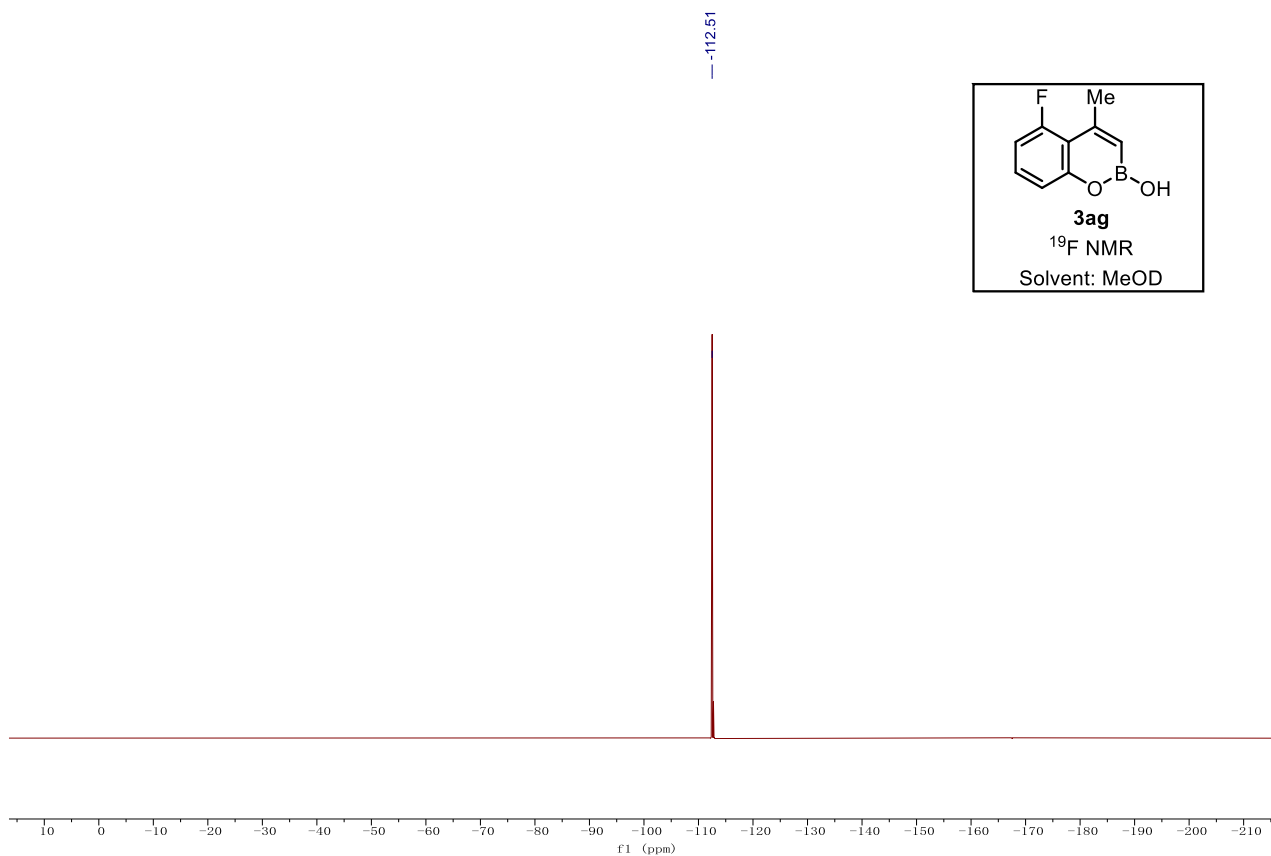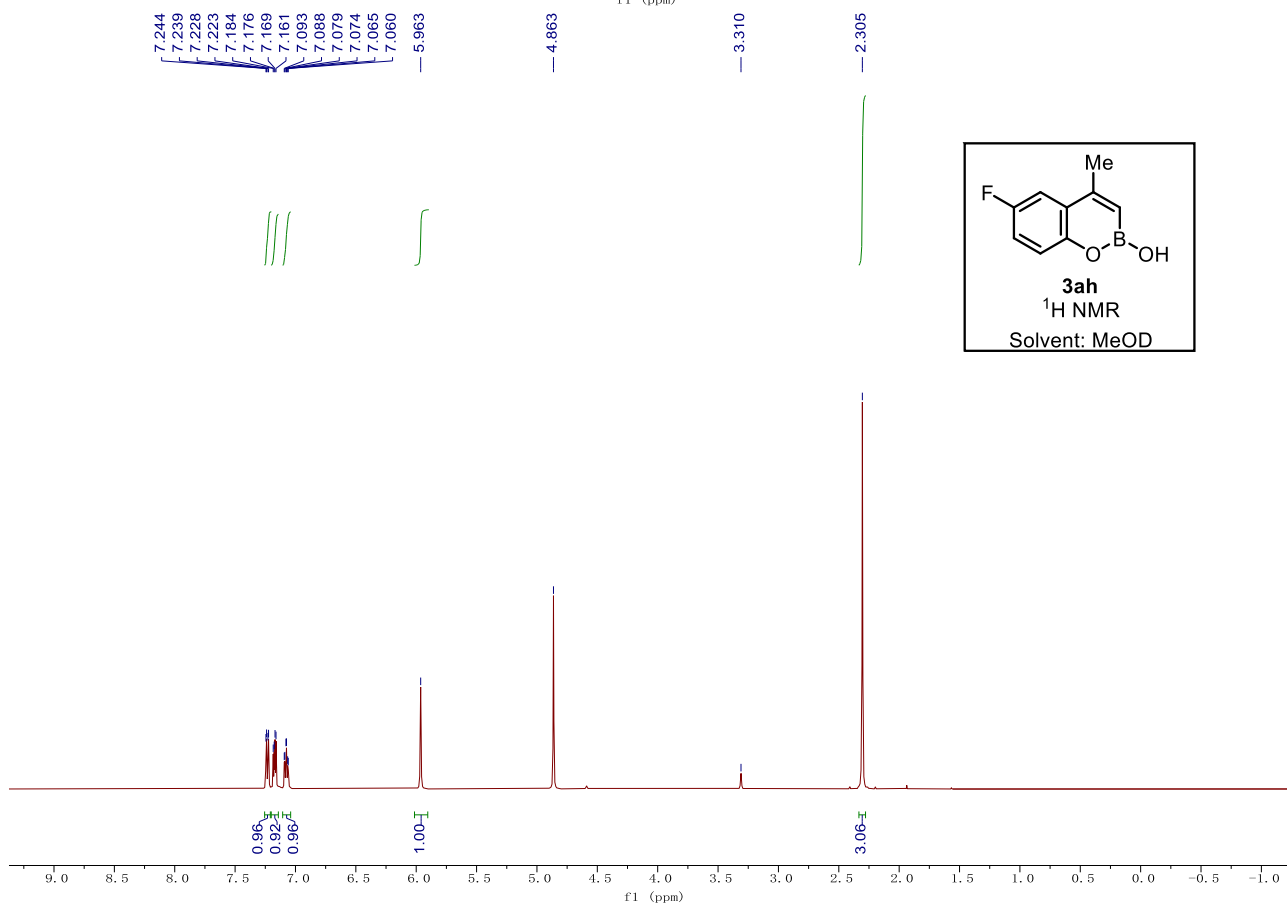

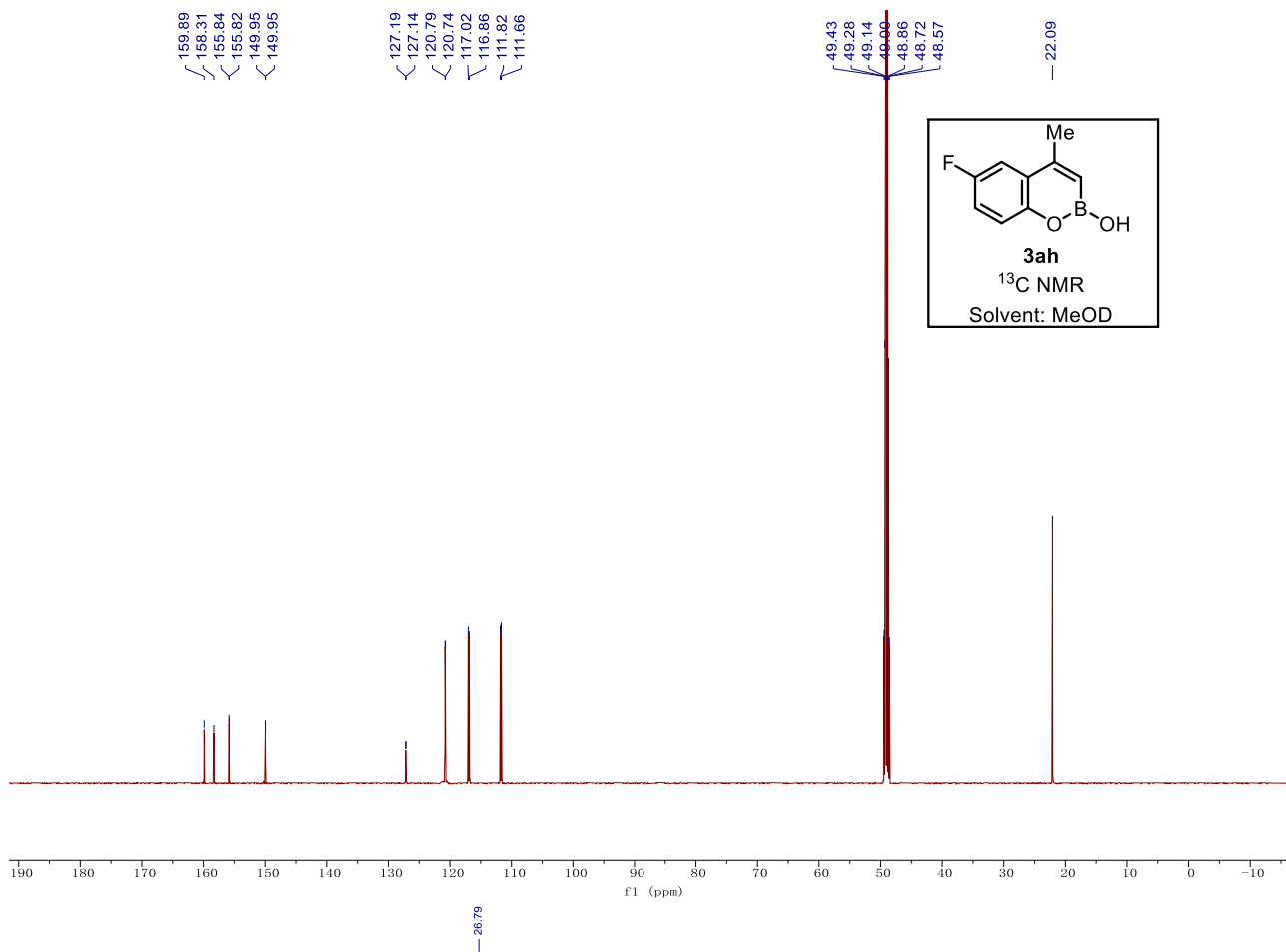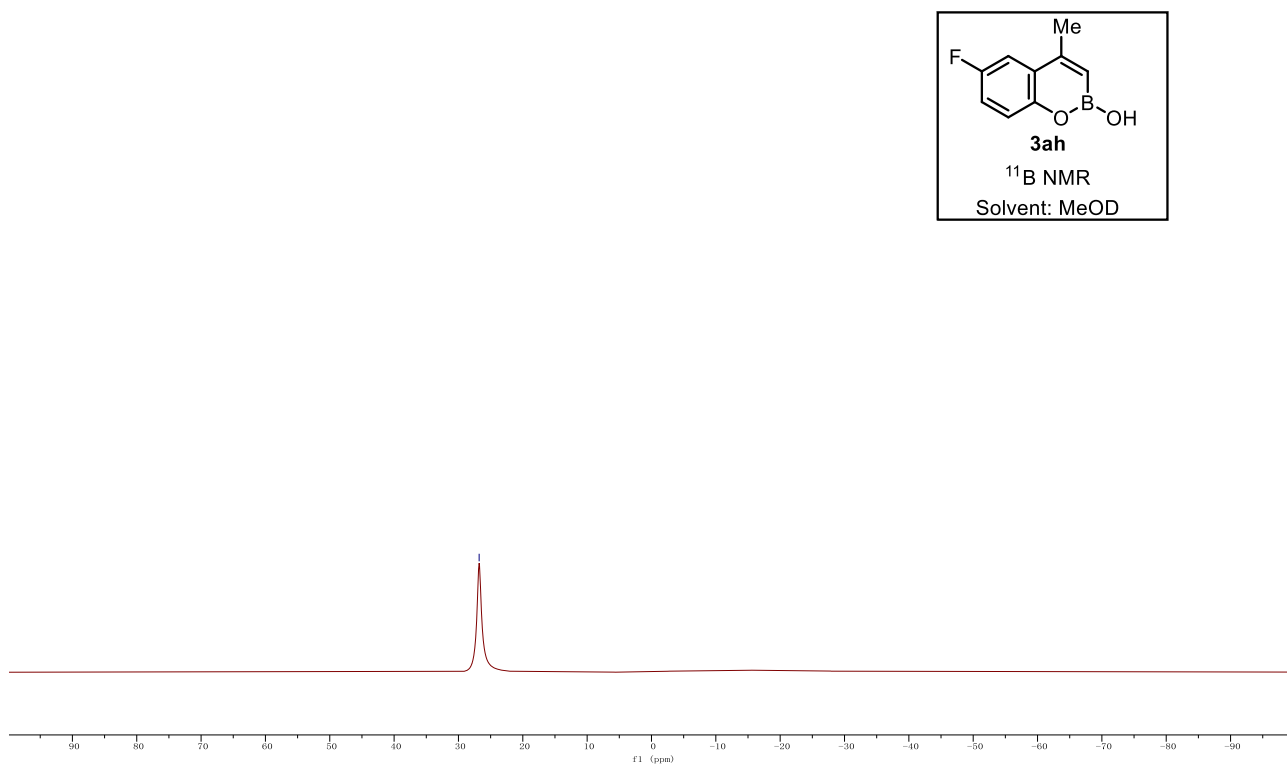

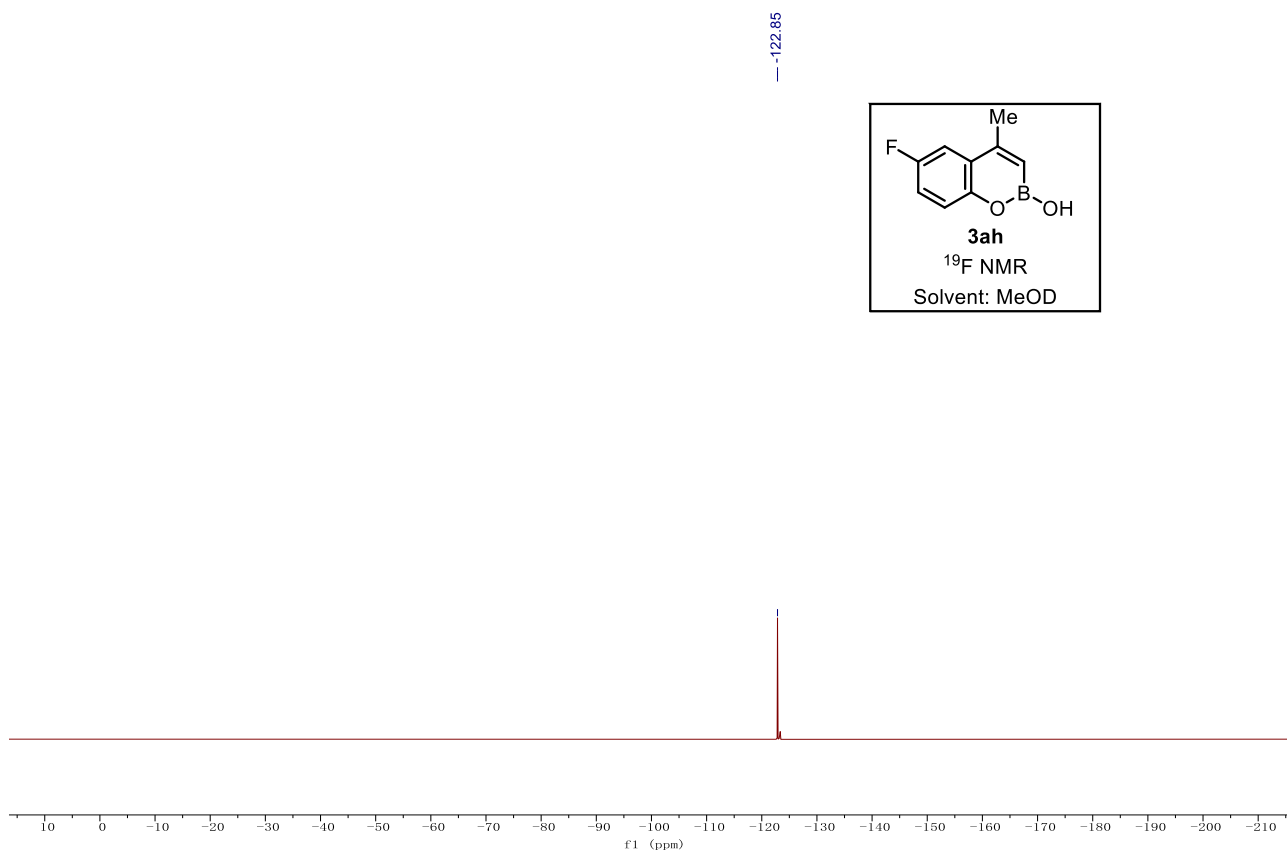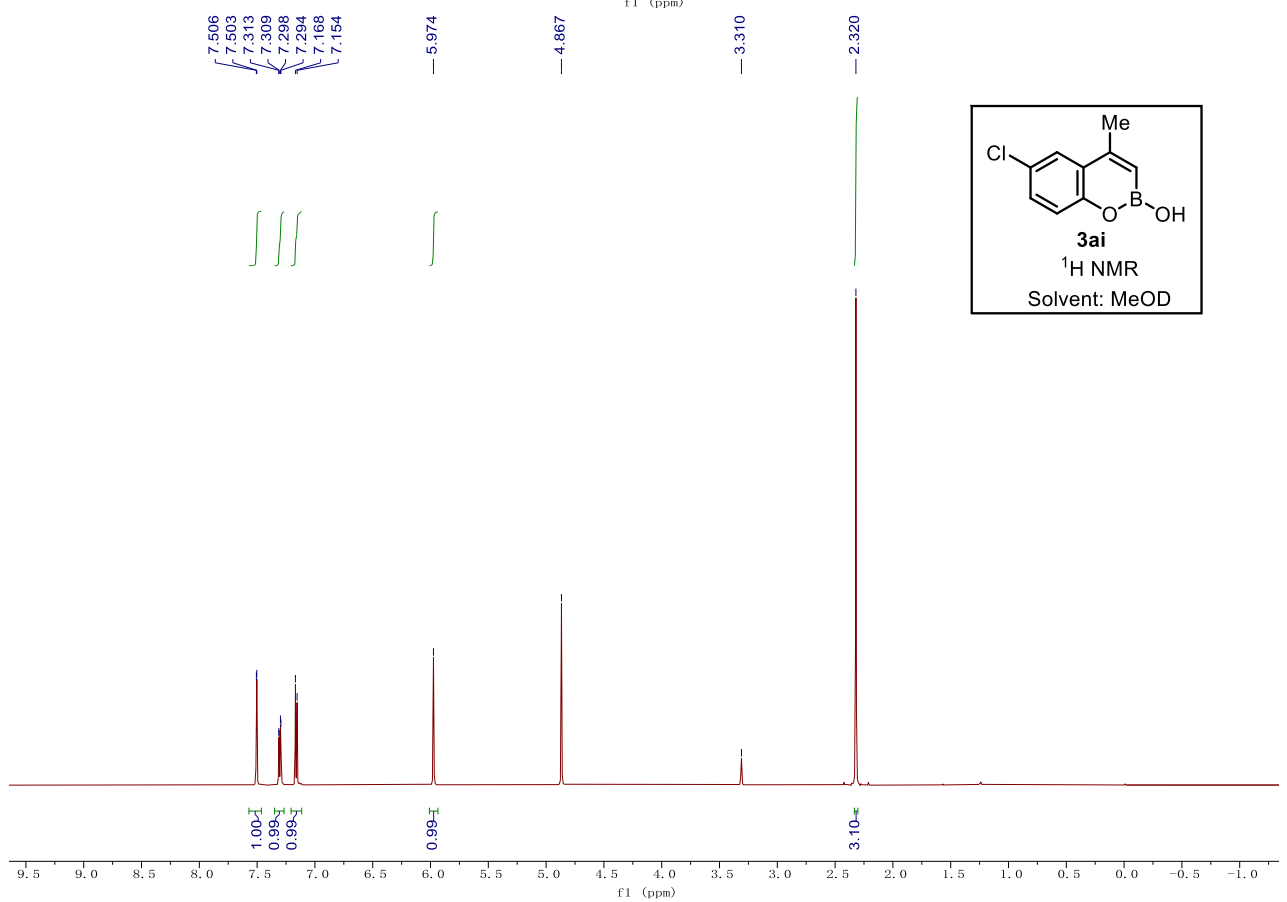

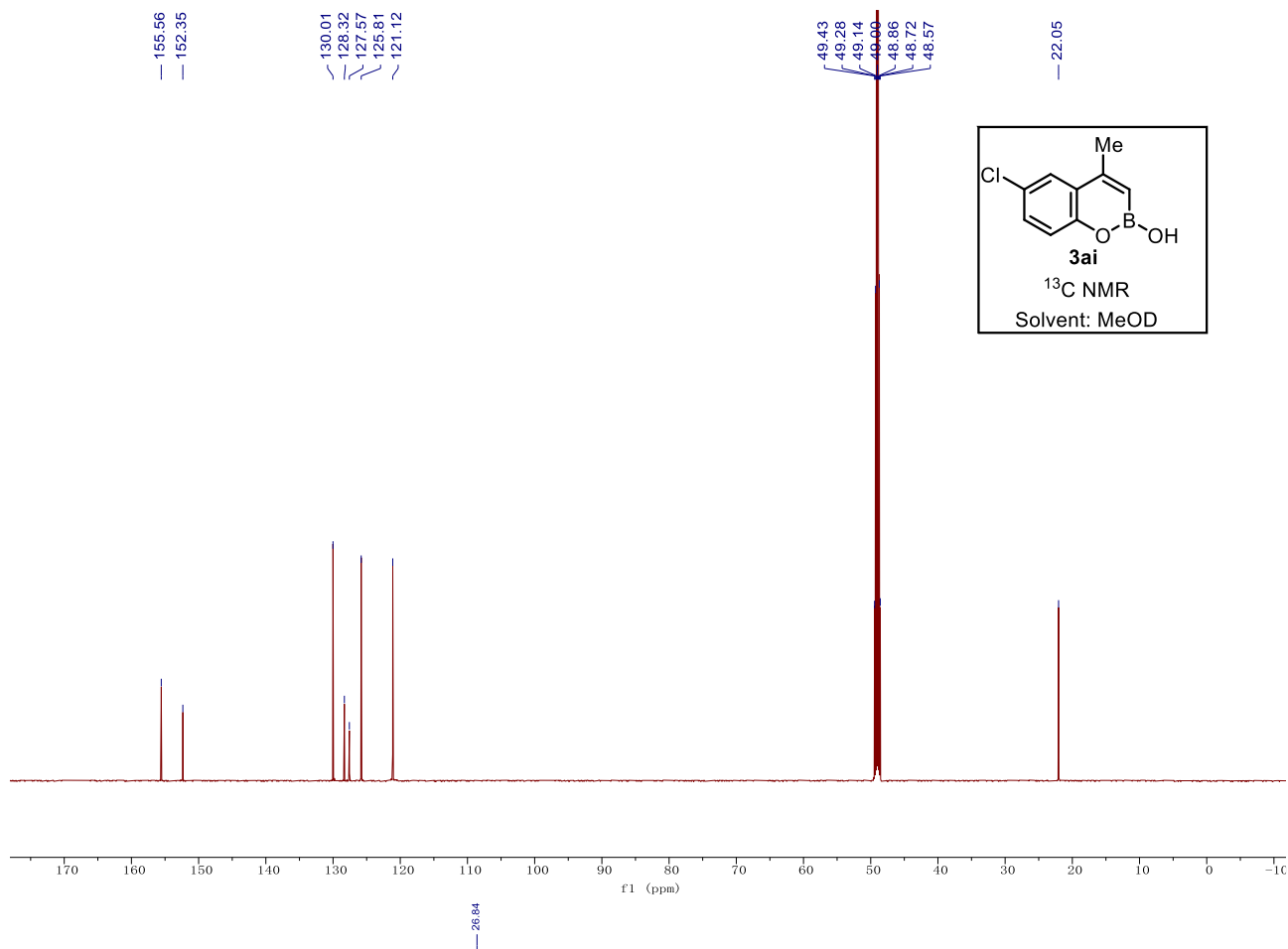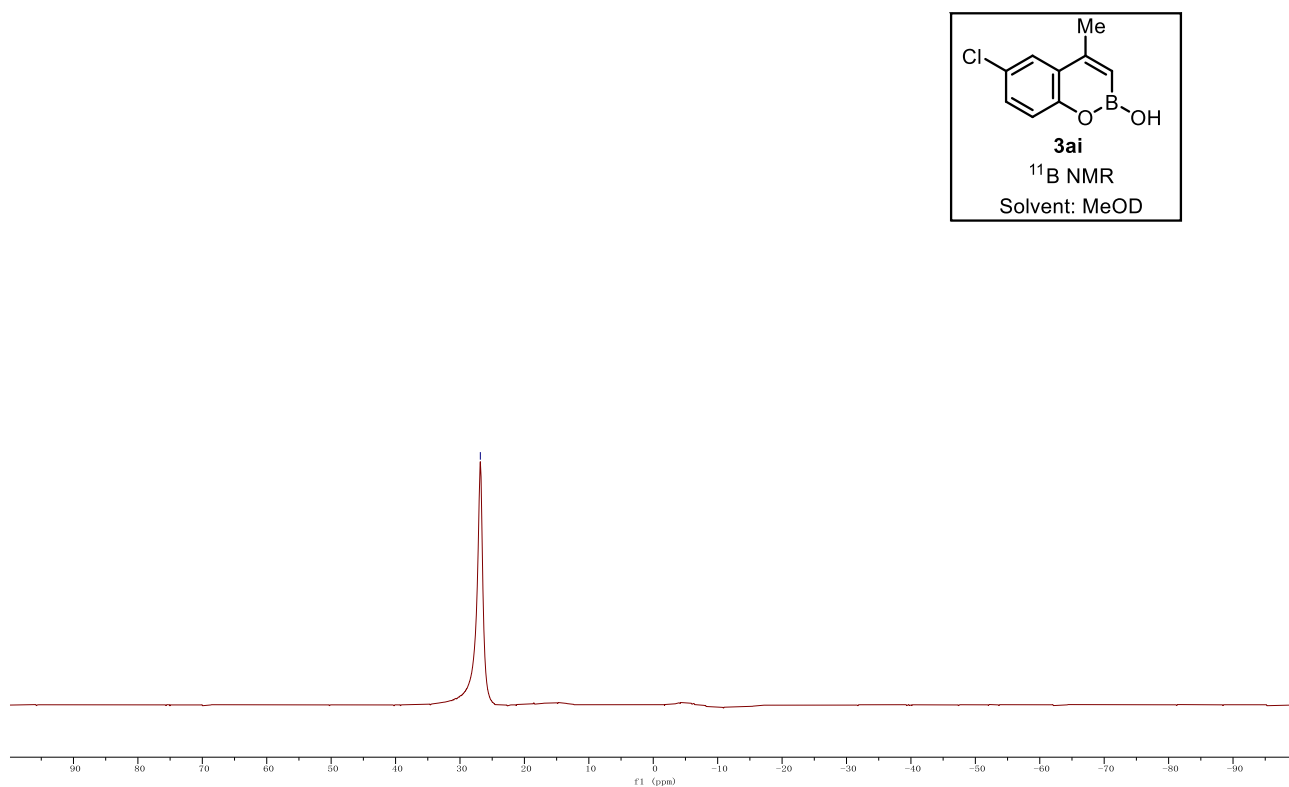

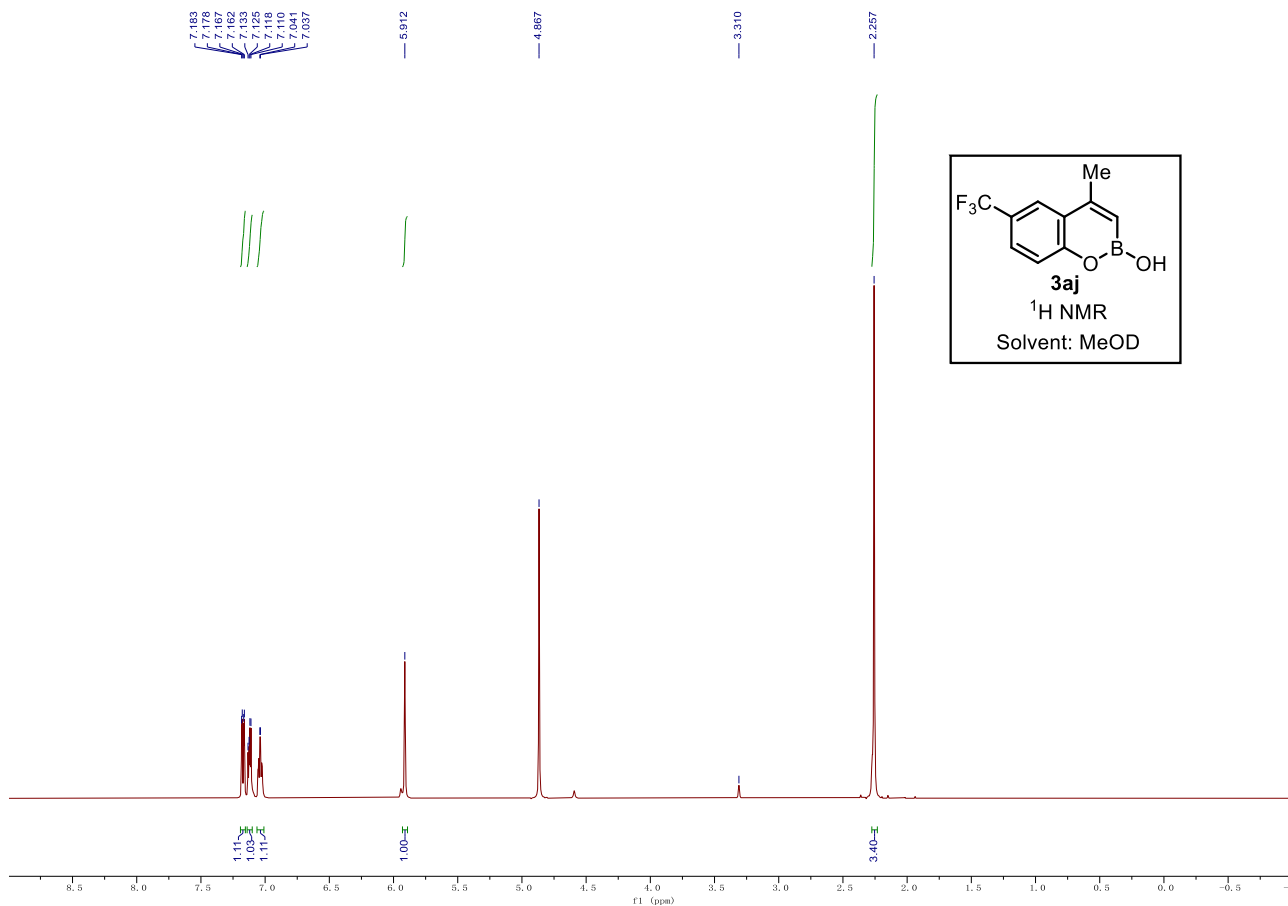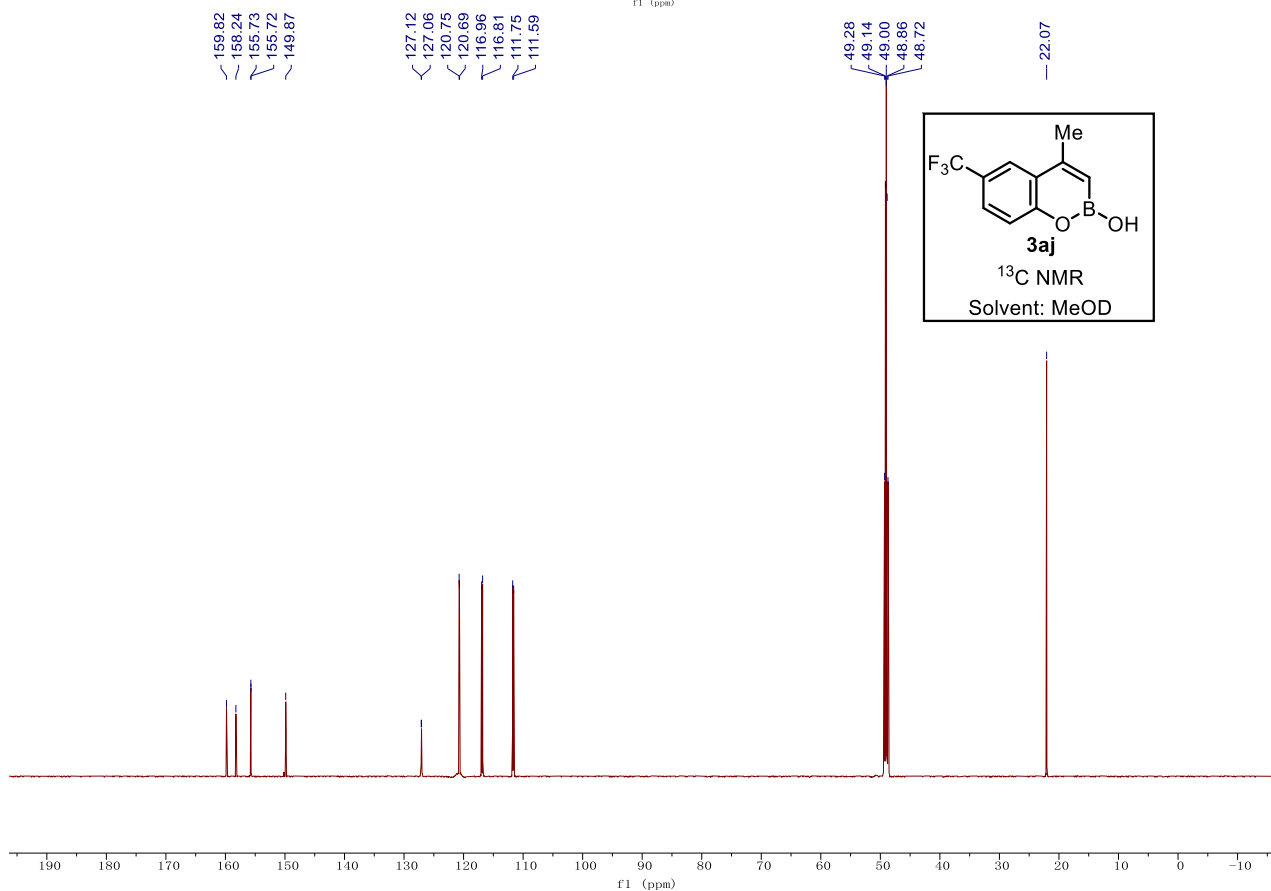

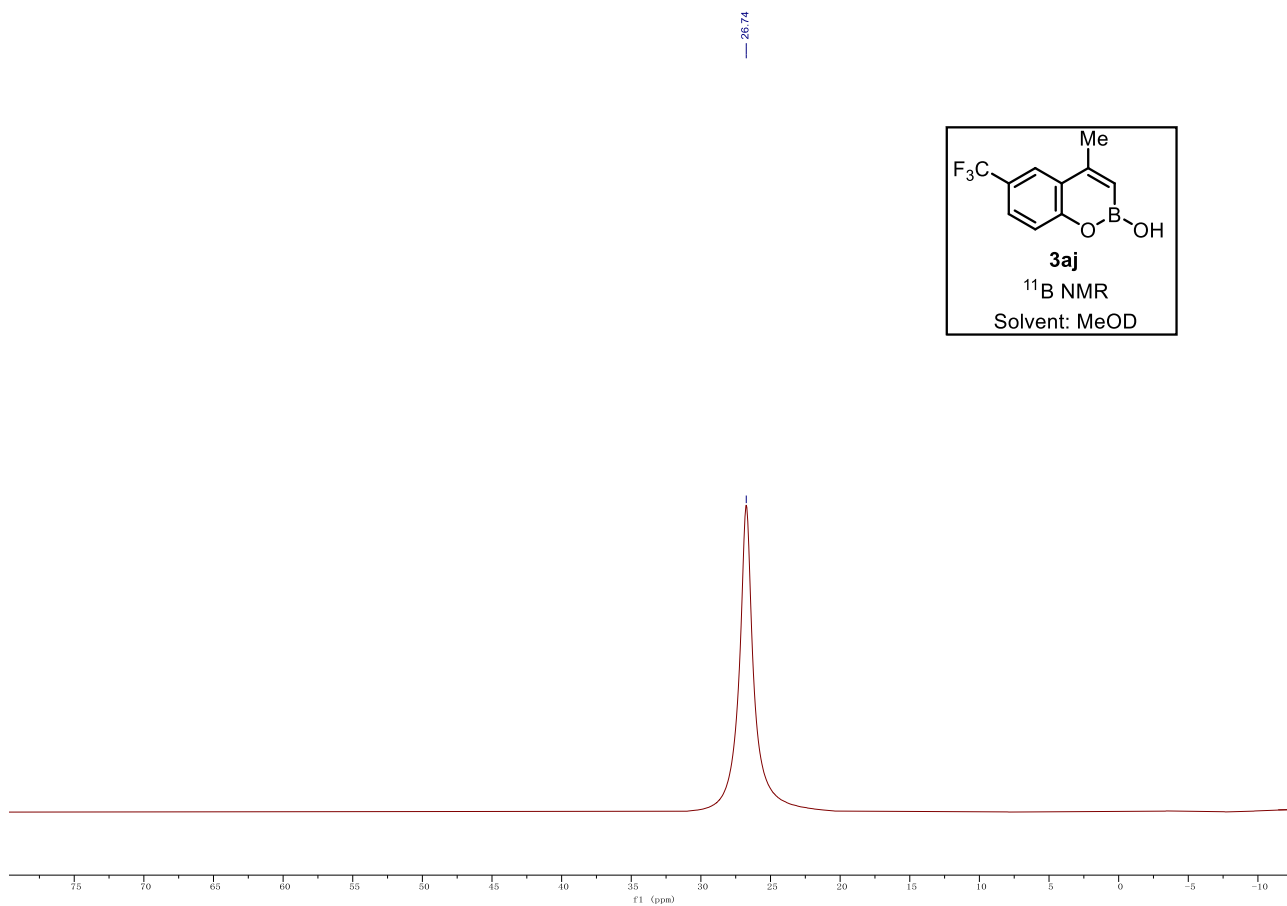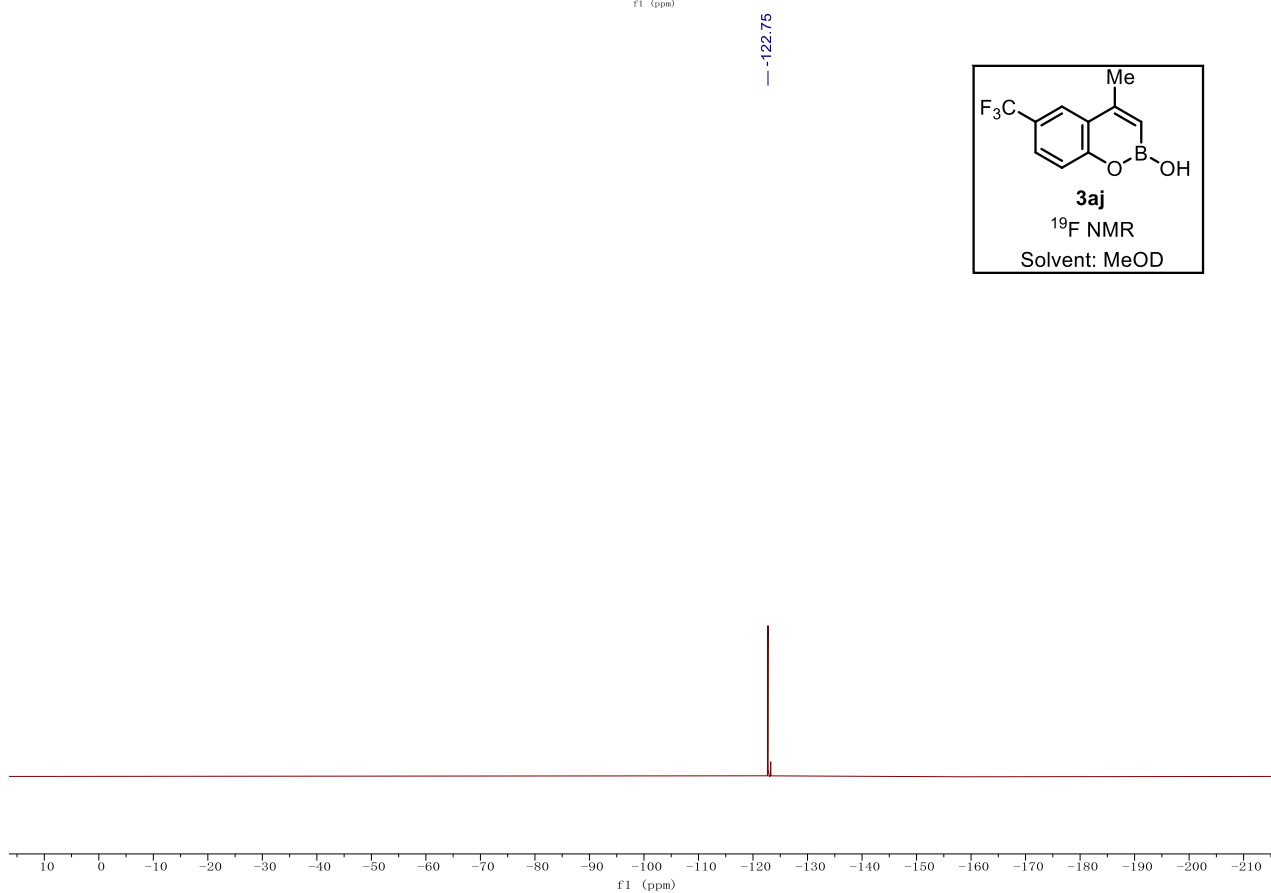

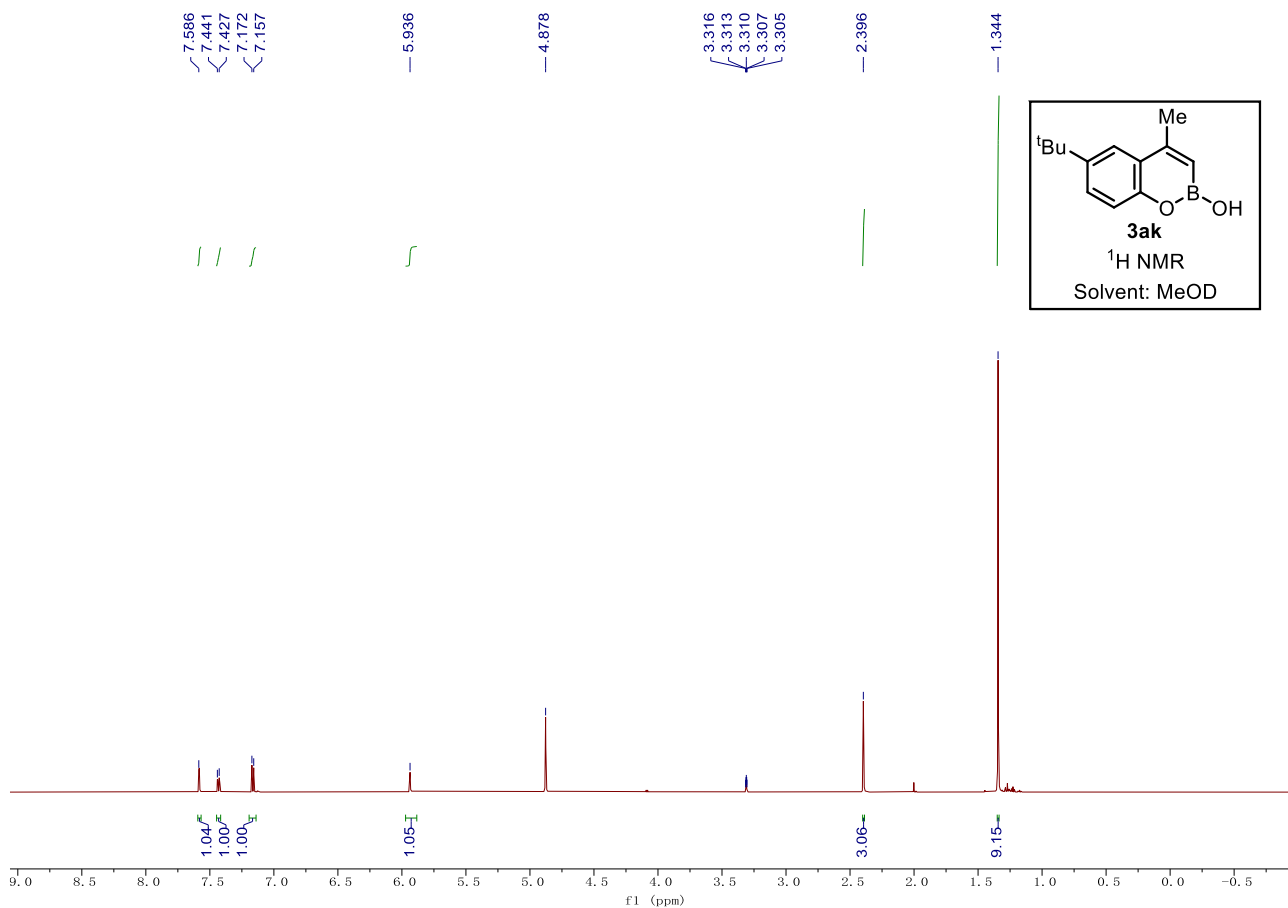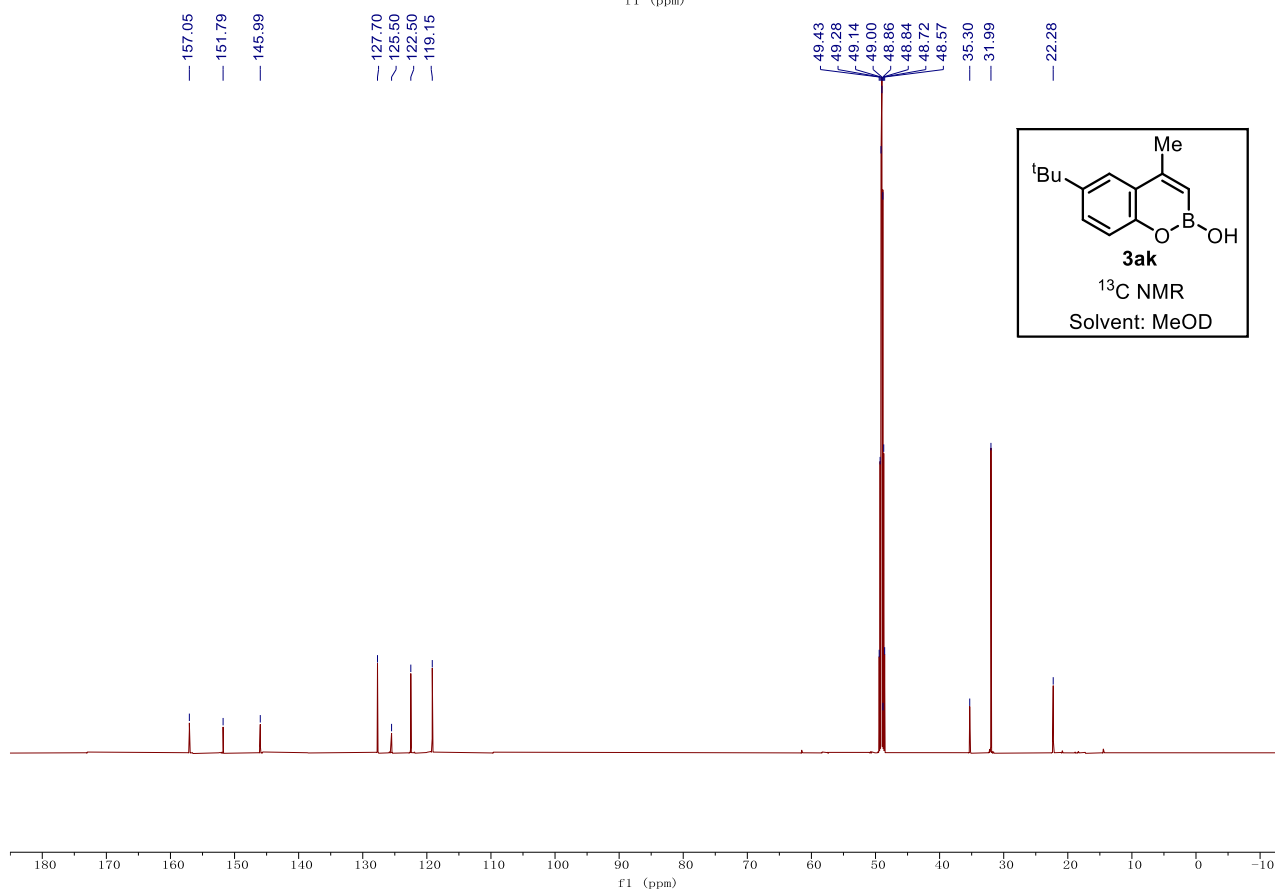

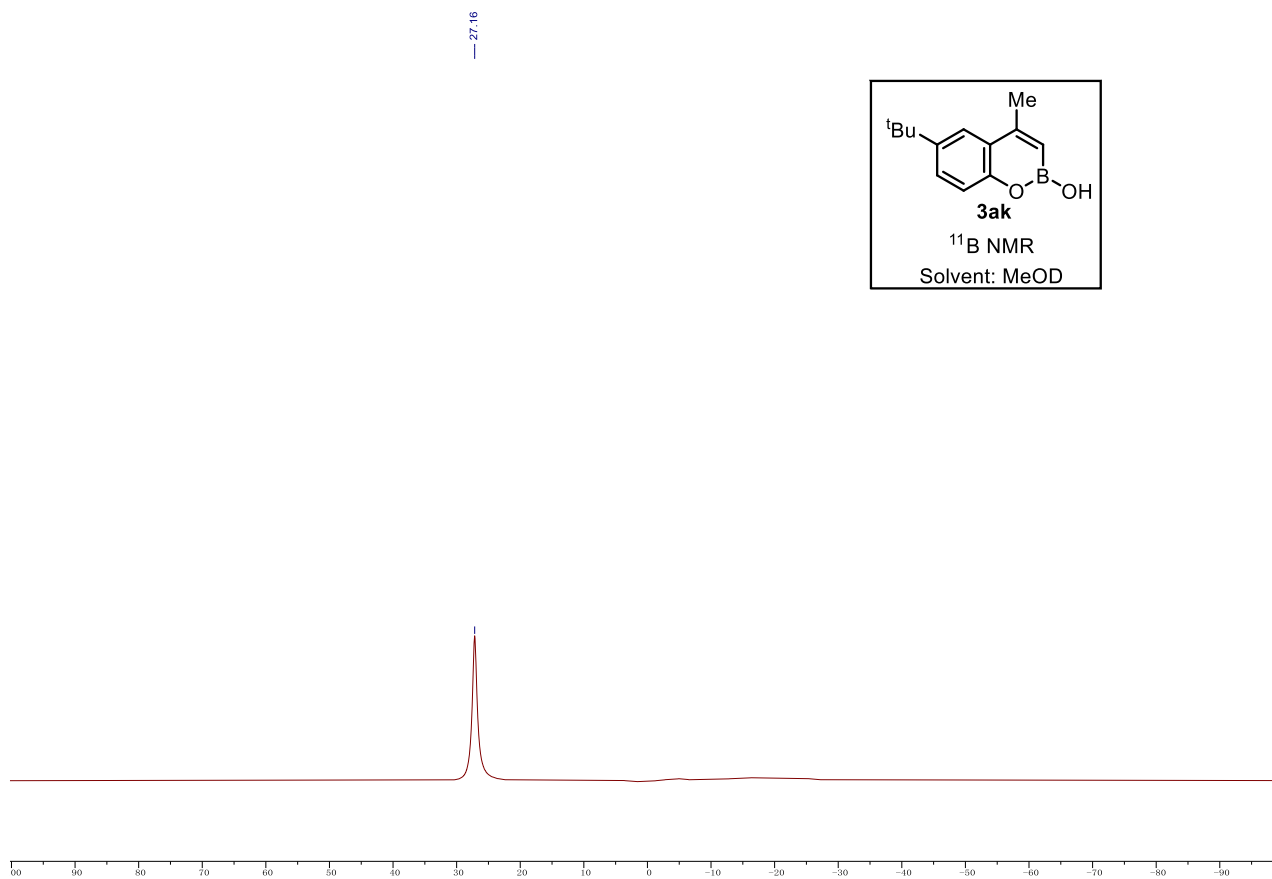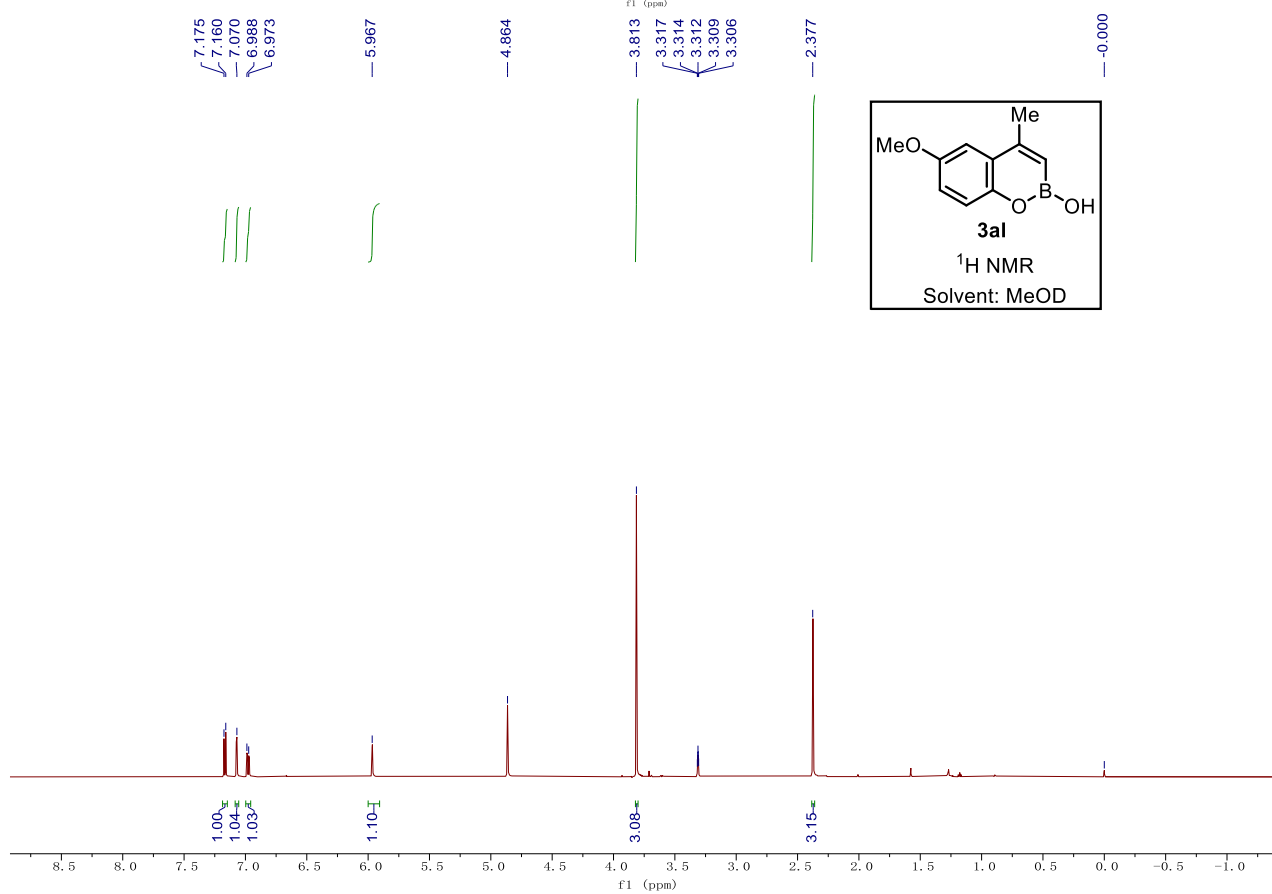

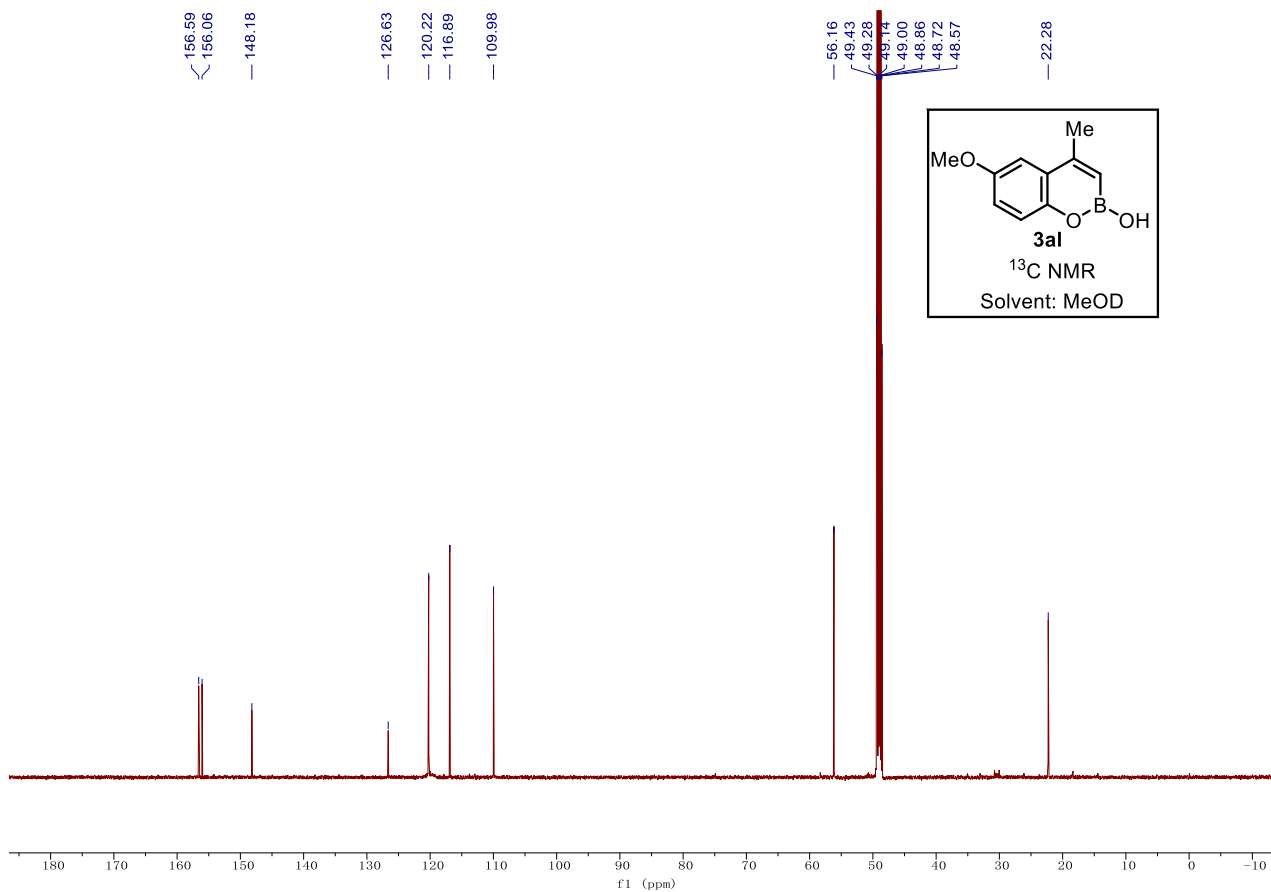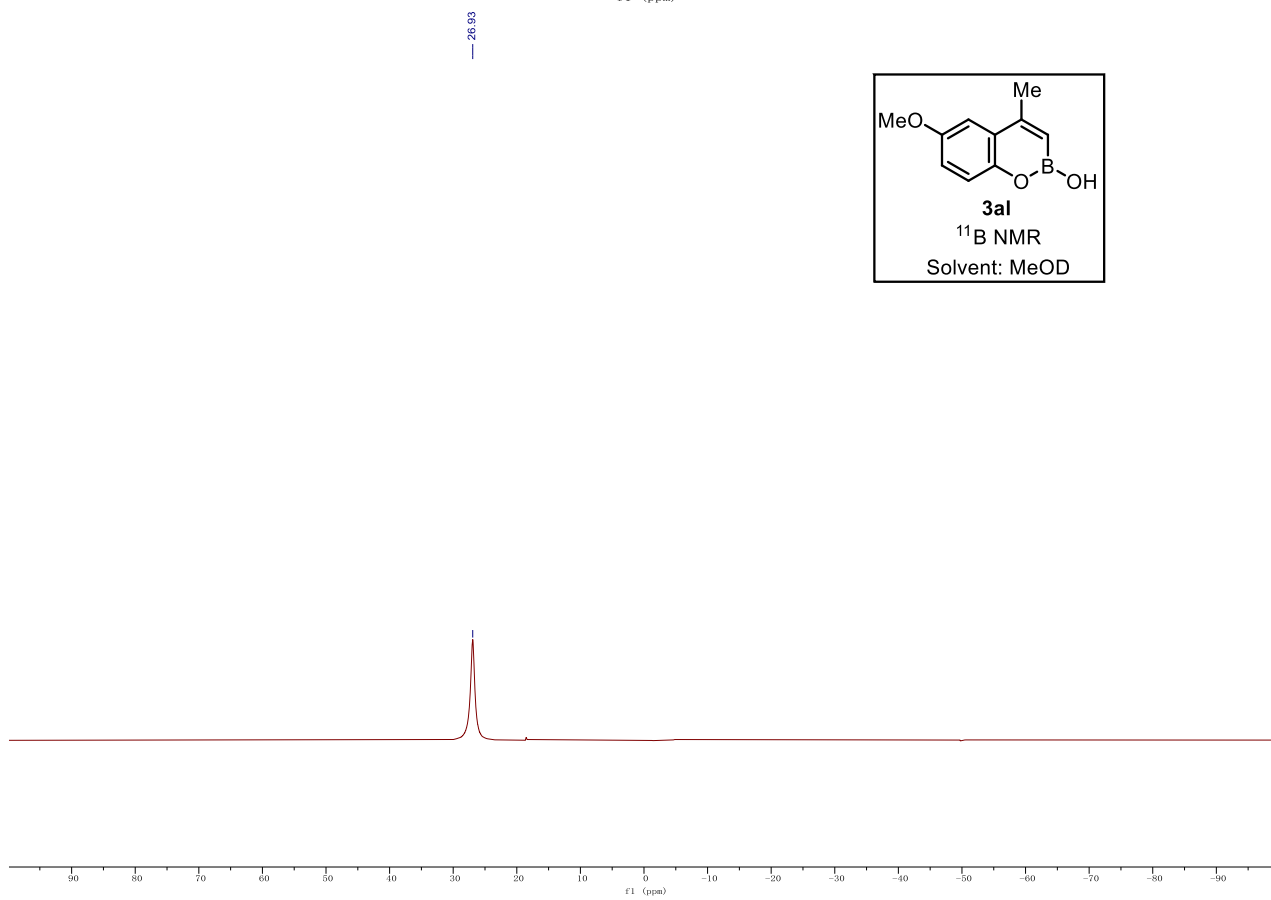

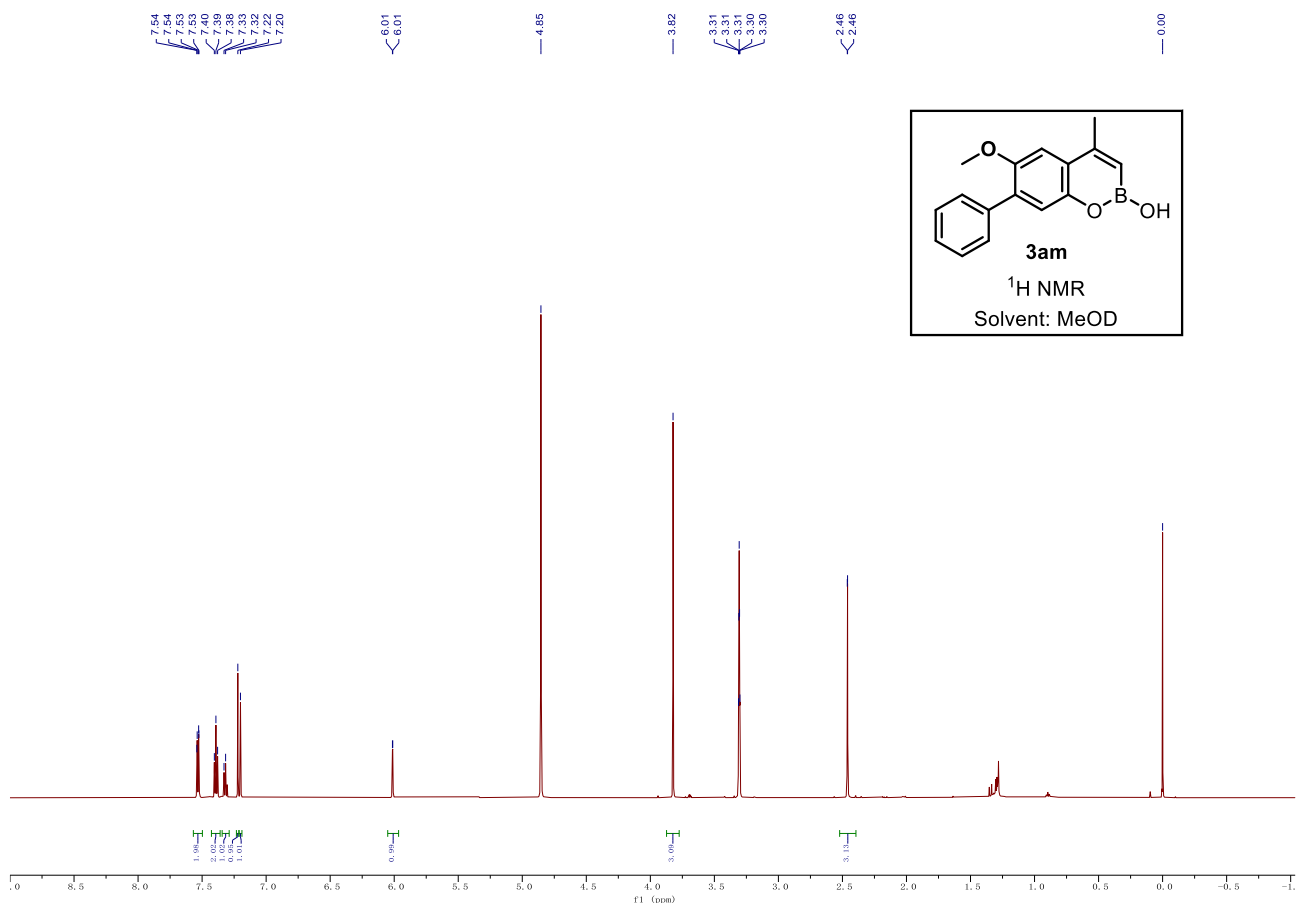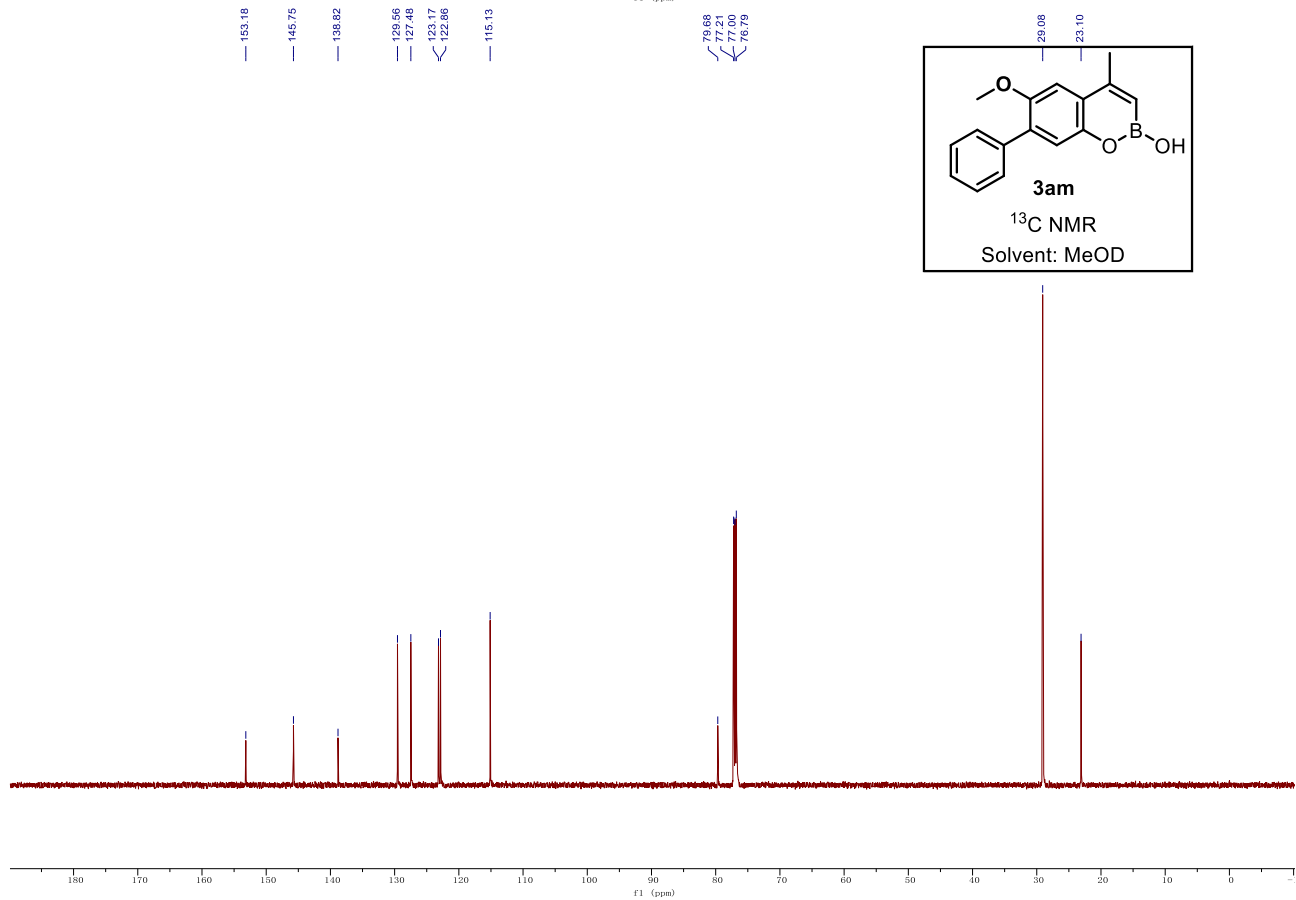

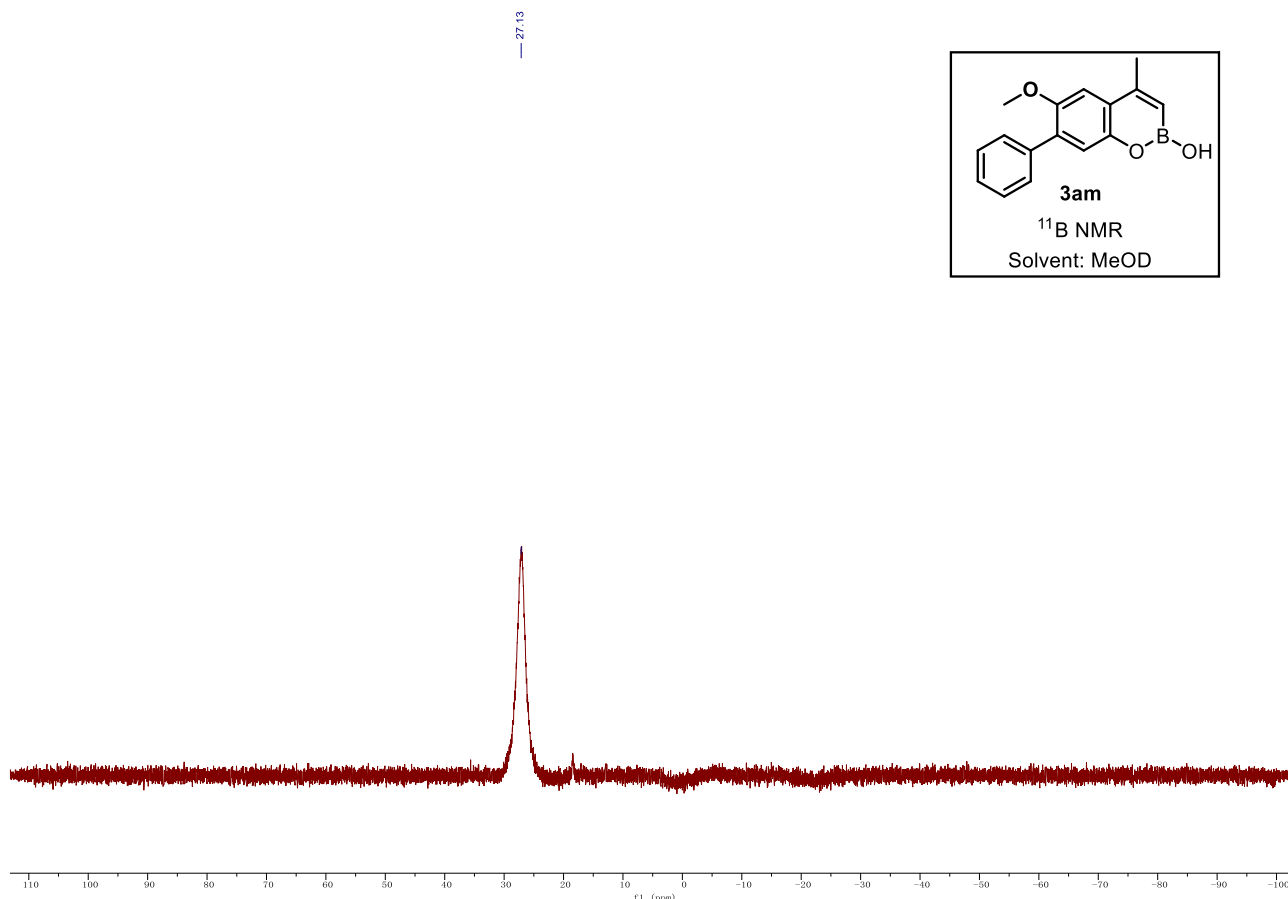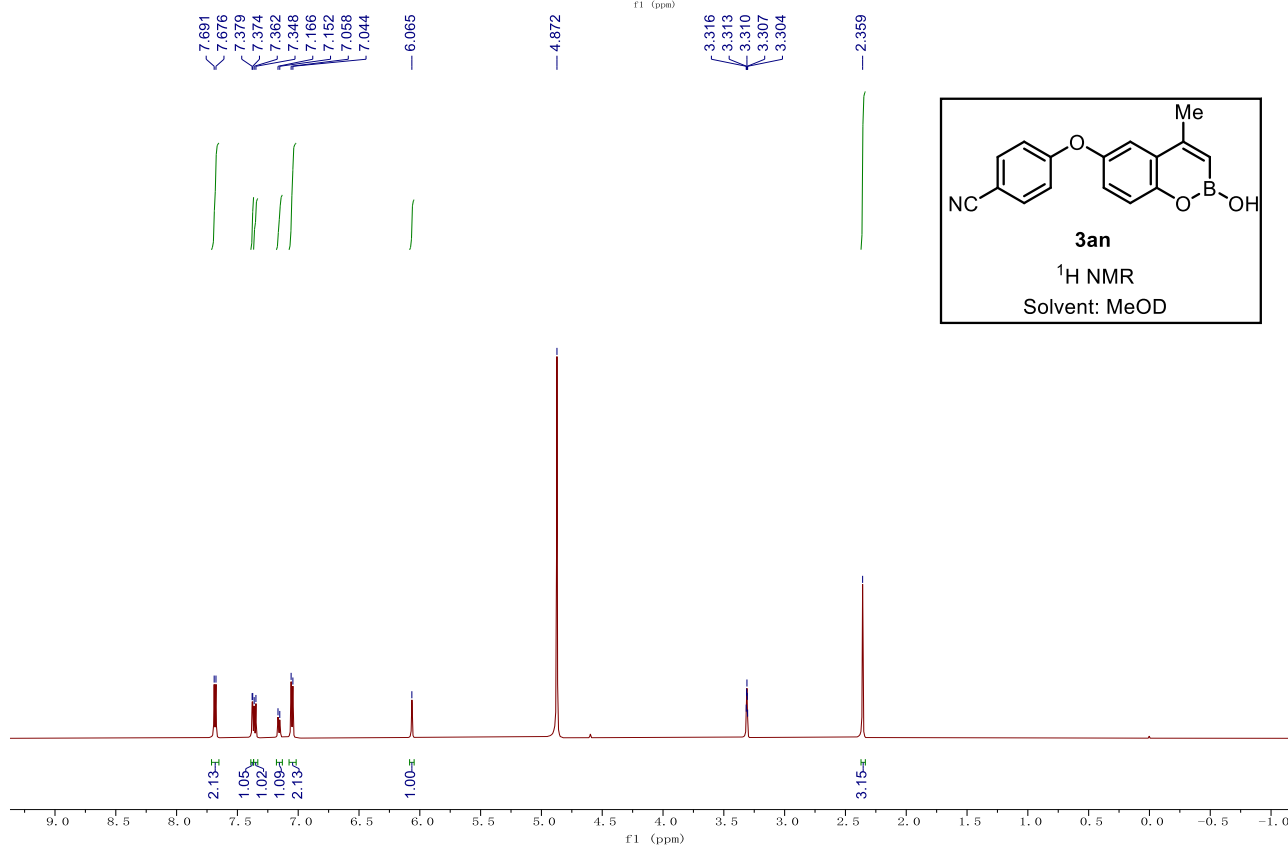

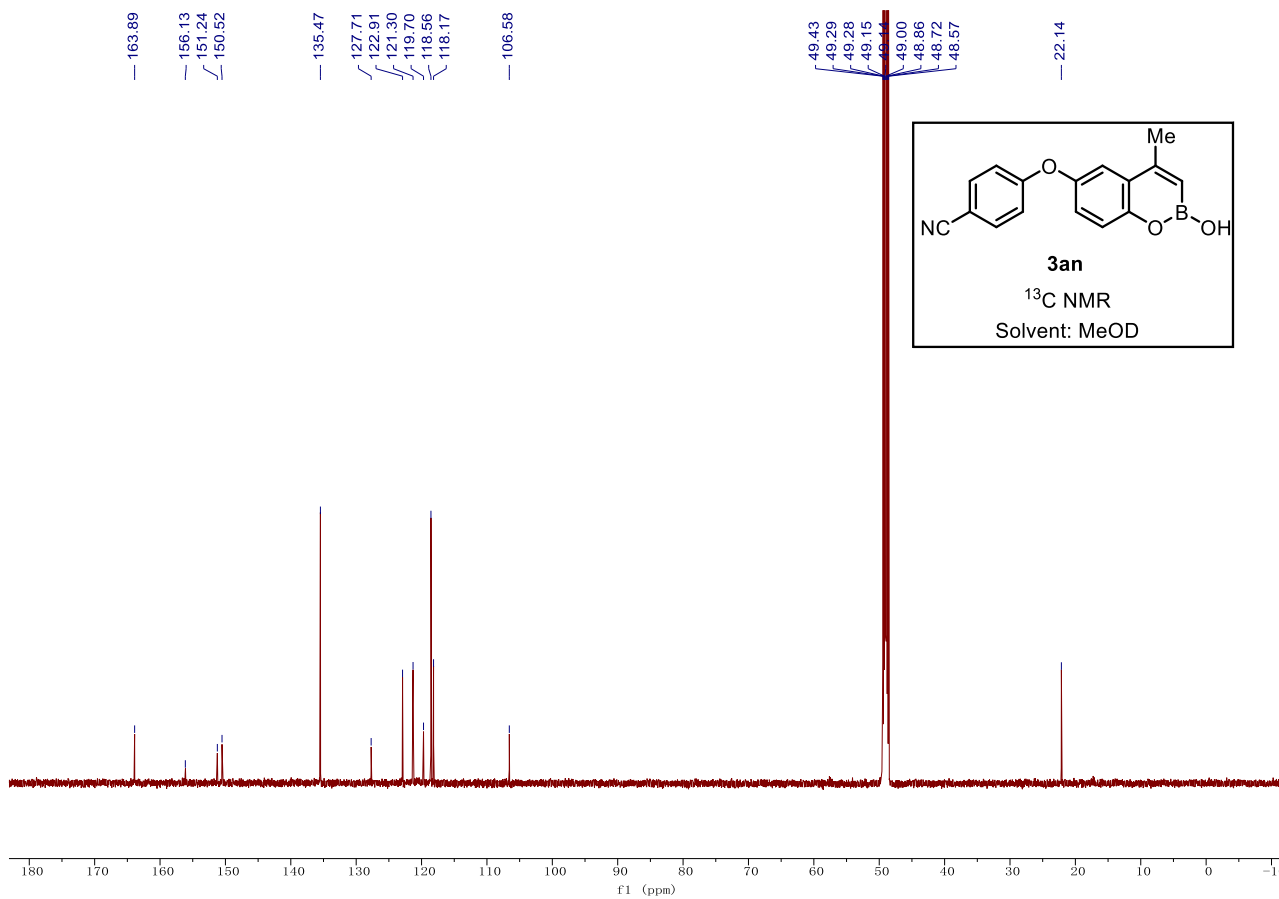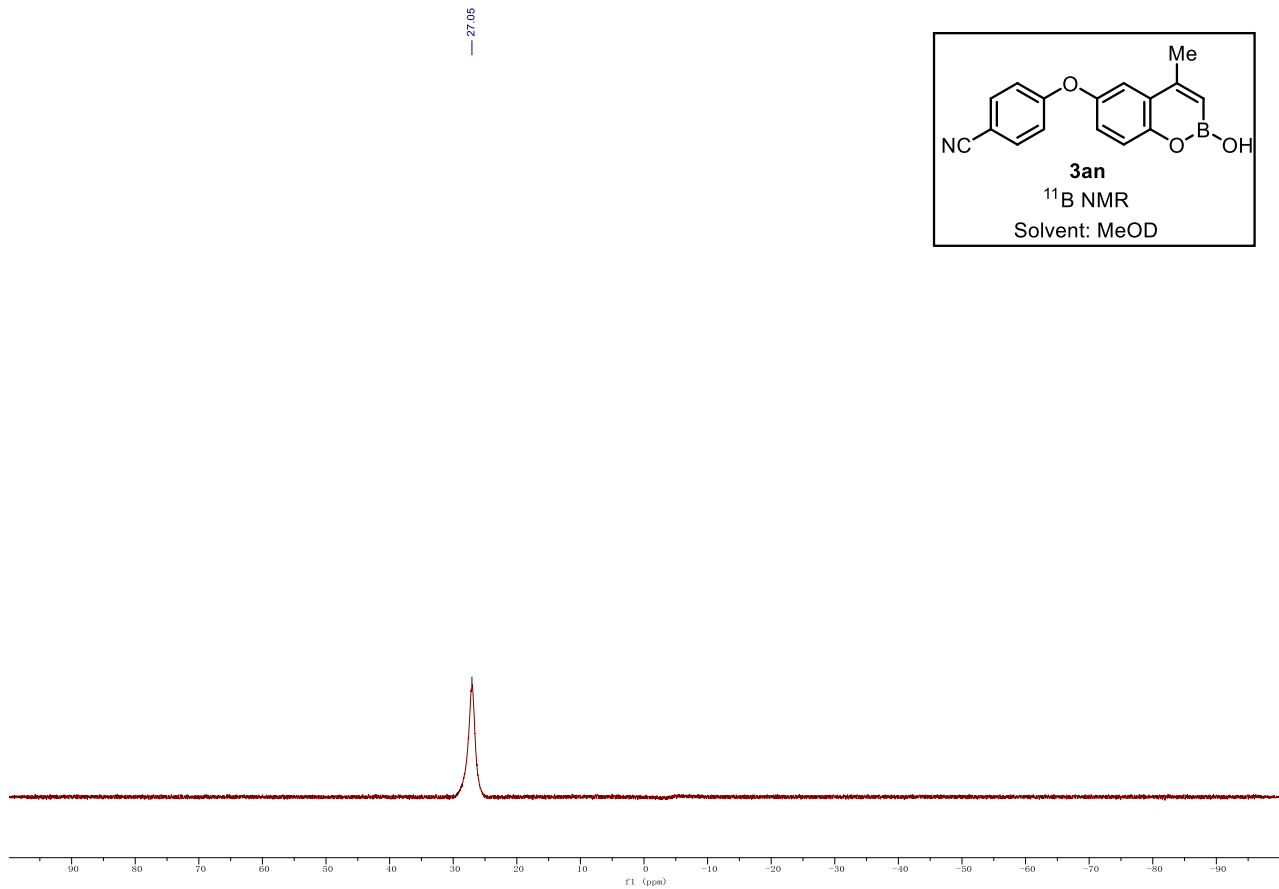

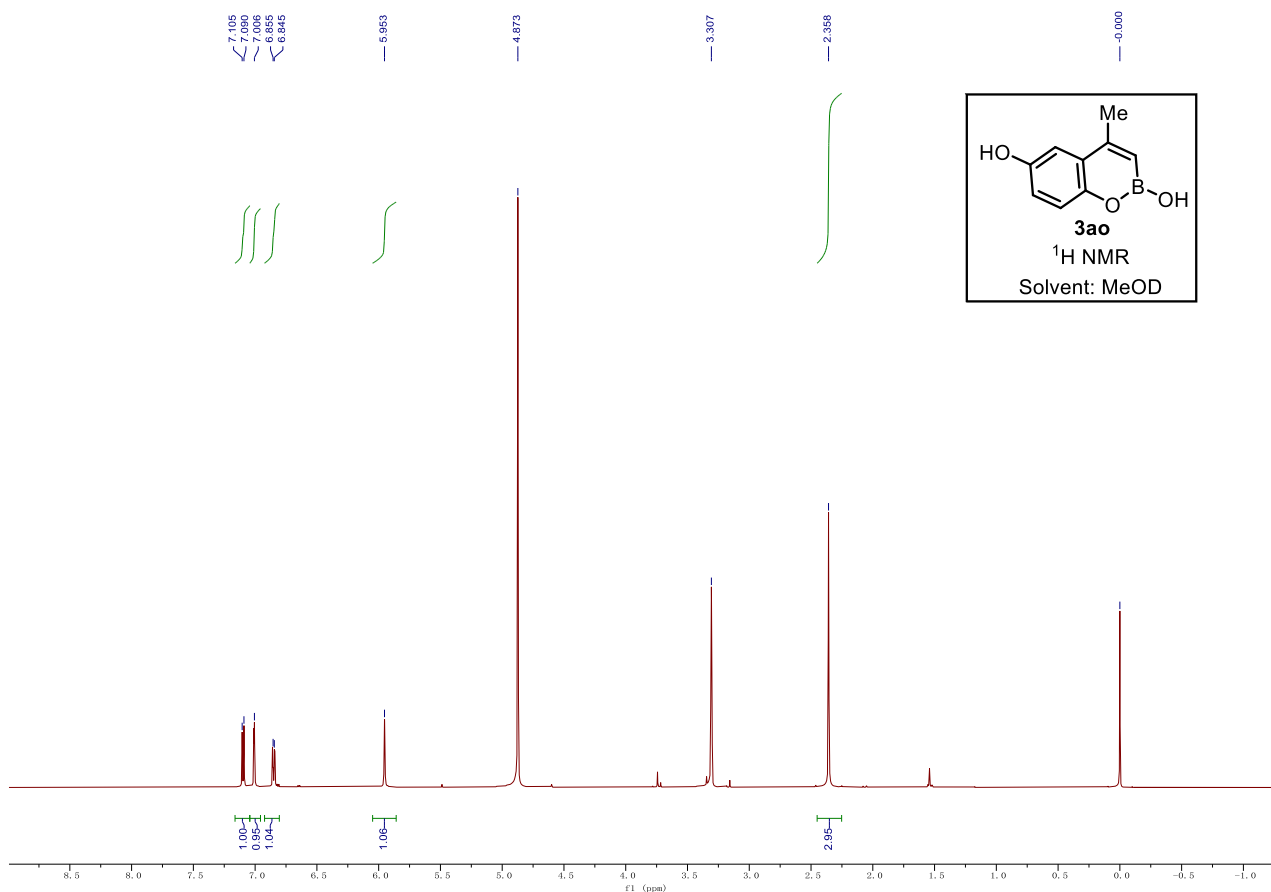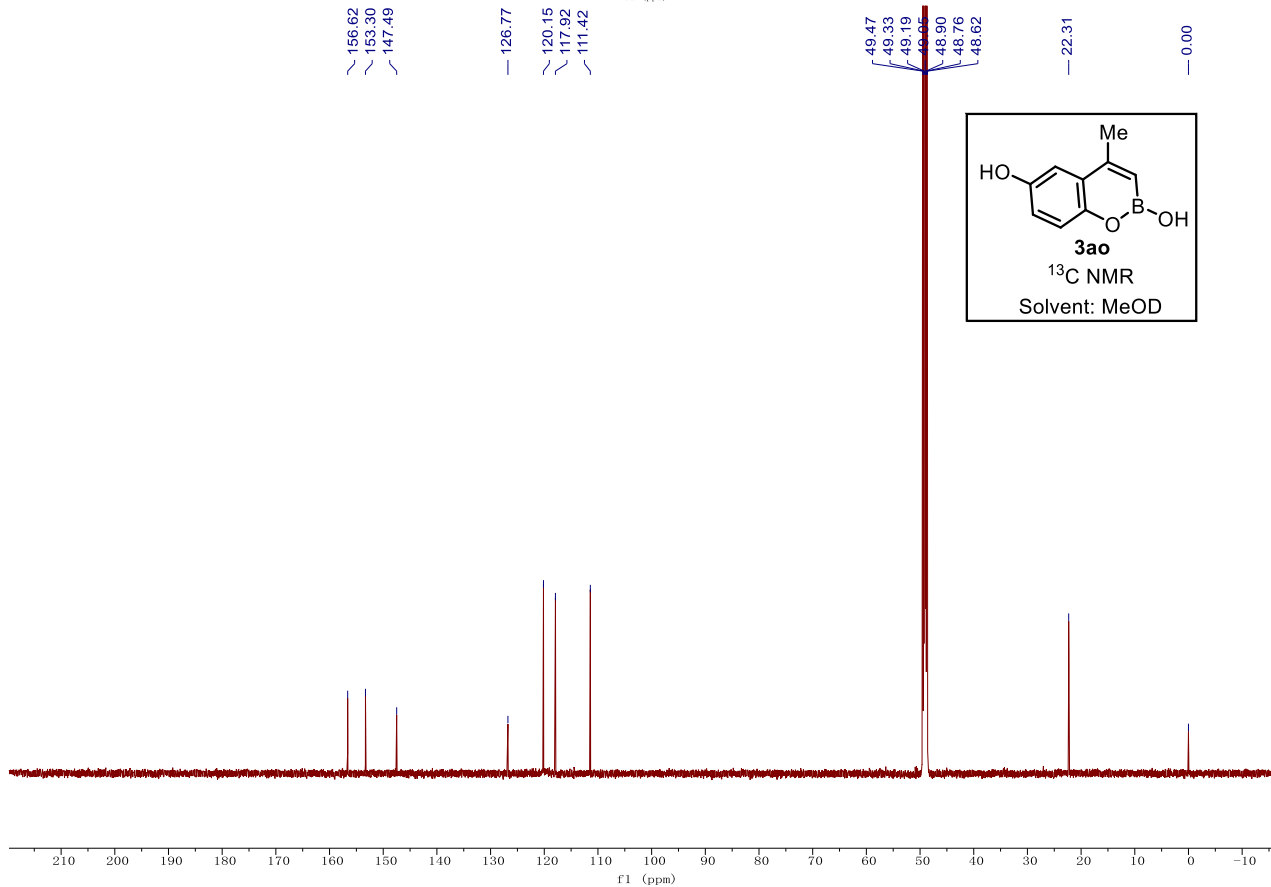

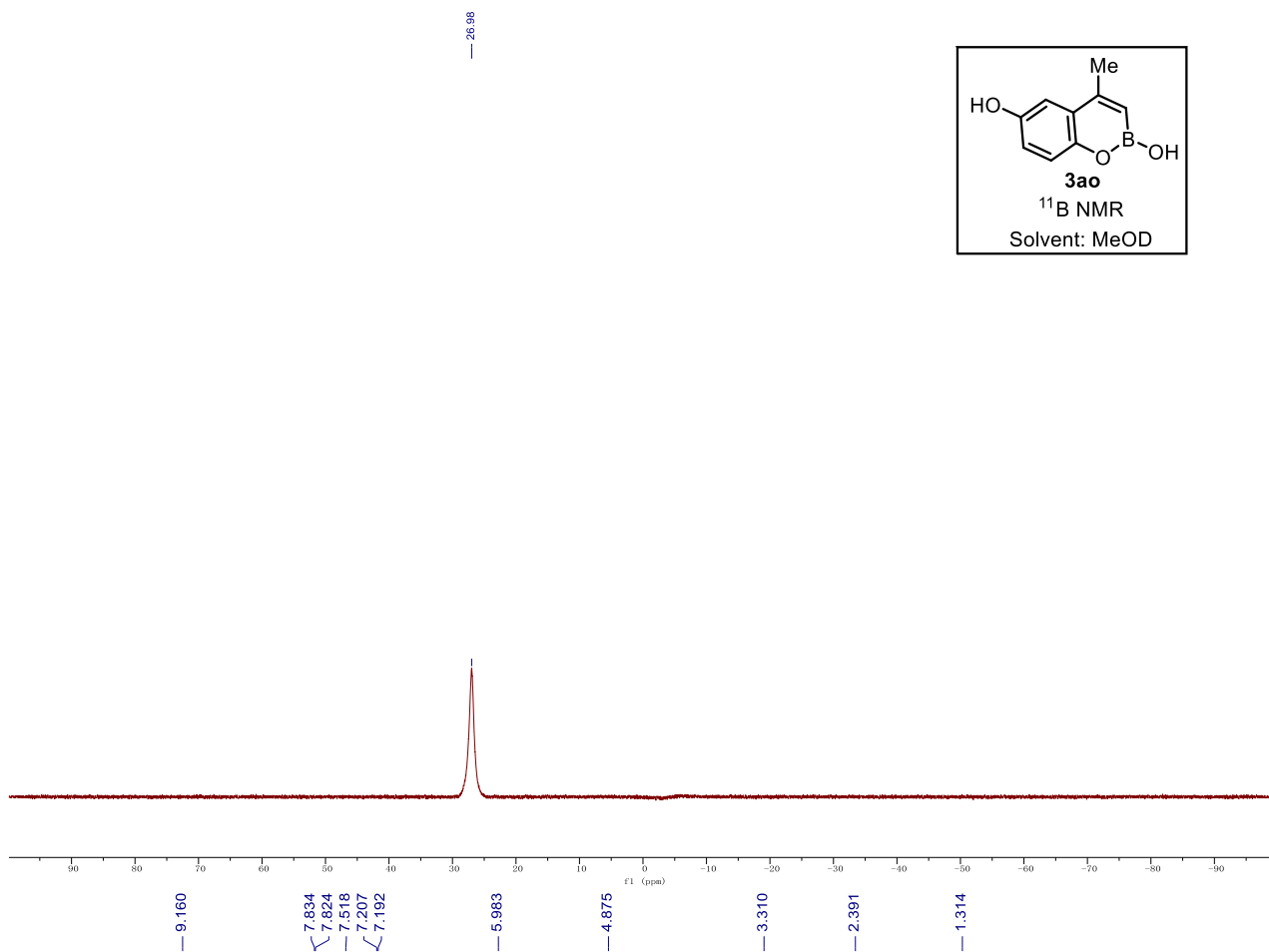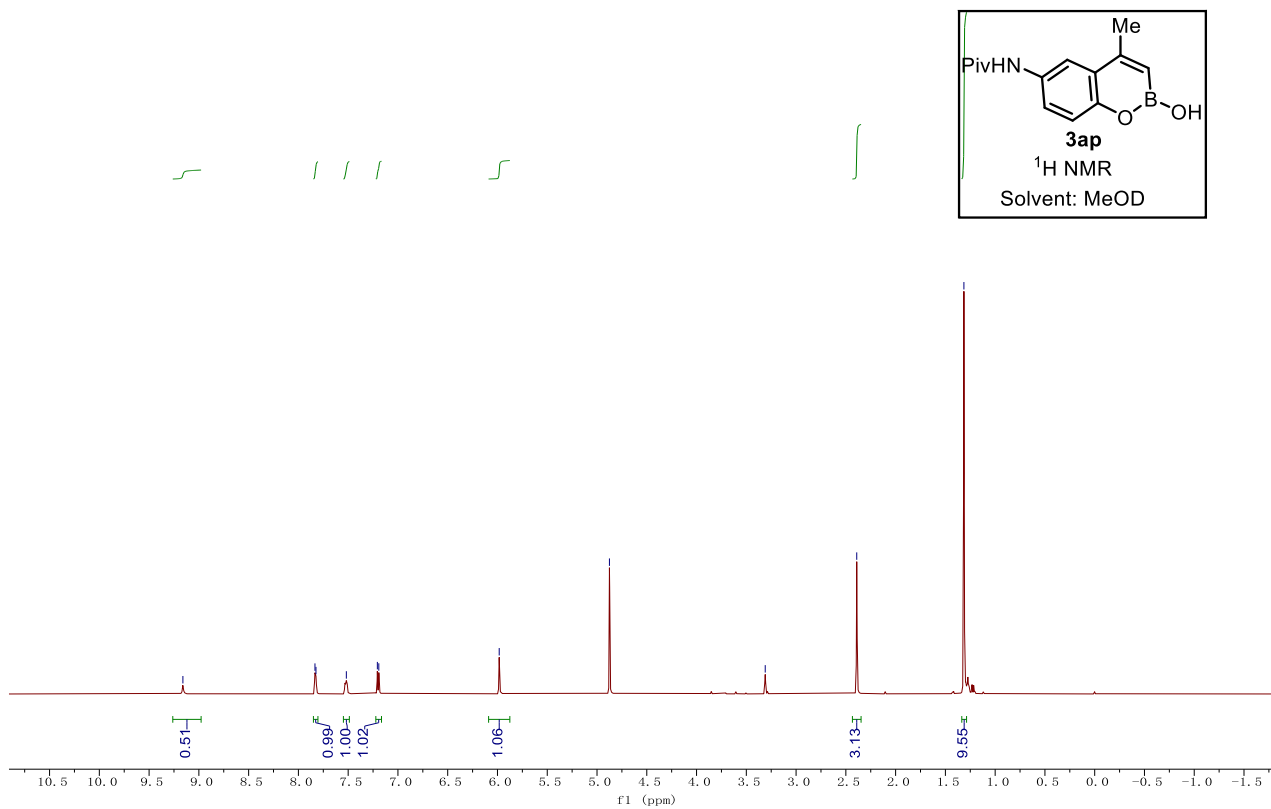

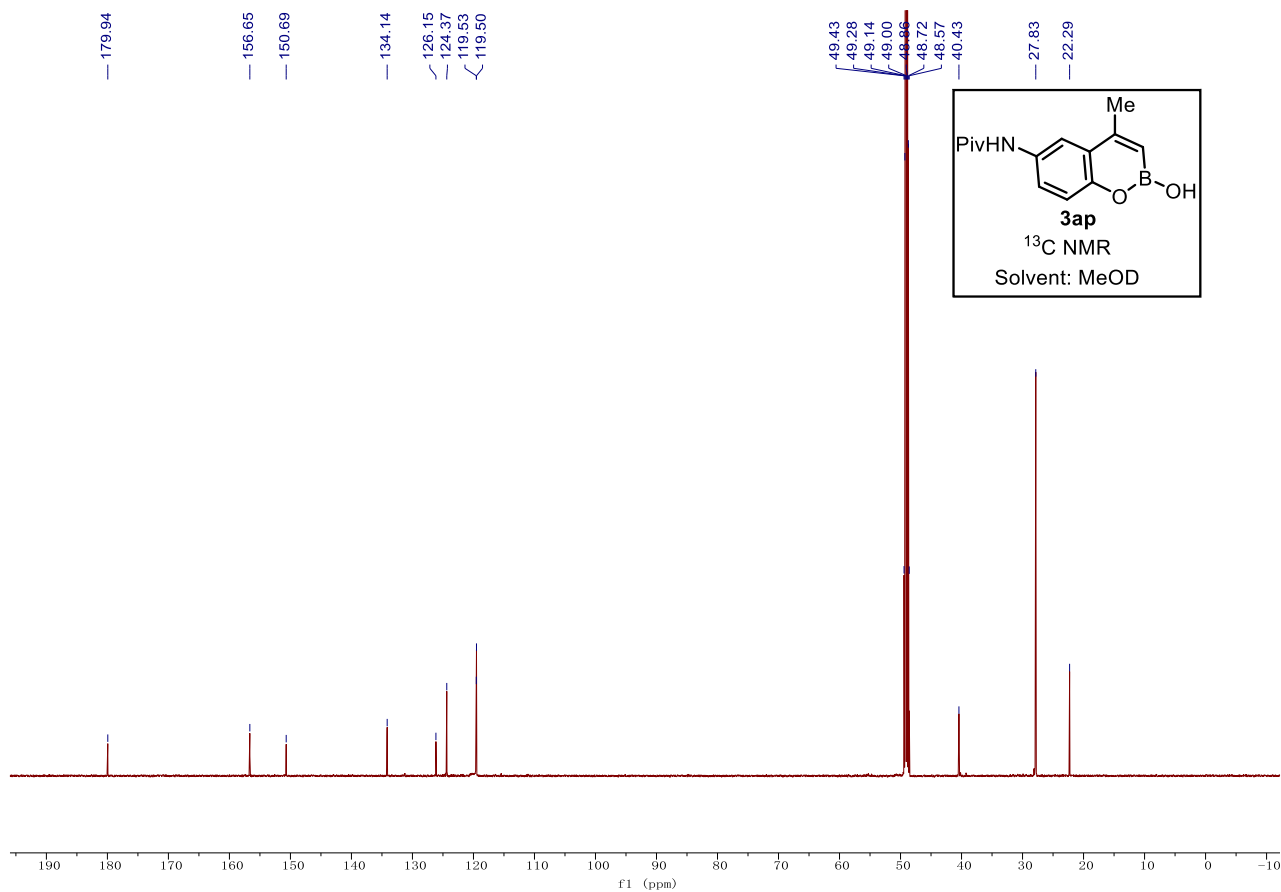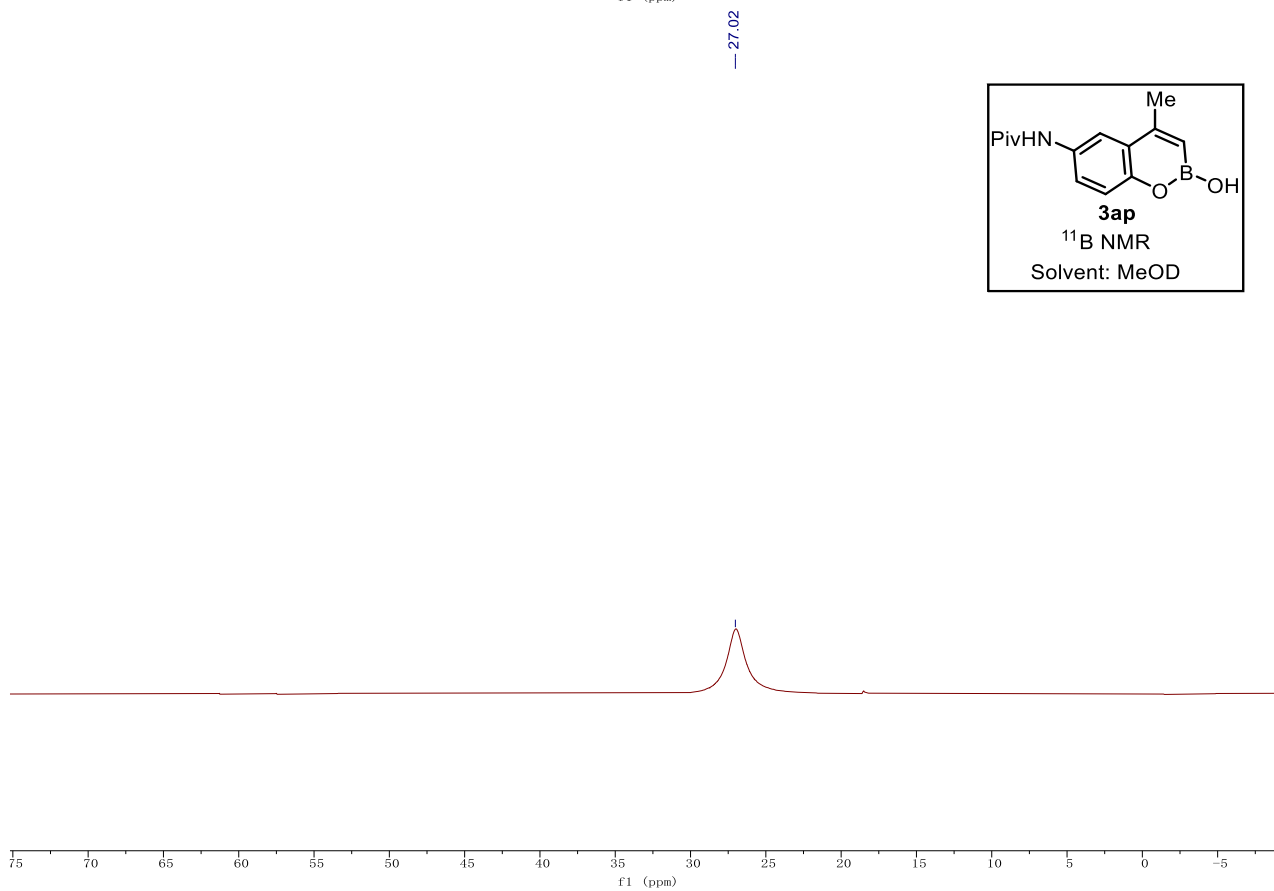

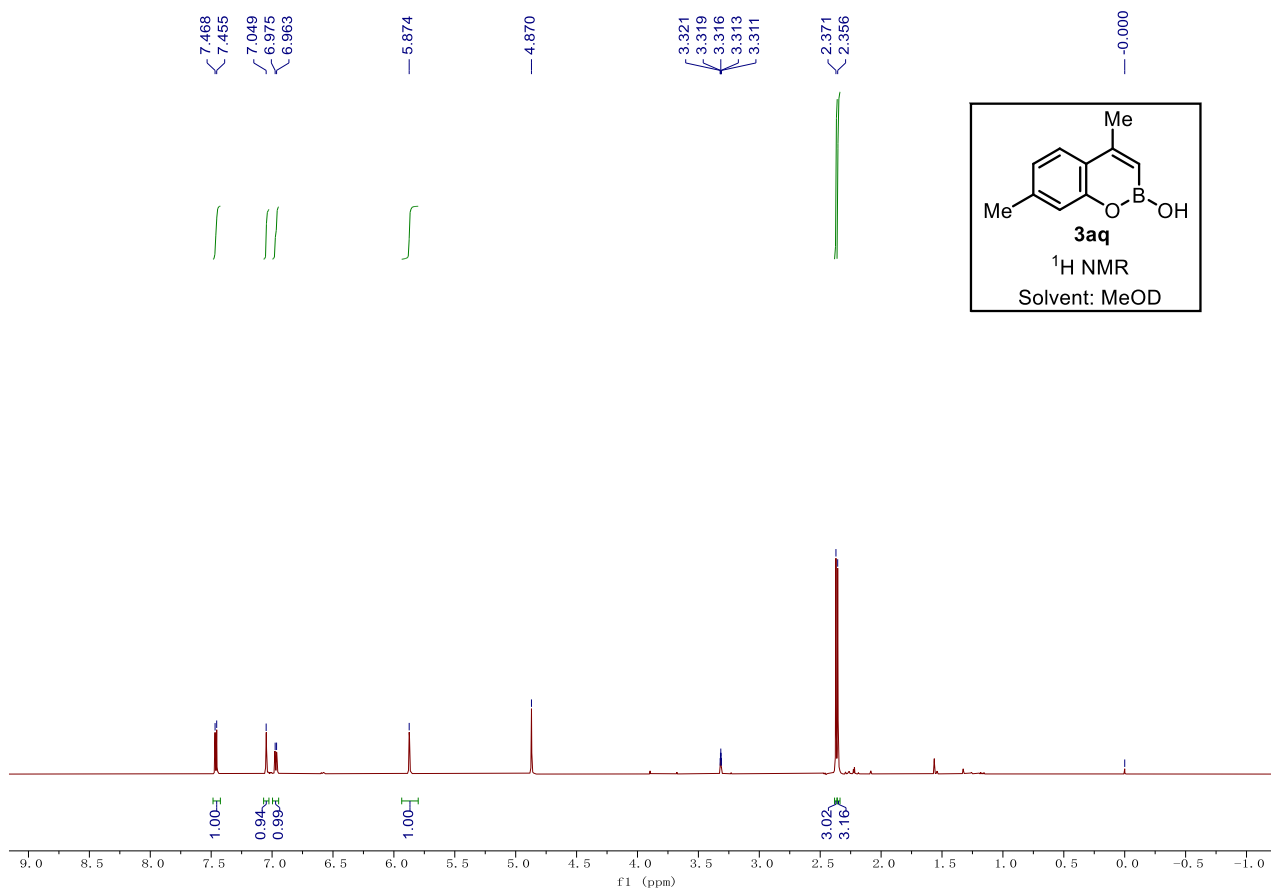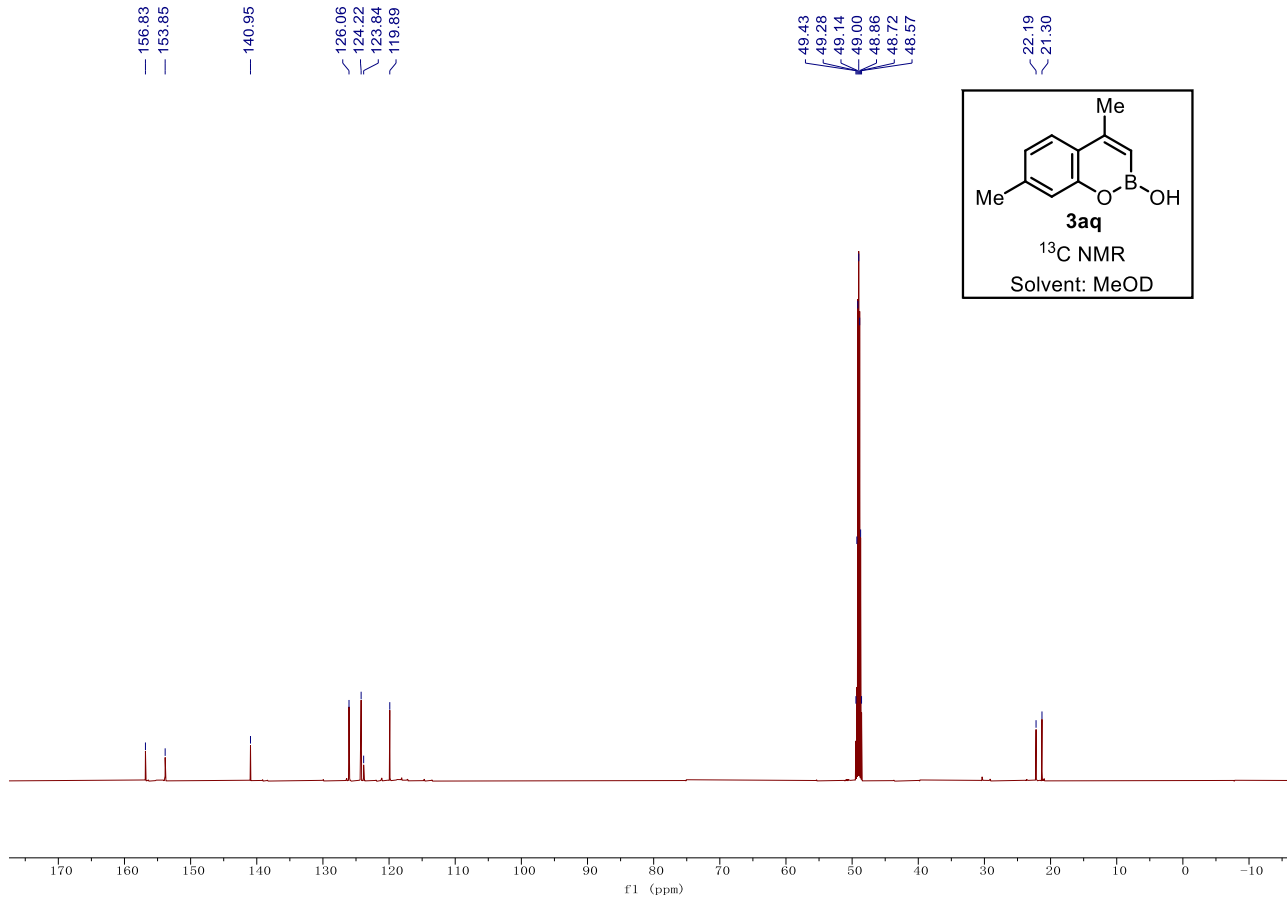

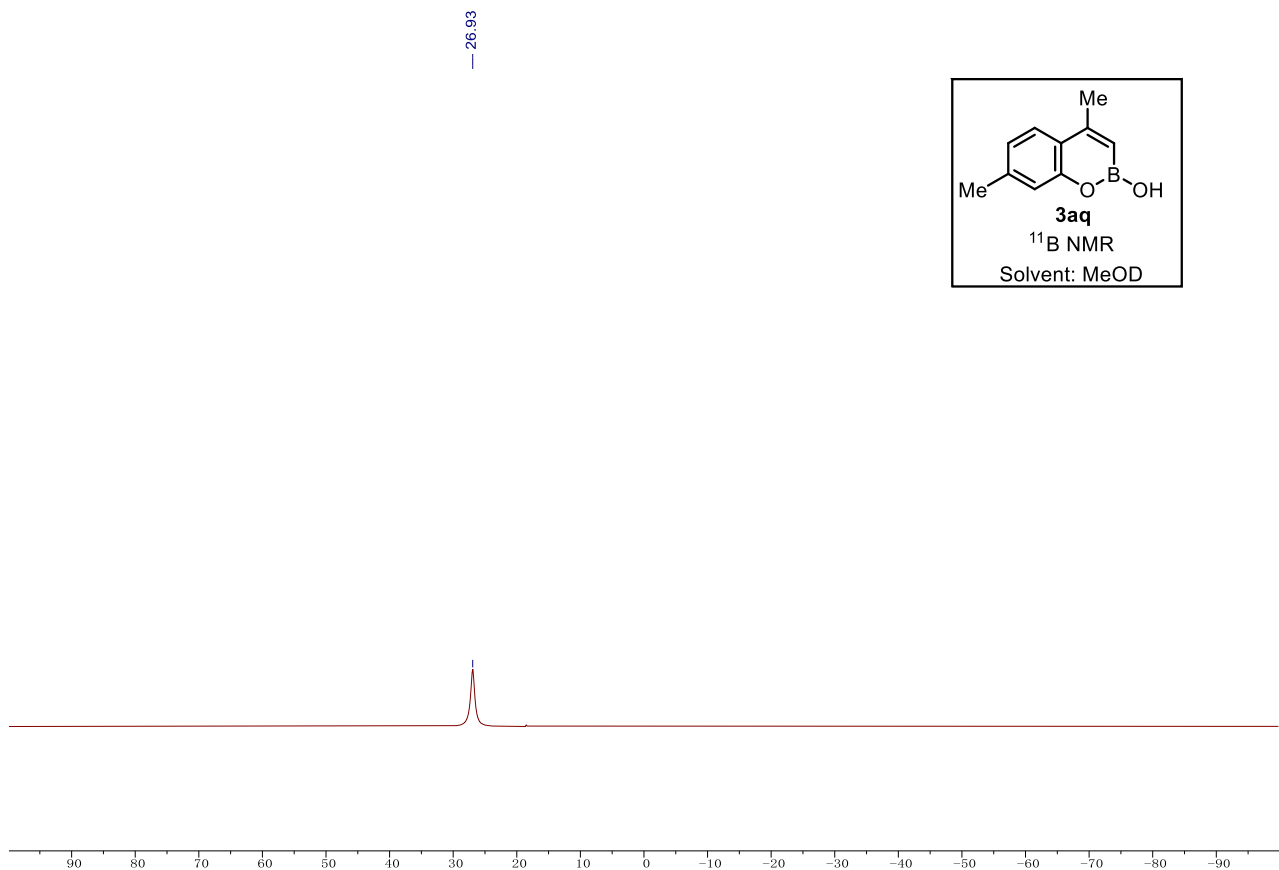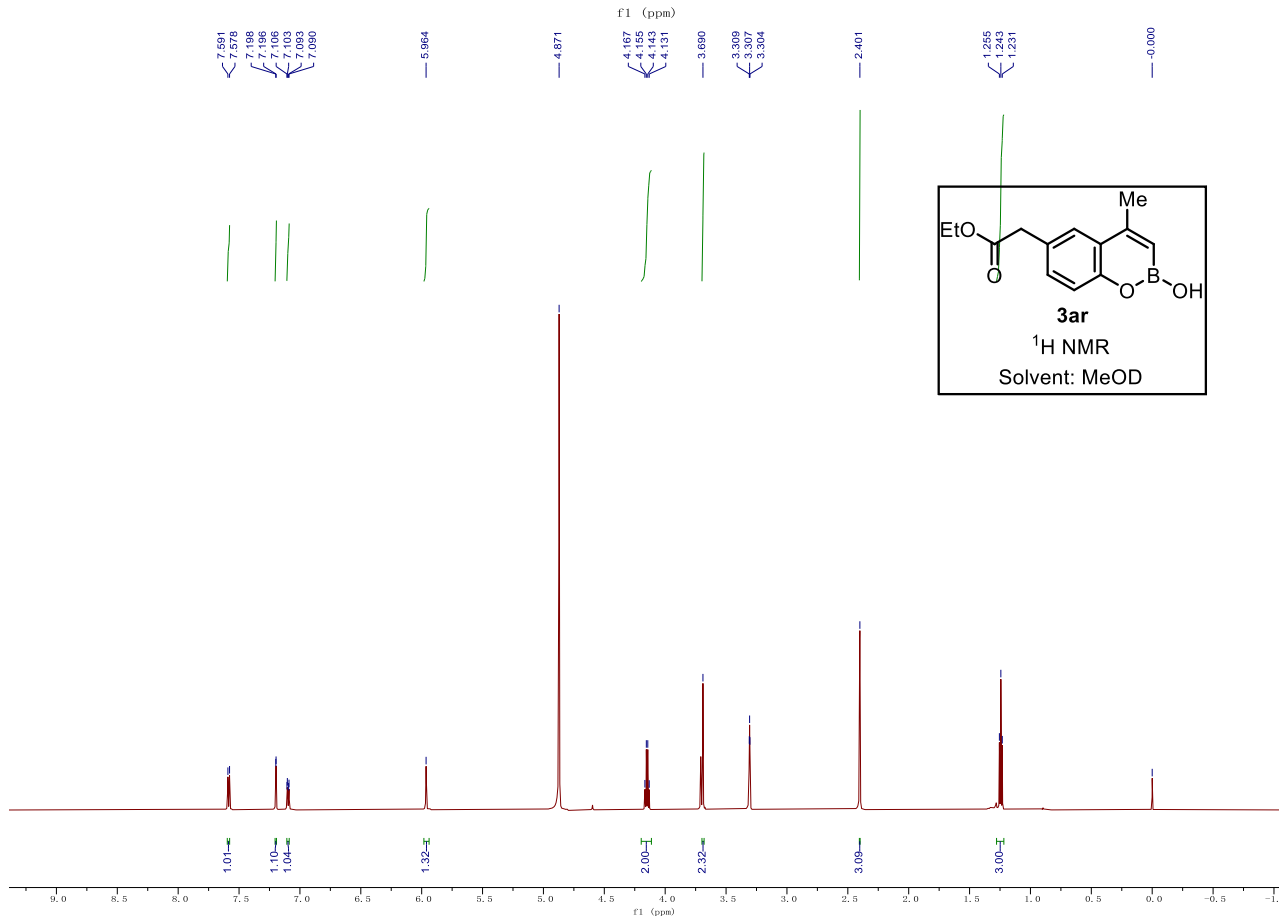

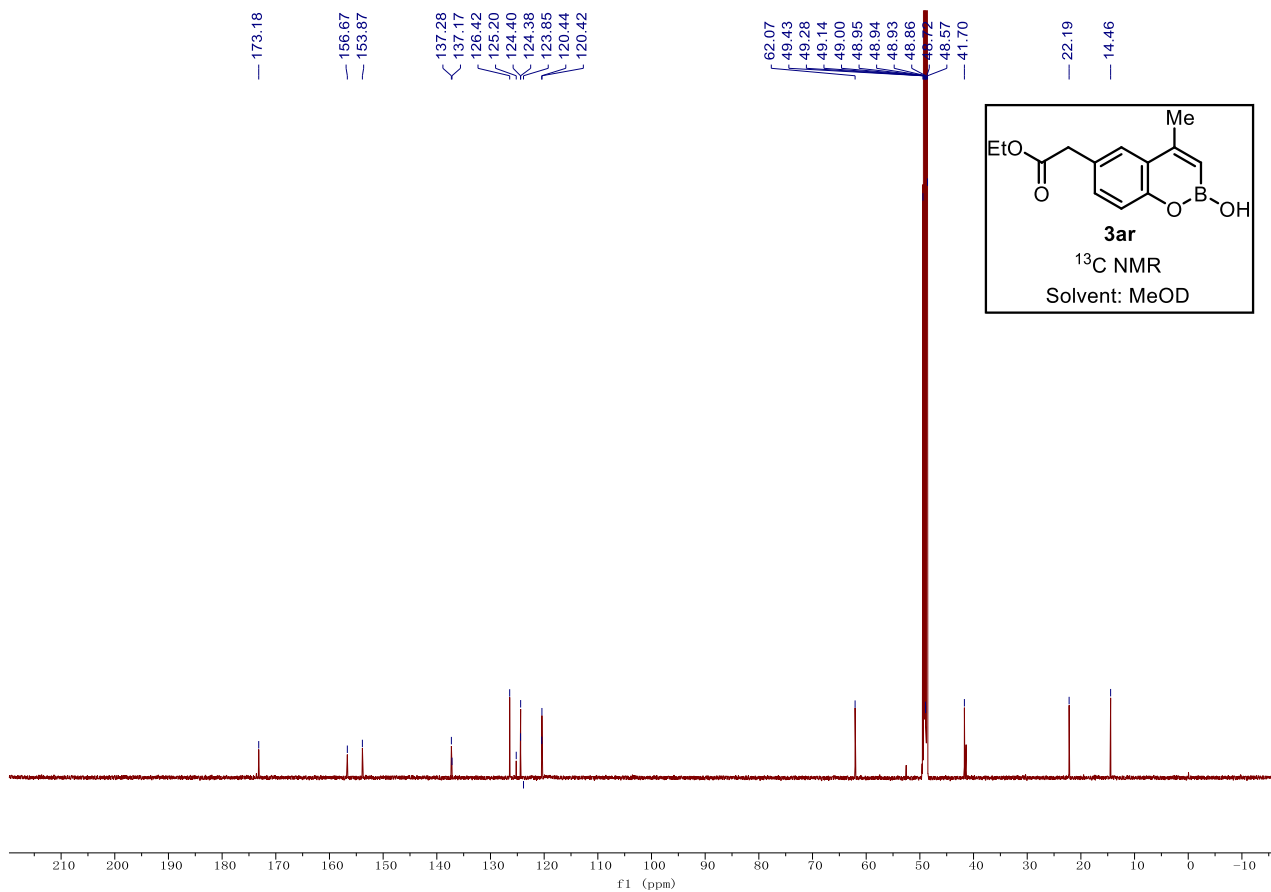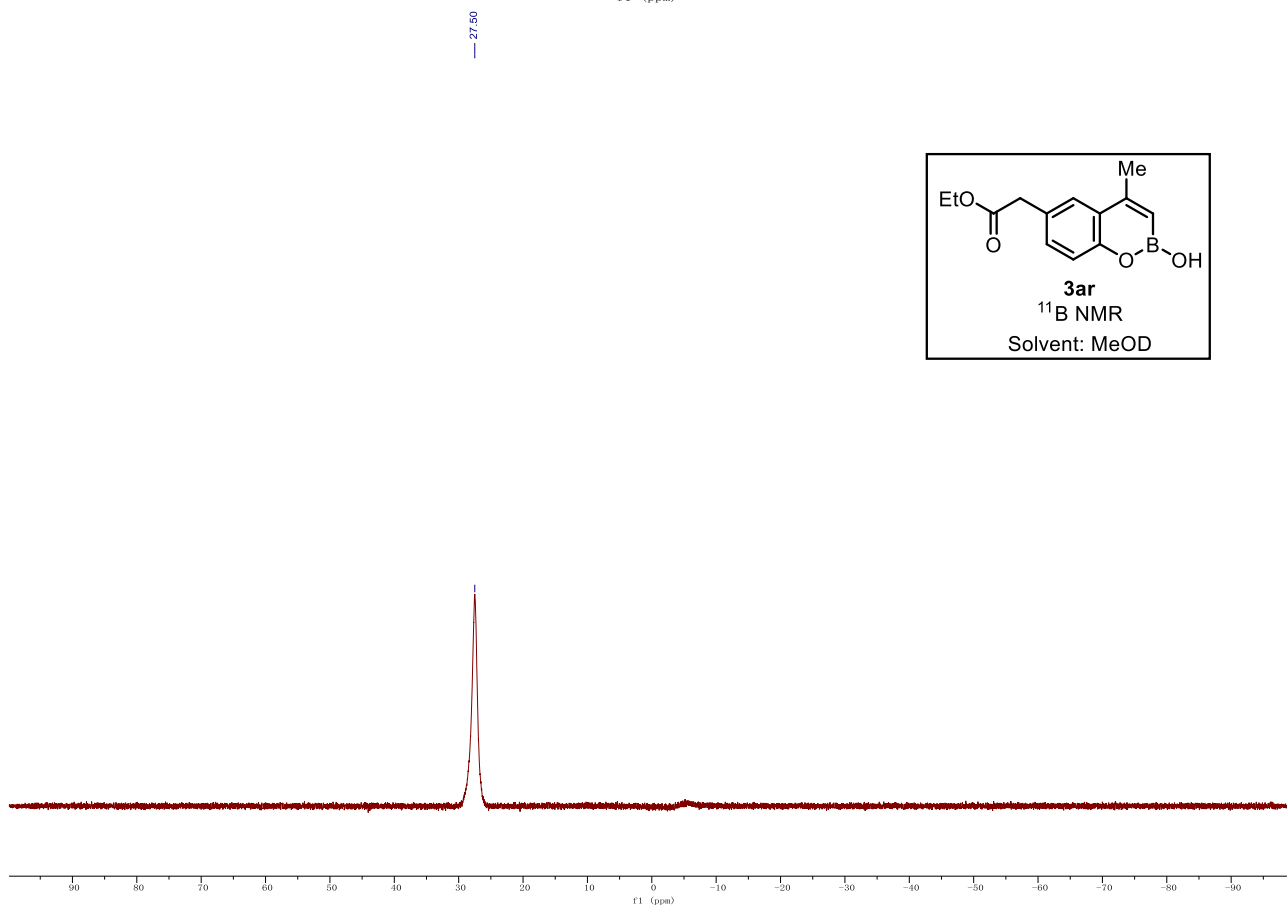

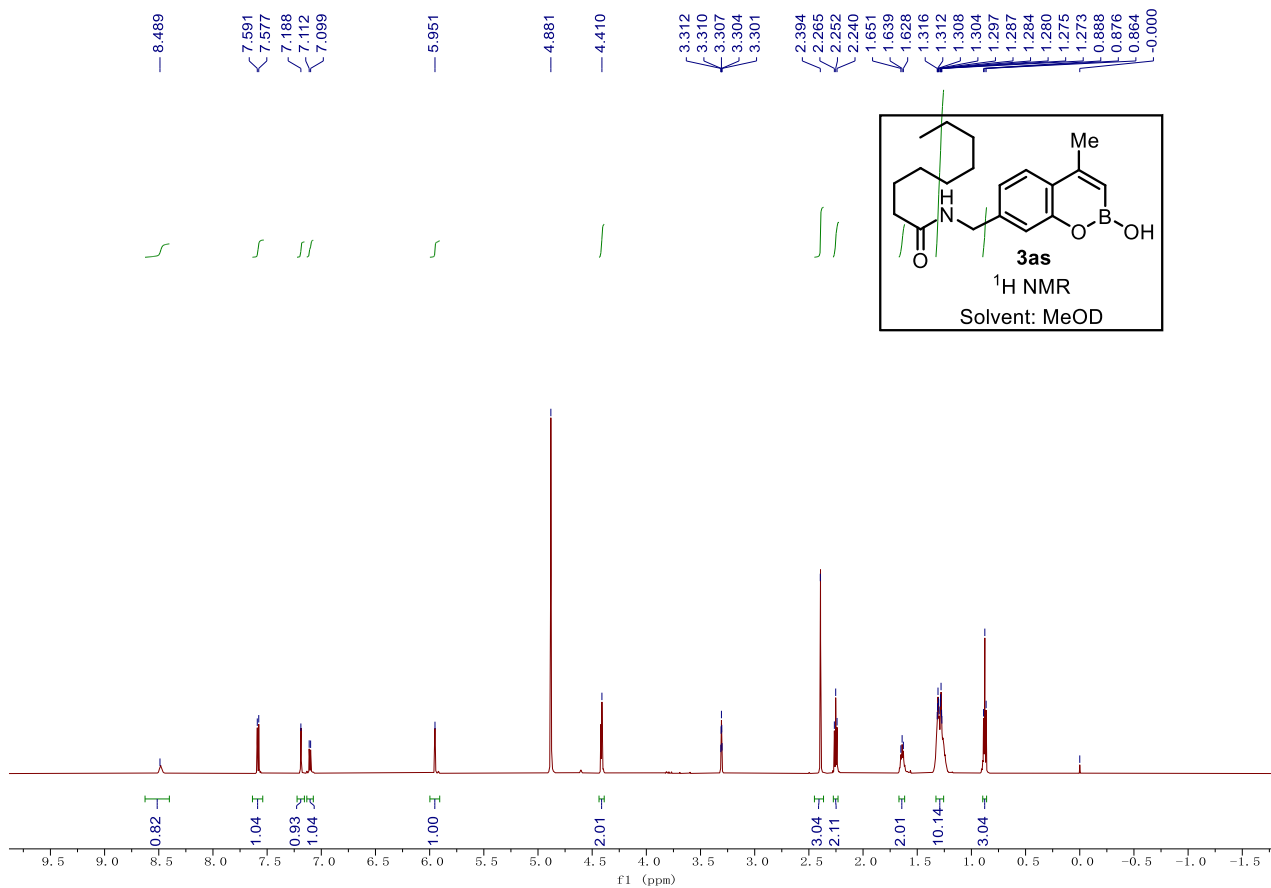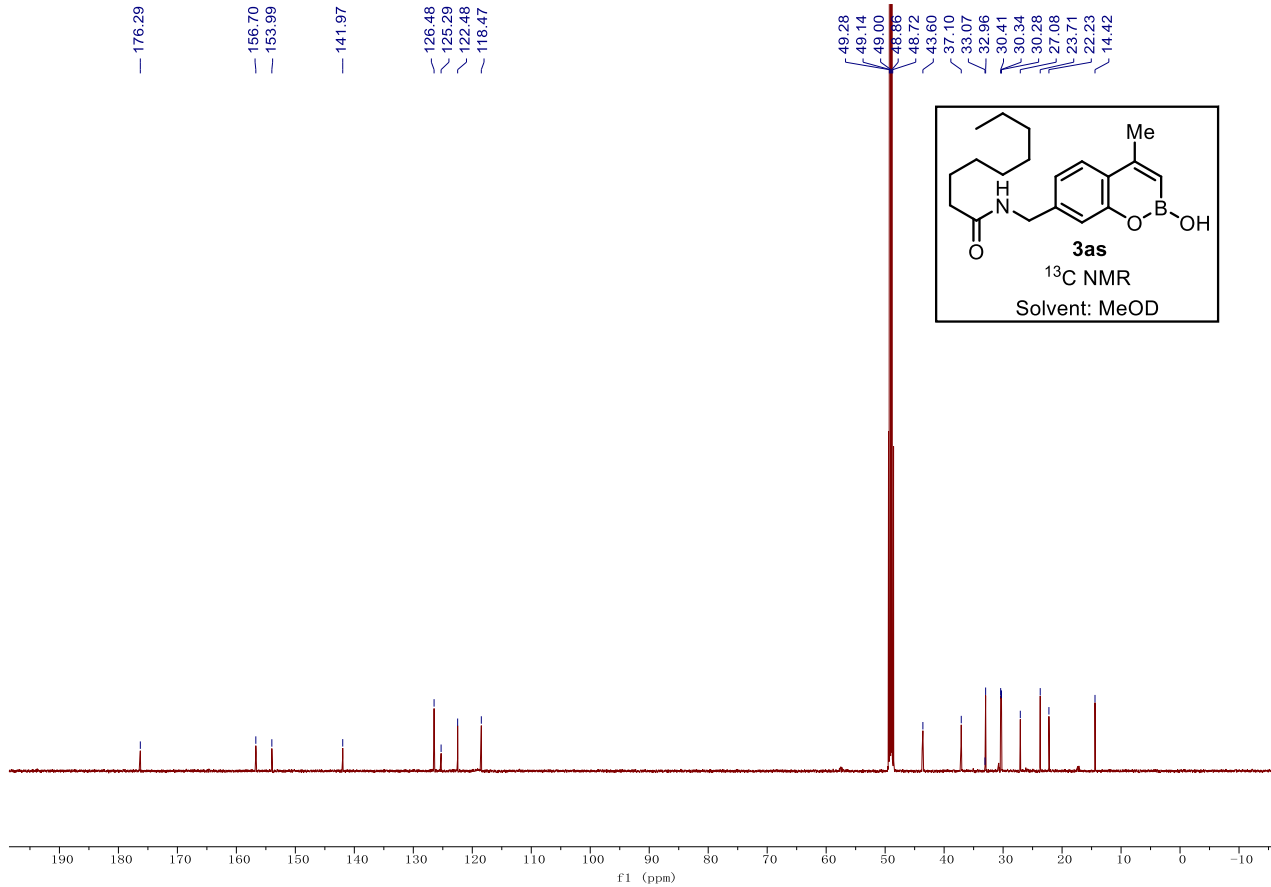

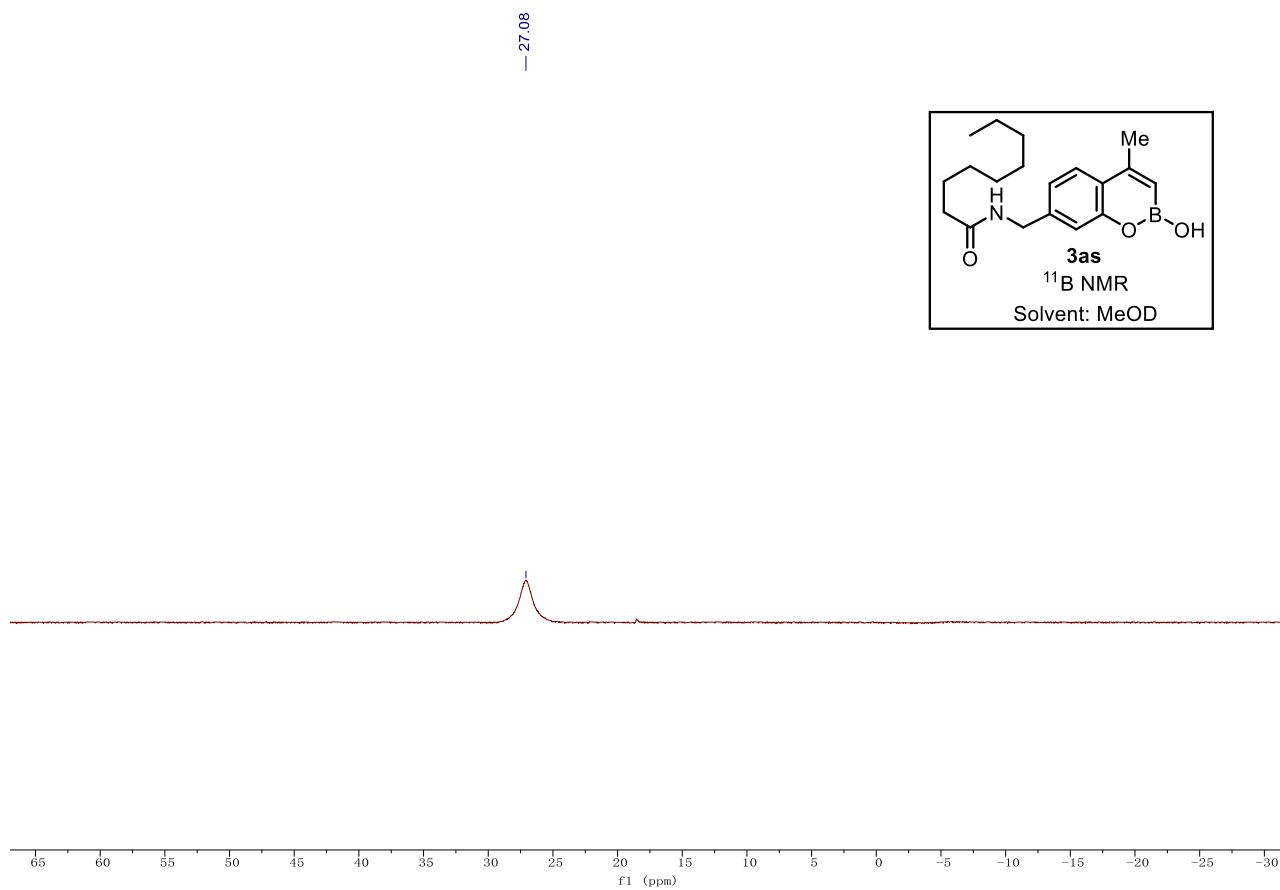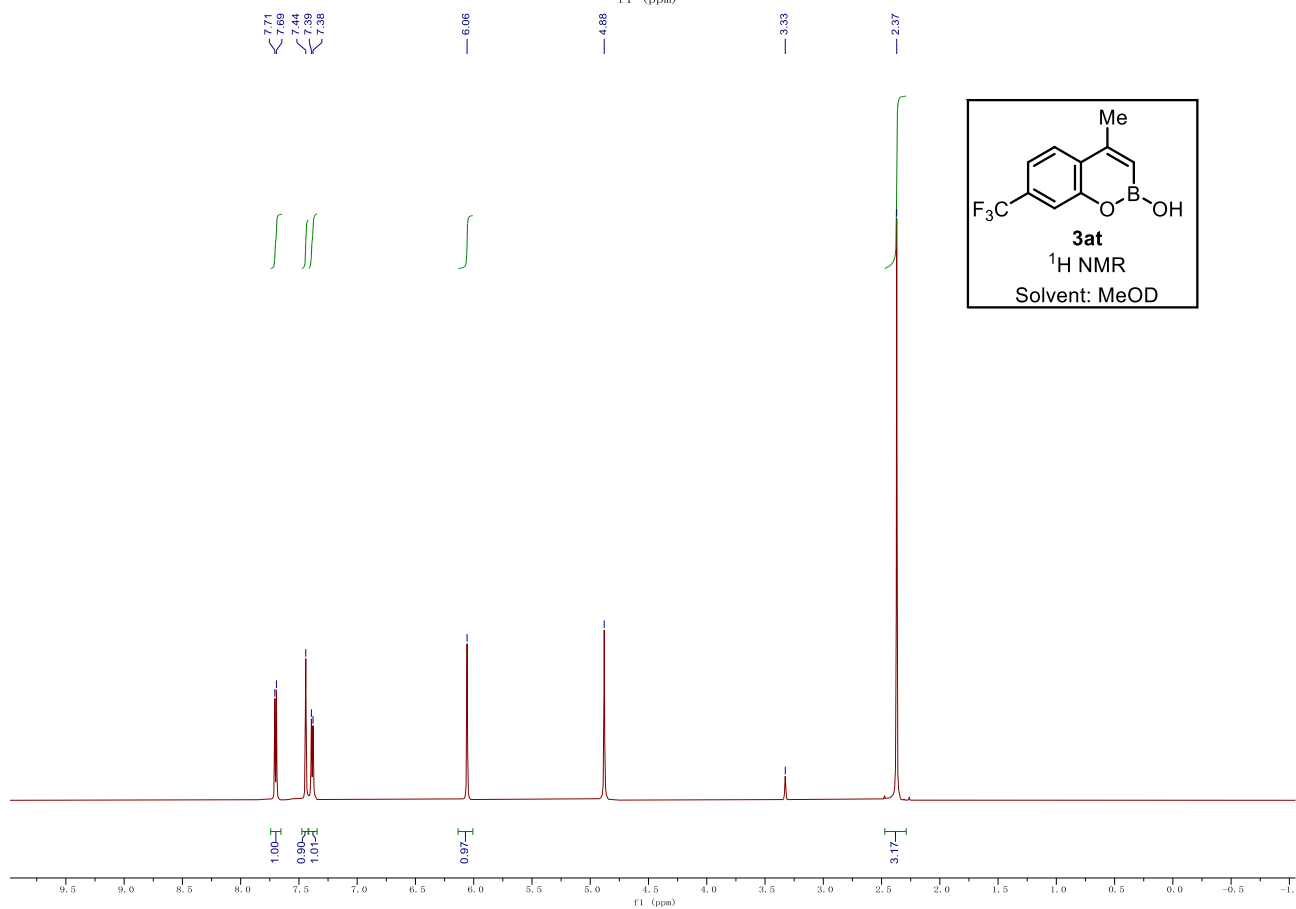

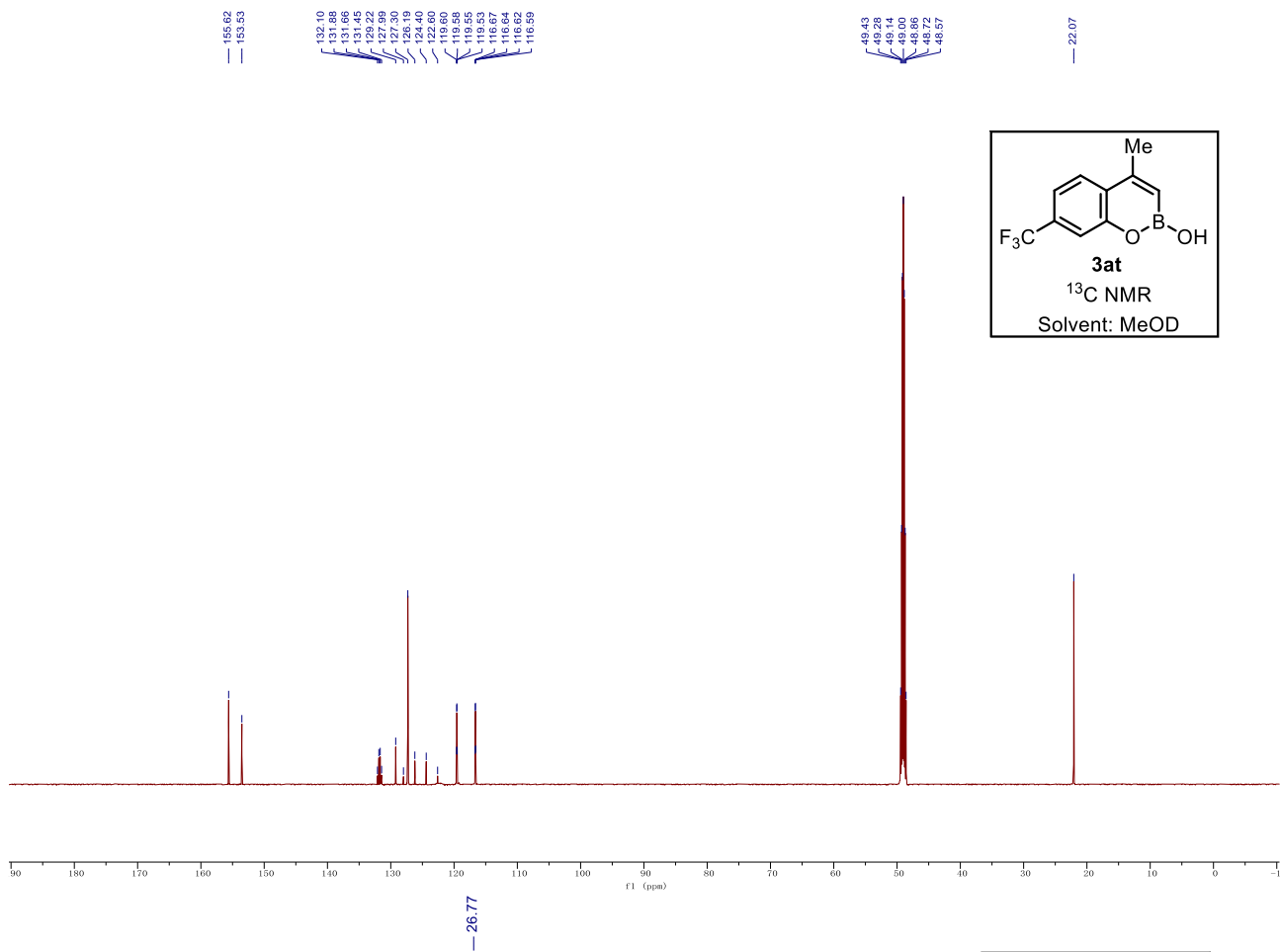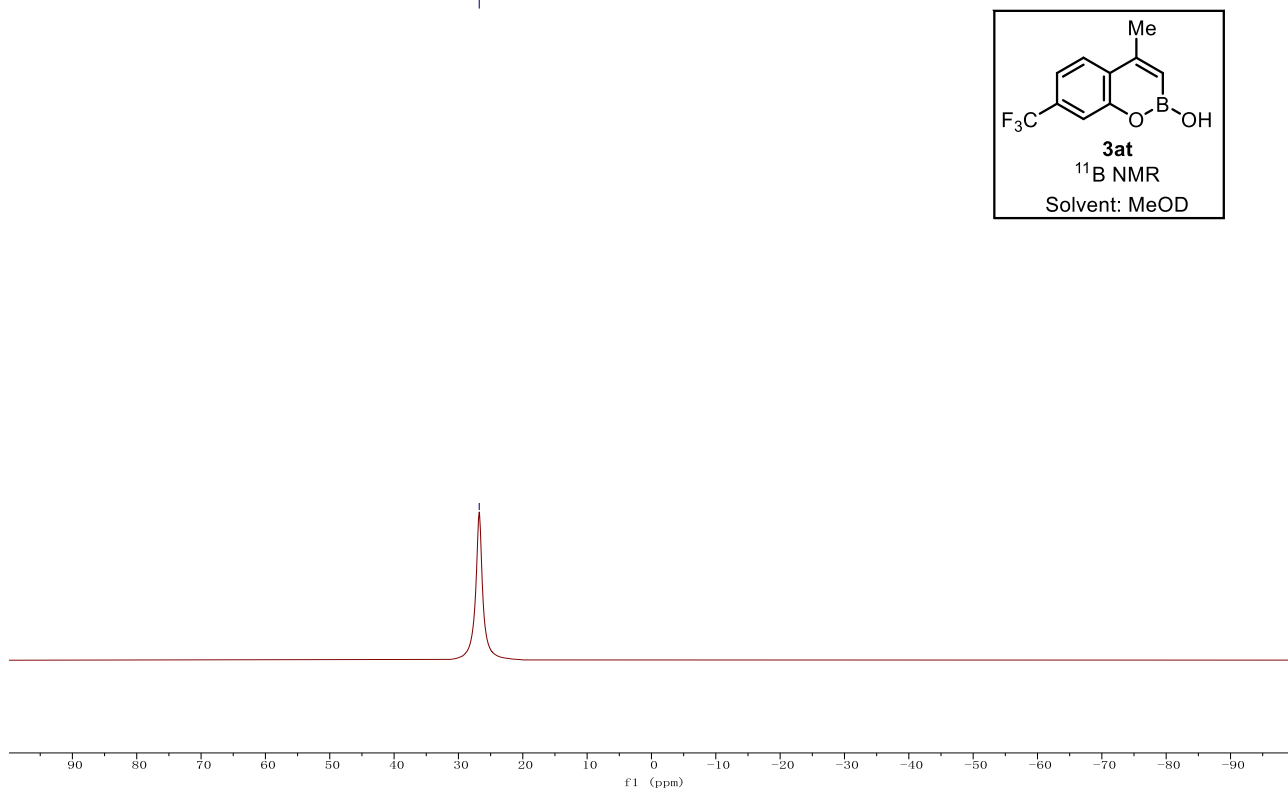

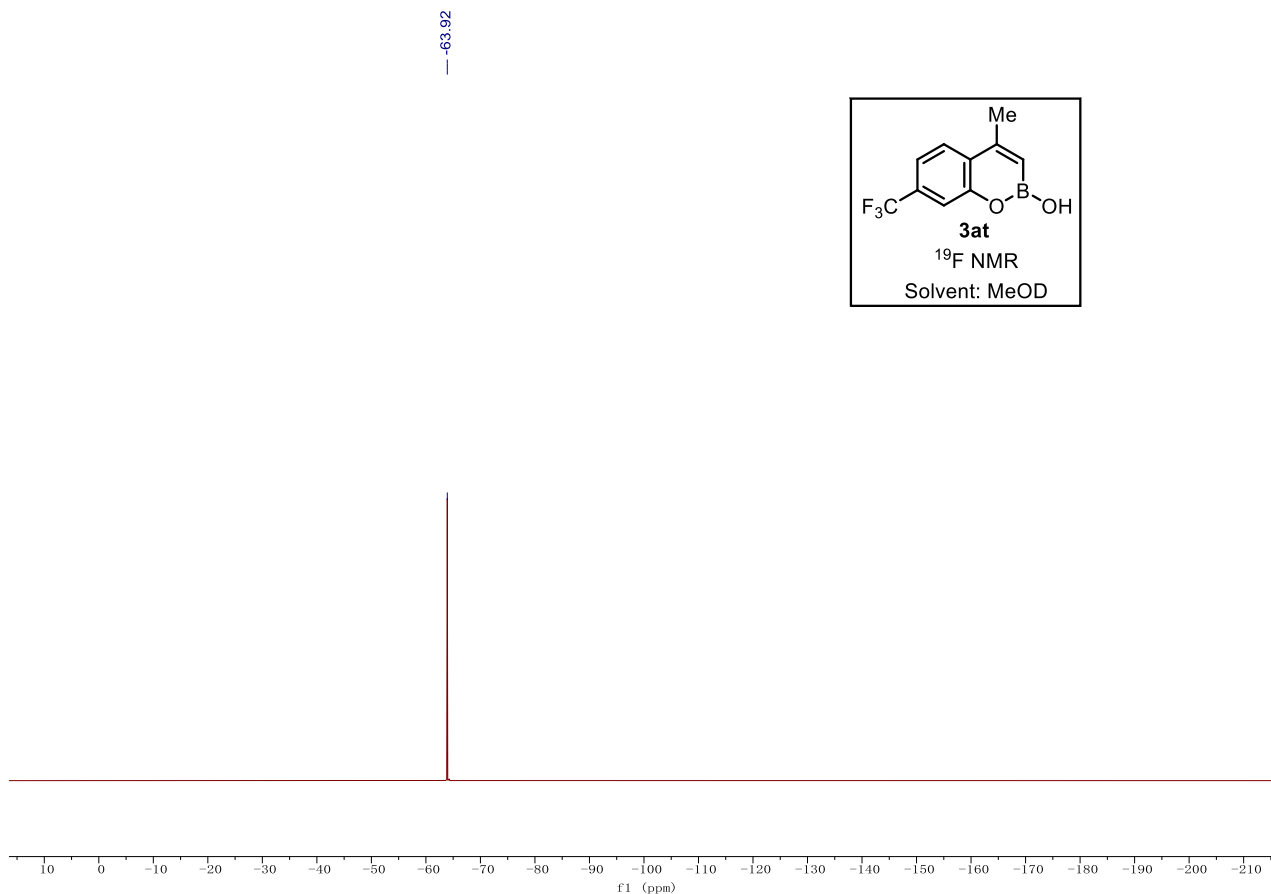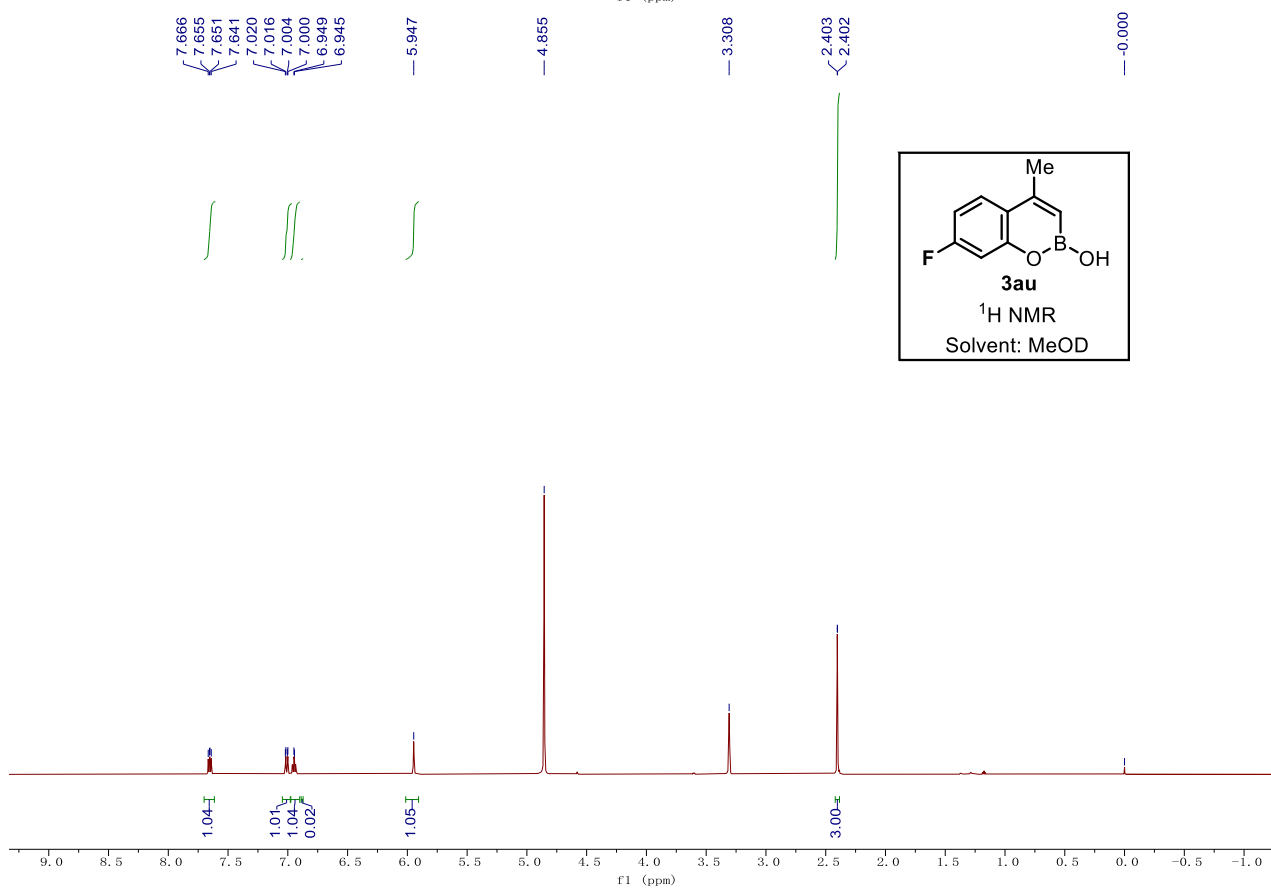

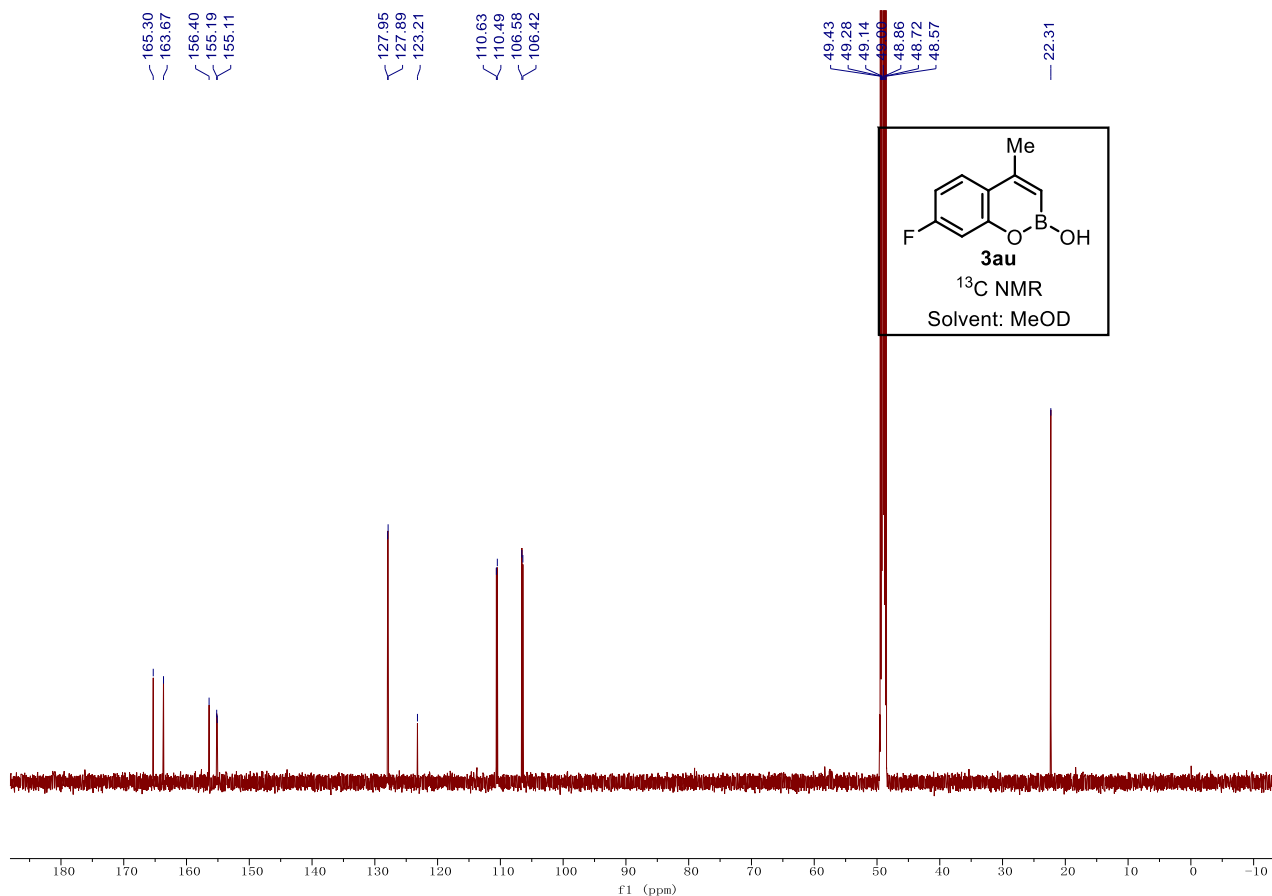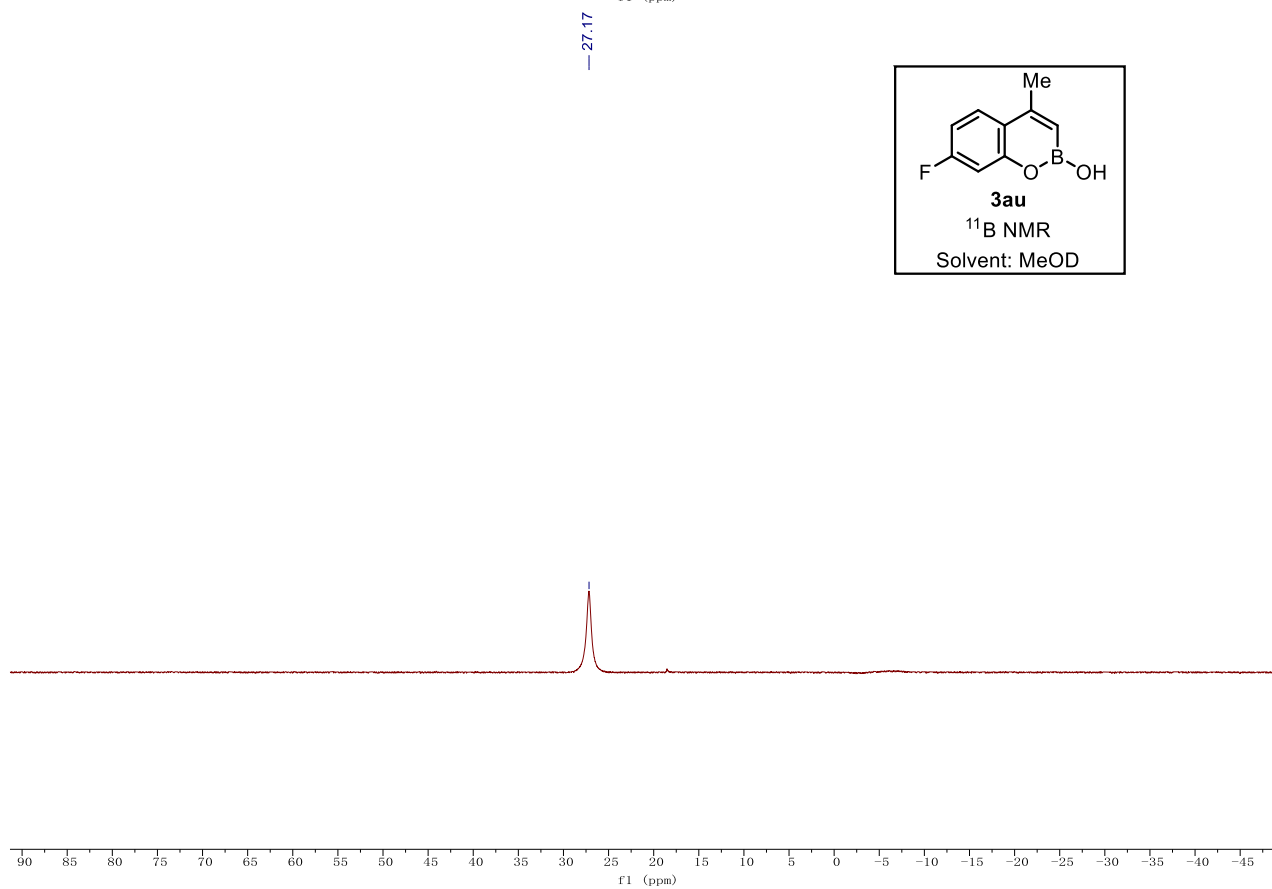

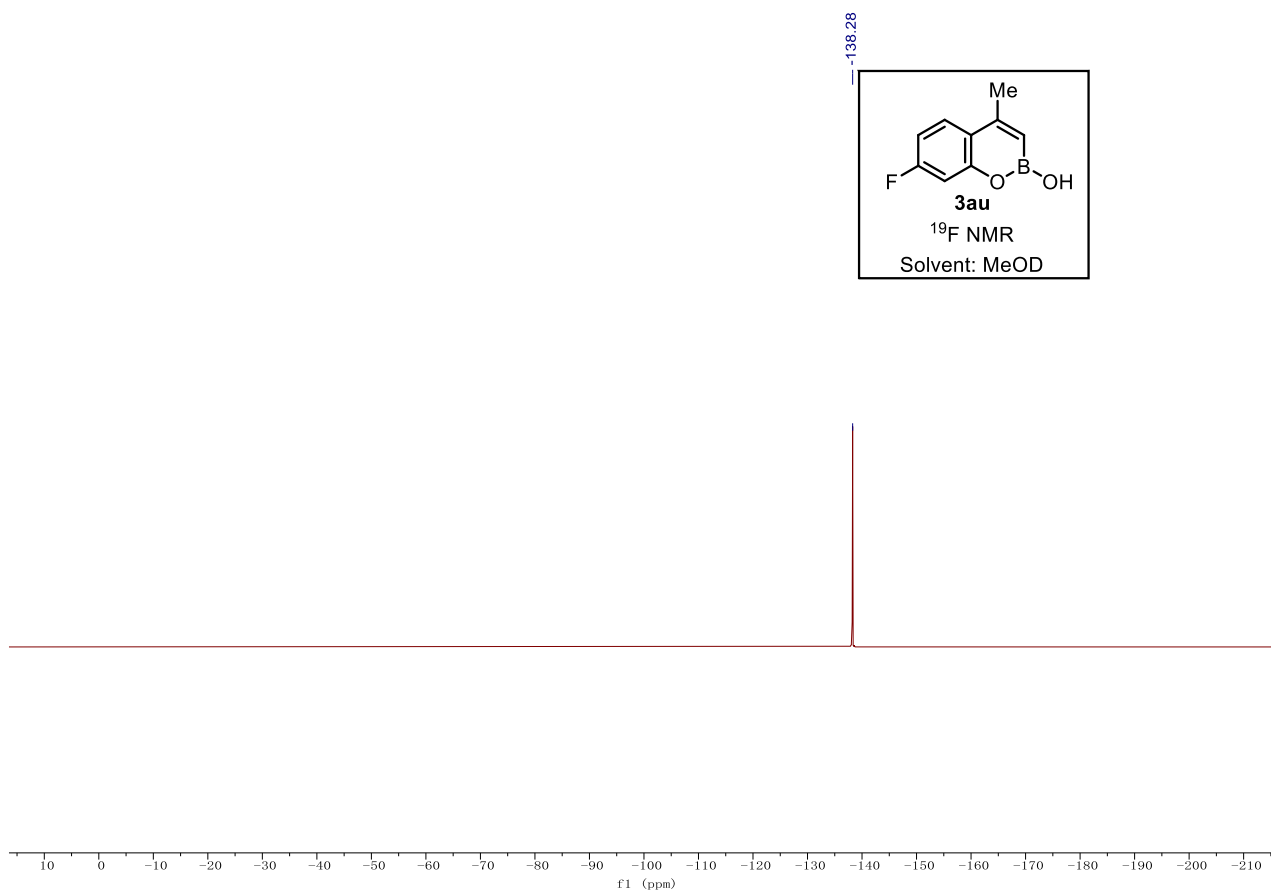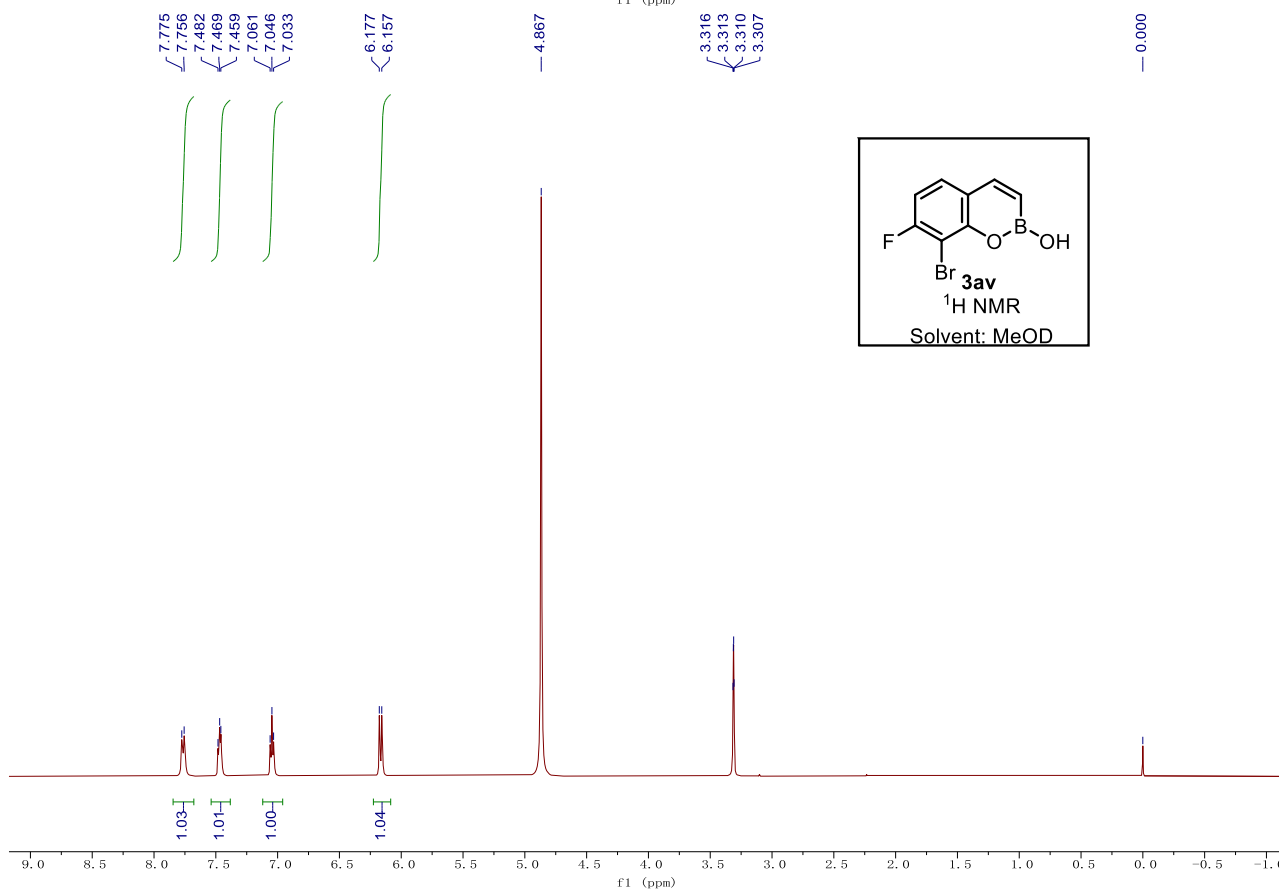

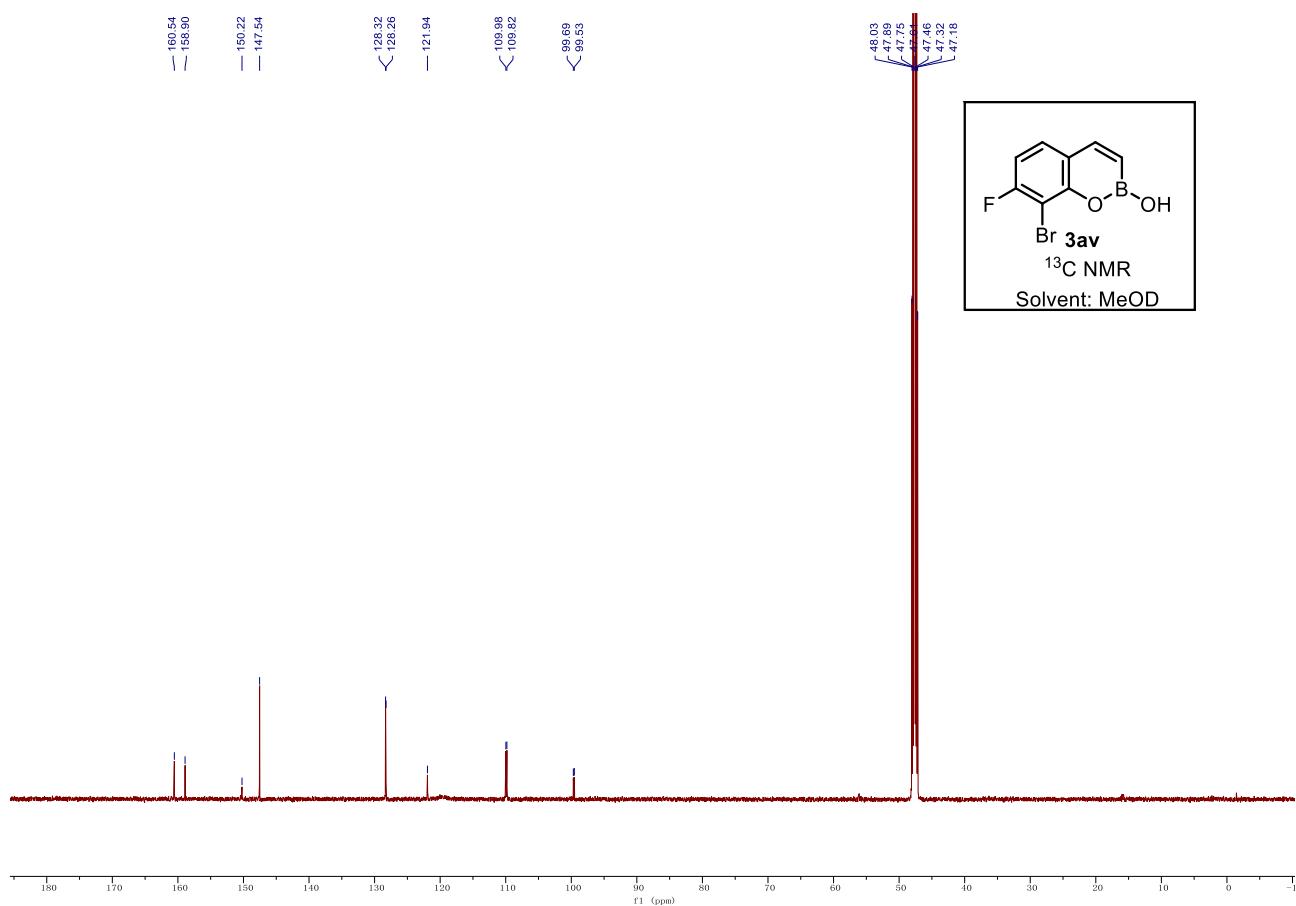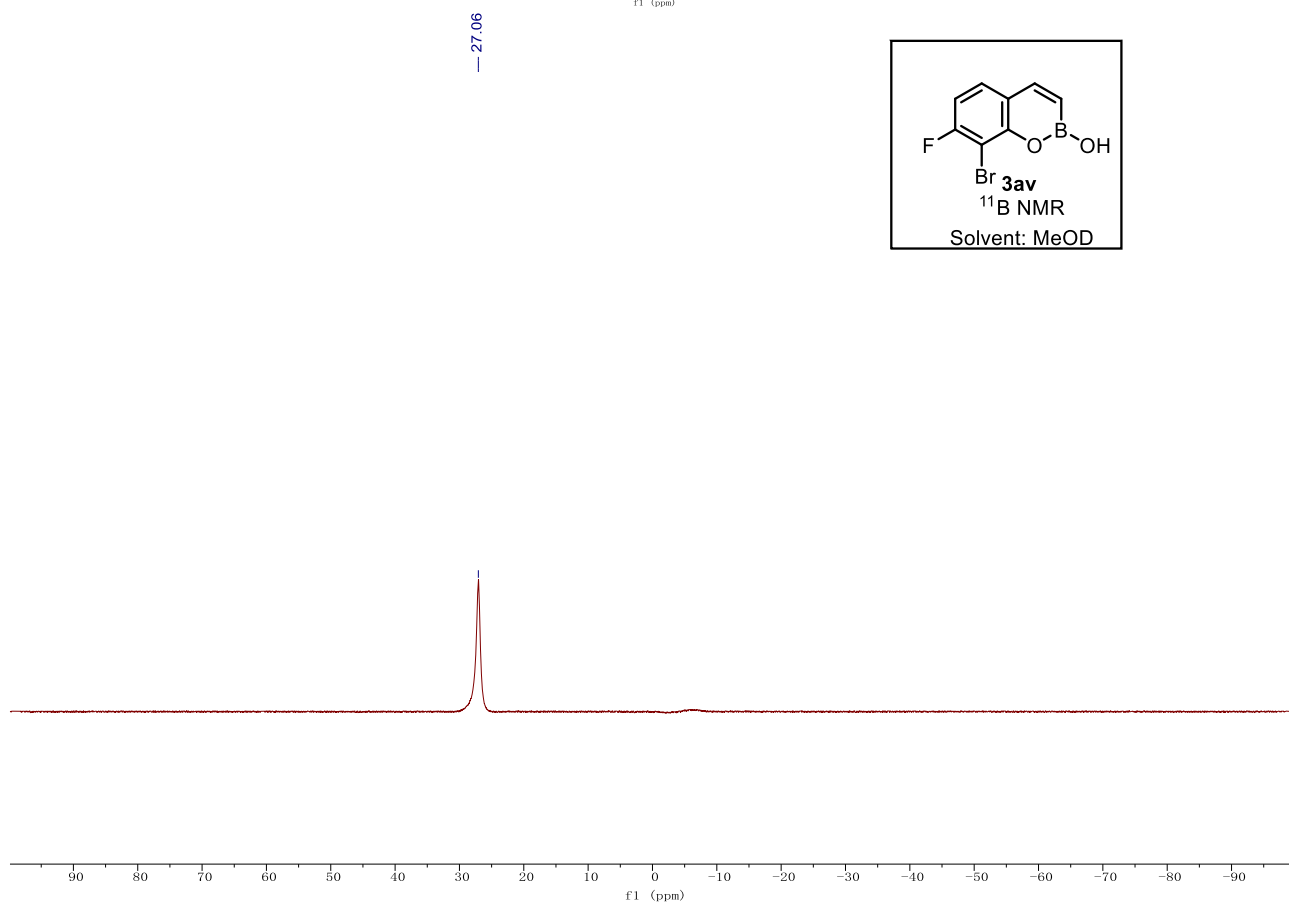

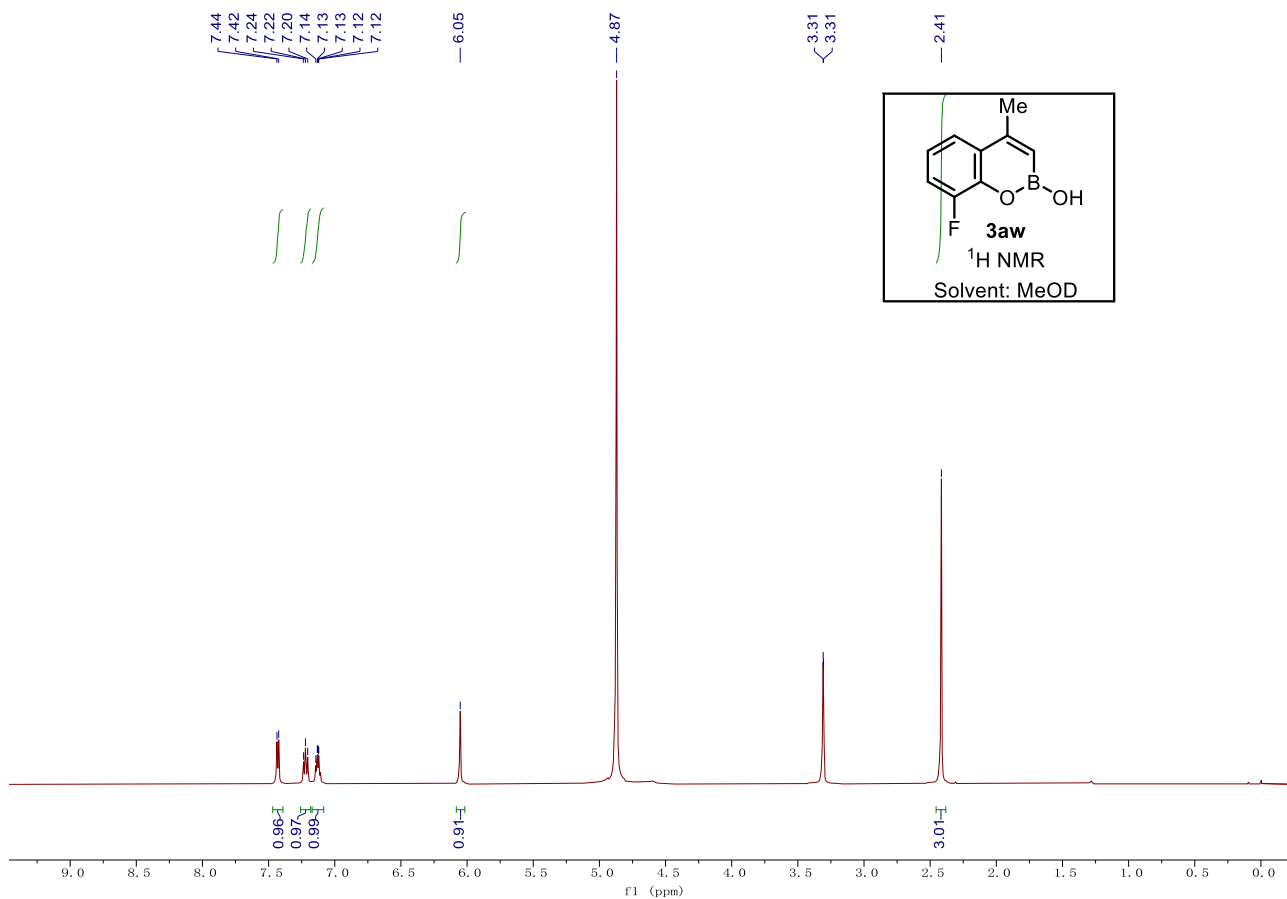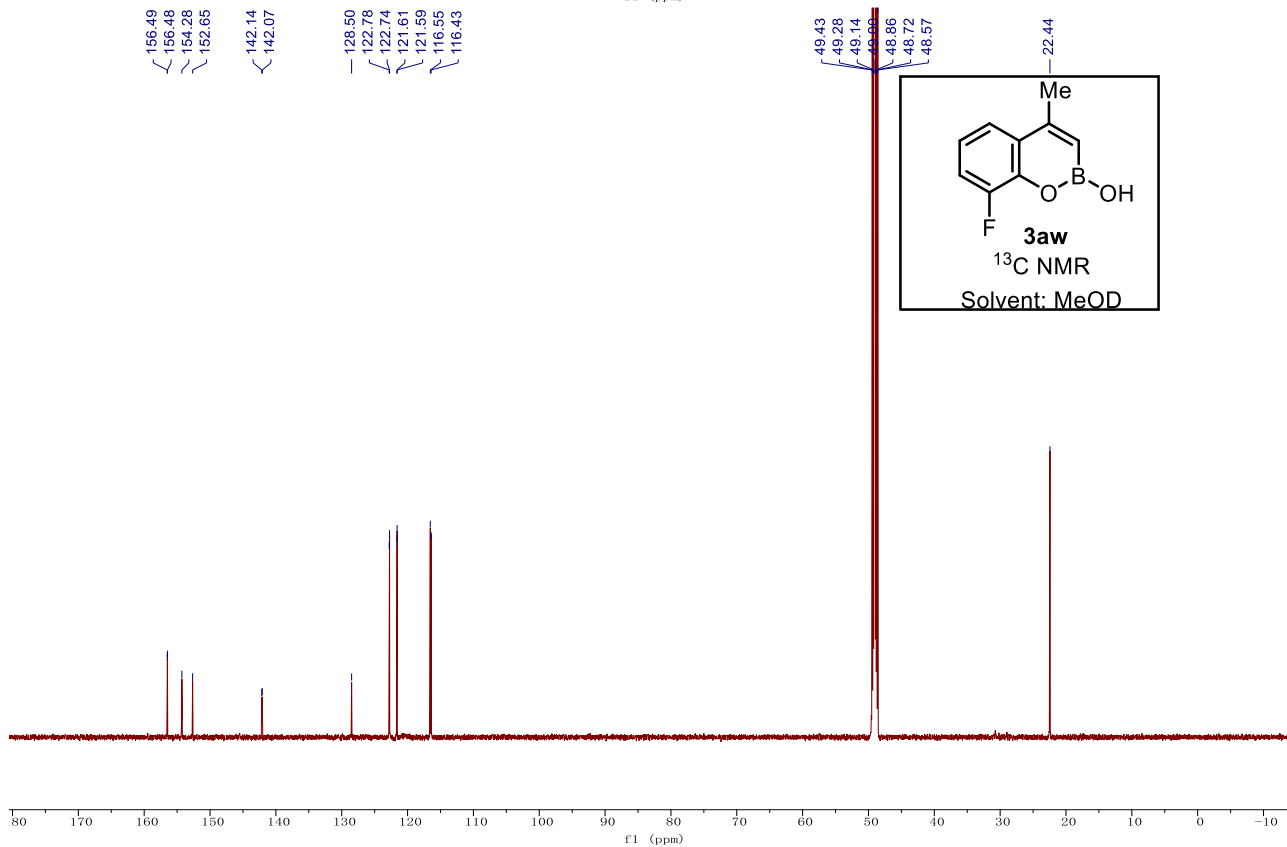

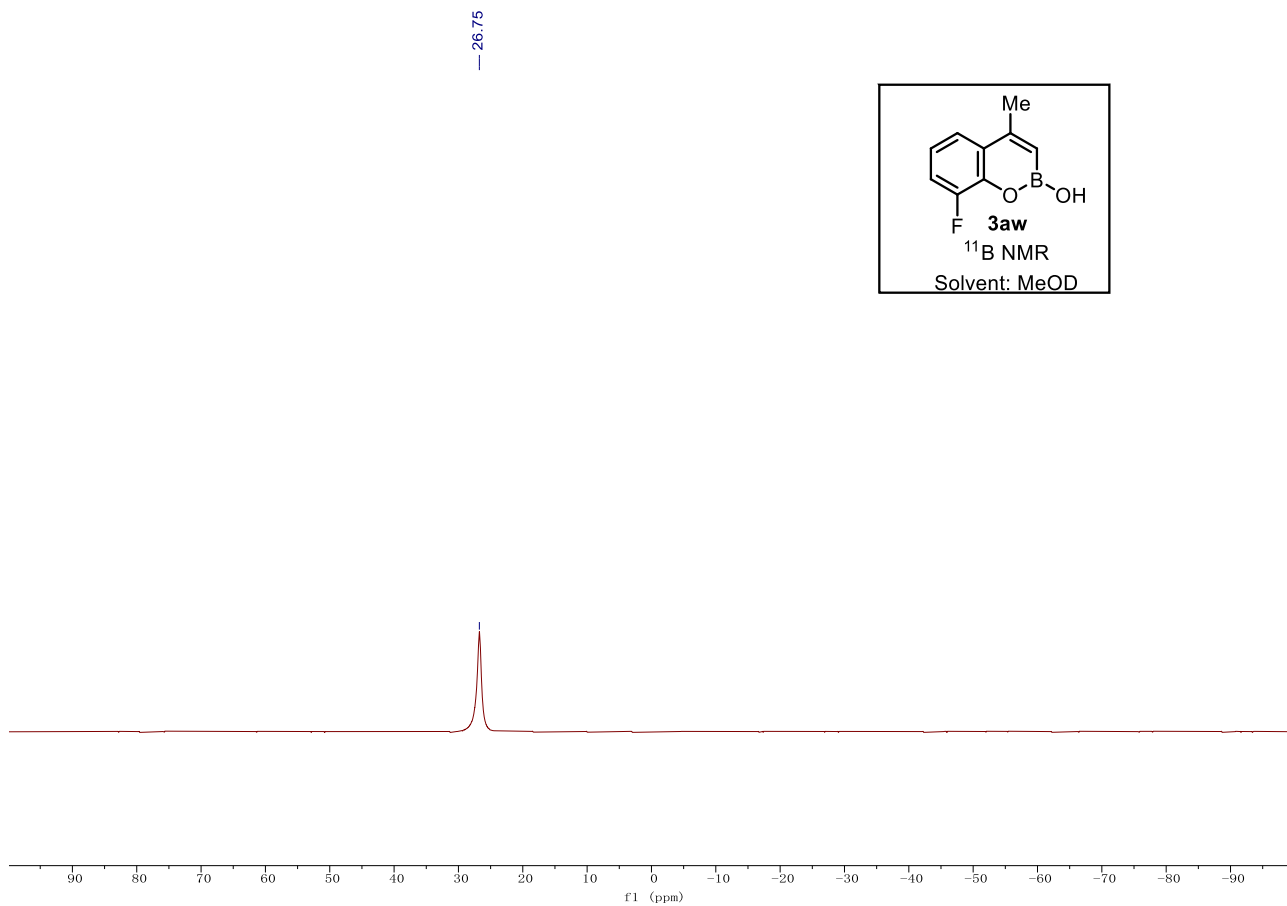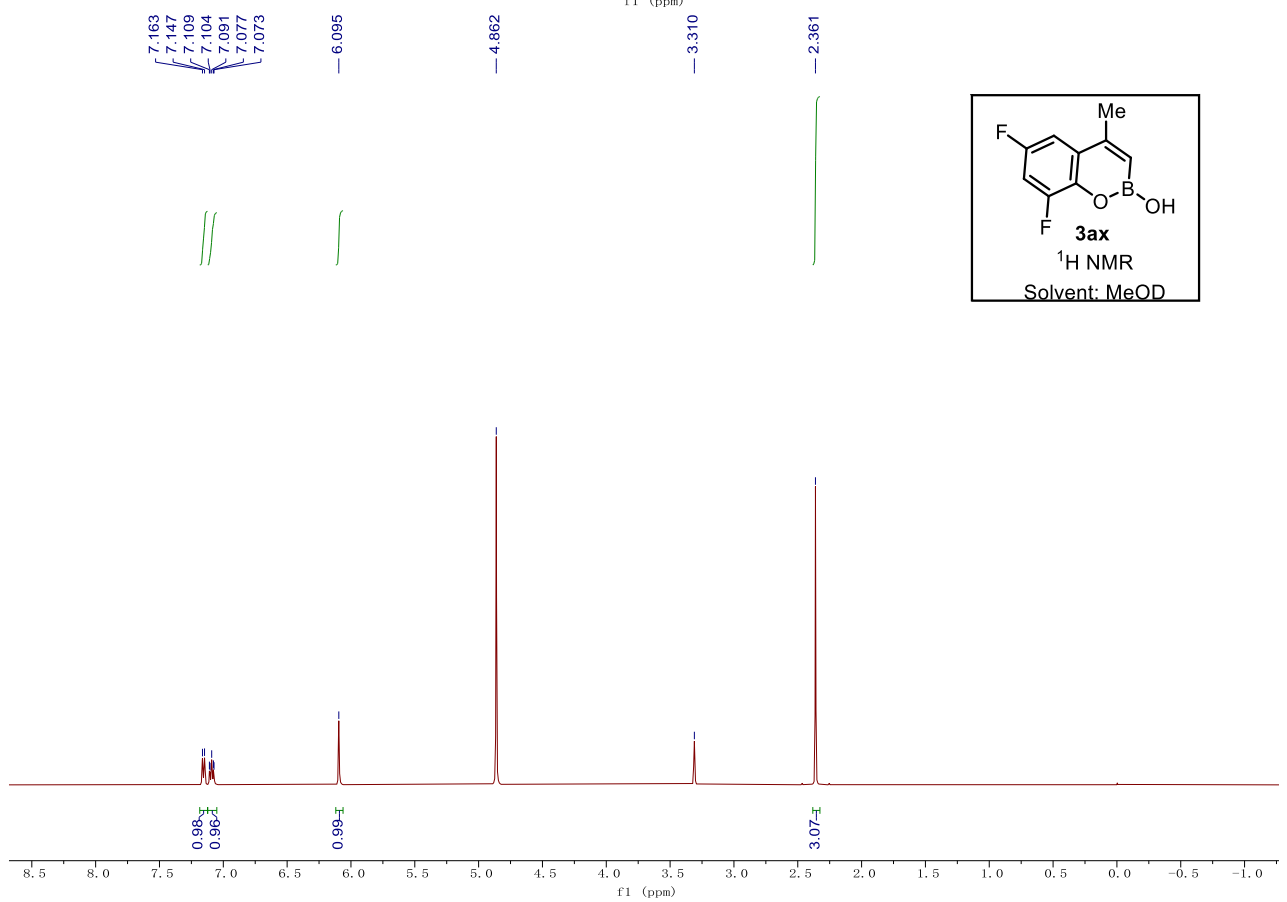

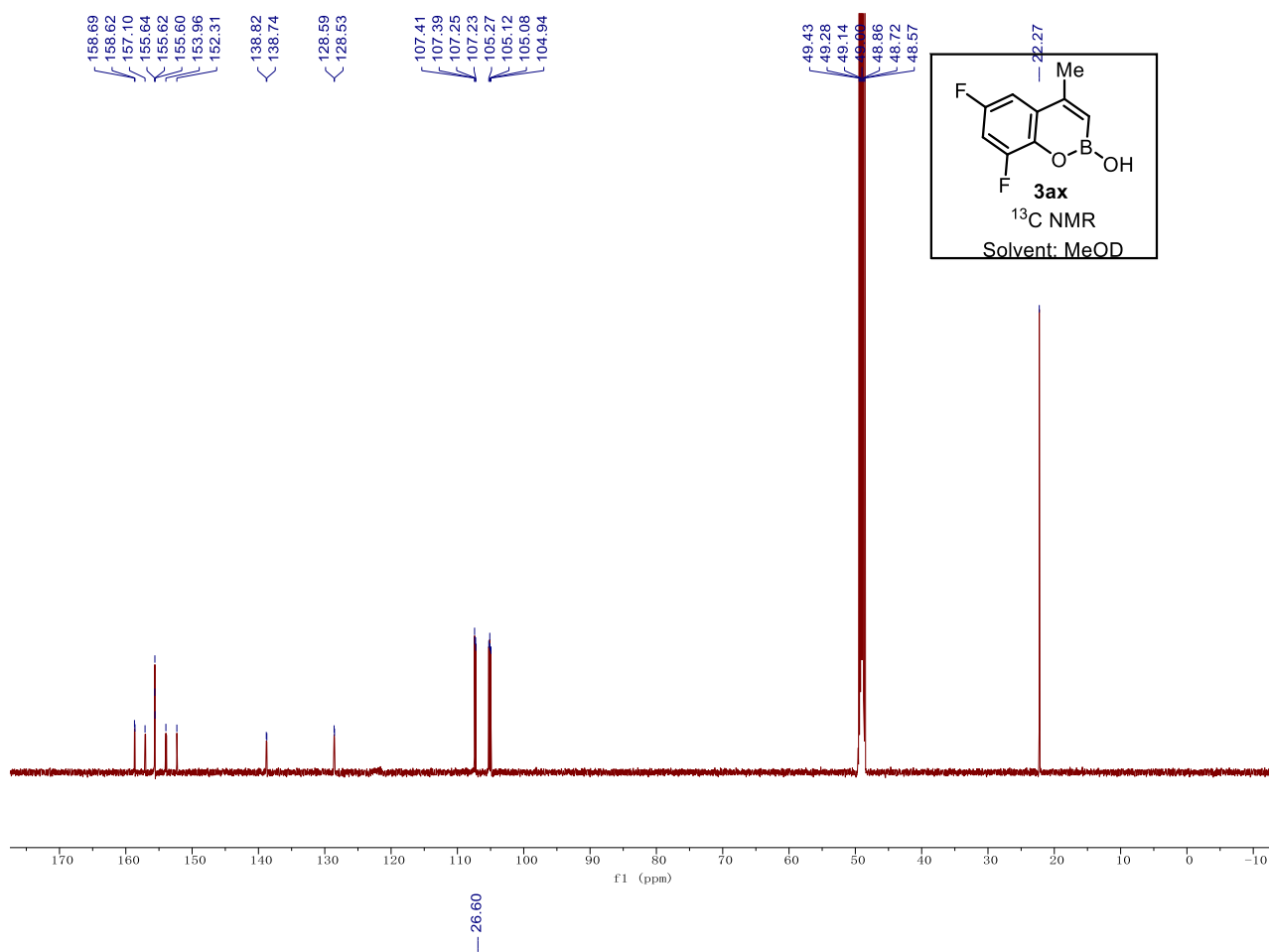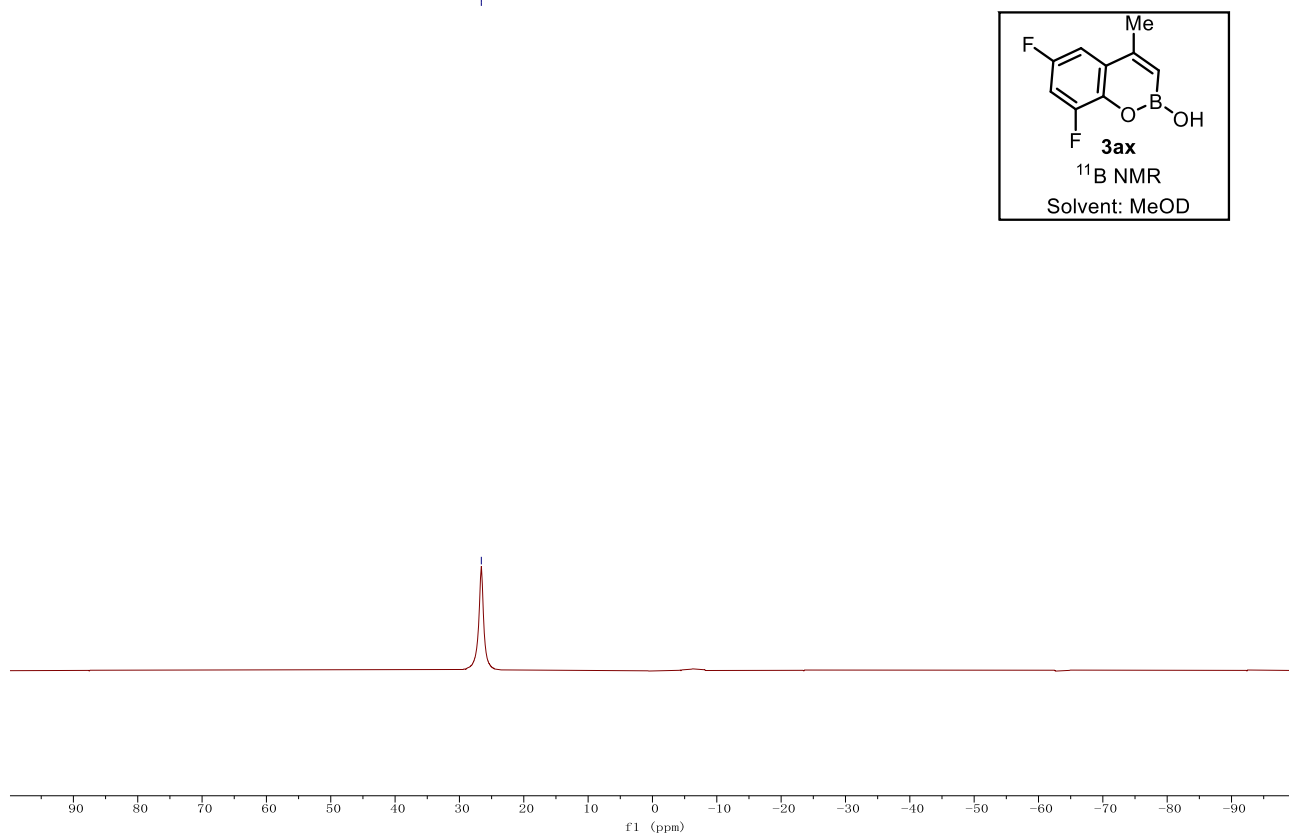

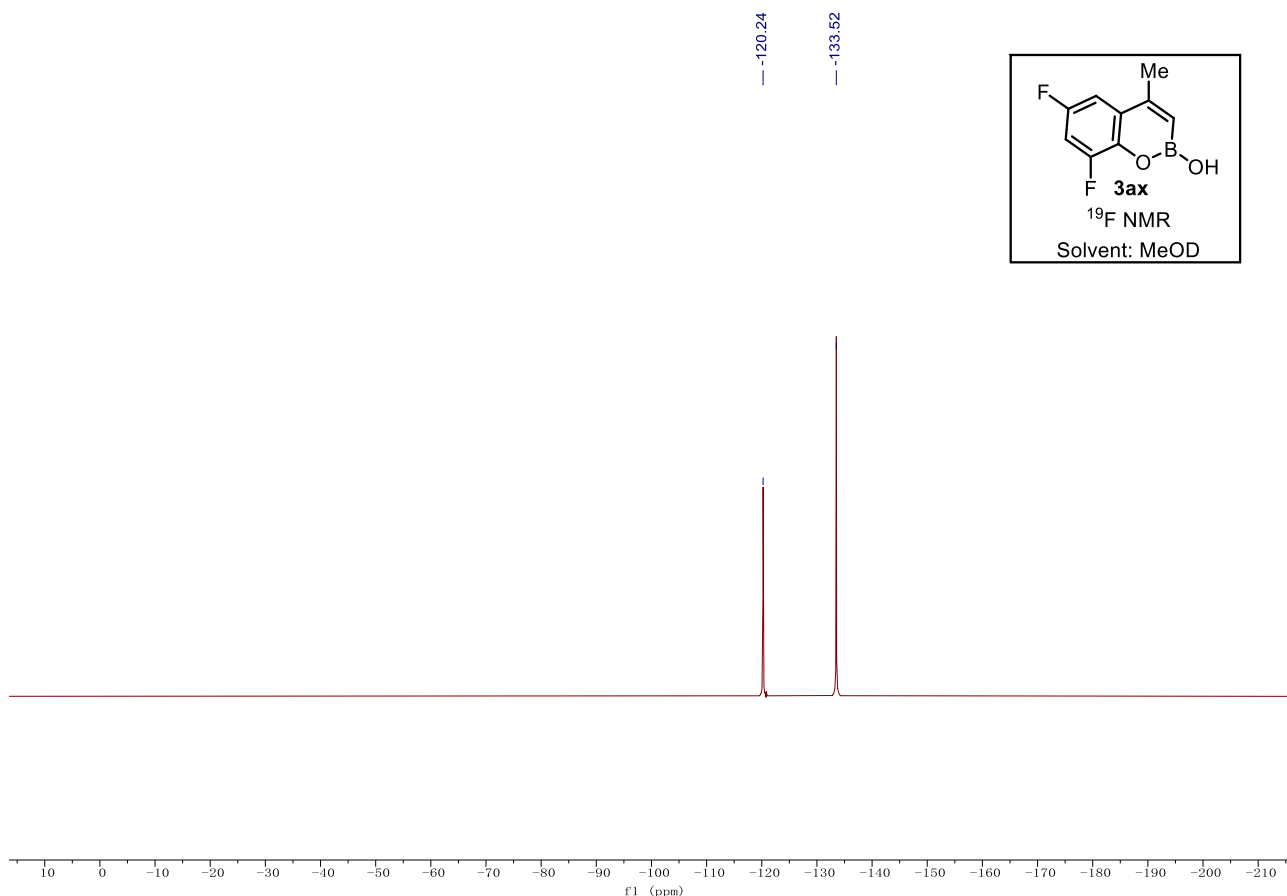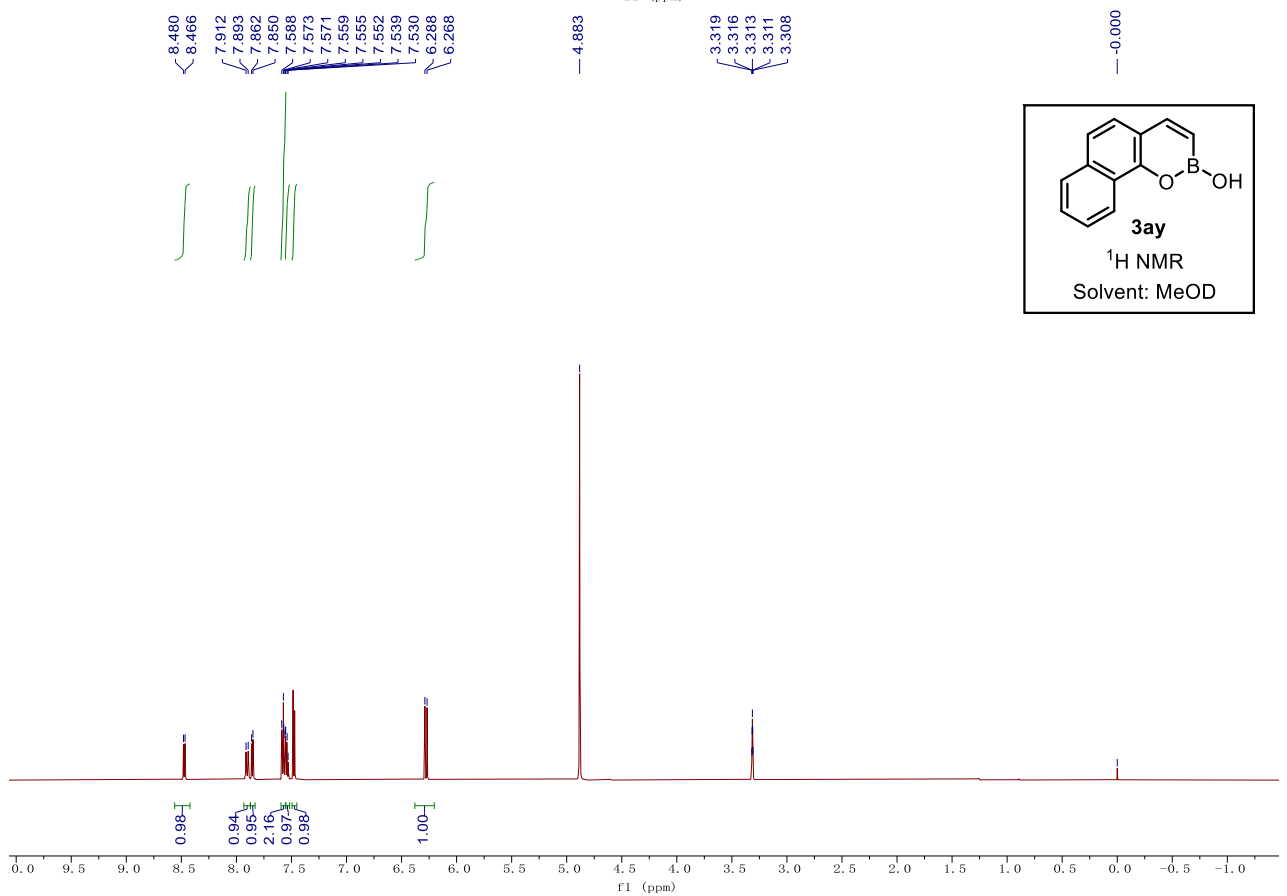

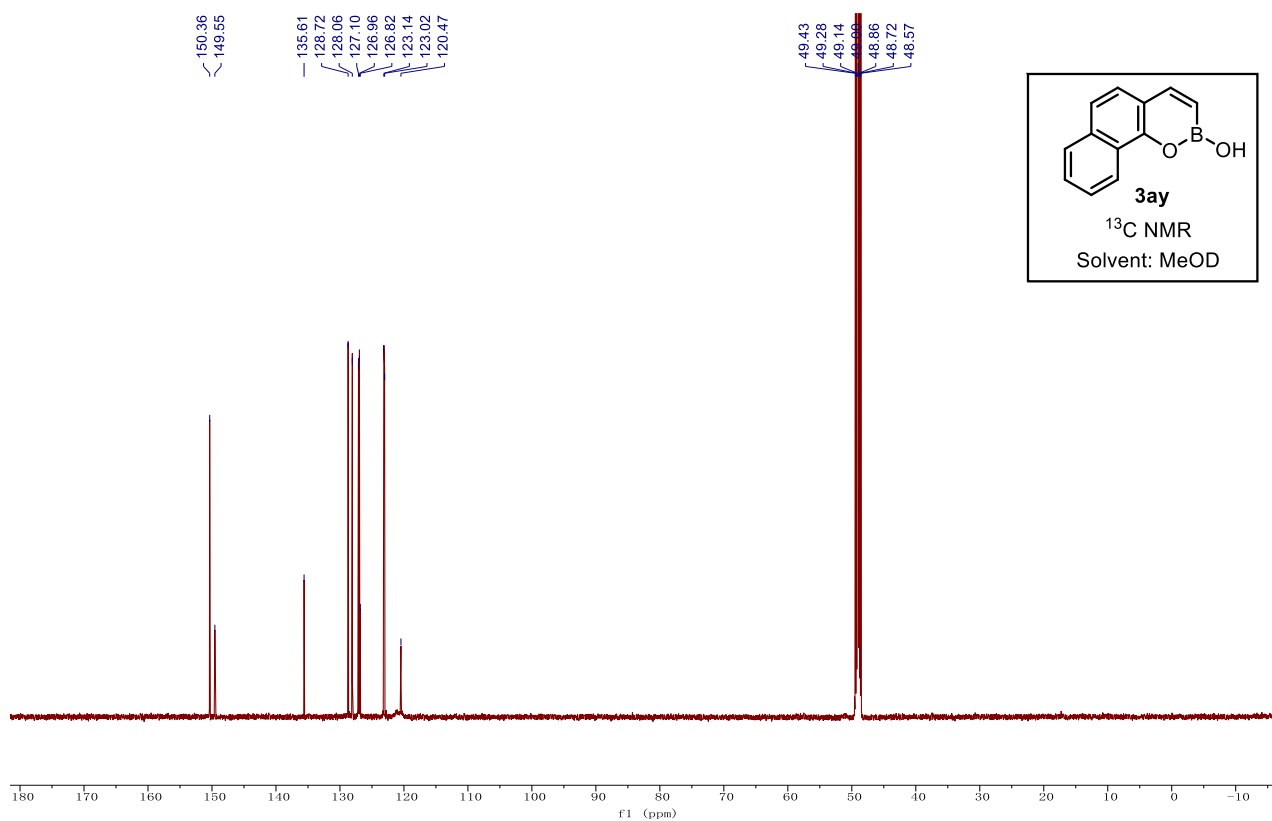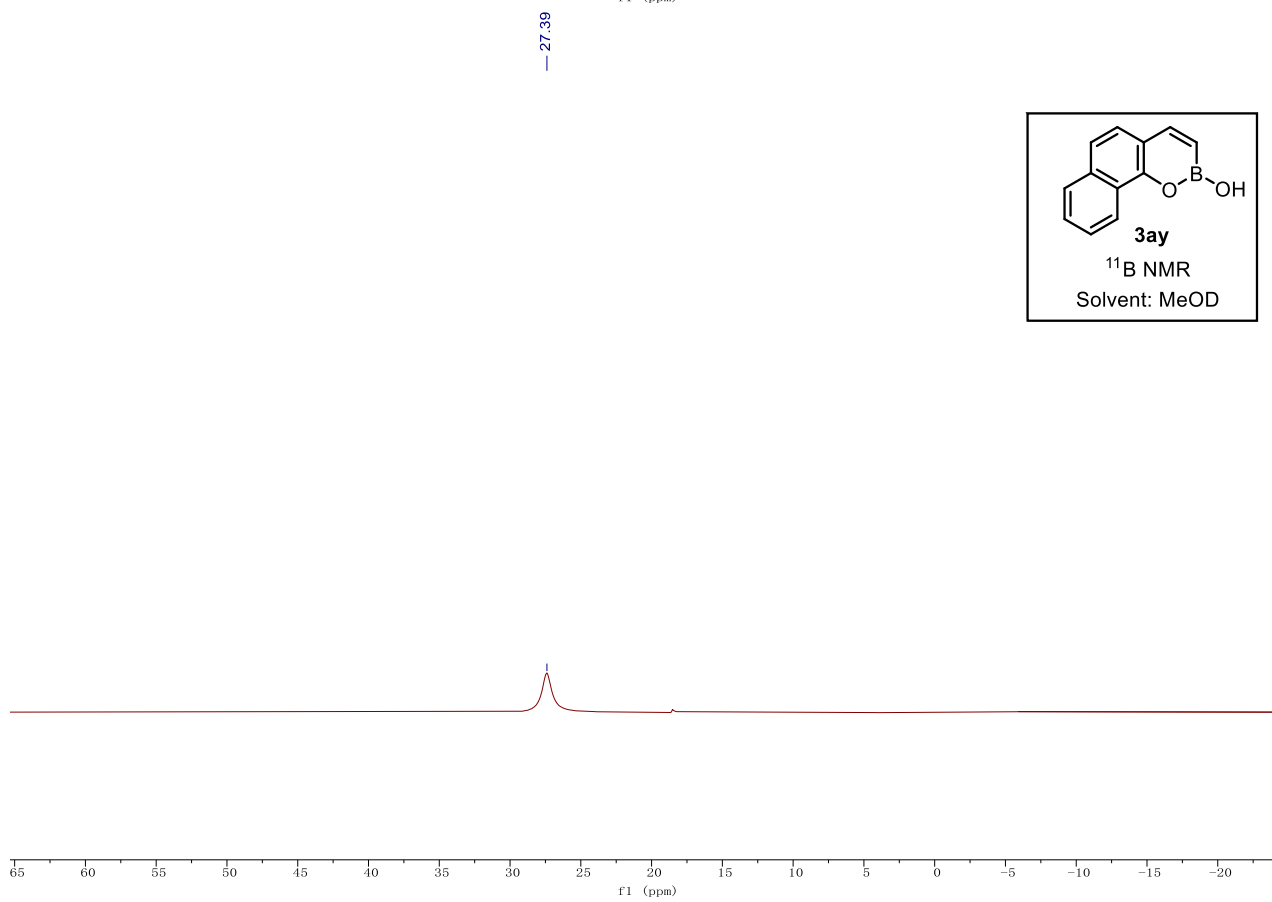

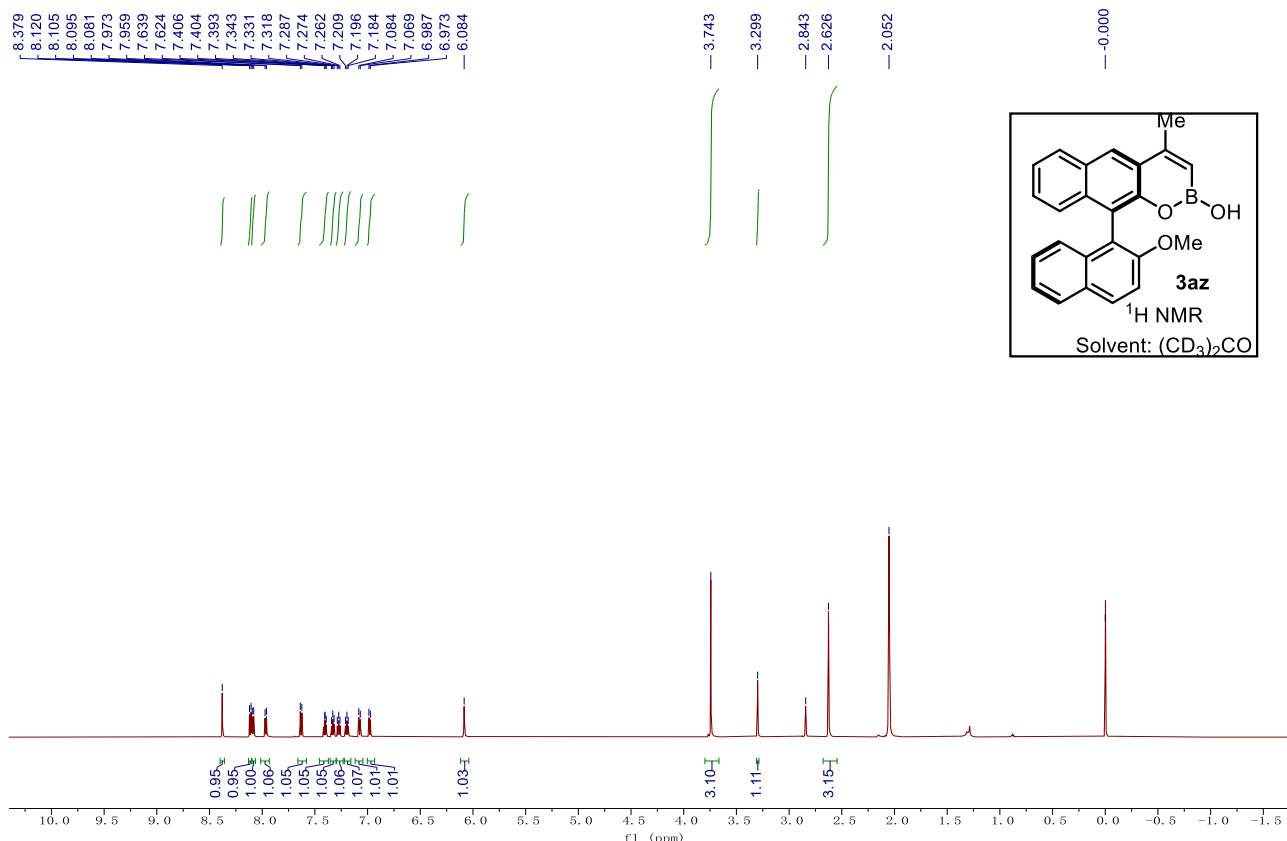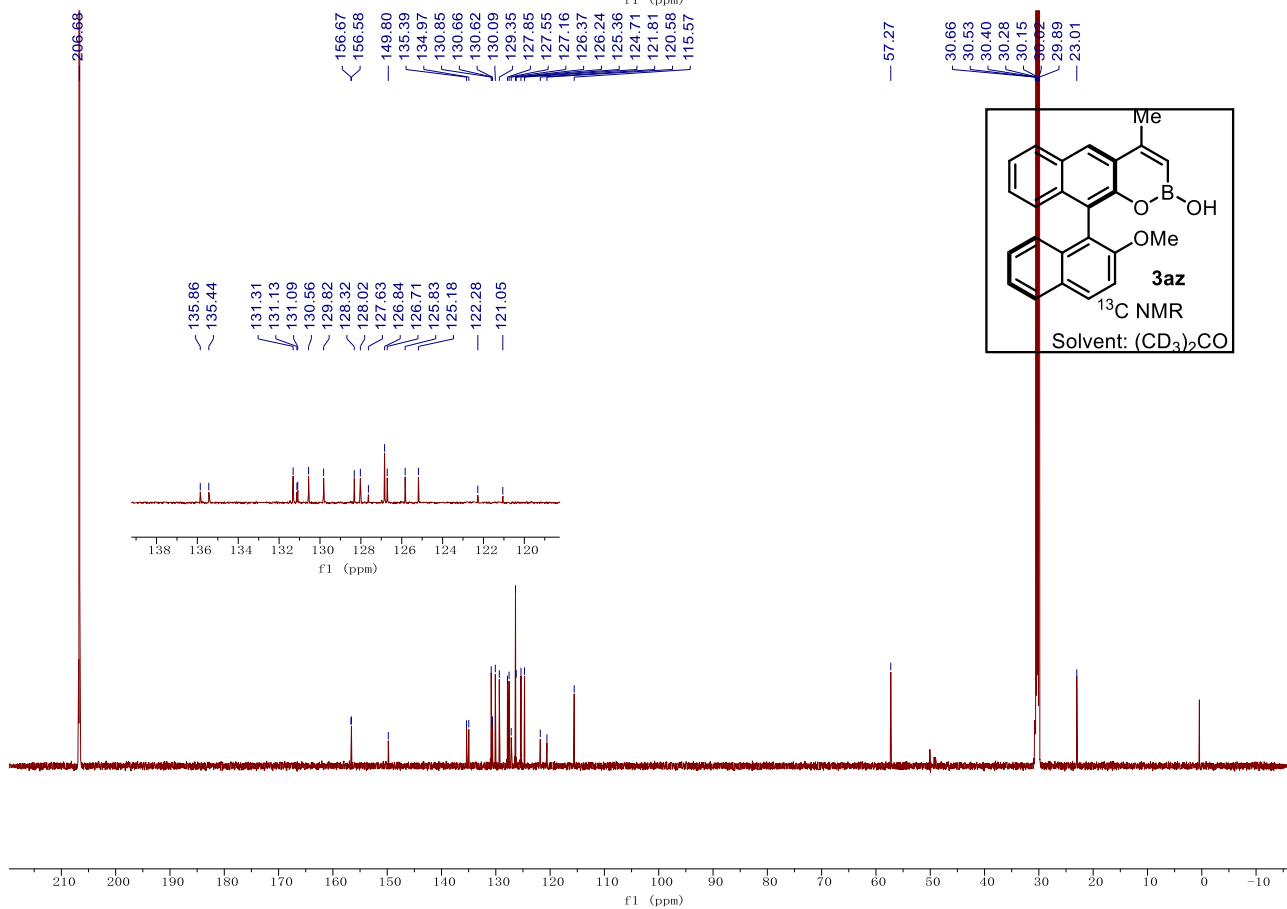

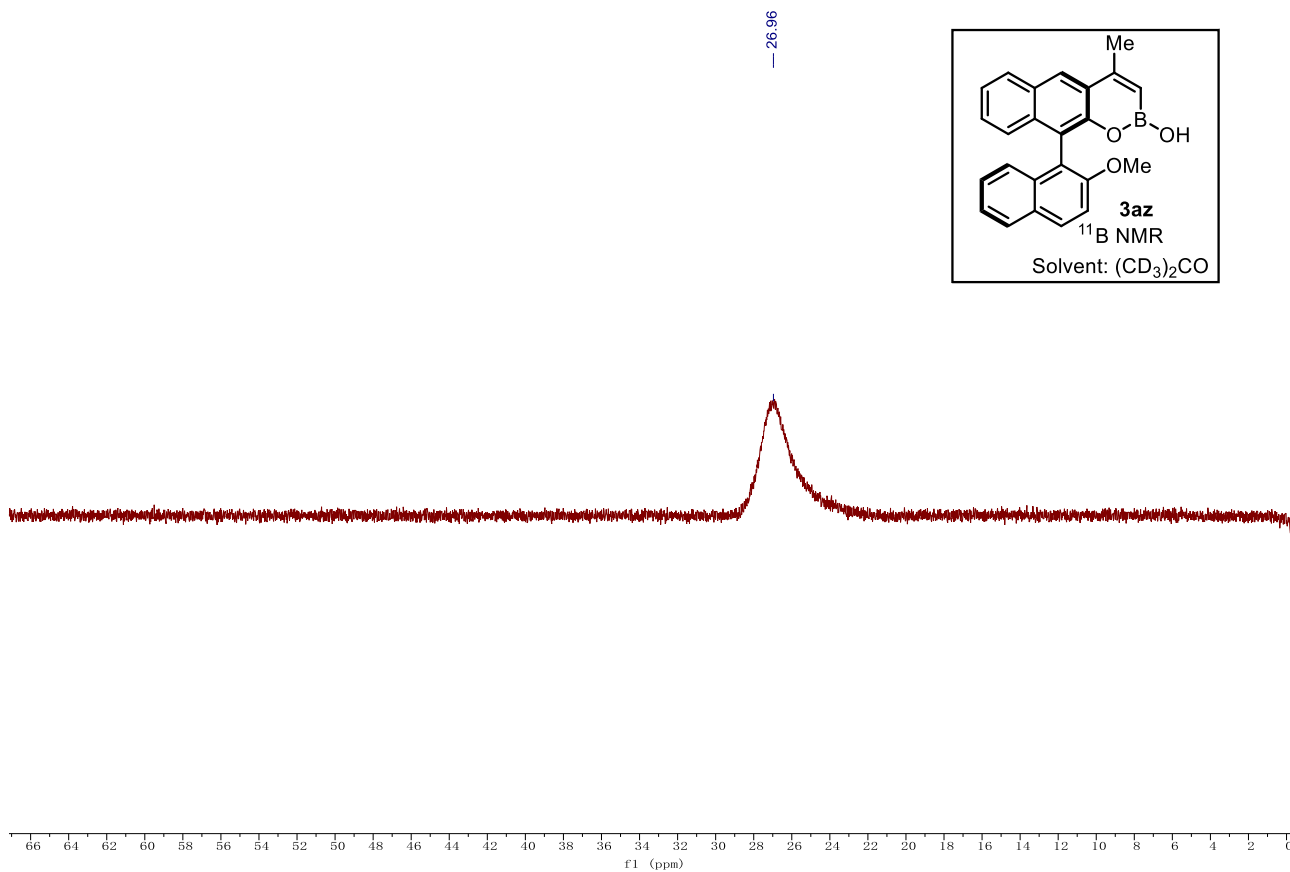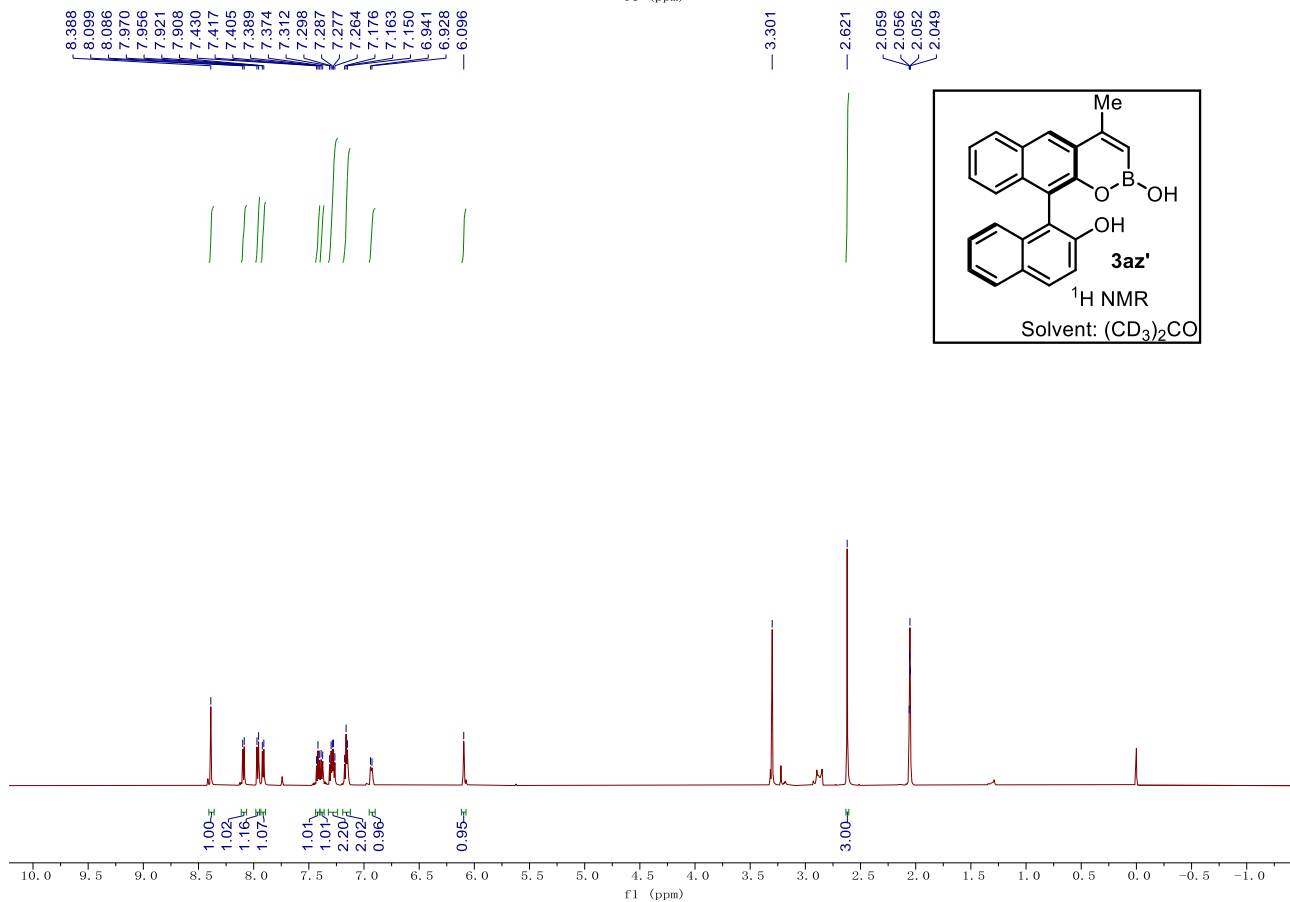

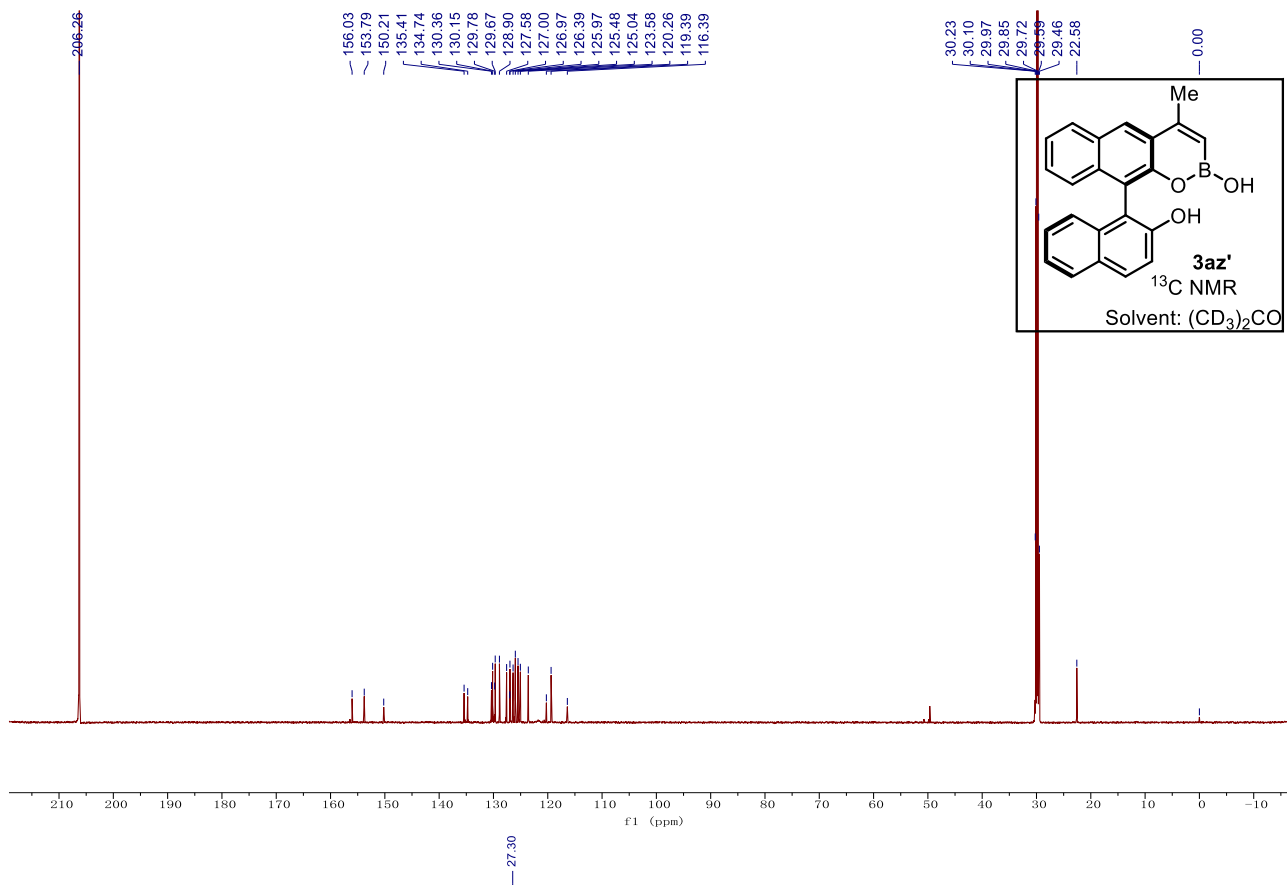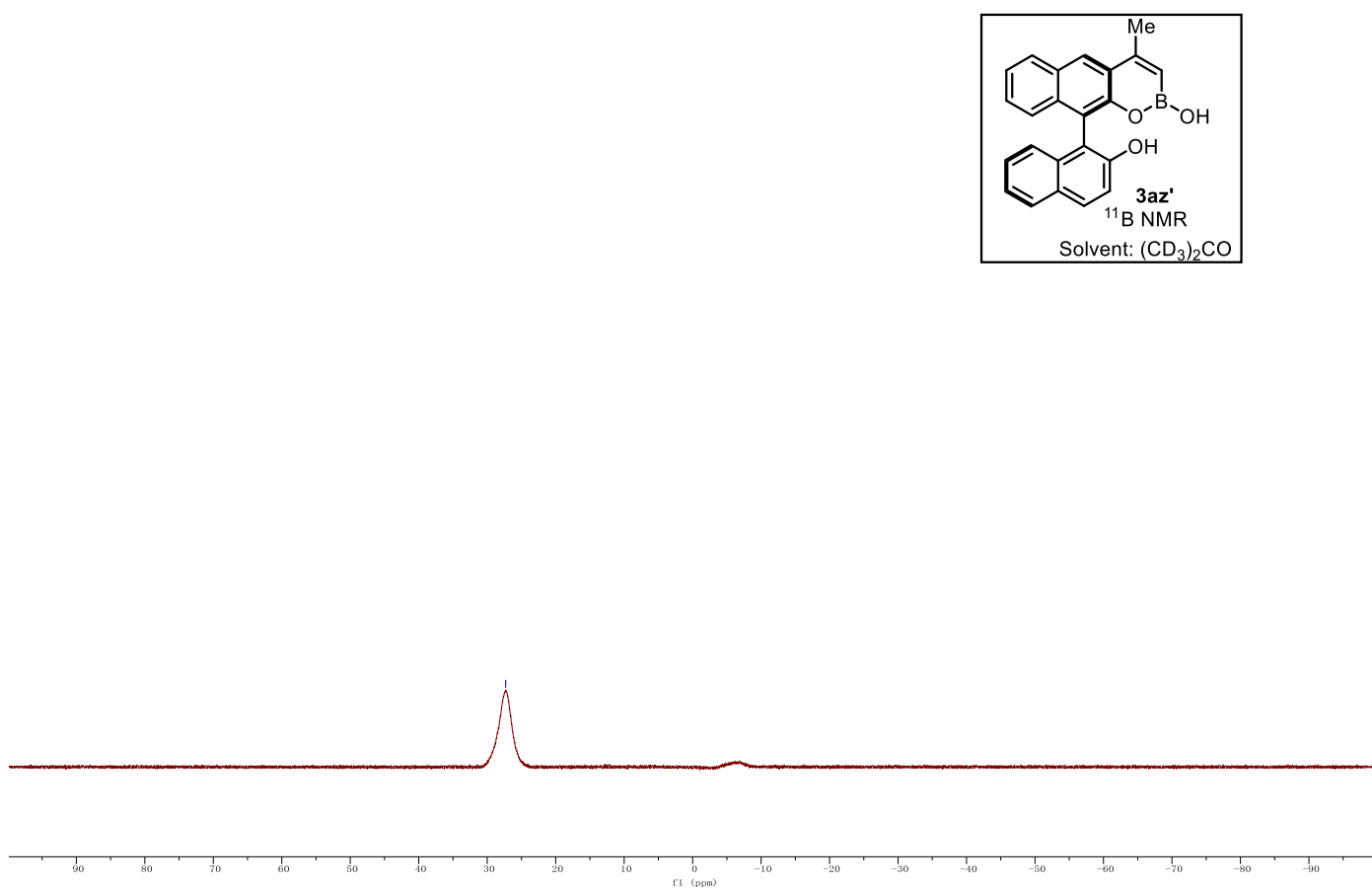

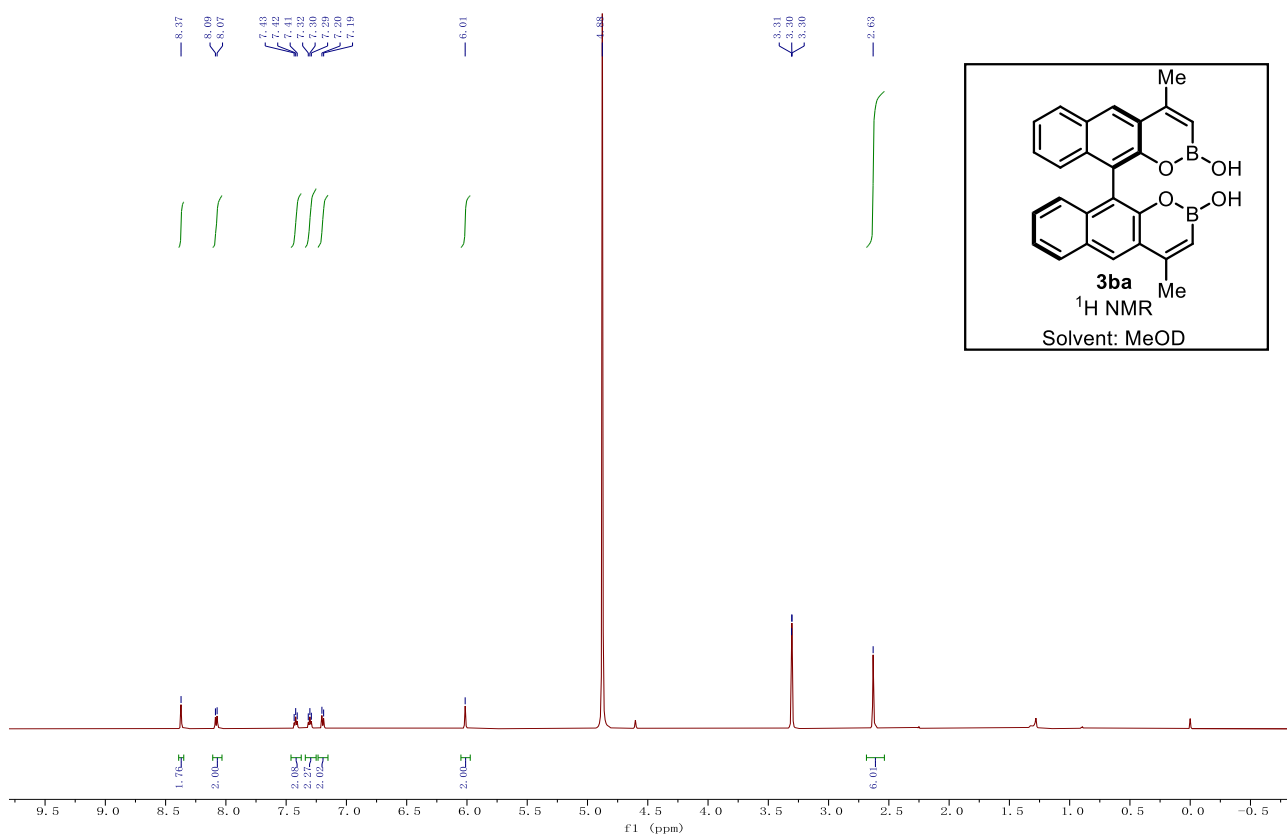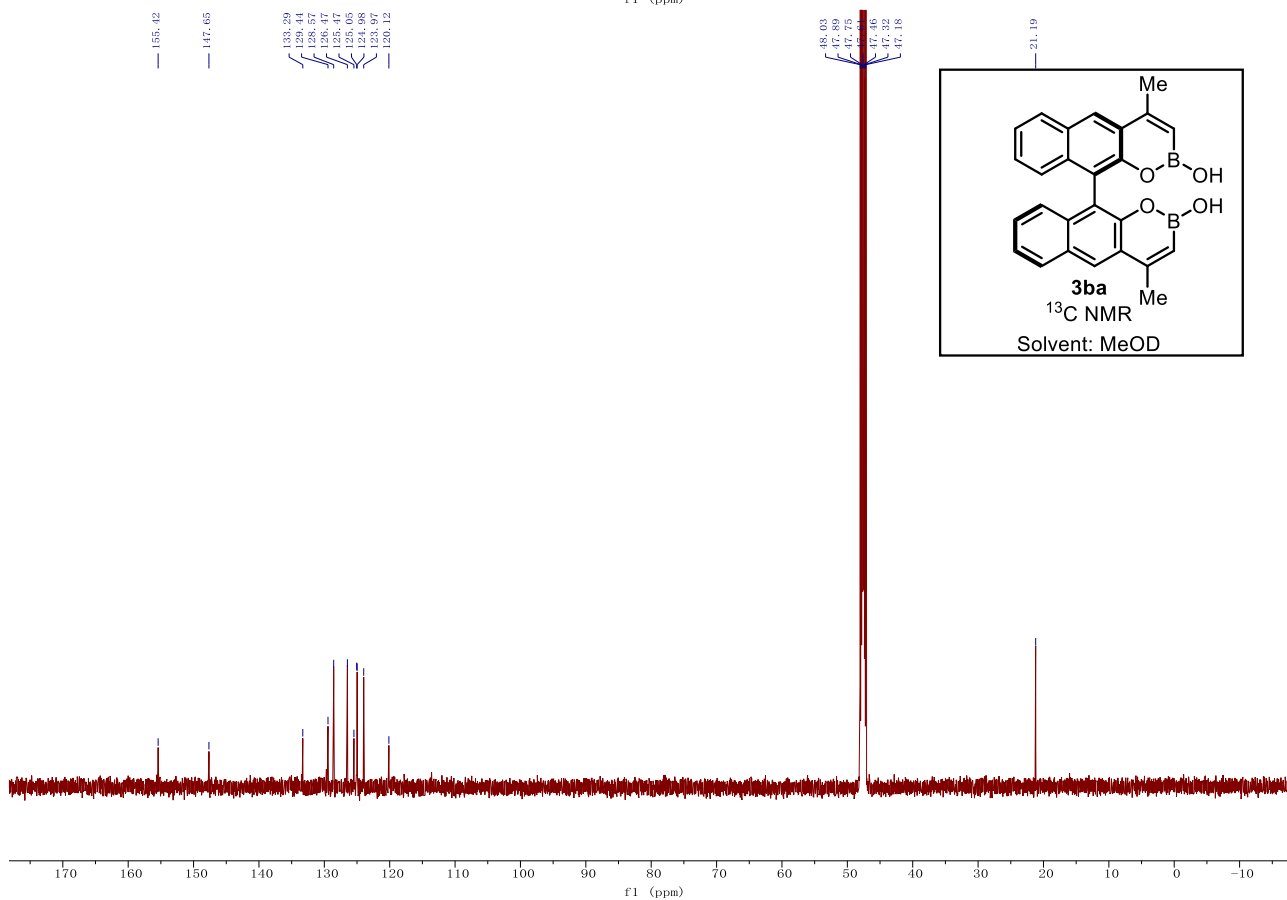

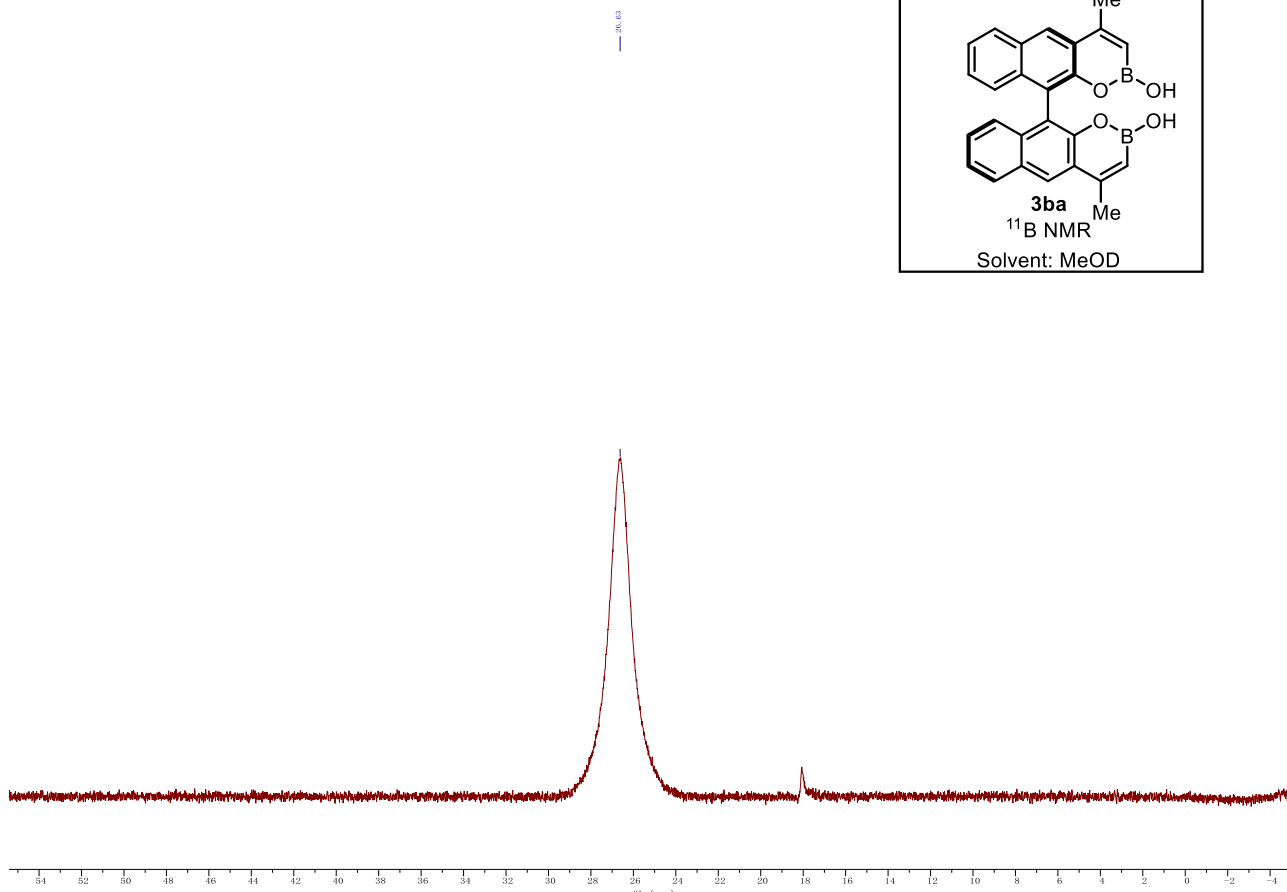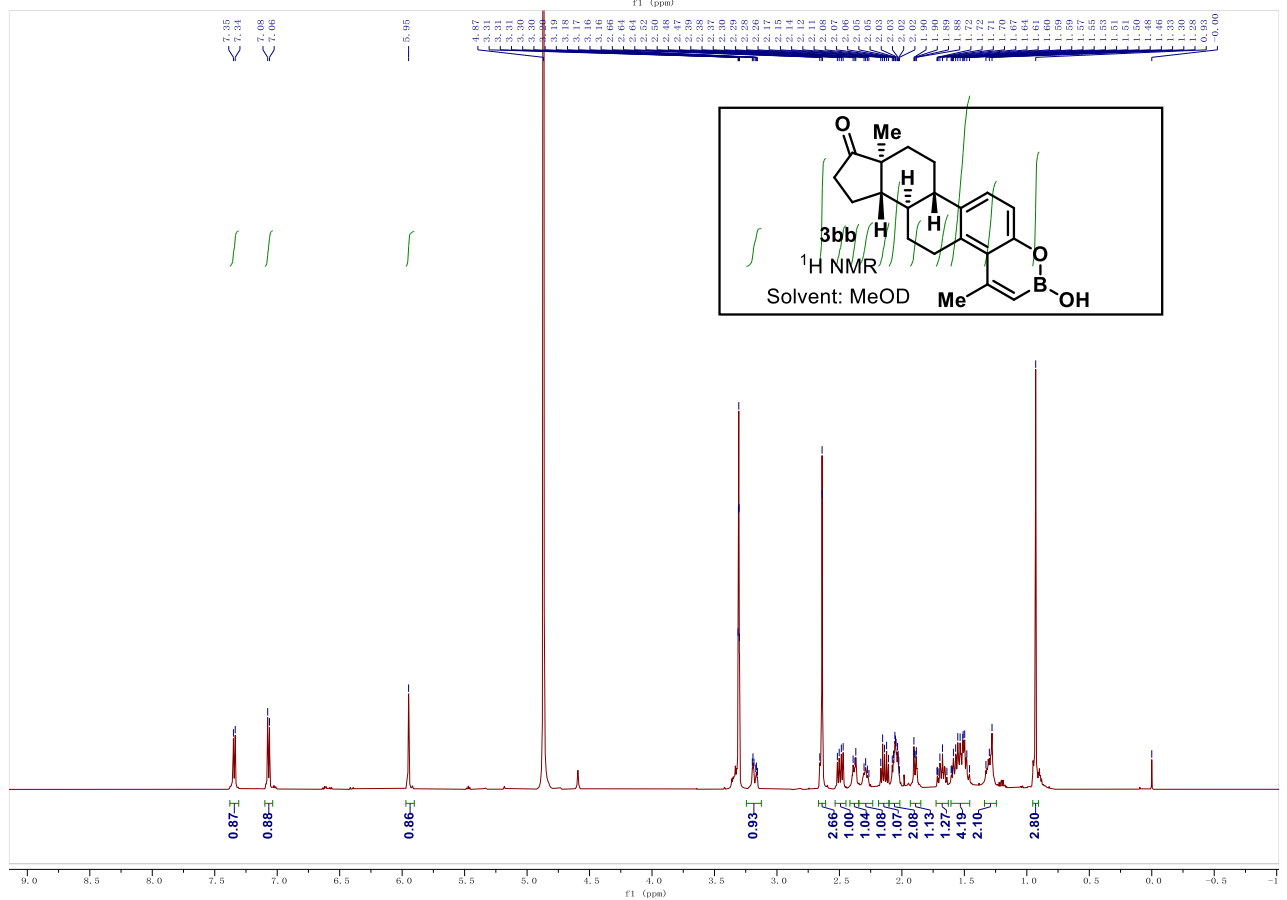

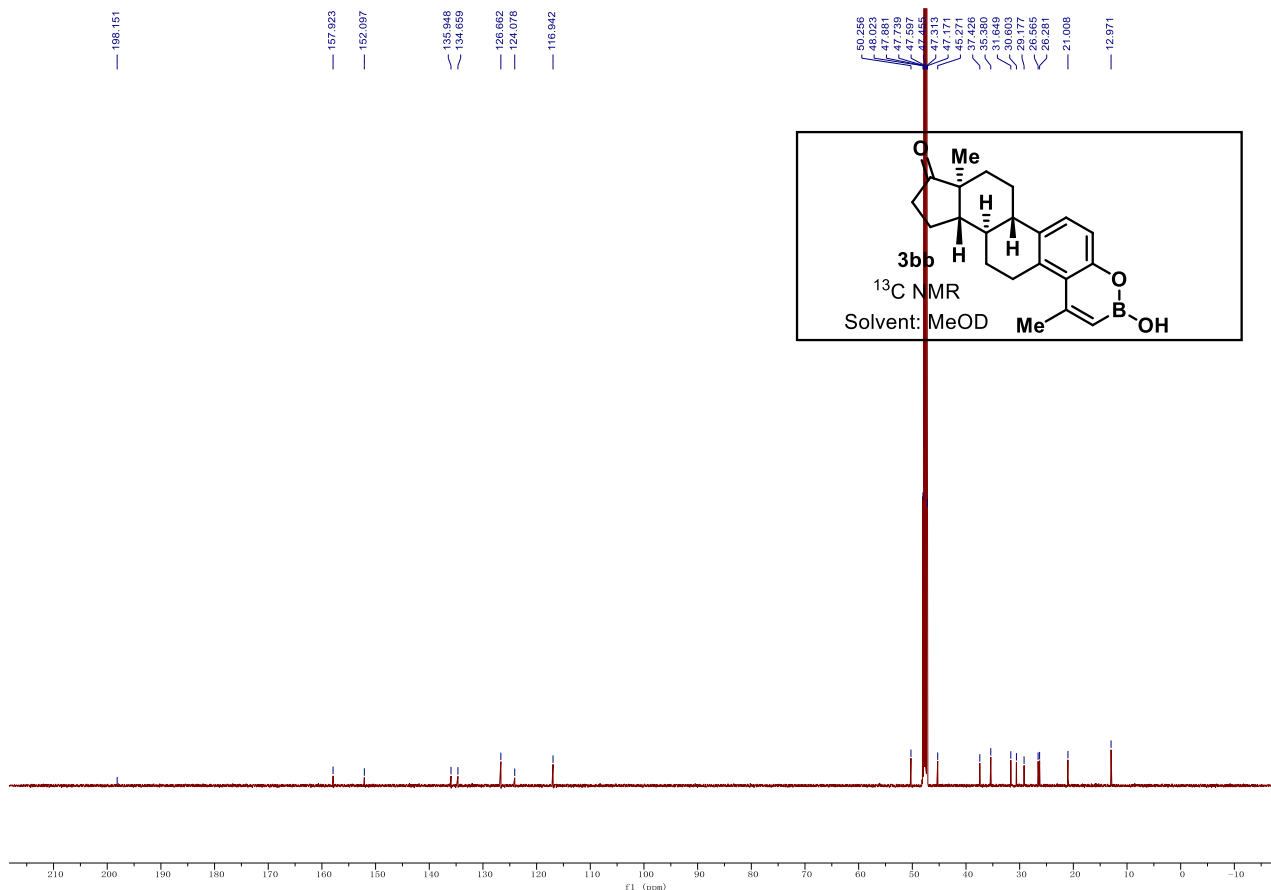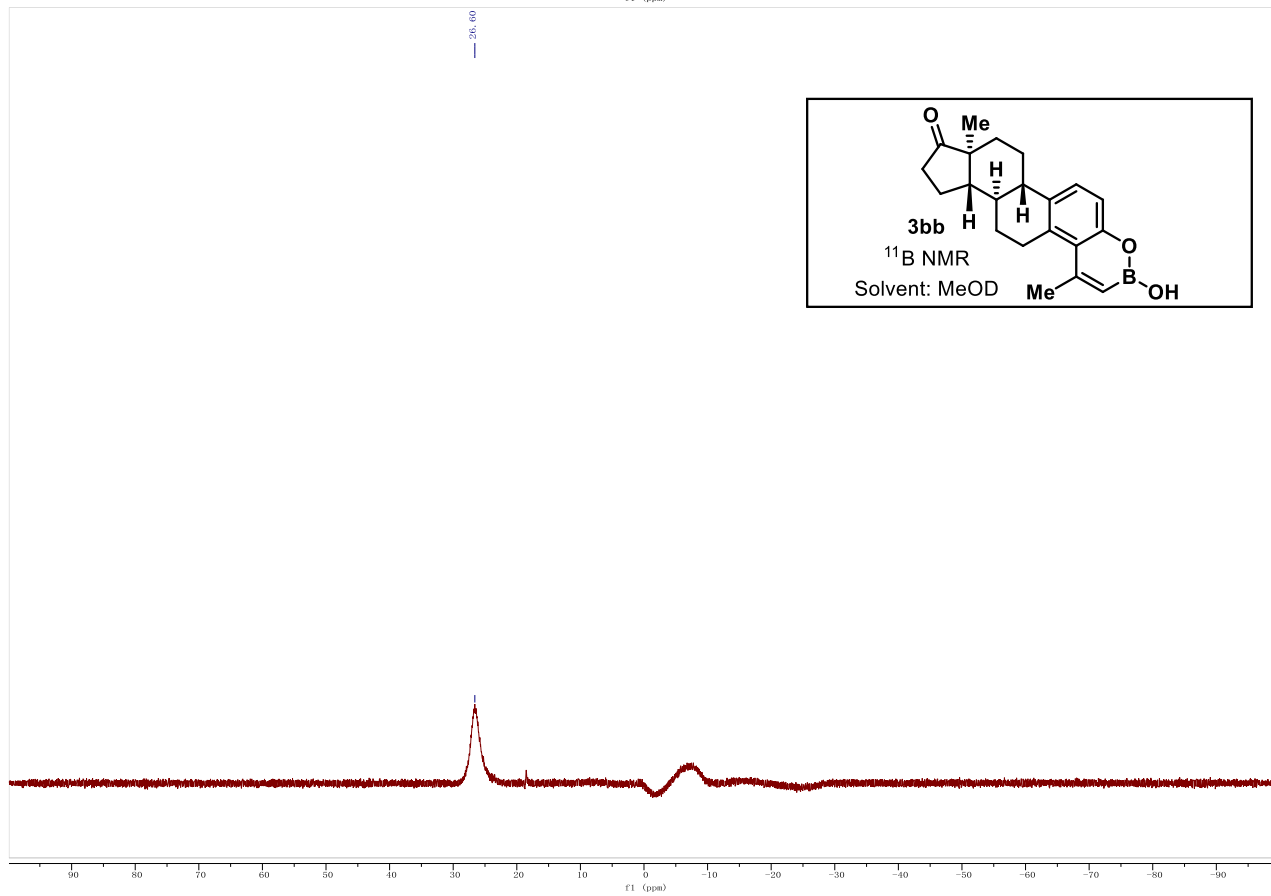

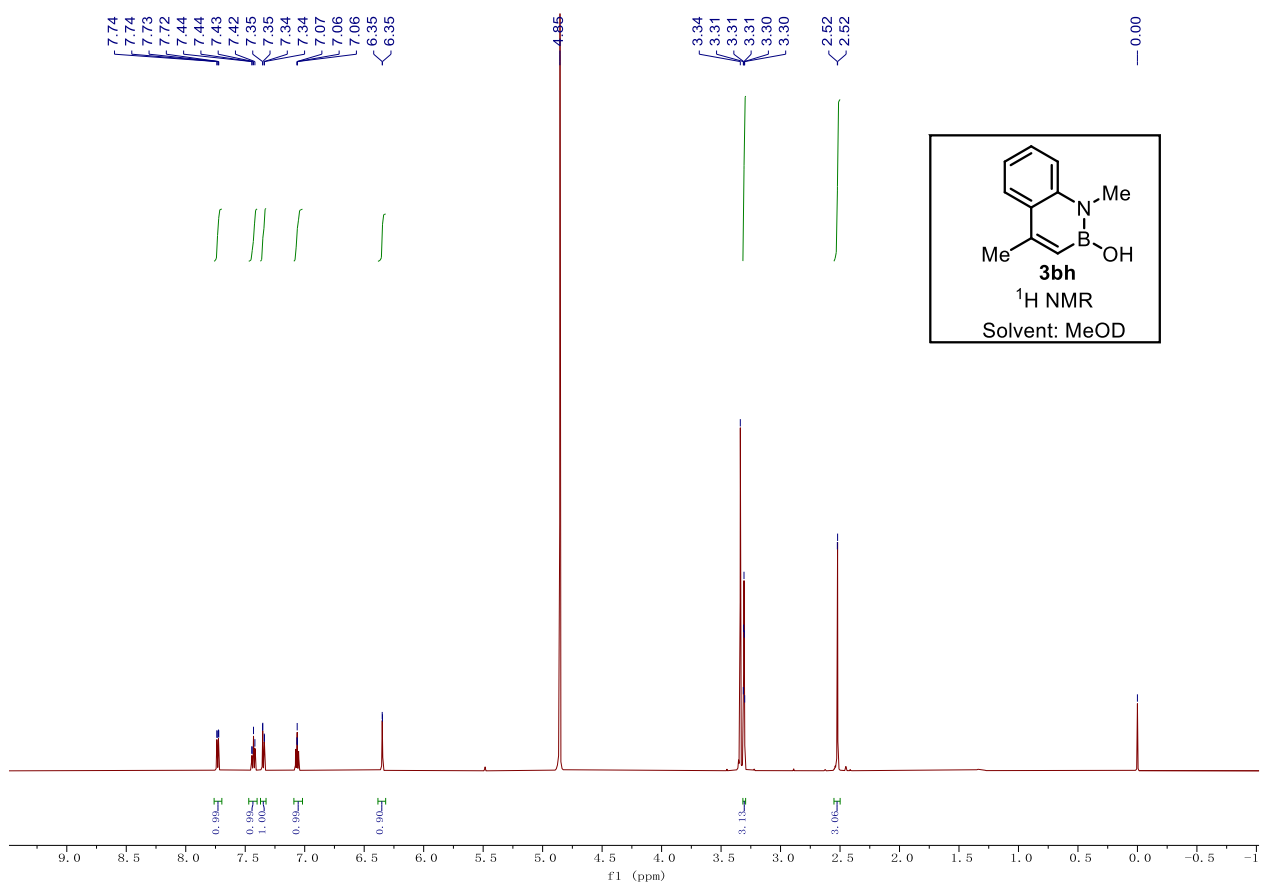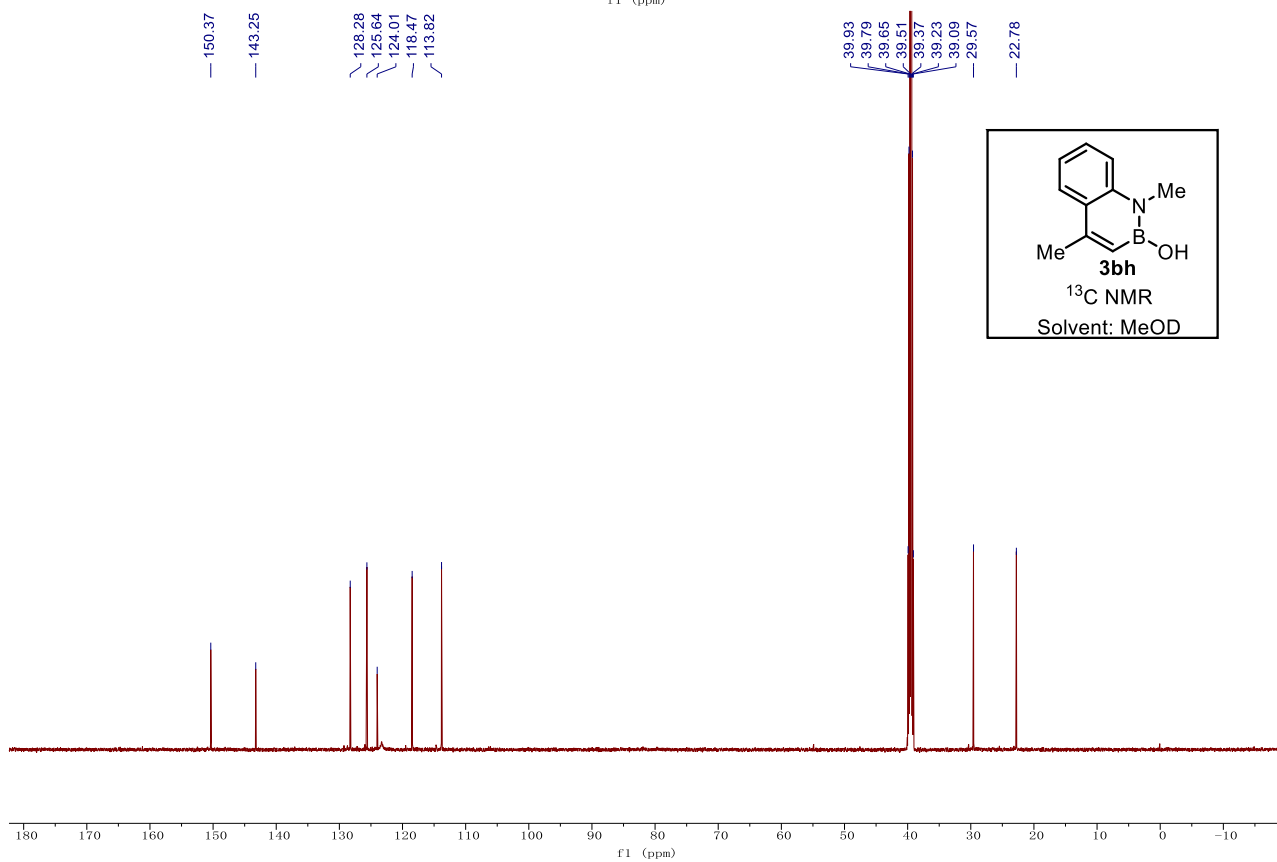

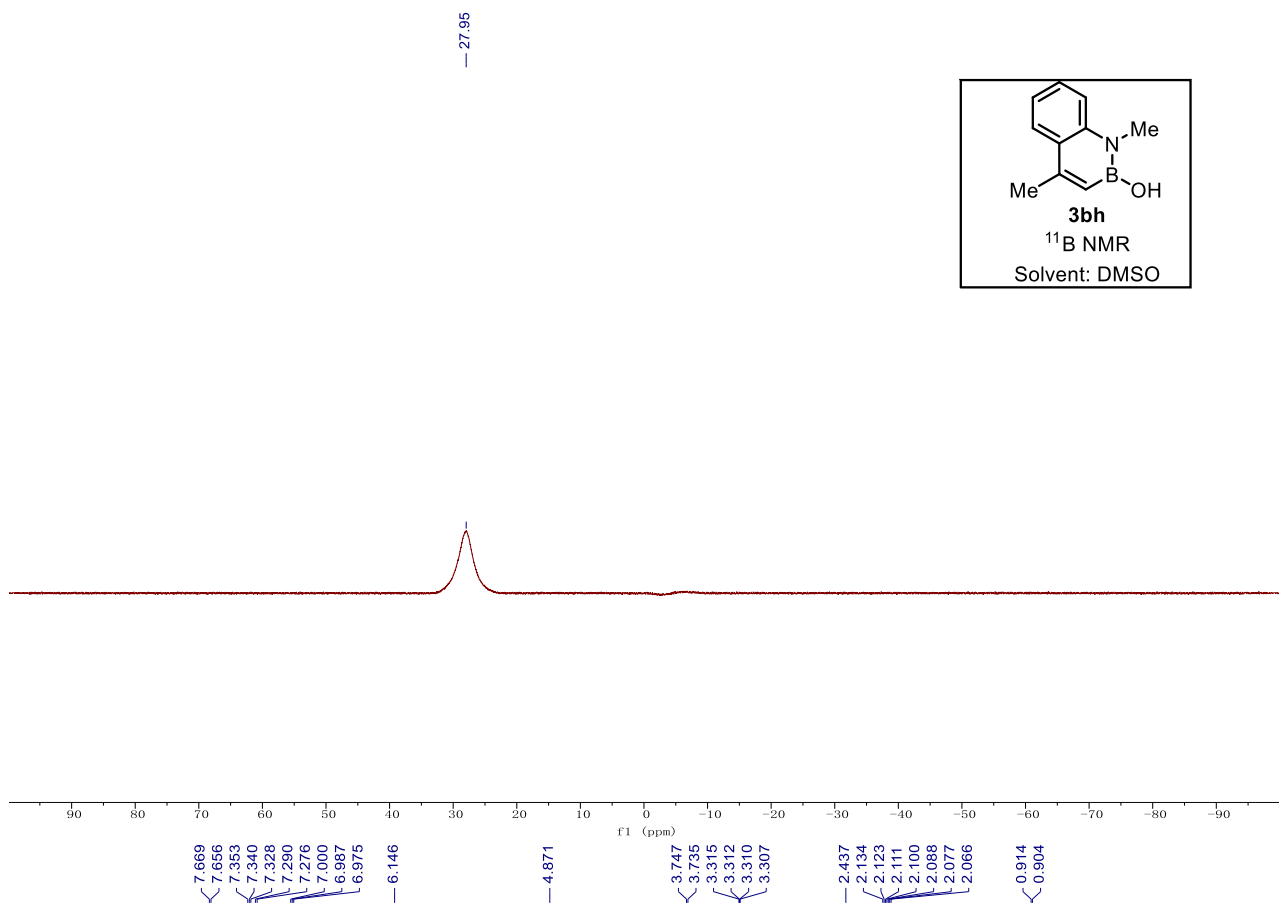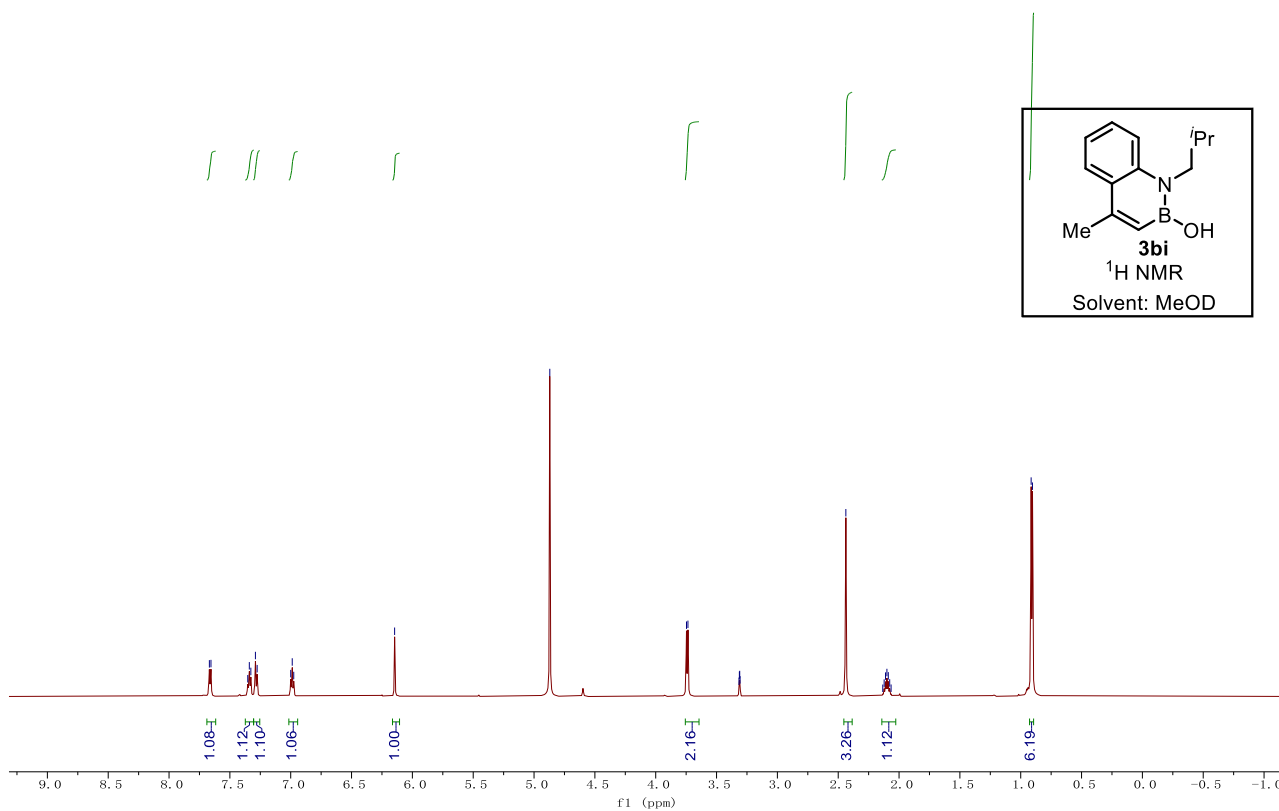

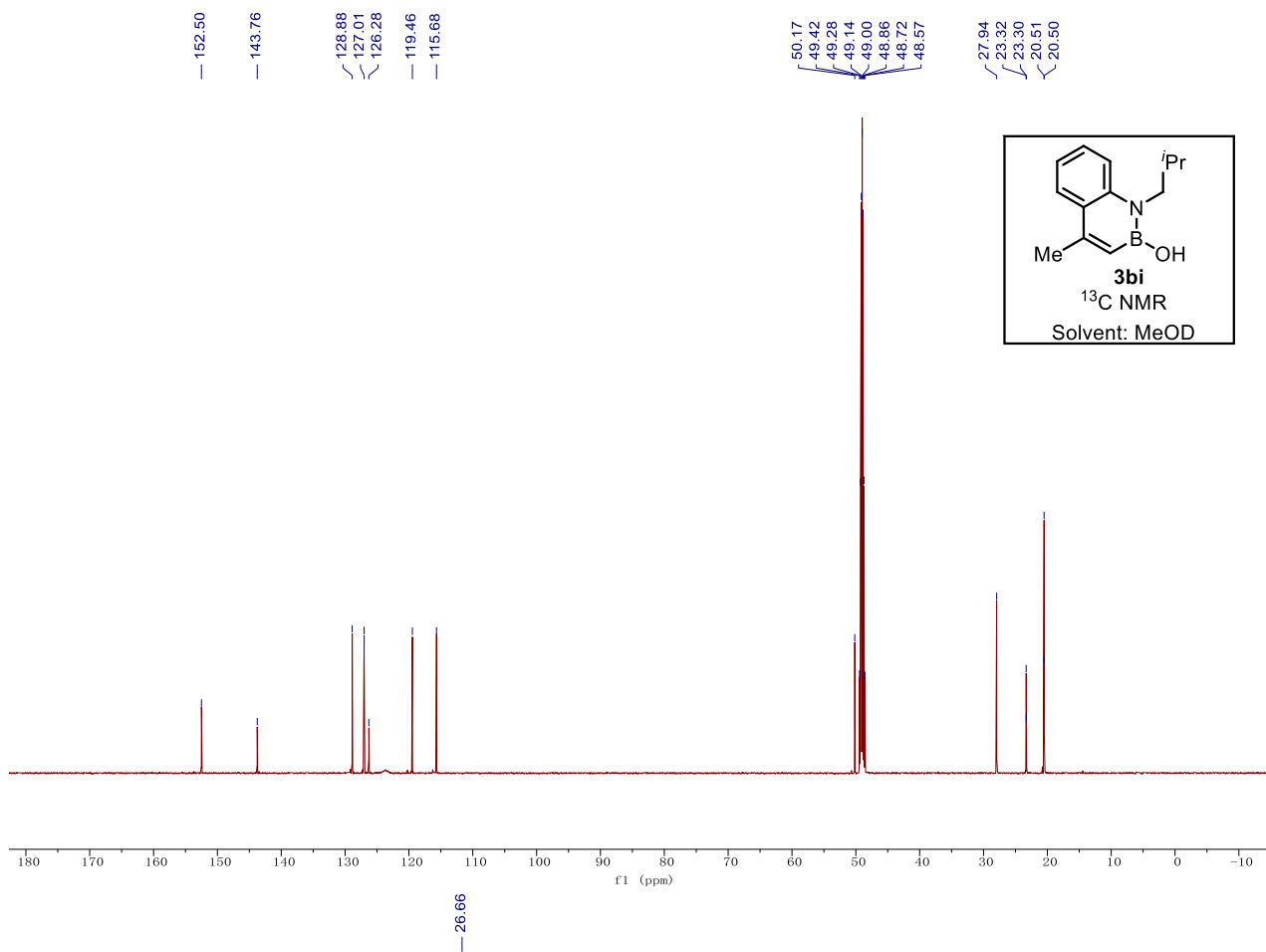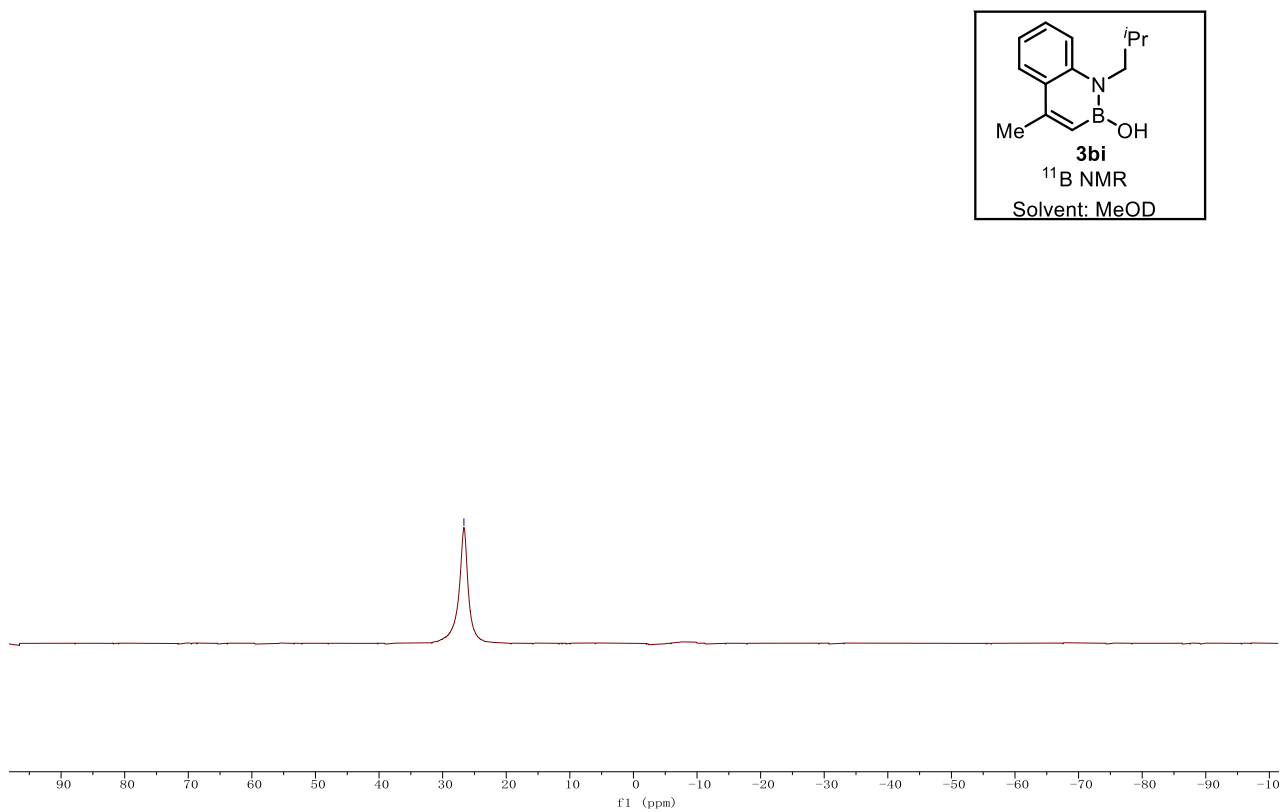

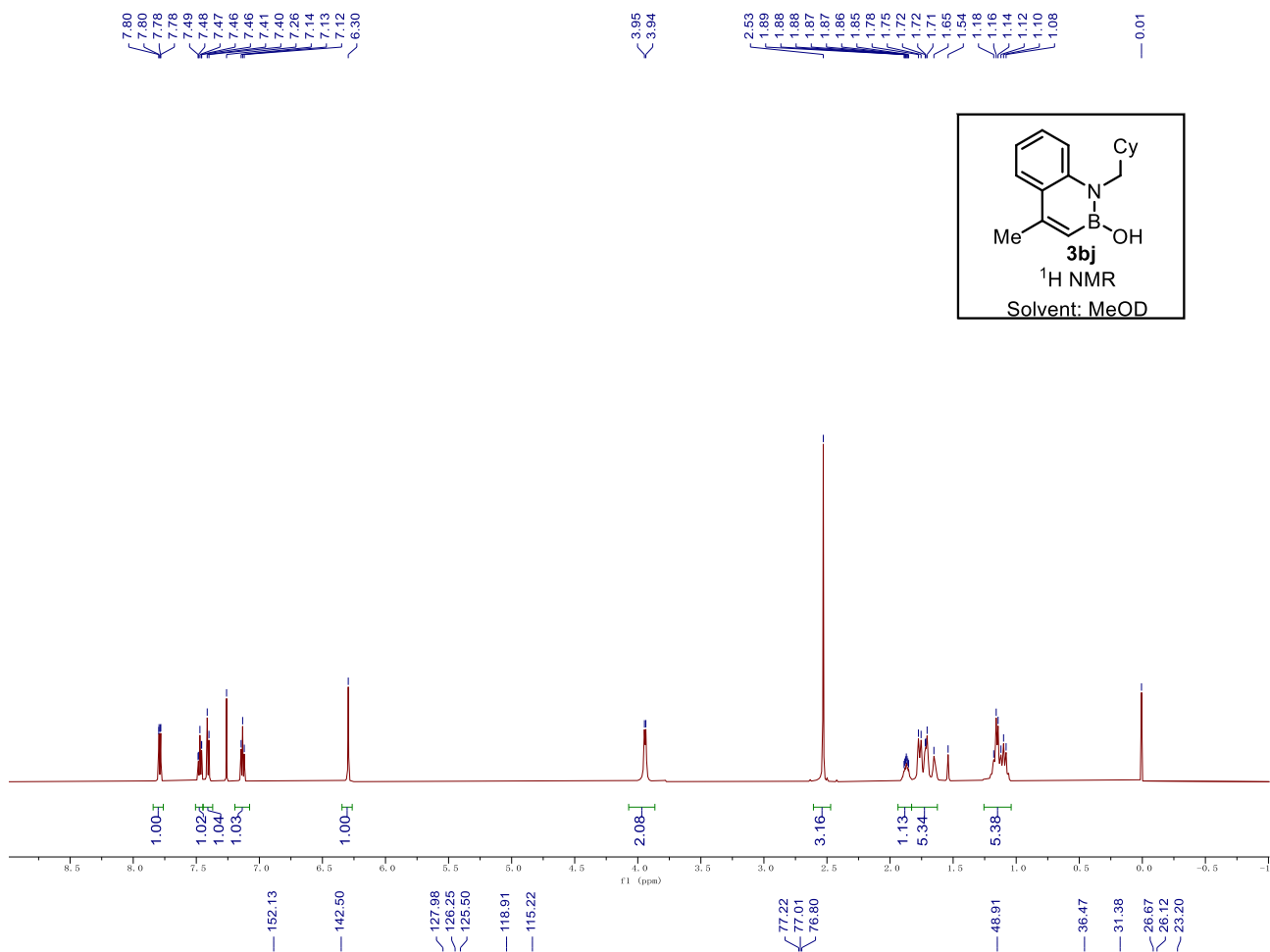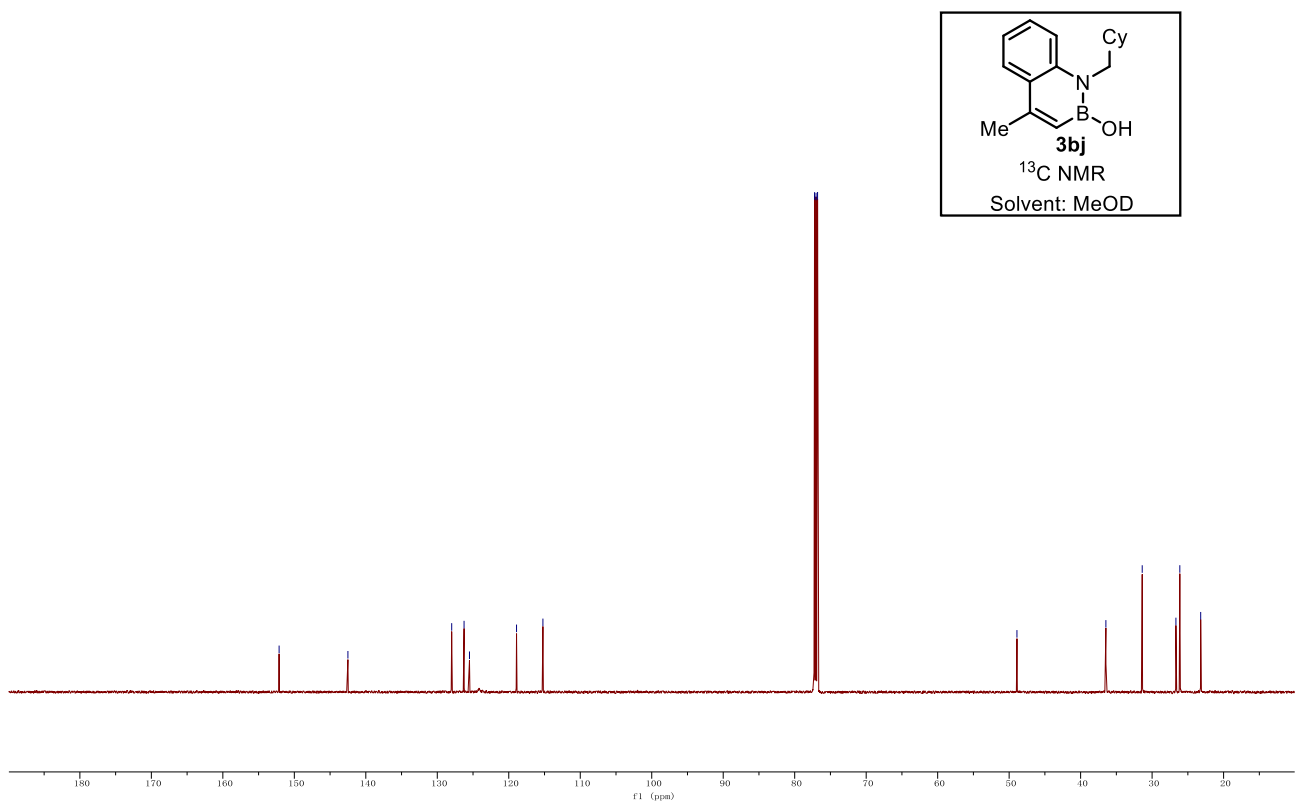

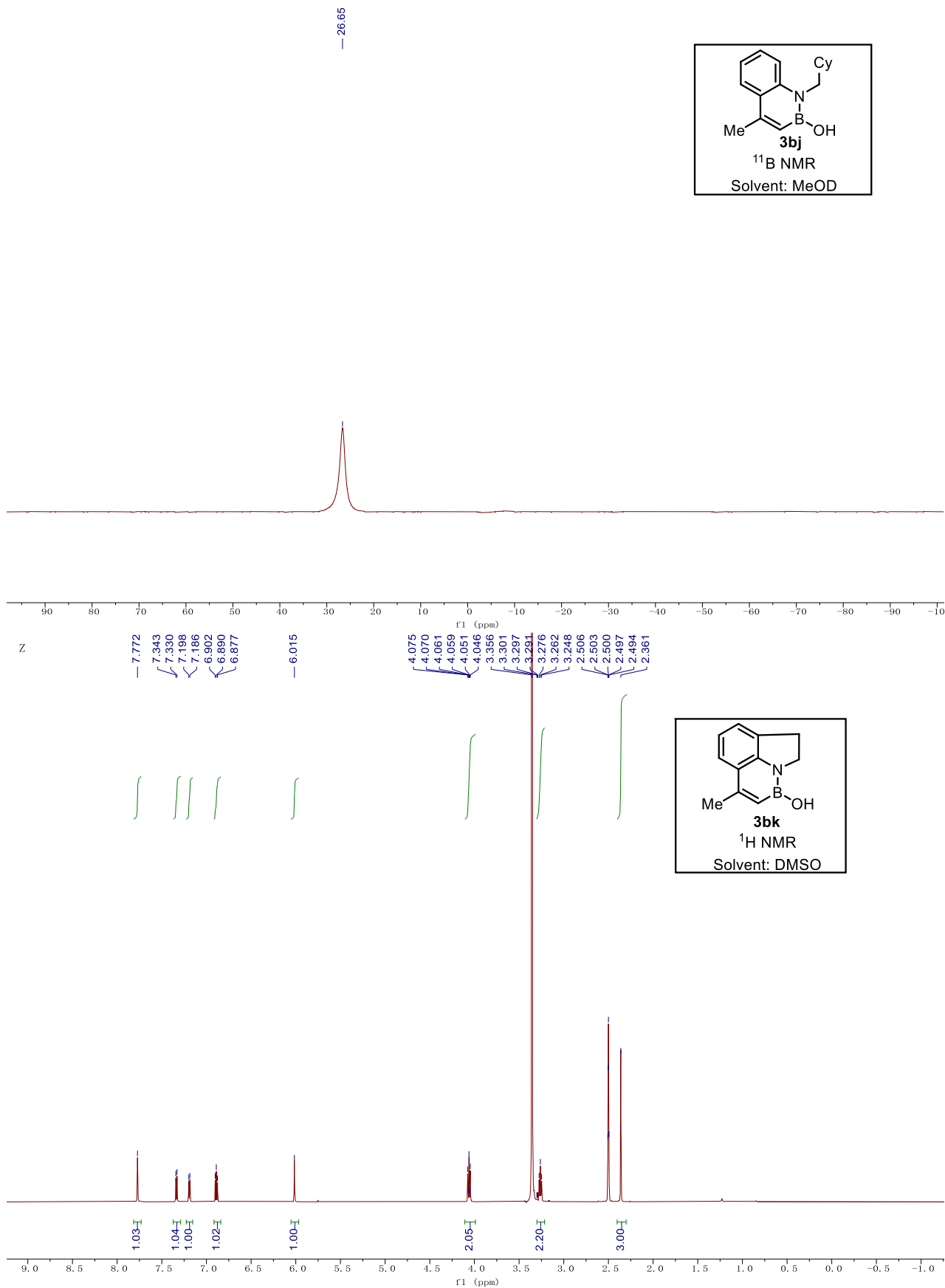

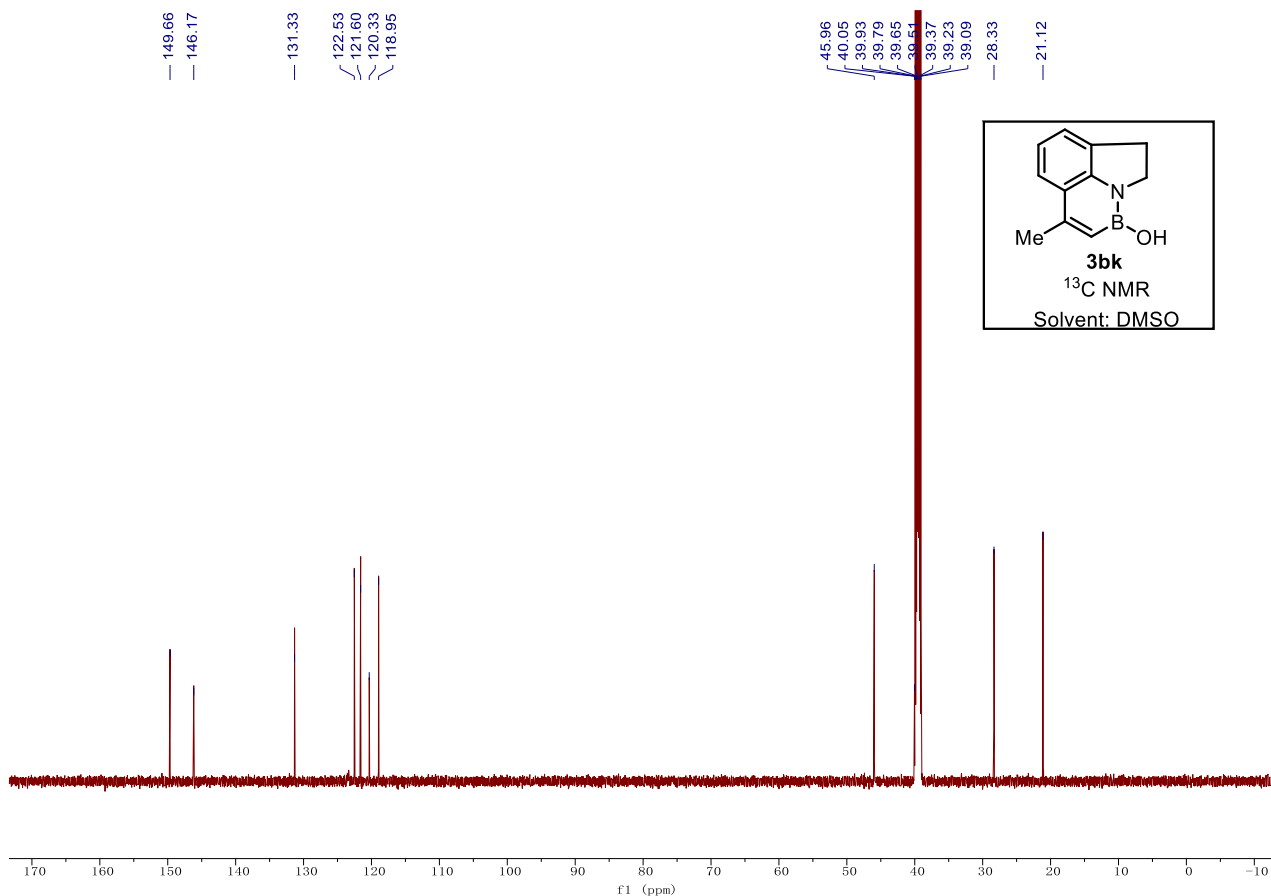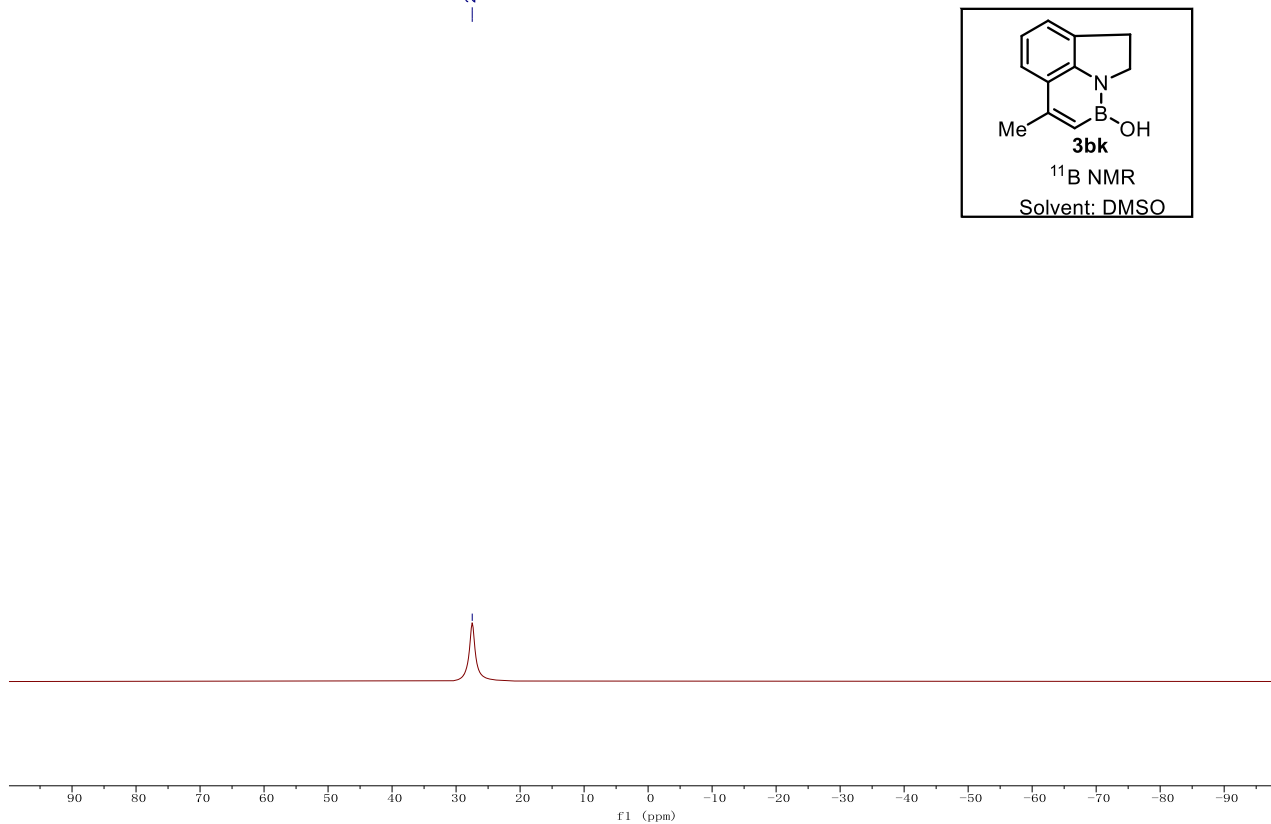

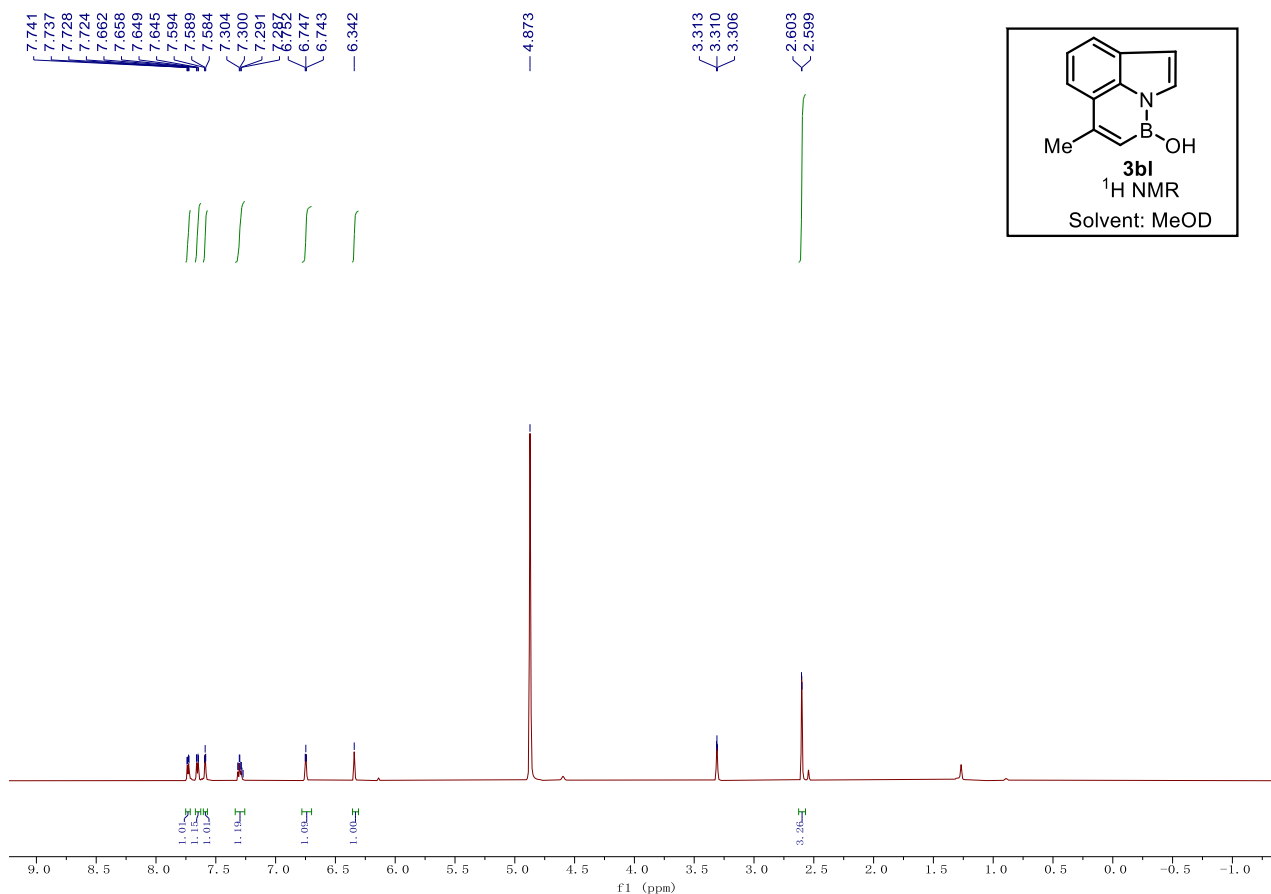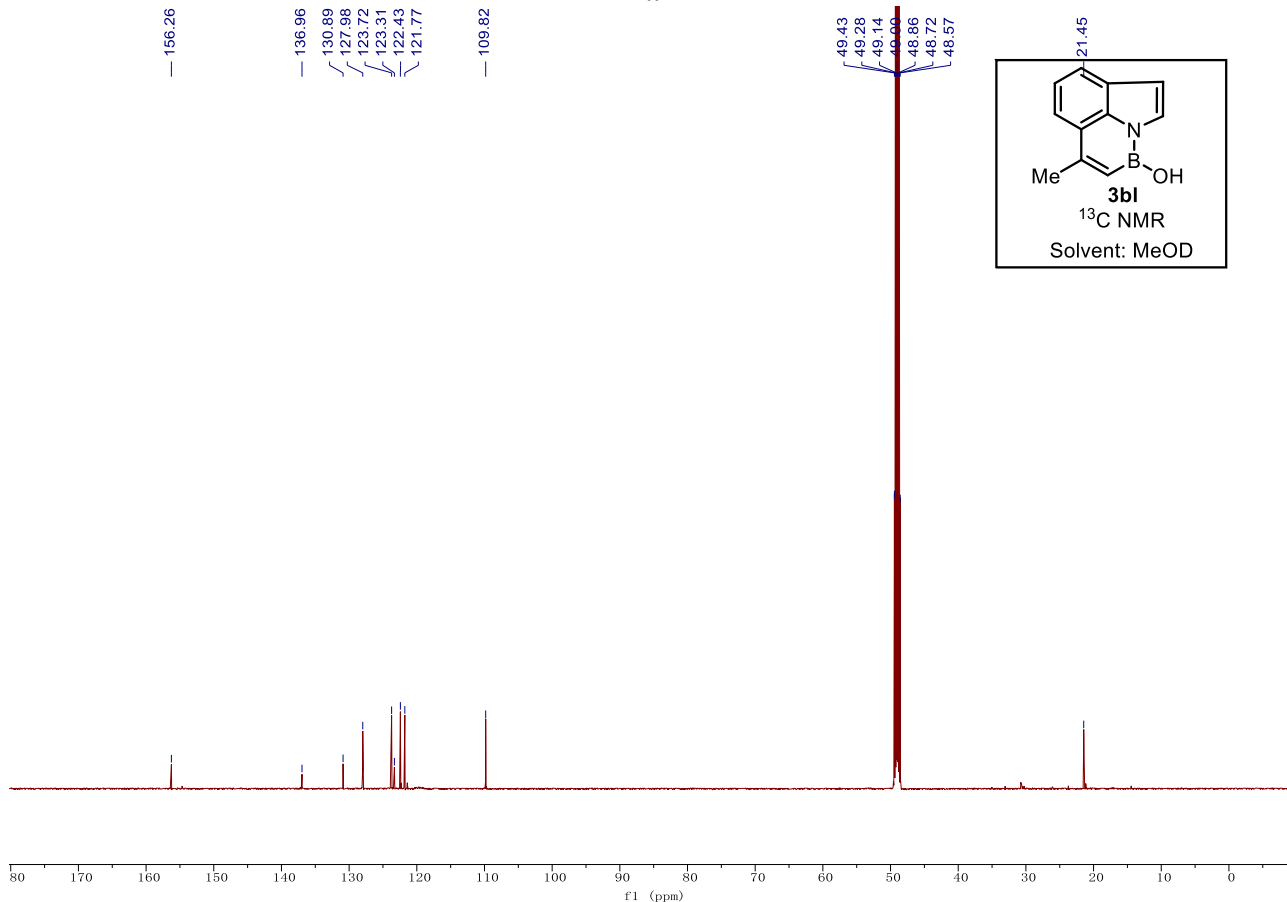

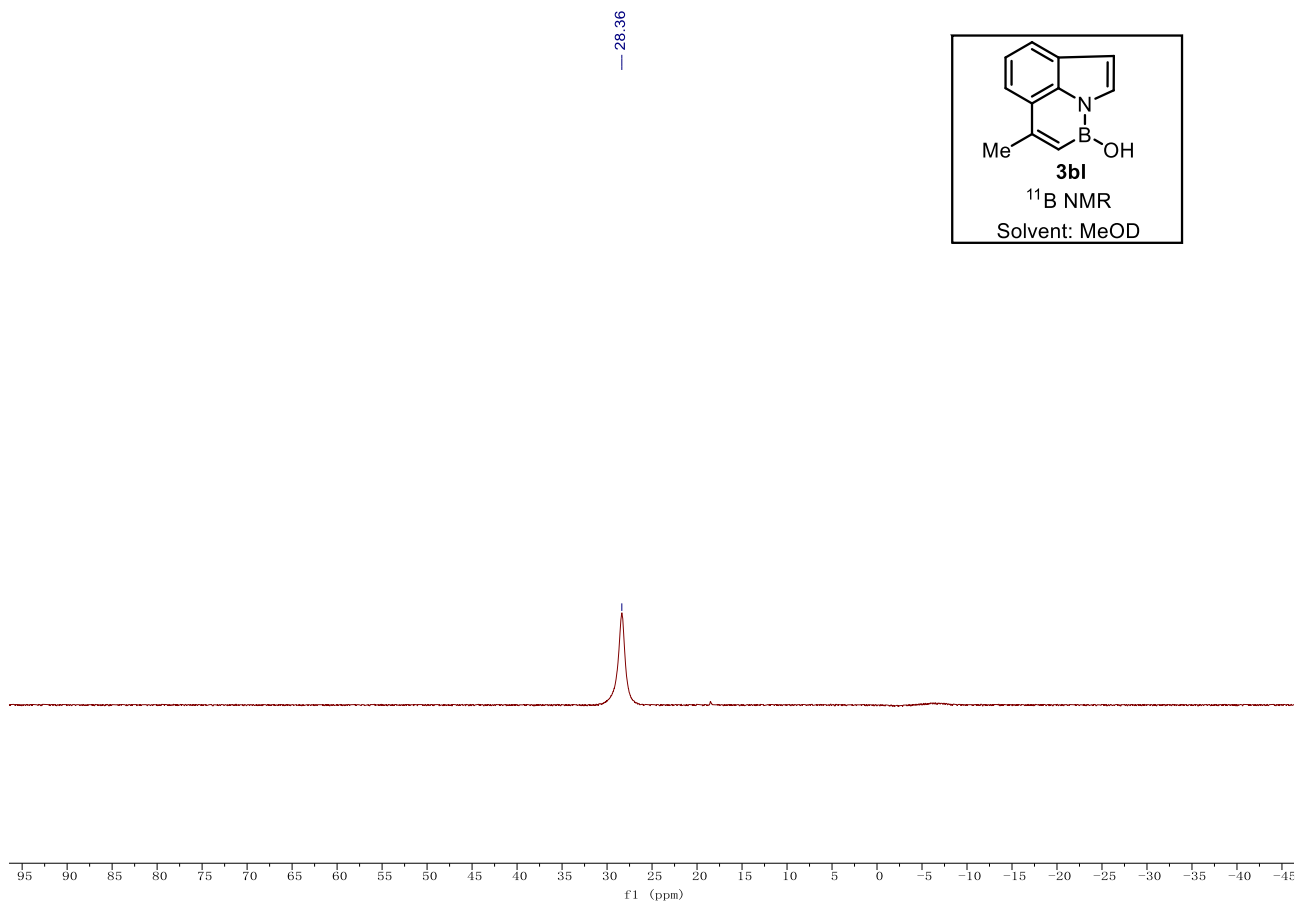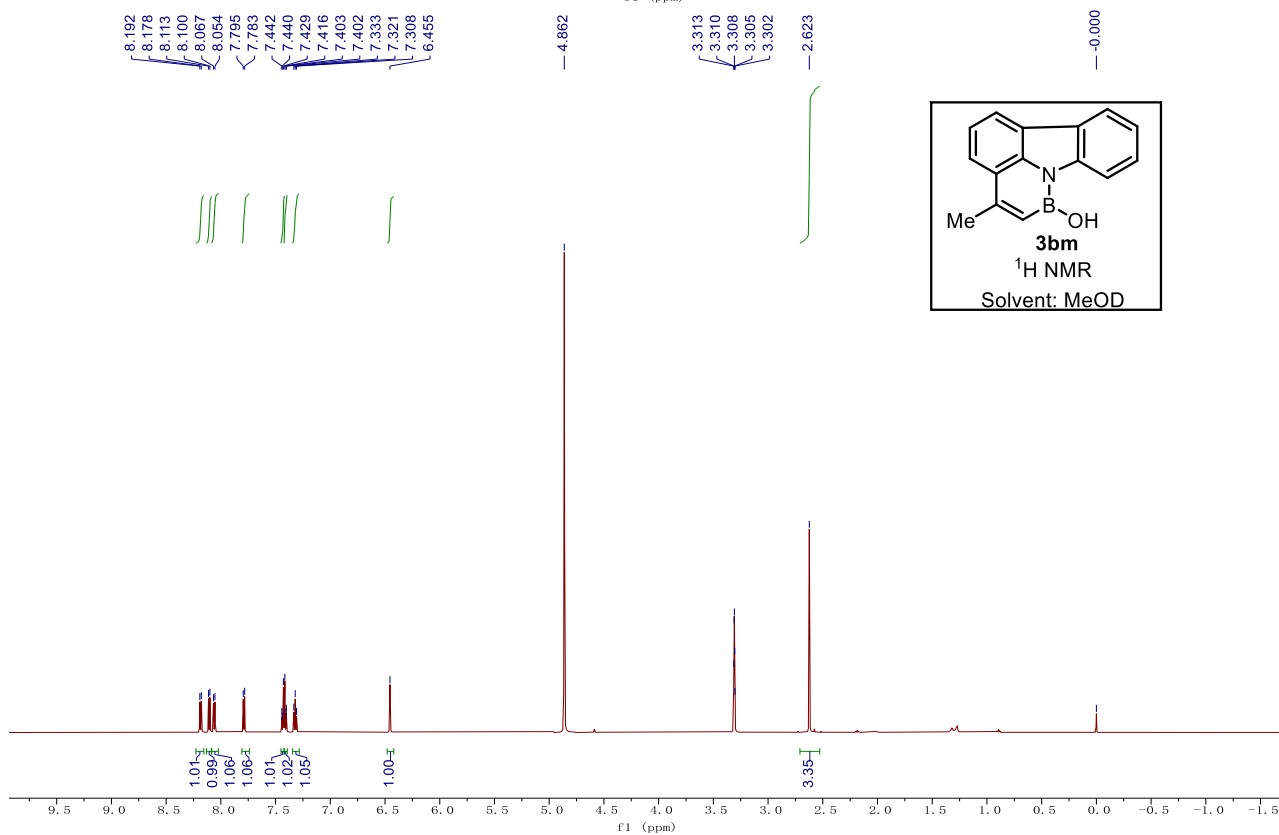

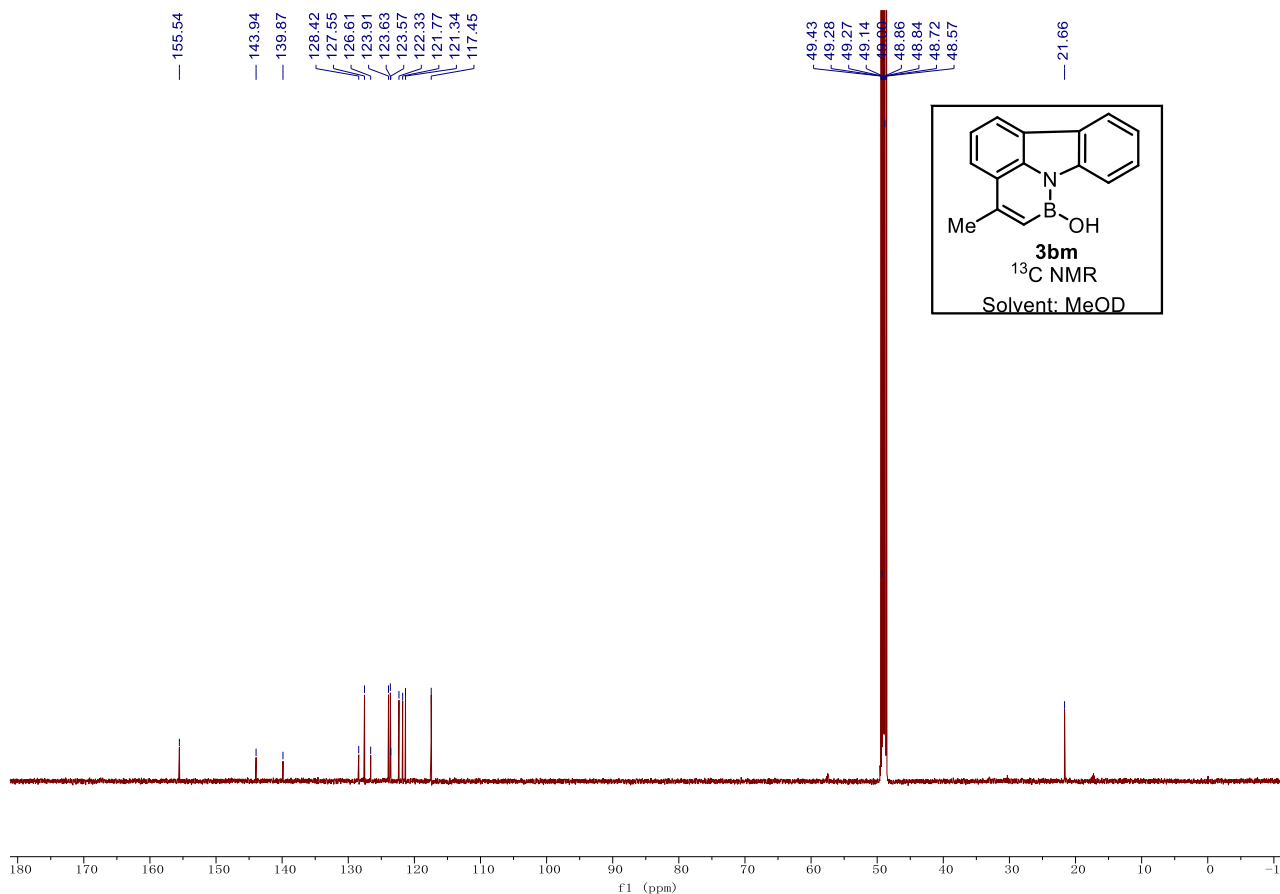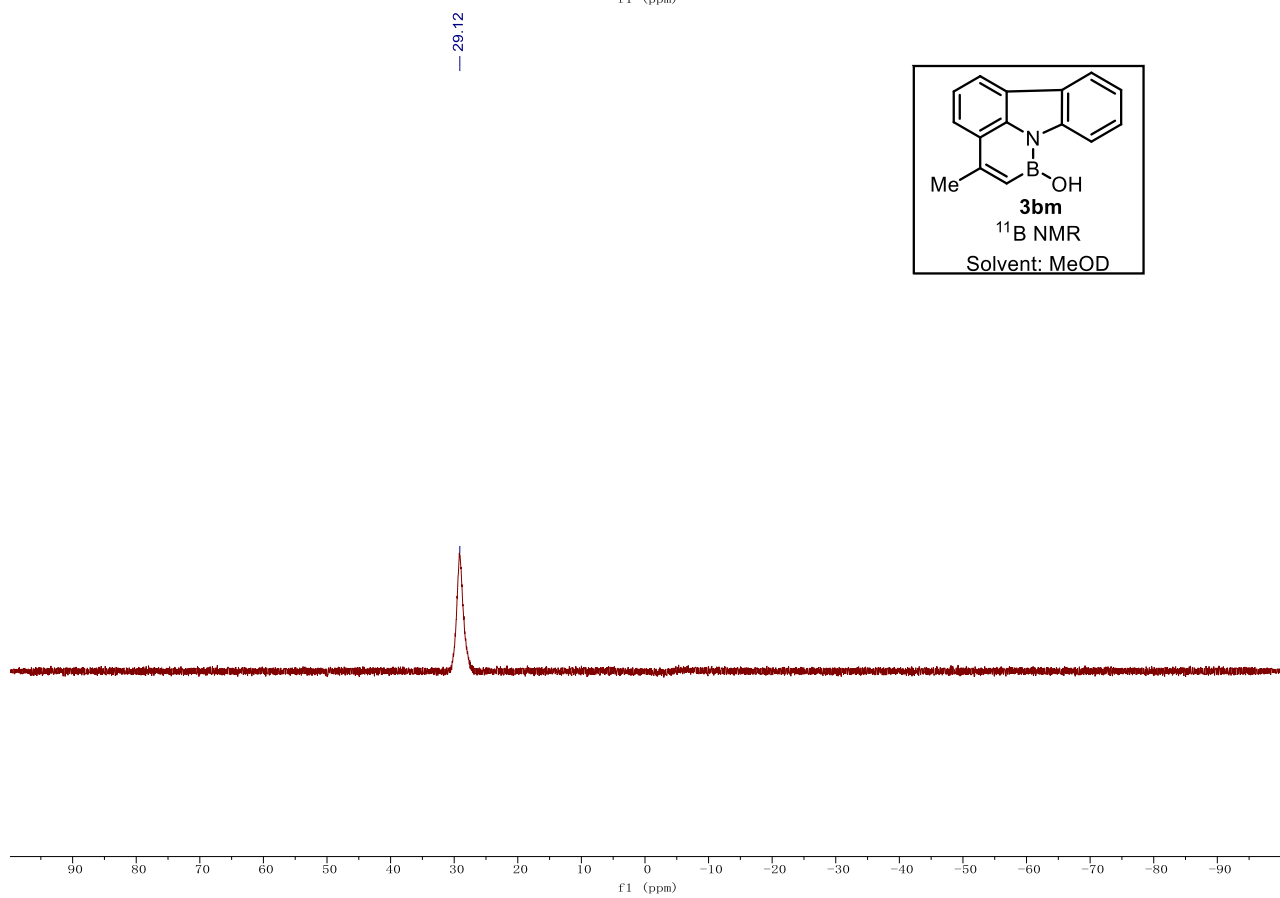

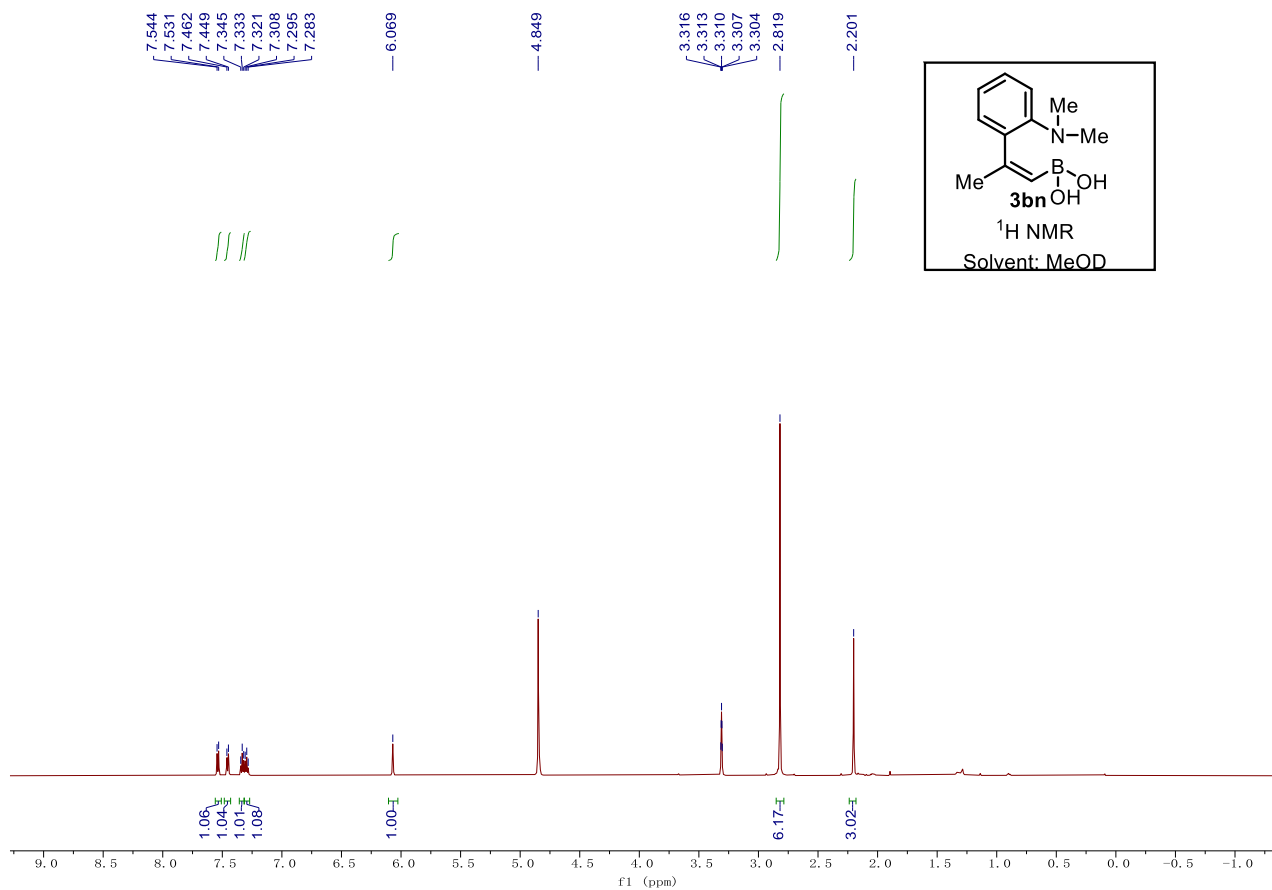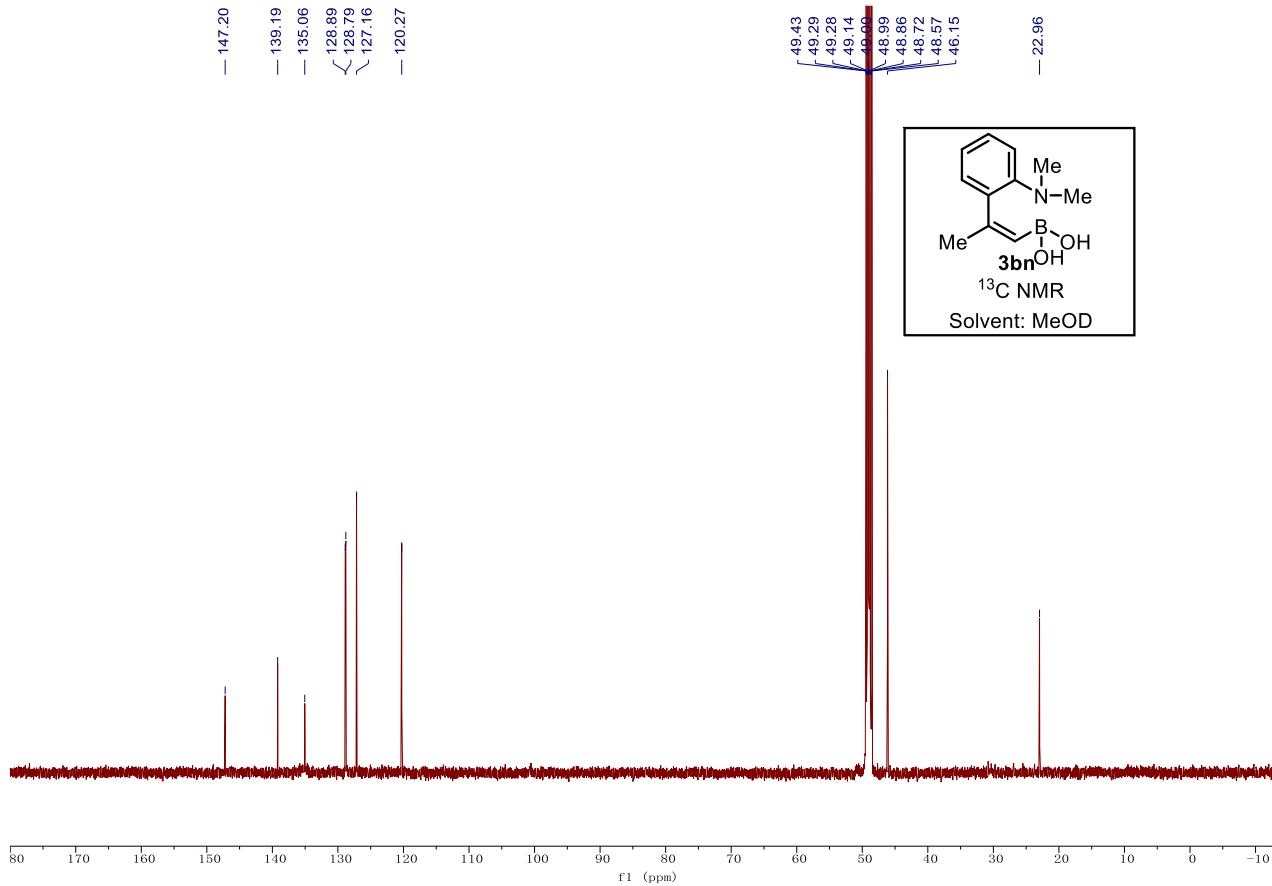

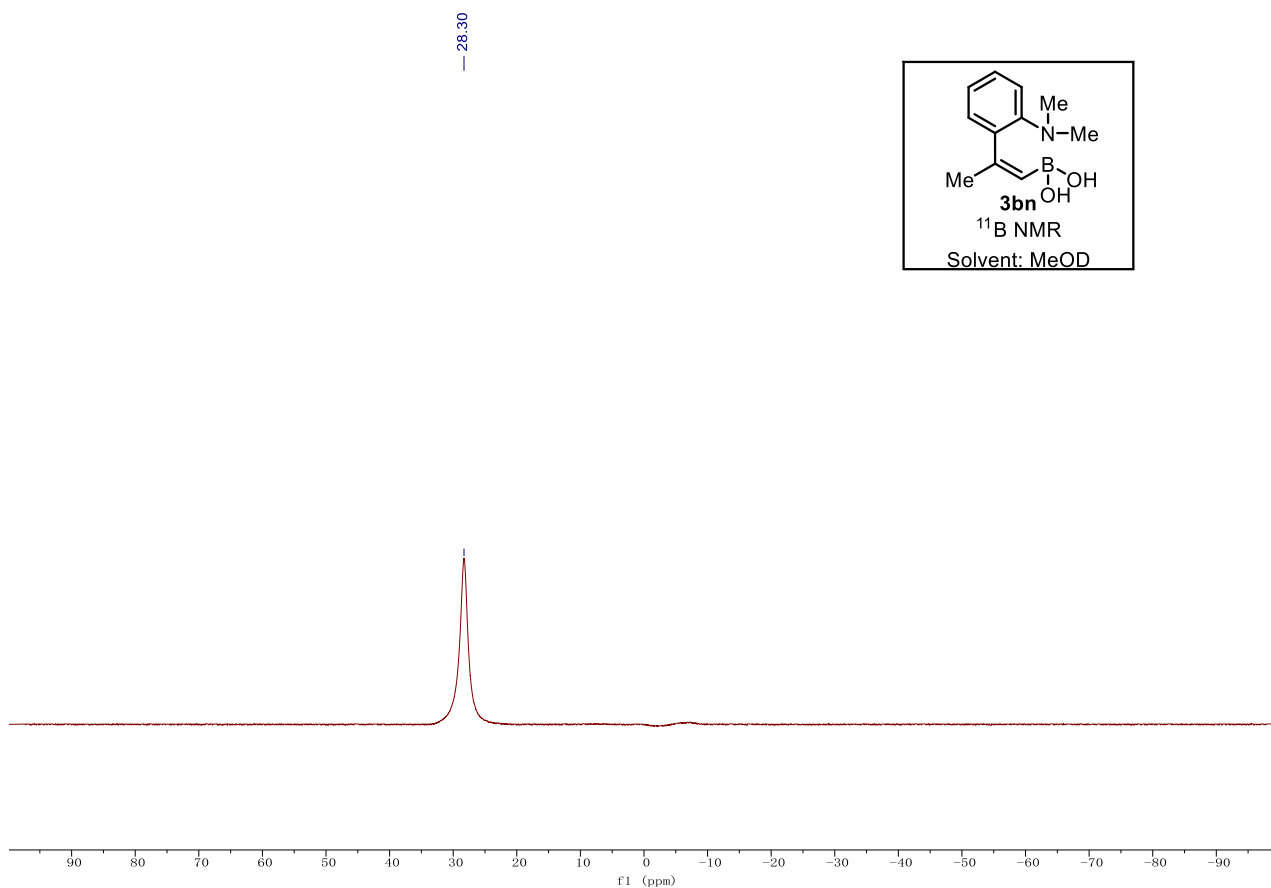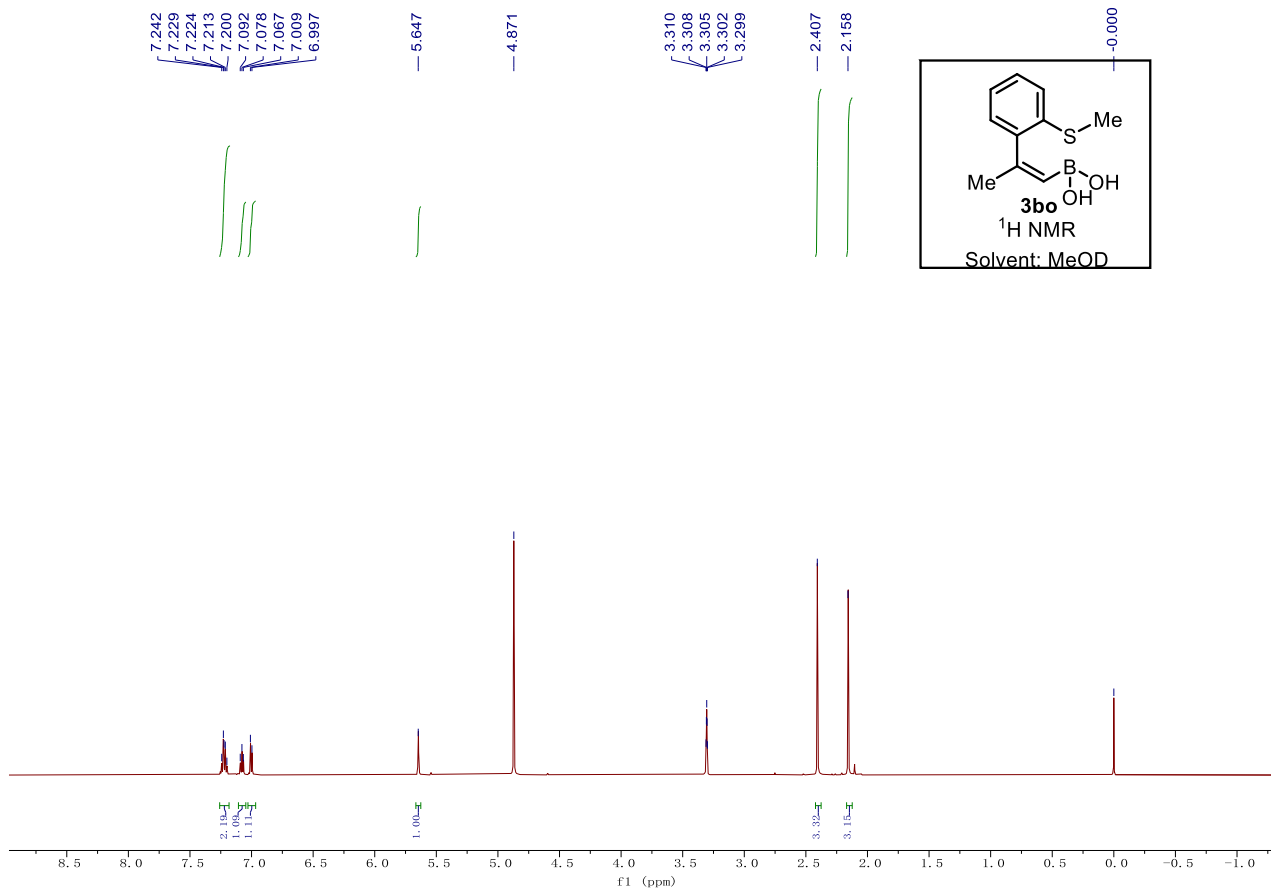

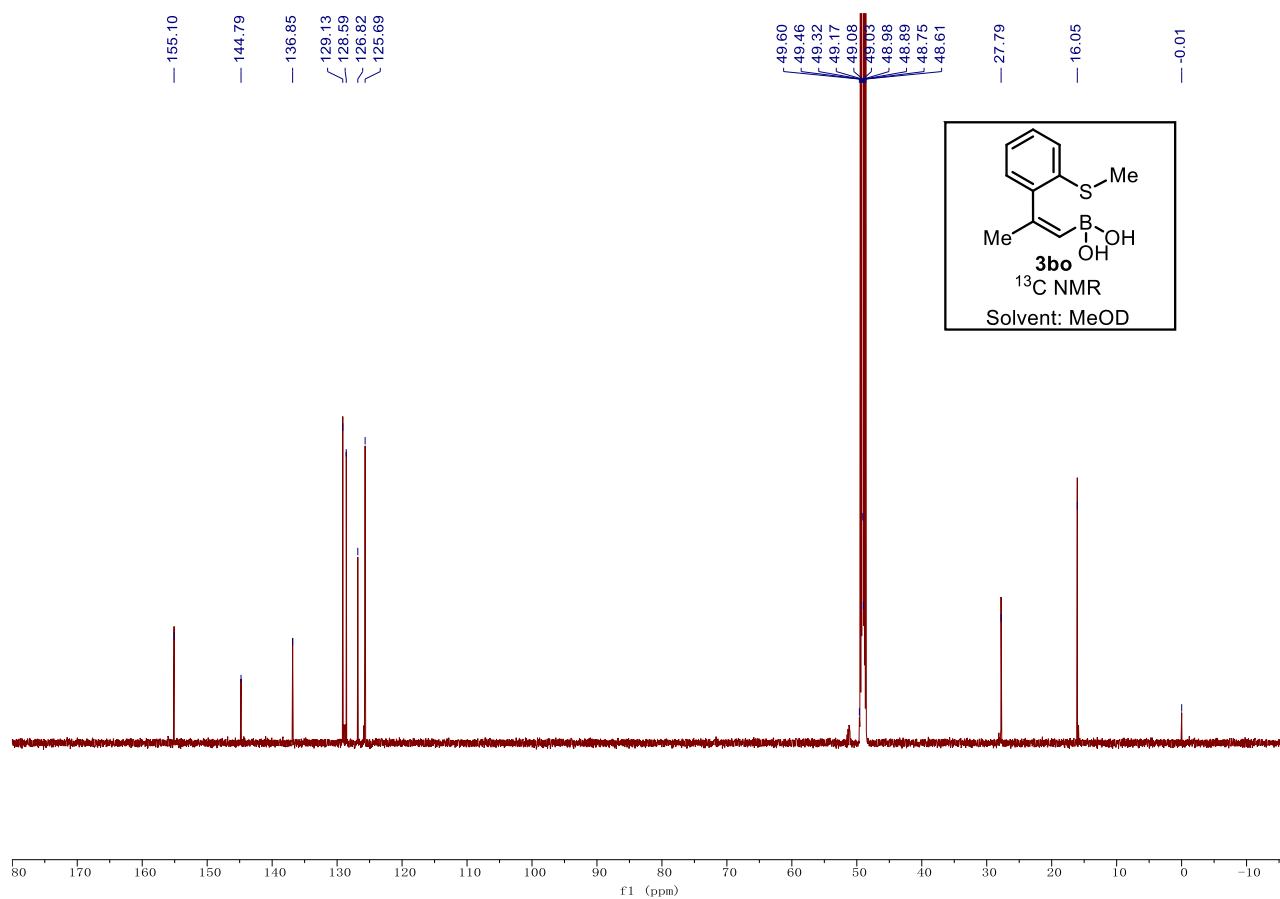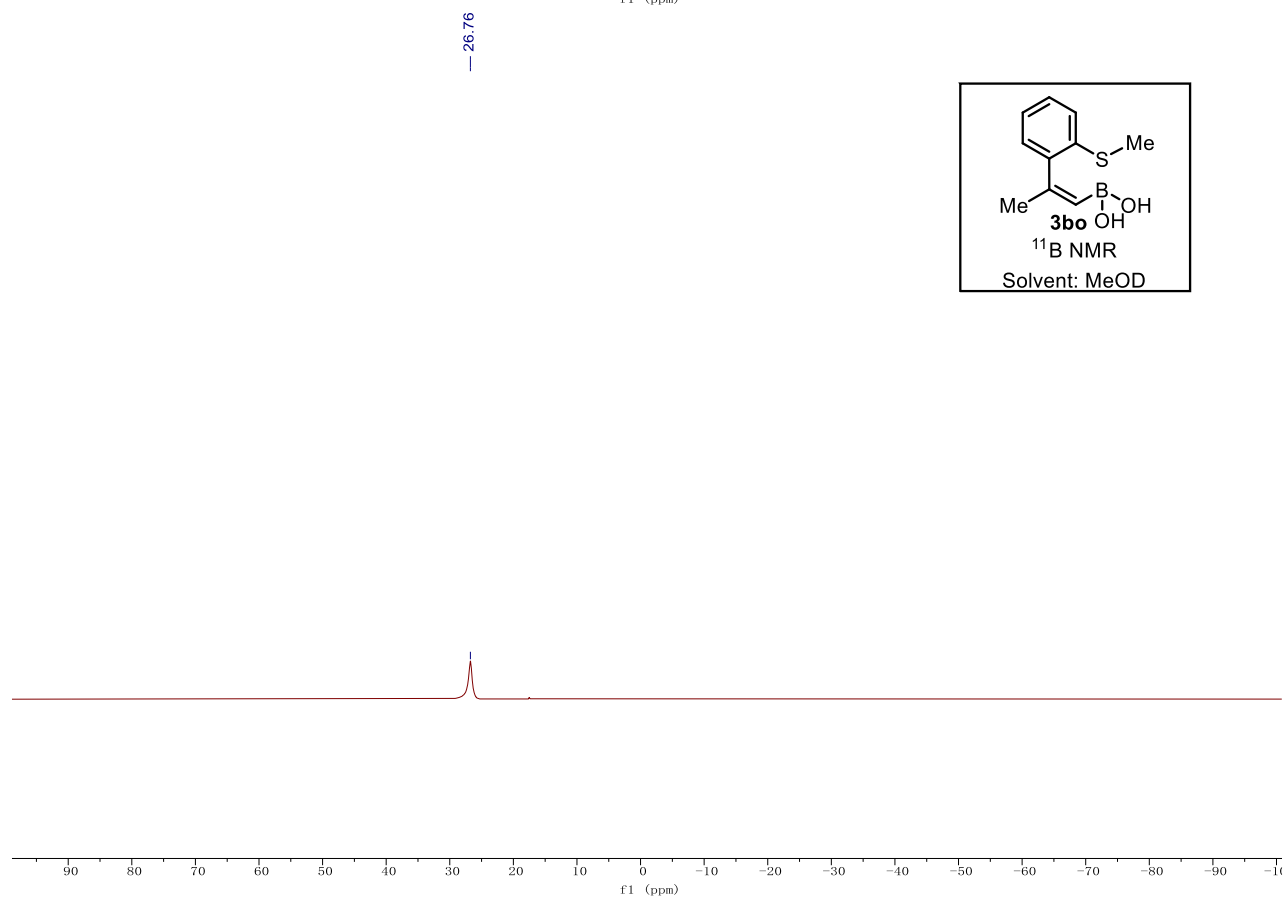

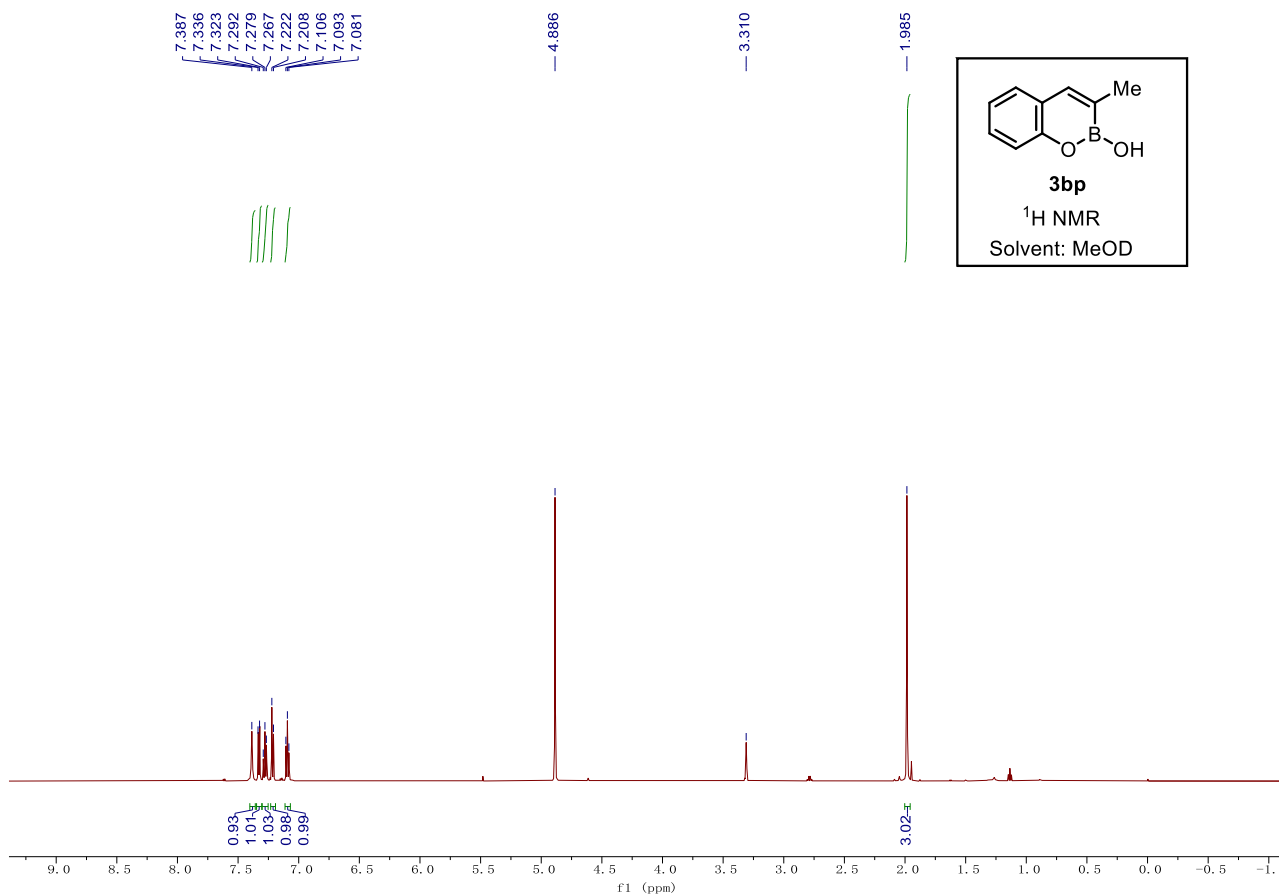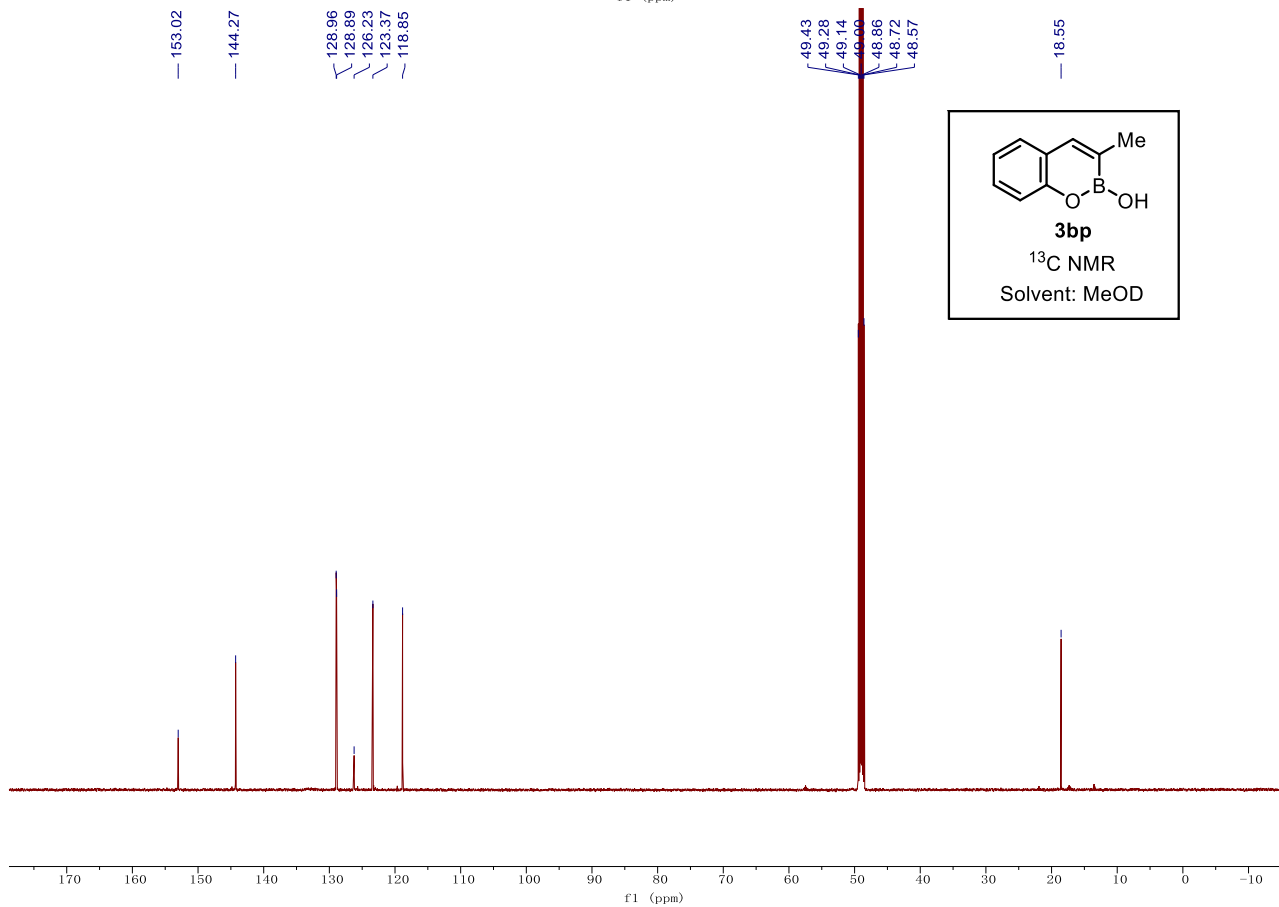

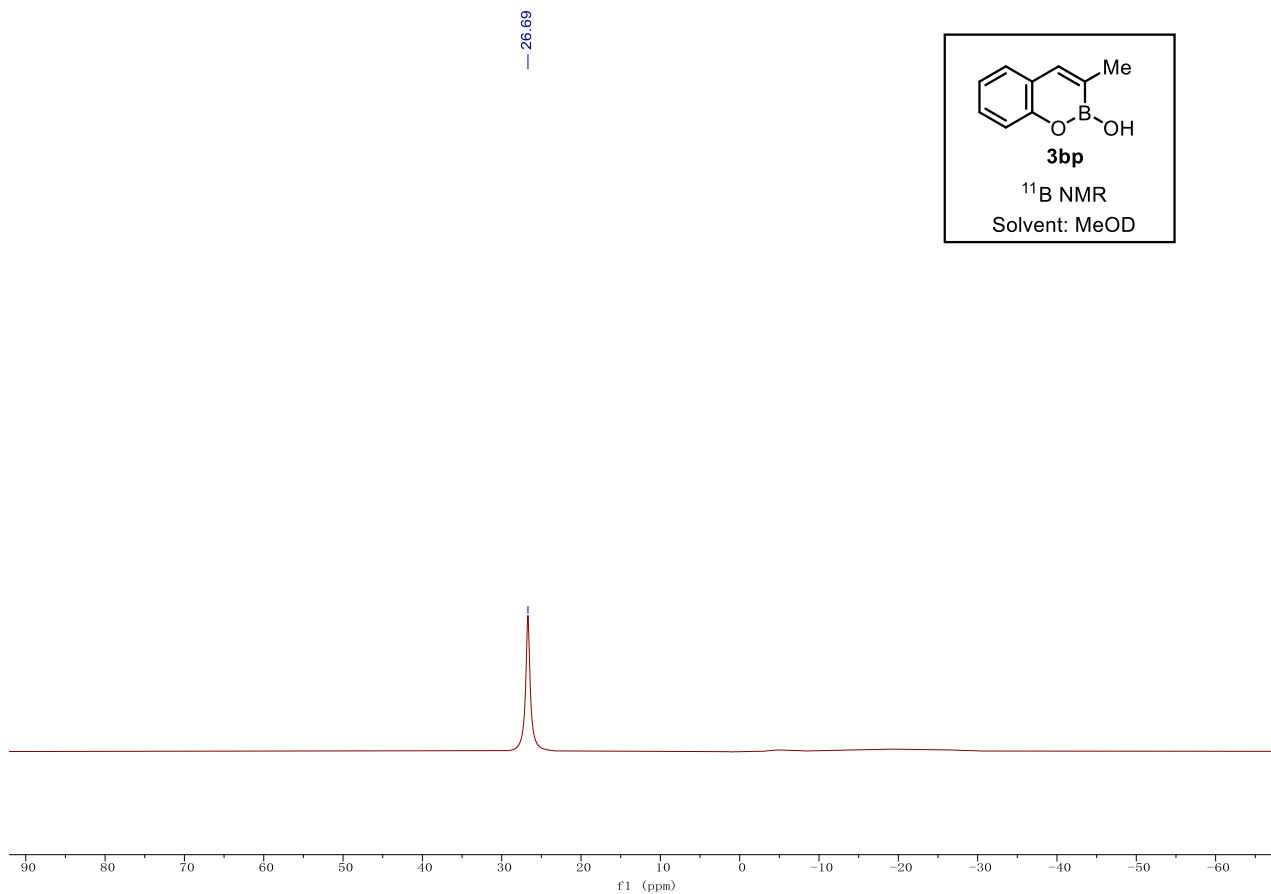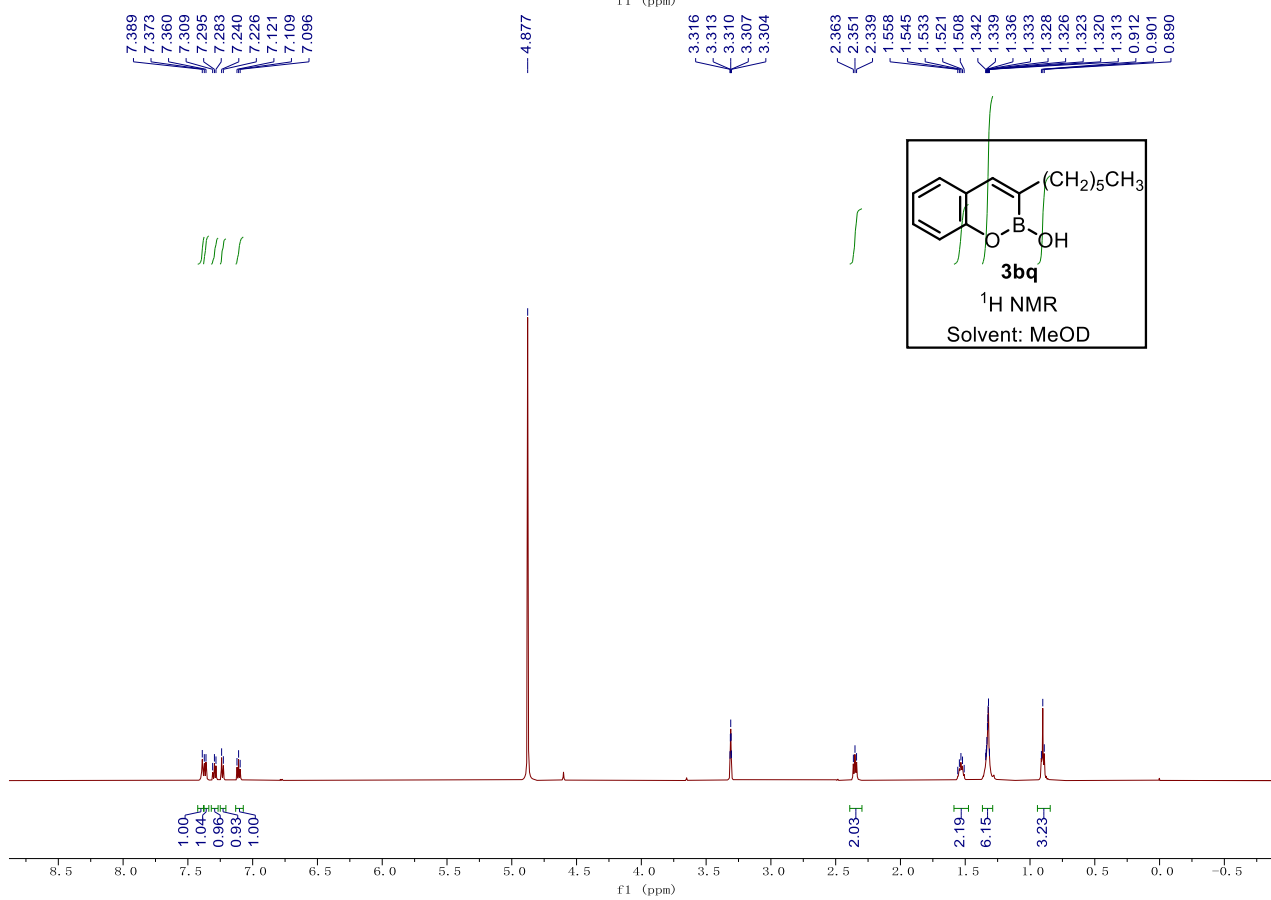

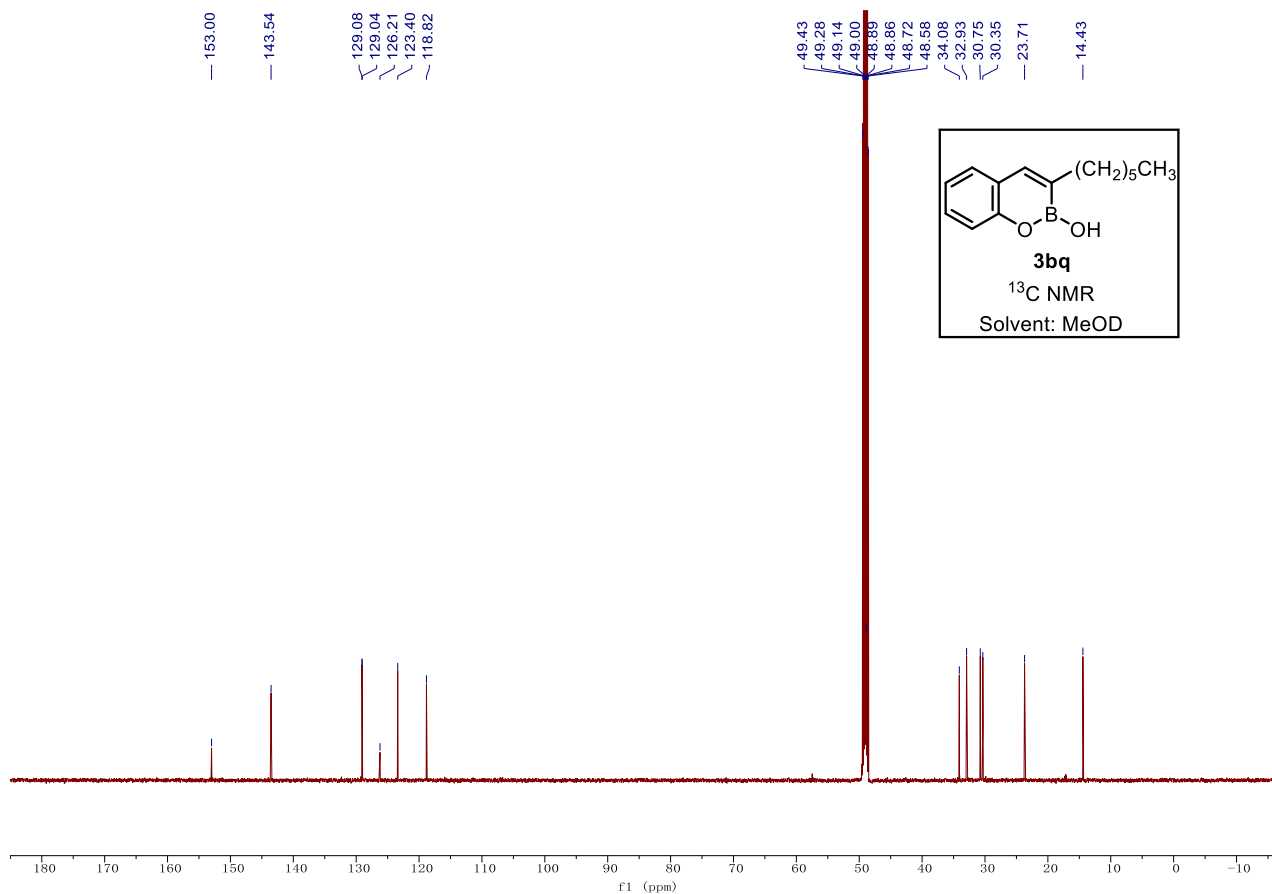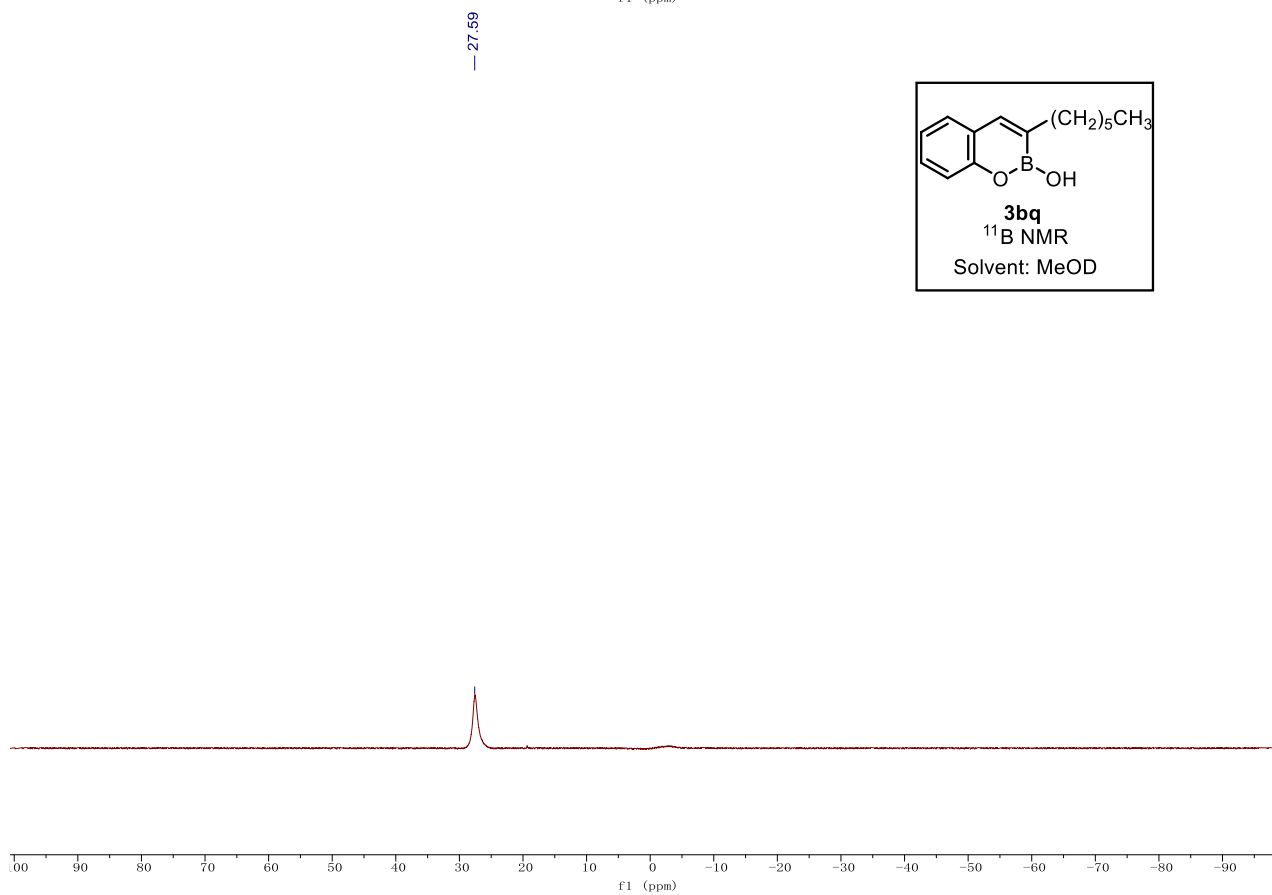

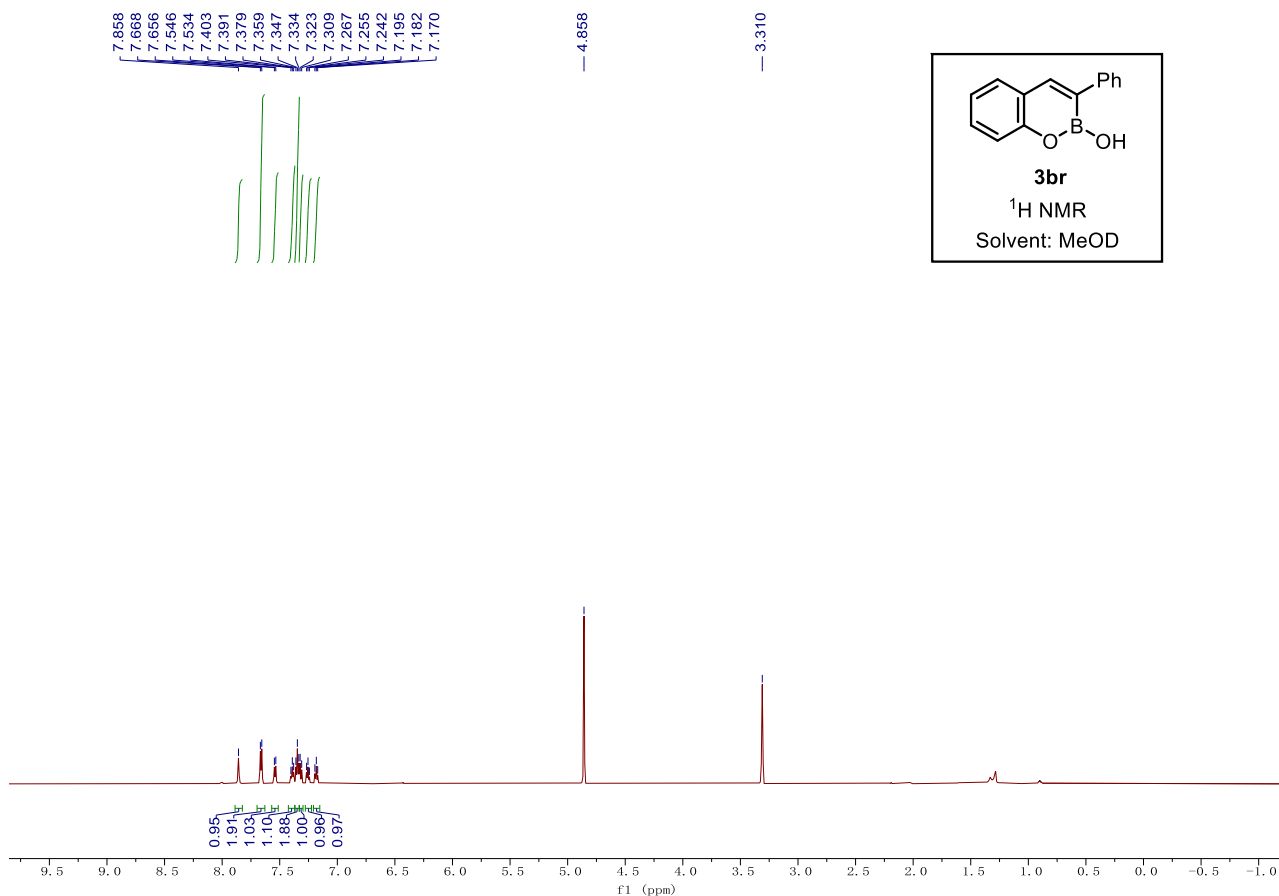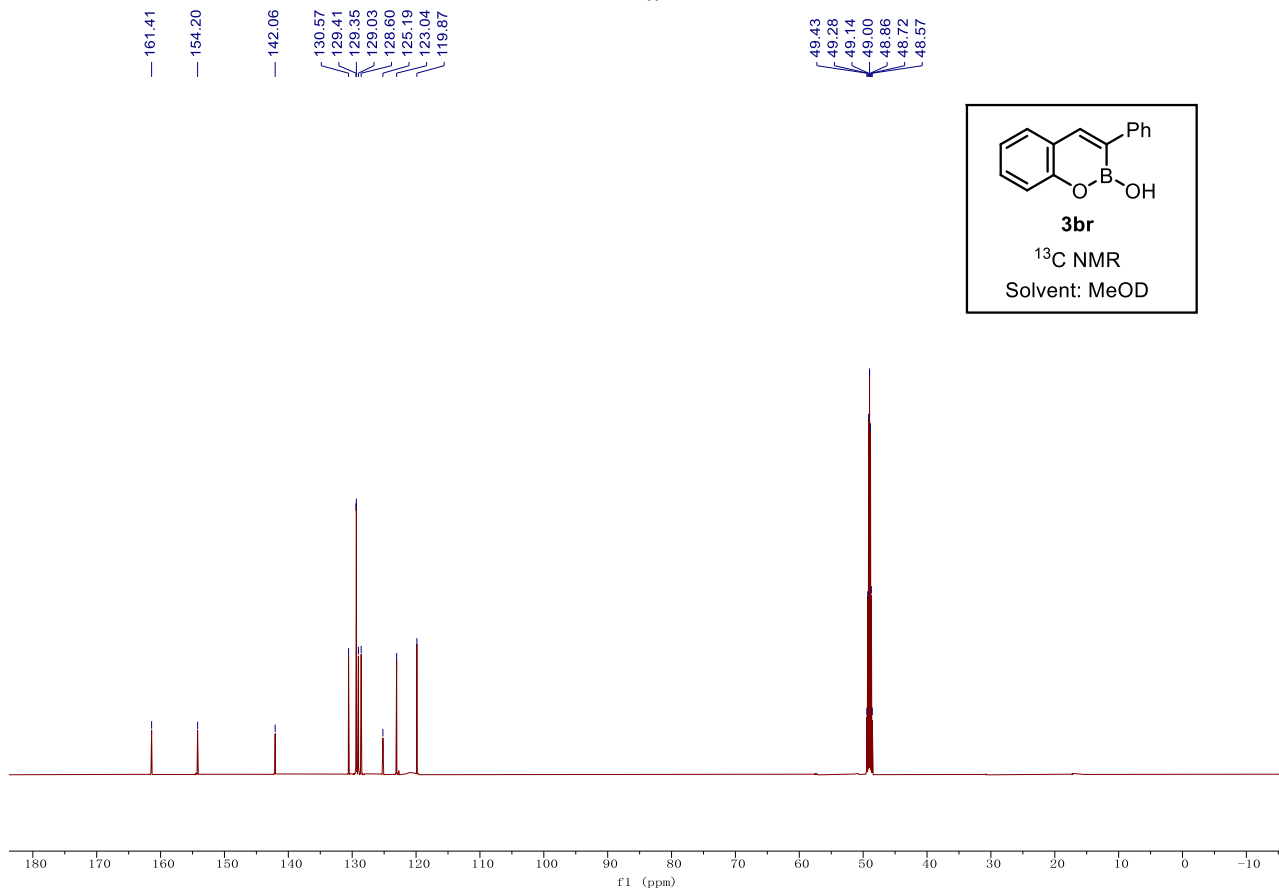

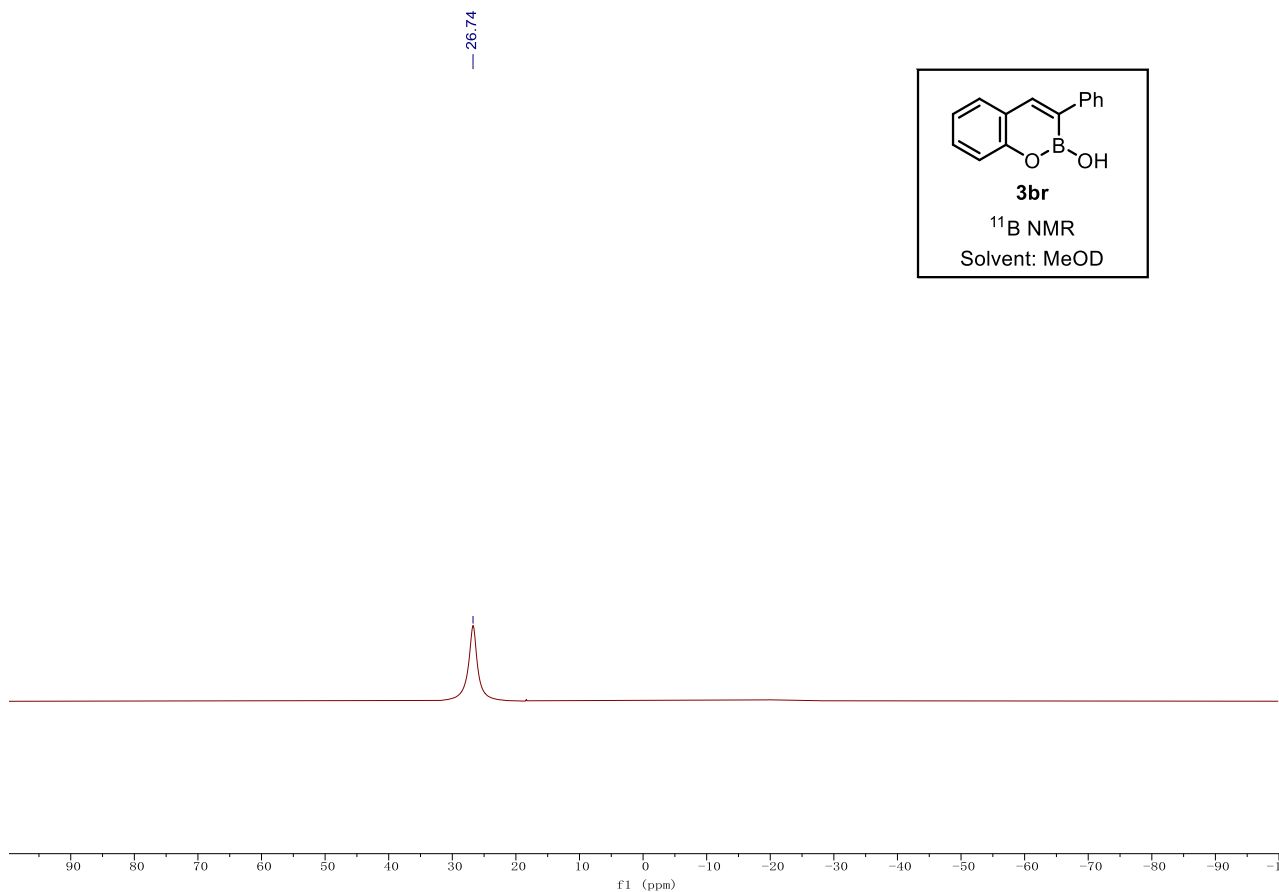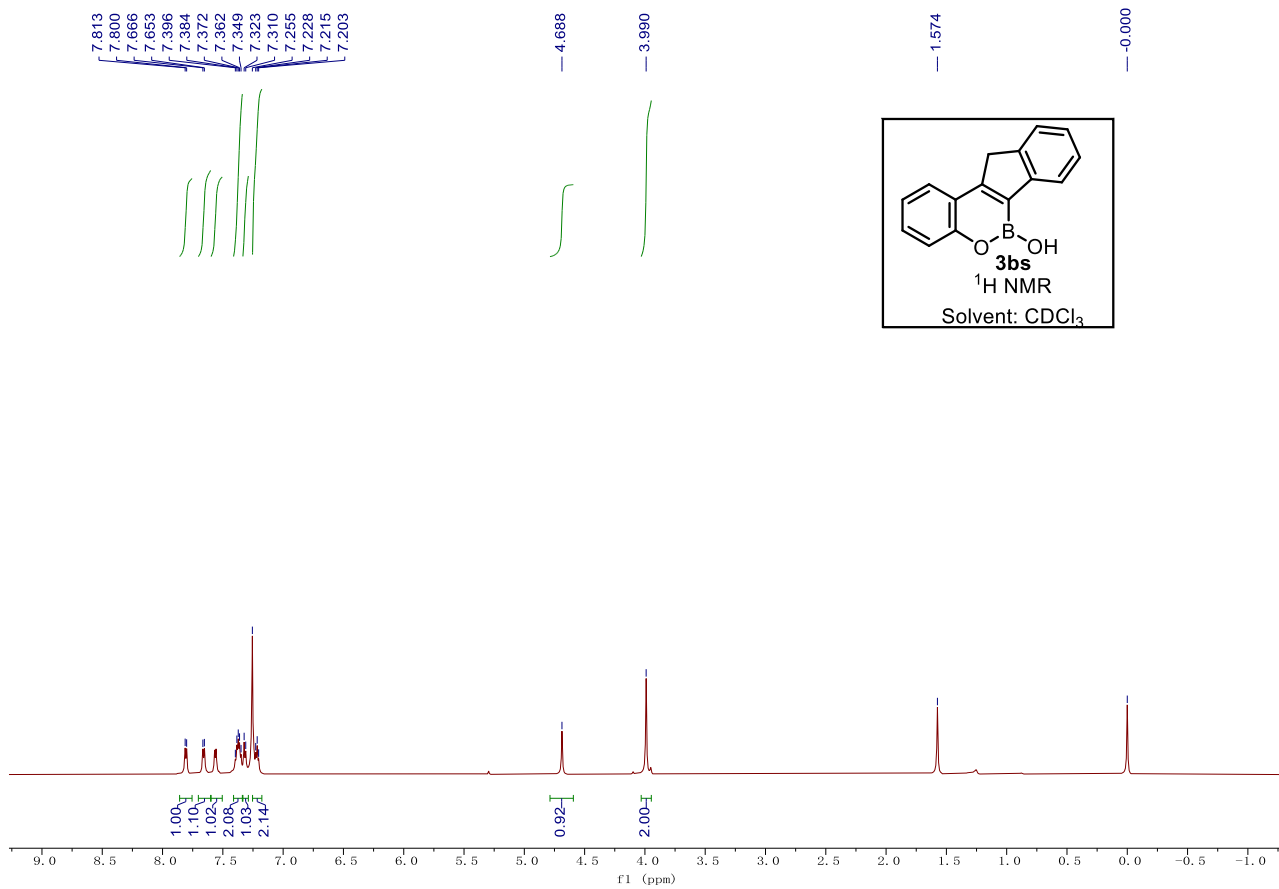

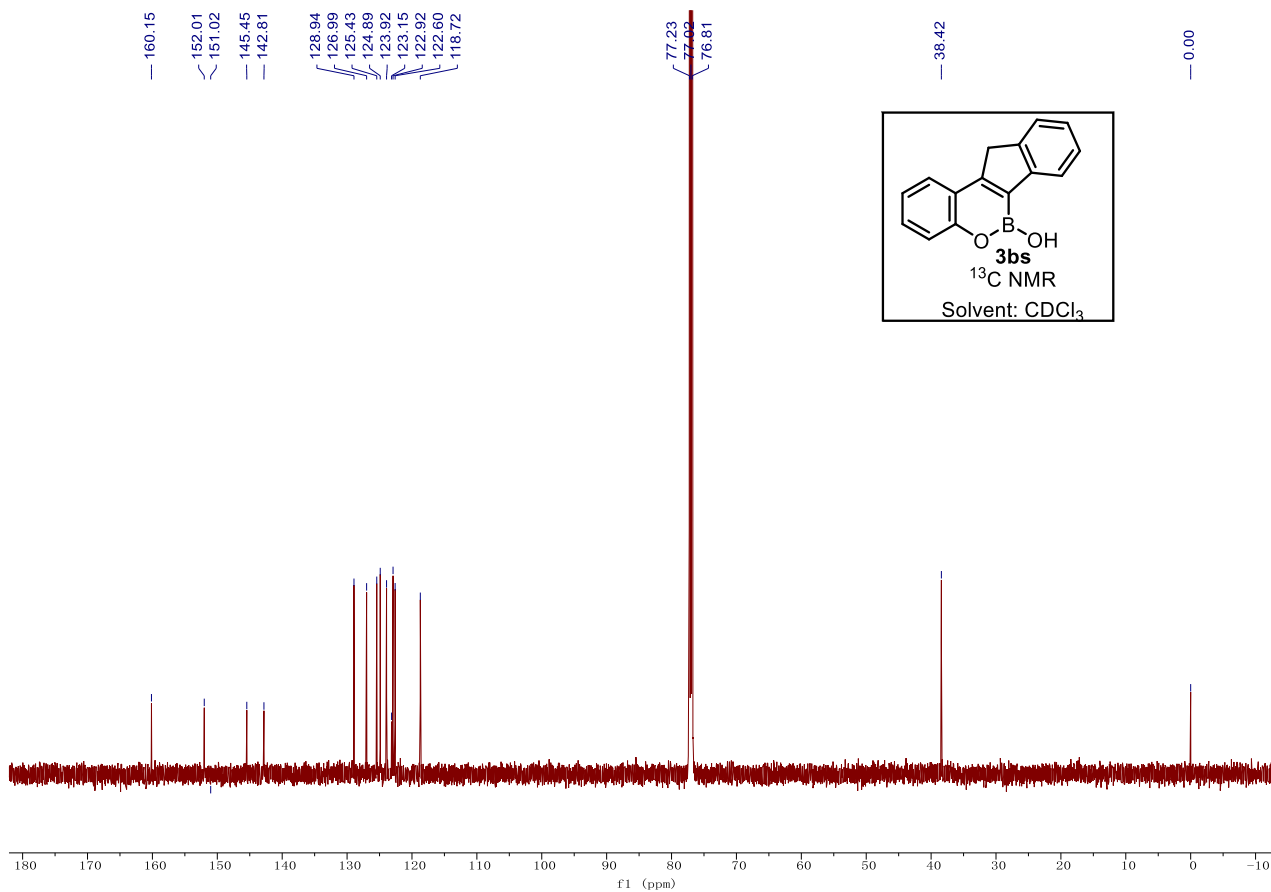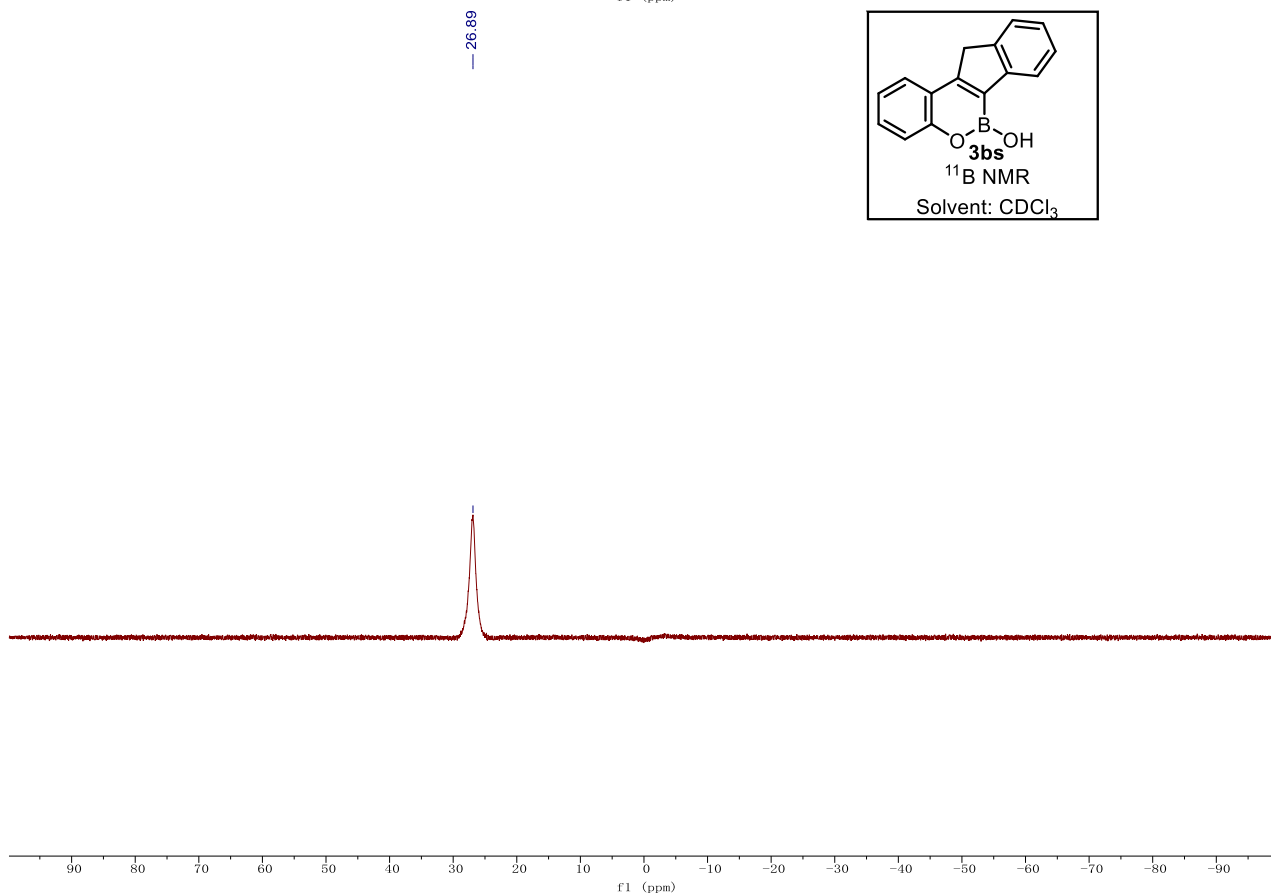

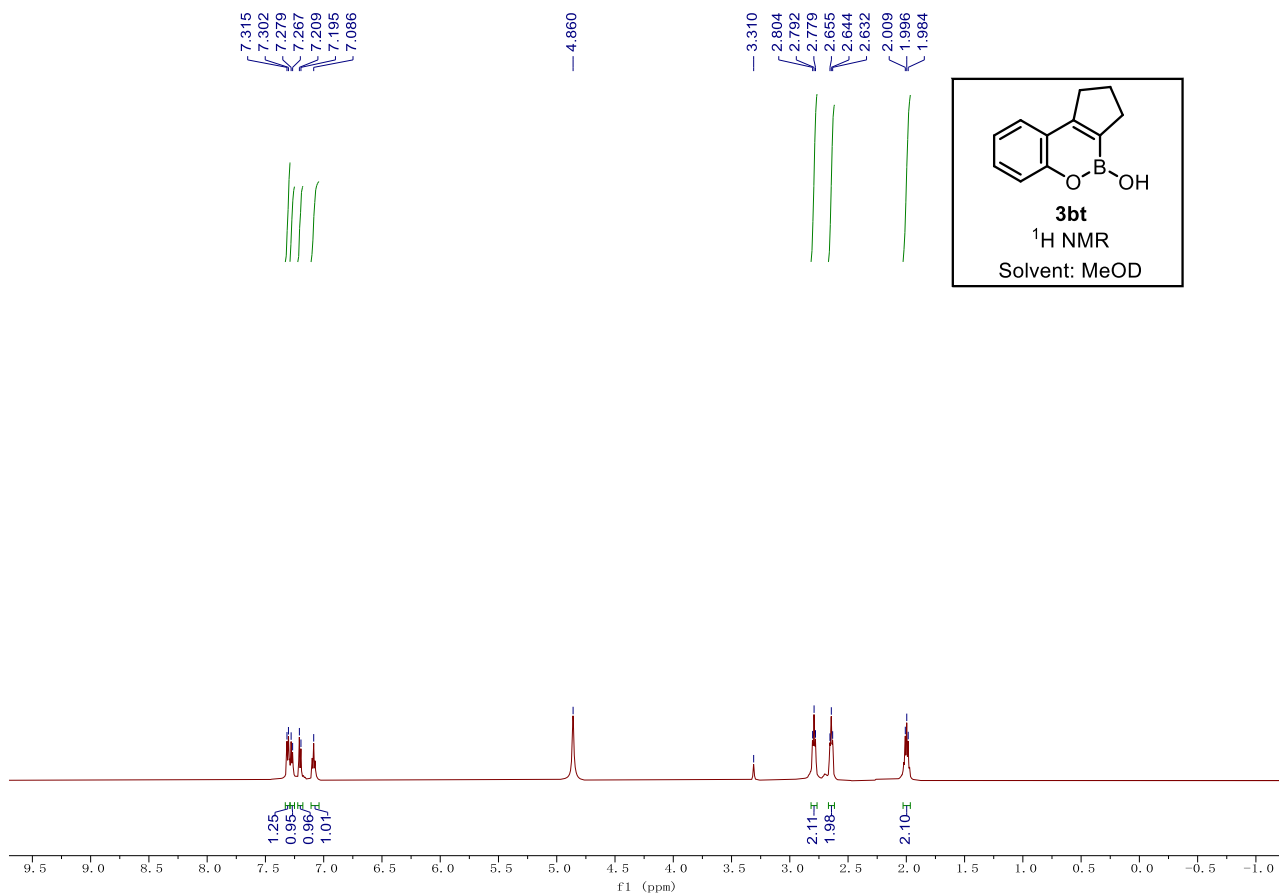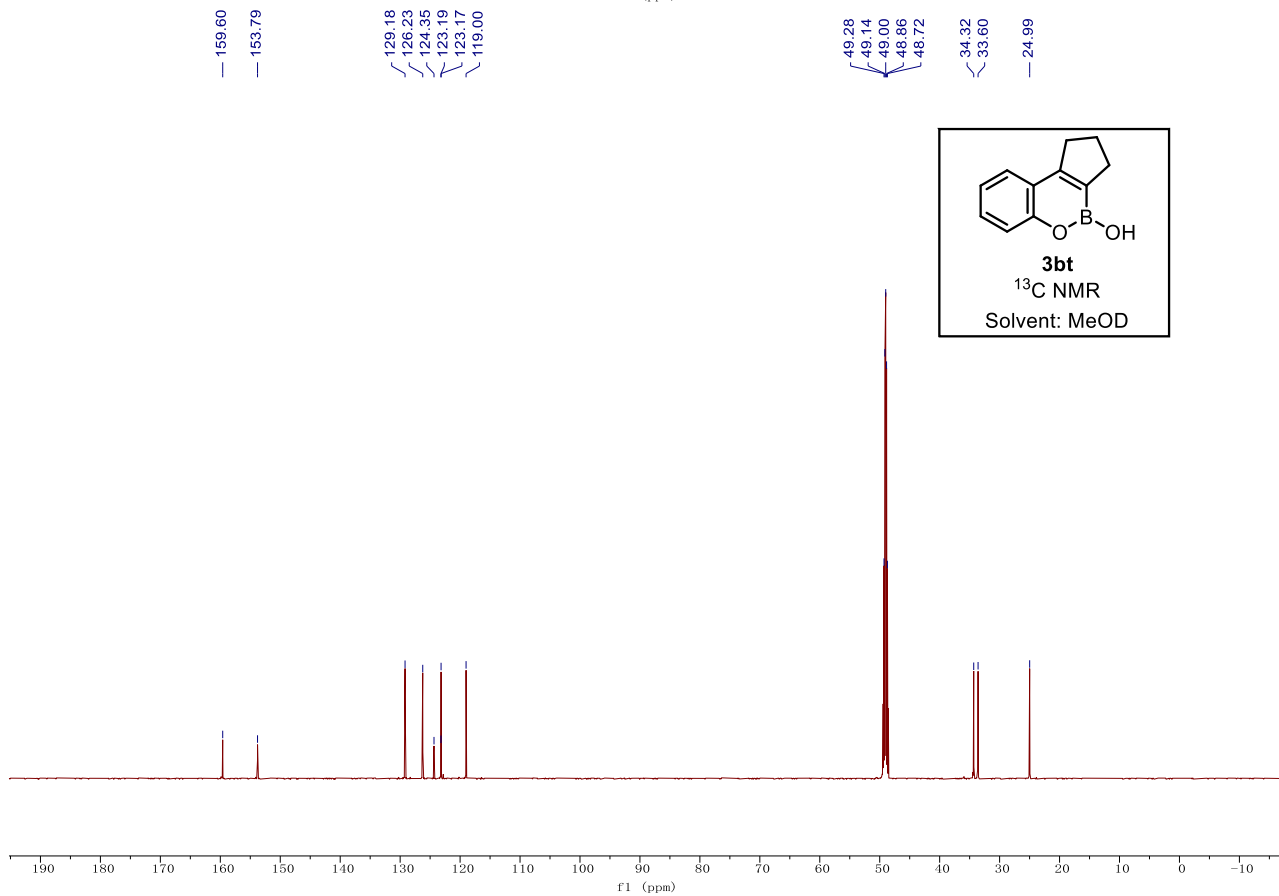

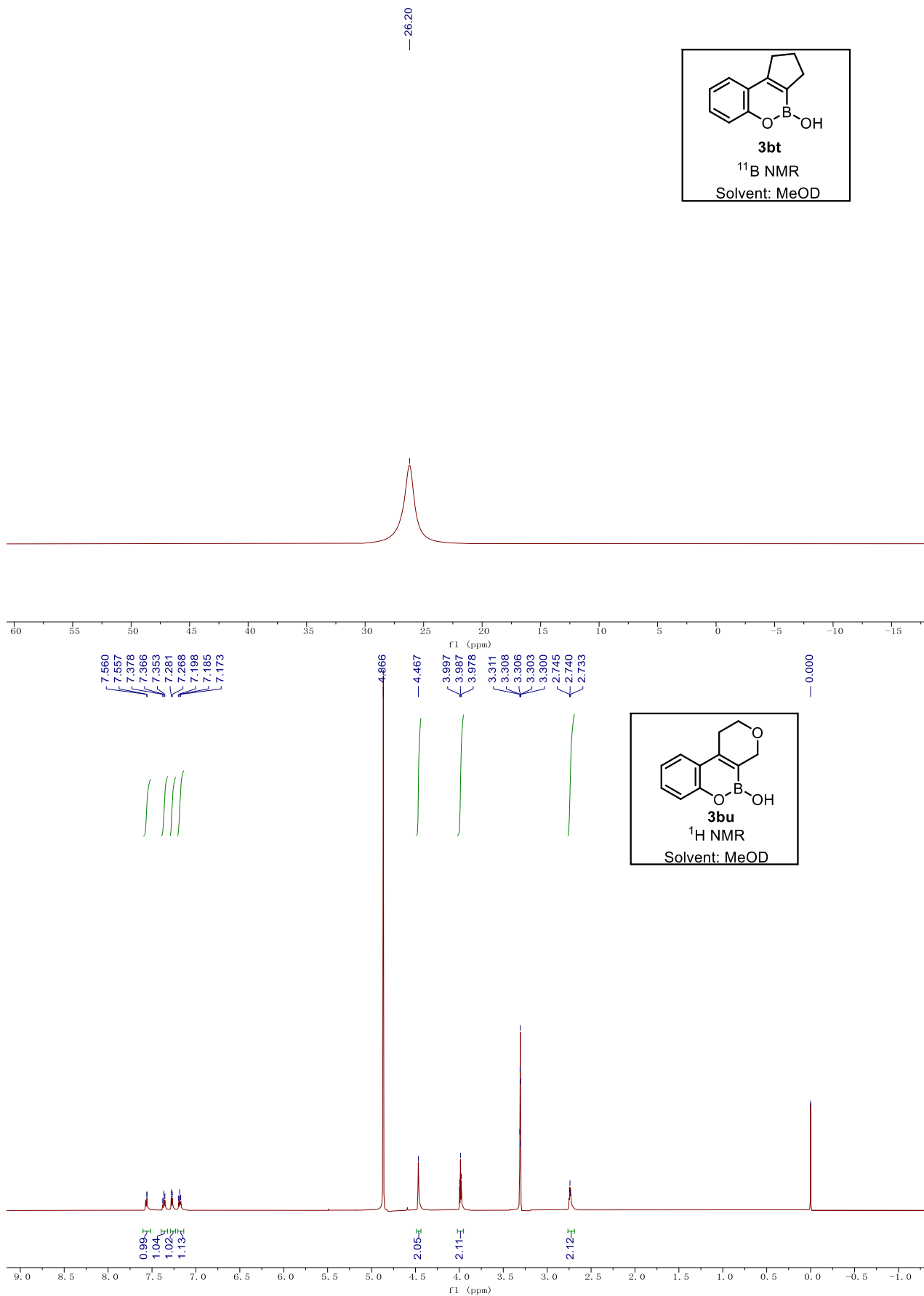

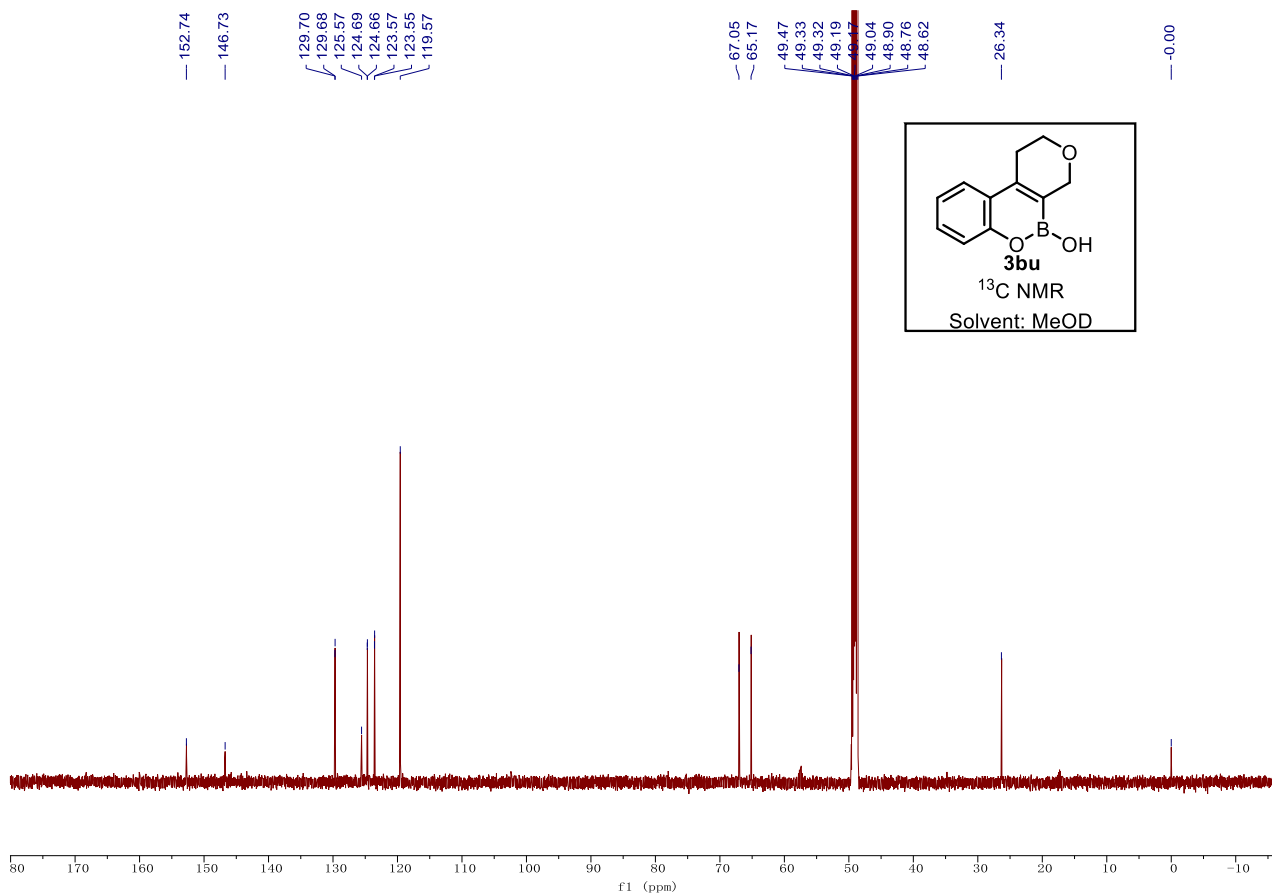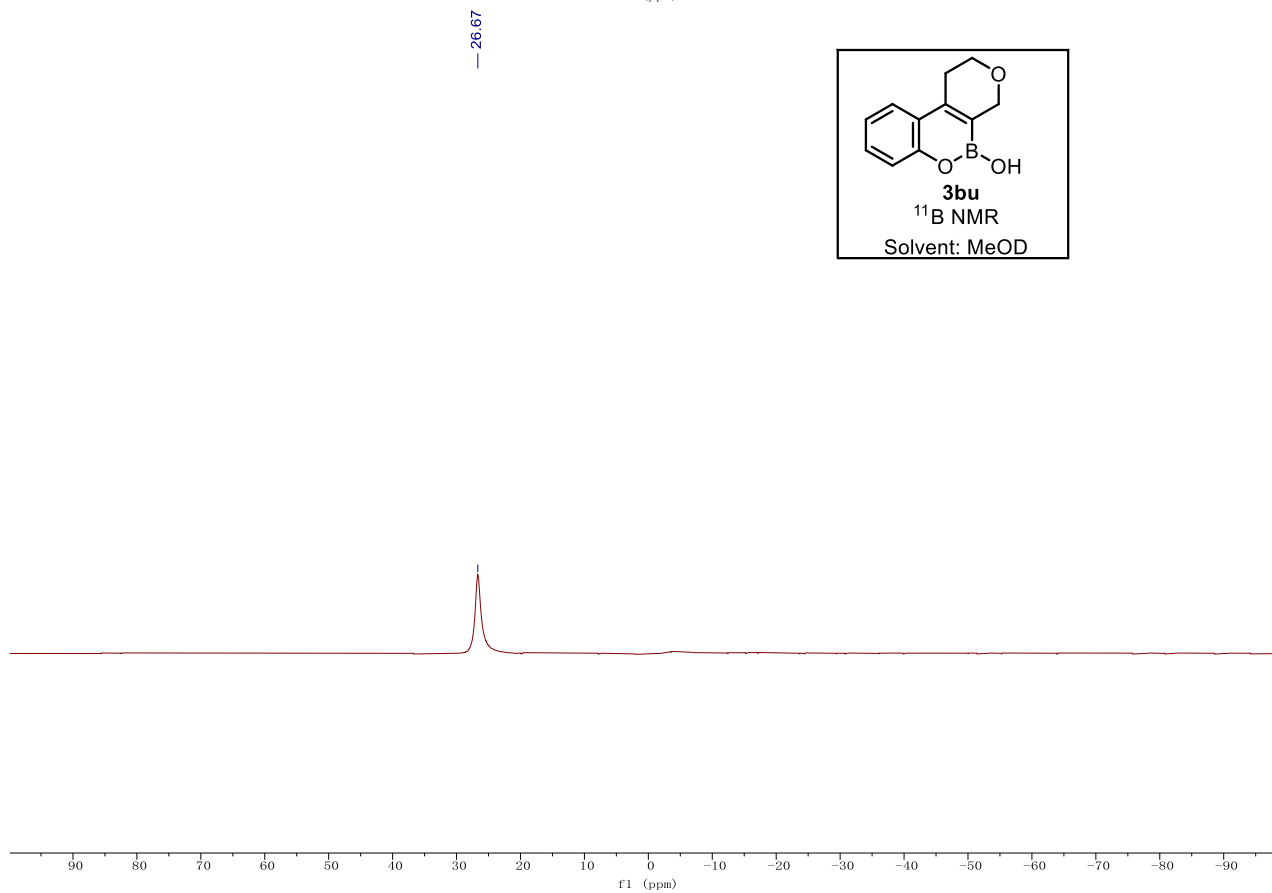

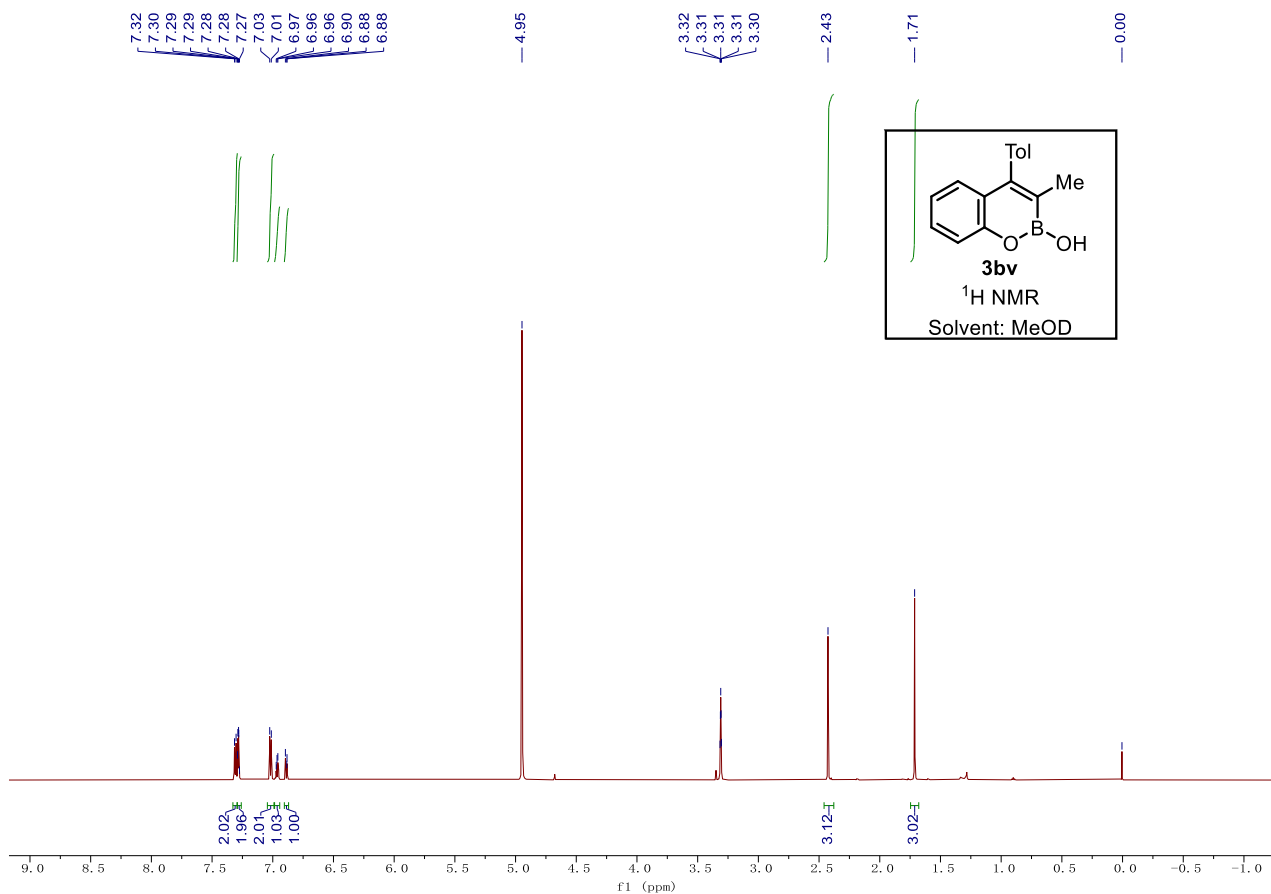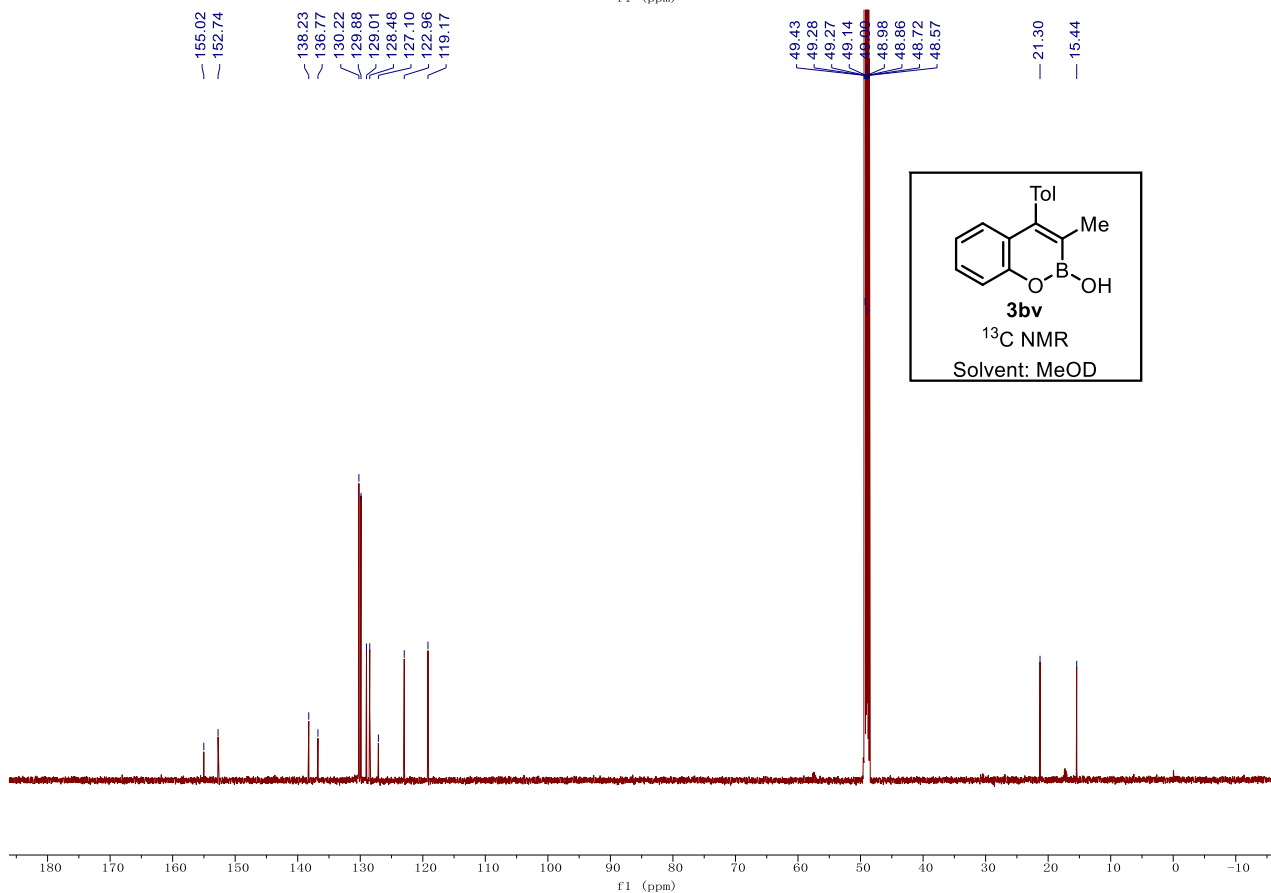

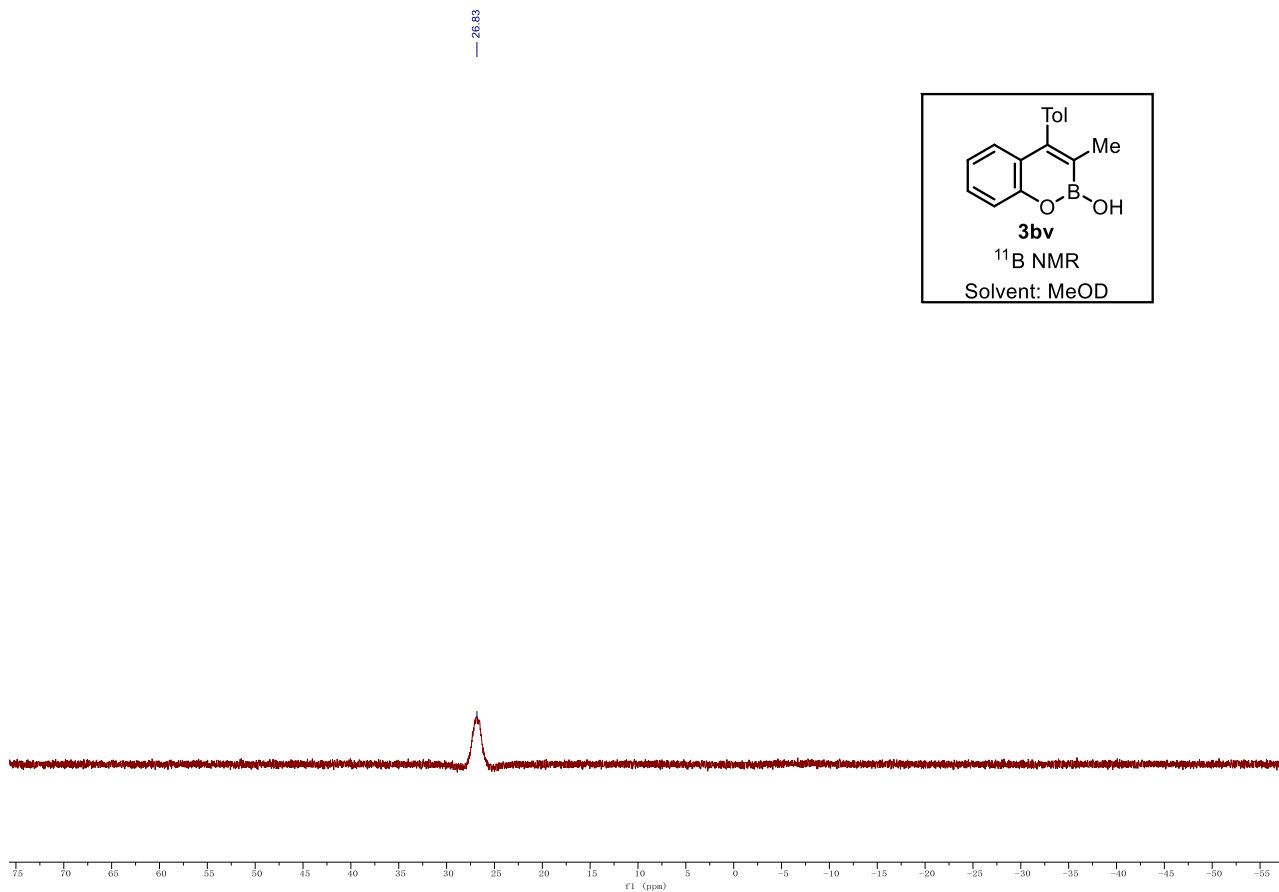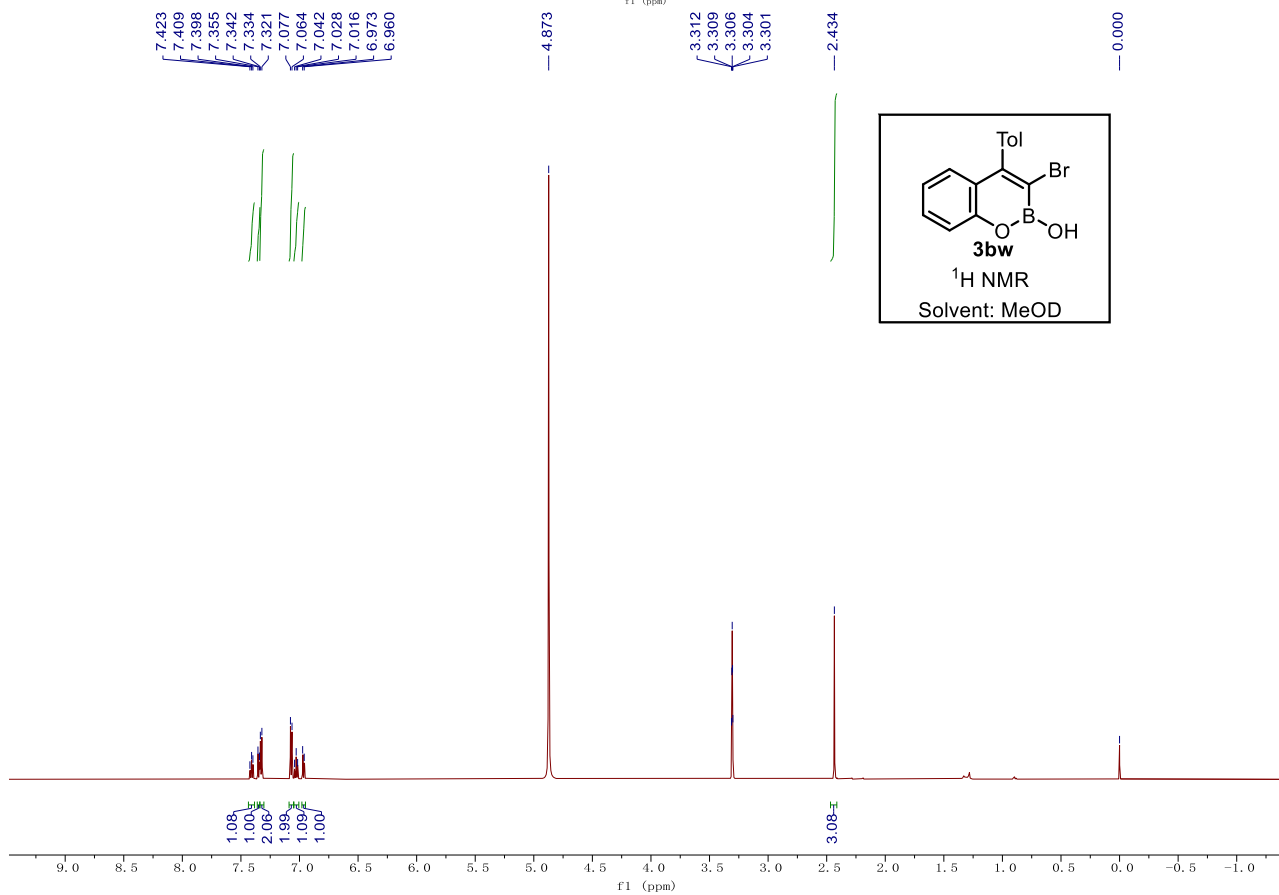

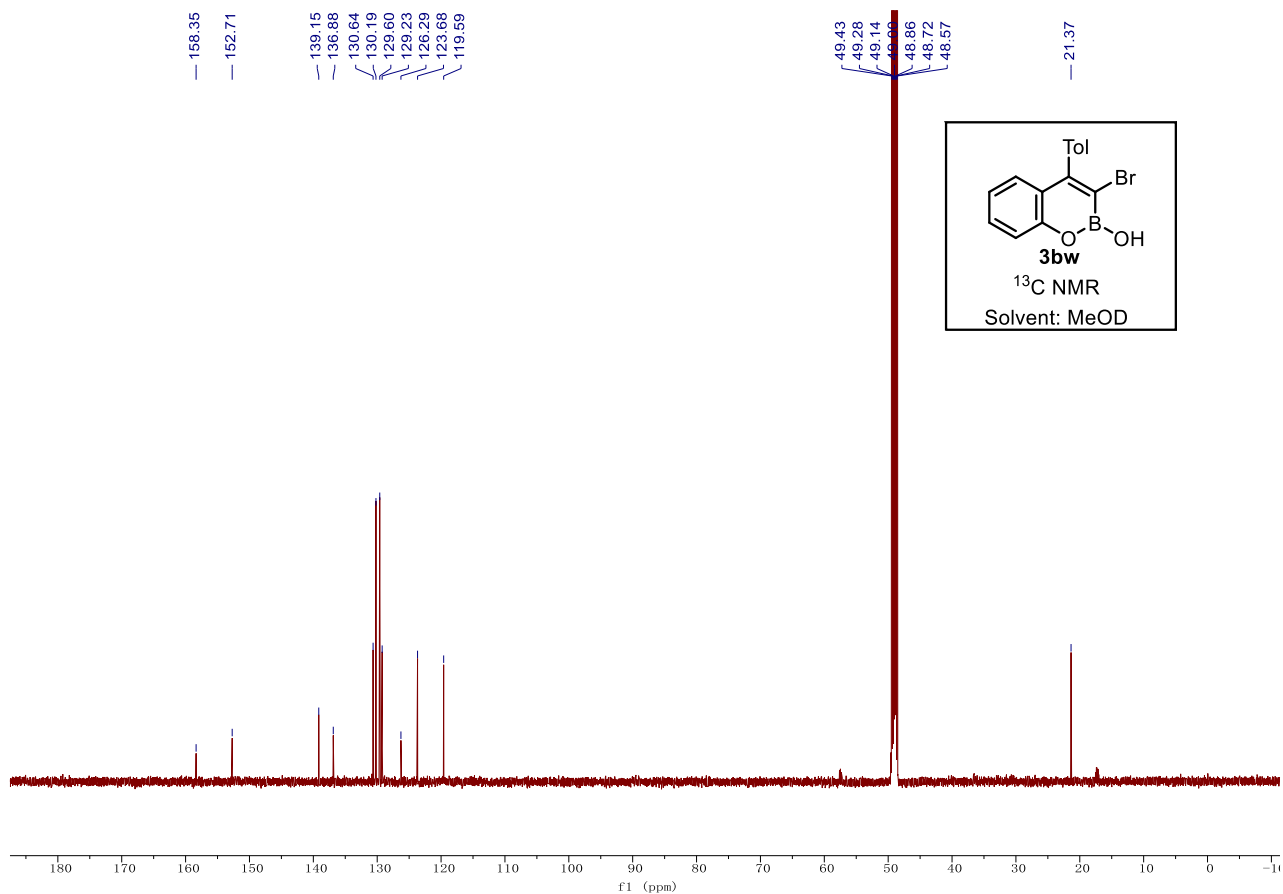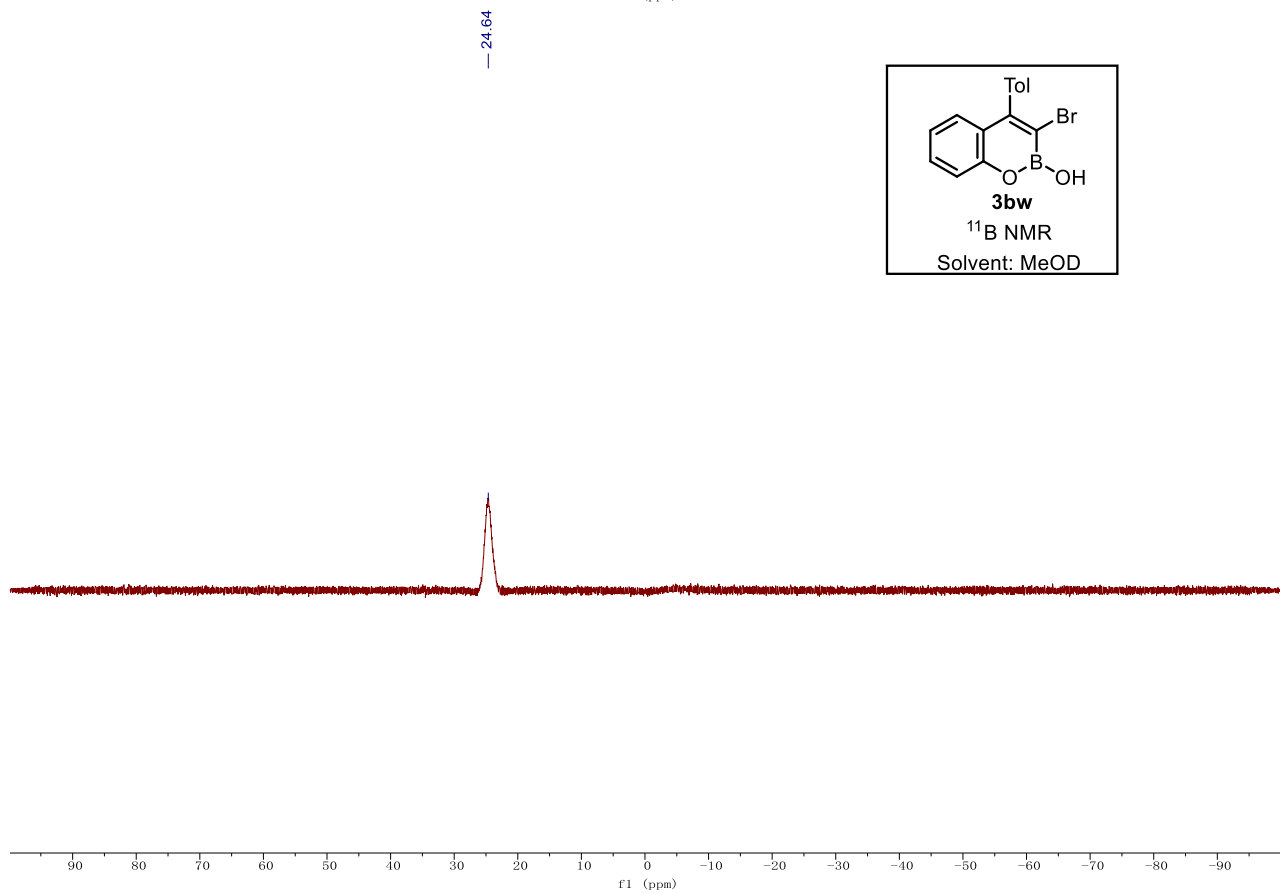

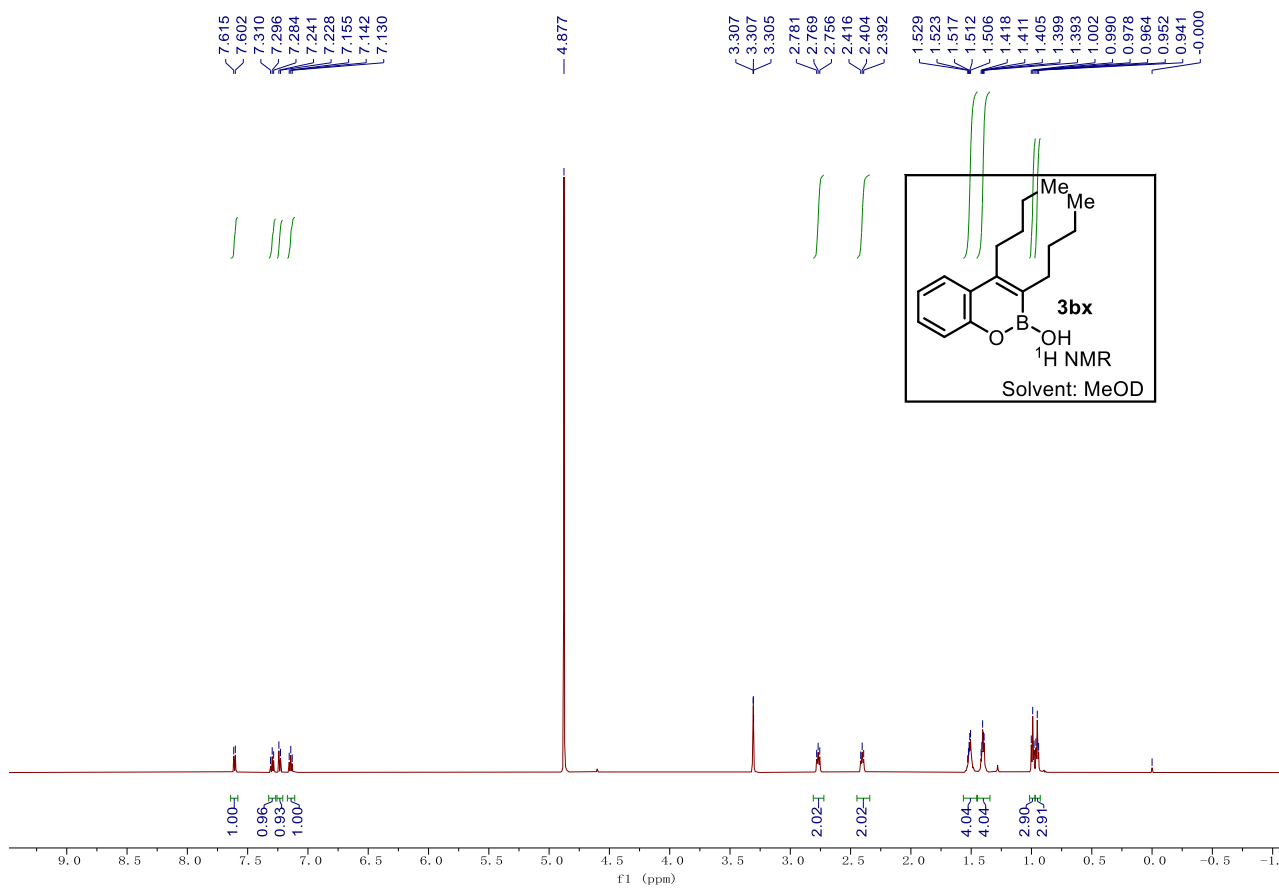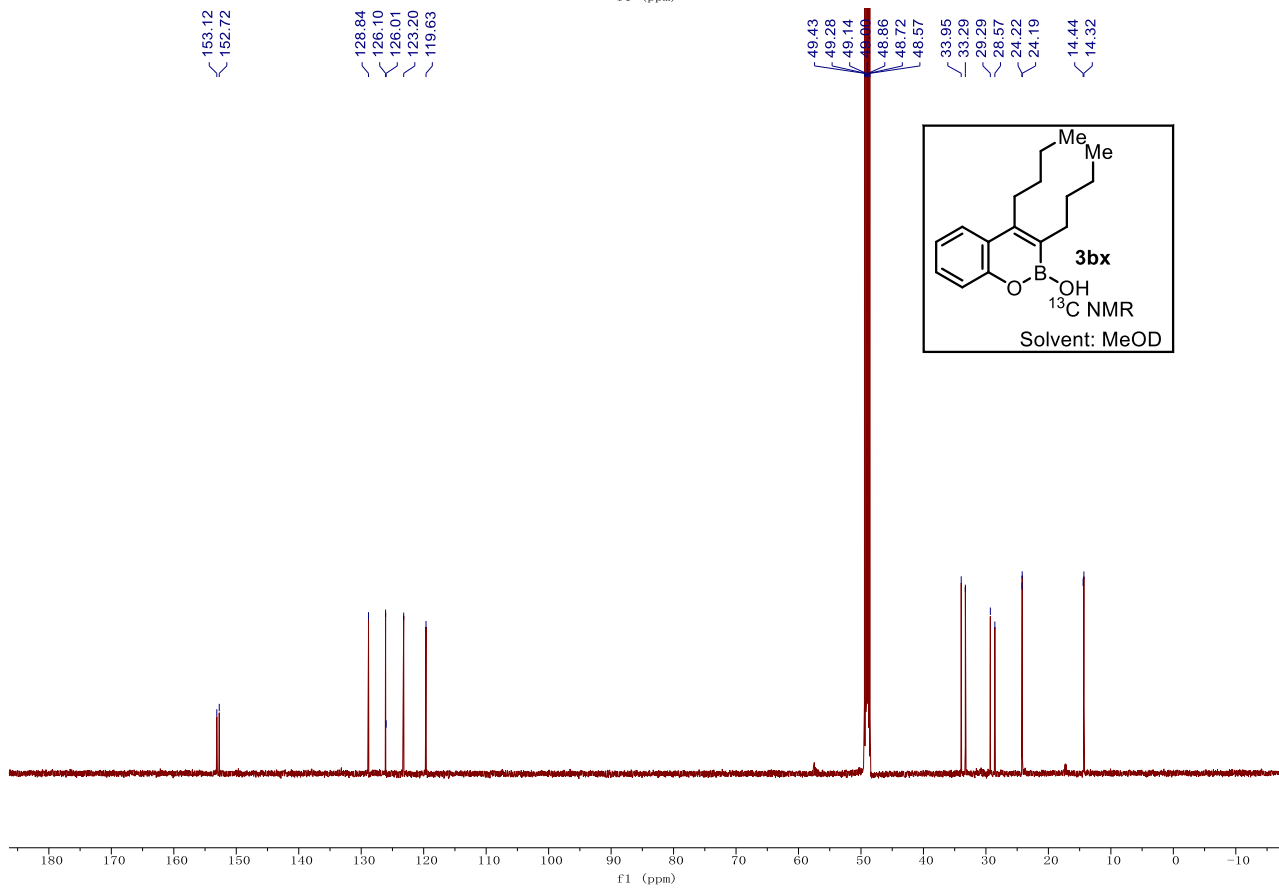

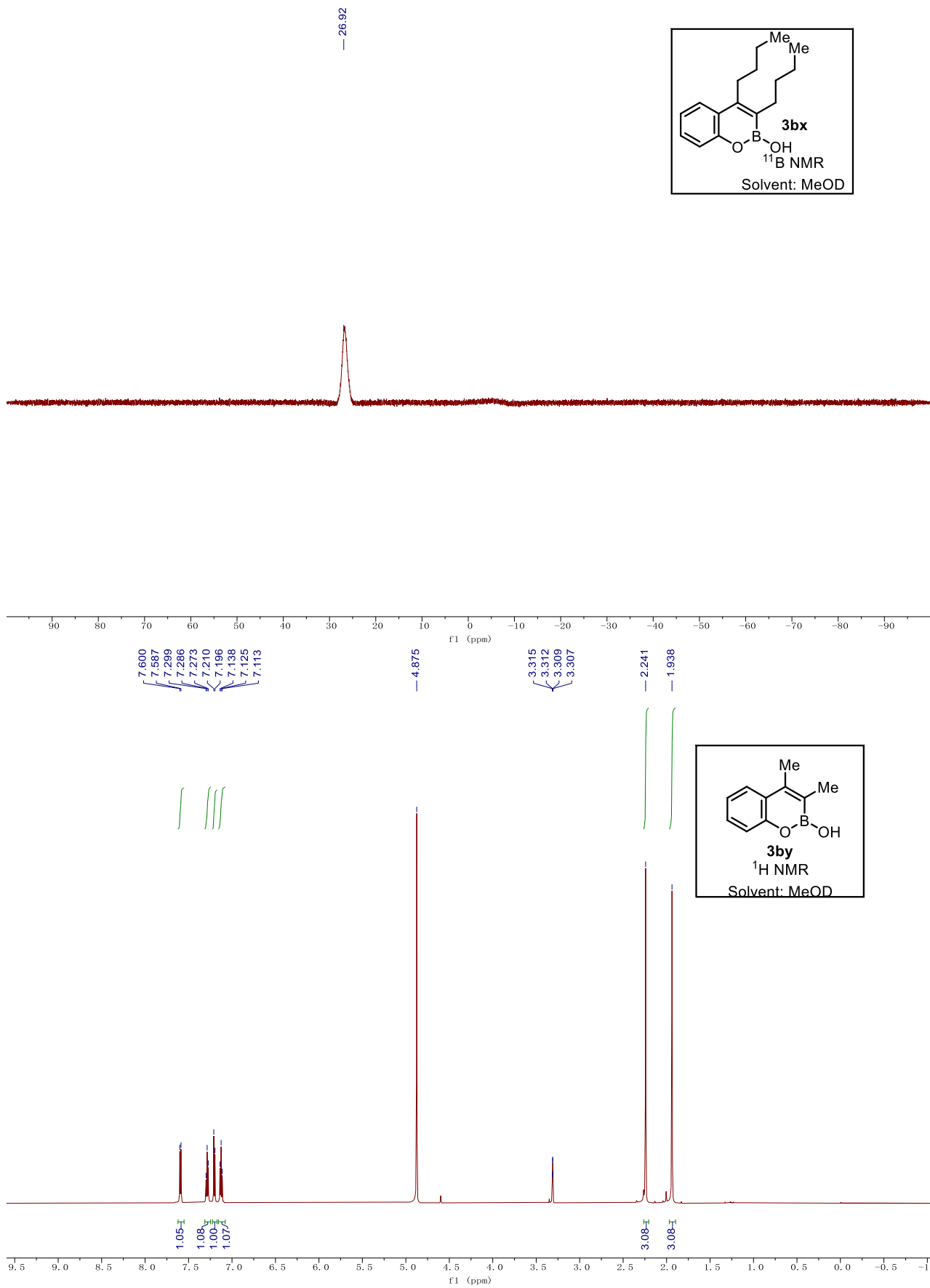

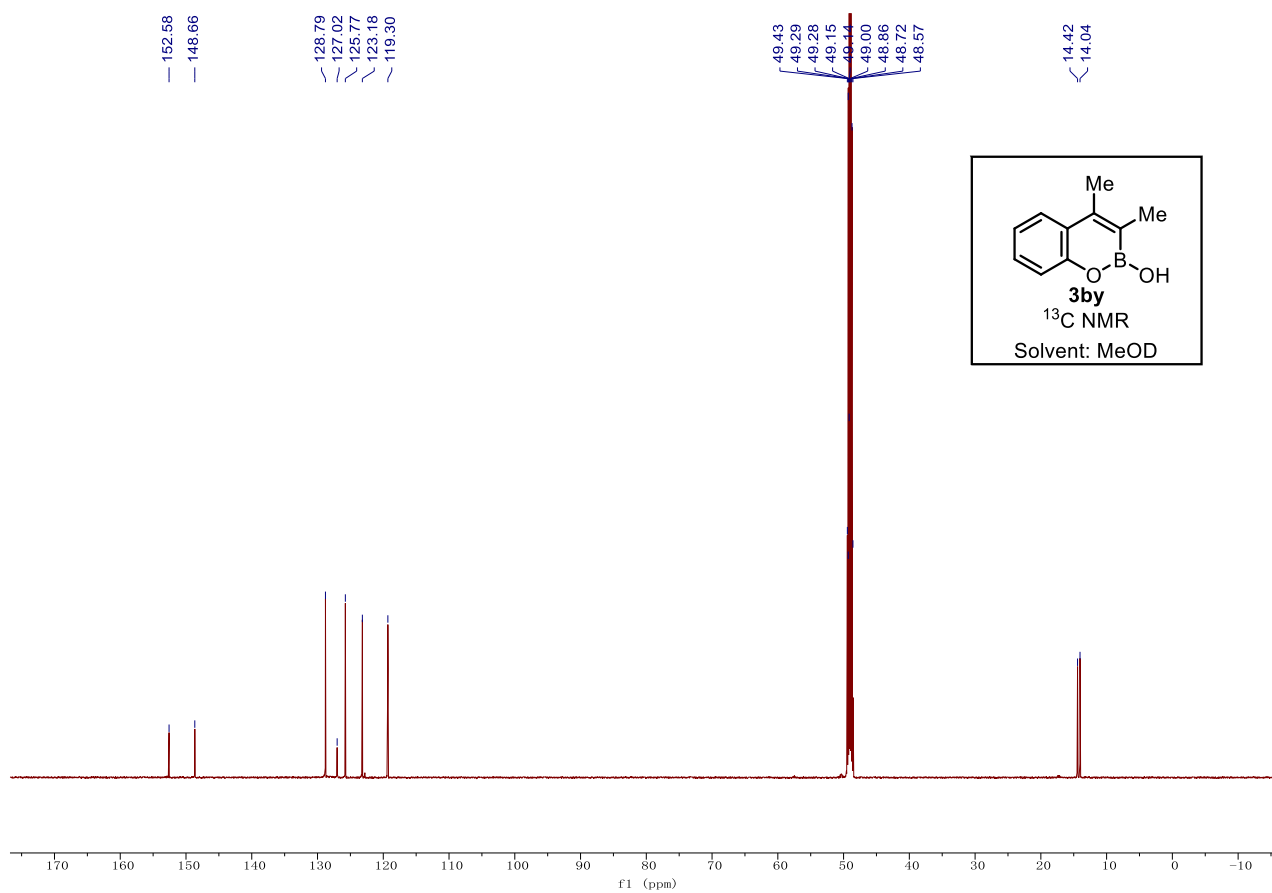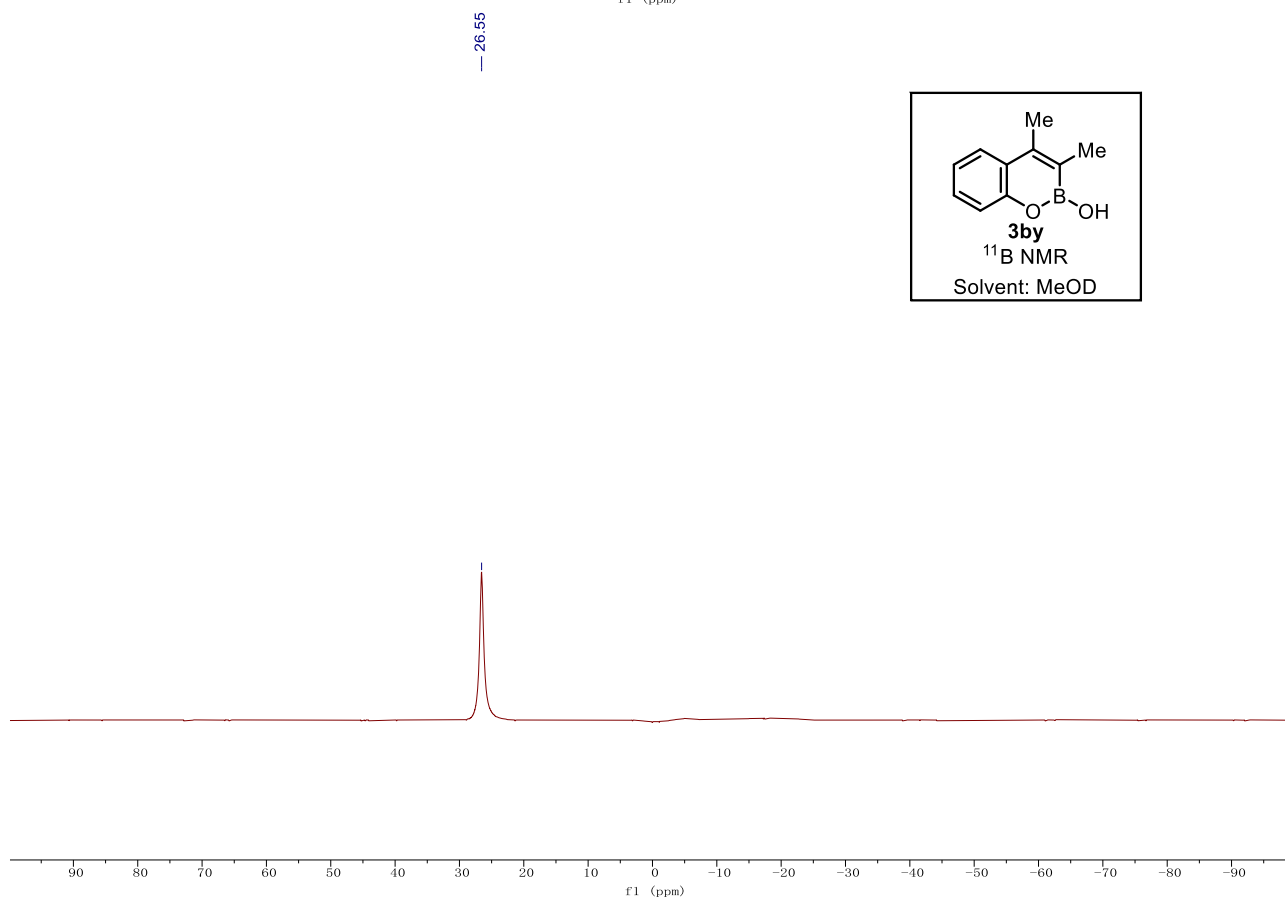

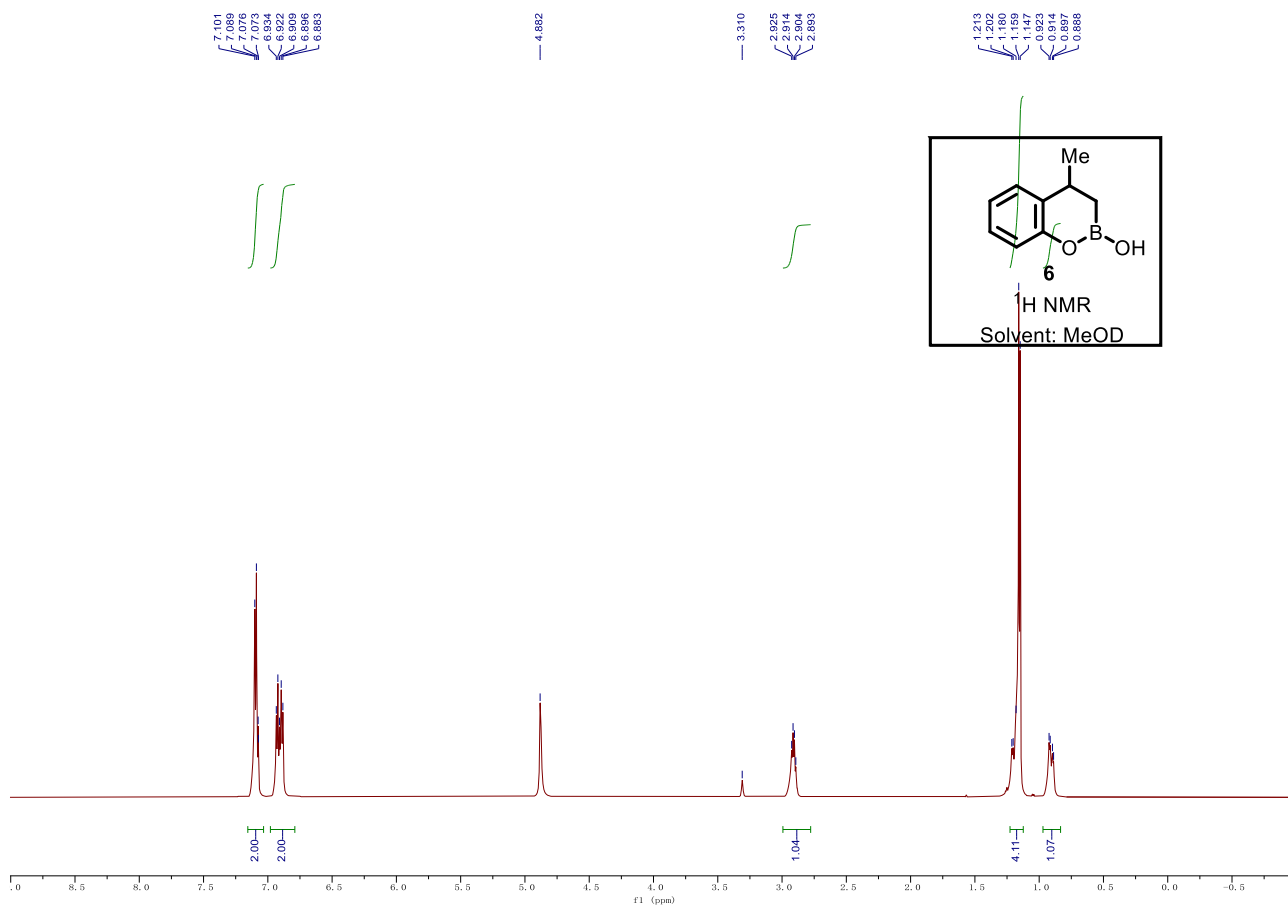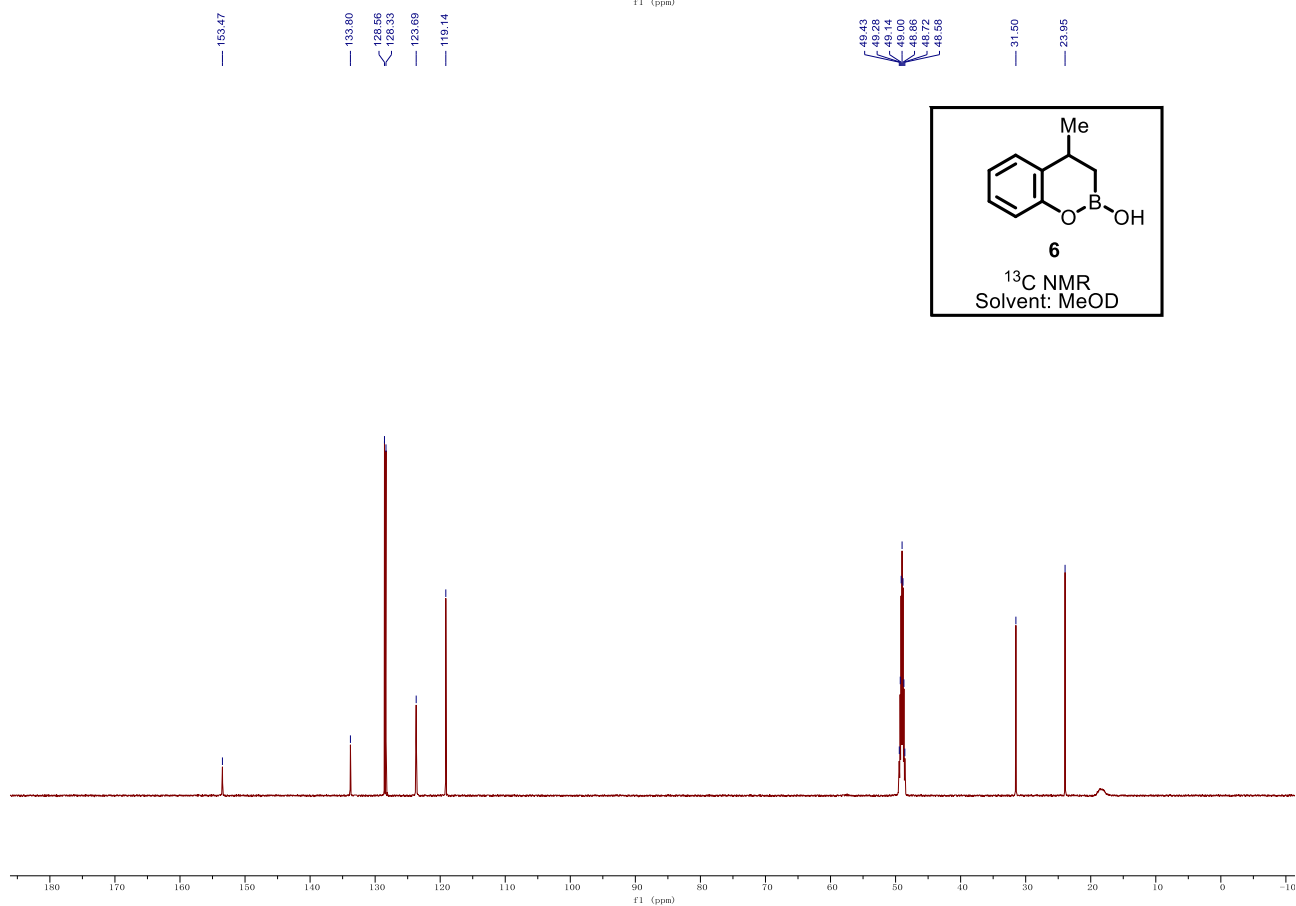

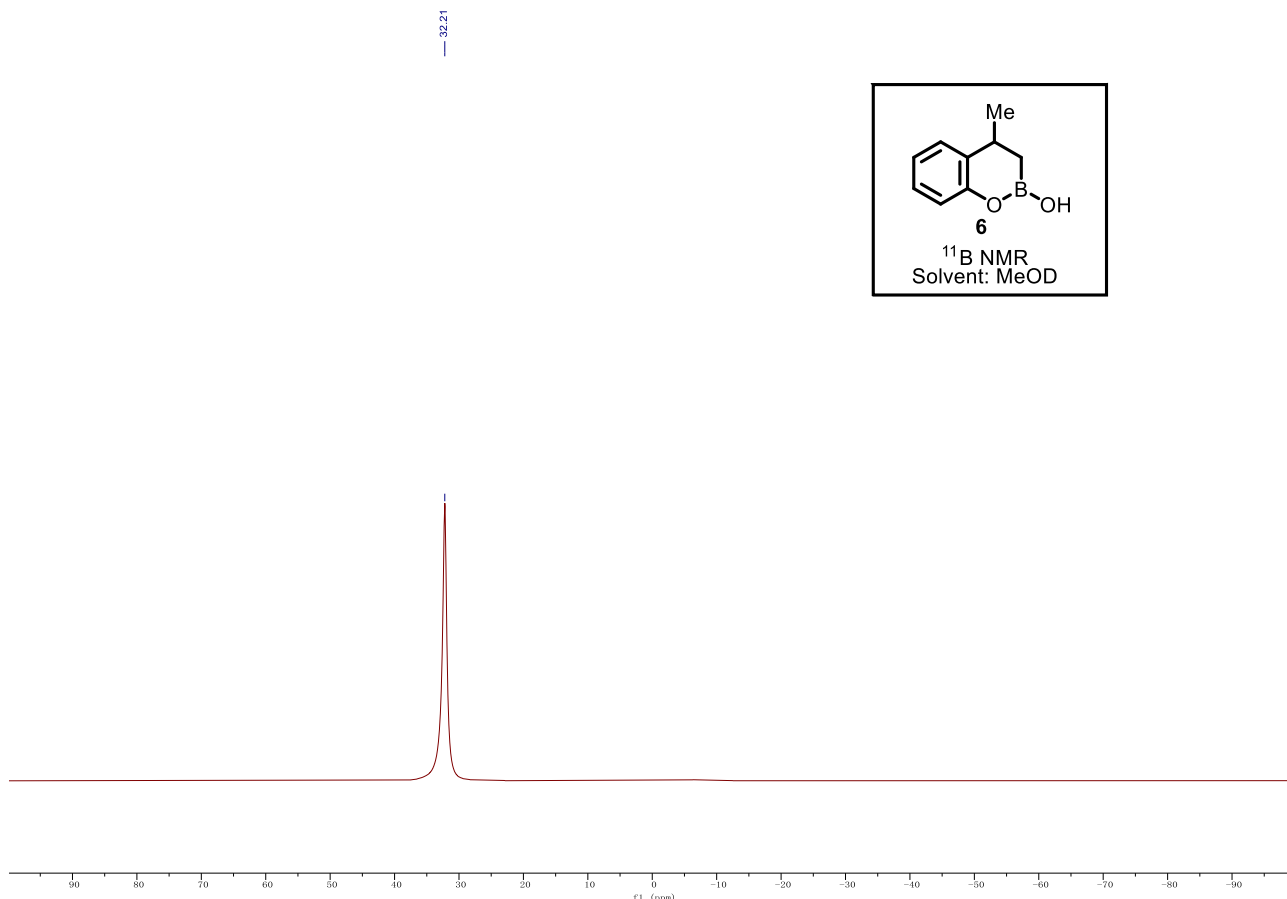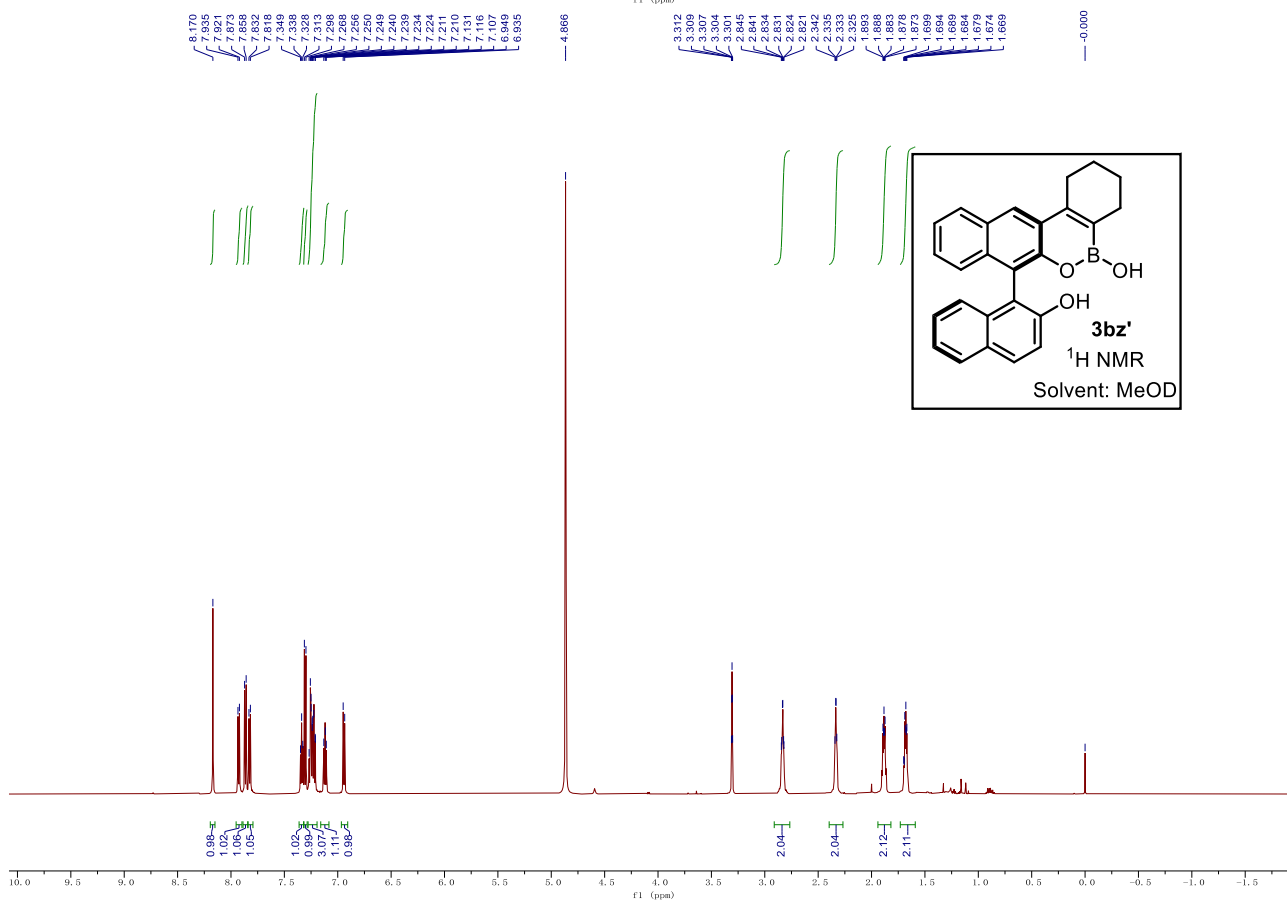

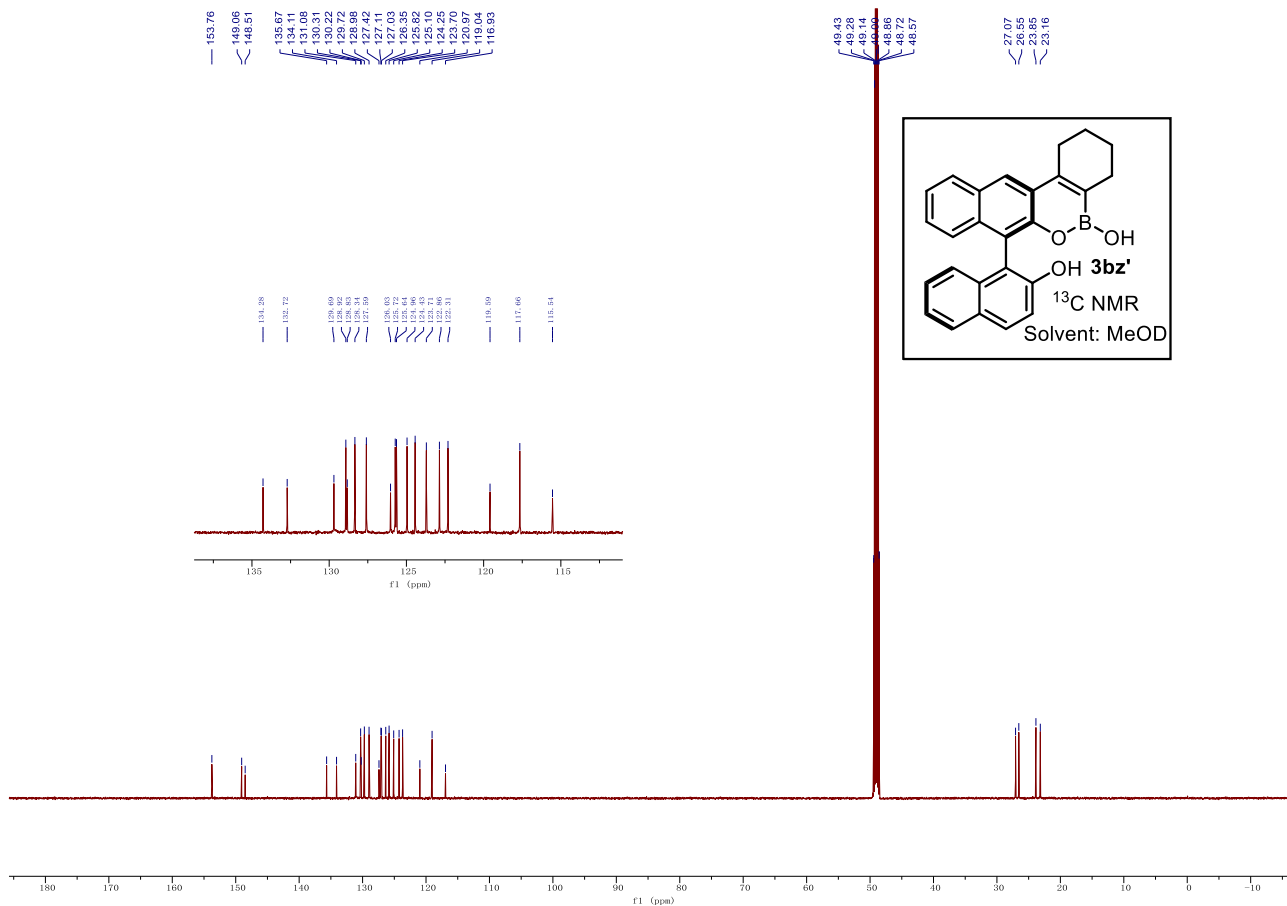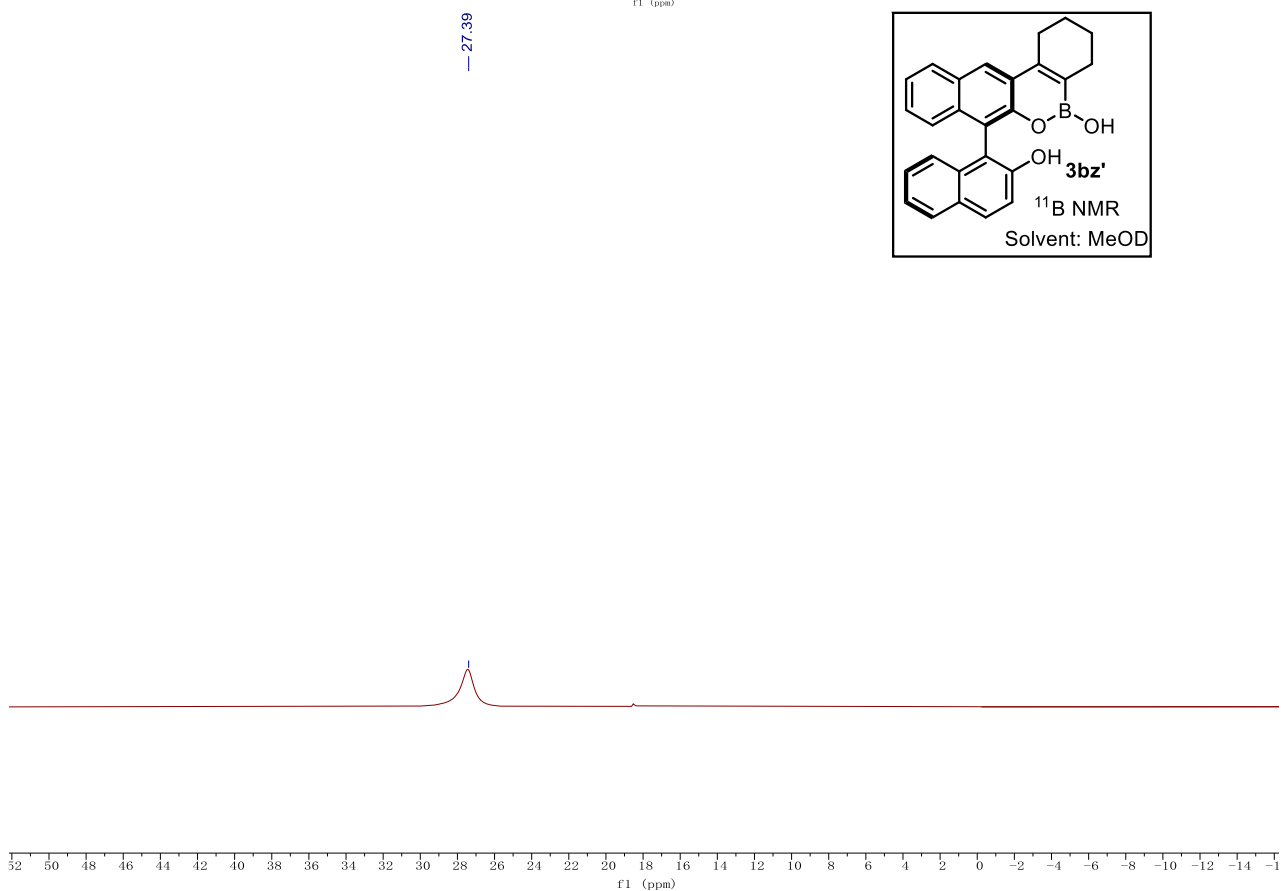

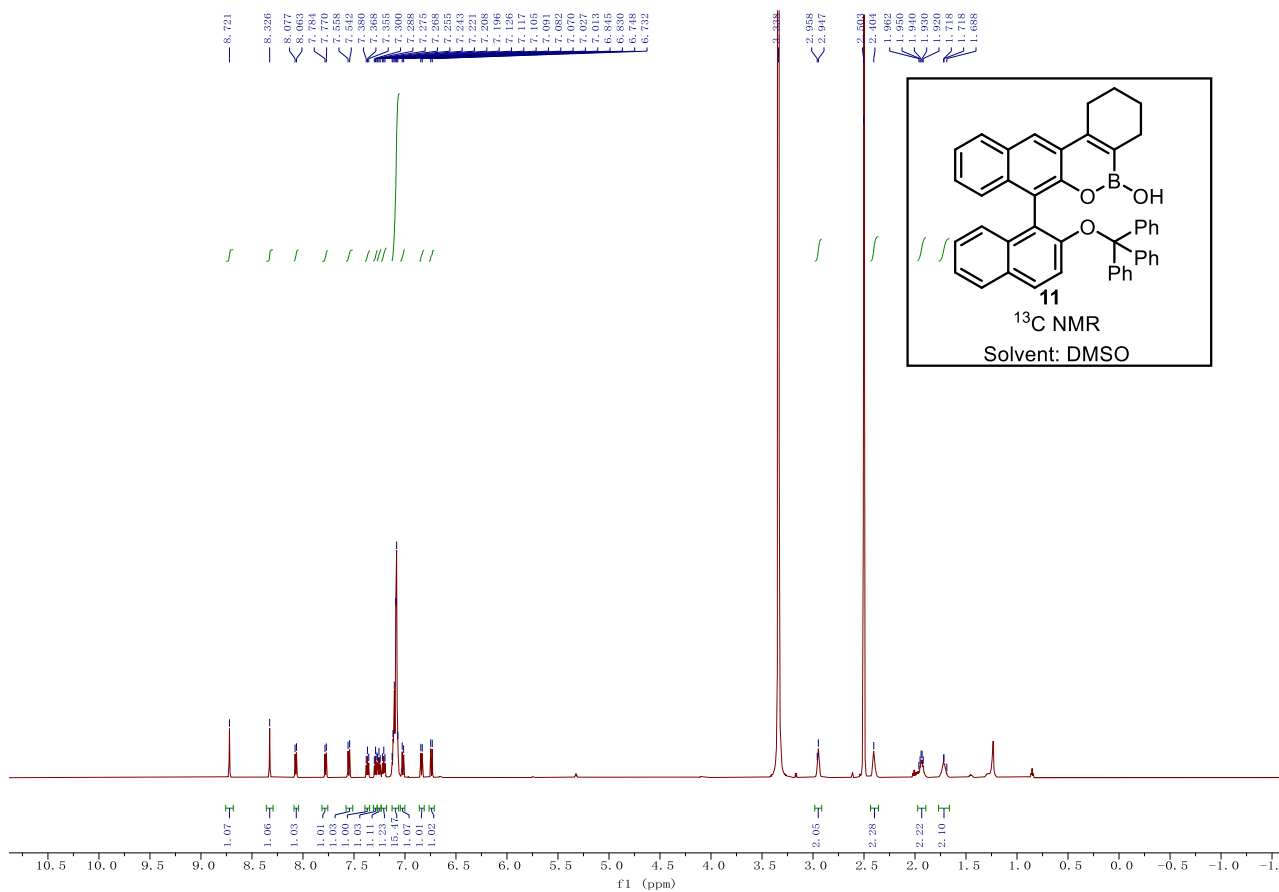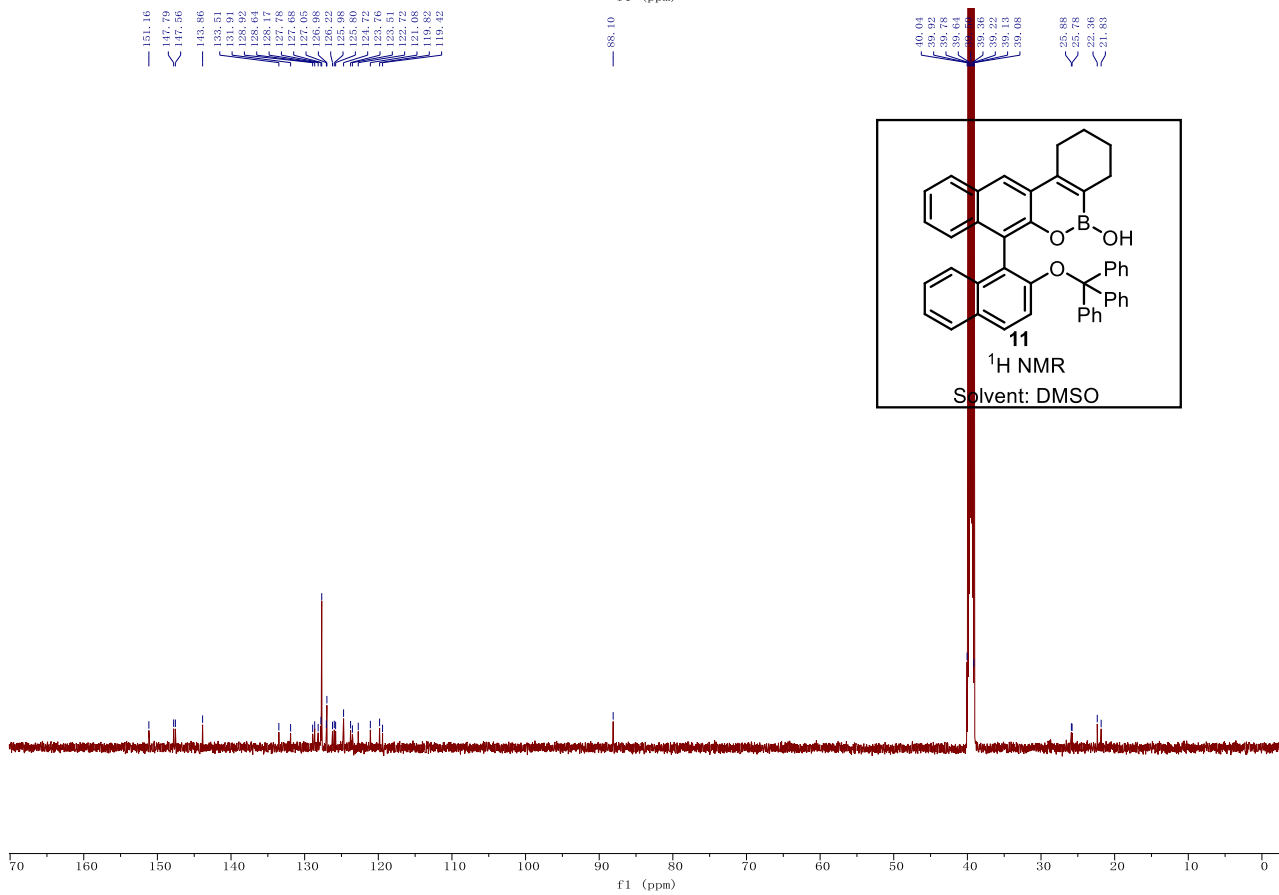

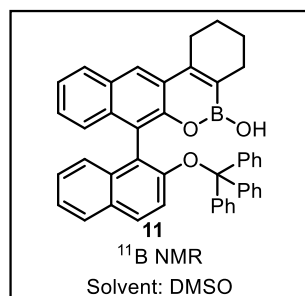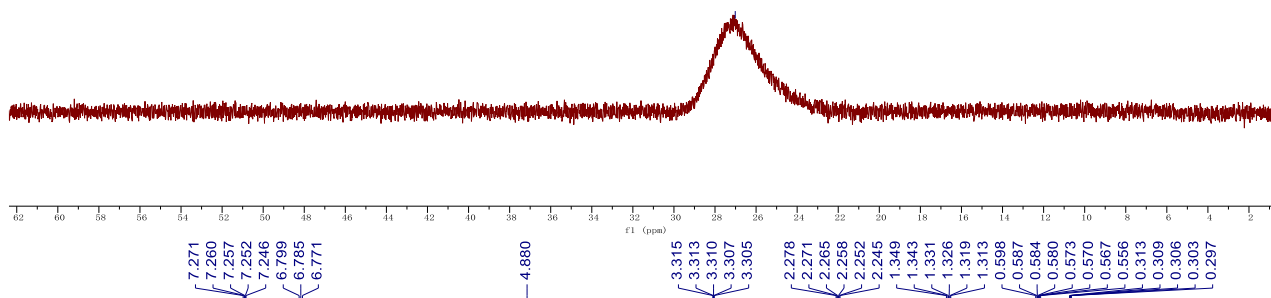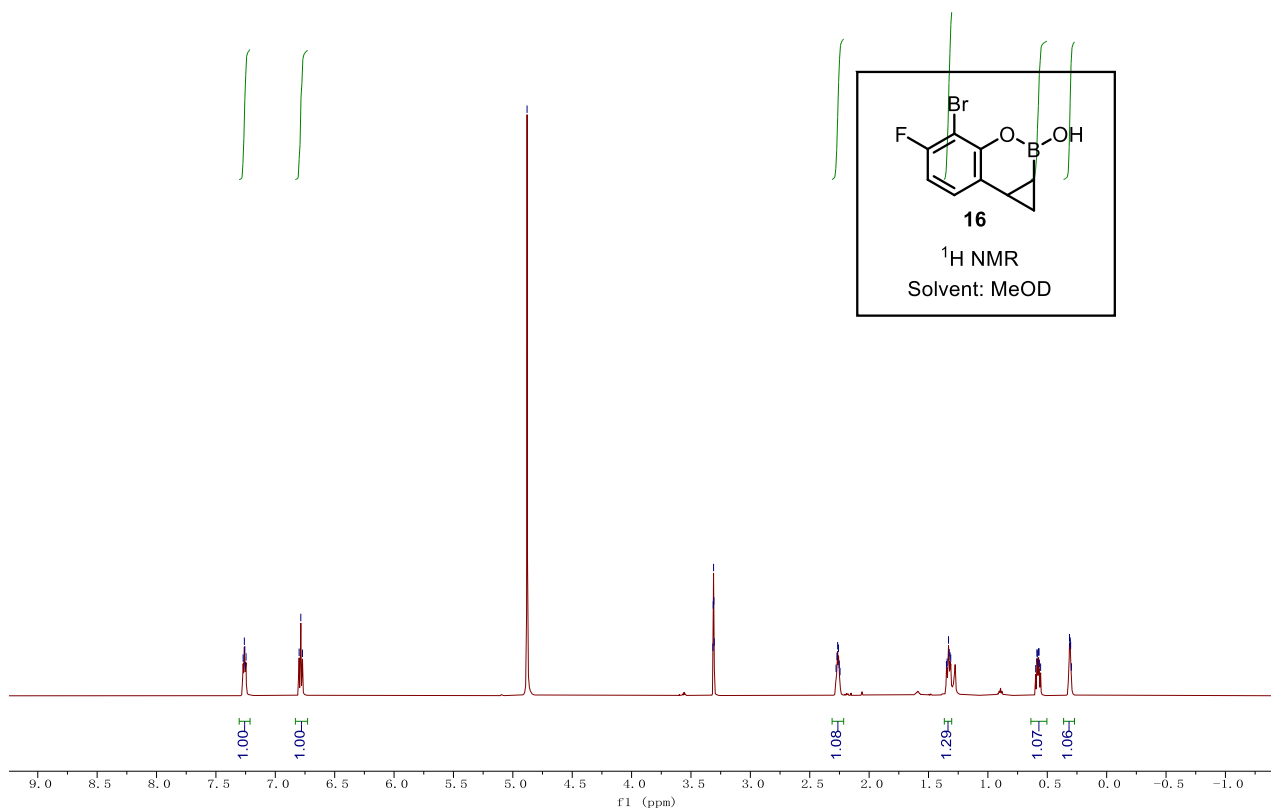

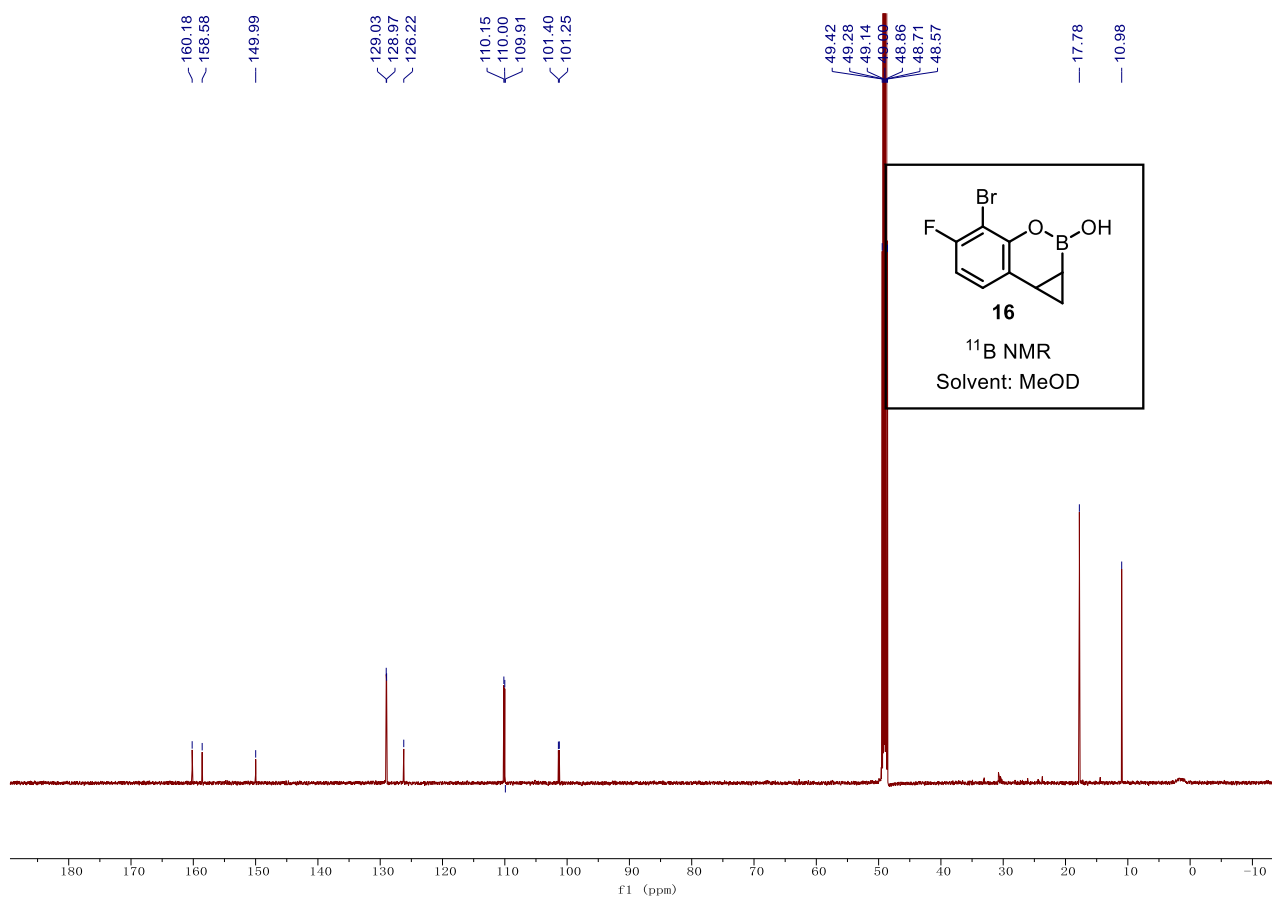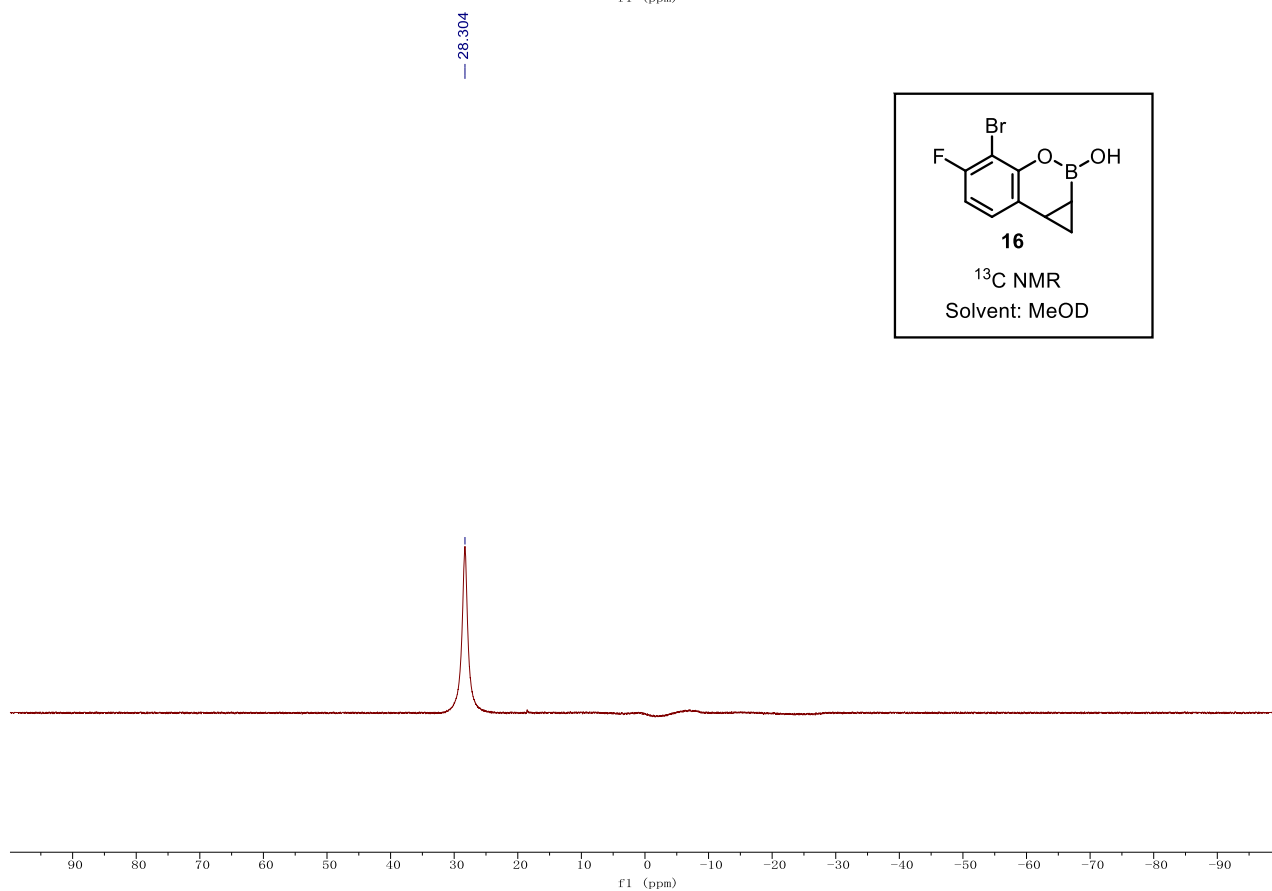

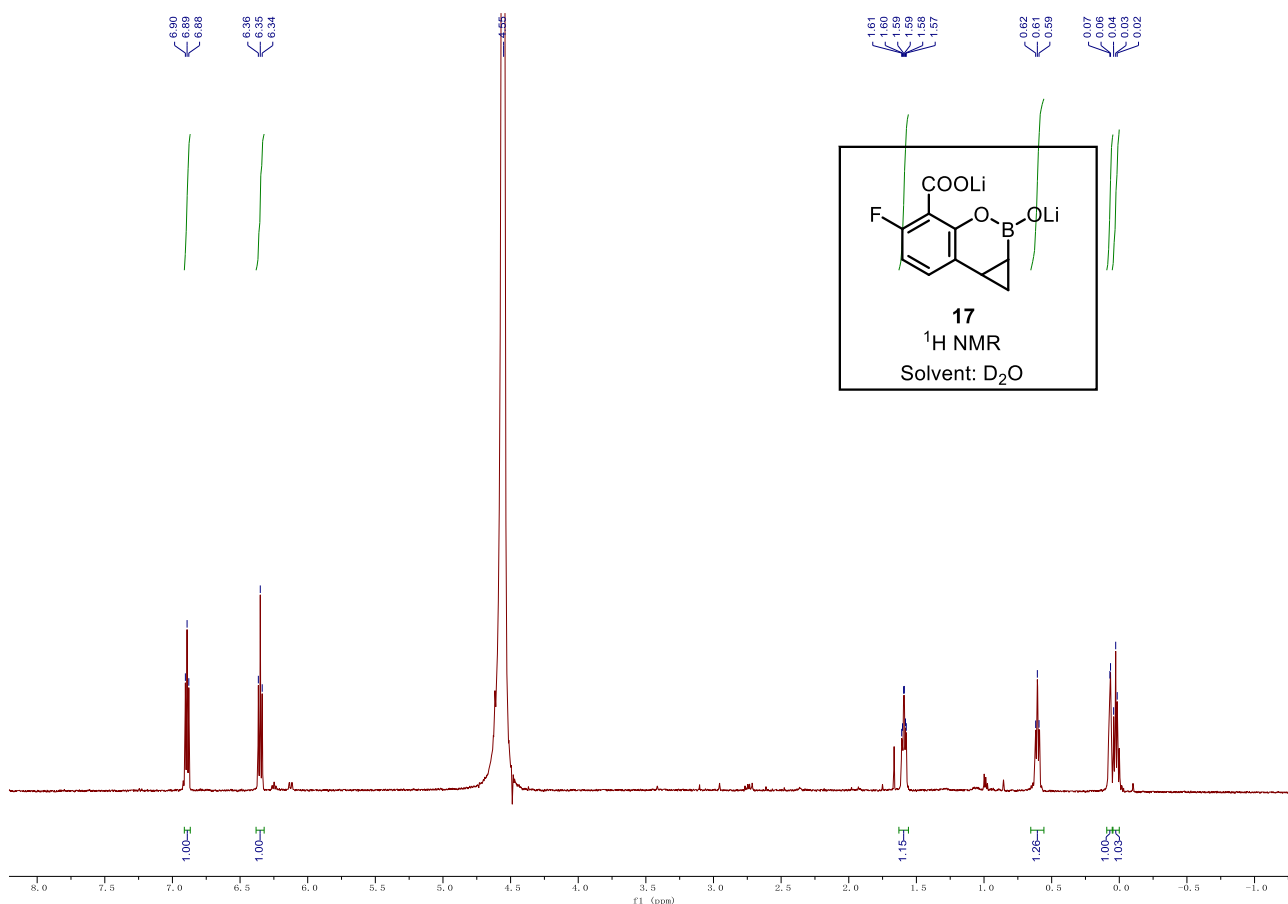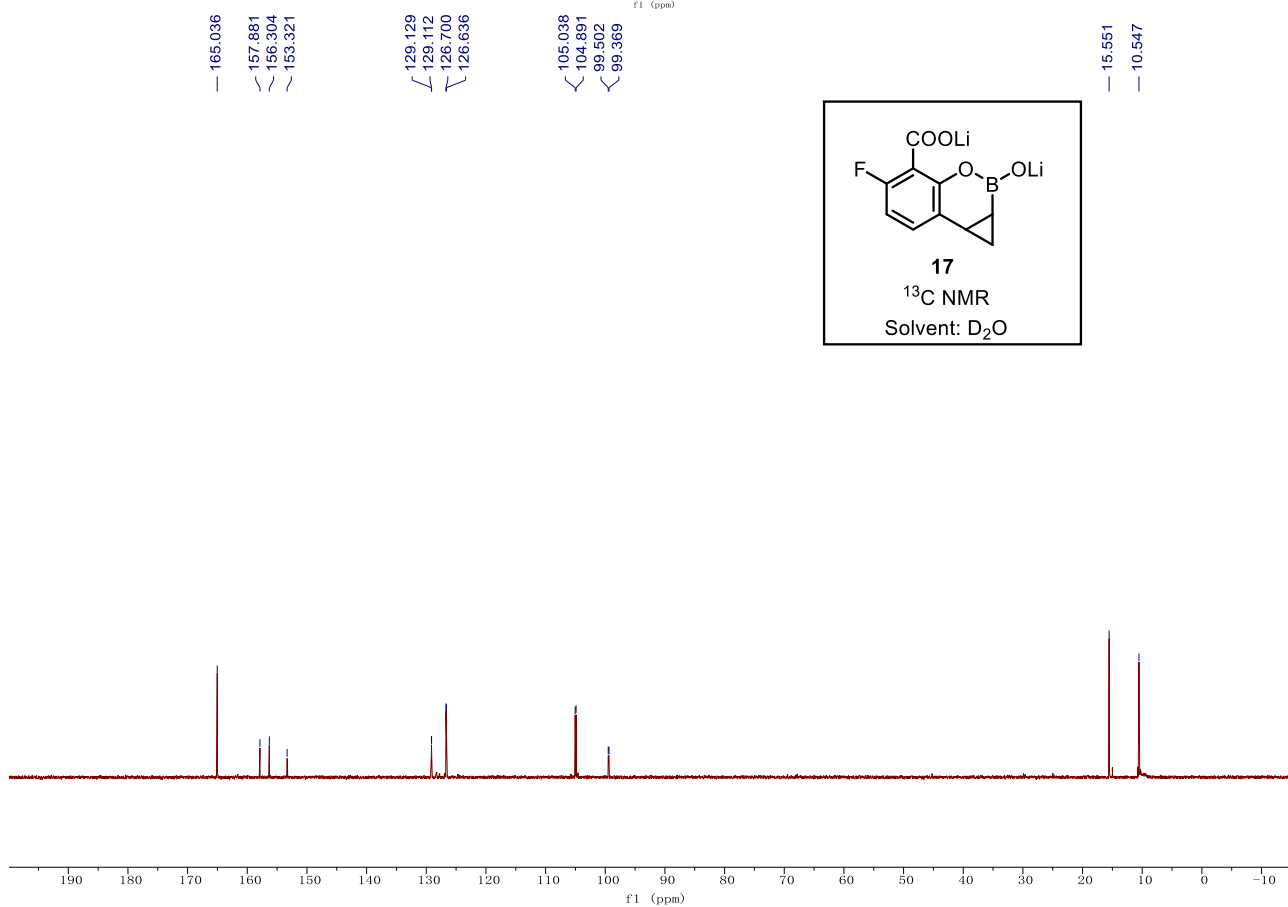

— 3.201

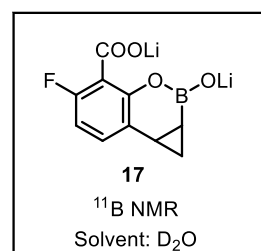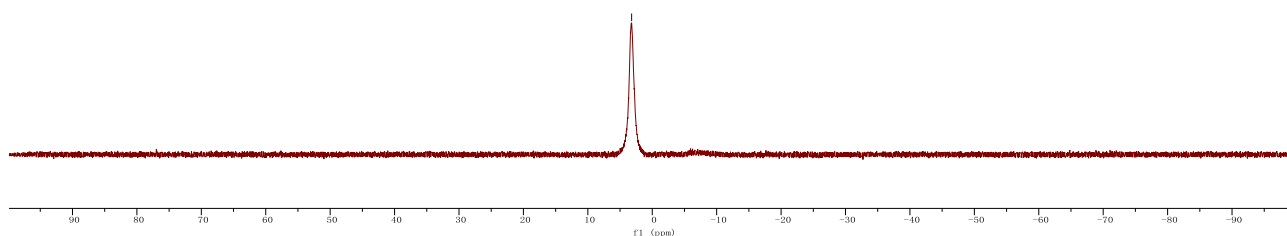

— -112.760

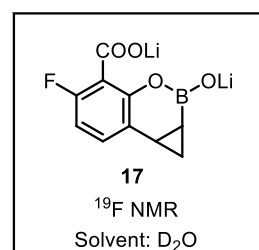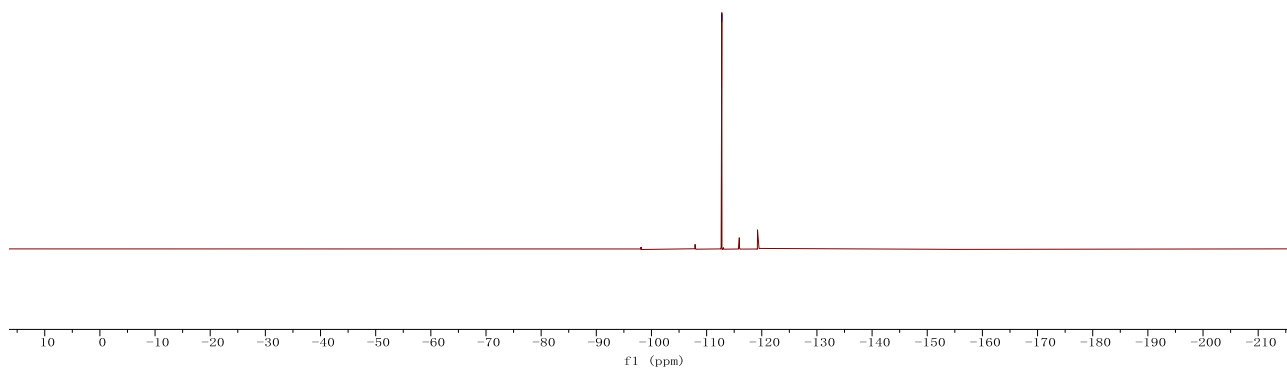

Supplement: Supplementary file 4 — Supplementary Data 1 [file 42004_2023_976_MOESM4_ESM.pdf]
